# C–H Insertion via Ruthenium Catalyzed *gem*-Hydrogenation of 1,3-Enynes

**DOI:** 10.1021/jacs.1c13446

**Published:** 2022-02-16

**Authors:** Sebastian Peil, Alejandro Gutiérrez González, Markus Leutzsch, Alois Fürstner

**Affiliations:** Max-Planck-Institut für Kohlenforschung, 45470 Mülheim/Ruhr, Germany

## Abstract

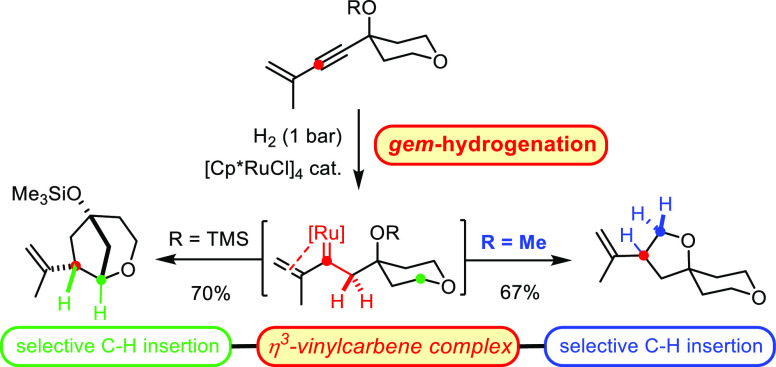

*gem*-Hydrogenation of an internal alkyne with the aid of [Cp*RuCl]_4_ as the precatalyst is a highly unorthodox transformation, in which one C atom of the triple bond is transformed into a methylene group, whereas the second C atom gets converted into a ruthenium carbene. In the case of 1,3-enynes bearing a propargylic steering substituent as the substrates, the reaction occurs regioselectively, giving rise to vinyl carbene complexes that adopt interconverting η^1^/η^3^-binding modes in solution; a prototypical example of such a reactive intermediate was characterized in detail by spectroscopic means. Although both forms are similarly stable, only the η^3^-vinyl carbene proved kinetically competent to insert into primary, secondary, or tertiary C–H bonds on the steering group itself or another suitably placed ether, acetal, orthoester, or (sulfon)amide substituent. The ensuing net hydrogenative C–H insertion reaction is highly enabling in that it gives ready access to spirocyclic as well as bridged ring systems of immediate relevance as building blocks for medicinal chemistry. Moreover, the reaction scales well and lends itself to the formation of partly or fully deuterated isotopologues. Labeling experiments in combination with PHIP NMR spectroscopy (PHIP = parahydrogen induced polarization) confirmed that the reactions are indeed triggered by *gem*-hydrogenation, whereas kinetic data provided valuable insights into the very nature of the turnover-limiting transition state of the actual C–H insertion step.

## Introduction

**Scheme 1:**
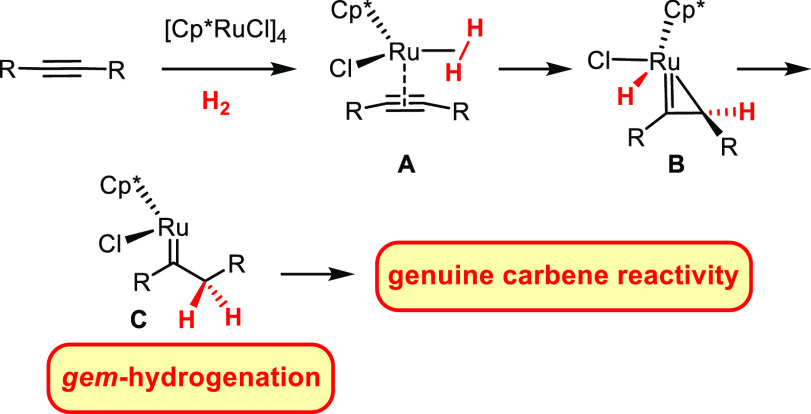
Concept of *gem*-Hydrogenation

The ability to transfer both H atoms of H_2_ to the same C atom of an internal alkyne is a fundamentally new reactivity mode that was discovered only recently (Scheme 1).^1^ The conversion of the receiving C atom into a methylene group is accompanied by the formation of a discrete metal carbene at the adjacent position. For the time being, such “*gem*-hydrogention” reactions have been accomplished with [Cp^X^RuCl] (or closely related metal fragments)^2,3^ as well as with [NHC(η^6^-cymene)RuCl_2_]; this latter system is photochemically driven and opens an unconventional entry into second-generation Grubbs-type catalysts.^4,5^ The use of [Cp^X^RuCl], in contrast, provides access to piano-stool ruthenium carbene complexes, the electrophilic character of which manifests itself in multifarious reactivity (Scheme 1):^6^ so far, it could be harnessed in the form of hydrogenative cyclopropanation,^3,7^ hydrogenative metathesis,^7,8^ hydrogenative heterocycle syntheses,^3,9^ hydrogenative ring expansion reactions,^3^ and hydrogenative rearrangements,^10−12^ all of which are conceptually new types of transformations; some of them are even counterintuitive when judged on the basis of conventional chemical logic.^13^

**Scheme 2:**
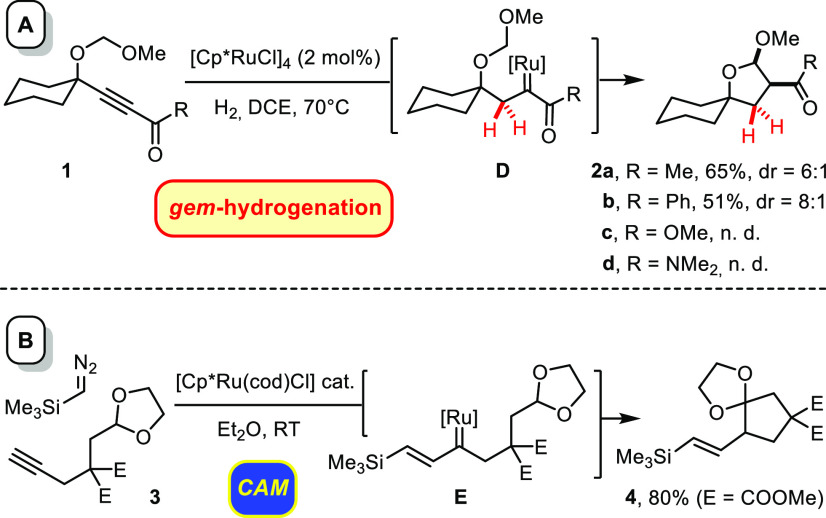
Lead Findings

It was during a recent application of hydrogenative metathesis to the total synthesis of the marine natural product sinularone F that C–H insertion was observed as yet another possibility for the transient carbene to evolve (Scheme 2A).^8^ However, this then undesired side reaction infringed only in cases such as **1**, in which the derived carbene complex **D** carried a neighboring ketone group. Even a flanking ester or dimethylamide did not suffice to upregulate the electrophilicity of the intermediate to the necessary extent; these compounds simply got reduced to alkene and alkane.^8,14^

Although these preliminary data spoke for a narrow window of opportunity, they sparked our interest. Additional motivation was drawn from a literature survey, which showed that certain piano-stool ruthenium carbenes generated by an entirely different route, namely, carbene/alkyne metathesis (CAM),^15−17^ are capable of inserting into *secondary* or *tertiary* C–H bonds of suitably disposed acetals or ethers in moderate to good yields (Scheme 2B);^18^ the reaction was carefully studied by computational means.^19−25^ If one were able to generate the presumed vinylcarbenes of type **E** by *gem*-hydrogenation, one could avoid the use of hazardous diazoalkanes altogether.^26^ At the same time, it might be possible to enlarge the scope of the reaction to a considerable extent, since, in practice, the CAM-based route had worked well only for the addition of *trimethylsilyl* diazomethane to *terminal* alkynes (Scheme 2B);^18,27^ a hydrogenative approach should not face such limitations.

At the outset of our project, however, the goal of establishing a reasonably general hydrogenative C–H insertion protocol seemed (over)ambitious: 1,3-enynes are known to bind very tightly to [Cp*Ru] fragments and had proven problematic in the past in various other reactions effected by such catalysts;^28−30^ it was therefore not clear whether they are amenable to *gem*-hydrogenation at all. Not only was this proved to be the case, but the ensuing C–H insertion reactions turned out to be truly enabling. Most notably, they open access to (spirocyclic) building blocks of immediate relevance for medicinal chemistry. In parallel, the gathered mechanistic information brings the understanding for this type of transformation to a new level.

## Results and Discussion

### Reaction Development and Control Experiments

**Scheme 3:**
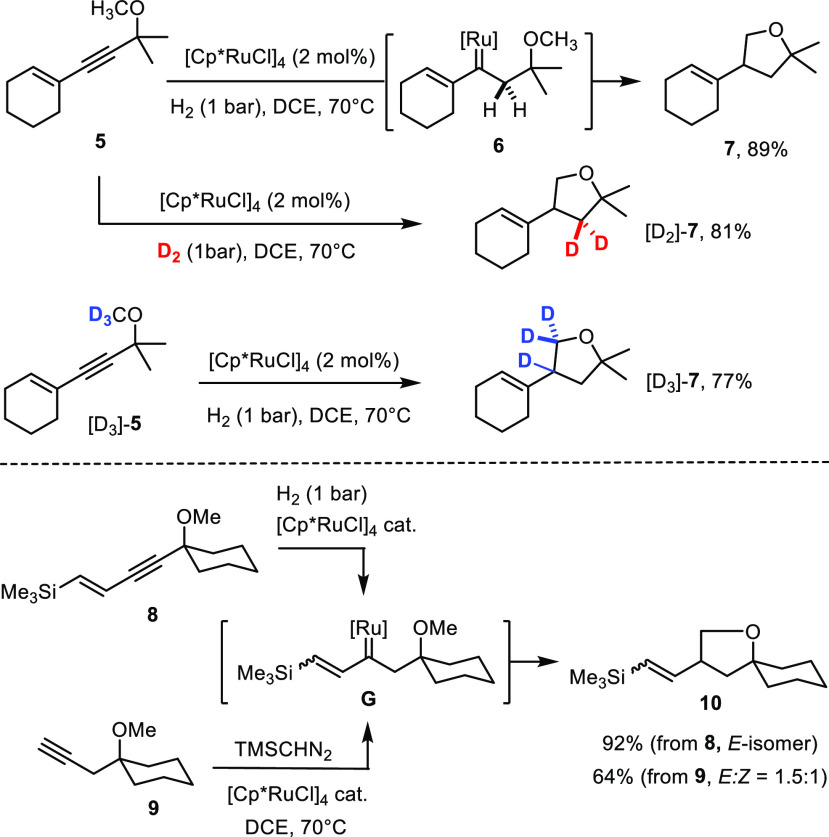
Proof-of-Concept and Control Experiments

All it took was to subject model compound **5** carrying a tertiary propargylic ether substituent to the conditions previously optimized for other *gem*-hydrogenation reactions in order to convert this substrate into the tetrahydrofuran derivative **7** in high yield (Scheme 3).^3,6,8^ [Cp*RuCl]_4_ proved to be the catalyst of choice;^31,32^ the cationic complexes [CpRu(MeCN)_3_]PF_6_ (43%) and [Cp*Ru(MeCN)_3_]PF_6_ (16%) turned out to be much less efficient and were not studied any further at this point.^33^ The reaction was best carried out under hydrogen atmosphere (1 bar) in 1,2-dichloroethane (DCE) as the solvent at 70 °C to ensure reasonable rates. This favorable result shows that (i) suitably functionalized 1,3-enynes are indeed amenable to *gem*-hydrogenation, (ii) the reaction proceeds regioselectively to generate the required vinylcarbene **6**, and (iii) this presumed intermediate is capable of inserting even into the *primary* C–H bond of the steering methyl ether. This latter aspect was deemed particularly encouraging since successful insertions of metal carbenes in general into aliphatic primary C–H bonds are rare^34−38^ and had been called a “remaining major challenge”.^39^ The specific literature precedent based on CAM had not reported any such example.^18^ This latter fact, however, might have solely been an oversight since CAM and *gem*-hydrogenation should pass through the same carbene. The direct comparison shown in Scheme 3 confirms this notion in that both reactions likely pass through a common intermediate **G** which evolves into the C–H insertion product **10**. At the same time, however, it reveals a first significant advantage of the novel hydrogenative approach: since product **10** derived from enyne **8** was obtained as a single compound, the sequence of *gem*-hydrogenation/insertion must have proceeded stereospecifically, whereas CAM converts substrate **9** into an inseparable mixture of double-bond isomers.^18^

Prior to exploring the scope of the reaction in more detail, several control experiments were carried out. Specifically, the labeled substrate [D_3_]-**5** furnished product [D_3_]-**7** exclusively. Even more compelling is the hydrogenation of unlabeled **5** with D_2_, which led to the *gem*-dideuterated tetrahydrofuran [D_2_]-**7** as the only detectable product. These results are in excellent agreement with a reaction sequence consisting of initial *gem*-hydrogenation followed by C–H insertion. The lack of scrambling speaks against any hidden mechanistic complexity along the reaction coordinate; therefore it is reasonable to assume that this method provides access to all possible isotopologues of **7** by proper combination of substrate (**5** versus [D_3_]-**5**) and reagent (H_2_ versus D_2_).

**Figure 1:**
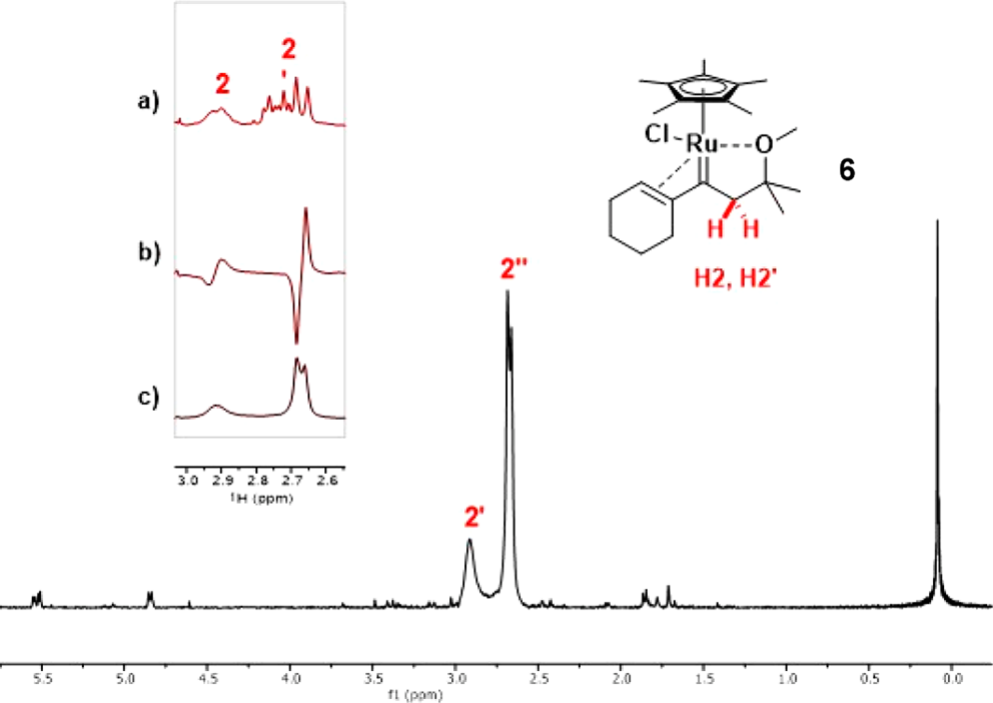
The “only parahydrogen spectrum (OPSY)” of the carbene intermediate **6** generated by *gem*-hydrogenaton of **5** using *p*-H_2_ shows massive signal enhancement indicative of pairwise delivery of the geminal hydrogen atoms to the same C atom. Inserts: (a) ordinary ^1^H NMR (methylene signals) of complex **6** formed using stoichiometric [Cp*RuCl] (see below); (b) PHIP hyperpolarized antiphase signal of the methylene group of **6** generated under catalytic conditions with *p*-H_2_; (c) signals of the OPSY spectrum of **6** for comparison.

In addition to the information gathered by analysis of the products, reactions performed with parahydrogen (*p*-H_2_) allowed the spectral fingerprints of the transient intermediates formed under catalytic conditions to be detected by virtue of the exceptional sensitivity of PHIP-enhanced ^1^H NMR spectroscopy (PHIP = *p*-H_2_-induced polarization) (Figure 1);^40−42^ the “only parahydrogen spectroscopy (OPSY)” pulse sequence proved particularly convenient.^43^ The appearance of the hyperpolarized antiphase signals characteristic of a diastereotopic methylene unit is contingent on pairwise delivery of both H atoms of H_2_ to the same C atom of the alkyne, which, in turn, implies a (largely concerted) *gem*-hydrogenation step (insert b). Stacking of the spectra of this transient intermediate with those of the carbene complex formed using stoichiometric amounts of [Cp*RuCl] proved identity.

### Scope

**Scheme 4:**
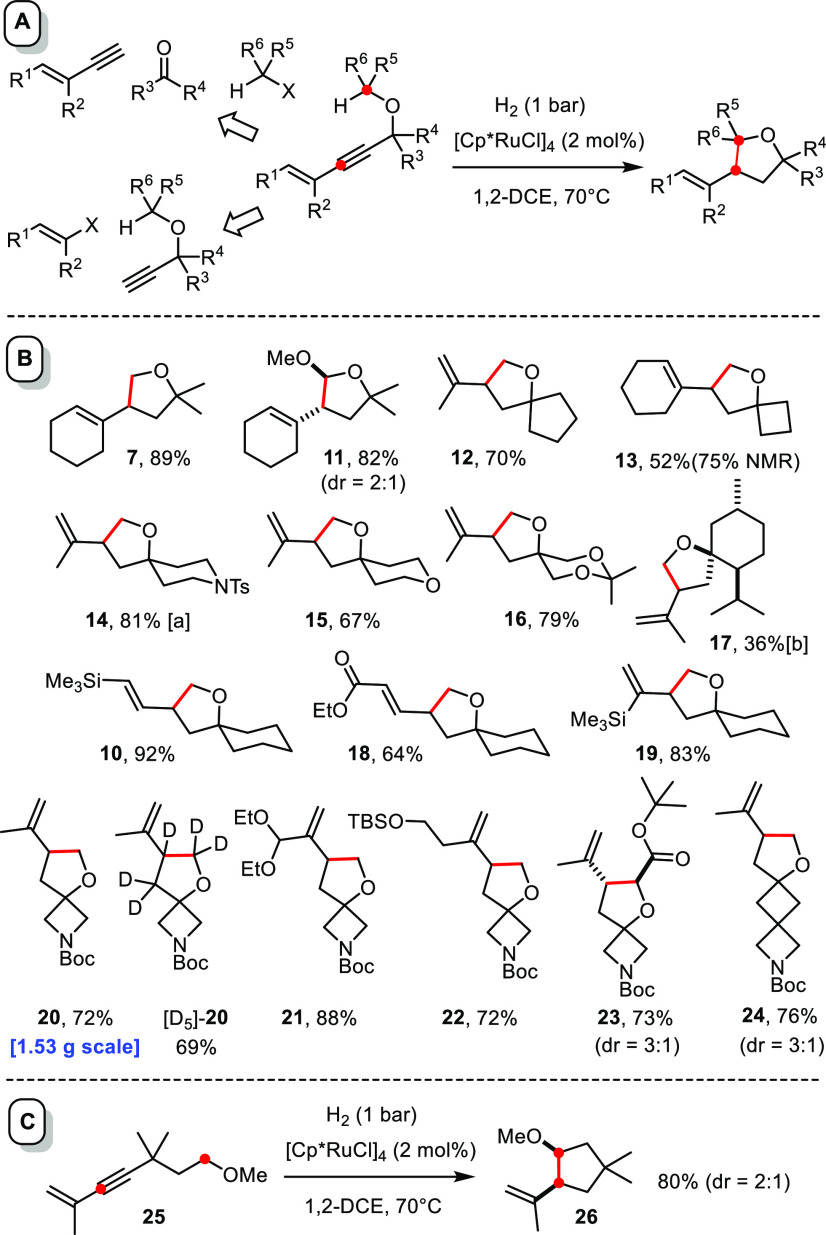
Hydrogenative C–H Insertion The newly formed C–C bond is highlighted in red. [a] The structure of this compound in the solid state is contained in the Supporting Information. [b] Single diastereomer of unknown stereochemistry at the propenyl branch.

A set of appropriate substrates was readily attained by one of two methods (Scheme 4A): (i) Sonogahira-type cross coupling of a propargyl alcohol derivative with a suitable alkenyl halide (sulfonate) or (ii) addition of a lithiated enyne to a ketone followed by (*in situ*) alkylation of the resulting alkoxide with the alkyl halide of choice (for details, see the Supporting Information).

Most of these enynes proved amenable to hydrogenative C–H insertion under standard conditions. It is important to note that many of the products shown in Scheme 4B would be difficult, if not even impossible, to obtain by the CAM-based route.^18^ The fact that preconfiguration of the alkene in the substrate allowed compounds such as **10** and **18** to be obtained in isomerically pure form has already been pointed out in the context of the control experiments discussed above. An even more significant advantage is the fact that products **12**, **14**–**17** and **19**–**24** would require hazardous diazomethane as carbene precursor if one were to use CAM for their synthesis, whereas the new route is simple, safe, and convenient. For this very reason, the reaction scales well, as illustrated by the preparation of **20**, which was obtained in virtually the same yield independent of whether 23 mg (70%) and 1.53 g (72%) of product were made. It is unnecessary to reiterate that this (and any other) compound can also be formed in partially or fully labeled format. Use of perdeuteromethyl iodide and D_2_ as two of the cheapest deuterium sources, for example, allowed us to make [D_5_]-**20**. Since labeled compounds are of eminent importance in medicinal chemistry and elsewhere, this facile, flexible, and if necessary, scalable entry is arguably significant.^44,45^

CAM struggles when it comes to using nonterminal alkynes, since delivery of the primary carbene derived from the diazo derivative is then typically regiounselective;^15,17,18^ once again, the new *gem*-hydrogenative approach has no problem in providing access to such products as amply illustrated by Scheme 4B. Moreover, compounds **7**, **11**, and **13** comprising a cyclic alkene moiety would be basically inaccessible via CAM.

Although the current study capitalized on insertions into the arguably most challenging primary C–H bonds of methyl ethers, (more activated) secondary and tertiary C–H bonds are also amenable to the reaction (see **11**, **23**, and Schemes 6 and 7). Particularly noteworthy in this context is the cyclization of compound **25**, in which the −OMe group is shifted away from the triple bond and the steering effect hence weak as had been shown in previous mechanistic studies.^28,29^ This aspect notwithstanding, a remarkably clean formation of cyclopentane **26** by kinetically favored regioselective insertion into the −CH_2_O– rather than the −OMe group was observed (Scheme 4C). This result suggests that the new method extends to carbocyclic rings and hence encourages further study (for further evidence, see the bicyclic products in Scheme 6).^46^

### Limitations

**Figure 2:**
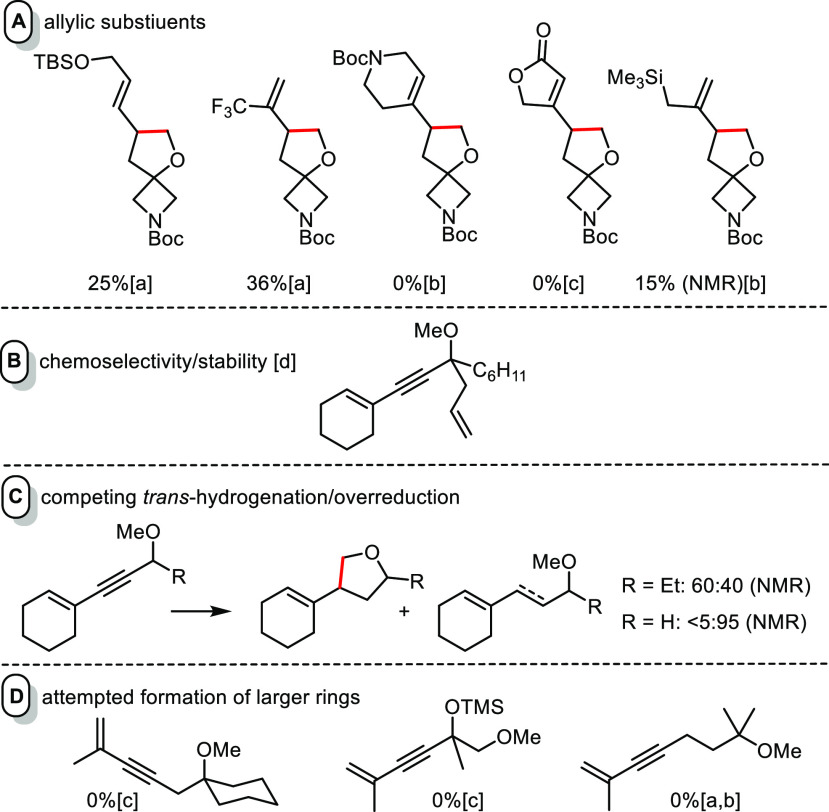
Representative examples illustrating current limitations of hydrogenative C–H insertion:(a) incomplete conversion; (b) complex mixture; (c) substrate recovered unchanged; (d) inseparable mixture of C–H insertion, cyclopropanation, and −OMe elimination products derived thereof

Limitations were encountered in case of substrates bearing allylic groups. Although the unsaturated acetal derivative **21** was obtained in excellent yield (Scheme 4), other compounds comprising an allylic silyl ether, ester, carbamate, or silane substituent reacted unselectively or even failed completely (Figure 2A). As functional groups of these types are compatible with the reaction otherwise, it must be the allylic placement that opens competitive pathways which consume the precatalyst and/or decompose the substrates.^47,48^ Unsurprisingly perhaps, insertion of a ruthenium carbene generated by *gem*-hydrogenation into the C–H bond of a methyl ether does not outcompete cyclopropanation of a suitably placed olefin (Figure 2B).

Our previous investigations had shown that alkyne *gem*-hydrogenation is mechanistically linked to *trans*-hydrogenation as a particularly facile competing process. For *gem*-hydrogenation to prevail, a tertiary −OR group at the propargylic position of the substrate is usually necessary.^1−3^ In line with this notion, enynes with secondary or primary propargylic ether substituents furnished product mixtures or were subject to *trans*-reduction only (Figure 2C).

The massive bias toward five-membered ring formation is a well-known hallmark of aliphatic C–H insertion in general.^49−55^ Indeed, first attempts at closing larger cycles by *gem*-hydrogenation have so far met with failure (Figure 2D). The same is true for intermolecular reactions; in assessing this aspect, however, one has to keep in mind that effective intermolecular trapping of vinylcarbenes generated via CAM by any reaction partner is basically unknown.^15−17^

### The Acetal and Orthoester Series

**Scheme 5:**
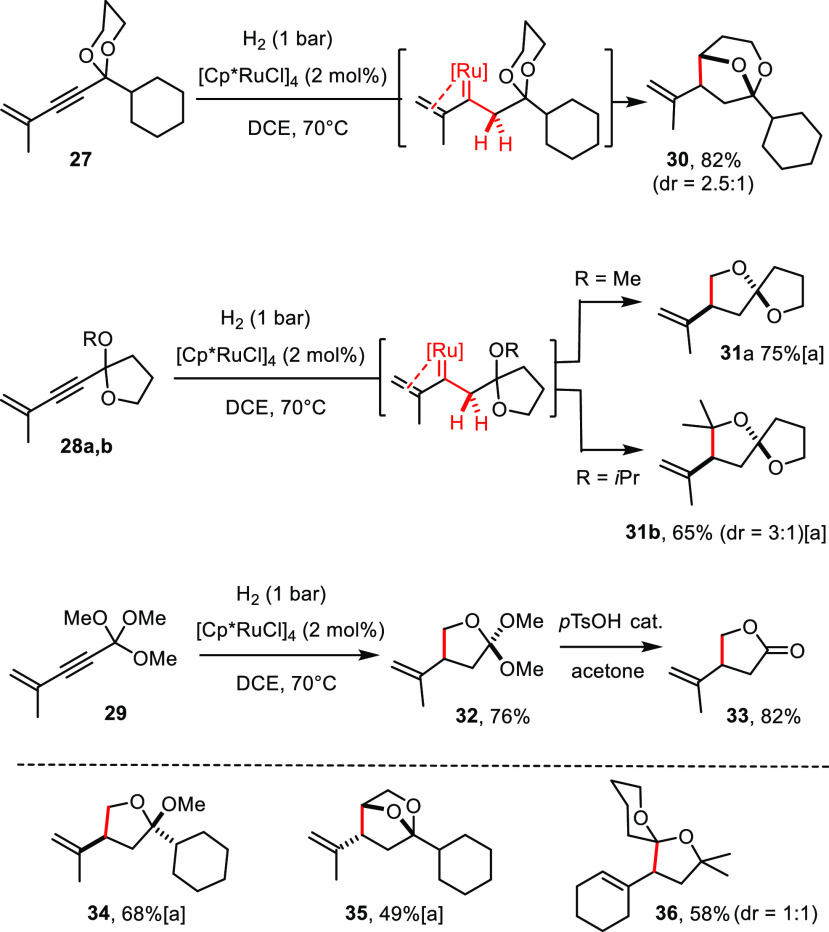
Hydrogenative C–H Insertion of Acetals and Orthoesters The product is acid sensitive.

A noteworthy extension of *gem*-hydrogenation pertains to enynes in which the steering substituent is part of an acetal or orthoester rather than a simple ether. Scheme 5 shows different ways of how the compliance of such substrates can be harnessed. Of arguably highest significance is the ability to form spiroketals from lactones in two straightforward and high-yielding steps via enyne addition/alkylation followed by *gem*-hydrogenative C–H insertion (see compounds **31a**,**b** and **36**); we are unaware of any precedent for this approach. Importantly, however, the concept is not limited to the formation of spirocycles, since bridged arrays such as **30** and **35** are equally within reach. When applied to an orthoester, the reaction provides access to butanolide derivatives such as **33** upon hydrolysis of the ortholactone **32** primarily formed.

### C–H Insertion Selectivity

These examples show that the insertion into primary, secondary, and tertiary C–H bonds proceeds with similar efficiency. It was therefore interesting to study whether substrates comprising more than one potentially reactive site are amenable to regioselective C–H insertion.

**Scheme 6:**
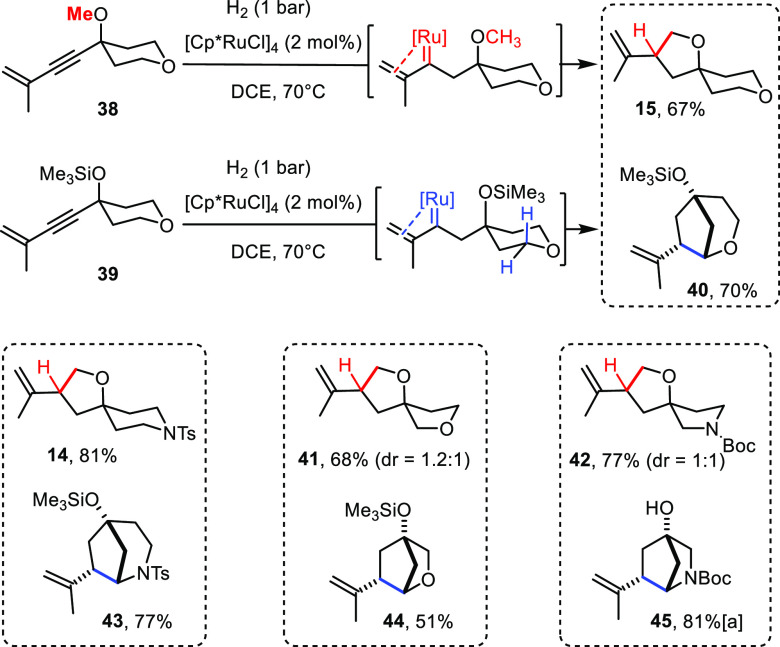
Structural Diversity by Site-Selective C–H Insertion After desilylation of the crude product with TBAF.

At first sight, the *gem*-hydrogenation with formation of product **31b** seems to follow the conventional order of C–H insertions^35−39^ because the weaker tertiary C–H bond of the R = O*i*Pr group rather than the endocyclic −OCH_2_– unit has reacted (Scheme 5). The analogous substrate **28a** (R = OMe) leads to the same spirocyclic scaffold in **31a**: it is important to note that this outcome mandates a drastic switch in selectivity in that exclusive insertion into the a priori less activated primary C–H bond of the methyl group must have taken place. This striking dichotomy suggests that the course of the reactions cannot be rationalized on thermodynamic grounds. In line with this notion, enyne **38** afforded spirocycle **15** as the only detectable product (Scheme 6), once again by exclusive insertion of the transient ruthenium carbene into the primary C–H bond of the steering −OMe substituent. The analogous reactive intermediate derived from **39**, however, gave rise to the bridged bicycle **40** in similar yield. This result proves that there is no inherent problem in engaging the methylene group of the pre-existing heterocycle into bond formation.^56^

**Figure 3:**
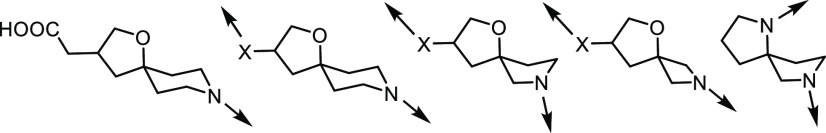
Spirocyclic scaffolds commonly used in medicinal chemistry.

Taken together, these results suggest that the observed selectivities are largely kinetic in origin, which, in turn, implies that the actual C–H insertion step must have a strong steric component to it, likely as a result of the bulky ancillary Cp* ligand. The C–H bond of a “tangling” and hence freely rotatable −OR substituent will align faster with the reactive [C=Ru] unit than a slightly more rigid cyclic array. In any case, the ability to produce noticeably different skeletons from a single precursor solely by switching a protecting group, as manifested in the couples **15**/**40**, **14**/**43**, **41**/**44**, and **42**/**45**, is deemed a significant asset (Scheme 6). Scaffolds of these types are prominently featured in contemporary medicinal chemistry as manifested in innumerous patents; the examples shown in Figure 3 are representative.

### Propargyl Amides

**Scheme 7:**
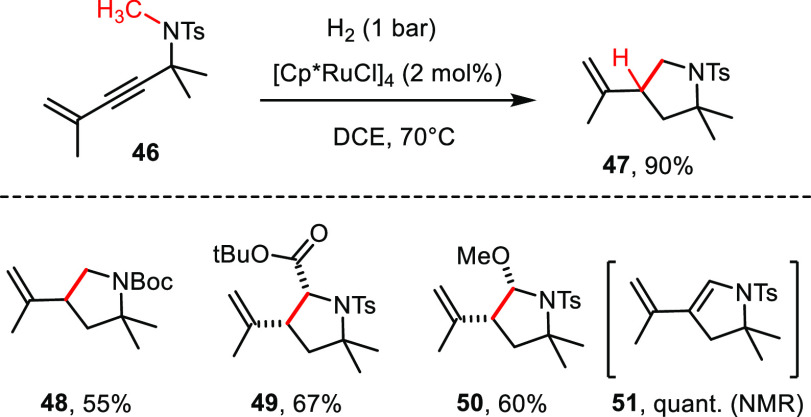
Formation of Pyrrolidine Derivatives by *gem*-Hydrogenation

As expected, the scope of the reaction extends beyond propargyl ethers and acetals (Scheme 7). Specifically, *tert*-amide derivatives such as **46** proved well behaved, even though the very nature of the amide group does affect the yield of the resulting pyrrolidine derivative (compare **47**/**48**).

The exclusive formation of the *cis*-configured compounds **49** and **50** is another remarkable feature; actually, product **49** is remotely related to kainic acid and related neuroexcitatory agents, the structures of which have been edited in countless ways.^57^ Hemiaminal **50** is a valuable *N*-sulfonyliminium ion surrogate. In the absence of external nucleophiles, catalytic HCl converts it into the functionalized 1,3-diene building block **51** in readiness for use in Diels–Alder cycloadditions. In line with the results outlined above, a Thorpe–Ingold effect in the substrate is also necessary in this series to ensure efficient C–H insertion.^58^

### Exemplary Downstream Functionalization

During the past decade, small ring systems in general and spirocyclic scaffolds in particular gained prominence as novel types of building blocks for medicinal chemistry.^59−63^ Replacement of traditional flat (hetero)aromatic cores of drug candidates by three-dimensional and sp^3^-rich templates can be largely beneficial: if properly chosen, they ensure optimal display of attached functionality toward reciprocal groups in the binding site of the targeted biological receptor; moreover, they provide potential advantages with regard to metabolic stability, often lead to reduced lipophilicity as compared to (hetero)arenes, open uncommon or even uncharted chemical and pharmacological space, and hence provide many opportunities for innovation and therapeutic advances.^59−63^

**Scheme 8:**
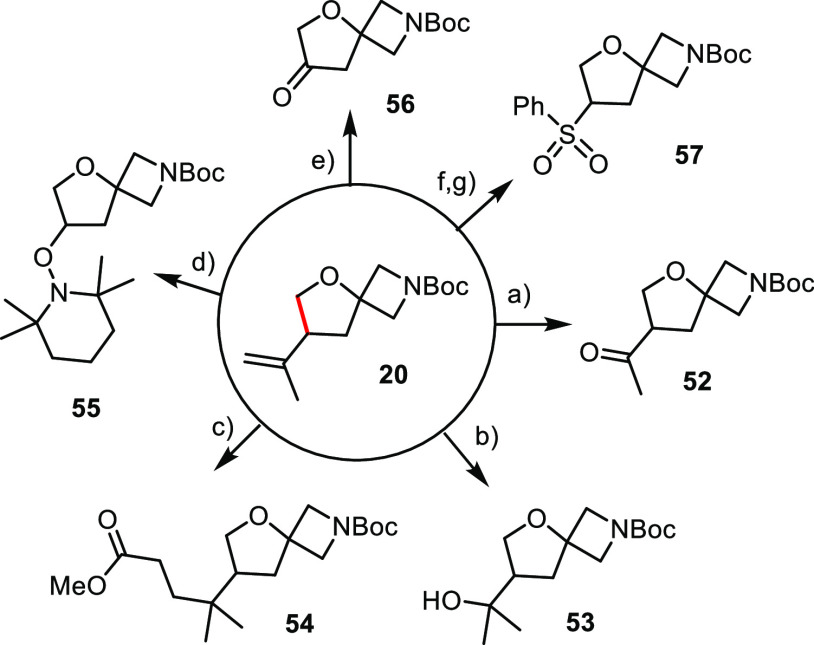
Downstream Functionalization Reagents and conditions: (a) (i) O_3_, CH_2_Cl_2_, −78 °C; (ii) Me_2_S, −78 °C → RT, 76%; (b) Co(acac)_3_ (25 mol %), PhSiH_3_, O_2_, THF, 69%; (c) Fe(acacc)_3_, PhSiH_3_, methyl acrylate, 1,2-dichloroethane, ethylene glycol, 60 °C, 74%; (d) (i) O_3_, MeOH, −78 °C; (ii) TEMPO, FeSO_4_·7H_2_O, −78 °C → RT, 63%; (e) (i) O_3_, MeOH, −78 °C; (ii) TEMPO, FeSO_4_·7H_2_O, magnesium bis(monoperoxyphthalate) hexahydrate (MMPP), −78 °C → RT, 62%; (f) (i) O_3_, MeOH, −78 °C; (ii) PhSSPh, −78 °C → 0 °C; FeSO_4_·7H_2_O; (g) *m*CPBA, CH_2_Cl_2_, 45% (over both steps).

The ease with which *gem*-hydrogenation brings such compounds into reach even on a larger scale encouraged us to briefly explore their downstream functionalization. Compound **20** was chosen as the model substrate since its isopropenyl substituent provides a versatile handle (Scheme 8). While the cleavage of the double bond by ozonolysis with formation of **52** is an obvious possibility, some other transformations are more involved.^64,65^ Specifically, a cobalt-catalyzed hydration furnished the tertiary alcohol derivative **53**; rather than trapping the transient radical with oxygen, an intermediate of this type can also be engaged in 1,4-addition reactions to, for example, ethyl acrylate as illustrated by the formation of **54**.^66^ The other entries illustrate the possibility of iron mediated dealkenylative oxidation with formation of either the valuable TEMPO-adduct **55** or ketone **56**.^67,68^ Sulfone **57** further illustrates the structural and functional diversity accessible from a single such platform.^69,70^ In this context, it is of note that compound **56** is a commercially available yet expensive building block,^71−73^ which the new route is able to deliver on scale and, if desirable, in labeled format. Extrapolation of the chemistry shown in Scheme 8 to the other (spirocyclic) products containing isopropenyl (or related alkenyl) substituents described above should give access to a multitude of valuable scaffolds for medicinal chemistry and chemical biology.

### Reactive Intermediates

As mentioned in the Introduction, *gem*-hydrogenation empowers various types of transformations including cyclopropanation, metathesis, skeletal rearrangements, and various heterocycle syntheses;^1−12^ C–H insertion was late to appear on scene.^8^ In view of this history, the ease of the reaction, as manifested in the examples outlined above, is all the more striking. It hence seemed possible that the ability to insert into C–H bonds might be a peculiarity of *vinyl*carbenes or α-*oxo*carbenes, which had led to the initial discovery (see Scheme 2).^8^

**Scheme 9:**
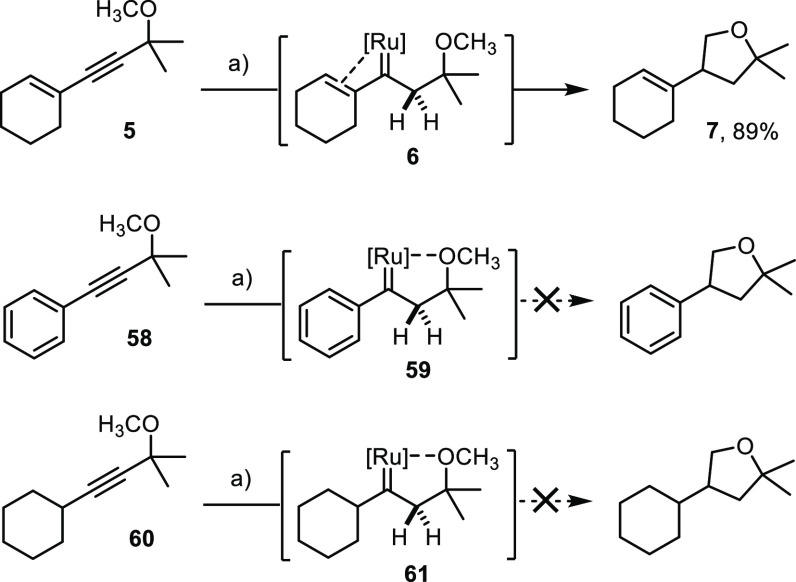
C–H Insertion Downstream of *gem*-Hydrogenation Is Contingent on the Presence of a Vinylcarbene Reagents and conditions: (a) [Cp*RuCl]_4_ (2 mol %), H_2_ (1 bar), 1,2-dichloroethane, 70 °C.

The comparison of substrates differing only in the degree of unsaturation of the flanking ring confirmed this notion (Scheme 9): only compound **5** which gives rise to the transient piano-stool ruthenium vinyl carbene intermediate **6** furnished the corresponding tetrahydrofuran derivative **7**; the two other compounds pass through the “ordinary” piano-stool carbenes **59** and **61** that proved incompetent in C–H insertion; rather, they evolve into the corresponding *trans*-alkenes and partial over-reduction products (for details, see the Supporting Information).^3^

To gain better understanding for why that is so, the *gem*-hydrogenation of **5** was repeated with a stoichiometric amount of [Cp*RuCl], as this might allow the structure and reactivity of the resulting vinylcarbene intermediate **6** to be studied in more detail. The reaction proceeded smoothly at 0 °C; the resulting crude material consisted of ∼85% of the expected complex as judged by NMR. The fact that the *gem*-hydrogenation occurs rapidly even at this low temperature whereas all catalytic reactions described above required gentle heating suggests that the turnover-limiting step must be later in the catalytic cycle (see below).

**Scheme 10:**
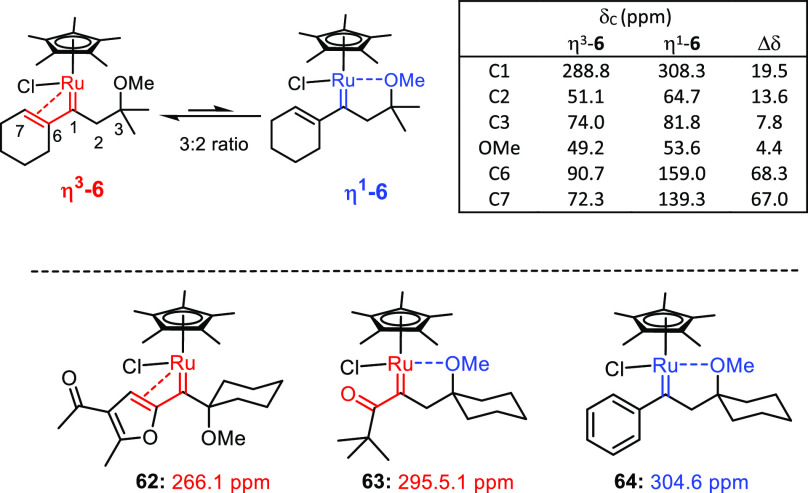
Spectral Data of the Carbene Intermediate: Comparison with Literature Data

Complex **6** proved too unstable for isolation in crystalline form, but the spectral data are highly informative (Scheme 10). Specifically, two different isomers are present in solution in a ratio of ≈3:2; the barrier for interconversion is on the order of only ≈10.4 kcal·mol^–1^ as deduced by VT-^1^H NMR spectroscopy (for details, see the Supporting Information). At −80 °C, both forms are frozen out and all relevant signals well resolved to allow for an unambiguous assignment.

The slightly preferred species is distinguished by η^3^-binding of the vinylcarbene unit to the ruthenium atom (η^3^-**6**). This coordination mode is manifested in the characteristic ^13^C NMR signals of the bound alkene (C6, C7) at δ_C_ = 90.7, 72.3 ppm; an upfield shift of almost 70 ppm relative to the alkene signals of the second isomer indicates a strong electronic communication between the π-bond and the metal center.^74^ The second isomer is an ordinary η^1^-vinylcarbene (η^1^-**6**), the double bond of which is slightly polarized but otherwise largely unperturbed by the neighboring carbene site as indicated by the resonances of C6 and C7 at δ_C_ = 159.0 and 139.3 ppm, respectively. Its ruthenium center likely reaches the 18e count by a supporting Lewis acid/base interaction with the ether moiety.^75^

The distinctly different shifts of the carbene centers of these two interconverting isomers are equally significant. With δ_C_ = 308.3 ppm, η^1^-**6** falls in the typical range previously observed for other piano-stool ruthenium carbene complexes with lateral Ru···O bonding such as **64**;^2,3^ no such derivative has been found competent in C–H insertion reactions. In sharp contrast, η^3^-**6** has the carbene resonance at δ_C_ = 288.8 ppm, which speaks for a notably different electronic character. In this regard, it is quite similar to the so far only structurally characterized α-oxocarbene complex **63** (δ_C_ = 295.5 ppm).^3^ The furyl carbene species **62**, the η^3^-binding mode of which has been crystallographically proven, shows an even stronger upfield shift (δ_C_ = 266.1 ppm);^9^ this complex is likely kept from undergoing intramolecular C–H insertion into the methyl ether substituent by the high strain of the four-membered ring that would ensue.

These experimental and spectroscopic data concur with the conclusions drawn by Saá and co-workers, who studied the fate of ruthenium vinylcarbene intermediates generated by CAM in silico.^19,20^ These authors suggested that the η^3^-bound isomer accounts for the downstream chemistry; it draws its higher reactivity from an out-of-plane distortion that causes an electronic perturbation and, at the same time, renders the site sterically more exposed.

### The Actual C–H Insertion Step

The ability to form **6** in fairly high purity allowed us to study the fate of this reactive intermediate by NMR spectroscopy. In the temperature range from +15 °C to +50 °C, the decay follows a first order rate law (Δ*G*^‡^ (25 °C) = 23 kcal·mol^–1^), as expected for an intramolecular C–H insertion (for details, see the Supporting Information). The experiments were repeated with [D_3_]-**6** carrying a perdeuterated methyl ether; comparison of the derived rate constants allowed the kinetic isotope effect (KIE) and its temperature-dependence to be accurately determined (Table 1; for the full data set, see the Supporting Information). With values on the order of 3.3–3.9, determined by measuring two separate rate constants, it is clear that C–H insertion occurs during the rate-determining step.^76,77^

**Table 1:**
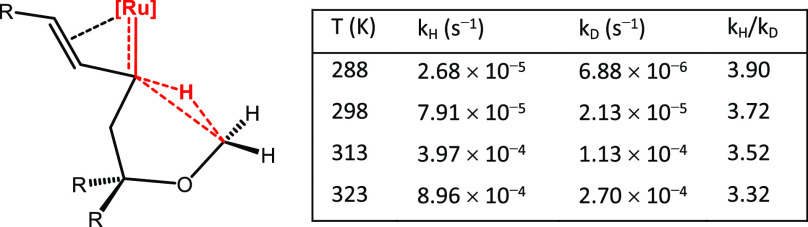
Kinetic Data for the Transformation of **6** or [D_3_]-**6** into **7** or [D_3_]-**7**, Respectively: Putative Transition State Connecting Vinylcarbene **6** and Product **7**

Importantly, these data also allow valuable information concerning the actual transition state (TS) to be deduced. In an insightful study, Kwart had analyzed four different extremes for three-center processes in general and found that each TS geometry has a characteristic footprint manifested in a data-triple consisting of the actual KIE (*k*_H_/*k*_D_, at 25 °C), the difference in activation energy [Δ*E*_A_], and the quotient of the pre-exponential terms of the Arrhenius equations (*A*_H_/*A*_D_).^78^ This formalism applies to the interaction between a carbene center and an incoming C–H bond. In the present case, the temperature-dependent KIE and a characteristic data-triple (*k*_H_/*k*_D_ (25 °C) = 3.72; [Δ*E*_A_] = 0.82 kcal·mol^–1^; *A*_H_/*A*_D_ = 0.94) are in excellent agreement with a linear, unsymmetrical H-transfer process. In other words, the electrophilic metal carbene center is attacked by the hydrogen atom, which develops hydridic character in a transition state distinguished by a very obtuse (in the extreme: linear) C–H–C angle (see the insert in Table 1).^79,80^ The recorded data rule out a more Dewar–Chatt–Duncanson-like scenario, in which the metal concomitantly interacts with the σ/σ* orbitals of the C–H bond, which would require a “bent” geometry (in the extreme a coplanar orientation of the carbene and the C–H bond to be broken); in such a case, the KIE is expected to be basically temperature-independent.^78^

Once again, the conclusions drawn from our experimental data tally well with the computational results of Saá and co-workers, who suggested that the vinylcarbene species formed by CAM evolve via a “hydride transfer” mechanism.^19,20^ In addition, our own computations of the C–H insertion pathway populated by ruthenium α-oxocarbene complexes such as **D** (Scheme 2) showed a very obtuse angle between the carbene center and the incoming reaction partner.^81^

## Conclusions

*gem*-Hydrogenation is a conceptually novel mode of H_2_ transfer to an organic substrate, which our group was able to discover after a century of intense research devoted to catalytic hydrogenation in innumerous academic as well as industrial laboratories. The present study showed that 1,3-enynes bearing an appropriate propargylic substituent are amenable to this process using [Cp*RuCl]_4_ as the catalyst. The resulting piano-stool ruthenium vinylcarbene intermediates adopt interconverting η^1^- and η^3^-binding modes, which are easily distinguished by virtue of their markedly different spectral fingerprints. Although these two forms are similarly stable, only the η^3^-isomer is competent to insert into primary, secondary, or tertiary C–H bonds of suitably disposed ethers, acetals, or N-alkylated (sulfon)amide derivatives. The ensuing reaction is highly enabling in preparative terms; most notably, it provides ready access to spirocyclic as well as bridged ring systems of immediate relevance as building blocks for medicinal chemistry and chemical biology. The method scales well and lends itself to the preparation of deuterated isotopologues. This novel hydrogenative C–H insertion process hence provides a notable addendum to the growing list of reactions exploiting *gem*-hydrogenation as a means to generate reactive intermediates and augurs well for further explorations of this field of research.

## Supporting Information Available

Experimental Section containing supporting NMR data (PDF)

## Accession Codes

CCDC 2106229 contains the supplementary crystallographic data for this paper. These data can be obtained free of charge via www.ccdc.cam.ac.uk/data_request/cif, or by emailing data_request@ccdc.cam.ac.uk, or by contacting The Cambridge Crystallographic Data Centre, 12 Union Road, Cambridge CB2 1EZ, UK; fax: +44 1223 336033.
