# Supplementary material for: C–H Insertion via Ruthenium Catalyzed gem-Hydrogenation of 1,3-Enynes
Source: J Am Chem Soc. 2022 Feb 16;144(9):4158–67. doi: 10.1021/jacs.1c13446 (PMC8915261; doi:10.1021/jacs.1c13446)
Supplement: Supplementary file 1 — ja1c13446_si_001.pdf [file ja1c13446_si_001.pdf]

SUPPORTING INFORMATION

C–H Insertion via Ruthenium Catalyzed *gem*-Hydrogenation of 1,3-Enynes

Sebastian Peil, Alejandro Gutiérrez González, Markus Leutzsch, and Alois Fürstner*

Max-Planck-Institut für Kohlenforschung, 45470 Mülheim/Ruhr (Germany)

E-mail: fuerstner@kofo.mpg.de

Contents

Supporting Crystallographic Information S2
General S3
Mechanistic Experiments S4
  Deuterium Labeling Experiments S4
  Carbene Complex 6 ....S6
    Variable Temperature NMR S13
    PHIP NMR S21
    C–H Insertion: Kinetic Analysis S22
    Temperature-Dependent KIE Analysis S27
  Catalytic KIE Experiments S33
  Comparison with Aliphatic and Benzylic Carbene Intermediates S35
  Independent Generation of a Catalytically Active Vinylcarbene Intermediate S35
Preparation of the Substrates S38
Hydrogenative C–H Insertion Reactions S55
Exemplary Downstream Functionalization S76
Copies of NMR Spectra S79
  Building Blocks S79
  Substrates for *gem*-Hydrogenation/C–H Insertion S88
  Products of *gem*-Hydrogenation / C–H Insertion S134
  Exemplary Downstream Functionalization S180
References S186

Supporting Crystallographic Information

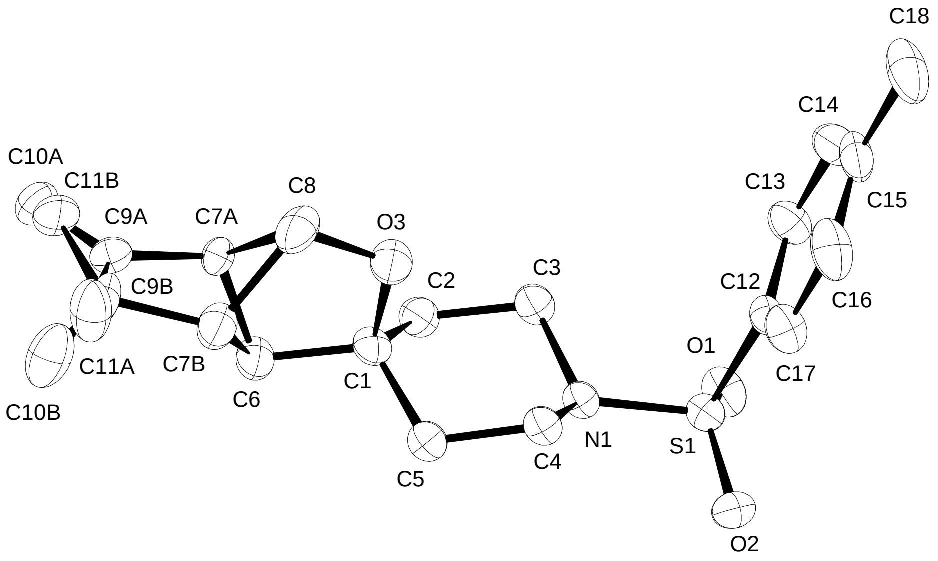

Figure S1. The structure of compound **14** in the solid state; H-atoms are omitted for clarity

Crystal data for Compound 14: $C_{18} H_{25} N O_3 S$, M = 335.45g mol^{-1}, colorless, crystal dimensions 0.14 x 0.06 x 0.03 mm, monoclinic P 2_1/c (no. 14), at 100(2) K a = 6.1272(3), b = 13.236(3), c = 21.559(3) Å , α = 90, β = 90.036(7), γ = 90°, V = 1748.4(4) Å^3, Z = 4, ρ = 1.274 Mgm^{-3}, μ = 0.199 mm^{-1}, λ = 0.71073 Å. X-ray diffraction data were collected using a Bruker AXS Enraf-Nonius KappaCCD diffractometer employing CCD and scans to cover reciprocal space up to 33.175° with 99.3% completeness, integration of raw data yielded a total of 27194 reflections, merged into 6639 unique reflections with Rint = 0.1149 after applying Lorentz, polarisation and absorption correction. The structure was solved by the dual method using SHELXT (Sheldrick, 2015), and atomic positions and displacement parameters were refined using full matrix least-squares based on Fsqd using SHELXL (Sheldrick, 2015). Refinement of 247 parameters using all reflections converged at R = 0.0743, wR = 0.1742, highest residual electron density peak 0.453 Å^3. Crystallographic data have been deposited with the Cambridge Crystallographic Data Centre and can be obtained free of charge by applying to: The Director, CCDC, 12 Union Road, Cambridge, CB2 1EZ, United Kingdom, quoting the reference no. **CCDC-2106229**.

General

Unless stated otherwise, all reactions were carried out under argon atmosphere in flame-dried Schlenk glassware. The solvents were purified by distillation over the indicated drying agents under argon: THF, Et_2O (Mg/anthracene), hexanes (Na/K), EtOH, MeOH (Mg), 1,2-dichloroethane, CD_2Cl_2, CH_2Cl_2 (CaH_2). DMF, DMSO, MeCN and Et_3N were dried by an absorbtion solvent purification system based on molecular sieves. 1,2-Dichloroethane (DCE), CD_2Cl_2 and CH_2Cl_2 were degassed via freeze-pump-thaw cycles (3 x) and stored over molecular sieves. Flash chromatography: Merck Geduran silica gel 60 (40 – 63 µm). TLCs were stained with $KMnO_4$, anisaldehyde, or molybdatophosphoric acid (5% in EtOH).

NMR spectra were recorded on Bruker AV III HD30, AMX 300, AV 400, AV III 500, AV III 600, or AVneo 600 spectrometers in the solvents indicated; chemical shifts are given in ppm relative to TMS, coupling constants (*J*) in Hz. The solvent signals were used as references and the chemical shifts converted to the TMS scale ($CDCl_3$: δ_C = 77.16 ppm; residual $CHCl_3$: δ_H = 7.26 ppm; CD_2Cl_2: δ_C = 54.00 ppm; residual $CHDCl_2$: δ_H = 5.32 ppm). Signal assignments are based on COSY, HSQC, HMBC and NOESY experiments.

PHIP NMR experiments were performed on a Bruker AVIII 500 MHz (11.7 T) NMR spectrometer equipped with a BBFO probe with z-gradient. OPSY spectra were recorded using the opsy-d pulse sequence.[1,2] *para*-Hydrogen enriched to 92% was freshly generated using a commercially available *p*-H_2 generator from Bruker BioSpin GmbH with an F–DGSi electrolytic hydrogen generator (WM.H2.500.V3) as the hydrogen source.

IR: Alpha Platinum ATR (Bruker), wavenumbers ($\tilde{\nu}$) in cm^{-1}.

MS (EI): Finnigan MAT 8200 (70 eV), ESI-MS: ESQ 3000 (Bruker), Thermo Scientific LTQ-FT or Thermo Scientific Exactive. HRMS: Bruker APEX III FT-MS (7 T magnet), MAT 95 (Finnigan), Thermo Scientific LTQ-FT or Thermo Scientific Exactive. GC-MS: Shimadzu GCMS-QP2010 Ultra instrument.

Hydrogen gas (N50, ≥99.999 vol%) was purchased from AirLiquide and was used without further purification. Deuterium gas (99.8 atom% D, 99.995% purity) was purchased from SigmaAldrich. Both H_2 and D_2 were handled with standard balloon techniques.

Unless stated otherwise, all commercially available compounds (abcr, Acros, TCI, Aldrich, Alfa Aesar) were used as received. The ruthenium complex [Cp*RuCl]$_4$ was prepared according to the literature procedure.[3] The building blocks required for the synthesis of the different substrates were prepared according to the cited literature (see below).

Mechanistic Experiments

Deuterium Labeling Experiments

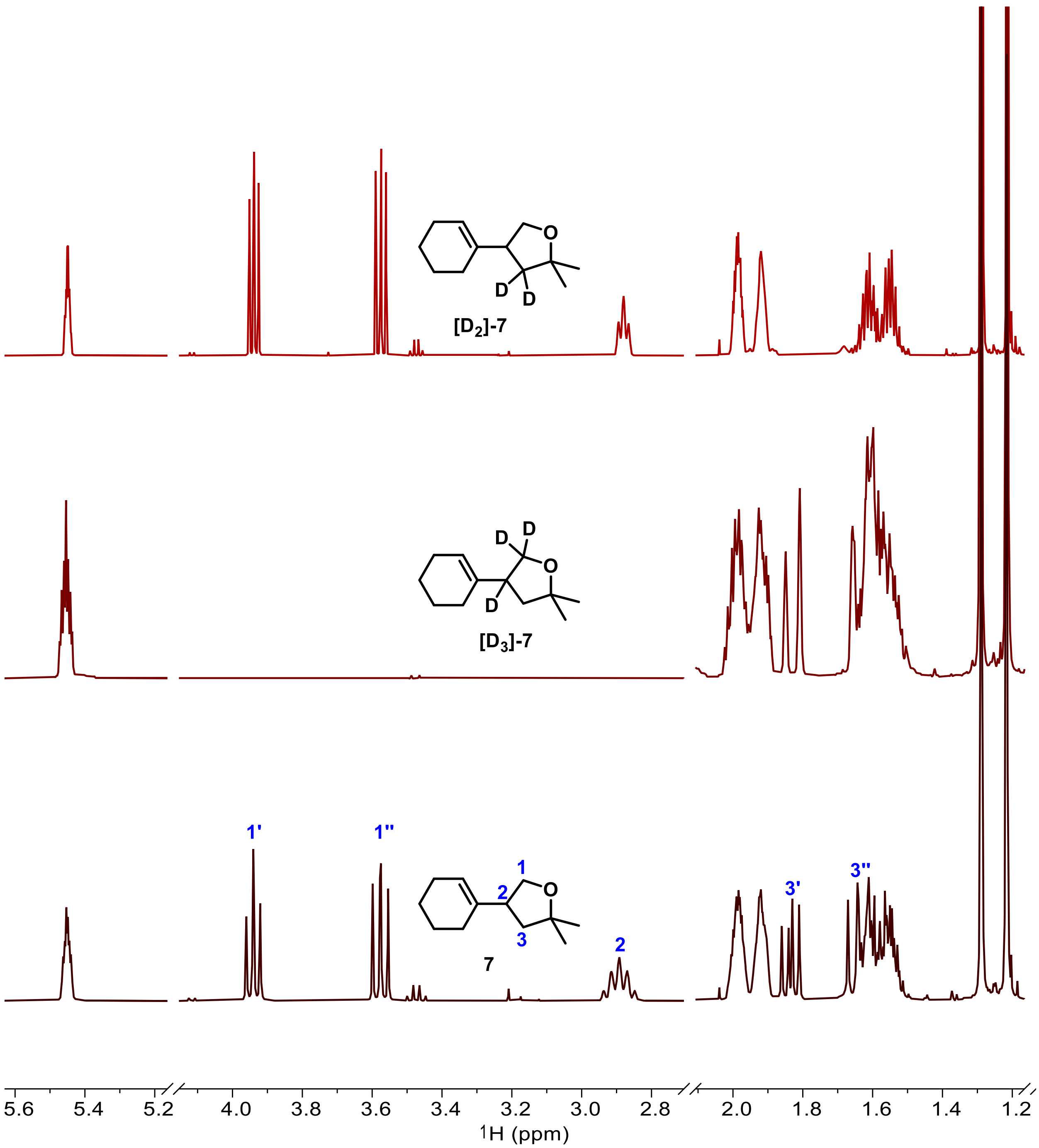

Figure S2. Stacked ^{1}H NMR spectra of selectively labeled and unlabeled tetrahydrofuran **7**.

Labeling experiments were conducted according to the general catalytic hydrogenation setup (see below) using [D_3]-**5** or [D_0]-**5** with either H_2 or D_2 gas. In both cases very high levels of deuteration/deuterium retention are observed as determined by NMR and MS, and the sites of deuterium incorporation are exclusive (no scrambling, cf. Scheme S1 and Figure S2).

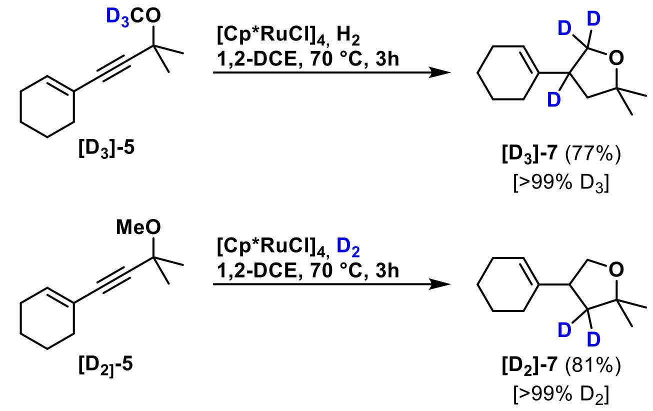

Scheme S1. Deuterium labeling experiments

Carbene Complex 6

***gem*-Hydrogenation: Preparation of the Carbene Complex 6.** $[Cp^*RuCl]_4$ (17.9 mg, 0.017 mmol) was added to a stirred solution of enyne **5** (9.8 mg, 0.055 mmol) in CD_2Cl_2 (1.0 mL) in a flame dried Schlenk tube at 0 °C under argon. H_2 was bubbled through the mixture for 2 min before the mixture was stirred for 15 min at 0 °C under an hydrogen atmosphere (balloon). The solution was cooled to –78 °C and subsequently subjected to NMR analysis at –80 °C. NMR spectra and signal assignments for the two interconverting species **η^3-6** and **η^1-6** are compiled in Table S1.

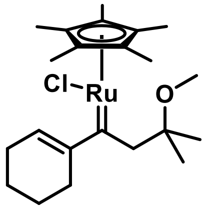

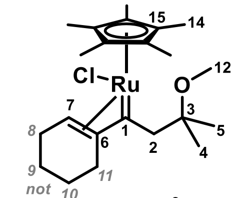

η^3-6

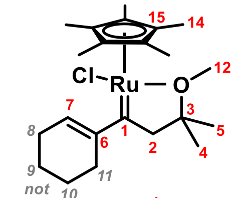

η^1-6

Table S1. NMR analysis of vinylcarbene species **η^3-6** (left) and **η^1-6** (right) (CD_2Cl_2, –80 °C, 400 MHz)

Atom [#] C	H	δ [ppm]	COSY	HSQC	HMBC
**1**		288.81			2', 2''
**2**		51.08		2', 2''	4, 5
	**2'**	3.48	2''	2	1, 3, 4, 5, 6
	**2''**	2.6	2'	2	1, 3, 4, 5, 6
**3**		74			2', 2'', 4, 5
**4**		24.87		4	2', 2'', 5
	**4**	1.07		4	2, 3, 5
**5**		24.64		5	2', 2'', 4
	**5**	0.67		5	2, 3, 4
**6**		90.68			2', 2''
**7**		72.25		7	
	**7**	4.94		7	
**12**		49.16		12	
	**12**	3.12		12	
**13**		97.07			14
**14**		9.65		14	
	**14**	1.50		14	13

Atom [#] C	H	δ [ppm]	COSY	HSQC	HMBC
**1**		308.34			2', 2''
**2**		64.7		2', 2''	4, 5
	**2'**	2.8	2''	2	1, 6
	**2''**	1.21	2'	2	1, 3, 4
**3**		81.8			2'', 4, 5, 12
**4**		21.84		4	2'', 5
	**4**	0.9		4	2, 3, 5
**5**		24.08		5	4
	**5**	1.31		5	2, 3, 4
**6**		159.04			2'
**7**		139.28		7	
	**7**	6.76		7	
**12**		53.62		12	
	**12**	3.21		12	3
**13**		88.91			14
**14**		9.46		14	
	**14**	1.31		14	13

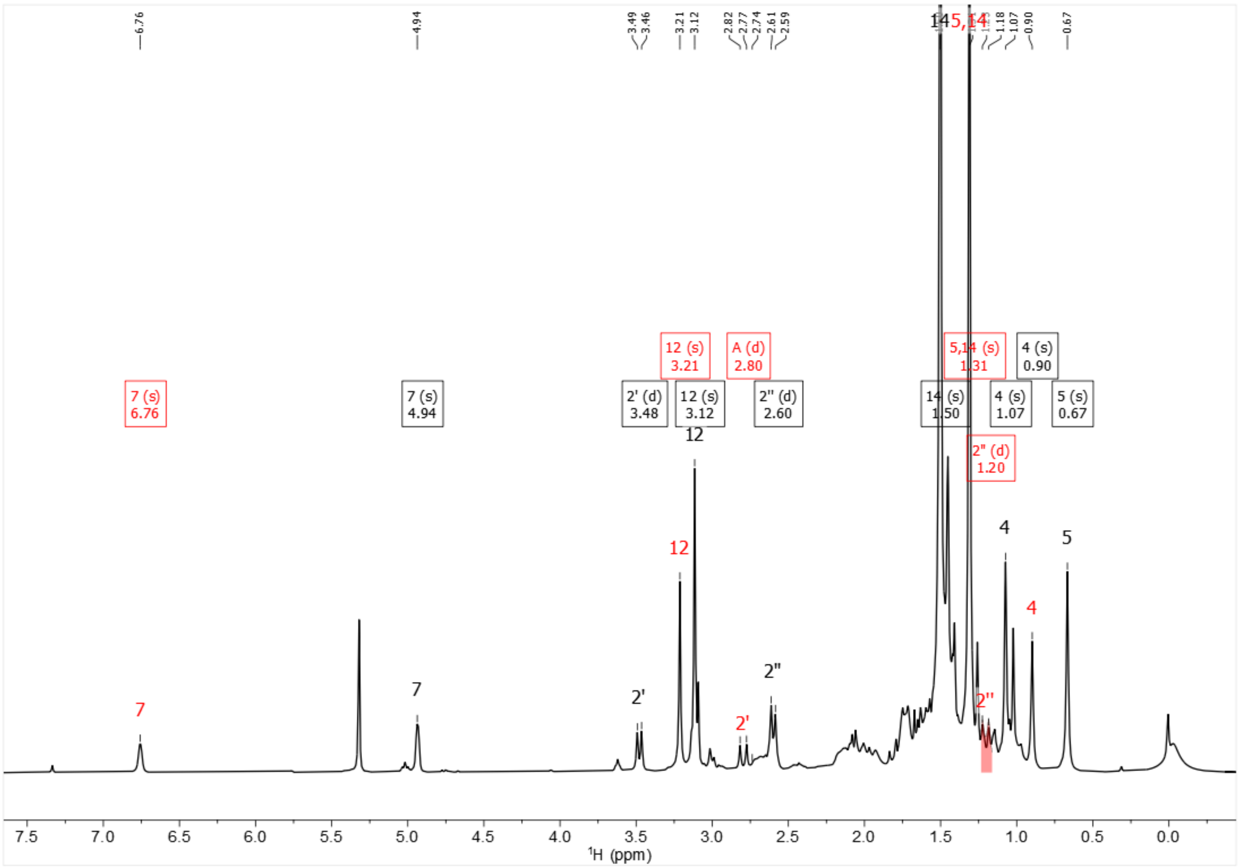

Figure S3. ^{1}H NMR spectrum recorded upon stoichiometric *gem*-hydrogenation of enyne **5** showing the two carbene complexes **η³-6** and **η¹-6** (assignments in black (**η³-6**) and red (**η¹-6**)); peak-picking shown only for signals attributed to the carbene complexes

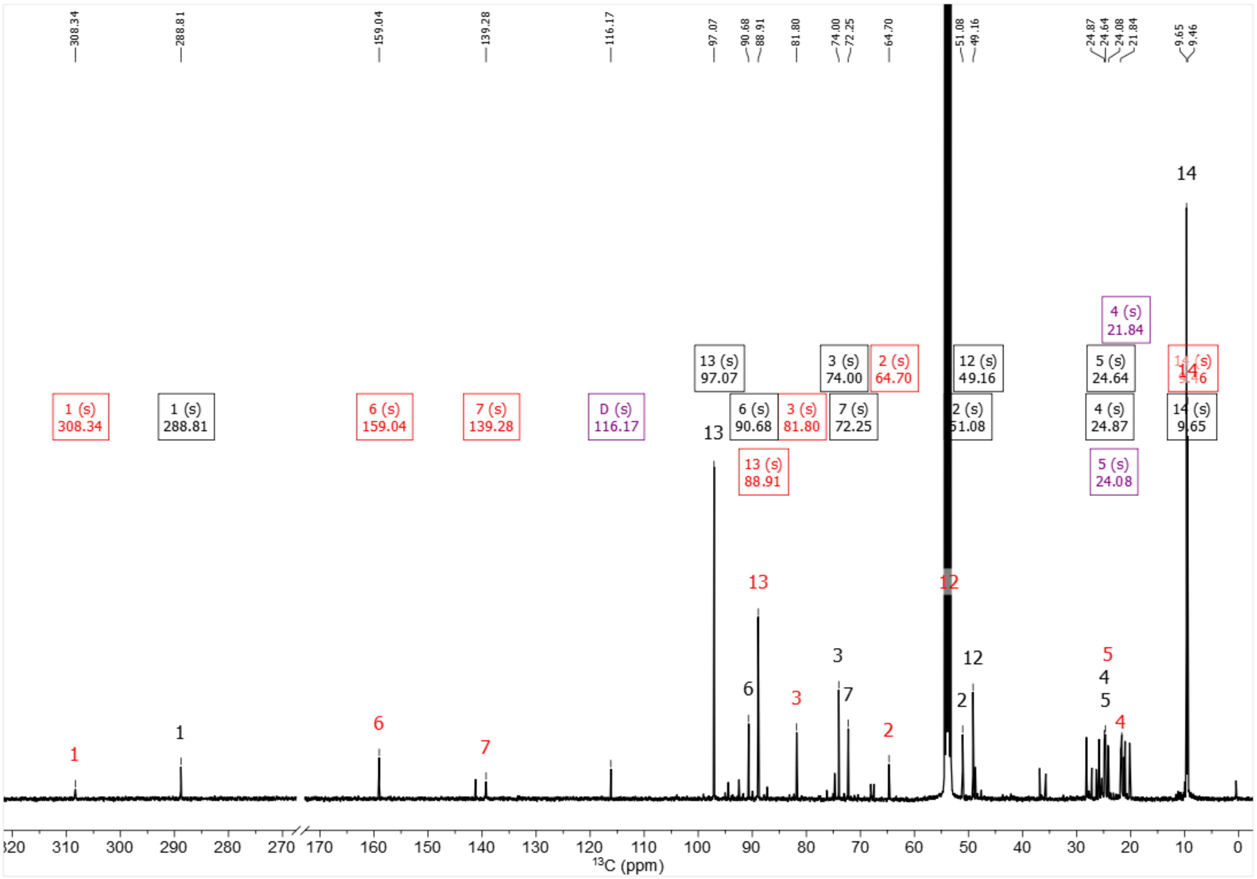

Figure S4. ^{13}C NMR spectrum showing the two carbene complexes **η^3-6** and **η^1-6** (assignments in black (**η^3-6**) and red (**η^1-6**)); peak-picking shown only for signals attributed to the carbene complexes.

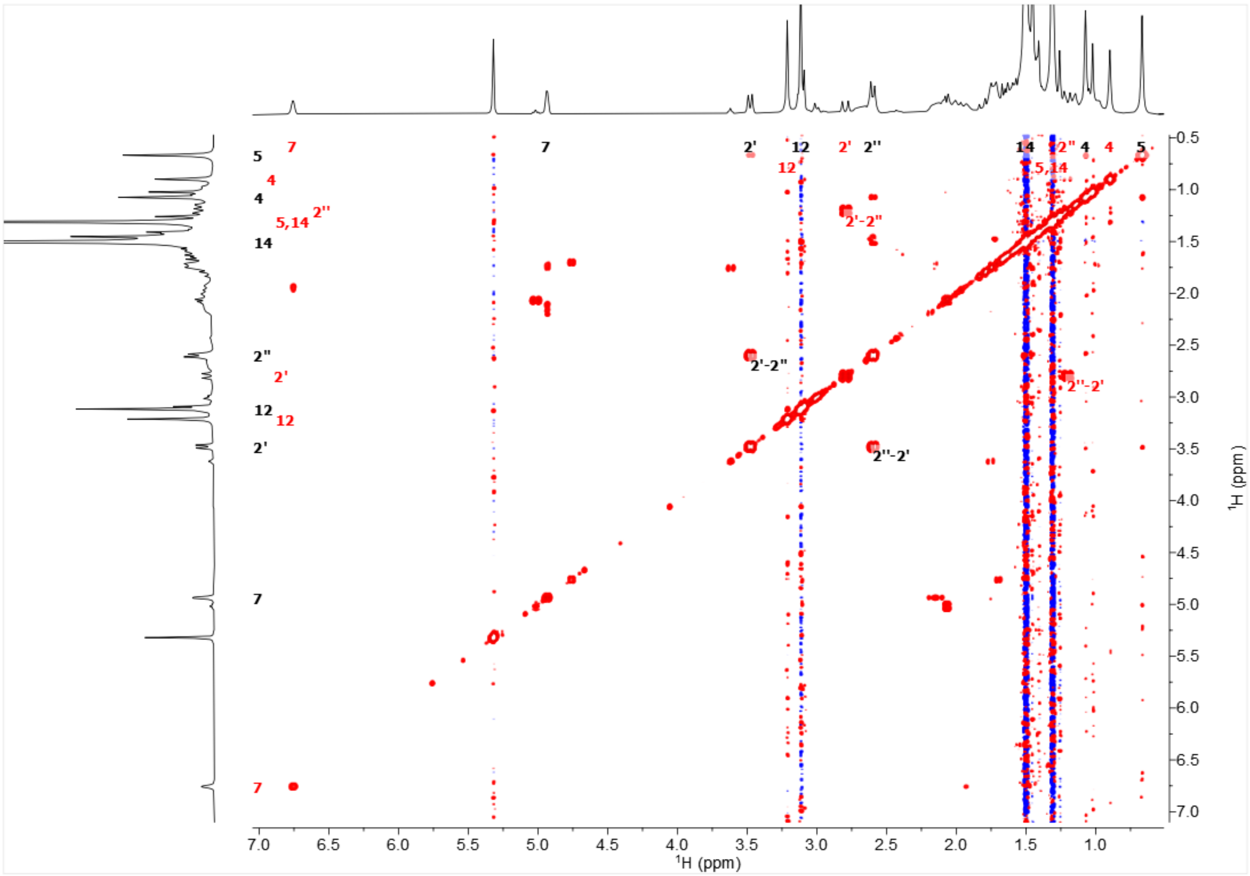

Figure S5. COSY spectrum showing the two carbene complexes **η³-6** and **η¹-6** (assignments in black (**η³-6**) and red (**η¹-6**)); peak-picking shown only for signals attributed to the carbene complexes

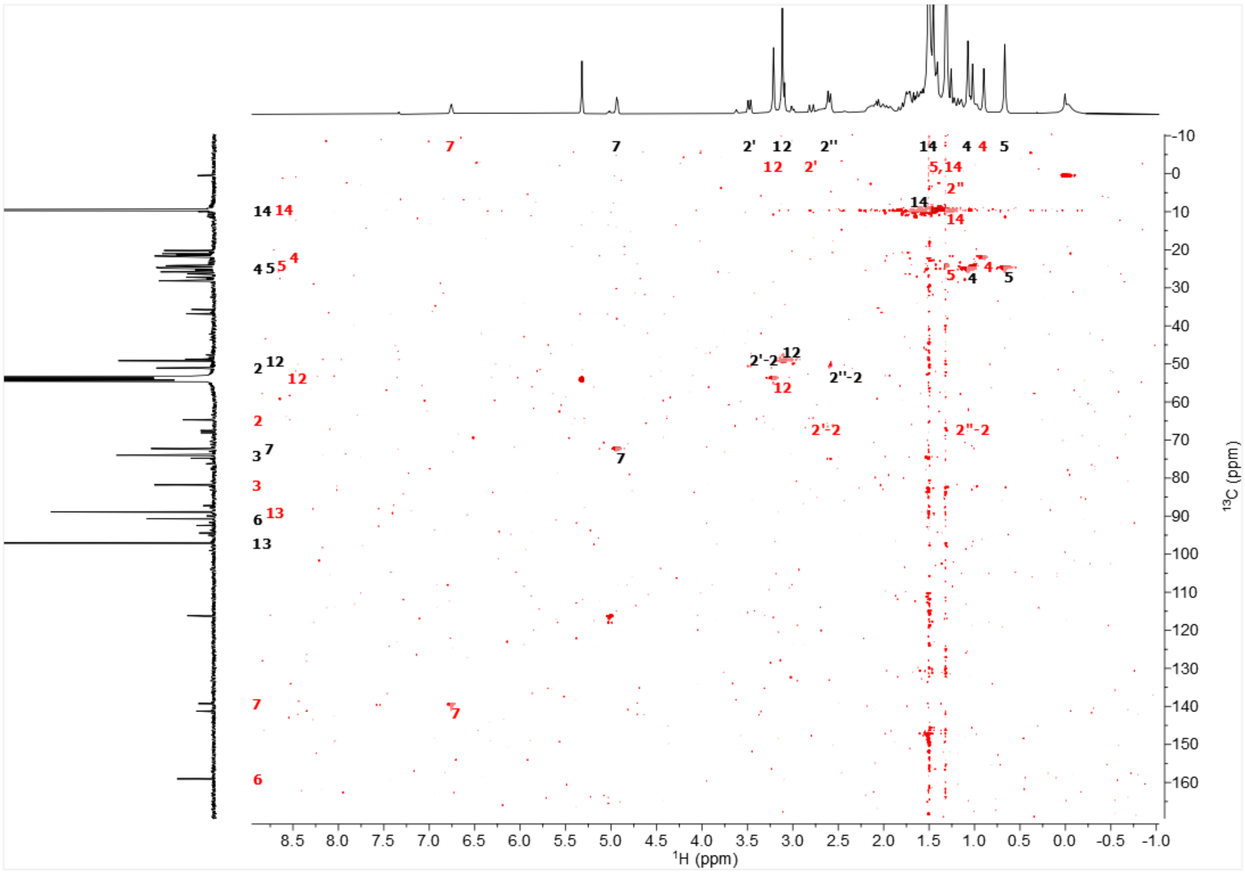

Figure S6. HSQC spectrum showing the two carbene complexes **η³-6** and **η¹-6** (assignments in black (**η³-6**) and red (**η¹-6**)); peak-picking shown only for signals attributed to the carbene complexes

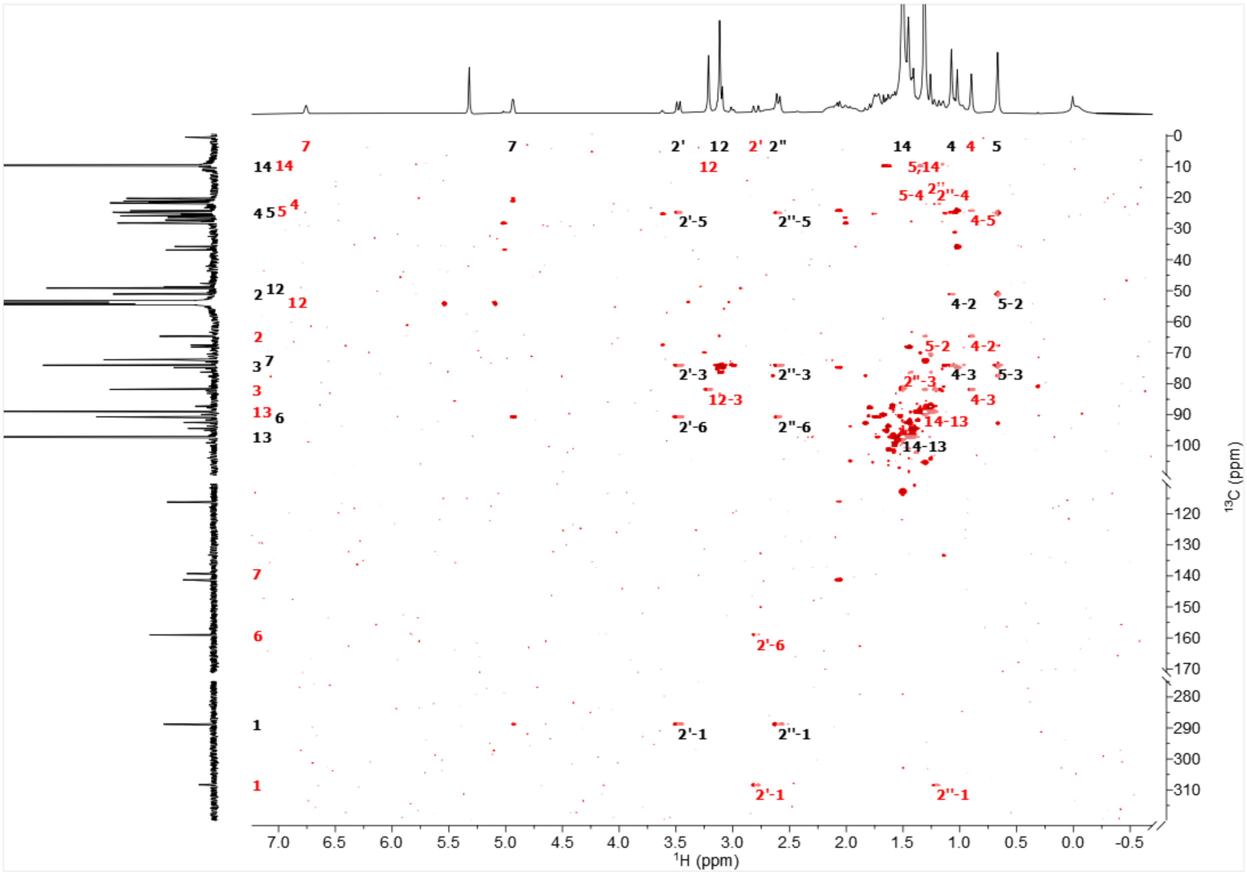

Figure S7. HMBC spectrum showing the two carbene complexes **η^3-6** and **η^1-6** (assignments in black (**η^3-6**) and red (**η^1-6**)); peak-picking shown only for signals attributed to the carbene complexes

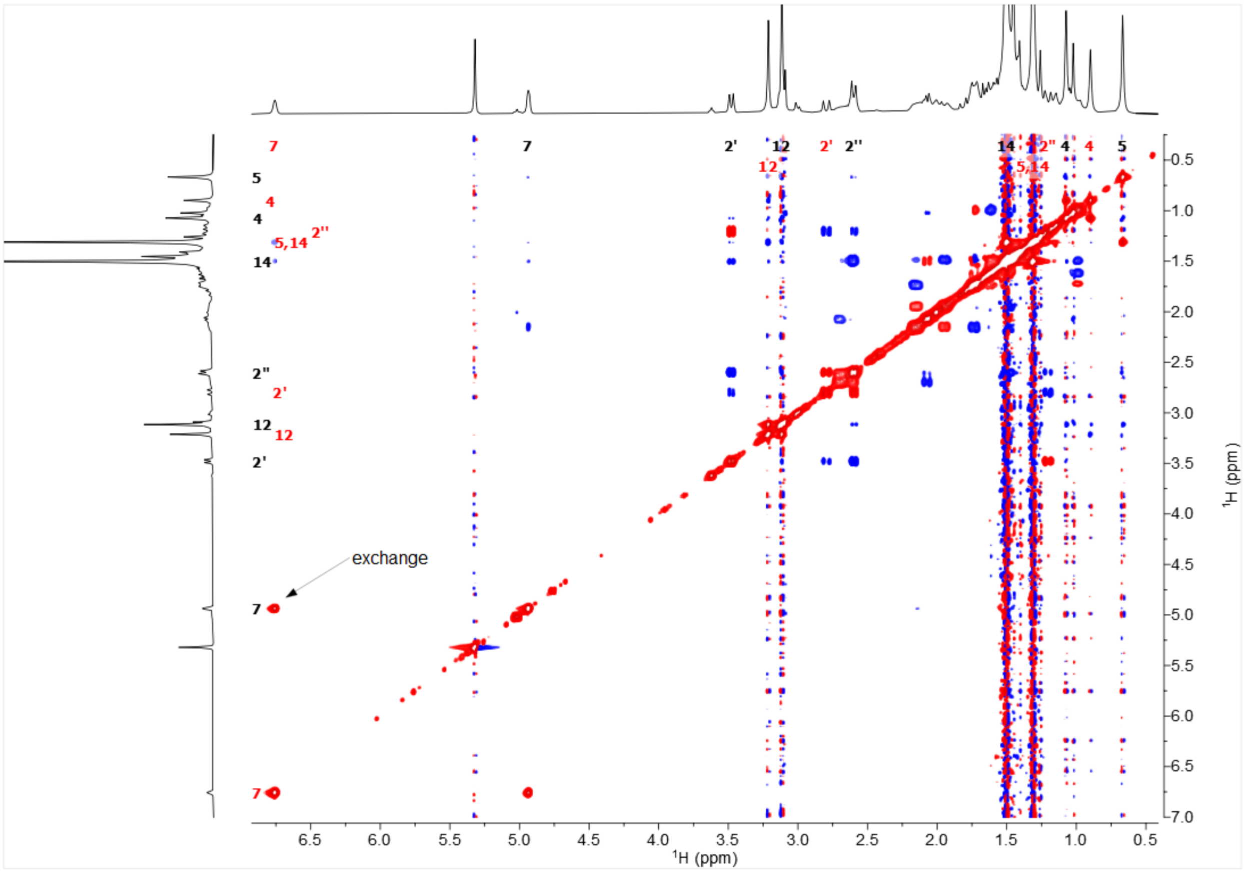

Figure S8. ROESY spectrum showing the interconversion of the two carbene complexes **η³-6** and **η¹-6** (assignments in black (**η³-6**) and red (**η¹-6**)); peak-picking shown only for signals attributed to the carbene complexes

Variable Temperature NMR

The reaction mixture obtained from *gem*-hydrogenation of **5** was analyzed at temperatures ranging from –80 °C to +35 °C (CD_2Cl_2, 600 MHz). The resulting complexes rapidly interconvert at room temperature, thus giving rise to one set of averaged signals. At lower temperatures, two distinct complexes can be observed, with **η³-6** being slightly favored (**η³-6** : **η¹-6** ≈ 3 : 2). The interconversion likely proceeds by dissociative ligand exchange via the 16e complex).

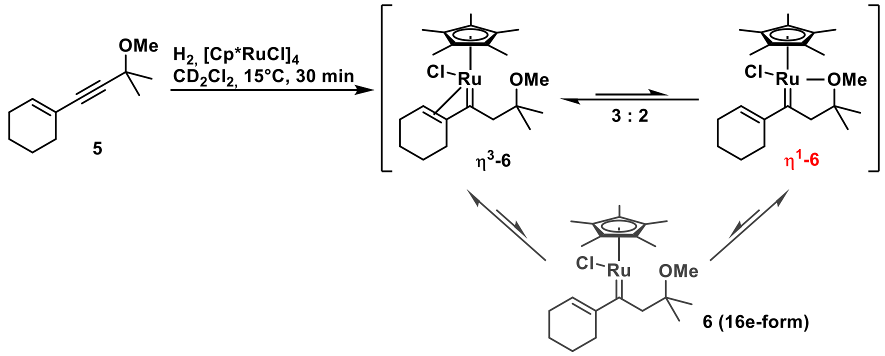

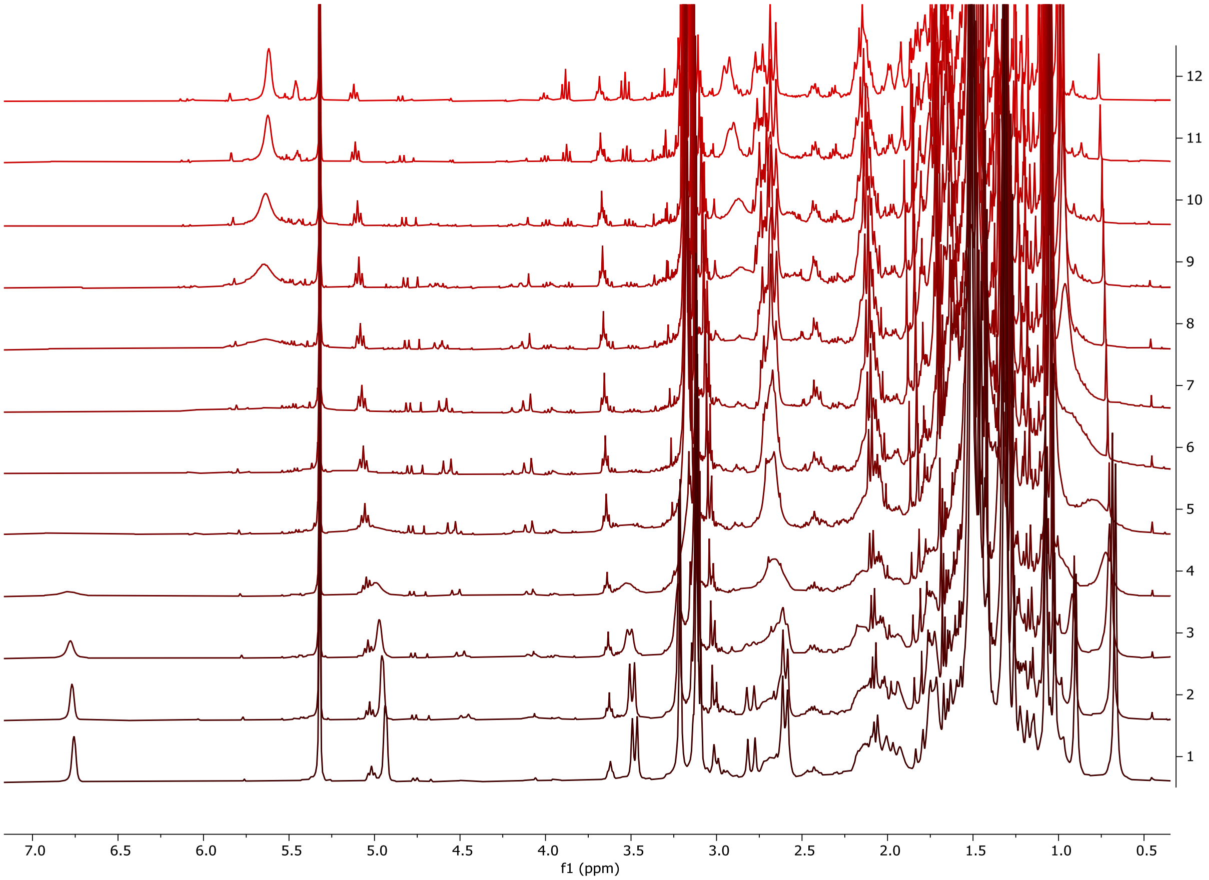

Figure S9. Complete Variable Temperature ¹H NMR spectra starting at –80 °C (trace 1) to +10 °C (trace 10) in 10 °C intervals (trace 11 = 25 °C; trace 12 = 35 °C).

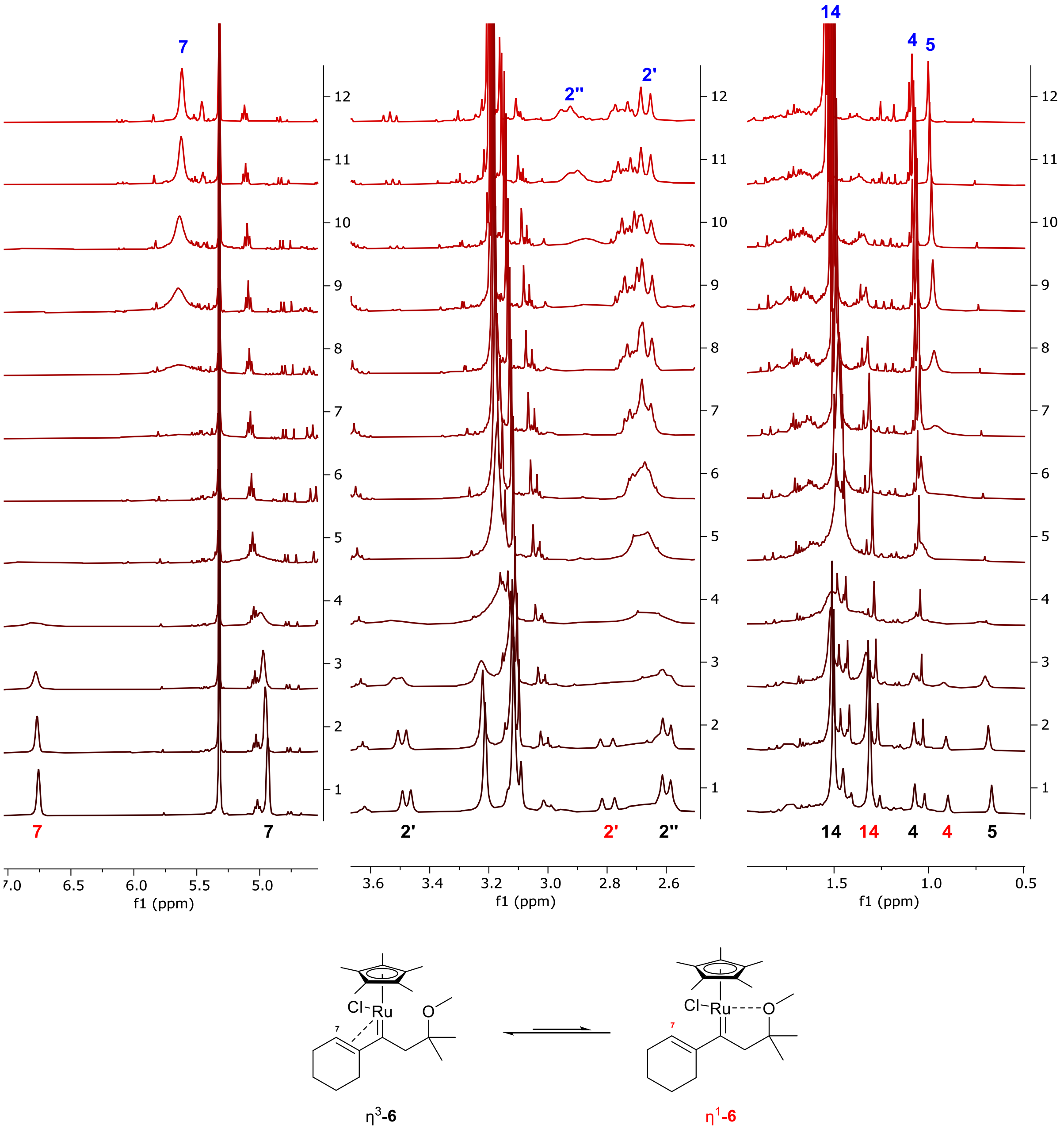

Figure S10. Excerpts from the VT-NMR ^{1}H NMR spectrum showing the coalescense of characteristic proton signals corresponding to the slow (–80 °C) and fast (+25 °C) interconversion of species **η^{3}-6** and **η^{1}-6** (color-coding and temperatures as above)

The exchange rates between η3-**6** and η1-**6** was analysed by NMR lineshape simulations at different temperatures. After Eyring analysis the barrier for interconversion $\mathbf{\Delta G^{\ddagger}_{298K}}$**= 10.4 ± 0.31 kcal/mol** was determined.

^{1}H NMR signals of the exchanging olefinic protons H-7 of η3-**6** and η1-**6** in were simulated and fitted in the DNMR module of Bruker Topspin 4.09 at 10 different temperatures (193K, 203K, 213K, 223K, 233K, 243K, 253K, 263K, 273K, 283K) to obtain the exchange rate *k* between the two complexes at different temperatures. A two site exchange model was used for the lineshape fitting (H-7->H-7, H-7->H-7). The coupling to the neighbors was simulated with an additional signal, which was not used in the analysis. At temperatures above 223 K where no individual chemical shift in the complexes could be extracted, the shift values were extrapolated from the VT behavior at lower temperatures. The analysis results are shown in Table S2.

Table S2. Overview over all the parameters used or obtained from lineshape fitting by DNMR. Values marked with an asterix (*) were obtained from fitting. All other values were fixed

	η1-**6** H-7			η3-**6** H-7					
**T (K)**	**δ (ppm)**	**X_1**	**$J_{H7,H8}$ (Hz)**	**δ (ppm)**	**X_2**	**$J_{H7,H8}$ (Hz)**	**Intensity (a.u.)**	**lb (Hz)**	***r* (s^{-1})**
193	6.7984*	0.3871*	4	4.9773*	0.6129*	4.5	17688754	5.0	5.04*
203	6.8102*	0.3993*	4	4.9961*	0.6007*	4.5	14669896	1.5	28.78*
213	6.8205*	0.3902*	4	5.0135*	0.6098*	4.5	13545861	1.0	81.34*
223	6.8311*	0.3675*	4	5.0311*	0.6325*	4.5	12002291	1.0	270.40*
233	6.8422	0.3540	4	5.0492	0.6460	4.5	16863576	1.0	785.25*
243	6.8530	0.3350	4	5.0671	0.6650	4.5	17541863	1.0	2150.00
253	6.8638	0.3440	4	5.0850	0.6560	4.5	17996347	1.0	4887.00
263	6.8747	0.3311*	4	5.1028	0.6689*	4.5	22089171	1.0	12942.60*
273	6.8855	0.3200*	4	5.1207	0.6800*	4.5	17372325	1.0	26938.40*
283	6.8964	0.3074*	4	5.1386	0.6927*	4.5	14454554	1.0	44620.10*
298	6.9126	0.2846*	4	5.1654	0.7155*	4.5	12009300	1.0	75311.90*

DNMR Lineshape Analysis

The following spectra show the result from the DNMR lineshape simulation at different temperatures; color code: **red**: simulated spectrum obtained from DNMR after fitting; **black**: experimental spectrum; **blue**: the difference between the experimental and the simulated spectrum.

193K

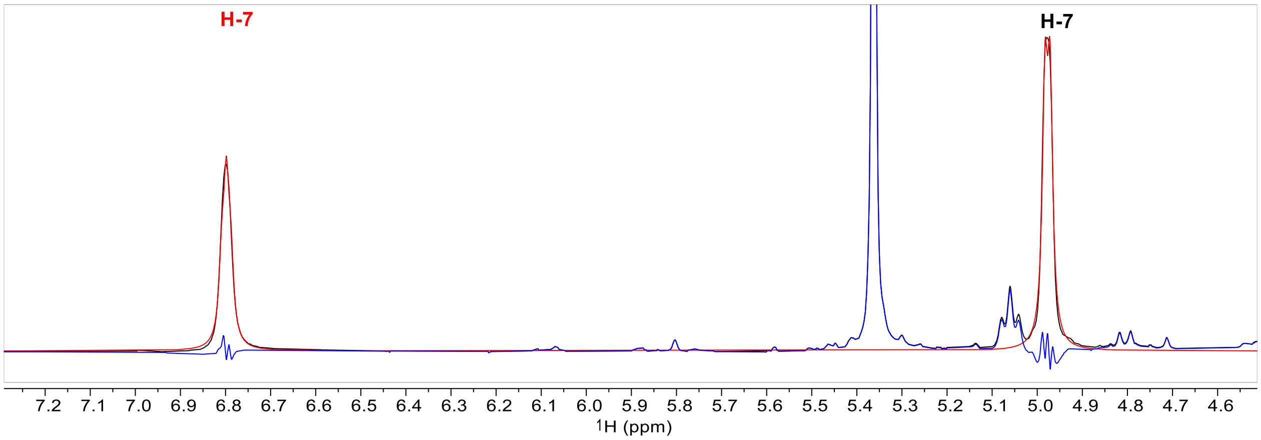

203K

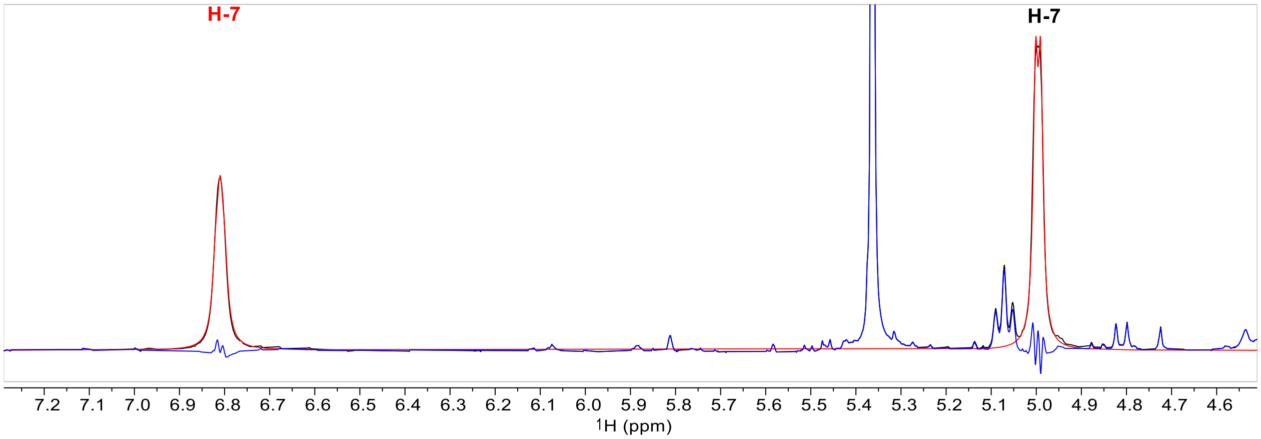

213K

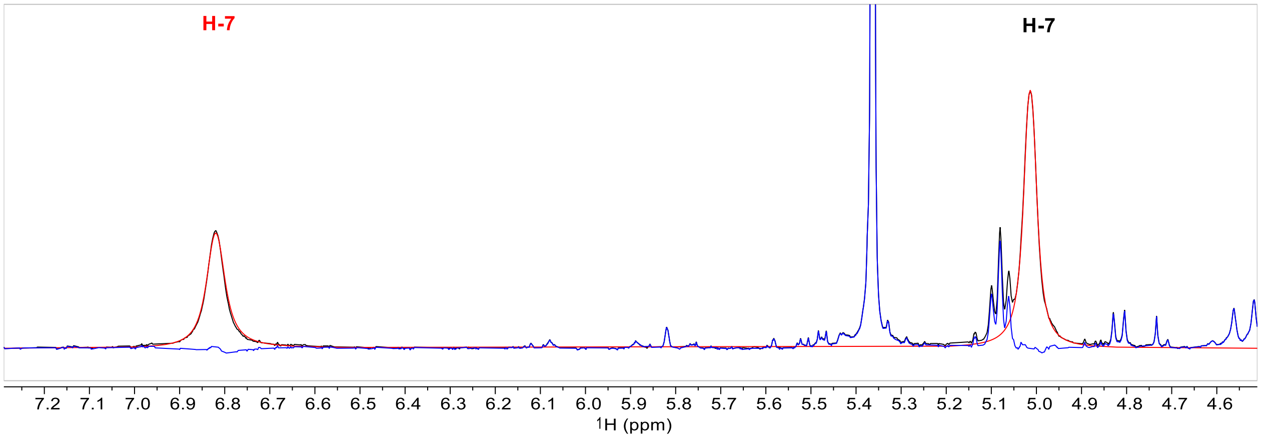

223K

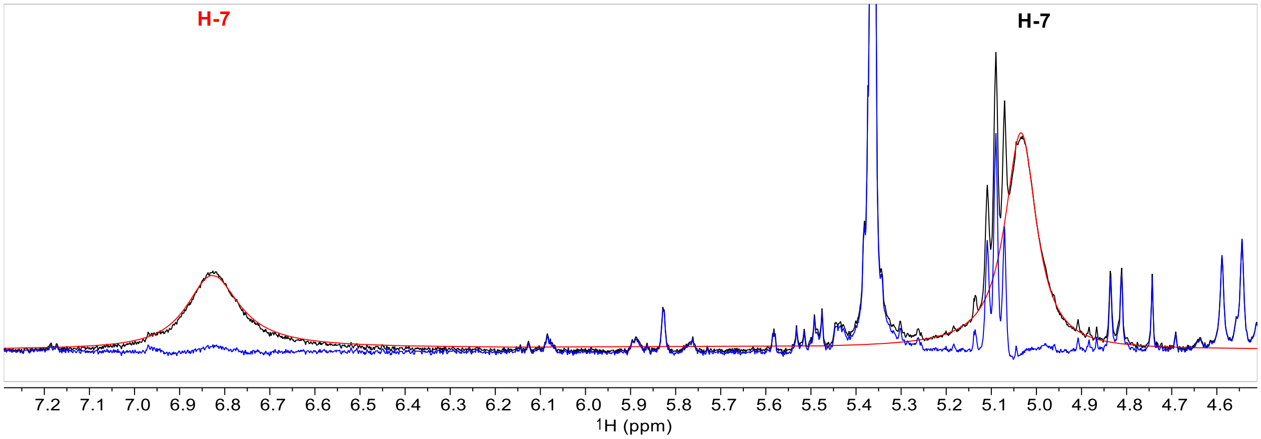

233K

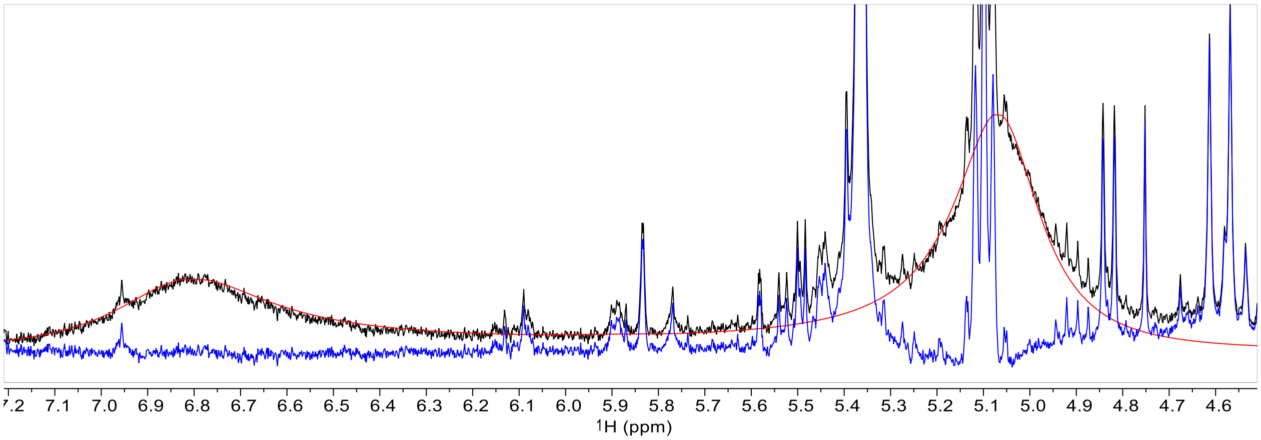

243K

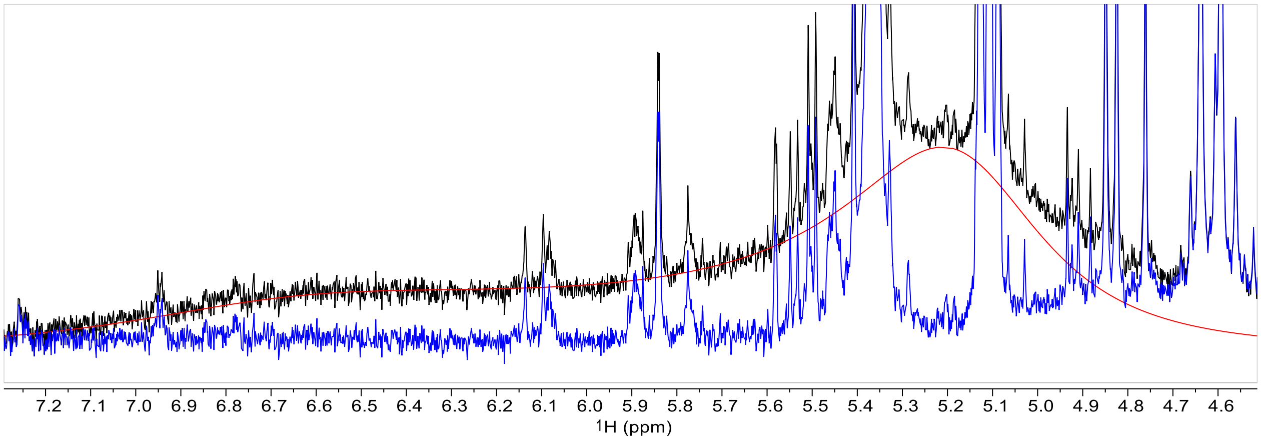

253K

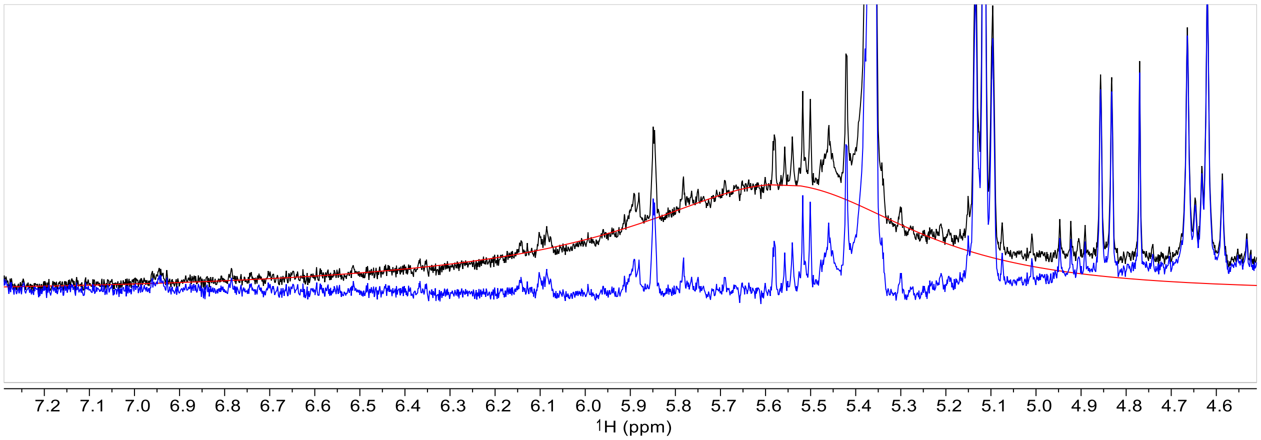

263K

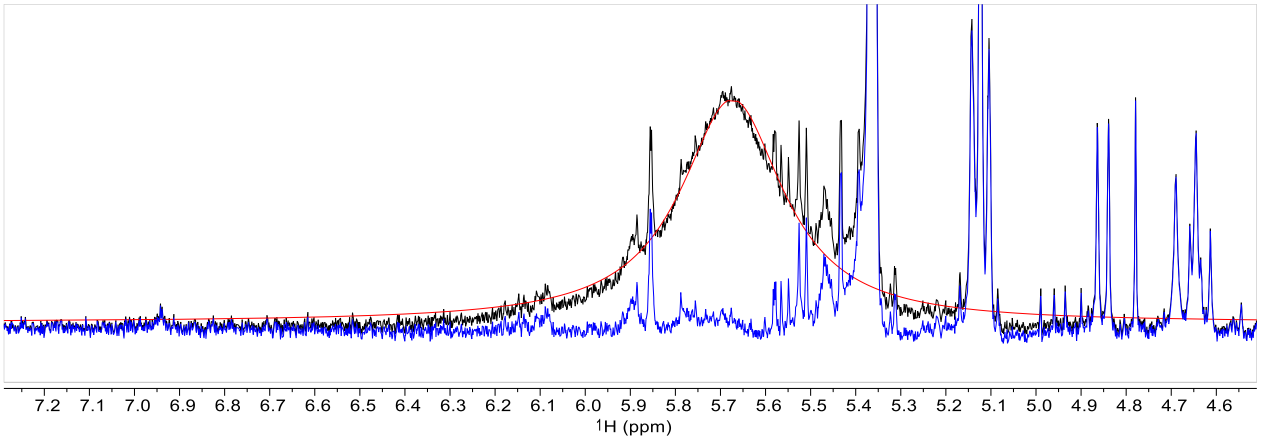

273K

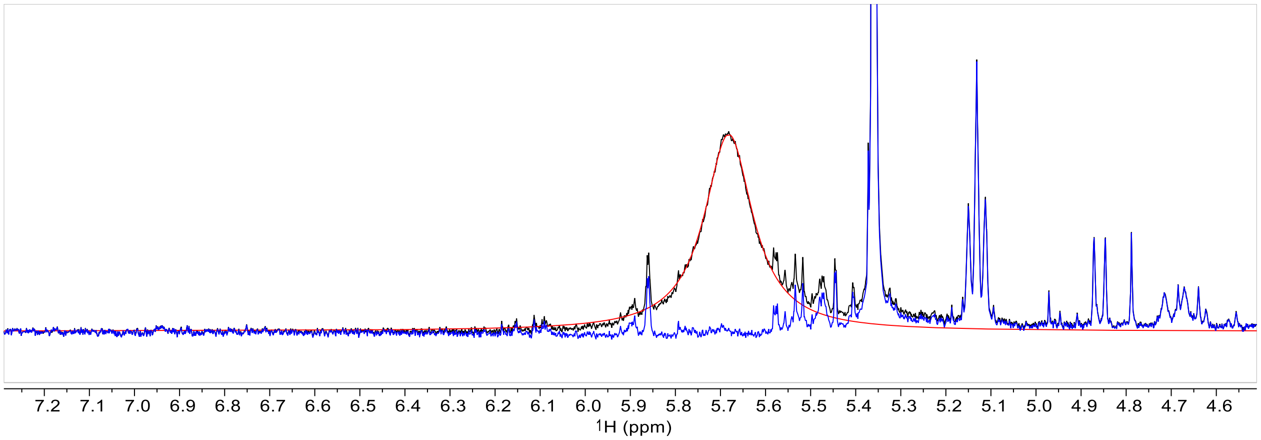

283K

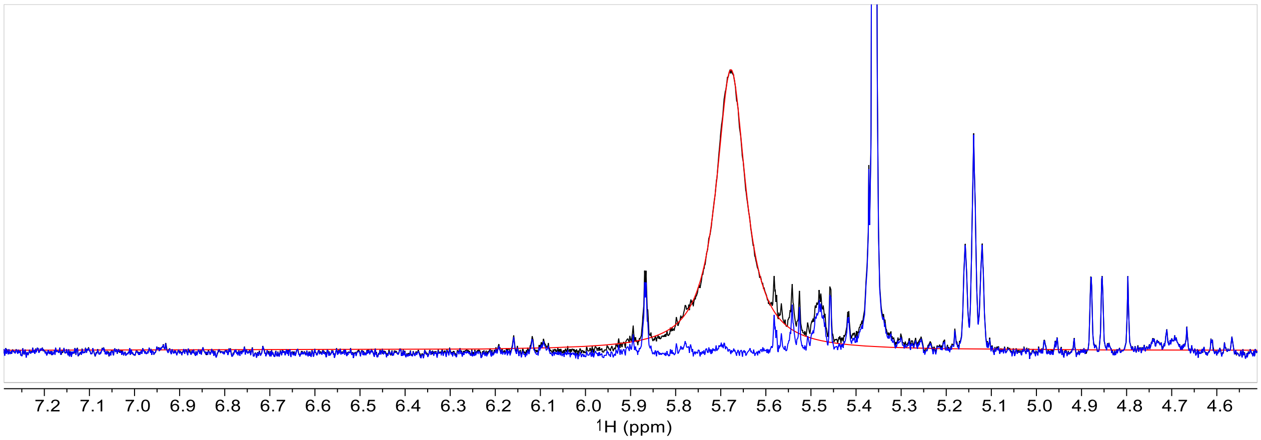

Eyring Analysis

The following physical contants were used for the Eyring analysis:

	Value	Unit
**R**	8.31446	J/(K*mol)
**h**	6.63E-34	J s
**k_B**	1.38E-23	J/K

Table S3. Parameters used for the Eyring analysis

T(K)	1/T	1/(RT)	k (s-1)	(k*h/(kb*T))	ln ((k*h/(kb*T)))
193.0	0.005181	6.232E-04	5.04	1.2542E-12	-27.4045
203.0	0.004926	5.925E-04	28.78	6.8033E-12	-25.7136
213.0	0.004695	5.647E-04	81.34	1.8327E-11	-24.7226
223.0	0.004484	5.393E-04	270.40	5.8194E-11	-23.5672
233.0	0.004292	5.162E-04	785.25	1.6174E-10	-22.545
243.0	0.004115	4.949E-04	2150.00	4.2462E-10	-21.5798
253.0	0.003953	4.754E-04	4887.00	9.2703E-10	-20.799
263.0	0.003802	4.573E-04	12942.60	2.3618E-09	-19.8639
273.0	0.003663	4.406E-04	26938.40	4.7357E-09	-19.1681
283.0	0.003534	4.250E-04	44620.10	7.5669E-09	-18.6995
298.0	0.003356	4.036E-04	75311.90	1.2129E-08	-18.2277

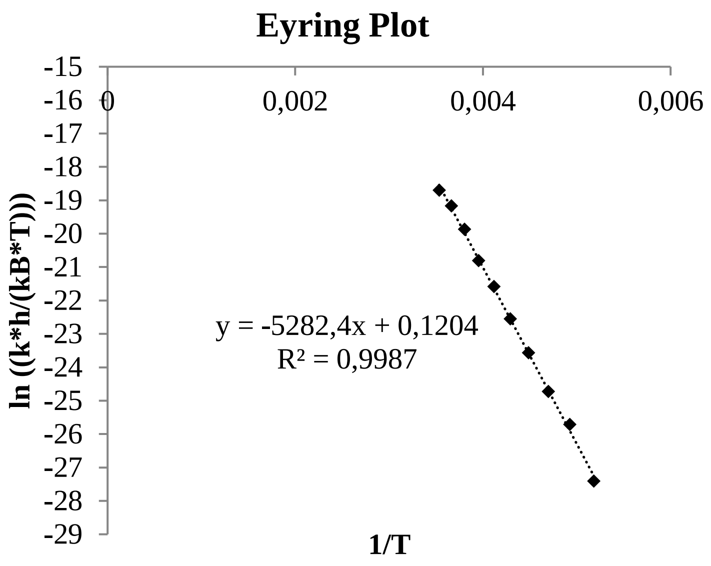

Figure S11. Eyring plot based on ^{1}H lineshape analysis

Table S4. Values obtained from linear regression after plotting ln ((k*h/(kb*T))) against 1/T.

	**-ΔH/R**	**ΔS/R**
value	-5282.3648	0.1204
St.Dev	68.1418	0.2928
R^2	0.9987	0.1128
F	6009.3838	8.0000
ssreg	76.5075	0.1019

Table S5. Thermodynamic parameters obtained by the Eyring analysis.

ΔH	43.9	± 0.6	kJ/mol
**ΔH**	**10.5**	**± 0.1**	**kcal / mol**
ΔS	1.0	± 2.4	J/mol/K
**ΔS**	**0.24**	**± 0.6**	**cal/mol/K**
ΔG (25°C)	43.6	± 1.3	kJ/mol
**ΔG (25°C)**	**10.4**	**± 0.31**	**kcal / mol**

PHIP NMR

Formation of Carbene 6. [Cp*RuCl]$_4$ (1.9 mg, 2 mol%) was added to a stirred solution of enyne **5** (15.7 mg, 0.09 mmol) in CD_2Cl_2 (1 mL) in a flame dried Schlenk tube under argon. The mixture was transferred into a pressure NMR tube (5 mm medium wall precision pressure/vacuum valve NMR sample tube, *Wilmad-LabGlass*), which was tightly closed. The tube was connected to the *p*-H_2 generator and all tubings were evacuated and backfilled with *para*-hydrogen (*p*-H_2) three times. Then, the pressure was increased to 5 bar and the valve was opened to fill the tube with *para*-hydrogen to a total pressure of ≈ 6 bar. After closing the valve, the tube was shaken and immediately inserted into the NMR magnet.[4] The hyperpolarized signals (Figure S12) confirm the pairwise addition of both hydrogens from the same H_2 molecule.

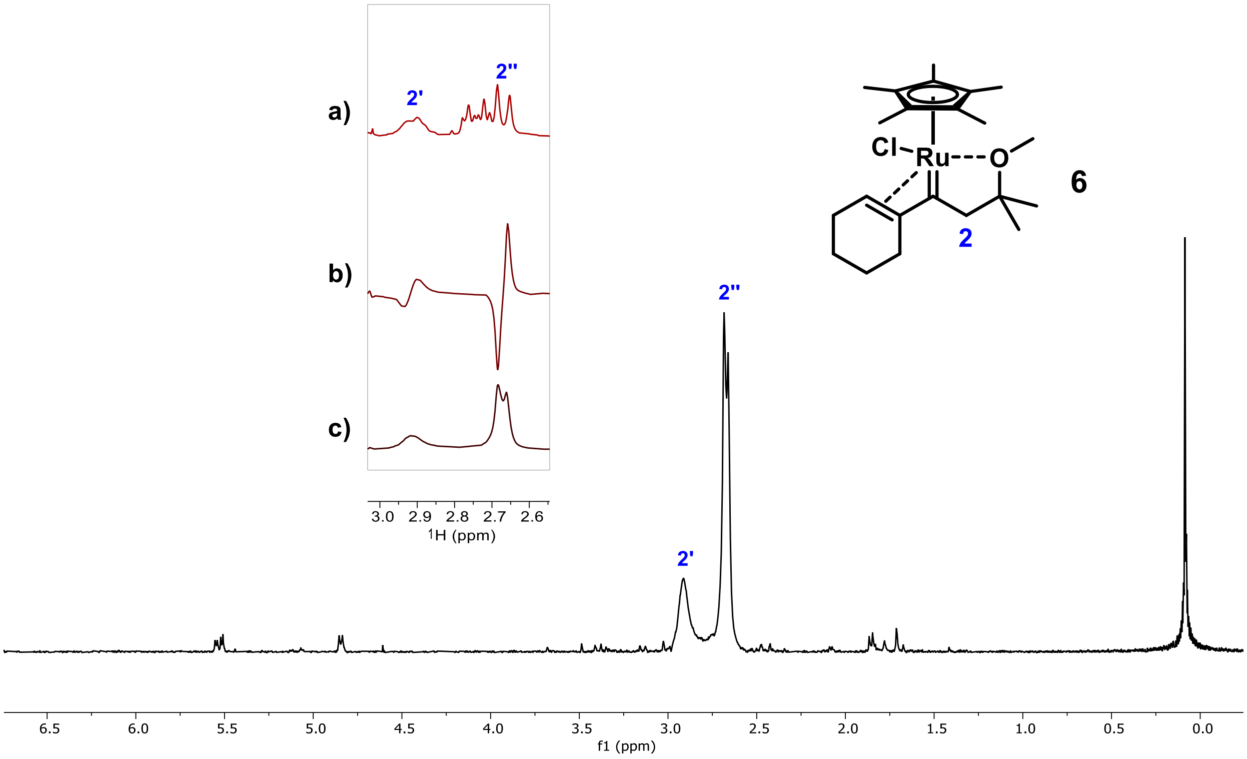

Figure S12. OPSY spectrum recorded during the generation of carbene complex **6** in the presence of *p*-H_2. Insert: stacked excerpts of a) ^{1}H NMR spectrum from the stoichiometric generation of **6** at room temperature, b) hyperpolarized antiphase signals of the methylene protons of the reactive intermeditae formed under catalytic conditions, c) excerpt from the OPSY spectrum for comparison

C–H Insertion: Kinetic Analysis

The decay of carbene complex **6** by C–H insertion was monitored by ^{1}H NMR at different temperatures (15 °C, 25 °C, 40 °C, 50 °C). Complex **6** was prepared immediately before use as described above. The reaction was followed by integration of the indicated methyl signals (*4* and *5*, Scheme S2). The reaction progress was analyzed using the MestRe Nova Reaction Monitoring tool. Integration/time data was imported into Excel, linearized and plotted. Rate constants were obtained by linear fitting. A representative carbene decay graph is shown in Figure S14 showing a first order reaction of complex **6** to product **7**. Linearized plots at all temperatures are given below.

Importantly, these experiments study the stoichiometric C–H insertion reaction, not *the overall catalytic process.*

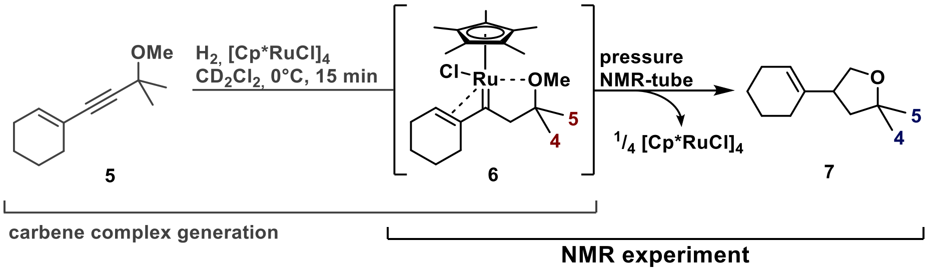

Scheme S2. Experimental setup

In parallel to formation of product **7**, tetrameric $[Cp^*RuCl]_4$ directly reassembles as evident from NMR. This observation means that one full turnover of the catalyst has been reached (no slow catalyst regeneration steps downstream the C–H insertion). The fact that formation of carbene complex **6** from **5** and H_2 is complete within 15 min at 0 °C but C–H insertion is slow even at room temperature ($t_{1/2}$ ~ 2 h) implies C–H insertion to be turnover limiting in the catalytic process.

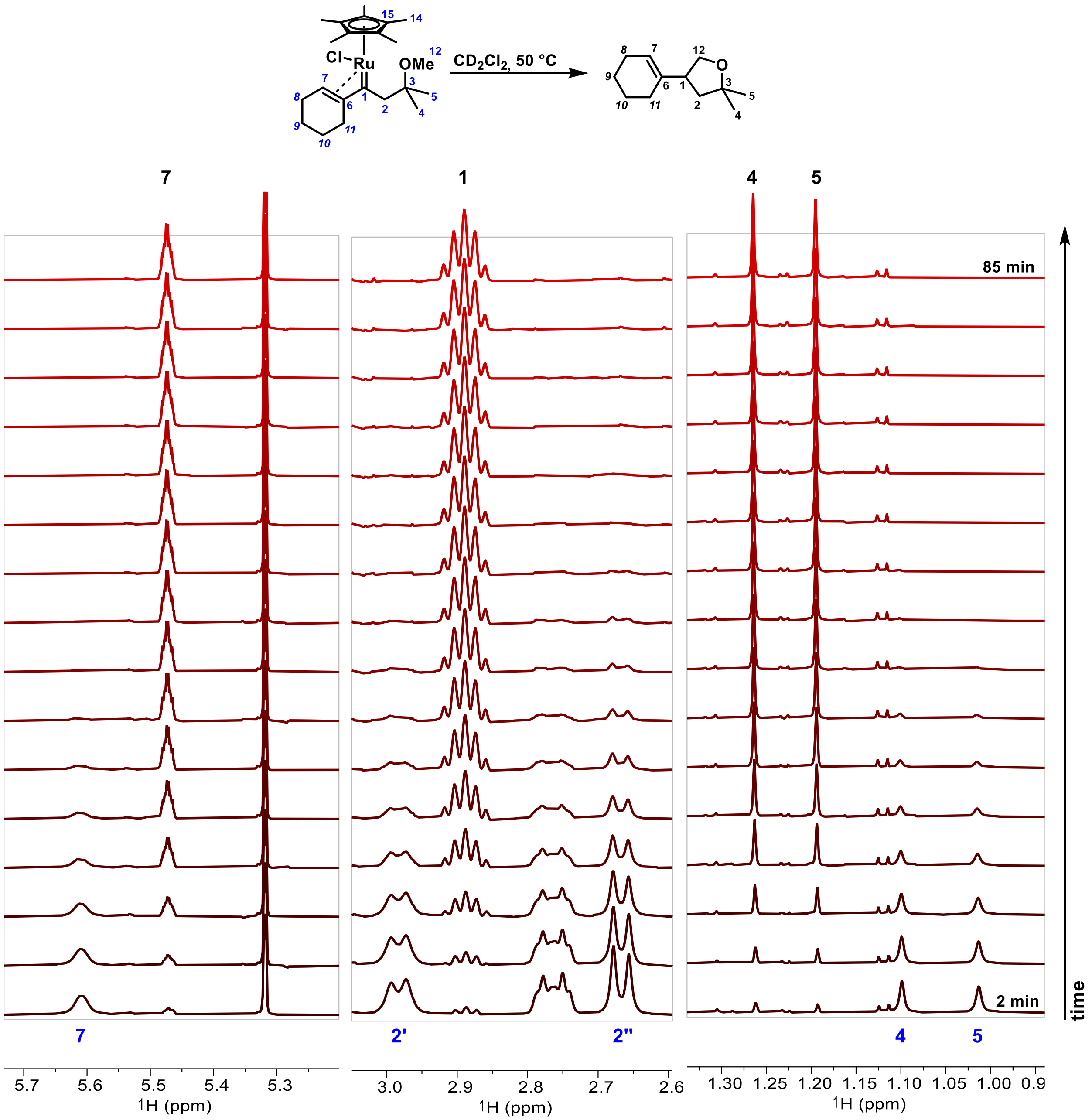

Figure S13. Excerpts of a stack of representative ^{1}H NMR spectra showing the decay of the ruthenium carbene **6** with formation of **7** at 50 °C over time (color-coded NMR signals as shown above).

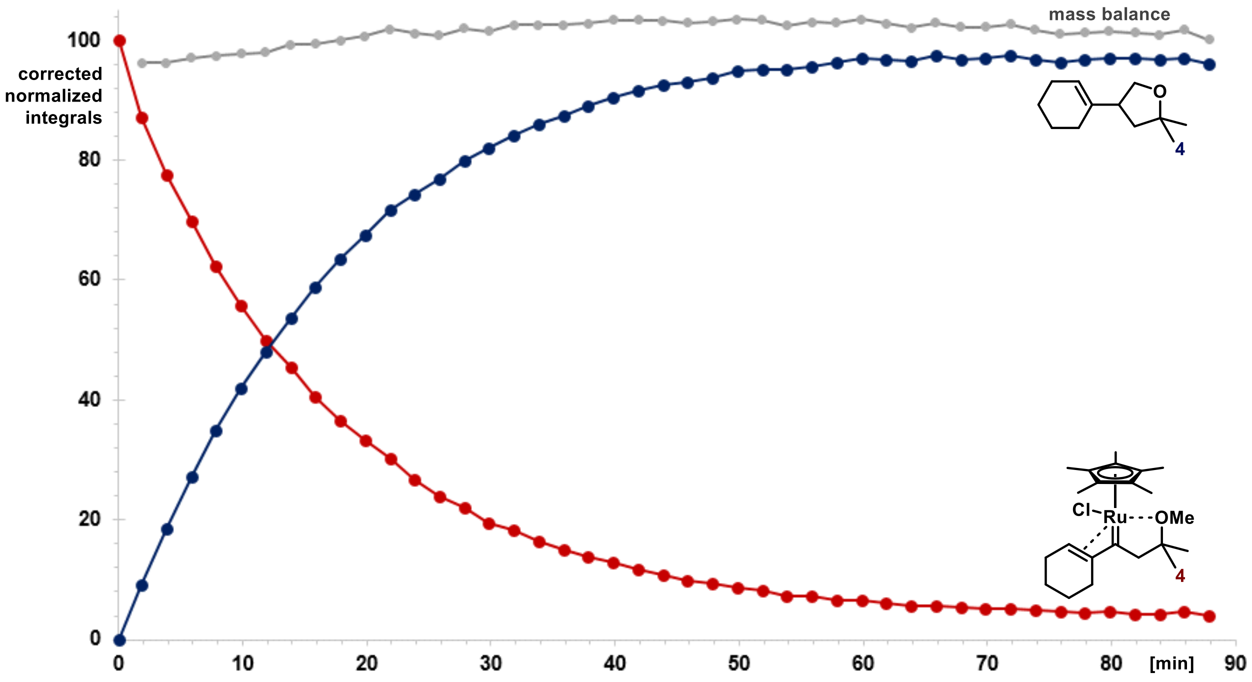

Figure S14. Plot of the kinetic data recorded for the C–H insertion reaction at 50 °C.

Reaction at 15 °C ($k_{(4)}$ = 2.67 x 10^{-5} s^{-1}, $k_{(5)}$ = 2.68 x 10^{-5} s^{-1}, k_{avg} = 2.68 x 10^{-5} s^{-1}, stdev: 1.82 x 10^{-8})

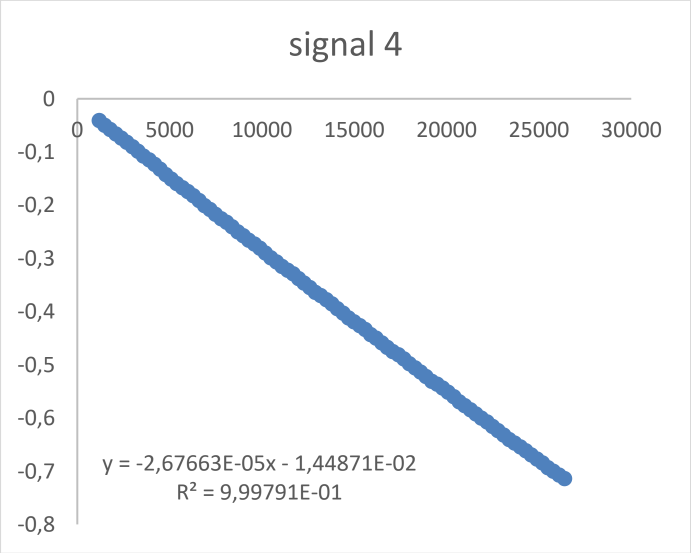

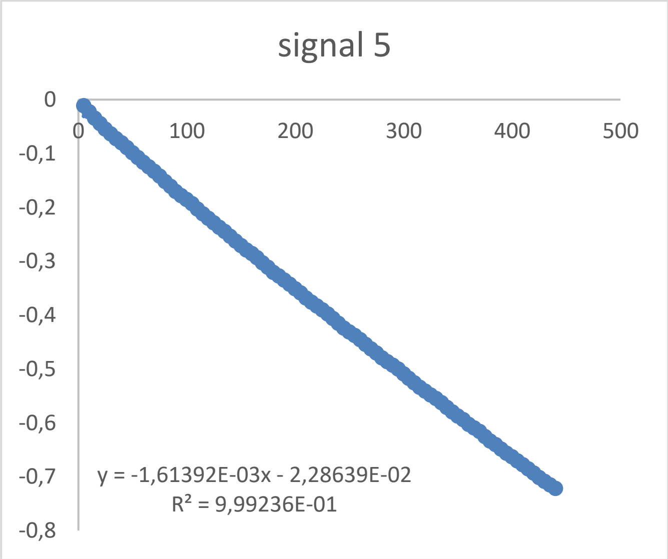

Reaction at 25 °C ($k_{(4)}$ = 7.73 x 10^{-5} s^{-1}, $k_{(5)}$ = 8.09 x 10^{-5} s^{-1}, k_{avg} = 7.91 x 10^{-5} s^{-1}, stdev: 2.52 x 10^{-6})

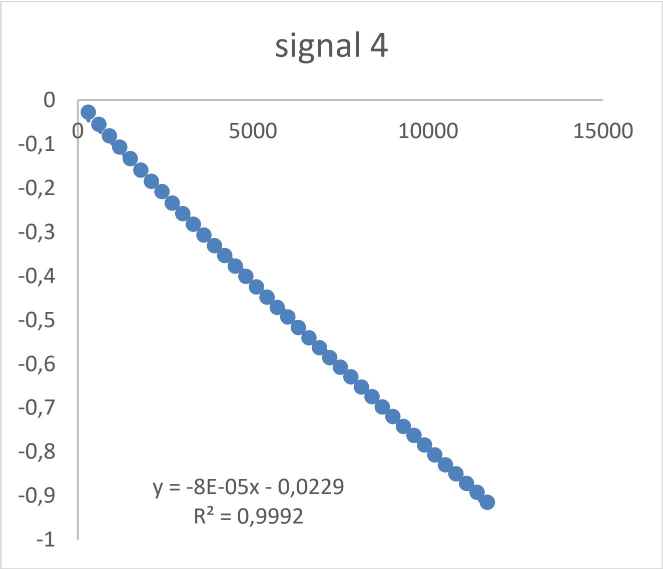

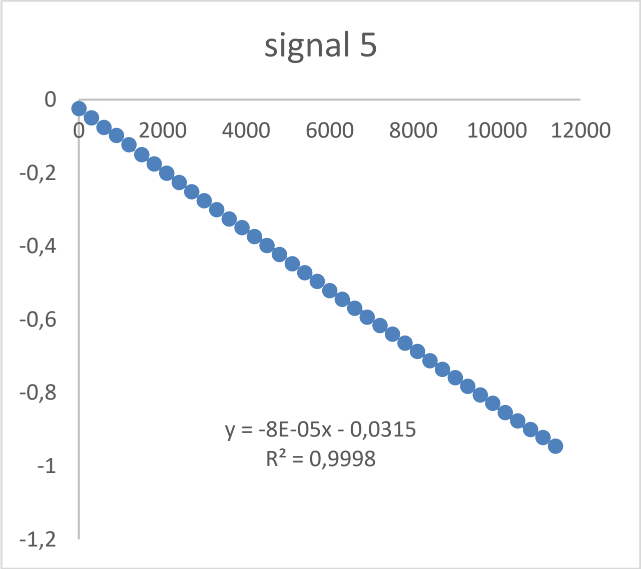

Reaction at 40 °C ($k_{(4)}$ = 3.94 x 10^{-4} s^{-1}, $k_{(5)}$ = 4.00 x 10^{-4} s^{-1}, k_{avg} = 3.97 x 10^{-4} s^{-1}, stdev: 4.26 x 10^{-6})

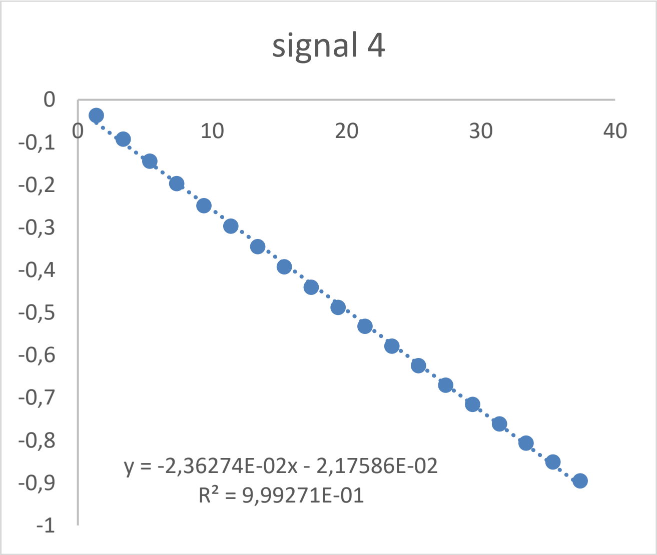

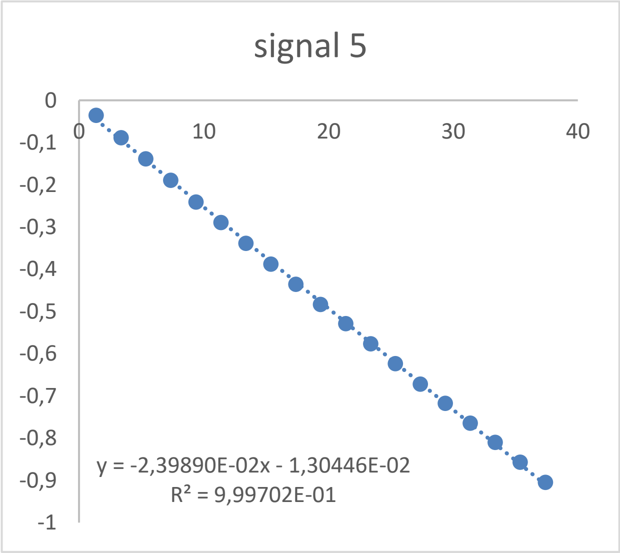

Reaction at 50 °C ($k_{(4)}$ = 9.07 x 10^{-4} s^{-1}, $k_{(5)}$ = 8.85 x 10^{-4} s^{-1}, k_{avg} = 8.96 x 10^{-4} s^{-1}, stdev: 1.58 x 10^{-5})

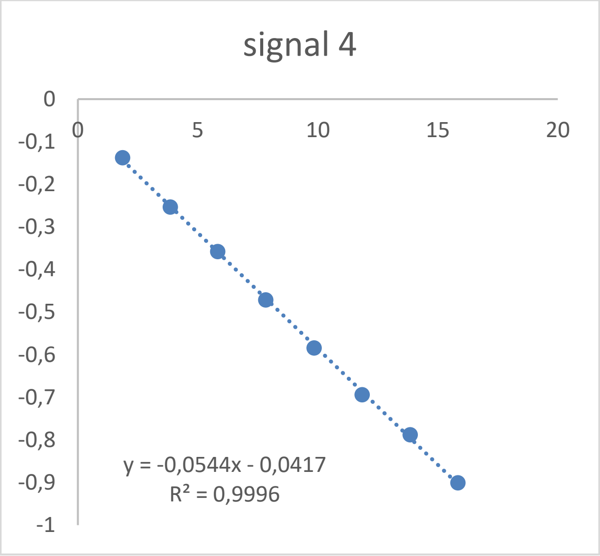

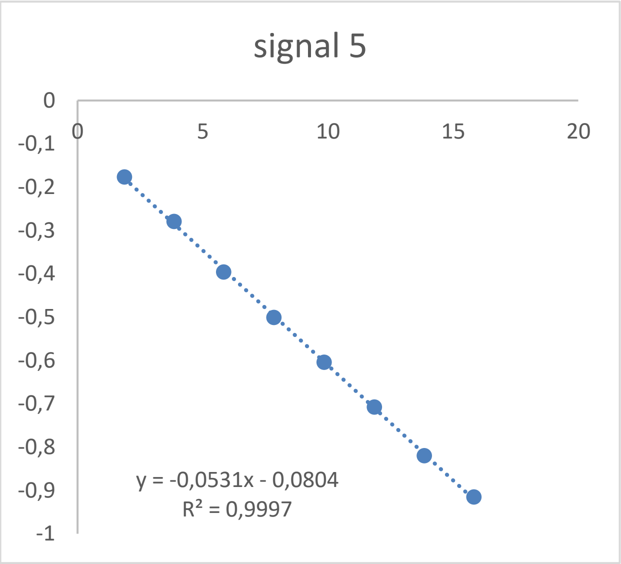

Based on the measured rates, values for ΔH, ΔS and ΔG were obtained by Eyring analysis:

T(°C)	T(K)	1/T	1/(RT)	k (s-1)	(k*h/(kb*T))	ln ((k*h/(kb*T)))
49.9	323.0	0.003096	3.724E-04	8.96E-04	1.33145E-16	-36.5551
39.9	313.0	0.003195	3.843E-04	3.97E-04	6.08421E-17	-37.3382
24.9	298.0	0.003356	4.036E-04	7.91E-05	1.27346E-17	-38.9022
14.9	288.0	0.003472	4.176E-04	2.68E-05	4.46644E-18	-39.9499

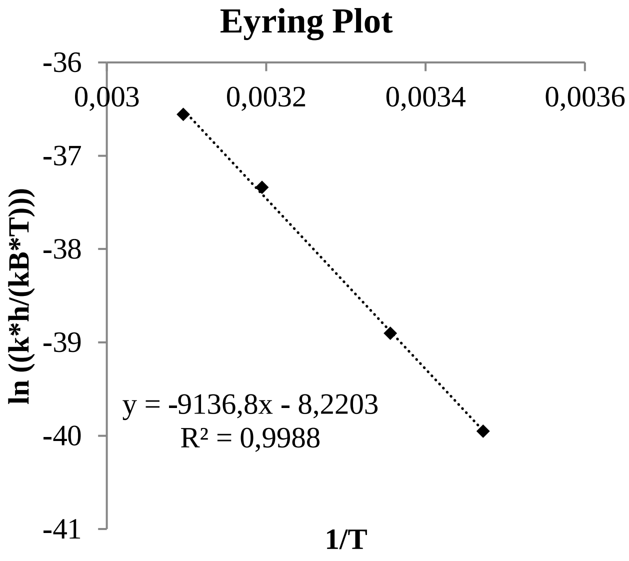

ΔH	76.0	± 1.8	kJ/mol
**ΔH**	**18.1**	**± 0.4**	**kcal / mol**
ΔS	-68.3	± 6.0	J/mol/K
**ΔS**	**16.32**	**± 1.43**	**cal / mol**
ΔG (25°C)	96.3		kJ/mol
**ΔG (25°C)**	**23.0**		**kcal / mol**

Temperature-Dependent KIE Analysis

In order to determine temperature dependent KIE values for the C–H insertion reaction, kinetic analyses were repeated with the deuterated substrate **[D$_3$]-5** under otherwise identical conditions.

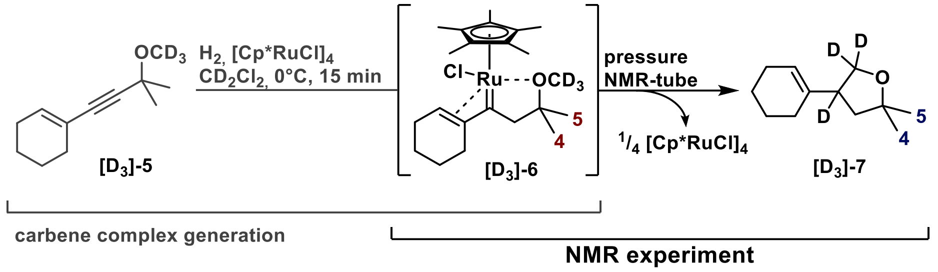

Scheme S3. Experimental setup

Reaction at 15 °C ($k_{(4)}$ = 7.00 x 10^{-6} s^{-1}, $k_{(5)}$ = 6.76 x 10^{-6} s^{-1}, k_{avg} = 6.88 x 10^{-6} s^{-1}, stdev: 1.69 x 10^{-7})

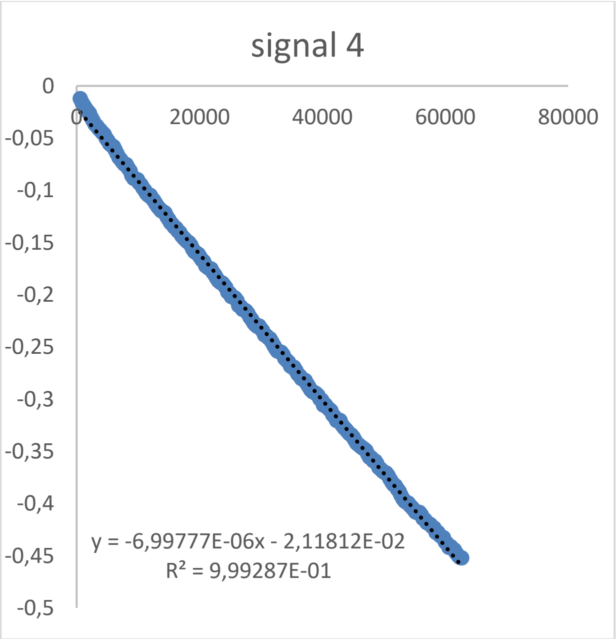

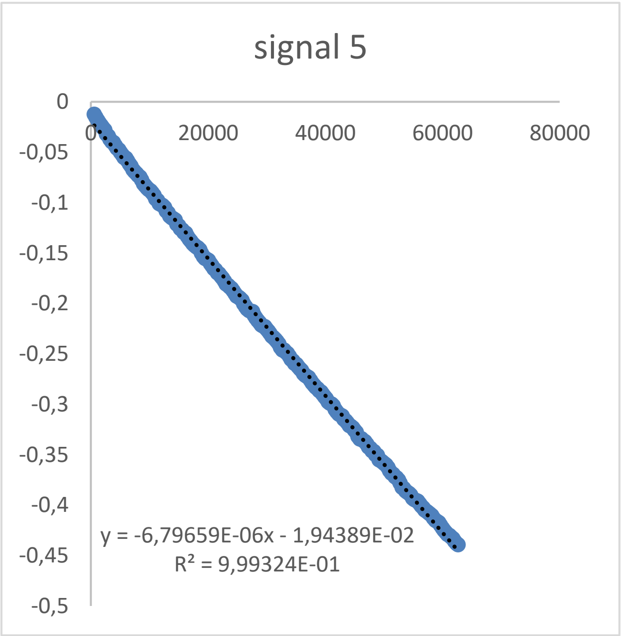

Reaction at 25 °C ($k_{(4)}$ = 2.15 x 10^{-5} s^{-1}, $k_{(5)}$ = 2.10 x 10^{-5} s^{-1}, k_{avg} = 2.13 x 10^{-5} s^{-1}, stdev: 3.39 x 10^{-7})

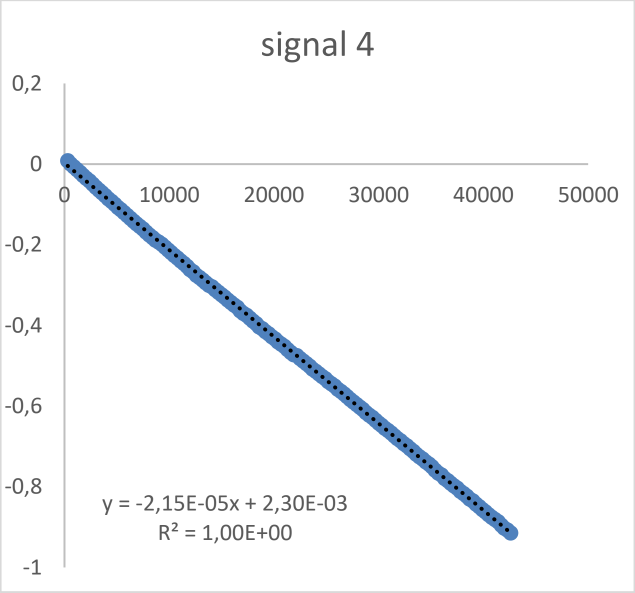

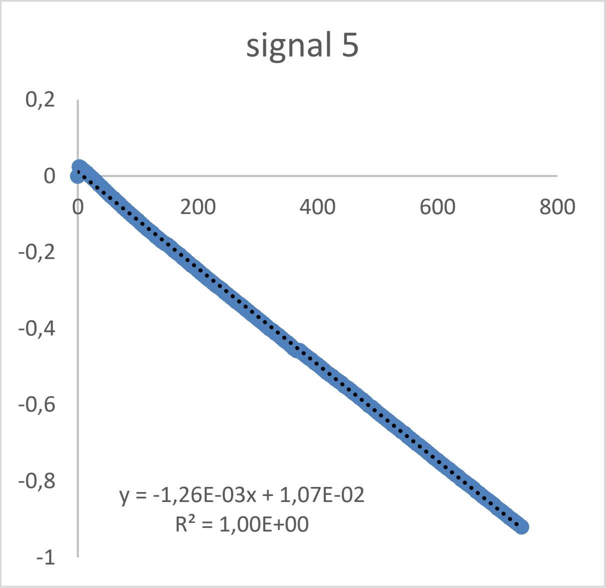

Reaction at 40 °C ($k_{(4)}$ = 1.18 x 10^{-4} s^{-1}, $k_{(5)}$ = 1.08 x 10^{-4} s^{-1}, k_{avg} = 1.13 x 10^{-4} s^{-1}, stdev: 7.19 x 10^{-6})

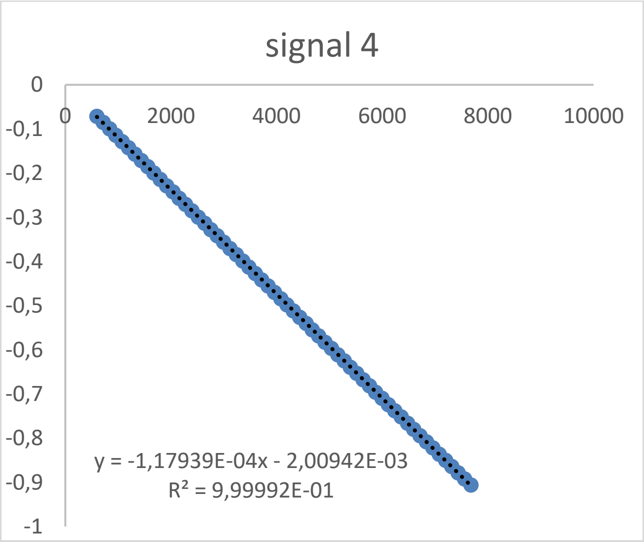

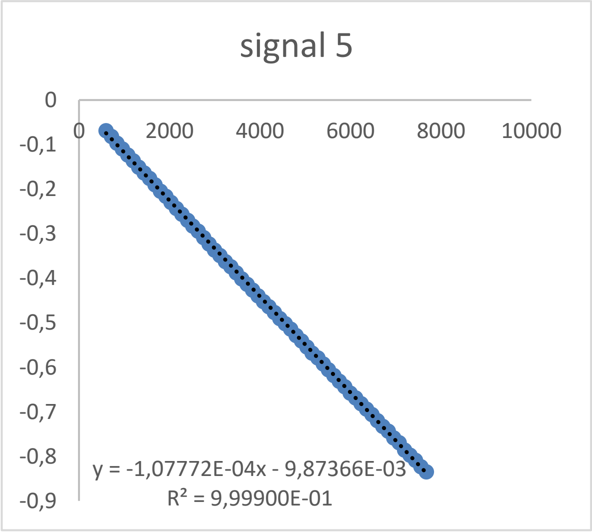

Reaction at 50 °C ($k_{(4)}$ = 2.66 x 10^{-4} s^{-1}, $k_{(5)}$ = 2.74 x 10^{-4} s^{-1}, k_{avg} = 2.70 x 10^{-4} s^{-1}, stdev: 5.92 x 10^{-6})

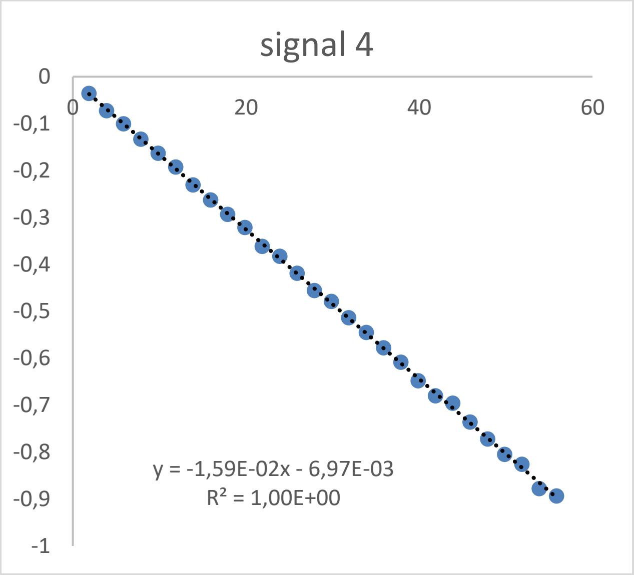

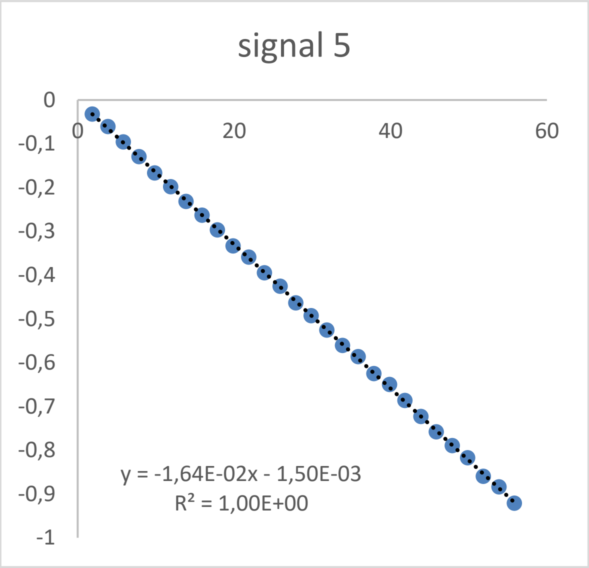

Based on the measured rates, values for ΔH, ΔS and ΔG were obtained by Eyring analysis:

T(°C)	T(K)	1/T	1/(RT)	k (s-1)	(k*h/(kb*T))	ln ((k*h/(kb*T)))
49.9	323.0	0.003096	3.724E-04	2.70E-04	4.0354E-17	-37.7488
39.9	313.0	0.003195	3.843E-04	1.13E-04	1.73185E-17	-38.5948
24.9	298.0	0.003356	4.036E-04	2.19E-05	3.53217E-18	-40.1846
14.9	288.0	0.003472	4.176E-04	6.86E-06	1.14287E-18	-41.313

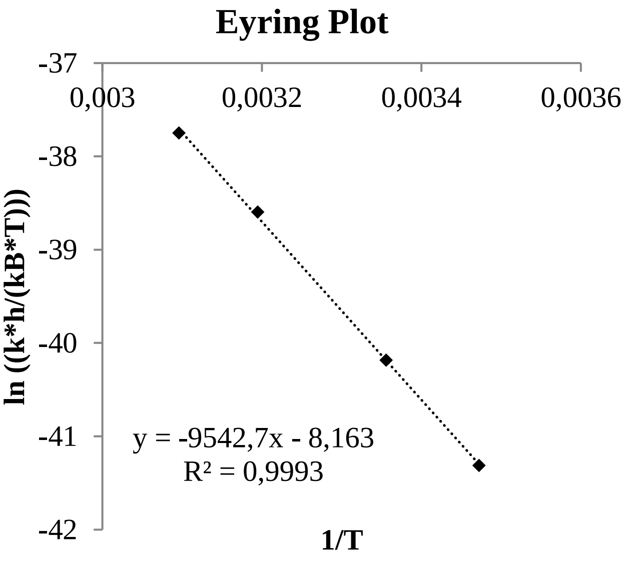

ΔH	79.3	± 1.5	kJ/mol
**ΔH**	**19.0**	**± 0.3**	**kcal / mol**
ΔS	-67.9	± 4.8	J/mol/K
**ΔS**	**-16.21**	**± 1.14**	**cal / mol**
ΔG (25°C)	99.6		kJ/mol
**ΔG (25°C)**	**23.8**		**kcal / mol**

Determination of the Temperature-Dependend KIE Values

Summery of the kinetic data (C–H and C–D insertion reactions):

T (K)	1/T	k_H (s^{-1})		k_D (s^{-1})	
		value	±	value	±
323	0.003096	8.96E-04	1.58E-05	2.70E-04	5.92E-06
313	0.003195	3.97E-04	4.26E-06	1.13E-04	7.19E-06
298	0.003356	7.91E-05	2.52E-06	2.13E-05	3.39E-07
288	0.003472	2.68E-05	1.82E-08	6.88E-06	1.69E-07

Plots of ln(k_H) and ln(k_D) against 1/T give well fitting Arrhenius correlations:

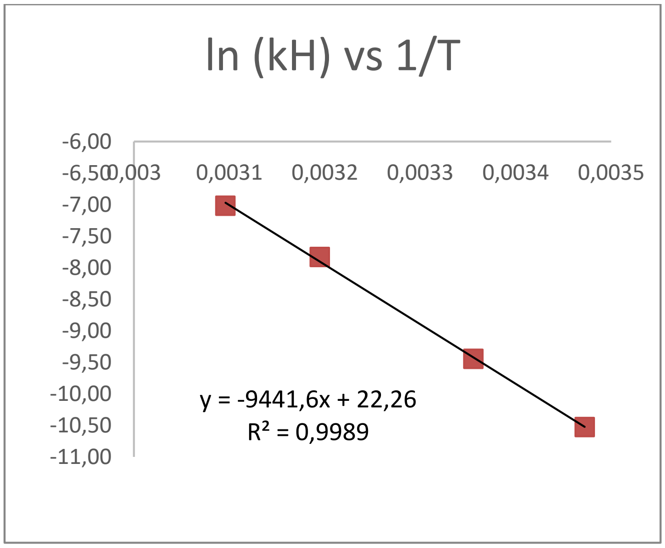

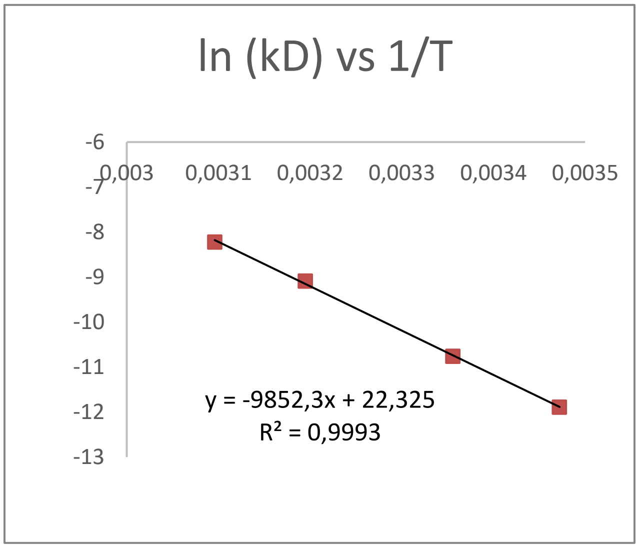

Independent rate KIE values were obtained by division of k_H / k_D :

T (K)	k_H/k_D		ln(k_H)		ln(k_D)		ln(k_H/k_D)	
	value	±	value	±	value	±	value	±
323	3.322335	0.093598	-7.01746	0.017667	-8.21813	0.021944	1.200668	0.028172
313	3.516035	0.227137	-7.83207	0.010738	-9.0894	0.063702	1.257334	0.0646
298	3.716471	0.13606	-9.44514	0.031917	-10.7579	0.017933	1.312775	0.03661
288	3.896657	0.095647	-10.527	0.00068	-11.8871	0.024537	1.360119	0.024546

A plot of ln(k_H/k_D) against 1/T results in a linear correlation, thus indicating a *temperature-dependent kinetic isotope effect*. Values for the activation energy difference $[\Delta E_0]^H{}_D$ and ratio of A-factors (A_H/A_D) can be derived from the linear regression:

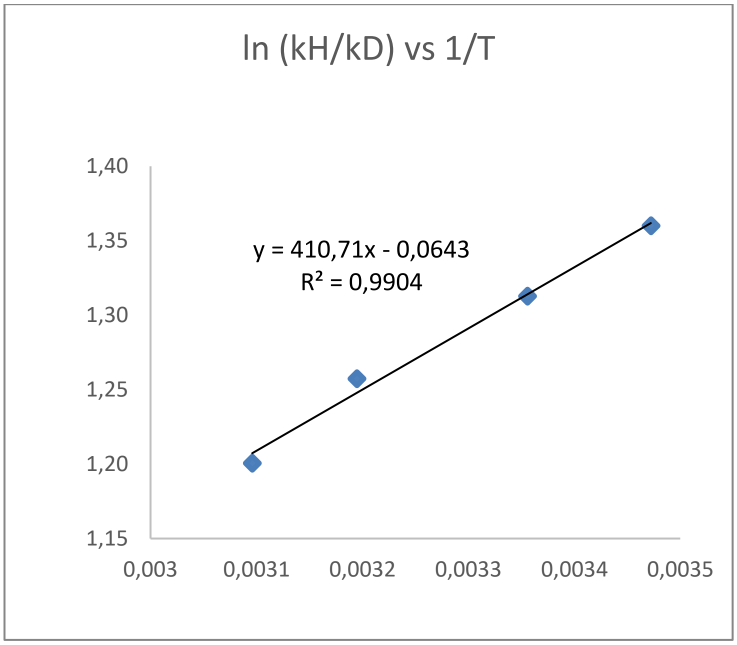

H/D			
-Ea/R	410.71	-0.06	A
±	28.58	0.09	±
ΔEa	-3.41	±0.24	kJ/mol
ΔEa	-0.82	±0.06	kcal/mol
A	0.94	±0.09	

According to Kwart,[5] the key values extracted from this T-dependent KIE study indicate a linear, unsymmetrical H-transfer during the C–H insertion transition state (Table S6). This TS arrangement is consistent with a previously calculated "hydride-transfer-like" C–H insertion reaction for the closely related system shown in Scheme S4.[6]

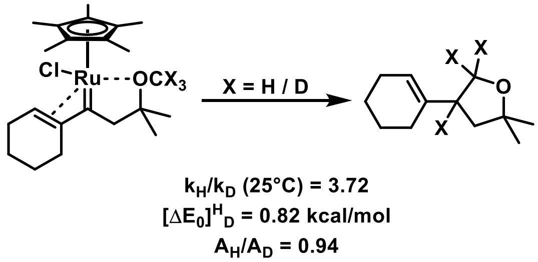

Table S6. Summary of temperature dependent KIE parameters and their corresponding TS classification[5]

$$\frac{k_H}{k_D} = \frac{A_H}{A_D} e^{-[\Delta E_A]_D^H / RT}$$

TS category	k_H/k_D	$[\Delta E_A]_D^H$	A_H/A_D
linear H-transfer symmetrical	"maximum" (~ 6 - 8 at 25°C)	= $[\Delta E_0]_D^H \rightarrow$ CH = 1.15 kcal	1 (0.7 - $2^{1/2}$)
linear H-transfer unsymmetrical	less than maximum (~ 2 - 5 at 25°C)	< $[\Delta E_0]_D^H$ ~ 0.3 - 1 kcal	1 (0.7 - $2^{1/2}$)
linear H-transfer tunneling	more than "maximum" (> 9 at 25°C)	> $[\Delta E_0]_D^H$ ~ 1.5 - 6 kcal	< 0.6
nonlinear H-transfer bent	> $2^{1/2}$	~ 0	$2^{1/2} \rightarrow$ ~ 6

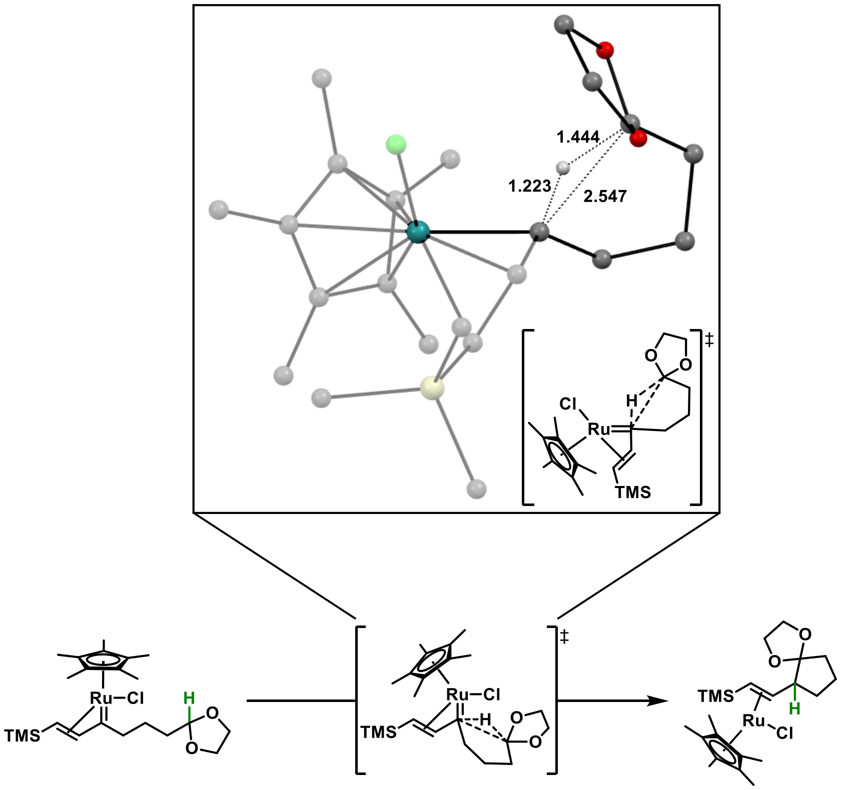

Scheme S4. Calculated transition state for C–H insertion by a closely related Ru carbene complex reported in the literature,[7] with structural parameters showing the almost linear hydride-transfer between the carbene center and the C–H donor.

Catalytic KIE Experiments

Intramolecular competition experiments were carried out to asses the kinetic isotope effect under catalytic conditions. The two different partially deuterated substrates **[D1]-5** and **[D2]-5** (Scheme S5) were reacted with H_2 according to the general catalytic reaction setup. Even though the KIE value of 2.58 at 70 °C differs from the KIE of the stoichiometric reaction (see above), it is qualitatively in line with the conclusions reached above.

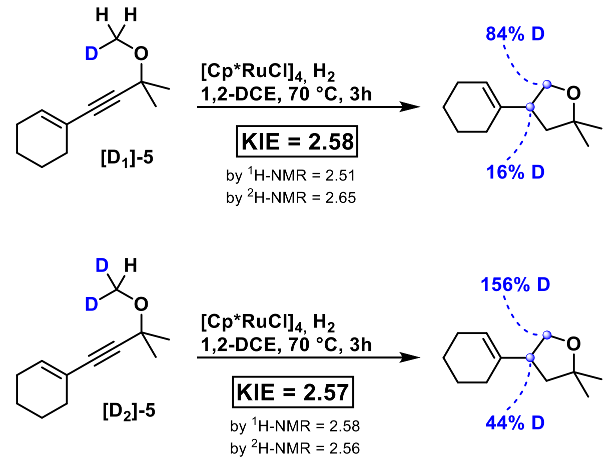

Scheme S5. Intramolecular C–H/C–D competition experiments

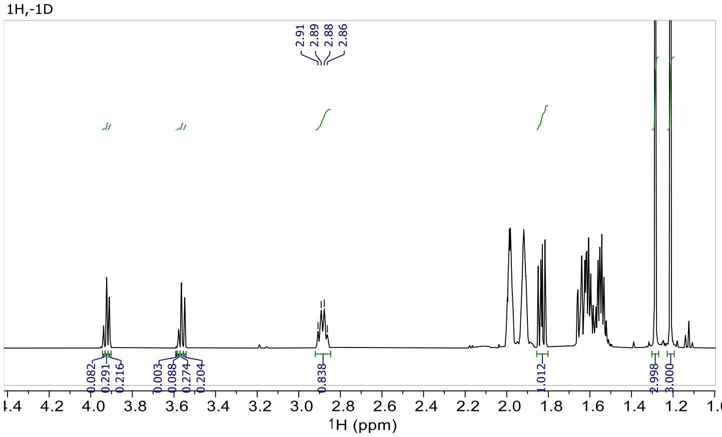

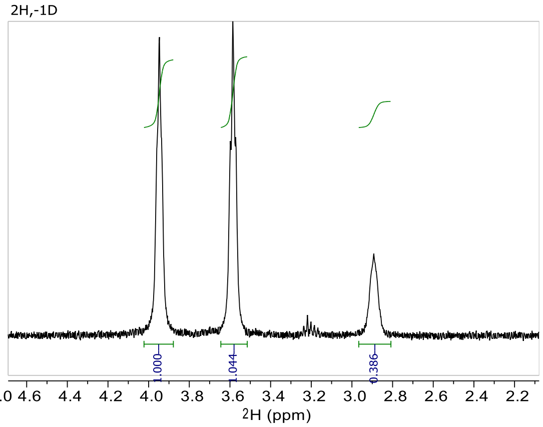

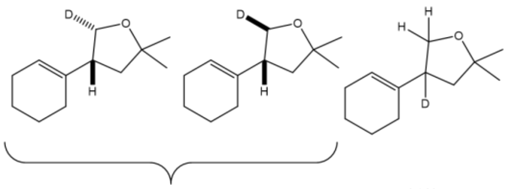

1H: 0.834 0.166

KIE = (0.834/2)/0.166 = 2.51

2H: 1 1.044 0.386

KIE = (2.044/2)/0.386 = 2.65

average KIE = 2.58 ± 0.10

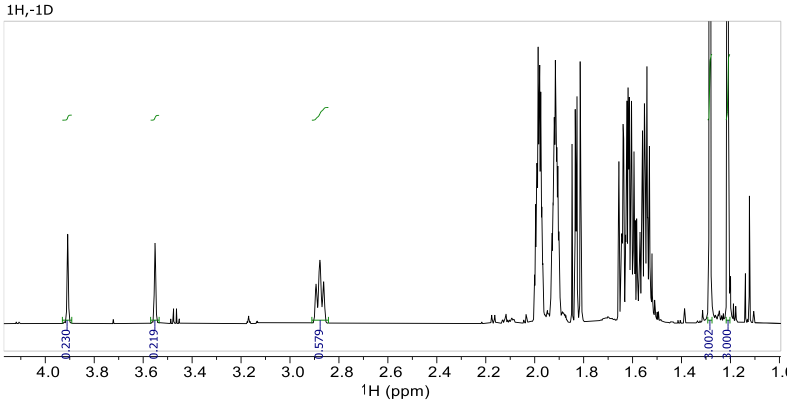

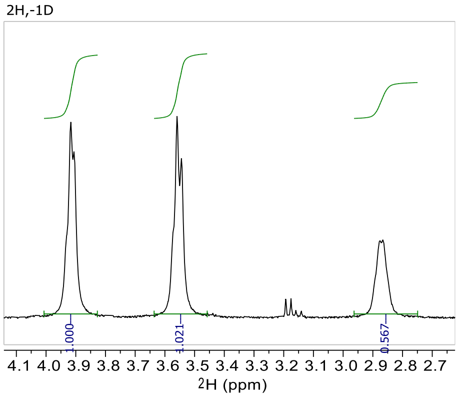

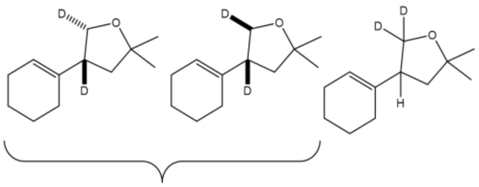

1H: 0.23 0.219 0.58

KIE = 0.579 / ((0.219+0.23)/2)= 2.58

2H: 0.567 (2.021-0.567)/2 = 0.727

KIE = (0.727)/(0.567/2) = 2.56

average KIE = 2.57 ± 0.01

Comparison with Aliphatic and Benzylic Carbene Intermediates

The comparison shown in Scheme S6 highlights the crucial role of the alkenyl moiety next to the transient carbene center formed by *gem*-hydrogenation

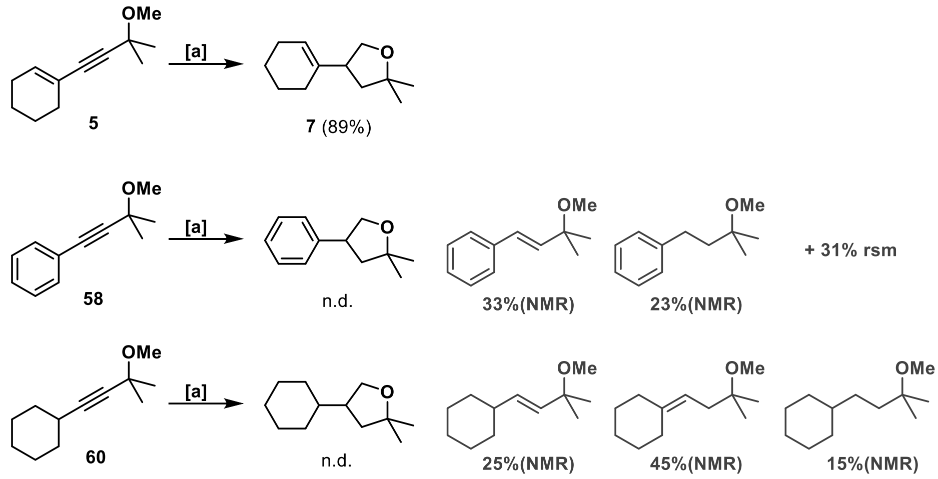

Scheme S6. Comparison of the reactivity of substrates with different degrees of unsaturation. Reagents and conditions: [a] $[Cp^*RuCl]_4$ (2 mol%), H_2 (1 atm, balloon), 1,2-dichloroethane, 70 °C, 3h; n.d.: not detected; rsm = recovered starting material

Independent Generation of a Catalytically Active Vinylcarbene Intermediate

Scheme S7 shows the formation of the same alkenylsilane product **10** by either *gem*-hydrogenation or carbene/alkyne metathesis (CAM).[6,8] This result supports the notion that ruthenium vinylcarbenes generated by *geminal* hydrogenation or carbene-alkyne-metathesis are chemically equivalent.

With regard to the olelfin configuration in **10**, the *gem*-hydrogenation reaction proceeded stereospecifically with retention of the alkene geometry in that ***E*-8** yields exclusively ***E*-10**. In contrast, CAM (with [CpRuCl] and $TMSCH_2N_2$) is believed to initially form a kinetically favored *Z*-configured vinylcarbene intermediate, which equilibrates to the thermodynamically favored *E*-vinylcarbene complex. Depending on the relative rates of *E*/*Z*-isomerization and downstream reactivity (e.g. C–H insertion) *E*/*Z*-mixtures will be obtained.[6,8] Indeed, reaction of alkyne **9** with trimethylsilyldiazomethane yielded **10** as a mixture of the *E*- and *Z*-isomers (i.e. the *E*/*Z*-isomerization muat occur at a rate close to that of the C–H insertion rate in this particular case).

To make sure that a common catalytic intermediate is generated and the outcome of the reactions can hence be directly compared, the CAM experiment was performed in 1,2-dichloroethane as solvent with $[Cp^*RuCl]_4$ as catalyst at 70 °C. Note that these conditions slightly deviate from those reported in the

*literature as being optimal for CAM (dioxane, [Cp*Ru(cod)Cl], 60 °C),[6] which may or may not explain the lower yield.*

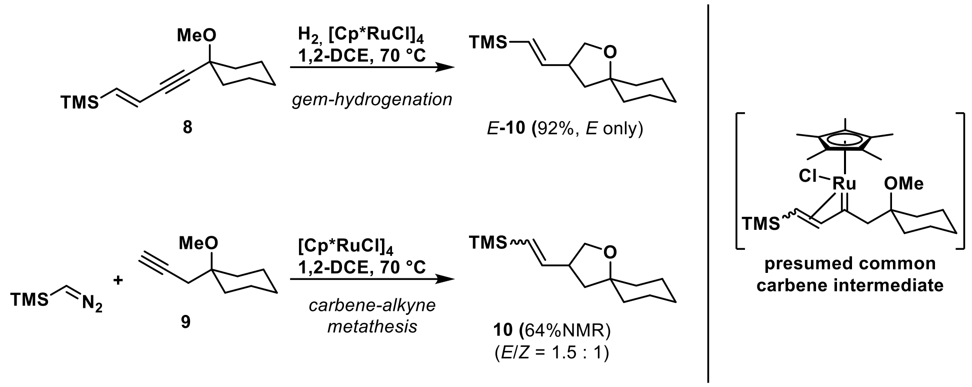

Scheme S7. Independent synthesis of alkenylsilane **10** by *gem*-hydrogenation and carbene-alkyne-metathesis via a common vinylcarbene intermediate

Carbene/Alkyne Metathesis (CAM). $[Cp^*RuCl]_4$ (2.1 mg, 2 mol%) was added to a solution of alkyne **9** (14.6 mg, 0.1 mmol) and trimethylsilyldiazomethane (2 M in hexanes, 72 µL, 0.14 mmol) in 1,2-dichoroethane (1 mL) in a flame dried Schlenk flask at room temperature under argon. The flask was sealed with a rubber septum and immersed into a pre-heated oil bath (70 °C). After 3 h, the mixture was allowed to cool to room temperature and the solvent was removed under reduced pressure. An aliquot of an internal standard stock solution (1,3,5-trimethoxybenzene) was added and the crude product was analyzed by ^1H NMR (see Scheme S8) showing full consumption of the substrate and formaiton of ***E*-10** (38%) as well as ***Z*-10** (26%).

Conducting the reaction at room temperature or with a smaller excess of trimethylsilyldiazomethane resulted in incomplete conversion.

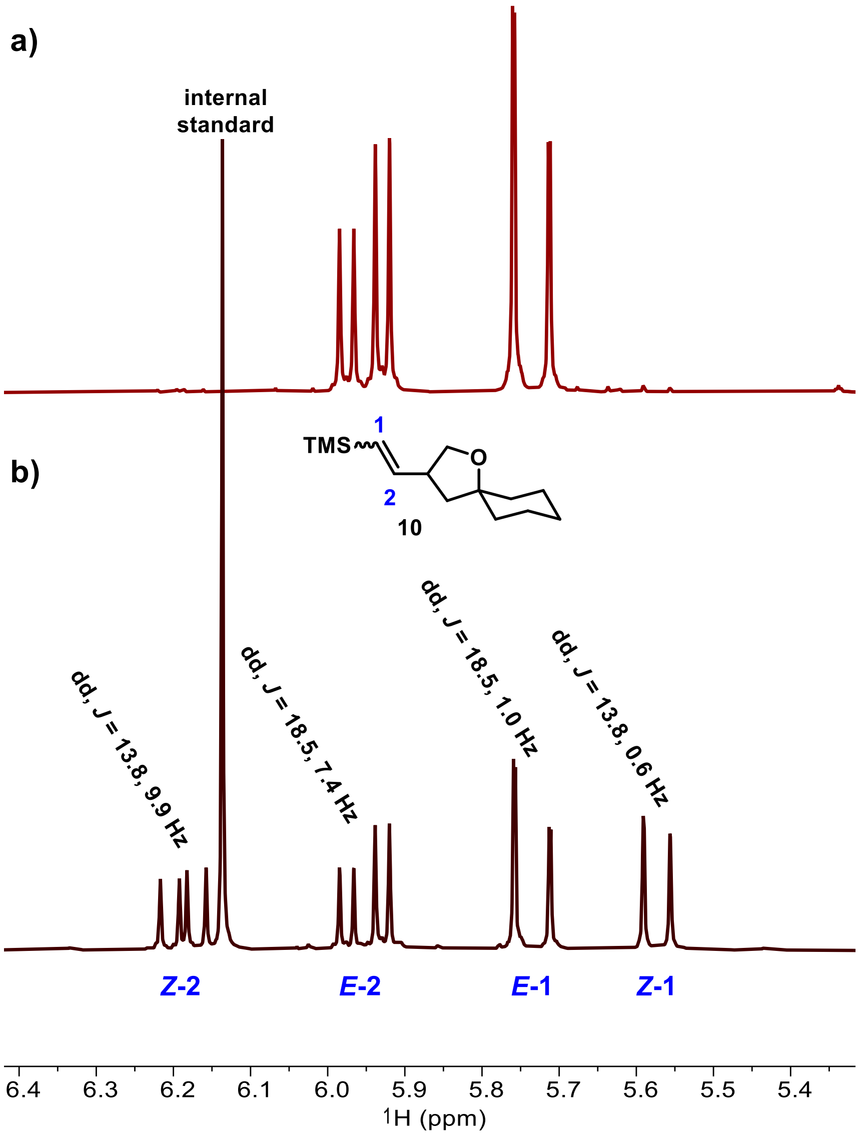

Scheme S8. Stacked ^{1}H NMR excerpts of C–H insertion product **10** generated by *gem*-hydrogenation (a) and carbene/alkyne metathesis (CAM) (b)

Preparation of the Substrates

Non-Commercial Building Blocks

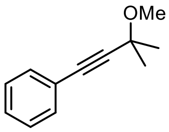

58

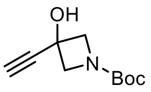

S1

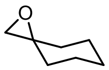

S2

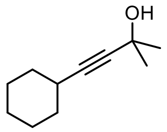

S3

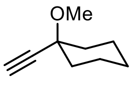

S4

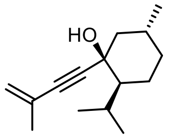

S5

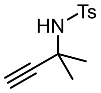

S6

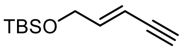

S7

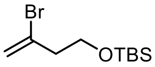

S8

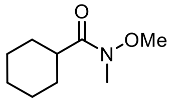

S9

The shown building blocks were prepared according to literature procedures: **58,**[9] **S1,**[10] **S2,**[11] **S3**,[12] **S4,**[13] **S5,**[14] **S6,**[15] **S7**,[16] **S8**,[17] and **S9**[18]

4-(Cyclohex-1-en-1-yl)-2-methylbut-3-yn-2-ol (S10). *n*-BuLi (1.6 M in hexanes, 17.7 mL, 28.3 mmol) was slowly added to a solution of 1-ethynylcyclohex-1-ene (3.00 g, 28.3 mmol) in THF (180 mL) at 0 °C. The mixture was stirred for 30 min at 0 °C before acetone (6.2 mL, 84.8 mmol) in THF (15 mL) was slowly introduced. The mixture was stirred for 10 min at 0 °C before sat. NH_4Cl solution (50 mL) and *tert*-butyl methyl ether (150 mL) were added. The layers were separated and the aqueous layer was extracted with *tert*-butyl methyl ether (2 x 150 mL). The combined organic layers were dried over $MgSO_4$ and the solvent was removed under reduced pressure. The crude product was purified by flash chromatography (silica, hexanes/EtOAc 10:1) to yield the title compound as a colorless oil (4.12 g, 89%). ^{1}H NMR (400 MHz, $CDCl_3$) δ 6.07 (tt, *J* = 3.5, 1.7 Hz, 1H), 2.13 – 2.05 (m, 4H), 1.95 – 1.81 (br, 1H), 1.66 – 1.54 (m, 4H), 1.53 (s, 6H). ^{13}C NMR (101 MHz, $CDCl_3$) δ 135.1, 120.3, 91.3, 84.0, 65.7, 31.8, 29.4, 25.7, 22.4, 21.6. The spectral data match the literature.[14]

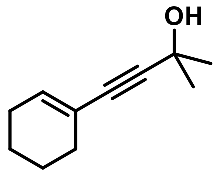

1-(3-Methylbut-3-en-1-yn-1-yl)cyclopentan-1-ol (S11). *n*-BuLi (1.6 M in hexanes, 14.2 mL, 22.7 mmol) was slowly added to a solution of 2-methylbut-1-en-3-yne (1.50 g, 22.7 mmol) in THF (150 mL) at 0 °C. The mixture was stirred for 30 min at 0 °C before a solution of cyclopentanone (3.0 mL, 34.0 mmol) in THF (7 mL) was slowly introduced. The mixture was stirred for 10 min at 0 °C before sat. NH_4Cl solution (50 mL) and *tert*-butyl methyl ether (100 mL) were added. The layers were separated and the aqueous layer was extracted with *tert*-butyl methyl ether (2 x 100 mL). The combined organic layers were dried over $MgSO_4$ and the solvent was removed under reduced pressure. The crude product was purified by flash chromatography (silica, hexanes/EtOAc 10:1) to yield the title compound as a colorless oil (2.47 g, 73%). ^{1}H NMR (400 MHz, $CDCl_3$) δ 5.26 (dq, *J* = 2.1, 1.0 Hz, 1H), 5.20 (p, *J* = 1.6 Hz, 1H), 2.02 – 1.91 (m, 4H), 1.88 (dd, *J* = 1.6, 1.0 Hz, 3H), 1.87 – 1.70 (m, 4H), 1.60 (br, 1H). ^{13}C NMR (101 MHz, $CDCl_3$) δ 126.6, 121.8, 92.0, 84.4, 74.9, 42.6, 23.6, 23.6. The spectral data match the literature.[19]

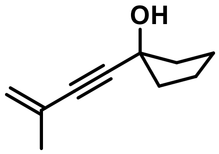

***tert*-Butyl 3-hydroxy-3-(3-methylbut-3-en-1-yn-1-yl)azetidine-1-carboxylate (S12).** *n*-BuLi (1.6 M in hexanes, 5.1 mL, 8.2 mmol) was slowly added to a solution of 2-methylbut-1-en-3-yne (630 mg, 9.5 mmol) in THF (60 mL) at –78 °C. The mixture was stirred for 15 min at –78 °C before 1-Boc-3-azetidinone (1.00 g, 5.8 mmol) in THF (10 mL) was slowly introduced. Stirring was continued at –78 °C for 15 min before the mixture was allowed to warm to –20 °C over 60 min. sat. NH_4Cl solution (40 mL) and EtOAc (40 mL) were added, the layers were separated, and the aqueous phase was extracted with EtOAc (2 x 40 mL). The combined organic layers were dried over $MgSO_4$ and the solvent was removed under reduced pressure. The crude product was purified by flash chromatography (silica, hexanes/EtOAc, 4:1) to obtain the title compound as white solid (1.15 g, 83 %). ^1H NMR (400 MHz, $CDCl_3$) δ 5.32 (dq, *J* = 2.0, 1.0 Hz, 1H), 5.27 (p, *J* = 1.6 Hz, 1H), 4.19 (dd, *J* = 9.0, 1.0 Hz, 2H), 4.04 (dd, *J* = 9.0, 1.1 Hz, 2H), 2.98 (br, 1H), 1.88 (dd, *J* = 1.6, 1.1 Hz, 3H), 1.43 (s, 9H). ^{13}C NMR (101 MHz, $CDCl_3$) δ 156.3, 125.9, 123.3, 87.9, 87.1, 80.2, 64.4, 62.6, 28.5, 23.3. IR (film) ṽ 3341 (br), 2977, 2882, 2249, 1671, 1420, 1367, 1157, 1087 cm^{-1}. HRMS (CI^+) for $C_{13}H_{19}NO_3$ $[M+H]^+$: calcd 238.1438, found 238.1435.

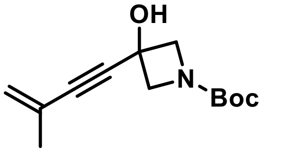

4-(3-Methylbut-3-en-1-yn-1-yl)-1-tosylpiperidin-4-ol (S13). Prepared analogously from 1-tosylpiperidin-4-one (253 mg, 2.0 mmol); colorless oil (362 mg, 57 %). ^1H NMR (400 MHz, $CDCl_3$) δ 7.69 – 7.61 (m, 2H), 7.36 – 7.28 (m, 2H), 5.18 (p, *J* = 1.6 Hz, 1H), 5.15 (dq, *J* = 2.0, 1.0 Hz, 1H), 3.26 (ddd, *J* = 11.3, 7.2, 3.7 Hz, 2H), 3.03 (ddd, *J* = 11.8, 8.1, 3.7 Hz, 2H), 2.43 (s, 3H), 1.99 (dddd, *J* = 13.4, 7.3, 3.7, 1.1 Hz, 2H), 1.93 – 1.82 (m, 3H), 1.75 (t, *J* = 1.3 Hz, 3H). ^{13}C NMR (101 MHz, $CDCl_3$) δ 143.7, 133.3, 129.8, 127.9, 125.8, 122.6, 89.6, 86.9, 65.9, 43.2, 38.5, 23.3, 21.6. IR (film) ṽ 3383 (br) 3478, 2958, 2929, 2861, 1615, 1340, 11168, 1083 cm^{-1}. HRMS (ESI^+) for $C_{17}H_{22}NO_3Na$ $[M+Na]^+$: calcd 320.1315, found 320.1317.

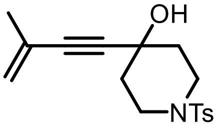

4-(3-Methylbut-3-en-1-yn-1-yl)tetrahydro-2*H*-pyran-4-ol (S14). Prepared analogously from tetrahydro-4*H*-pyran-4-one (417 mg, 4.2 mg); colorless oil (481 mg, 70%). ^1H NMR (400 MHz, $CDCl_3$) δ 5.31 (dq, *J* = 2.0, 1.0 Hz, 1H), 5.25 (p, *J* = 1.6 Hz, 1H), 3.91 (dt, *J* = 12.0, 4.7 Hz, 2H), 3.65 (ddd, *J* = 11.9, 9.1, 2.9 Hz, 2H), 1.94 (dddd, *J* = 13.0, 4.9, 3.1, 1.7 Hz, 2H), 1.90 (dd, *J* = 1.6, 1.0 Hz, 3H), 1.82 (ddd, *J* = 13.1, 9.3, 4.1 Hz, 2H). ^{13}C NMR (101 MHz, $CDCl_3$) δ 126.2, 122.5, 90.4, 86.5, 66.3, 65.1, 40.2, 23.6. IR (film) ṽ 3385 (br), 2965, 2861, 1614, 1427, 1338, 1132, 1094 cm^{-1}.HRMS (ESI^+) for $C_{10}H_{14}O_2Na$ $[M+Na]^+$: calcd. 189.0886, found 189.0887.

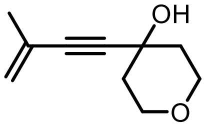

3-(3-Methylbut-3-en-1-yn-1-yl)tetrahydrofuran-3-ol (S15). Prepared analogously from tetrahydrofuran-3-on (230 mg, 2.7 mmol); colorless oil (391 mg, 96 %). ^1H NMR (400 MHz, $CDCl_3$) δ 5.31 (dq, *J* = 2.0, 1.0 Hz, 1H), 5.26 (p, *J* = 1.6 Hz, 1H), 4.13 – 3.94 (m, 2H), 3.93 – 3.82 (m, 2H), 2.38 – 2.20 (m, 3H), 1.89 (dd, *J* = 1.6, 1.0 Hz, 3H). ^{13}C NMR (101 MHz, $CDCl_3$) δ 126.1, 122.9, 87.6, 86.4, 80.2, 73.6, 67.8, 42.6, 23.4. IR (film) ṽ 3383 (br), 2954, 2876, 1809, 1614, 1297, 1240, 1048 cm^{-1}. HRMS (ESI^+) for $C_9H_{12}O_2Na$ $[M+Na]^+$: calcd 175.0730, found 175.0729.

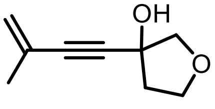

1-Cyclohexyl-4-methylpent-4-en-2-yn-1-one (S16). *n*-BuLi (1.6 M in hexanes, 1.7 mL, 2.7 mmol) was slowly added to a solution of 2-methylbut-1-en-3-yne (200 mg, 3.0 mmol) in THF (10 mL) at –78 °C. The mixture was stirred for 15 min at this temperature before a solution of N-methoxy-N-methylcyclohexanecarboxamide (**S9**) (430 mg, 2.5 mmol) in THF (3 mL) was slowly added. Stirring was continued at –78 °C for 15 min and for 60 min at

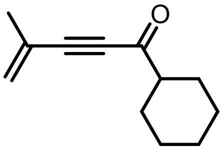

−20 °C. NH_4Cl solution (20 mL) and *tert*-butyl methyl ether (20 mL) were added, the layers were separated, and the aqueous phase was extracted with *tert*-butyl methyl ether (2 x 20 mL). After evaporation of the solvents, the crude product was purified by flash chromatography (silica, hexanes/ *tert*-butyl methyl ether, 98:2) to yield the title compound as a pale yellow oil (377 mg, 85 %). ^1H NMR (400 MHz, $CDCl_3$) δ 5.58 (dq, *J* = 2.2, 1.1 Hz, 1H), 5.50 (p, *J* = 1.6 Hz, 1H), 2.42 (tt, *J* = 11.2, 3.6 Hz, 1H), 2.03 – 1.93 (m, 5H), 1.84 – 1.74 (m, 2H), 1.71 – 1.61 (m, 1H), 1.50 – 1.13 (m, 5H). ^{13}C NMR (101 MHz, $CDCl_3$) δ 191.7, 127.4, 125.1, 92.4, 86.1, 52.4, 28.4, 25.9, 25.5, 22.7. ṽ 3385 (br), 2929, 2855, 2192, 1665, 1449, 1290, 11149, 1109 cm^{-1}. HRMS (CI^+) for $C_{12}H_{17}O$ $[M+H]^+$: calcd 177.1274, found 177:1276.

***tert*-Butyl 3-ethynyl-3-methoxyazetidine-1-carboxylate (S17).** A solution of alcohol **S1** (3.05 g, 15.5 mmol) in THF (5 mL) was slowly added to a suspension of NaH (334 mg, 13.9 mmol) in THF (60 mL) and DMF (10 mL) at room temperature. The mixture was stirred for 30 min before a solution of MeI (1.1 mL, 17.0 mmol) in DMF (10 mL) was carefully added. After stirring for antoher 10 min, sat. NH_4Cl solution (25 mL) and EtOAc (100 mL) were introduced and the layers were separated. The aqueous phase was extracted with EtOAc (2 x 100 mL) and the combined organic layers were washed with brine (2 x 15 mL) and dried over $MgSO_4$. The solvent was evaporated and the residue was purified by flash chromatography (silica, hexanes/EtOAc 10:1 – 5:1) to yield the title compound as a colorless oil (2.85 g, 97% (*wrt. NaH*)). ^1H NMR (400 MHz, $CDCl_3$) δ 4.08 (dd, *J* = 9.1, 1.0 Hz, 2H), 3.99 (dd, *J* = 9.0, 1.0 Hz, 2H), 3.34 (s, 3H), 2.66 (s, 1H), 1.44 (s, 9H). ^{13}C NMR (101 MHz, $CDCl_3$) δ 156.2, 81.1, 80.1, 75.9, 67.8, 61.2 (br), 52.7, 28.5. IR (film) ṽ 3237, 2976, 2113, 1697, 1391, 1163, 1095 cm^{-1}. HRMS (ESI^+) for $C_{11}H_{17}NO_3$ $[M+Na]^+$: calcd 234.1101, found 234.1100.

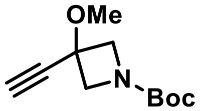

3,3-Dimethylpent-4-yn-1-ol (S18). A solution of *n*-butyllithium in hexanes (1.6 M, 18 mL, 29 mmol) was slowly added to a solution of 3-methyl-1-butyne (1.5 mL, 15 mmol) in diethyl ether (5 mL) at 0 °C. When the addition was complete, tetramethylethylenediamine (2.2 mL, 15 mmol) was introduced, which led to the formation of a pale yellow/white slurry. The mixture was then stirred at reflux temperature (65 °C bath temperature) for 2 h, leading to the formation of a homogeneous red solution. The mixture was cooled to −78 °C, and a solution of ethylene oxide in THF (ca. 5 M, 3.2 mL, 16 mmol) was slowly added via syringe. When the addition was complete and the red color had disappeared, the mixture was further stirred for 10 min at −78 °C, before it was warmed to 0 °C. Water (20 mL) and diethyl ether (15 mL) were introduced, and the mixture was allowed to warm to room temperature. The layers were separated and the organic layer was washed with water (3 × 10 mL) and brine (10 mL). The aqueous phases were extracted with diethyl ether (3 × 10 mL) and the combined organic layers were dried over anhydrous sodium sulfate. The drying agent was filtered off, and the solvent was carefully removed under reduced pressure (200 mbar, 35 °C). Purification of the residue by flash chromatography (silica, pentane/diethyl ether, 2:1 → 1:1) furnished the title compound as a pale yellow liquid (633 mg, 38%). ^1H NMR (400 MHz, $CDCl_3$) δ 3.86 (t, *J* = 6.6 Hz, 2H), 2.16 (s, 1H), 1.73 (t, *J* = 6.6 Hz, 2H), 1.26 (s, 6H). The analytical data are in agreement with those reported in the literature.[20]

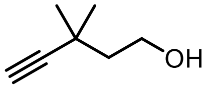

Sonogashira Coupling Reactions

***tert*-Butyl (2,5-dimethylhex-5-en-3-yn-2-yl)carbamate (S19).** NEt_3 (0.57 mL, 4.1 mmol), CuI (52 mg, 0.27 mmol) and $Pd(PPh_3)_4$ (157 mg, 0.14 mmol) were added to a stirred solution of 2-bromoprop-1-ene (500 mg, 4.1 mmol) and *tert*-butyl (2-methylbut-3-yn-2-yl)carbamate (250 mg, 1.36 mmol) in DMF (7 mL). The mixture was stirred for 2 h at room temperature before sat. NH_4Cl solution (7 mL) and *tert*-butyl methyl ether (15 mL) were introduced. The layers were separated and the aqueous phase was extracted with *tert*-butyl methyl ether (2 x 25 mL). The combined organic layers were washed with sat. NaCl solution (2 x 10 mL) and dried over $MgSO_4$. The solvent was removed under reduced pressure and the residue was purified by flash chromatography (silica, hexanes/EtOAc 20:1 – 10:1) to yield the title compound as a colorless oil (165 mg, 54%). ^{1}H NMR (400 MHz, $CDCl_3$) δ 5.23 (dt, *J* = 2.1, 1.1 Hz, 1H), 5.17 (tt, *J* = 1.6, 0.9 Hz, 1H), 4.72 (br, 1H), 1.89 – 1.83 (dd, *J* = 1.5, 1.0 Hz, 3H), 1.58 (s, 6H), 1.46 (s, 9H). ^{13}C NMR (101 MHz, $CDCl_3$) δ 154.5 (br), 126.8, 121.5, 92.3, 82.0, 79.7 (br), 47.9, 29.6 (br), 28.5, 23.7. IR (film) ṽ 3359, 3266, 2977, 2930, 1697, 1489, 1364, 1158 cm^{-1}. HRMS (CI^+) for $C_{13}H_{21}NO_2$ $[M+H]^+$: calcd 224.1645, found 224.1646.

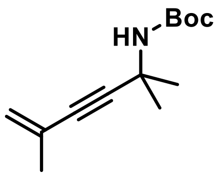

N-(2,5-Dimethylhex-5-en-3-yn-2-yl)-4-methylbenzenesulfonamide (S20). Prepared analogously from propargylamine **S6** (2.00 g, 8.43 mmol) as a pale yellow oil (1.85 g, 79%). ^{1}H NMR (400 MHz, $CDCl_3$) δ 7.82 – 7.77 (m, 2H), 7.28 – 7.23 (m, 2H), 5.08 – 5.06 (m, 1H), 5.01 (br, 1H), 4.93 (dt, *J* = 2.0, 1.1 Hz, 1H), 2.39 (s, 3H), 1.62 (dd, *J* = 1.6, 1.0 Hz, 3H), 1.56 (s, 6H). ^{13}C NMR (101 MHz, $CDCl_3$) δ 143.1, 138.9, 129.5, 127.7, 126.2, 122.0, 89.8, 84.4, 50.6, 31.2, 23.2, 21.6. IR (film) ṽ 3261 (br), 2983, 2256, 1382, 1320, 1145, 1093 cm^{-1}. HRMS (ESI^+) for $C_{15}H_{19}NO_2S$ $[M+Na]^+$: calcd 300.1029, found 300.1030.

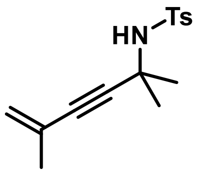

3,3,6-Trimethylhept-6-en-4-yn-1-ol (S21). CuI (43.0 mg, 0.226 mmol) and $[(Ph_3P)_2PdCl_2]$ (80.0 mg, 0.114 mmol) were added to a solution of 2-bromopropene (0.75 mL, 8.4 mmol) and diisopropylamine (1.2 mL, 8.6 mmol) in THF (15 mL), and the resulting mixture was cooled to 0 °C (ice bath). A solution of alkyne **S18** (633 mg, 5.64 mmol) in THF (5 mL) was introduced. The mixture was stirred for 15 min at 0 °C, the cooling bath was removed and stirring continued for 15 h at ambient temperature. The mixture was diluted with pentane (20 mL) and washed with half-saturated aqueous ammonium chloride solution (30 mL) and water (3 × 10 mL). The aqueous phases were extracted with pentane (3 × 10 mL), the combined organic layers were dried over anhydrous sodium sulfate, and the solvent was carefully removed under reduced pressure (200 mbar, 35 °C). Purification of the residue by flash chromatography (silica, pentane/diethyl ether, 1:0 $\rightarrow$ 2:1) furnished the title compound as a yellow liquid (783 mg, 91%). ^{1}H NMR (400 MHz, $CDCl_3$) δ 5.22 – 5.11 (m, 2H), 3.85 (t, *J* = 6.6 Hz, 2H), 1.85 (s, 3H), 1.73 (t, *J* = 6.6 Hz, 2H), 1.26 (s, 6H). ^{13}C NMR (101 MHz, $CDCl_3$) δ 126.8, 120.8, 95.5, 82.5, 60.6, 45.5, 29.8, 29.7, 23.8. IR (film) ṽ 3333 (br), 3097, 2969, 2927, 1178, 1614, 1456, 1372, 1316, 1245, 1202, 1141, 1062, 1028, 990, 892 cm^{-1}. HRMS (EI^+) for $C_{10}H_{16}O$ $[M]^+$: calcd 152.1196, found 152.1198.

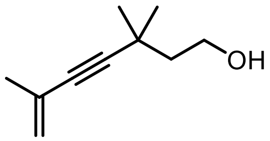

Substrates for *gem*-Hydrogenation/C–H Insertion

Via Alkylation

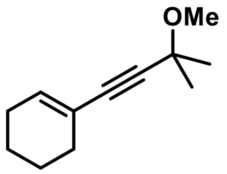

1-(3-Methoxy-3-methylbut-1-yn-1-yl)cyclohex-1-ene (5). A solution of alcohol **S10** (300 mg, 1.8 mmol) in THF (1 mL) was added to a stirred suspension of NaH (109 mg, 4.6 mmol) in THF (7 mL) at 0 °C. Stirring was continued for 30 min at room temperature before MeI (0.57 mL, 9.1 mmol) was introduced. After stirring for another 1 h, sat. NH_4Cl solution (3 mL), water (6 mL) and *tert*-butyl methyl ether (30 mL) were added and the layers separated. The aqueous phase was extracted with *tert*-butyl methyl ether (2 x 30 mL) and the combined organic layers were washed with brine and dried over $MgSO_4$. The solvent was removed under reduced pressure and the crude product was purified by flash chromatography (silica, hexanes/EtOAc 1:0 – 40:1) to give the title compound as a colorless oil (210 mg, 64%). ^1H NMR (400 MHz, $CDCl_3$) δ 6.09 (m, 1H), 3.35 (s, 3H), 2.16 – 2.02 (m, 4H), 1.68 – 1.53 (m, 4H), 1.46 (s, 6H). ^{13}C NMR (101 MHz, $CDCl_3$) δ 134.9, 120.4, 88.3, 86.2, 71.0, 51.7, 29.5, 28.6, 25.7, 22.4, 21.6. IR (film) ṽ 2982, 2931, 2214, 1255, 1171, 1075 cm^{-1}. HRMS (ESI^+) for $C_{12}H_{18}O$ $[M+Na]^+$: calcd 201.1250, found 201.1250.

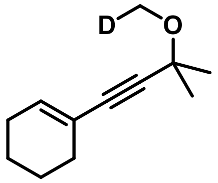

(3-(Methoxy-[D])-3-methylbut-1-yn-1-yl)cyclohex-1-ene ([D_1]-5). Prepared analogously using mono-deutero-methyl iodide; colorless oil (287 mg, 88%). ^1H NMR (400 MHz, $CDCl_3$) δ 6.09 (tt, *J* = 3.7, 1.7 Hz, 1H), 3.34 – 3.33 (t, *J* = 1.6 Hz, 2H), 2.14 – 2.05 (m, 4H), 1.67 – 1.53 (m, 4H), 1.45 (s, 6H). ^{13}C NMR (101 MHz, $CDCl_3$) δ 134.9, 120.4, 88.3, 86.2, 71.0, 51.4 (t, *J* = 21.5 Hz), 29.5, 28.6, 25.7, 22.4, 21.6. IR (film) ṽ 2929, 2211, 2143, 1359, 1252, 1160, 1078 cm^{-1}. HRMS (EI^+) for $C_{12}H_{17}DO$ $[M]^+$: calcd 179.1415, found 179.1414.

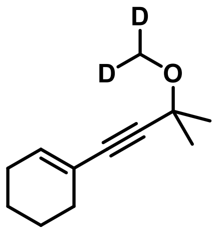

1-(3-(Methoxy-[D_2])-3-methylbut-1-yn-1-yl)cyclohex-1-ene ([D_2]-5). Prepared analogously using dideutero-methyl iodide; colorless oil (282 mg, 86%). ^1H NMR (400 MHz, $CDCl_3$) δ 6.08 (tt, *J* = 3.7, 1.7 Hz, 1H), 3.32 (p, *J* = 1.6 Hz, 1H), 2.15 – 2.05 (m, 4H), 1.67 – 1.53 (m, 4H), 1.45 (s, 6H). ^{13}C NMR (101 MHz, $CDCl_3$) δ 134.8, 120.4, 88.3, 86.2, 70.9, 51.1 (p, *J* = 21.6 Hz), 29.5, 28.6, 25.7, 22.4, 21.6. IR (film) ṽ 2930, 2860, 2216, 2134, 1359, 1252, 1161, 1088 cm^{-1}. HRMS (EI^+) for $C_{12}H_{16}D_2O$ $[M]^+$: calcd 180.1478, found 180.1477.

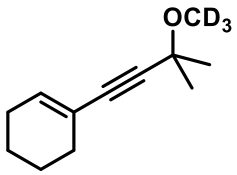

1-(3-([D_3]-Methoxy)-3-methylbut-1-yn-1-yl)cyclohex-1-ene ([D_3]-5). Prepred analogously using CD_3I; colorless oil (250 mg, 76%). ^1H NMR (400 MHz, $CDCl_3$) δ 6.09 (tt, *J* = 3.8, 1.7 Hz, 1H), 2.16 – 2.03 (m, 4H), 1.67 – 1.53 (m, 4H), 1.45 (s, 6H). ^{13}C NMR (101 MHz, $CDCl_3$) δ 134.9, 120.4, 88.4, 86.1, 70.9, 29.5, 28.7, 25.7, 22.4, 21.6; (signal of $–CD_3$ not detected). IR (film) ṽ 2983, 2931, 2263, 1359, 1253, 1164, 1114, 1045 cm^{-1}. HRMS (EI^+) for $C_{12}H_{15}D_3O$ $[M]^+$: calcd 181.1540, found 181.1540.

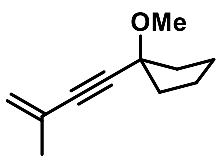

1-Methoxy-1-(3-methylbut-3-en-1-yn-1-yl)cyclopentane (S22). A solution of alcohol **S11** (280 mg, 1.9 mmol) in THF (1 mL) was added to a stirred suspension of NaH (112 mg, 4.7 mmol) in THF (7 mL) at 0 °C. The mixture was stirred for 30 min at room temperature before MeI (0.58 mL, 9.3 mmol) was introduced. After stirring for 1 h at room temperature, sat. NH_4Cl solution (3 mL), water (6 mL) and *tert*-butyl methyl ether (30 mL) were

added and the layers separated. The aqueous phase was extracted with *tert*-butyl methyl ether (2 x 30 mL) and the combined organic layers were washed with brine and dried over $MgSO_4$. The solvent was removed under reduced pressure and the crude product was purified by flash chromatography (silica, hexanes/EtOAc 1:0 – 40:1) to give the title compound as a colorless oil (225 mg, 73%). ^{1}H NMR (400 MHz, $CDCl_3$) δ 5.27 (dq, *J* = 2.1, 1.0 Hz, 1H), 5.21 – 5.19 (m, 1H), 3.33 (s, 3H), 2.07 – 1.97 (m, 2H), 1.92 – 1.82 (m, 2H), 1.89 (dd, *J* = 1.6, 1.0 Hz, 3H), 1.78 – 1.67 (m, 4H). ^{13}C NMR (101 MHz, $CDCl_3$) δ 126.8, 121.6, 89.6, 86.3, 81.1, 52.1, 39.2, 23.8, 23.5. IR (film) ṽ 2968, 2874, 1615, 1436, 1291, 1073 cm^{-1}. HRMS (ESI$^+$) for $C_{11}H_{16}O$ $[M+Na]^+$: calcd 187.1093, found 187.1094.

1-((1-Methoxycyclobutyl)ethynyl)cyclohex-1-ene (S23). Prepared analogously from cyclobutanone and 1-ethynylcyclohex-1-ene; colorless oil (230 mg, 94%).

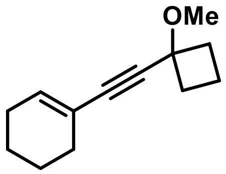

^{1}H NMR (400 MHz, $CDCl_3$) δ 6.12 (tt, *J* = 3.9, 1.8 Hz, 1H), 3.27 (s, 3H), 2.36 – 2.28 (m, 2H), 2.28 – 2.18 (m, 2H), 2.17 – 2.05 (m, 4H), 1.92 – 1.76 (m, 2H), 1.68 – 1.53 (m, 4H). ^{13}C NMR (101 MHz, $CDCl_3$) δ 135.0, 120.5, 87.6, 86.8, 73.8, 51.7, 35.7, 29.5, 25.8, 22.5, 21.6, 13.3. IR (film) ṽ 2988, 2934, 2210, 1436, 1282, 1124, 1042 cm^{-1}. HRMS (EI$^+$) for $C_{13}H_{18}O$ $[M]^+$: calcd 190.1352, found 190.1352.

(1S,2S,5R)-2-Isopropyl-1-methoxy-5-methyl-1-(3-methylbut-3-en-1-yn-1-yl)cyclohexane (S24). A solution of alcohol **S5** (175 mg, 0.79 mmol) in DMF (1 mL) was added to a stirred suspension of NaH (48 mg, 2.0 mmol) in DMF (4 mL) at room temperature. The mixture was stirred for 20 min before MeI (0.25 mL, 1.6 mmol) was introduced. After stirring for another 2 h, sat. NH_4Cl solution (6 mL), water (3 mL) and *tert*-butyl methyl ether (15 mL) were added and the layers separated. The aqueous phase was extracted with *tert*-butyl methyl ether (2 x 30 mL) and the combined organic layers were washed with brine (3 x 10 mL) and dried over $MgSO_4$. The solvent was removed under reduced pressure and the crude product was purified by flash chromatography (silica, hexanes/EtOAc 1:0 – 100:1) to give the title compound as a colorless oil (25.9 mg, 14%).

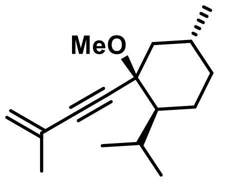

^{1}H NMR (400 MHz, $CDCl_3$) δ 5.25 (dq, *J* = 2.1, 1.0 Hz, 1H), 5.20 – 5.18 (m, 1H), 3.27 (s, 3H), 2.33 (heptd, *J* = 6.9, 2.1 Hz, 1H), 2.21 (ddd, *J* = 14.2, 3.4, 2.3 Hz, 1H), 1.89 (dd, *J* = 1.6, 1.0 Hz, 3H), 1.73 (dtd, *J* = 12.5, 3.5, 2.3 Hz, 1H), 1.62 – 1.41 (m, 3H), 1.33 (ddd, *J* = 12.1, 4.1, 2.1 Hz, 1H), 1.08 (dd, *J* = 14.2, 12.3 Hz, 1H), 0.94 (d, *J* = 7.0 Hz, 3H), 0.92 – 0.86 (m, 1H), 0.89 (d, *J* = 6.9 Hz, 3H), 0.86 (d, *J* = 6.6 Hz, 3H). ^{13}C NMR (101 MHz, $CDCl_3$) δ 126.9, 121.3, 90.6, 87.0, 77.7, 51.6, 50.7, 43.7, 35.1, 28.8, 27.1, 24.1, 23.9, 22.1, 20.9, 18.9. IR (film) ṽ 2950, 2873, 1456, 1288, 1081 cm^{-1}. HRMS (CI$^+$) for $C_{16}H_{26}O$ $[M+H]^+$: calcd 235.2056, found 235.2054.

1-(3-(Methoxymethoxy)-3-methylbut-1-yn-1-yl)cyclohex-1-ene (S25). A solution of alcohol **S10** (300 mg, 1.8 mmol) in THF (1 mL) was added to a stirred suspension of NaH (88 mg, 3.7 mmol) in THF (7 mL) at 0 °C. The mixture was stirred for 30 min at room temperature before MOMCl (0.28 mL, 3.7 mmol) was slowly introduced at 0 °C. After stirring for 18 h at room temperature, sat. NH_4Cl solution (3 mL), water (6 mL) and *tert*-butyl methyl ether (30 mL) were added and the layers separated. The aqueous phase was extracted with *tert*-butyl methyl ether (2 x 30 mL) and the combined organic layers were washed with brine and dried over $MgSO_4$. The solvent was removed under reduced pressure and the crude product was purified by flash chromatography (silica, hexanes/EtOAc 1:0 – 30:1) to give the title compound as a colorless oil (169 mg, 44%). ^{1}H NMR (400 MHz, $CDCl_3$) δ 6.08 (tt, *J* = 3.8, 1.6 Hz, 1H), 4.90 (s, 2H), 3.39 (s, 3H), 2.09 (m,

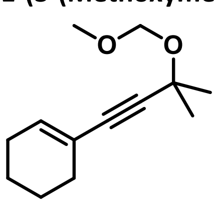

4H), 1.66 – 1.53 (m, 4H), 1.52 (s, 6H). ^{13}C NMR (101 MHz, $CDCl_3$) δ 135.1, 120.3, 93.3, 88.2, 86.7, 71.6, 55.6, 30.5, 29.4, 25.7, 22.4, 21.6. IR (film) ṽ 2983, 2931, 2212, 1257, 1144, 1032 cm^{-1}. HRMS (ESI$^+$) for $C_{13}H_{20}O_2$ $[M+Na]^+$: calcd 231.1355, found 231.1355.

***tert*-Butyl 3-(2-(*tert*-butoxy)-2-oxoethoxy)-3-(3-methylbut-3-en-1-yn-1-yl)azetidine-1-carboxylate (S26).**

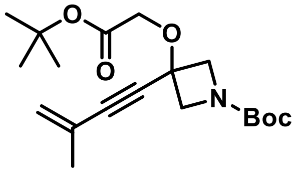

A solution of alcohol **S12** (200 mg, 0.84 mmol) in DMF (1 mL) was added to a stirred suspension of NaH (51 mg, 2.1 mmol) in DMF (4 mL) at room temperature. The mixture was stirred for 20 min before *tert*-butyl 2-bromoacetate (0.31 mL, 2.1 mmol) was introduced. After stirring for 15 min at room temperature, sat. NH_4Cl solution (3 mL), water (3 mL) and EtOAc (15 mL) were added and the layers were separated. The aqueous phase was extracted with EtOAc (2 x 15 mL) and the combined organic layers were washed with brine (2 x 10 mL) and dried over $MgSO_4$. The solvent was removed under reduced pressure and the crude product was purified by flash chromatography (silica, hexanes/EtOAc 10:1) to give the title compound as a colorless oil (271 mg, 91%). ^{1}H NMR (400 MHz, $CDCl_3$) δ 5.35 (dq, J = 2.1, 1.1 Hz, 1H), 5.30 (p, J = 1.6 Hz, 1H), 4.14 (d, J = 9.3 Hz, 2H), 4.10 (dd, J = 8.9, 0.8 Hz, 2H), 4.06 (s, 2H), 1.89 (dd, J = 1.6, 1.0 Hz, 3H), 1.48 (s, 9H), 1.43 (s, 9H). ^{13}C NMR (101 MHz, $CDCl_3$) δ 168.7, 156.2, 125.7, 123.7, 89.8, 84.6, 82.1, 80.1, 68.3, 64.2, 62.3 (br), 28.5, 28.2, 23.3. IR (film) ṽ 2978, 2931, 2255, 1703, 1392, 1105 cm^{-1}. HRMS (ESI$^+$) for $C_{19}H_{29}NO_5$ $[M+Na]^+$: calcd 374.1938, found 374.1936.

7-Methoxy-2,5,5-trimethylhept-1-en-3-yne (25). A solution of alcohol **S21** (772 mg, 5.07 mmol) in

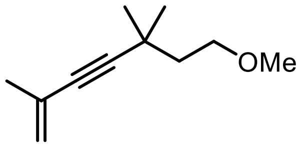

THF (10 mL) was added to a suspension of NaH (337 mg, 14.0 mmol) in THF (12 mL) at 0 °C (ice bath). The cooling bath was removed and the mixture stirred at ambient temperature for 30 min. Methyl iodide (1.6 mL, 26 mmol) was introduced at 0 °C and stirring continued for another 3 h. For work-up, half-saturated aqueous ammonium chloride solution (30 mL) was carefully added at 0 °C and the mixture was diluted with pentane (20 mL). The layers were separated and the organic phase was washed with water (3 × 10 mL). The aqueous phases were extracted with pentane (3 × 10 mL), and the combined organic layers were dried over anhydrous sodium sulfate. The drying agent was filtered off, and the solvent was carefully removed under reduced pressure (200 mbar, 35 °C). Purification of the residue by flash chromatography (silica, pentane/diethyl ether, 1:0 $\rightarrow$ 25:1) furnished the title compound as a yellow liquid (833 mg, 99% yield). ^{1}H NMR (400 MHz, $CDCl_3$) δ 5.20 – 5.11 (m, 2H), 3.60 – 3.54 (m, 2H), 3.35 (s, 3H), 1.88 – 1.85 (m, 3H), 1.75 – 1.69 (m, 2H), 1.24 (s, 6H). ^{13}C NMR (101 MHz, $CDCl_3$) δ 127.4, 120.5, 95.6, 82.0, 70.6, 58.7, 42.4, 30.3, 29.8, 24.1. IR (film) ṽ 3096, 2970, 2925, 2872, 2809, 1614, 1453, 1372, 1313, 1207, 1119, 1067, 966, 892 cm^{-1}. HRMS (EI$^+$) for $C_{11}H_{18}O$ $[M]^+$: calcd 166.1352, found 166.1352.

(3-Methoxy-3-methylbut-1-yn-1-yl)cyclohexane (60). A solution of alcohol **S3** (300 mg, 1.80 mmol) in THF

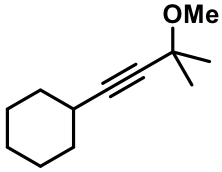

(1 mL) was added to a stirred suspension of NaH (108 mg, 4.51 mmol) in THF (7 mL) at 0 °C. The mixture was stirred for 30 min at room temperature before MeI (0.56 mL, 9.0 mmol) was introduced. After stirring for another 1 h, sat. NH_4Cl solution (3 mL), water (6 mL) and *tert*-butyl methyl ether (30 mL) were added and the layers separated. The aqueous phase was extracted with *tert*-butyl methyl ether (2 x 30 mL) and the combined organic layers were washed with brine and dried over $MgSO_4$. The solvent was removed under reduced pressure and the crude product was purified by flash chromatography (silica,

hexanes/EtOAc 1:0 – 40:1) to give the title compound as a colorless oil (268 mg, 82%). ^{1}H NMR (400 MHz, $CDCl_3$) δ 3.34 (s, 3H), 2.40 (tt, *J* = 9.0, 3.7 Hz, 1H), 1.76 (m, 2H), 1.73 – 1.64 (m, 2H), 1.52 – 1.39 (m, 3H), 1.42 (s, 6H), 1.30 (m, 3H). ^{13}C NMR (101 MHz, $CDCl_3$) δ 89.0, 81.9, 70.7, 51.5, 32.9, 29.0, 28.8, 26.1, 24.9. IR (film) ṽ 2982, 2929, 2854, 2222, 1448, 1250, 1171, 1077 cm^{-1}. HRMS (CI^+) for $C_{12}H_{20}O$ $[M+H]^+$: calcd 181.1587, found 181.1584.

Via One-Pot Alkynylation/O-Alkylation

***tert*-Butyl 3-methoxy-3-(3-methylbut-3-en-1-yn-1-yl)azetidine-1-carboxylate (S27).** *n*-BuLi (1.6 M in hexanes, 1.07 mL, 1.72 mmol) was added to a stirred solution of 2-methyl-1-buten-3-yne (122 mg, 1.84 mmol) in THF (7.4 mL) at –78 °C under argon and the resulting mixture was stirred for 15 min at that temperature. A solution of 1-Boc-3-azetidinone (210 mg, 1.23 mmol) in THF (2.0 mL) was slowly added and the mixture was stirred for 15 min before it was allowed to warm to –20 °C over the course of 1 h. A solution of MeI (0.31 mL, 4.91 mmol) in DMSO (4.8 mL) was added and the mixture was allowed to reach room temperature within 1 h. The reaction was quenched by addition of sat. NH_4Cl solution (6 mL) and water (6 mL) and the biphasic mixture was extracted with EtOAc (3 x 30 mL). The combined organic layers were washed with brine (2 x 10 mL), dried over $MgSO_4$ and the solvent was removed under reduced pressure. The crude product was purified by flash chromatography (silica, hexanes/EtOAc 20:1 – 10:1) to obtain the title compound as a colorless oil (270 mg, 88%). *When performed on larger scale, the yield of product was even higher* (2.16 g, 92%).

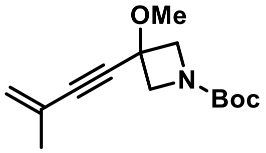

^{1}H NMR (400 MHz, $CDCl_3$) δ 5.34 (dq, *J* = 1.9, 1.0 Hz, 1H), 5.29 (p, *J* = 1.6 Hz, 1H), 4.08 (dd, *J* = 8.9, 1.0 Hz, 2H), 4.01 (dd, *J* = 8.9, 1.0 Hz, 2H), 3.34 (s, 3H), 1.90 (dd, *J* = 1.6, 1.0 Hz, 3H), 1.44 (s, 9H). ^{13}C NMR (101 MHz, $CDCl_3$) δ 156.3, 125.9, 123.2, 88.9, 85.2, 80.0, 68.3, 61.6, 52.7, 28.5, 23.4. IR (film) ṽ 2976, 2882, 2220, 1703, 1390, 1164, 1097 cm^{-1}. HRMS (CI^+) for $C_{14}H_{21}NO_3$ $[M-H]^-$: calcd 250.1438, found 250.1433.

***tert*-Butyl 3-(methoxy-[D_3])-3-(3-methylbut-3-en-1-yn-1-yl)azetidine-1-carboxylate ([D_3]-S27).** Prepared analogously from 1-Boc-3-azetidinone and CD_3I; colorless oil (276 mg, 88%). ^{1}H NMR (300 MHz, $CDCl_3$) δ 5.34 (m, 1H), 5.28 (m, 1H), 4.08 (dd, *J* = 8.9, 1.0 Hz, 2H), 4.00 (dd, *J* = 8.8, 1.0 Hz, 2H), 1.90 (dd, *J* = 1.7, 1.0 Hz, 3H), 1.44 (s, 9H). ^{13}C NMR (101 MHz, $CDCl_3$) δ 156.3, 125.9, 123.2, 88.8, 85.3, 80.0, 68.2, 61.6, 28.5, 23.4; (signal of –CD_3 not detected). IR (film) ṽ 2976, 2882, 2211, 2065, 1703, 1391, 1165, 1098 cm^{-1}. HRMS (ESI^+) for $C_{14}H_{18}D_3NO_3$ $[M+Na]^+$: calcd 277.1602, found 277.1600.

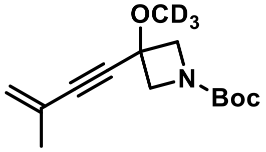

4-Methoxy-4-(3-methylbut-3-en-1-yn-1-yl)-1-tosylpiperidine (S28). Prepared analogously from 1-tosylpiperidin-4-one; colorless solid (332 mg, 79%). ^{1}H NMR (400 MHz, $CDCl_3$) δ 7.67 – 7.63 (m, 2H), 7.35 – 7.30 (m, 2H), 5.17 (p, *J* = 1.6 Hz, 1H), 5.14 (dq, *J* = 2.0, 1.0 Hz, 1H), 3.28 (s, 3H), 3.21 (ddd, *J* = 11.3, 7.2, 3.7 Hz, 2H), 3.01 (ddd, *J* = 11.8, 8.0, 3.6 Hz, 2H), 2.43 (s, 3H), 1.97 (dddd, *J* = 13.5, 7.2, 3.6, 1.1 Hz, 2H), 1.87 (ddd, *J* = 13.1, 8.1, 3.8 Hz, 2H), 1.75 (dd, *J* = 1.5, 1.0 Hz, 3H). ^{13}C NMR (101 MHz, $CDCl_3$) δ 143.6, 133.3, 129.7, 127.8, 126.0, 122.3, 88.9, 87.0, 71.5, 51.1, 43.0, 35.5, 23.4, 21.6. IR (film) ṽ 2944, 2856, 1703, 1466, 1338, 1161, 1088 cm^{-1}. HRMS (EI^+) for $C_{18}H_{23}NO_3S$ $[M-H]^+$: calcd 332.1315, found 332.1316.

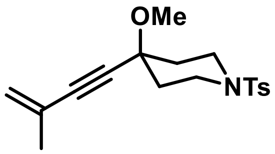

4-Methoxy-4-(3-methylbut-3-en-1-yn-1-yl)tetrahydro-2*H*-pyran (38). Prepared analogously from tetrahydro-4*H*-pyran-4-one; colorless oil (164 mg, 73%). ^{1}H NMR (400 MHz, $CDCl_3$) δ 5.31 (dq, *J* = 2.0, 1.0 Hz, 1H), 5.25 (p, *J* = 1.6 Hz, 1H), 3.88 (dt, *J* = 12.1, 4.7 Hz, 2H), 3.65 (ddd, *J* = 11.9, 9.4, 2.8 Hz, 2H), 3.38 (s, 3H), 1.98 – 1.92 (m, 2H), 1.91 (dd, *J* = 1.6, 1.1 Hz, 3H), 1.79 (ddd, *J* = 13.2, 9.5, 4.0 Hz, 2H). ^{13}C NMR (101 MHz, $CDCl_3$) δ 126.3, 122.3, 88.5, 87.7, 71.9, 64.9, 50.8, 37.3, 23.7. IR (film) ṽ 2956, 2856, 1466, 1432, 1304, 1152, 1096 cm^{-1}. HRMS (EI$^+$) for $C_{11}H_{16}O_2$ [M]$^+$: calcd 180.1145, found 180.1145.

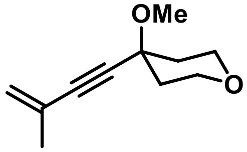

5-Methoxy-2,2-dimethyl-5-(3-methylbut-3-en-1-yn-1-yl)-1,3-dioxane (S29). Prepared analogously from 2,2-dimethyl-1,3-dioxan-5-one; pale yellow oil (198 mg, 76%). ^{1}H NMR (400 MHz, $CDCl_3$) δ 5.33 (dq, *J* = 2.1, 1.1 Hz, 1H), 5.27 (p, *J* = 1.6 Hz, 1H), 3.94 (s, 4H), 3.43 (s, 3H), 1.89 (dd, *J* = 1.6, 1.1 Hz, 3H), 1.47 (q, *J* = 0.6 Hz, 3H), 1.44 (q, *J* = 0.6 Hz, 3H). ^{13}C NMR (101 MHz, $CDCl_3$) δ 125.9, 123.3, 98.4, 89.5, 83.8, 69.5, 65.6, 51.9, 27.3, 23.5, 19.9. IR (film) ṽ 2990, 2942, 1452, 1372, 1198, 1086 cm^{-1}. HRMS (ESI$^+$) for $C_{12}H_{18}O_3$ [M+Na]$^+$: calcd 233.1148, found 233.1147.

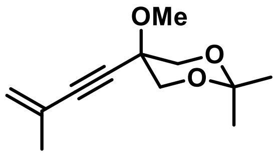

***tert*-Butyl 6-Methoxy-6-(3-methylbut-3-en-1-yn-1-yl)-2-azaspiro[3.3]heptane-2-carboxylate (S30).** Prepared analogously from the corresponding spiroketone; pale yellow oil (336 mg, 93%). ^{1}H NMR (400 MHz, $CDCl_3$) δ 5.28 (dq, *J* = 2.0, 1.0 Hz, 1H), 5.23 (p, *J* = 1.7 Hz, 1H), 4.02 (s, 2H), 3.89 (s, 2H), 3.26 (s, 3H), 2.59 – 2.52 (m, 2H), 2.42 – 2.36 (m, 2H), 1.88 (dd, *J* = 1.6, 1.0 Hz, 3H), 1.43 (s, 9H). ^{13}C NMR (101 MHz, $CDCl_3$) δ 156.3, 126.3, 122.3, 88.3, 86.9, 79.5, 69.3, 61.7, 60.6, 52.1, 46.4, 30.6, 28.5, 23.5. IR (film) ṽ 2976, 2933, 1700, 1391, 1174, 1081 cm^{-1}. HRMS (CI$^+$) for $C_{17}H_{25}NO_3$ [M+H]$^+$: calcd 292.1907, found 292.1903.

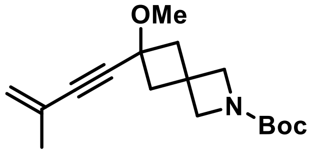

***tert*-Butyl (*E*)-3-(5-((*tert*-butyldimethylsilyl)oxy)pent-3-en-1-yn-1-yl)-3-methoxyazetidine-1-carboxylate (S31).** Prepared analogously from 1-Boc-3-azetidinone and (*E*)-*tert*-butyldimethyl(pent-2-en-4-yn-1-yloxy)silane (**S7**); colorless oil (400 mg, 89 %).^{1}H NMR (400 MHz, $CDCl_3$) 6.26 (dt, *J* = 15.8, 4.1 Hz, 1H), 5.81 (dt, *J* = 15.7, 2.3 Hz, 1H), 4.24 (dd, *J* = 4.1, 2.3 Hz, 2H), 4.08 (dd, *J* = 8.9, 1.0 Hz, 2H), 4.00 (dd, *J* = 8.9, 1.0 Hz, 2H), 3.33 (s, 3H), 1.44 (s, 9H), 0.91 (s, 9H), 0.07 (s, 6H). ^{13}C NMR (101 MHz, $CDCl_3$) δ 156.3, 144.0, 107.6, 86.5, 86.0, 80.0, 68.4, 62.9, 61.7, 52.7, 28.5, 26.0, 18.5, –5.2. IR (film) ṽ 2950, 2857, 2215, 1705, 1391, 1252, 1106 cm^{-1}. HRMS (ESI$^+$) for $C_{20}H_{35}NO_4Na$ [M+Na]$^+$: calcd 404.2228, found 404.2229.

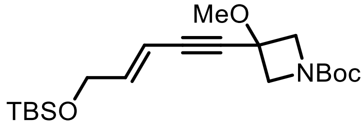

Preparation of *tert*-Butyl 3-methoxy-3-(3-methylbut-3-en-1-yn-1-yl)pyrrolidine-1-carboxylate (S32). Prepared analogously from N-Boc-3-pyrrolidinone (185 mg, 1.0 mmol); colorless oil (127 mg, 48 %). ^{1}H NMR (400 MHz, $CDCl_3$) δ 5.31 (dq, *J* = 2.0, 1.0 Hz, 1H), 5.26 (p, *J* = 1.6 Hz, 1H), 3.68 (dd, *J* = 38.0, 11.8 Hz, 1H), 3.57 – 3.27 (m, 6H), 2.24 (s, 1H), 2.19 – 2.01 (m, 1H), 1.89 (dd, *J* = 1.6, 1.0 Hz, 3H), 1.46 (s, 9H). IR (film) ṽ 2976, 2889, 1695, 1395, 1126 cm^{-1}. HRMS (ESI$^+$) for $C_{15}H_{23}NO_3Na$ [M+Na]$^+$: calcd 288.1570, found 288.1670.

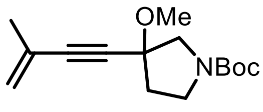

3-Methoxy-3-(3-methylbut-3-en-1-yn-1-yl)tetrahydrofuran (S33). Prepared analogously from

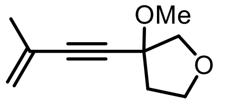

tetrahydrofuran-3-one (225 mg, 2.6 mmol); colorless oil (329 mg, 76 %). ^{1}H NMR (400 MHz, $CDCl_3$) δ 5.31 (dq, *J* = 2.0, 1.0 Hz, 1H), 5.25 (p, *J* = 1.6 Hz, 1H), 4.05 – 3.90 (m, 3H), 3.77 (d, *J* = 9.4 Hz, 1H), 3.38 (s, 3H), 2.32 (dddd, *J* = 12.7, 6.8, 4.7, 1.1 Hz, 1H), 2.25 – 2.14 (m, 1H), 1.90 (dd, *J* = 1.6, 1.0 Hz, 3H). IR (film) ṽ 2948, 2875, 1615, 1437, 1298, 1126, 1072 cm^{-1}. HRMS (ESI$^+$) for $C_{10}H_{14}O_2Na$ $[M+Na]^+$: calcd 189.0886, found 189.0887.

1-Methoxy-1-(prop-2-yn-1-yl)cyclohexane (9). Oxirane **S2** (1.29 g, 11.5 mmol) was slowly added to a

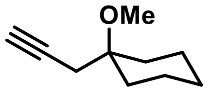

stirred suspension of lithium acetylide ethylenediamine complex (1.38 g, 15.0 mmol) in DMSO (12 mL) at 0 °C. The mixture was allowed to reach room temperature and stirring was continued for 5 d before sat. NH_4Cl solution (20 mL) and *tert*-butyl methyl ether (50 mL) were added. The aqueous phase was extracted with *tert*-butyl methyl ether (2 x 100 mL) and the combined organic layers were washed with HCl (2 M, 25 mL), sat. $CuSO_4$ solution (25 mL) and brine, and were then dried over $MgSO_4$. The solvent was removed under reduced pressure and the crude product was purified by flash chromatography (silica, hexanes/EtOAc 20:1 – 10:1) to give the corresponding homopropargylic alcohol (861 mg, 54%).

A solution of this homopropargylic alcohol (409 mg, 2.96 mmol) in THF (1 mL) was added to a stirred suspension of NaH (63 mg, 2.66 mmol) in THF (4 mL) at 0 °C. The mixture was stirred for 30 min at room temperature before MeI (0.20 mL, 3.25 mmol) was introduced. After stirring for another 2 h at room temperature, sat. NH_4Cl solution (3 mL), water (3 mL) and *tert*-butyl methyl ether (30 mL) were introduced and the layers separated. The aqueous phase was extracted with *tert*-butyl methyl ether (2 x 30 mL) and the combined organic layers were washed with brine and dried over $MgSO_4$. The solvent was removed under reduced pressure and the crude product was purified by flash chromatography (silica, hexanes/EtOAc 1:0 – 50:1) to give the title compound as a colorless oil (196 mg, 48%). ^{1}H NMR (400 MHz, $CDCl_3$) δ 3.22 (s, 3H), 2.35 (d, *J* = 2.7 Hz, 2H), 2.02 (t, *J* = 2.7 Hz, 1H), 1.84 – 1.74 (m, 2H), 1.63 – 1.39 (m, 7H), 1.30 – 1.16 (m, 1H). ^{13}C NMR (101 MHz, $CDCl_3$) δ 81.0, 74.6, 70.6, 48.8, 33.6, 27.5, 25.7, 21.9. IR (film) ṽ 3309, 2931, 2856, 2121, 1456, 1147, 1079 cm^{-1}. HRMS (EI$^+$) for $C_{10}H_{16}O$ $[M]^+$: calcd 152.1196, found 152.1198.

Via Sonogashira Coupling

(*E*)-(4-(1-Methoxycyclohexyl)but-1-en-3-yn-1-yl)trimethylsilane (8). *i*-Pr_2NEt (0.97 mL, 5.6 mmol), CuI

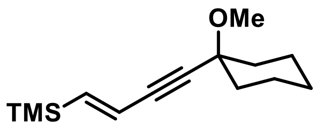

(106 mg, 0.56 mmol) and $Pd(PPh_3)_2Cl_2$ (196 mg, 0.28 mmol) were added to a stirred solution of (*E*)-(2-bromovinyl)trimethylsilane (500 mg, 2.79 mmol) and alkyne **S4** (500 mg, 3.63 mmol) in DMF (14 mL). The mixture was stirred for 1 h at room temperature before sat. NH_4Cl solution (15 mL) and *tert*-butyl methyl ether (30 mL) were introduced. The layers were separated and the aqueous phase was extracted with *tert*-butyl methyl ether (2 x 50 mL). The combined organic layers were washed with sat. NaCl solution (3 x 10 mL) and dried over $MgSO_4$. The solvent was removed under reduced pressure and the residue was purified by flash chromatography (silica, hexanes/EtOAc 1:0 – 100:1 – 50:1) to yield the title compound as a pale orange oil (288 mg, 44%). ^{1}H NMR (400 MHz, $CDCl_3$) δ 6.42 (d, *J* = 19.3 Hz, 1H), 6.00 (d, *J* = 19.3 Hz, 1H), 3.36 (s, 3H), 1.89 (m, 2H), 1.70 – 1.45 (m, 7H), 1.36 – 1.24 (m, 1H), 0.09 (s, 9H). ^{13}C NMR (101 MHz,

$CDCl_3$) δ 145.5, 123.3, 91.0, 86.5, 74.4, 50.9, 36.9, 25.6, 23.0, -1.5. IR (film) ṽ 2934, 1573, 1447, 1248, 1092 cm^{-1}. HRMS (ESI^+) for $C_{14}H_{24}OSi$ $[M+Na]^+$: calcd 259.1489, found 259.1491.

Ethyl (*E*)-5-(1-methoxycyclohexyl)pent-2-en-4-ynoate (S34). *i*-Pr_2NEt (0.92 mL, 5.3 mmol), CuI (101 mg, 0.53 mmol) and $Pd(PPh_3)_2Cl_2$ (185 mg, 0.26 mmol) were added to a stirred solution of ethyl (*E*)-3-bromoacrylate (500 mg, 2.64 mmol) and alkyne **S4** (434 mg, 3.14 mmol) in DMF (13 mL). The mixture was stirred for 1 h at room temperature before sat. NH_4Cl solution (15 mL) and *tert*-butyl methyl ether (30 mL) were introduced. The layers were separated and the aqueous phase was extracted with *tert*-butyl methyl ether (2 x 50 mL). The combined organic layers were washed with sat. NaCl solution (3 x 10 mL) and dried over $MgSO_4$. The solvent was removed under reduced pressure and the residue was purified by flash chromatography (silica, hexanes/EtOAc 1:0 – 40:1) to yield the title compound as a colorless oil (376 mg, 60%). ^1H NMR (400 MHz, $CDCl_3$) δ 6.81 (d, *J* = 15.9 Hz, 1H), 6.22 (d, *J* = 15.9 Hz, 1H), 4.21 (q, *J* = 7.1 Hz, 2H), 3.36 (s, 3H), 1.92 (m, 2H), 1.73 – 1.56 (m, 4H), 1.50 (m, 3H), 1.36 – 1.26 (m, 1H), 1.29 (t, *J* = 7.1 Hz, 3H). ^{13}C NMR (101 MHz, $CDCl_3$) δ 166.0, 130.6, 125.0, 100.1, 83.1, 74.5, 60.9, 51.1, 36.6, 25.5, 22.8, 14.4. IR (film) ṽ 2935, 2858, 2216, 1715, 1620, 1447, 1260, 1161 cm^{-1}. HRMS (ESI^+) for $C_{14}H_{20}O_3$ $[M+Na]^+$: calcd 259.1305, found 259.1305.

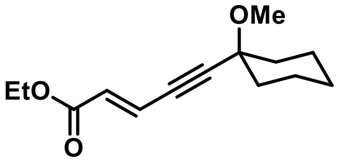

(4-(1-Methoxycyclohexyl)but-1-en-3-yn-2-yl)trimethylsilane (S35). *i*-Pr_2NEt (0.97 mL, 5.6 mmol), CuI (106 mg, 0.56 mmol) and $Pd(PPh_3)_2Cl_2$ (196 mg, 0.28 mmol) were added to a stirred solution of (1-bromovinyl)trimethylsilane (500 mg, 2.79 mmol) and alkyne **S4** (500 mg, 3.63 mmol) in DMF (14 mL). The mixture was stirred for 1 h at room temperature before sat. NH_4Cl solution (15 mL) and *tert*-butyl methyl ether (30 mL) were introduced. The layers were separated and the aqueous phase was extracted with *tert*-butyl methyl ether (2 x 50 mL). The combined organic layers were washed with sat. NaCl solution (3 x 10 mL) and dried over $MgSO_4$. The solvent was removed under reduced pressure and the residue was purified by flash chromatography (silica, hexanes/EtOAc 1:0 – 100:1 – 50:1) and HPLC to yield the title compound as a colorless oil (120 mg, 18%). ^1H NMR (400 MHz, $CDCl_3$) δ 6.07 (d, *J* = 3.4 Hz, 1H), 5.68 (d, *J* = 3.5 Hz, 1H), 3.38 (s, 3H), 1.97 – 1.86 (m, 2H), 1.73 – 1.47 (m, 7H), 1.29 (m, 1H), 0.17 (s, 9H). ^{13}C NMR (101 MHz, $CDCl_3$) δ 134.4, 133.8, 94.6, 87.5, 74.7, 50.8, 37.1, 25.7, 23.1, -2.0. IR (film) ṽ 2934, 2857, 1446, 1248, 1091 cm^{-1}. HRMS (EI^+) for $C_{14}H_{24}OSi$ $[M]^+$: calcd 236.1591, found 236.1586.

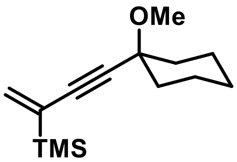

tert-Butyl 3-(3-(diethoxymethyl)but-3-en-1-yn-1-yl)-3-methoxyazetidine-1-carboxylate (S36). Prepared analogously from *tert*-butyl 3-ethynyl-3-methoxyazetidine-1-carboxylate (211 mg, 1.0 mmol) (**S17**) and 2-bromopropenal diethyl acetal (250 mg, 1.3 mmol), colorless oil (69.2 mg, 22%). ^1H NMR (400 MHz, C_6D_6) δ 5.74 (t, *J* = 1.6 Hz, 1H), 5.52 (dd, *J* = 2.0, 1.2 Hz, 1H), 4.86 (s, 1H), 4.17 (d, *J* = 8.7 Hz, 2H), 4.09 (d, *J* = 9.1 Hz, 2H), 3.51 (dq, *J* = 9.5, 7.1 Hz, 2H), 3.31 (dq, *J* = 9.5, 7.1 Hz, 2H), 3.11 (s, 3H), 1.40 (s, 9H), 1.07 (t, *J* = 7.0 Hz, 6H). ^{13}C NMR (101 MHz, C_6D_6) δ 156.2, 129.8, 124.5, 100.3, 88.3, 85.9, 79.3, 68.7, 62.2 (br), 61.1, 52.3, 28.4, 15.4. IR (film) ṽ 2976, 2882, 1705, 1392, 1167, 1098 cm^{-1}. HRMS (ESI^+) for $C_{18}H_{29}NO_5Na$ $[M+Na]^+$: calcd 362.1938, found 362.1937.

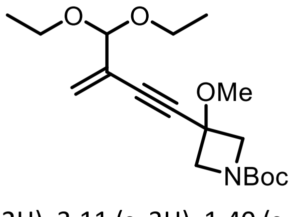

***tert*-Butyl 3-(5-((*tert*-butyldimethylsilyl)oxy)-3-methylenepent-1-yn-1-yl)-3-methoxyazetidine-1-carboxylate (S37).** A solution of *tert*-butyl 3-ethynyl-3-methoxyazetidine-1-carboxylate (**S17**) (220 mg, 1.1 mmol) and ((3-bromobut-3-en-1-yl)oxy)(*tert*-butyl)dimethylsilane (**S8**) (325 mg, 1.2 mmol) in THF (2.0 mmol) was added to a solution of CuI (4.9 mg, 0.03 mmol) and $Pd(PPh_3)_4$ (3.6 mg, 0.003 mmol) in NEt_3 (0.5 mL). The mixture was stirred at ambient temperature until TLC indicated full conversion. The mixture was filtered through a plug of Florisil, eluting with *tert*-butyl methyl ether. The combined filtrates were successively washed with HCl (0.1 M, 15 mL) and $NaHCO_3$ (15 mL), dried over $MgSO_4$, and evaporated. Purification of the crude product by flash chromatography (silica, hexanes/ *tert*-butyl methyl ether, 85:15) gave the title compound as a colorless oil (76.7 mg, 19 %). ^{1}H NMR (400 MHz, $CDCl_3$) δ 5.46 – 5.41 (m, 1H), 5.35 (dt, *J* = 2.1, 1.3 Hz, 1H), 4.07 (dd, *J* = 8.9, 0.9 Hz, 2H), 4.01 (dd, *J* = 8.8, 1.0 Hz, 2H), 3.77 (t, *J* = 6.7 Hz, 2H), 3.34 (s, 3H), 2.37 (td, *J* = 6.7, 0.7 Hz, 2H), 1.44 (s, 9H), 0.89 (s, 9H), 0.05 (s, 6H). ^{13}C NMR (101 MHz, $CDCl_3$) δ 156.3, 127.4, 124.5, 87.9, 86.0, 80.0, 68.3, 61.6, 52.7, 40.6, 28.5, 26.1, 18.5, -5.1. IR (film) ṽ 2930, 2857, 1708, 1391, 1254, 1167, 1097 cm^{-1}.

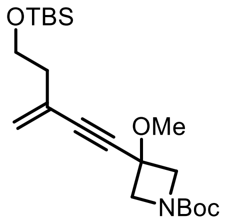

***tert*-Butyl 3-methoxy-3-(3-(trifluoromethyl)but-3-en-1-yn-1-yl)azetidine-1-carboxylate (S38).** Prepared analogously from alkyne **S17** (211 mg, 1.0 mmol) and 2-bromo-3,3,3-trifluoro-1-propene (227.0 mg, 1.3 mmol); pale yellow oil (217 mg, 72 %). ^{1}H NMR (400 MHz, $CDCl_3$) δ 6.14 (s, 1H), 5.94 (s, 1H), 4.12 (d, *J* = 10.0 Hz, 2H), 4.04 (d, *J* = 10.1 Hz, 2H), 3.35 (s, 3H), 1.45 (s, 9H).^{13}C NMR (101 MHz, $CDCl_3$) δ 156.2, 128.49 (q, *J* = 4.4 Hz), 122.12 (q, *J* = 273 Hz). 119.8, 90.6, 80.3, 79.9, 68.1, 61.2, 52.9, 28.5. ^{19}F NMR (282 MHz, $CDCl_3$) δ -68.1. IR (film) ṽ 2978, 2885, 2832, 1704, 1392, 1243, 1135 cm^{-1}. HRMS (ESI$^+$) for $C_{14}H_{18}NO_2F_3$ $[M+H]^+$: calcd 328.1131, found 328.1132.

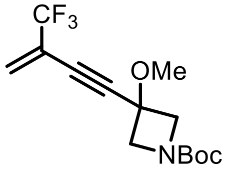

***tert*-Butyl 4-((1-(tert-butoxycarbonyl)-3-methoxyazetidin-3-yl)ethynyl)-3,6-dihydropyridine-1(2*H*)-carboxylate (S39)**. Prepared analogously from alkyne **S17** (217 mg, 1.0 mmol) and N-Boc-4-trifluoromethanesulfonyloxy-3,6-dihydro-2*H*-pyridine (398.0 mg, 1.2 mmol); colorless oil (400.0 mg, 99%). ^{1}H NMR (400 MHz, $CDCl_3$) δ 6.1 (s, 1H), 4.0 (dd, *J* = 13.3, 8.8 Hz, 4H), 4.0 (q, *J* = 3.1 Hz, 2H), 3.5 (t, *J* = 5.7 Hz, 2H), 3.3 (s, 3H), 2.2 (qd, *J* = 6.0, 3.5 Hz, 2H), 1.5 (s, 9H), 1.4 (s, 9H). ^{13}C NMR (101 MHz, $CDCl_3$) δ 156.3, 154.8, 132.1 (br), 118.6 (br), 87.6, 85.5, 80.1, 80.1, 68.3, 61.5 (br), 52.6, 43.9 (br), 39.3 (br), 29.2, 28.6, 28.5. IR (film) ṽ 2975, 2933, 2882, 2196, 1695, 1391, 1365, 1238, 1151 cm^{-1}. HRMS (ESI$^+$) for $C_{21}H_{32}N_2O_5Na$ $[M+Na]^+$: calcd 415.2203, found 415.2206

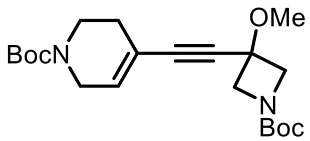

***tert*-Butyl 3-methoxy-3-((5-oxo-2,5-dihydrofuran-3-yl)ethynyl)azetidine-1-carboxylate (S40)**. Prepared analogously from alkyne **S17** (209 mg, 0.099 mmol) and 4-bromofuran-2(5*H*)-one (209.5 mg, 1.3 mmol); colorless oil (255 mg, 80 %). ^{1}H NMR (400 MHz $CDCl_3$) δ 6.3 (t, *J* = 2.0 Hz, 1H), 4.8 (d, *J* = 2.0 Hz, 2H), 4.1 (dd, *J* = 8.9, 1.0 Hz, 2H), 4.1 (dd, *J* = 8.9, 1.0 Hz, 2H), 3.4 (s, 3H), 1.4 (s, 9H). ^{13}C NMR (101 MHz, $CDCl_3$) δ 172.7, 156.1, 145.7, 124.3, 102.2, 80.5, 72.9, 68.3, 60.9 (br), 53.2, 28.5. IR (film) ṽ 2976, 2883, 2219, 1777, 1749, 1699, 1392, 1156, 1083 cm^{-1}. HRMS (ESI$^+$) for $C_{15}H_{19}NO_5Na$ $[M+Na]^+$: calcd 316.1155, found 316.1154.

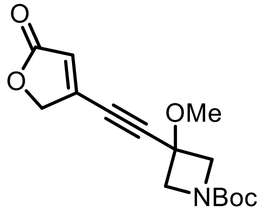

tert-butyl 3-methoxy-3-(3-((trimethylsilyl)methyl)but-3-en-1-yn-1-yl)azetidine-1-carboxylate (S41).

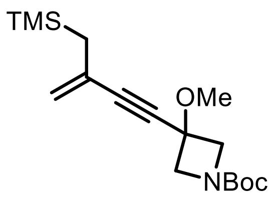

Prepared analogously from alkyne **S17** (209 mg, 1.0 mmol) and 2-bromo-allyl-trimethylsilane (272 mg, 1.3 mmol); colorless oil (255 mg, 80 %). ^{1}H NMR (400 MHz, $CDCl_3$) δ 5.2 (d, *J* = 1.9 Hz, 1H), 5.1 (q, *J* = 1.2 Hz, 1H), 4.1 (dd, *J* = 8.9, 1.0 Hz, 2H), 4.0 (dd, *J* = 8.9, 0.9 Hz, 2H), 3.3 (s, 3H), 1.7 (d, *J* = 1.1 Hz, 2H), 1.4 (s, 9H), 0.1 (s, 9H). ^{13}C NMR (101 MHz, $CDCl_3$) δ 156.3, 127.9, 120.4, 89.8, 85.1, 80.0, 68.3, 61.5 (br), 52.7, 28.5, 28.2, -1.5. IR (film) ṽ 2952, 2885, 1705, 1391, 1163, 1094 cm^{-1}. HRMS (ESI$^+$) for $C_{17}H_{29}NO_3SiNa$ $[M+Na]^+$: calcd 346.1809, found 346.1811.

Acetal Series

2-Cyclohexyl-2-(3-methylbut-3-en-1-yn-1-yl)-1,3-dioxane1-cyclohexyl (27).

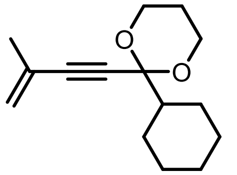

Trimethyl orthoformate (0.11 mL, 1.0 mmol) and *p*-TsOH·H_2O (6.0 mg, 0.08 mmol) were added to a solution of 4-methylpent-4-en-2- yn-1-one (**S16**) (150 mg, 0.85 mmol) in 1,3-propanediol (4.0 mL). The mixture was stirred for 24 h before the reaction was quenched with sat. $NaHCO_3$ (15 mL) and *tert*-butyl methyl ether (15 mL). The aqueous phase was extracted with *tert*-butyl methyl ether (2 x 15 mL), and the combined organic layers were washed with brine (10 mL) and dried over $MgSO_4$. The solvent was removed under reduced pressure to yield the title compound as pale yellow oil (169 mg, 85 %). ^{1}H NMR (400 MHz, C_6D_6) δ 5.37 (dq, *J* = 2.2, 1.0 Hz, 1H), 5.05 (p, *J* = 1.6 Hz, 1H), 4.19 (dddd, *J* = 12.8, 10.5, 2.6, 1.5 Hz, 2H), 3.68 (ddt, *J* = 10.5, 5.2, 1.4 Hz, 2H), 2.45 – 2.34 (m, 2H), 2.05 (tt, *J* = 12.0, 3.3 Hz, 1H), 1.87 – 1.72 (m, 6H), 1.66 – 1.51 (m, 3H), 1.31 – 1.08 (m, 3H), 0.73 (dtt, *J* = 13.2, 2.7, 1.4 Hz, 1H). ^{13}C NMR (101 MHz, C_6D_6) δ 126.6, 122.5, 99.4, 88.8, 85.3, 62.4, 49.0, 27.3, 27.0, 26.6, 25.9, 23.5. IR (film) ṽ 2924, 2853, 1614, 1452, 1372, 1114, 1101 cm^{-1}. HRMS (CI$^+$) for $C_{15}H_{23}O_2$ $[M+H]^+$: calcd 225.1692, found 225.1693.

2-Methoxy-2-(3-methylbut-3-en-1-yn-1-yl)tetrahydrofuran (28a).

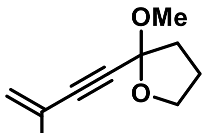

n-BuLi (1.6 M in hexanes, 4.25 mL, 6.81 mmol) was added to a stirred solution of 2-methyl-1-buten-3-yne (500 mg, 7.56 mmol) in THF (30 mL) at –78 °C under argon and was stirred for 30 min at that temperature. BF_3·OEt_2 (1 M in Et_2O, 8.3 mL, 8.3 mmol) was added and the mixture stirred for 30 min at –78 °C before γ-butyrolactone (0.70 mL, 9.1 mmol) was slowly introduced. The mixture was allowed to warm to room temperature and stirring was continued for 18 h before sat. NH_4Cl solution (6 mL) and water (6 mL) were added at 0 °C. The biphasic mixture was extracted with EtOAc (3 x 75 mL) and the combined organic layers were washed with brine and dried over $MgSO_4$. The solvent was removed under reduced pressure and the crude product was purified by flash chromatography (silica, hexanes/EtOAc 2:1 – 3:2 – 1:1) to yield a mixture of the corresponding γ-hydroxyketone, its lactol and its butyric acid ester (692 mg, ~60%).

This mixture (200 mg, ~1.3 mmol) was dissolved in MeOH (5 mL) and *p*-TsOH·H_2O (25 mg, 0.13 mmol) was added at room temperature. The mixture was stirred for 18 h before sat. $NaHCO_3$ solution (3 mL) was introduced. The mixture was extracted with *tert*-butyl methyl ether (3 x 20 mL) and the combined organic layers were washed with brine and dried over $MgSO_4$. The solvent was removed under reduced pressure and the crude product was purified by flash chromatography (silica, hexanes/EtOAc 20:1) to yield the title product as a colorless oil (126 mg, 58%). ^{1}H NMR (400 MHz, C_6D_6) δ 5.34 (dq, *J* = 2.1, 1.1 Hz, 1H), 5.00 (p, *J* = 1.6 Hz, 1H), 3.76 – 3.65 (m, 2H), 3.49 (s, 3H), 2.30 – 2.22 (m, 1H), 2.11 (ddd, *J* = 12.7, 9.6, 8.7 Hz, 1H),

1.81 – 1.72 (m, 1H), 1.71 (dd, *J* = 1.6, 1.1 Hz, 3H), 1.47 (m, 1H). ^{13}C NMR (101 MHz, C_6D_6) δ 126.6, 122.8, 102.7, 86.4, 85.5, 67.9, 50.8, 40.6, 24.5, 23.3. IR (film) ṽ 2956, 2890, 2224, 1317, 1133, 1038 cm^{-1}. HRMS (ESI$^+$) for $C_{10}H_{14}O_2$ [M+Na]$^+$: calcd 189.0886, found 189.0886.

2-Isopropoxy-2-(3-methylbut-3-en-1-yn-1-yl)tetrahydrofuran (28b). Prepared analogously using *i*PrOH

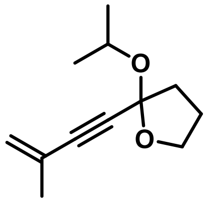

instead of MeOH (101 mg, 34%). ^{1}H NMR (400 MHz, C_6D_6) δ 5.34 (dq, *J* = 2.1, 1.0 Hz, 1H), 5.01 (p, *J* = 1.6 Hz, 1H), 4.55 (hept, *J* = 6.2 Hz, 1H), 3.81 – 3.70 (m, 2H), 2.31 – 2.21 (m, 1H), 2.10 (ddd, *J* = 12.5, 9.5, 8.6 Hz, 1H), 1.84 – 1.73 (m, 1H), 1.72 (dd, *J* = 1.6, 1.0 Hz, 3H), 1.49 (ddddd, *J* = 11.8, 9.4, 7.7, 5.9, 4.1 Hz, 1H), 1.31 (d, *J* = 6.2 Hz, 3H), 1.25 (d, *J* = 6.2 Hz, 3H). ^{13}C NMR (101 MHz, C_6D_6) δ 126.7, 122.5, 102.5, 87.9, 84.6, 67.8, 67.7, 41.3, 24.7, 24.5, 24.0, 23.3. IR (film) ṽ 2971, 2890, 1439, 1316, 1118, 1003 cm^{-1}. HRMS (ESI$^+$) for $C_{12}H_{18}O_2$ [M+Na]$^+$: calcd 217.1199, found 217.1199.

(1,1-Dimethoxy-4-methylpent-4-en-2-yn-1-yl)cyclohexane (S42). Trimethyl orthoformate (0.47 mL,

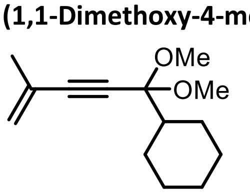

4.3 mmol) was added to a solution of 1-cyclohexyl- 4-methylpent-4-en-2-yn-1-one (**S16**) (150 mg, 0.85 mmol) in MeOH (3.0 mL) at 0 °C., followed by *p*-TsOH·H_2O (6.0 mg, 0.08 mmol). Once GC/MS indicated full conversion, sat. $NaHCO_3$ (15 mL) and *tert*-butyl methyl ether (15 mL) were introduced, the aqueous phase was extracted with *tert*-butyl methyl ether (2 x 15 mL), and the combined organic phases were washed with brine (10 mL) and dried over $MgSO_4$. The solvent was removed under reduced pressure to yield the title compound as pale yellow oil (189 mg, quant). ^{1}H NMR (400 MHz, C_6D_6) δ 5.41 (dq, *J* = 2.2, 1.1 Hz, 1H), 5.08 (p, *J* = 1.6 Hz, 1H), 3.33 (s, 6H), 2.22 – 2.12 (m, 2H), 1.99 (tt, *J* = 11.8, 3.4 Hz, 1H), 1.89 – 1.76 (m, 5H), 1.72 – 1.53 (m, 3H), 1.35 – 1.13 (m, 3H). ^{13}C NMR (101 MHz, C_6D_6) δ 126.3, 122.4, 102.7, 87.3, 85.1, 49.7, 43.8, 27.7, 26.6, 26.3, 23.1. IR (film) ṽ 2929, 2853, 2227, 1615, 1451, 1312, 1110, 1047 cm^{-1}. HRMS (CI$^+$) for $C_{14}H_{23}O_2$ [M+H]$^+$: calcd 223:1693, found 223.1690.

2-Cyclohexyl-2-(3-methylbut-3-en-1-yn-1-yl)-1,3-dioxolane (S43). Trimethyl orthoformate (0.11 mL,

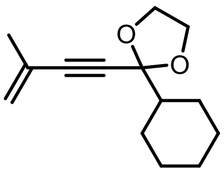

1.0 mmol) and *p*-TsOH·H_2O (6.0 mg, 0.08 mmol) were added to a solution of 1-cyclohexyl- 4-methylpent-4-en-2-yn-1-one (**S16**) (150 mg, 0.85 mmol) in ethylene glycol (4.0 mL). The mixture was stirred for 24 h before sat. $NaHCO_3$ (15 mL) and *tert*-butyl methyl ether (15 mL) were added. The layers were separated, the aqueous phase was extracted with *tert*-butyl methyl ether (2 x 15 mL), and the combined organic layers were washed with brine (10 mL) and dried over $MgSO_4$. The solvent was removed under reduced pressure to yield the title compound as pale yellow oil (167 mg, 89 %). ^{1}H NMR (400 MHz, C_6D_6) δ 5.33 (dd, *J* = 2.2, 1.1 Hz, 1H), 5.00 (p, *J* = 1.6 Hz, 1H), 3.85 – 3.71 (m, 2H), 3.57 – 3.44 (m, 2H), 2.34 – 2.17 (m, 2H), 1.97 (tt, *J* = 12.0, 3.4 Hz, 1H), 1.82 – 1.62 (m, 5H), 1.61 – 1.47 (m, 3H), 1.27 – 1.04 (m, 3H). ^{13}C NMR (101 MHz, C_6D_6) δ 126.7, 122.6, 106.5, 87.0, 85.6, 64.7, 47.0, 27.8, 26.7, 26.3, 23.4. ṽ 2929, 2854, 2197; 1669, 1614, 1285, 11192, 1085, 1042 cm^{-1}. HRMS (CI$^+$) for $C_{14}H_{21}O_2$ [M+H]$^+$: calcd 221.1536, found 221.1536.

2-((4-(Cyclohex-1-en-1-yl)-2-methylbut-3-yn-2-yl)oxy)tetrahydro-2*H*-pyran (S44). 3,4-Dihydro-2*H*-pyran (0.14 mL, 1.6 mmol) and toluenesulfonic acid monohydrate (11 mg, 0.06 mmol) were added to a solution of 4-(cyclohex-1-en-1-yl)-2-methylbut-3-yn-2-ol (200 mg, 1.2 mmol) (**S10**) in CH_2Cl_2 (2.5 mL) at 0°C and the resulting mixture was stirred at this temperature overnight. sat. $NaHCO_3$ (15 mL) and *tert*-butyl methyl ether (15 mL) were introduced, the layers were separated, the aqueous phase was extracted with *tert*-butyl methyl ether (2 x 15 mL), and the combined organic layers were washed with brine (10 mL) and dried over $MgSO_4$. The solvent was removed under reduced pressure and the crude material purified by flash chromatography (silica, hexanes/ *tert*-butyl methyl ether, 98:2) to give the title compound as a colorless oil (90.2 mg, 30%). ^1H NMR (400 MHz, C_6D_6) δ 6.10 (tt, *J* = 3.9, 1.8 Hz, 1H), 5.52 (t, *J* = 3.5 Hz, 1H), 3.95 (ddd, *J* = 11.5, 8.3, 3.2 Hz, 1H), 3.46 (dddd, *J* = 11.3, 5.2, 3.9, 1.4 Hz, 1H), 2.15 – 2.07 (m, 2H), 1.87 – 1.69 (m, 8H), 1.64 (s, 3H), 1.40 – 1.25 (m, 7H). ^{13}C NMR (101 MHz, C_6D_6) δ 134.6, 121.0, 95.8, 89.8, 86.7, 71.8, 62.2, 32.3, 31.4, 30.6, 29.8, 26.0, 25.7, 22.6, 21.8, 20.1. . IR (film) ṽ 2934, 2860, 2211, 1679, 1438, 1225, 1123, 1075 cm^{-1}. HRMS (CI^+) for $C_{16}H_{24}O_2Na$ $[M+Na]^+$: calcd 271.1668, found 271.1667.

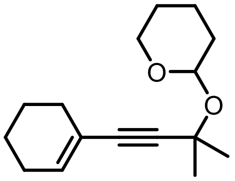

Orthoester

5,5,5-Trimethoxy-2-methylpent-1-en-3-yne (29). *n*BuLi (1.79 mL, 2.86 mmol, 1.6 M in hexanes) was slowly added to a solution of 2-methyl-1-buten-3-yne (0.27 mL, 2.86 mmol) in THF (120 mL) at −78°C, and the resulting mixture was stirred for 30 min. In parallel, $BF_3{\cdot}Et_2O$ (0.74 mL, 2.86 mmol) was added dropwise to a solution of tetramethyl orthocarbonate (0.40 mL, 3.01 mmol) in Et_2O (20 mL) at −78°C. After the addition, the temperature was raised to 0°C for 1h and the mixture finally cooled again to -78°C. The solution of the organolithium reagent was then added via cannula to the solution of the oxonium salt. After the addition was complete, the mixture was stirred for 1h at -78°C and for another 1h at 25°C. Finally the mixture was cooled to -20°C before it was poured into a solution of Na_2CO_3 (70 mL) at 0°C. The aqueous phase was extracted with CH_2Cl_2 (3 x 50 mL), the combined organic layers were dried over Na_2SO_4, filtrated and concentrated to give the desired product as a colorless oil, which was used without further purification (377 mg, 77%). ^1H NMR (400 MHz, CD_2Cl_2) δ 5.44 – 5.38 (m, 1H), 5.38 – 5.32 (m, 1H), 3.33 (s, 9H), 1.95 – 1.89 (m, 3H); ^{13}C NMR (101 MHz, CD_2Cl_2) δ 125.8, 124.2, 111.0, 85.9, 81.5, 50.9, 23.3; FT-IR (neat): 2960, 2837, 1438, 1259, 1150, 1099, 1042, 1006, 793 cm^{-}1; HRMS (ESI): *m/z* calcd for $C_9H_{14}O_3Na^+$: 193.0835. $[M+Na]^+$; found: 193.0835

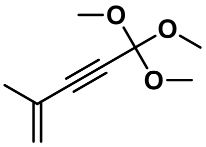

Silylether Derivatives

Trimethyl((4-(3-methylbut-3-en-1-yn-1-yl)tetrahydro-2*H*-pyran-4-yl)oxy)silane (39). 1*H*-Imidazol (70 mg, 1.0 mmol) was added in one portion to a solution of 4-(3-methylbut-3-en-1-yn-1-yl)tetrahydro-2*H*-pyran-4-ol (**S14**) (150 mg, 0.90 mmol) in CH_2Cl_2 (3.0 mL) and the resulting mixture was stirred for 15 min. TMSCl (0.12 mL, 0.96 mmol) was added and stirring continued for 2 h. The reaction was quenched with water (20 mL) and the mixture diluted with 20 mL of *tert*-butyl methyl ether (20 mL). The layers were separated and the aqueous phase was extracted with *tert*-butyl methyl ether (2 x 20 mL). The combined organic layers were washed with brine and dried over $MgSO_4$, the solvent was removed under reduced pressure, and the crude product was purified by flash chromatography (silica, hexanes/Et_2O 98:2) to give the title compound as a colorless

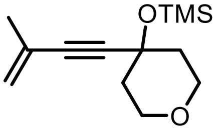

oil (211 mg, 92 %). ^{1}H NMR (400 MHz, $CDCl_3$) δ 5.28 (dq, *J* = 2.0, 1.0 Hz, 1H), 5.24 (p, *J* = 1.6 Hz, 1H), 3.89 – 3.79 (m, 2H), 3.64 (ddd, *J* = 11.6, 8.5, 3.1 Hz, 2H), 1.94 – 1.84 (m, 5H), 1.79 (ddd, *J* = 13.7, 8.7, 3.9 Hz, 2H), 0.20 (s, 9H). ^{13}C NMR (101 MHz, $CDCl_3$) δ 126.5, 121.9, 91.1, 87.3, 67.3, 64.9, 41.5, 23.4, 2.0. IR (film) ṽ 2957, 2929, 2857, 1615, 1249, 1110, 1005 cm^{-1}. HRMS (ESI^+) for $C_{13}H_{22}O_2SiNa$ $[M+Na]^+$: calcd 261.1281, found 261.1282.

4-(3-Methylbut-3-en-1-yn-1-yl)-1-tosyl-4-((trimethylsilyl)oxy)piperidine (S45). Prepared analogously

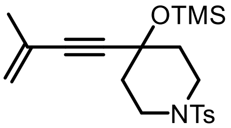

from from 4-(3-methylbut-3-en-1-yn-1-yl)-1-tosylpiperidin-4-ol (**S13**) (200 mg, 0.63 mmol); colorless oil (245 mg, 99 %). ^{1}H NMR (400 MHz, $CDCl_3$) δ 7.69 – 7.61 (m, 2H), 7.36 – 7.28 (m, 2H), 5.16 (p, *J* = 1.6 Hz, 1H), 5.09 (dq, *J* = 2.0, 1.0 Hz, 1H), 3.23 (ddd, *J* = 11.3, 6.9, 4.1 Hz, 2H), 2.96 (ddd, *J* = 11.8, 8.2, 3.7 Hz, 2H), 2.42 (s, 3H), 1.95 – 1.78 (m, 4H), 1.74 (dd, *J* = 1.6, 1.0 Hz, 3H), 0.12 (s, 9H). ^{13}C NMR (101 MHz, $CDCl_3$) δ 143.6, 133.2, 129.6, 127.9, 126.1, 121.9, 90.1, 88.0, 67.1, 43.2, 39.6, 23.1, 21.6, 1.8. IR (film) ṽ 2959, 2933, 2860, 1605, 1342, 1164, 1045 cm^{-1}. HRMS (ESI^+) for $C_{20}H_{30}NO_3SSi$ $[M+H]^+$: calcd 392.1710, found 392.1711.

Trimethyl((3-(3-methylbut-3-en-1-yn-1-yl)tetrahydrofuran-3-yl)oxy)silane (S46). Prepared analogously

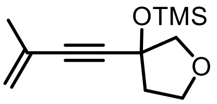

from from 3-(3-methylbut-3-en-1-yn-1-yl)tetrahydrofuran-3-ol (**S15**) (200 mg, 1.31 mmol), colorless oil (200 mg, 68 %). ^{1}H NMR (400 MHz, $CDCl_3$) δ 5.28 (dq, *J* = 2.0, 1.0 Hz, 1H), 5.24 (p, *J* = 1.6 Hz, 1H), 4.06 – 3.92 (m, 2H), 3.84 (s, 2H), 2.31 – 2.15 (m, 2H), 1.89 (dd, *J* = 1.6, 1.0 Hz, 3H), 0.21 (s, 9H). ^{13}C NMR (101 MHz, $CDCl_3$) δ 126.3, 122.3, 89.0, 86.8, 81.1, 74.6, 67.9, 43.9, 23.3, 1.7. . IR (film) ṽ 2955, 2871, 1615, 1436, 1249, 1124, 1037 cm^{-1}. HRMS (ESI^+) for $C_{12}H_{20}O_2SiNa$ $[M+Na]^+$: calcd 247.1125, found 247.1125.

***tert*-Butyl 3-(3-methylbut-3-en-1-yn-1-yl)-3-((trimethylsilyl)oxy)pyrrolidine-1-carboxylate (S47).**

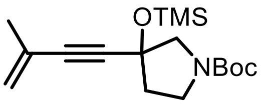

n-BuLi (1.6 M in hexanes, 0.88 mL, 1.4 mmol) was slowly added to a solution of 2-methylbut-1-en-3-yne (153 mg, 1.6 mmol) in THF (6.0 mL) at –78 °C. The mixture was stirred for 15 min at –78 °C before a solution of N-Boc-3-pyrrolidinone (190 mg, 1.0 mmol) in THF (2.0 mL) was slowly added. The mixture was stirred at –78 °C for 15 min and then warmed to –20 °C over 60 min. sat. NH_4Cl solution (40 mL) and EtOAc (40 mL) were introduced, the layers were separated, and the aqueous phase was extracted with EtOAc (2 x 40 mL). The combined organic layers were dried over $MgSO_4$ and the solvent was removed under reduced pressure. The crude product was purified by flash chromatography (silica, hexanes/EtOAc, 4:1) to yield the corresponding *tert*-alcohol in slightly impure form (200 mg, ~70 %).

1*H*-Imidazol (63 mg, 0.93 mmol) was added to a solution of this compound (200 mg, ~0.72 mmol) in CH_2Cl_2 (3.0 mL). After stirring for 15 min, TMSCl (0.11 mL, 0.86 mmol) was introduced and the resulting mixture was stirred for 2 h. The reaction was quenched with water (20 mL) and *tert*-butyl methyl ether (20 mL), the layers were separated, and the aqueous phase was extracted with *tert*-butyl methyl ether (2 x 20 mL). The combined organic layers were washed with brine and dried over $MgSO_4$. The solvent was removed under reduced pressure and the crude material was purified by flash chromatography (silica, hexanes/ *tert*-butyl methyl ether, 98:2) to give the title compound as a colorless oil (150 mg, 65 %). ^{1}H NMR (400 MHz, $CDCl_3$) δ 5.29 – 5.26 (m, 1H), 5.26 – 5.22 (m, 1H), 3.57 – 3.39 (m, 4H), 2.16 – 2.06 (m, 2H), 1.88 (q, *J* = 1.4 Hz, 3H), 1.46 (s, 9H), 0.20 (d, *J* = 1.8 Hz, 9H). ^{13}C NMR (101 MHz, $CDCl_3$) δ 154.7, 126.3, 126.2, 122.4, 122.3, 88.9, 88.8, 86.6, 79.5, 79.4, 73.3, 72.5, 60.5, 59.9, 44.6, 44.2, 42.0, 41.2, 28.6, 23.3, 1.6, 1.6.

IR (film) ṽ 2974, 2894, 1699, 1396, 1250, 1132, 1033 cm^{-1}. HRMS (ESI^+) for $C_{17}H_{29}NO_3SiNa$ $[M+Na]^+$: calcd 346.1809, found 346.1807.

Propargyl Amine Derivatives

***tert*-Butyl (2,5-dimethylhex-5-en-3-yn-2-yl)(methyl)carbamate (S48).** A solution of carbamate **S19** (160 mg, 0.72 mmol) in DMF (1 mL) was added to a stirred suspension of NaH (26 mg, 1.1 mmol) in DMF (3 mL) at room temperature. The mixture was stirred for 30 min before MeI (0.10 mL, 1.6 mmol) was introduced. After stirring for 1 h at room temperature, sat. NH_4Cl solution (3 mL), water (3 mL) and *tert*-butyl methyl ether (15 mL) were added and the layers separated. The aqueous phase was extracted with *tert*-butyl methyl ether (2 x 15 mL) and the combined organic layers were washed with brine (3 x 10 mL) and dried over $MgSO_4$. The solvent was removed under reduced pressure and the crude product was purified by flash chromatography (silica, hexanes/EtOAc 40:1 – 20:1) to give the title compound as a colorless oil (148 mg, 87%). ^1H NMR (400 MHz, $CDCl_3$) δ 5.21 (m, 1H), 5.17 (m, 1H), 2.99 (s, 3H), 1.87 (s, 3H), 1.66 (s, 6H), 1.47 (s, 9H). ^{13}C NMR (101 MHz, $CDCl_3$) δ 155.7, 126.9, 121.1, 92.9, 83.8, 80.0, 53.9, 32.8, 29.0, 28.7, 23.7. IR (film) ṽ 2976, 2933, 1691, 1350, 1119 cm^{-1}. HRMS (ESI^+) for $C_{14}H_{23}NO_2$ $[M+Na]^+$: calcd 260.1621, found 260.1620.

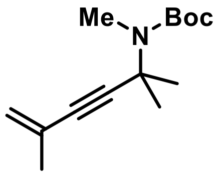

N-(2,5-Dimethylhex-5-en-3-yn-2-yl)-N,4-dimethylbenzenesulfonamide (46). Prepared analogously from enyne **S20** (300 mg, 1.08 mmol) as a colorless oil (270 mg, 86%). ^1H NMR (400 MHz, $CDCl_3$) δ 7.74 – 7.70 (m, 2H), 7.29 – 7.23 (m, 2H), 5.13 – 5.11 (m, 1H), 5.03 (dq, *J* = 2.0, 1.0 Hz, 1H), 3.07 (s, 3H), 2.40 (s, 3H), 1.70 (dd, *J* = 1.6, 1.0 Hz, 3H), 1.68 (s, 6H). ^{13}C NMR (101 MHz, $CDCl_3$) δ 143.0, 138.3, 129.5, 127.6, 126.3, 121.8, 89.8, 85.1, 56.3, 34.4, 30.4, 23.3, 21.6. IR (film) ṽ 2988, 2925, 1456, 1339, 1159, 1088 cm^{-1}. HRMS (ESI^+) for $C_{16}H_{21}NO_2S$ $[M+Na]^+$: calcd 314.1185, found 314.1186.

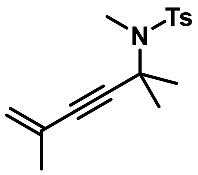

***tert*-Butyl N-(2,5-dimethylhex-5-en-3-yn-2-yl)-N-tosylglycinate (S49).** Sodium hydride (26.0 mg, 1.1 mmol) was added to a solution of the enyne **S20** (200 mg, 0.72 mmol) in DMF (1.0 mL) and the resulting mixture was stirred at ambient temperature for 15 min. *tert*-Butyl bromoacetate (0.16 mL, 1.1 mmol) was introduced and the solution was stirred for 4 h. The reaction was quenched with sat. NH_4Cl solution (5 mL) and the aqueous phase was extracted with EtOAc (3 x 20 mL). The combined organic layers were washed with brine (2 x 10 mL), dried over $MgSO_4$ and evaporated. The crude product was purified by flash chromatography (silica, hexanes/EtOAc, 80:20) to give the title compound as a white solid (230.0 mg, 82 %).^1H NMR (400 MHz, $CDCl_3$) δ 7.9 – 7.9 (m, 2H), 7.3 – 7.2 (m, 2H), 5.2 – 5.1 (m, 2H), 4.2 (s, 2H), 2.4 (s, 3H), 1.8 (dd, *J* = 1.6, 1.0 Hz, 3H), 1.7 (s, 6H), 1.4 (s, 9H). ^{13}C NMR (101 MHz, $CDCl_3$) δ 169.4, 143.1, 140.0, 129.5, 127.8, 126.2, 122.3, 90.3, 85.4, 81.8, 57.6, 49.4, 30.1, 28.1, 23.3, 21.6. . IR (film) ṽ 2979, 2945, 2258, 1749, 1455, 1224, 1146, 1090 cm^{-1}. HRMS (ESI^+) for $C_{21}H_{29}NO_4SiNa$ $[M+Na]^+$: calcd 414.1710, found 414.1710.

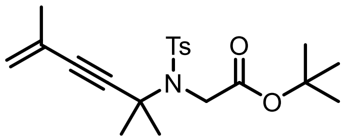

N-(2,5-Dimethylhex-5-en-3-yn-2-yl)-N-(methoxymethyl)-4-methylbenzenesulfonamide (S50). A solution

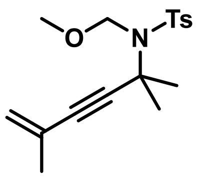

of enyne **S20** (300 mg, 1.08 mmol) in DMF (1 mL) was slowly added to a stirred suspension of NaH (39 mg, 1.6 mmol) in DMF (5 mL) at room temperature. The mixture was stirred for 30 min before MOMCl (0.18 mL, 2.4 mmol) was introduced. After stirring for 30 min at room temperature, sat. NH_4Cl solution (5 mL), water (5 mL) and *tert*-butyl methyl ether (30 mL) were added and the layers separated. The aqueous phase was extracted with *tert*-butyl methyl ether (2 x 30 mL) and the combined organic layers were washed with brine (3 x 10 mL) and dried over $MgSO_4$. The solvent was removed under reduced pressure and the crude product was purified by flash chromatography (silica, hexanes/EtOAc 20:1 – 10:1) to give the title compound as a colorless oil (290 mg, 83%). ^{1}H NMR (400 MHz, $CDCl_3$) δ 7.75 – 7.69 (m, 2H), 7.28 – 7.22 (m, 2H), 5.14 (p, *J* = 1.6 Hz, 1H), 5.09 (s, 2H), 5.08 (m, 1H), 3.42 (s, 3H), 2.39 (s, 3H), 1.73 (dd, *J* = 1.6, 1.0 Hz, 3H), 1.68 (s, 6H). ^{13}C NMR (101 MHz, $CDCl_3$) δ 142.87, 140.40, 129.48, 127.07, 126.34, 122.03, 90.69, 85.24, 79.67, 56.45, 55.45, 31.19, 23.31, 21.59. IR (film) ṽ 2987, 2937, 1453, 1330, 1154, 1042 cm^{-1}. HRMS (ESI$^+$) for $C_{17}H_{23}NO_3S$ [M+Na]$^+$: calcd 344.1291, found 344.1294.

Hydrogenative C–H Insertion Reactions

Representative Procedure. Preparation of 4-(Cyclohex-1-en-1-yl)-2,2-dimethyltetrahydrofuran (7).

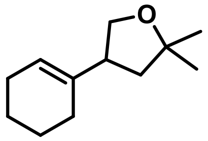

[Cp*RuCl]$_4$ (2.3 mg, 2 mol%) was added to a stirred solution of enyne **5** (19.0 mg, 0.11 mmol) in 1,2-dichloroethane (1.1 mL, 0.1 M) in a flame dried Schlenk tube under argon. H_2 was bubbled through the mixture for 2 min before the flask was immersed into a pre-heated oil bath (70 °C), keeping a static H_2 atmosphere (ambient pressure, balloon filled with H_2). After stirring for 3 h at 70 °C, the mixture was allowed to cool to room temperature, the solvent was removed under reduced pressure, and the crude product was purified by flash chromatography (silica, pentane/Et_2O 1:0 – 40:1) to yield the title compound as a colorless oil (17.4 mg, 89%). ^{1}H NMR (400 MHz, $CDCl_3$) δ 5.45 (tt, *J* = 3.7, 1.8 Hz, 1H), 3.94 (t, *J* = 7.9 Hz, 1H), 3.58 (dd, *J* = 9.5, 8.3 Hz, 1H), 2.96 – 2.82 (m, 1H), 1.99 (m, 2H), 1.95 – 1.88 (m, 2H), 1.84 (dd, *J* = 12.1, 7.7 Hz, 1H), 1.69 – 1.50 (m, 5H), 1.29 (s, 3H), 1.22 (s, 3H). ^{13}C NMR (101 MHz, $CDCl_3$) δ 136.7, 121.4, 80.9, 70.9, 47.2, 43.2, 29.0, 28.5, 26.9, 25.3, 23.1, 22.7. IR (film) ṽ 2967, 2924, 2858, 1449, 1364, 1050 cm^{-1}. HRMS (ESI$^+$) for $C_{12}H_{20}O$ [M+Na]$^+$: calcd 203.1406, found 203.1407.

4-(Cyclohex-1-en-1-yl)-2,2-dimethyltetrahydrofuran-3,3-[D$_2$] ([D$_2$]-7) Prepared according to the

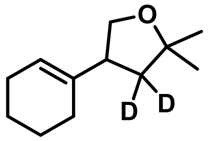

Representative Procedure from enyne **5** using D_2 instead of H_2 gas (19.1 mg, 0.11 mmol); colorless oil (15.9 mg, 81%). ^{1}H NMR (600 MHz, $CDCl_3$) δ 5.45 (tt, *J* = 3.7, 1.5 Hz, 1H), 3.94 (dd, *J* = 8.3, 7.6 Hz, 1H), 3.58 (dd, *J* = 9.5, 8.3 Hz, 1H), 2.88 (t, *J* = 8.7 Hz, 1H), 1.99 (m, 2H), 1.92 (m, 2H), 1.66 – 1.51 (m, 4H), 1.29 (s, 3H), 1.21 (s, 3H). ^{2}H NMR (92 MHz, $CDCl_3$) δ 1.83 (s, 1H), 1.63 (d, *J* = 1.6 Hz, 1H). ^{13}C NMR (151 MHz, $CDCl_3$) δ 136.7, 121.3, 80.8, 70.9, 47.0, 42.4 (p, *J* = 20.0 Hz), 28.9, 28.5, 26.9, 25.3, 23.1, 22.7. IR (film) ṽ 2967, 2925, 2857, 1447, 1161, 1045 cm^{-1}. HRMS (CI$^+$) for $C_{12}H_{18}D_2O$ [M+H]$^+$: calcd 183.1712, found 183.1713.

4-(Cyclohex-1-en-1-yl)-2,2-dimethyltetrahydrofuran-4,5,5-[D$_3$] ([D$_3$]-7). Prepared according to the Representative Procedure from enyne **[D$_3$]-5** (24.3 mg, 0.13 mmol); colorless oil (19.0 mg, 77%). ^{1}H NMR (600 MHz, CDCl$_3$) δ 5.45 (tt, *J* = 3.7, 1.7 Hz, 1H), 1.99 (m, 2H), 1.92 (m, 2H), 1.83 (d, *J* = 12.2 Hz, 1H), 1.67 – 1.51 (m, 5H), 1.29 (s, 3H), 1.21 (s, 3H). ^{2}H NMR (92 MHz, CDCl$_3$) δ 3.93 (s, 1H), 3.57 (s, 1H), 2.89 (s, 1H). ^{13}C NMR (151 MHz, CDCl$_3$) δ 136.7, 121.4, 80.9, 70.1 (pent, *J* = 22.0 Hz), 46.6 (t, *J* = 19.5 Hz), 43.1, 29.0, 28.5, 26.8, 25.3, 23.1, 22.7. IR (film) ṽ 2967, 2928, 2194 (br), 1095 (br), 1449, 1365, 1092, 1018 cm^{-1}. HRMS (EI$^+$) for $C_{12}H_{17}D_3O$ [M]$^+$: calcd 183.1697, found 183.1698.

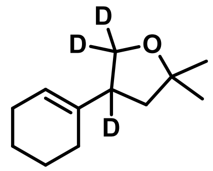

Gram-Scale Reaction. Preparation of *tert*-Butyl 7-(prop-1-en-2-yl)-5-oxa-2-azaspiro[3.4]octane-2-carboxylate (20). A flame dried two-necked round-bottom flask was charged with [Cp*RuCl]$_4$ (183 mg, 0.17 mmol). The flask was evacuated and refilled with H_2 (by means of attaching a balloon filled with hydrogen via a needle and septum). A solution of enyne **S27** (2.12 g, 8.43 mmol) in 1,2-dichloroethane (85 mL) was introduced before the flask was immersed into a pre-heated oil bath (70 °C), while keeping a static H_2 atmosphere (ambient pressure, balloon filled with H_2). After stirring for 3 h at 70 °C, the mixture was allowed to cool to room temperature, the flask was vented, and the solvent was removed under reduced pressure. The crude product was purified by flash chromatography (silica, hexanes/EtOAc 20:1 – 10:1) to yield the title product as a pale yellow oil (1.53 g, 72%). ^{1}H NMR (400 MHz, CDCl$_3$) δ 4.79 (s, 1H), 4.75 (s, 1H), 4.05 (d, *J* = 9.4 Hz, 1H), 4.01 – 3.91 (m, 3H), 3.82 (d, *J* = 8.8 Hz, 1H), 3.65 (t, *J* = 8.4 Hz, 1H), 2.86 (p, *J* = 8.1 Hz, 1H), 2.28 (dd, *J* = 12.7, 7.3 Hz, 1H), 2.01 (dd, *J* = 12.7, 9.6 Hz, 1H), 1.72 (s, 3H), 1.43 (s, 9H). ^{13}C NMR (101 MHz, CDCl$_3$) δ 156.5, 143.7, 111.1, 79.7, 78.6, 71.9, 63.3, 61.8, 45.8, 40.9, 28.5, 21.1. IR (film) ṽ 2973, 2944, 2875, 1699, 1391, 1157, 1094 cm^{-1}. HRMS (CI$^+$) for $C_{14}H_{23}NO_3$ [M+H]$^+$: calcd 254.1751, found 254.1749.

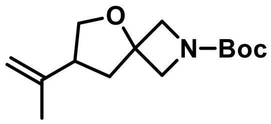

(*E*)-(2-(1-Oxaspiro[4.5]decan-3-yl)vinyl)trimethylsilane (10). Prepared according to the Representative Procedure from enyne **8** (26.9 mg, 0.11 mmol); colorless oil (22.3 mg, 92%). ^{1}H NMR (400 MHz, CDCl$_3$) δ 5.90 (dd, *J* = 18.5, 7.4 Hz, 1H), 5.68 (dd, *J* = 18.5, 1.0 Hz, 1H), 3.95 (t, *J* = 7.8 Hz, 1H), 3.51 (t, *J* = 8.7 Hz, 1H), 3.00 – 2.87 (m, 1H), 1.98 (dd, *J* = 12.4, 8.0 Hz, 1H), 1.72 – 1.30 (m, 10H), 1.45 (dd, *J* = 12.5, 9.5 Hz, 1H), 0.04 (s, 9H). ^{13}C NMR (101 MHz, CDCl$_3$) δ 146.9, 130.9, 83.3, 71.3, 46.8, 43.4, 38.3, 37.4, 25.8, 24.0, 24.0, –1.1. IR (film) ṽ 2930, 2856, 1615, 1246 cm^{-1}. HRMS (EI$^+$) for $C_{14}H_{26}OSi$ [M]$^+$: calcd 238.1747, found 238.1743.

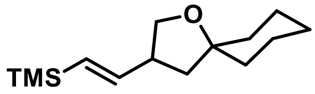

4-(Cyclohex-1-en-1-yl)-5-methoxy-2,2-dimethyltetrahydrofuran (11). Prepared according to the Representative Procedure from enyne **S25** (32.2 mg, 0.15 mmol); colorless oil (26.8 mg, 82%, mixture of diastereomers ~ 2:1). ^{1}H NMR (400 MHz, C_6D_6) major δ 5.46 (tt, *J* = 3.7, 1.7 Hz, 1H), 4.82 (d, *J* = 4.3 Hz, 1H), 3.21 (s, 3H), 2.72 – 2.62 (m, 1H), 2.10 (dd, *J* = 12.7, 11.5 Hz, 1H), 2.05 – 1.89 (m, 4H), 1.67 – 1.42 (m, 4H), 1.55 (dd, *J* = 11.5, 7.4 Hz, 1H), 1.38 (s, 3H), 1.18 (s, 3H). minor δ 5.56 (ddt, *J* = 5.1, 3.6, 1.6 Hz, 1H), 4.92 (d, *J* = 3.7 Hz, 1H), 3.31 (s, 3H), 3.02 (m, 1H), 2.05 – 1.89 (m, 4H), 1.81 (dd, *J* = 12.2, 8.3 Hz, 1H), 1.67 – 1.42 (m, 4H), 1.66 (dd, *J* = 12.3, 9.6 Hz, 1H), 1.37 (s, 3H), 1.30 (s, 3H). ^{13}C NMR (101 MHz, C_6D_6) major δ 135.0, 122.2, 105.4, 81.2, 53.9, 51.7, 39.1, 31.3, 29.4, 28.5, 25.7, 23.4, 23.0. minor δ 136.9, 121.7, 109.8, 82.0, 55.1, 54.2, 42.6, 29.7, 29.2, 27.4, 25.6, 23.4, 22.9. IR (film) ṽ 2925, 2835, 1450, 1108, 1032, 1013 cm^{-1}. HRMS (CI$^+$) for $C_{13}H_{22}O$ [M+H]$^+$: calcd 211.1693, found 211.1691.

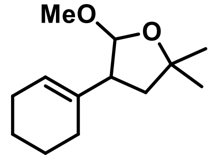

3-(Prop-1-en-2-yl)-1-oxaspiro[4.4]nonane (12). Prepared according to the Representative Procedure from

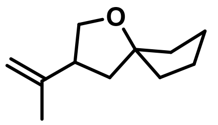

enyne **S22** (32.2 mg, 0.20 mmol); colorless oil (22.9 mg, 70%). ^{1}H NMR (400 MHz, $CDCl_3$) δ 4.74 (p, *J* = 1.1 Hz, 2H), 3.94 (t, *J* = 8.0 Hz, 1H), 3.58 (t, *J* = 8.6 Hz, 1H), 3.01 – 2.89 (m, 1H), 1.98 (dd, *J* = 12.2, 7.7 Hz, 1H), 1.87 – 1.46 (m, 8H), 1.82 (dd, *J* = 12.2, 10.2 Hz, 1H), 1.73 (t, *J* = 1.2 Hz, 3H). ^{13}C NMR (101 MHz, $CDCl_3$) δ 145.2, 110.2, 91.8, 70.6, 46.8, 41.5, 38.9, 38.8, 24.3, 24.0, 21.0. IR (film) ṽ 2957, 2869, 1646, 1449, 1052 cm^{-1}. HRMS (EI$^+$) for $C_{11}H_{18}O$ [M]$^+$: calcd 166.1352, found 166.1351.

7-(Cyclohex-1-en-1-yl)-5-oxaspiro[3.4]octane (13). Prepared according to the Representative Procedure

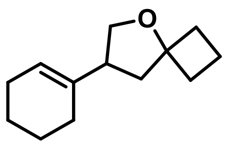

from enyne **S23** (18.6 mg, 0.10 mmol); colorless oil (9.7 mg, 52%, 75% NMR). ^{1}H NMR (400 MHz, $CDCl_3$) δ 5.47 (m, 1H), 3.88 (t, *J* = 7.9 Hz, 1H), 3.54 (dd, *J* = 9.3, 8.0 Hz, 1H), 2.79 (p, *J* = 9.7 Hz, 1H), 2.30 (q, *J* = 10.4 Hz, 1H), 2.17 – 2.02 (m, 4H), 1.99 (m, 2H), 1.93 (m, 2H), 1.78 (dd, *J* = 11.8, 10.6 Hz, 1H), 1.73 – 1.42 (m, 6H). ^{13}C NMR (101 MHz, $CDCl_3$) δ 136.5, 121.6, 83.8, 71.2, 46.1, 41.4, 37.2, 35.7, 26.8, 25.4, 23.1, 22.7, 12.8. IR (film) ṽ 2925, 2857, 1438, 1122, 1026 cm^{-1}. HRMS (EI$^+$) for $C_{13}H_{20}O$ [M]$^+$: calcd 192.1509, found 192.1511.

3-(Prop-1-en-2-yl)-8-tosyl-1-oxa-8-azaspiro[4.5]decane (14). Prepared according to the Representative

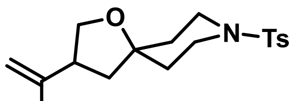

Procedure from enyne **S28** (37.1 mg, 0.11 mmol); colorless oil (30.2 mg, 81%). ^{1}H NMR (400 MHz, $CDCl_3$) δ 7.65 – 7.61 (m, 2H), 7.32 – 7.28 (m, 2H), 4.74 (m, 1H), 4.70 (m, 1H), 3.87 (dd, *J* = 8.5, 7.4 Hz, 1H), 3.51 (dd, *J* = 9.3, 8.5 Hz, 1H), 3.47 – 3.40 (m, 2H), 2.88 (p, *J* = 8.0 Hz, 1H), 2.72 (qd, *J* = 11.5, 3.4 Hz, 2H), 2.42 (s, 3H), 1.88 (dd, *J* = 12.5, 7.9 Hz, 1H), 1.84 – 1.65 (m, 4H), 1.69 (t, *J* = 1.1 Hz, 3H), 1.59 (dd, *J* = 12.5, 10.4 Hz, 1H). ^{13}C NMR (101 MHz, $CDCl_3$) δ 144.1, 143.5, 133.5, 129.8, 127.8, 110.7, 79.3, 70.6, 45.9, 43.7, 43.4, 42.3, 36.5, 36.3, 21.7, 21.1. IR (film) ṽ 2945, 2926, 2861, 2244, 1329, 1169, 1048 cm^{-1}. HRMS (EI$^+$) for $C_{18}H_{25}NO_3S$ [M]$^+$: calcd 335.1550, found 355.1551.

Single crystals suitable for X-ray analysis were obtained by slow evaporation of a concentrated solution in CH_2Cl_2/pentane (1:1).

3-(Prop-1-en-2-yl)-1,8-dioxaspiro[4.5]decane (15). Prepared according to the Representative Procedure

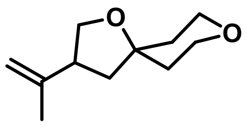

from enyne **38** (21.5 mg, 0.12 mmol); colorless oil (14.9 mg, 67%). ^{1}H NMR (400 MHz, $CDCl_3$) δ 4.77 (m, 1H), 4.75 (m, 1H), 4.00 (dd, *J* = 8.4, 7.5 Hz, 1H), 3.80 (m, 2H), 3.69 – 3.62 (m, 2H), 3.62 (dd, *J* = 9.3, 8.5 Hz, 1H), 2.95 (p, *J* = 9.0 Hz, 1H), 1.99 (dd, *J* = 12.4, 8.0 Hz, 1H), 1.73 (t, *J* = 1.0 Hz, 3H), 1.76 – 1.58 (m, 5H). ^{13}C NMR (101 MHz, $CDCl_3$) δ 144.5, 110.6, 79.9, 70.5, 65.6, 65.4, 46.0, 42.2, 38.2, 37.9, 21.2. IR (film) ṽ 2940, 2859, 1646, 1443, 1230, 1103, 1009 cm^{-1}. HRMS (EI$^+$) for $C_{11}H_{18}O_2$ [M]$^+$: calcd 182.1301, found 182.1303.

8,8-Dimethyl-3-(prop-1-en-2-yl)-1,7,9-trioxaspiro[4.5]decane (16). Prepared according to the

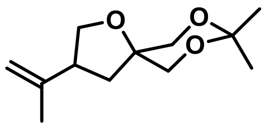

Representative Procedure from enyne **S29** (22.7 mg, 0.11 mmol); colorless oil (18.0 mg, 79%). ^{1}H NMR (400 MHz, $CDCl_3$) δ 4.79 (m, 1H), 4.75 (m, 1H), 4.04 (dd, *J* = 8.3, 7.4 Hz, 1H), 3.80 (dd, *J* = 11.6, 1.1 Hz, 1H), 3.75 – 3.60 (m, 4H), 2.92 – 2.81 (m, 1H), 2.13 (dd, *J* = 12.8, 7.6 Hz, 1H), 1.73 (s, 3H), 1.61 (dd, *J* = 12.9, 10.6 Hz, 1H), 1.46 (s, 3H), 1.42 (s, 3H). ^{13}C NMR (101 MHz, $CDCl_3$) δ 143.6, 110.9, 98.3, 77.5, 71.5, 68.0, 67.6, 45.6, 37.6,

23.8, 23.6, 21.5. IR (film) ṽ 2991, 2942, 2870, 1647, 1371, 1199, 1082 cm^{-1}. HRMS (ESI$^+$) for $C_{12}H_{20}O_3$ [M+Na]$^+$: calcd 235.1305, found 235.1303.

(5*R*,6*S*,9*R*)-6-Isopropyl-9-methyl-3-(prop-1-en-2-yl)-1-oxaspiro[4.5]decane (17). Prepared according to the Representative Procedure from enyne **S24** (21.3 mg, 0.09 mmol), colorless oil (7.8 mg, 36%; single isomer, isopropenyl stereochemistry unknown). ^{1}H NMR (400 MHz, $CDCl_3$) δ 4.75 (m, 1H), 4.72 (m, 1H), 3.94 (dd, *J* = 8.2, 6.9 Hz, 1H), 3.51 (dd, *J* = 11.0, 8.3 Hz, 1H), 2.82 – 2.69 (m, 1H), 2.08 (dd, *J* = 12.7, 8.6 Hz, 1H), 1.96 (hept, *J* = 6.8 Hz, 1H), 1.78 – 1.67 (m, 6H), 1.53 – 1.37 (m, 3H), 1.06 (ddd, *J* = 12.2, 4.1, 1.5 Hz, 1H), 0.95 – 0.81 (m, 11H). ^{13}C NMR (101 MHz, $CDCl_3$) δ 144.6, 110.3, 86.0, 70.1, 50.3, 48.7, 47.6, 42.6, 35.1, 29.5, 26.7, 24.0, 22.6, 21.8, 21.6, 18.3. IR (film) ṽ 2950, 2867, 1647, 1454, 1051 cm^{-1}. HRMS (CI$^+$) for $C_{16}H_{28}O$ [M+H]$^+$: calcd 237.2213, found 237.2217.

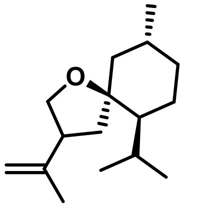

Ethyl (*E*)-3-(1-oxaspiro[4.5]decan-3-yl)acrylate (18). According to the Representative Procedure from enyne **S34** (28.2 mg, 0.12 mmol); colorless oil (18.2 mg, 64%). ^{1}H NMR (400 MHz, $CDCl_3$) δ 6.86 (dd, *J* = 15.6, 8.5 Hz, 1H), 5.84 (dd, *J* = 15.6, 1.1 Hz, 1H), 4.18 (q, *J* = 7.1 Hz, 2H), 3.99 (dd, *J* = 8.7, 7.3 Hz, 1H), 3.59 (t, *J* = 8.5 Hz, 1H), 3.06 (m, 1H), 2.02 (dd, *J* = 12.6, 8.2 Hz, 1H), 1.72 – 1.32 (m, 10H), 1.53 (dd, *J* = 12.5, 9.1 Hz, 1jH), 1.28 (t, *J* = 7.1 Hz, 3H). ^{13}C NMR (101 MHz, $CDCl_3$) δ 166.6, 148.8, 121.8, 83.5, 70.6, 60.5, 43.2, 42.6, 38.0, 37.3, 25.6, 23.9 (2C), 14.4. IR (film) ṽ 2929, 2856, 1717, 1654, 1447, 1266, 1183, 1034 cm^{-1}. HRMS (EI$^+$) for $C_{14}H_{22}O_3$ [M+Na]$^+$: calcd 238.1563, found 238.1564.

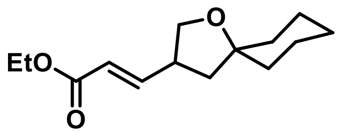

(1-(1-Oxaspiro[4.5]decan-3-yl)vinyl)trimethylsilane (19). According to the Representative Procedure from enyne **S35** (25.5 mg, 0.11 mmol); colorless oil (21.4 mg, 83%). ^{1}H NMR (400 MHz, $CDCl_3$) δ 5.66 (dd, *J* = 2.4, 1.5 Hz, 1H), 5.40 (dd, *J* = 2.3, 1.0 Hz, 1H), 3.99 (ddd, *J* = 8.0, 7.0, 0.8 Hz, 1H), 3.52 (dd, *J* = 9.9, 8.3 Hz, 1H), 3.07 – 2.96 (m, 1H), 1.96 (ddd, *J* = 12.2, 7.6, 0.8 Hz, 1H), 1.76 – 1.25 (m, 11H), 0.10 (s, 9H). ^{13}C NMR (101 MHz, $CDCl_3$) δ 151.6, 123.8, 82.9, 71.9, 44.7, 43.1, 38.6, 37.8, 25.8, 24.0, 23.9, –1.0. IR (film) ṽ 2929, 2856, 1447, 1248, 1053 cm^{-1}. HRMS (EI$^+$) for $C_{14}H_{26}OSi$ [M]$^+$: calcd 238.1747, found 238.1743.

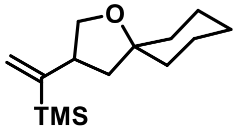

***tert*-Butyl 7-(prop-1-en-2-yl)-5-oxa-2-azaspiro[3.4]octane-2-carboxylate-6,6,7,8,8-[D$_5$] ([D$_5$]-20).** Prepared according to the Representative Procedure from enyne **[D$_3$]-S27** (33.6 mg, 0.13 mmol) but using D_2 instead of H_2 gas; colorless oil (23.5 mg, 69%). ^{1}H NMR (600 MHz, $CDCl_3$) δ 4.78 (m, 1H), 4.74 (m, 1H), 4.04 (dd, *J* = 9.3, 1.2 Hz, 1H), 3.93 (ddd, *J* = 13.0, 9.1, 1.2 Hz, 2H), 3.82 (dd, *J* = 9.0, 1.2 Hz, 1H), 1.73 – 1.70 (m, 3H), 1.42 (s, 9H). ^{2}H NMR (92 MHz, $CDCl_3$) δ 3.91 (s, 1H), 3.58 (s, 1H), 2.79 (s, 1H), 2.21 (s, 1H), 1.94 (s, 1H). ^{13}C NMR (151 MHz, $CDCl_3$) δ 156.5, 143.6, 111.0, 79.6, 78.5, 71.1 (p, *J* = 22.3 Hz), 63.2, 61.7, 45.1 (t, *J* = 20.0 Hz), 40.1 (p, *J* = 20.0 Hz), 28.5, 21.0. IR (film) ṽ 2975, 2875, 2228, 2116, 1699, 1390, 1098 cm^{-1}. HRMS (ESI$^+$) for $C_{14}H_{18}D_5NO_3$ [M+Na]$^+$: calcd 281.1884, found 281.1888.

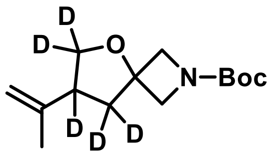

tert-Butyl 7-(3,3-diethoxyprop-1-en-2-yl)-5-oxa-2-azaspiro[3.4]octane-2-carboxylate (21). Prepared analogously from enyne **S36** (30.7 mg, 0.090 mmol); colorless oil (27.3 mg, 88 %). ^{1}H NMR (400 MHz, C_6D_6) δ 5.1 (s, 1H), 4.8 (s, 1H), 4.5 (s, 1H), 4.2 (d, *J* = 9.0 Hz, 1H), 4.1 – 4.0 (m, 2H), 3.9 (d, *J* = 8.9 Hz, 1H), 3.7 (d, *J* = 8.2 Hz, 1H), 3.5 (t, *J* = 8.4 Hz, 1H), 3.5 – 3.3 (m, 2H), 3.2 (tq, *J* = 9.4, 7.1 Hz, 2H), 3.0 – 2.8 (m, 1H), 1.9 (dd, *J* = 12.5, 7.1 Hz, 1H), 1.8 (dd, *J* = 12.5, 9.8 Hz, 1H), 1.5 (s, 9H), 1.1 (q, *J* = 7.0 Hz, 6H). ^{13}C NMR (101 MHz, C_6D_6) δ 156.3, 146.2, 112.3, 104.4, 78.9, 78.3, 73.3, 63.7 (br), 62.5 (br), 61.9, 61.8, 41.8, 39.9, 28.5, 15.3, 15.3. IR (film) ṽ 2926, 2815, 1700, 1319, 1059 cm^{-1}.HRMS (CI^{+}) for $C_{18}H_{31}NO_5Na$ [M+Na]$^{+}$: calcd 364.2094, found 364.2095.

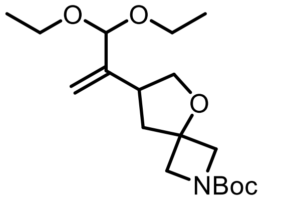

tert-Butyl 7-(4-((_tert_-butyldimethylsilyl)oxy)but-1-en-2-yl)-5-oxa-2-azaspiro[3.4]octane-2-carboxylate (22). Prepared analogously from enyne **S37** (36.0 mg, 0.091 mmol), colorless oil (26.0 mg, 72 %). ^{1}H NMR (400 MHz, $CDCl_3$) δ 4.83 (m, 2H), 4.06 – 3.98 (m, 2H), 3.93 (t, *J* = 9.7 Hz, 2H), 3.81 (dd, *J* = 8.9, 1.1 Hz, 1H), 3.70 (t, *J* = 6.9 Hz, 2H), 3.63 (t, *J* = 8.4 Hz, 1H), 2.89 (p, *J* = 8.1 Hz, 1H), 2.29 (dd, *J* = 12.6, 7.1 Hz, 1H), 2.22 (tt, *J* = 7.0, 1.3 Hz, 2H), 2.01 (dd, *J* = 12.6, 9.6 Hz, 1H), 1.42 (s, 9H), 0.88 (s, 9H), 0.04 (s, 6H). ^{13}C NMR (101 MHz, $CDCl_3$) δ 156.5, 145.4, 111.0, 79.6, 78.5, 72.4, 63.3, 62.6, 61.9, 44.9, 41.3, 38.6, 28.5, 26.0, 18.4, -5.2. IR (film) ṽ 2930, 2858, 1703, 1391, 1253, 1158, 1092 cm^{-1}. HRMS (ESI^{+}) for $C_{21}H_{39}NO_4SiNa$ [M+Na]$^{+}$: calcd 420.2541 found 420.2541.

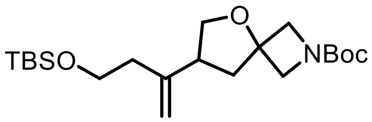

Di-_tert_-butyl (6S*,7R*)-7-(prop-1-en-2-yl)-5-oxa-2-azaspiro[3.4]octane-2,6-dicarboxylate (23). Prepared according to the Representative Procedure from enyne **S26** (44.2 mg, 0.13 mmol), colorless oil (36.1 mg containing 10% *trans*-hydrogenation product, 73%). An aliquot (26.4 mg) was purified by HPLC to yield an analytically pure sample (19.8 mg). ^{1}H NMR (400 MHz, $CDCl_3$) mixture of diastereomers (~3:1 in favor of title compound); major δ 4.85 (p, *J* = 1.5 Hz, 1H), 4.84 – 4.82 (m, 1H), 4.26 (d, *J* = 7.0 Hz, 1H), 4.21 (dd, *J* = 9.3, 1.1 Hz, 1H), 4.10 (dd, *J* = 9.4, 1.0 Hz, 1H), 3.92 (dd, *J* = 9.4, 1.2 Hz, 1H), 3.84 (dd, *J* = 9.3, 1.2 Hz, 1H), 2.91 (q, *J* = 7.6 Hz, 1H), 2.32 (dd, *J* = 12.7, 7.2 Hz, 1H), 2.10 (dd, *J* = 12.7, 8.9 Hz, 1H), 1.75 (dd, *J* = 1.5, 0.8 Hz, 3H), 1.45 (s, 9H), 1.42 (s, 9H); minor δ 4.87 (p, *J* = 1.3 Hz, 1H), 4.78 (m, 1H), 4.52 (d, *J* = 7.8 Hz, 1H), 4.30 (dd, *J* = 9.5, 1.0 Hz, 1H), 4.03 (dd, *J* = 9.7, 1.2 Hz, 1H), 3.99 (dd, *J* = 9.0, 1.1 Hz, 1H), 3.84 (m, 1H), 2.98 (dt, *J* = 13.1, 6.9 Hz, 1H), 2.42 (t, *J* = 12.4 Hz, 1H), 2.23 (dd, *J* = 12.6, 6.6 Hz, 1H), 1.83 (s, 3H), 1.43 (s, 9H), 1.39 (s, 9H). ^{13}C NMR (101 MHz, $CDCl_3$) major δ 171.8, 156.5, 142.5, 112.8, 81.9, 81.5, 79.8, 79.7, 63.5, 60.9, 50.8, 41.1, 28.5, 28.1, 20.2; minor 170.2, 156.6, 140.8, 112.3, 82.0, 80.6, 79.6, 79.4, 63.8, 63.0, 49.4, 38.2, 28.5, 28.2, 23.4. IR (film) ṽ 2976, 2938, 1701, 1392, 1366, 1155, 1089 cm^{-1}. HRMS (ESI^{+}) for $C_{19}H_{31}NO_5$ [M+Na]$^{+}$: calcd 376.2094, found 376.2096.

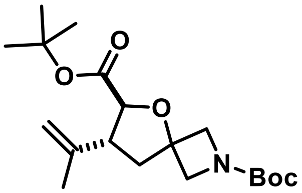

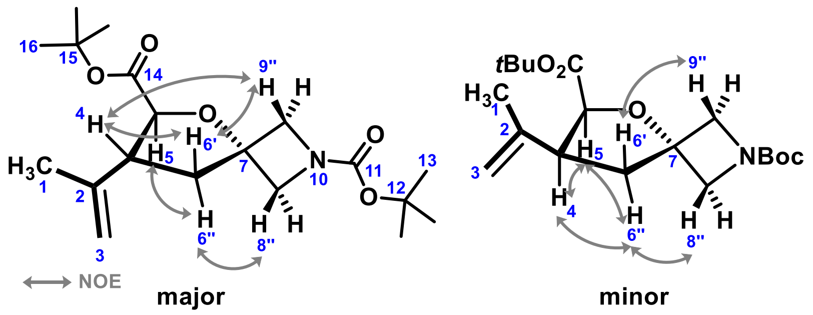

Table S7. Detailed NMR analysis of product **23** ($CDCl_3$, 25 °C, 600 MHz)

Atom [#] C	H	δ [ppm]	COSY	HSQC	HMBC	NOESY
**1**		20.08		1	3', 3'', 4	
	**1**	1.75	3', 3''	1	2, 3, 4	3', 4, 5, 6''
**2**		142.4			1, 3', 3'', 4, 5, 6', 6''	
**3**		112.62		3', 3''	1, 4	
	**3'**	4.85	1, 3''	3	1, 2, 4	1, 16
	**3''**	4.83	1, 3', 4	3	1, 2, 4	4, 5, 6'', 8', 8'', 16
**4**		50.66		4	1, 3', 3'', 5, 6', 6''	
	**4**	2.91	3'', 5, 6', 6''	4	1, 2, 3, 5, 6, 7, 14	1, 3'', 6', 9'', 16
**5**		81.39		5	4, 6', 6''	
	**5**	4.26	4, 6'	5	2, 4, 6, 7, 14	1, 3'', 6'', 16
**6**		40.92		6', 6''	4, 5, 8', 8'', 9', 9''	
	**6'**	2.32	4, 5, 6''	6	2, 4, 5, 7, 8, 9	4, 9''
	**6''**	2.1	4, 6'	6	2, 4, 5, 7, 8, 9	1, 3'', 5, 8''
**7**		79.68			4, 5, 6', 6'', 8', 8'', 9', 9''	
**8**		60.75		8', 8''	6', 6'', 9', 9''	
	**8'**	4.1	8'', 9''	8	6, 7, 9, 11	3'', 13
	**8''**	3.92	8', 9'	8	6, 7, 9, 11	3'', 6'', 13
**9**		63.32		9', 9''	6', 6'', 8', 8''	
	**9'**	4.21	8'', 9''	9	6, 7, 8, 11	13, 16
	**9''**	3.84	8', 9'	9	6, 7, 8, 11	4, 6', 13
**10 N**		-315.6				
**11**		156.34			8', 8'', 9', 9''	
**12**		79.5			13	
**13**		28.34		13		
	**13**	1.42		13	12	8', 8'', 9', 9''
**14**		171.61			4, 5	
**15**		81.76			16	
**16**		27.96		16		
	**16**	1.45		16	15	3', 3'', 4, 5, 9'

***tert*-Butyl 9-(prop-1-en-2-yl)-7-oxa-2-azadispiro[3.1.4^6.1^4]undecane-2-carboxylate (24).** Prepared according to the Representative Procedure from enyne **S30** (36.0 mg, 0.12 mmol); colorless oil (27.7 mg, 76%). ^{1}H NMR (400 MHz, $CDCl_3$) δ 4.76 (m, 1H), 4.74 (m, 1H), 3.90 (m, 5H), 3.55 (dd, *J* = 9.0, 8.2 Hz, 1H), 2.87 (p, *J* = 9.0 Hz, 1H), 2.49 – 2.43 (m, 1H), 2.35 – 2.25 (m, 2H), 2.18 (dd, *J* = 11.8, 4.8 Hz, 1H), 1.98 (dd, *J* = 12.1, 7.2 Hz, 1H), 1.80 (dd, *J* = 12.1, 10.3 Hz, 1H), 1.73 – 1.69 (m, 3H), 1.42 (s, 9H). ^{13}C NMR (101 MHz, $CDCl_3$) δ 156.3, 144.2, 110.8, 79.4, 79.4, 71.1, 61.8 (br), 60.4 (br), 47.6, 46.1, 46.0, 42.0, 29.6, 28.5, 21.0. IR (film) ṽ 2924, 2869, 1699, 1391, 1165, 1062 cm^{-1}. HRMS (CI^+) for $C_{17}H_{27}NO_3$ $[M+H]^+$: calcd 294.2064, found 294.2063.

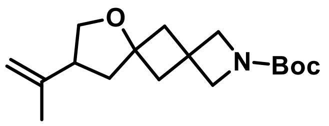

3-Methoxy-1,1-dimethyl-4-(prop-1-en-2-yl)cyclopentane (26). Prepared according to the Representative Procedure from enyne **25** (43.0 mg, 0.259 mmol) after 14 h reaction time to reach full conversion (34.9 mg, 80%, dr ≈ 2:1). ^{1}H NMR (400 MHz, $CDCl_3$) major isomer (*cis*): δ 4.85 – 4.82 (m, 1H), 4.72 – 4.69 (m, 1H), 3.80 (td, *J* = 4.9, 1.6 Hz, 1H), 3.23 (s, 3H), 2.62 – 2.52 (m, 1H), 1.84 – 1.72 (m, 5H), 1.59 – 1.50 (m, 2H), 1.13 (s, 3H), 1.02 (s, 3H); minor isomer (*trans*): δ 4.81 – 4.79 (m, 1H), 4.77 – 4.77 (m, 1H), 3.70 (td, *J* = 7.7, 6.2 Hz, 1H), 3.30 (s, 3H), 2.66 (dtd, *J* = 11.7, 8.0, 1.1 Hz, 1H), 1.84 – 1.74 (m, 4H), 1.63 (ddd, *J* = 12.7, 8.0, 1.3 Hz, 1H), 1.48 (ddd, *J* = 13.1, 6.3, 1.3 Hz, 1H), 1.37 (dd, *J* = 12.7, 11.6 Hz, 1H), 1.09 (s, 3H), 1.03 (s, 3H). ^{13}C NMR (101 MHz, $CDCl_3$) major isomer (*cis*): δ 145.4, 110.6, 84.3, 56.4, 51.1, 45.6, 42.7, 36.7, 32.0, 31.5, 22.7; minor isomer (*trans*): δ 146.9, 110.0, 85.1, 57.4, 53.0, 46.1, 44.7, 36.3, 31.2, 30.6, 20.7. IR (film) ṽ 3085, 2949, 2867, 2819, 1646, 1458, 1363, 1268, 1195, 1163, 1128, 1090, 996, 883 cm^{-1}. HRMS (ESI^+) for $C_{11}H_{21}O$ $[M+H]^+$: calcd 169.1587, found 169.1587.

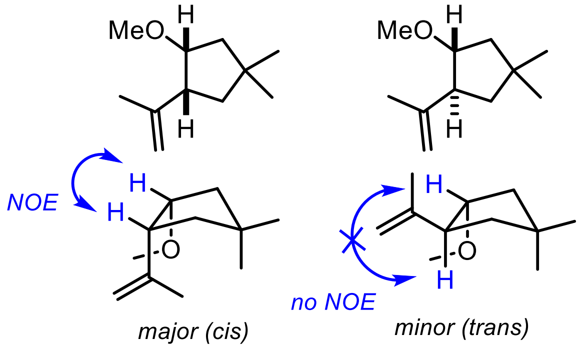

***tert*-Butyl (*E*)-7-(3-((*tert*-butyldimethylsilyl)oxy)prop-1-en-1-yl)-5-oxa-2-azaspiro[3.4]octane-2-carboxylate (S51).** Prepared analogously from enyne **S31** (39.7 mg, 0.10 mmol); colorless oil (18.0 mg 45%).^{1}H NMR (400 MHz, $CDCl_3$) δ 5.68 – 5.57 (m, 1H), 5.53 (ddt, *J* = 15.3, 7.7, 1.4 Hz, 1H), 4.12 (ddd, *J* = 4.8, 1.4, 0.7 Hz, 2H), 4.04 (dd, *J* = 9.2, 1.0 Hz, 1H), 4.01 – 3.88 (m, 3H), 3.82 (dd, *J* = 9.0, 1.1 Hz, 1H), 3.53 (t, *J* = 8.2 Hz, 1H), 2.90 (h, *J* = 7.6 Hz, 1H), 2.30 (dd, *J* = 12.8, 7.2 Hz, 1H), 1.90 (dd, *J* = 12.8, 8.9 Hz, 1H), 1.43 (s, 9H), 0.90 (s, 9H), 0.06 (s, 6H). ^{13}C NMR (101 MHz, $CDCl_3$) δ 156.5, 131.1, 129.4, 79.7, 78.6, 73.1, 63.5, 63.0, 62.3, 43.0, 42.1, 28.5, 26.1, 18.6, -5.0. IR (film) ṽ 2929, 2987, 1703, 1391, 1251, 1090 cm^{-1}. HRMS (ESI^+) for $C_{20}H_{37}NO_4SiNa$ $[M+Na]^+$: calcd 406.2384, found 406.2386.

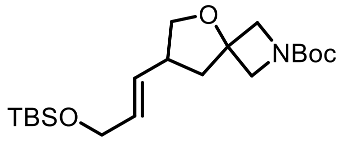

***tert*-Butyl 7-(3,3,3-trifluoroprop-1-en-2-yl)-5-oxa-2-azaspiro[3.4]octane-2-carboxylate (S52).** Prepared analogously from enyne **S38** (32.7 mg, 0.11 mmol); pale yellow oil (11.0 mg, 36 %). ^{1}H NMR (400 MHz, $CDCl_3$) δ 5.81 – 5.75 (m, 1H), 5.47 – 5.41 (m, 1H), 4.13 – 4.04 (m, 2H), 3.97 (ddd, *J* = 19.3, 9.2, 1.1 Hz, 2H), 3.85 (dd, *J* = 9.1, 1.1 Hz, 1H), 3.74 – 3.66 (m, 1H), 3.03 (p, *J* = 7.6 Hz, 1H), 2.45 (dd, *J* = 12.9, 7.2 Hz, 1H), 2.12 (dd, *J* = 12.9, 9.1 Hz, 1H), 1.43 (s, 9H). ^{13}C NMR (101 MHz, $CDCl_3$) δ 156.3, 138.0 (d, *J* = 29.4 Hz), 123.4 (q,

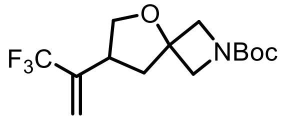

J = 273.9 Hz), 117.8 (q, J = 5.8 Hz), 79.7, 78.3, 72.2, 63.0 (br), 61.6 (br), 38.4, 28.4. ^{19}F NMR (282 MHz, $CDCl_3$) δ –67.9. IR (film) ṽ 2977, 2877, 1699, 1393, 1119 cm^{-1}. HRMS (ESI$^+$) for $C_{14}H_{20}NO_3FNa$ [M+Na]$^+$: calcd 330.1287 found 330.1286.

Acetal and Orthoester Series

1-Cyclohexyl-6-(prop-1-en-2-yl)-2,8-dioxabicyclo[3.2.1]octane (30). Prepared analogously from enyne **27**

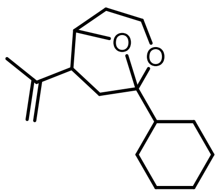

(26.4 mg, 0.11 mmol); colorless oil (21.8 mg, 82 %, mixture of diastereoisomers ~ 2.5:1). ^{1}H NMR (400 MHz, C_6D_6) major δ 4.8 (q, J = 1.0 Hz, 1H), 4.8 (h, J = 1.5 Hz, 1H), 4.2 (ddt, J = 6.6, 4.1, 1.3 Hz, 1H), 3.8 (td, J = 12.0, 4.2 Hz, 1H), 3.7 – 3.6 (m, 1H), 2.8 – 2.7 (m, 1H), 2.3 (dtt, J = 14.2, 3.2, 1.6 Hz, 1H), 2.1 – 2.1 (m, 1H), 2.0 – 1.9 (m, 2H), 1.9 – 1.7 (m, 4H), 1.7 – 1.6 (m, 2H), 1.4 (q, J = 1.0 Hz, 3H), 1.4 – 1.1 (m, 5H), 1.0 – 0.8 (m, 1H), minor δ 4.8 (q, J = 1.6 Hz, 1H), 4.7 (p, J = 1.5 Hz, 1H), 4.2 (t, J = 2.9 Hz, 1H), 3.7 – 3.6 (m, 2H), 2.5 (dd, J = 9.2, 4.4 Hz, 1H), 2.2 (dtt, J = 15.5, 3.3, 1.9 Hz, 1H), 2.1 – 2.0 (m, 1H), 2.0 – 1.9 (m, 2H), 1.9 – 1.7 (m, 4H), 1.7 – 1.6 (m, 2H), 1.5 – 1.4 (m, 3H), 1.4 – 1.1 (m, 5H), 0.7 – 0.6 (m, 1H). ^{13}C NMR (101 MHz, C_6D_6) major δ 141.6, 110.8, 109.3, 76.4, 59.6, 48.5, 46.4, 38.1, 33.3, 27.5, 27.4, 27.1, 26.8, 26.8, 26.7, 23.2; minor δ 148.5, 110.0, 109.3, 79.5, 59.4, 48.7, 46.2, 38.1, 31.2, 27.8, 27.7, 27.0, 26.7, 19.8. IR (film) ṽ 2926, 2853, 1647, 1451, 1319, 1114 cm^{-1}. HRMS (CI$^+$) for $C_{15}H_{24}O_2$ [M+H]$^+$: calcd 236.1771, found 236.1771.

(3S*,5R*)-3-(Prop-1-en-2-yl)-1,6-dioxaspiro[4.4]nonane (31a). Prepared according to the Representative

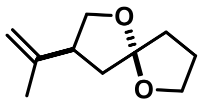

Procedure from enyne **28a** (34.4 mg, 0.21 mmol); colorless oil (26.2 mg, 75%). ^{1}H NMR (400 MHz, C_6D_6) δ 4.79 (dp, J = 1.8, 0.9 Hz, 1H), 4.73 (p, J = 1.7 Hz, 1H), 3.91 – 3.84 (m, 2H), 3.80 (dd, J = 9.9, 8.2 Hz, 1H), 3.76 – 3.70 (m, 1H), 2.73 (ttd, J = 9.7, 8.1, 1.1 Hz, 1H), 2.16 (dd, J = 13.4, 8.3 Hz, 1H), 2.02 – 1.94 (m, 2H), 1.93 – 1.81 (m, 1H), 1.62 (t, J = 1.2 Hz, 3H), 1.61 – 1.48 (m, 2H). ^{13}C NMR (101 MHz, C_6D_6) δ 144.6, 115.1, 111.2, 70.3, 67.0, 46.7, 39.9, 36.1, 24.9, 20.4. IR (film) ṽ 2973, 2880, 1646, 1441, 1141, 1011 cm^{-1}. HRMS (ESI$^+$) for $C_{10}H_{16}O_2$ [M+Na]$^+$: calcd 191.1042, found 191.1042.

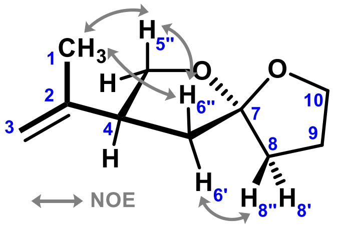

Table S8. Detailed NMR analysis of spiroketal **31a** (CD_2Cl_2, 25 °C, 600 MHz)

Atom [#] C	H	δ [ppm]	J [Hz]	COSY	HSQC	HMBC	NOESY
**1**		20.83			1	3, 4	
	**1**	1.74		3, 4	1	2, 3, 4	3, 4, 5', 5''
**2**		144.83				1, 3, 4, 5', 5'', 6', 6''	
**3**		110.71			3	1, 4	
	**3**	4.77		1, 4	3	1, 2, 4, 5	1, 4, 5'', 6''
**4**		46.47			4	1, 3, 5', 5'', 6', 6''	

	**4**	2.89		1, 3, 5', 5'', 6', 6''	4	1, 2, 3, 5, 6	1, 3, 5'
**5**		70.5			5', 5''	3, 4, 6'	
	**5'**	3.89	8.2, 7.5	4, 5'', 6', 6''	5	2, 4, 6, 7	1, 4
	**5''**	3.61	10.1, 8.2	4, 5', 6'	5	2, 4, 6	1, 3
**6**		40.09			6', 6''	4, 5', 5''	
	**6'**	2.2	13.4, 9.5	4, 5', 5'', 6''	6	2, 4, 5, 8	8''
	**6''**	2.05	13.3, 9.1	4, 5', 6'	6	2, 4, 7, 8	3
**7**		115.41				5', 6'', 8', 8'', 9', 9''	
**8**		36.46			8', 8''	6', 6'', 9', 9'', 10	
	**8'**	2.02		8'', 9', 9''	8	7, 9, 10	
	**8''**	1.88		8', 9', 9''	8	7, 9, 10	6'
**9**		24.92			9', 9''	8', 8'', 10	
	**9'**	2.01		8', 8'', 9'', 10	9	7, 8, 10	
	**9''**	1.88		8', 8'', 9', 10	9	7, 8, 10	
**10**		67.35			10	8', 8'', 9', 9''	
	**10**	3.83		9', 9''	10	8, 9	

(3S*,5R*)-2,2-Dimethyl-3-(prop-1-en-2-yl)-1,6-dioxaspiro[4.4]nonane (31b). Prepared according to the Representative Procedure from enyne **28b** (39.1 mg, 0.20 mmol); colorless oil (25.6 mg, 65%). ^{1}H NMR (400 MHz, C_6D_6) mixture of diastereomers (~3:1) δ 4.84 (m, 2H), 4.83 (m, 0.3H), 4.74 (m, 0.3H), 3.99 – 3.85 (m, 1.3H), 3.77 – 3.64 (m, 1.3H), 3.11 (dd, *J* = 12.6, 6.3 Hz, 0.3H), 2.66 (dd, *J* = 13.2, 12.0 Hz, 1H), 2.46 (dd, *J* = 11.9, 7.9 Hz, 1H), 2.13 (t, *J* =12.4 Hz, 0.3H), 2.04 – 1.83 (m, 3.9H), 1.63 (s, 3H), 1.61 – 1.59 (m, 0.9H), 1.69 – 1.46 (m, 2.6H), 1.51 (s, 0.9H), 1.31 (s, 3H), 1.29 (s, 3H), 1.03 (s, 0.9H). ^{13}C NMR (101 MHz, C_6D_6) major δ 143.1, 113.2, 111.8, 82.9, 67.0, 55.9, 40.5, 38.5, 29.7, 25.0, 23.7, 23.0; minor δ 143.6, 113.0, 112.3, 82.5, 66.7, 54.3, 39.9, 35.6, 31.3, 24.8, 24.6, 23.4. IR (film) ṽ 2972, 2880, 1454, 1367, 1180 cm^{-1}. HRMS (CI$^+$) for $C_{21}H_{29}NO_4S$ [M+H]$^+$: calcd 197.1536, found 197.1535.

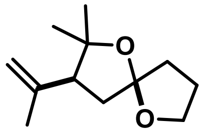

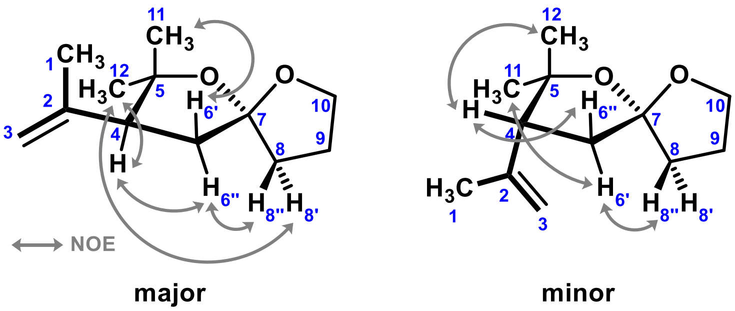

Table S12. Detailed NMR analysis of the major diastereomer of product **31b** (C_6D_6, 25 °C, 600 MHz)

Atom [#] C	H	δ [ppm]	COSY	HSQC	HMBC	NOESY
**1**		22.98		1	3, 4	
	**1**	1.63	3, 4	1	2, 3, 4, 5	4, 6', 11, 12
**2**		143			1, 3, 4, 6'	
**3**		113.12		3	1, 4	
	**3**	4.84	1, 4	3	1, 2, 4, 5	4, 6'
**4**		55.89		4	1, 3, 6', 6'', 11, 12	
	**4**	2.46	1, 3, 6', 6''	4	1, 2, 3, 5, 6, 11, 12	1, 3, 6'', 12
**5**		82.8			1, 3, 4, 6'', 11, 12	
**6**		40.45		6', 6''	4, 8''	
	**6'**	2.65	4, 6''	6	2, 4, 7, 8	1, 3, 11
	**6''**	1.98	4, 6'	6	4, 5, 8	4, 8''
**7**		112.96			6', 8', 9', 10', 10''	
**8**		38.47		8', 8''	6', 6'', 9', 9'', 10', 10''	
	**8'**	1.95	8'', 9', 9''	8	7, 9, 10	12
	**8''**	1.55	8', 9', 9''	8	6, 9, 10	6''
**9**		24.98		9', 9''	8', 8'', 10', 10''	
	**9'**	1.89	8', 8'', 9'', 10', 10''	9	7, 8, 10	
	**9''**	1.55	8', 8'', 9', 10', 10''	9	8, 10	
**10**		66.95		10', 10''	8', 8'', 9', 9''	
	**10'**	3.9	9', 9'', 10''	10	7, 8, 9	11
	**10''**	3.72	9', 9'', 10'	10	7, 8, 9	
**11**		23.59		11	4, 12	
	**11**	1.28		11	4, 5, 12	1, 6', 10', 12
**12**		29.64		12	4, 11	
	**12**	1.31		12	4, 5, 11	1, 4, 8', 11

2,2-Dimethoxy-4-(prop-1-en-2-yl)tetrahydrofuran (32). Prepared analogously from enyne **29** (70.0 mg, 0.41 mmol) as a colorless oil (53.8 mg, 76%). ^{1}H NMR (400 MHz, $CDCl_3$) δ 4.79 – 4.77 (m, 1H), 4.77 – 4.74 (m, 1H), 4.06 (t, J = 8.0 Hz, 1H), 3.74 (dd, J = 9.4, 8.2 Hz, 1H), 3.31 (s, 3H), 3.28 (s, 3H), 3.12 – 3.01 (m, 1H), 2.26 – 2.18 (m, 1H), 1.92 (dd, J = 12.7, 10.5 Hz, 1H), 1.75 – 1.69 (m, 3H). ^{13}C NMR (101 MHz, $CDCl_3$) δ 143.7, 122.8, 111.2, 70.9, 50.4, 49.6, 44.8, 37.3, 20.7. IR (film) ṽ 2960, 2837, 1438, 1259, 1150, 1099, 1042, 1006, 793 cm^{-1}.

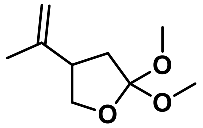

Methyl-4-(prop-1-en-2-yl)dihydrofuran-2(3*H*)-one (33). *p*-Toluenesulfonic acid monohydrate (3.1 mg, 0.016 mmol) was added to a stirred solution of ortholactone **32** (28.0 mg, 0.16 mmol) in acetone (1.0 mL) in a flame dried Schlenk tube under argon. The mixture was vigorously stirred at 25°C for 4 h before it was concentrated. The residue was purified by flash chromatography (silica, CH_2Cl_2) to give the title compound as a colorless oil (16.9 mg, 82%). ^{1}H NMR (400 MHz, $CDCl_3$) δ 4.89 (dd, J = 2.0, 1.2 Hz, 1H), 4.83 (q, J = 1.0 Hz, 1H), 4.45 (dd, J = 9.0, 7.6 Hz, 1H), 4.11 (dd, J = 9.0, 7.6 Hz, 1H), 3.20 (m, 1H), 2.65 (dd, J = 17.3, 8.5 Hz, 1H), 2.47 (dd, J = 17.4, 8.8 Hz, 1H), 1.77 (t, J = 1.2 Hz, 3H). ^{13}C NMR (101 MHz, $CDCl_3$) δ 176.8, 142.4, 112.4, 71.8, 42.5, 3.2, 20.4. IR (film) ṽ 2991, 2916, 1760, 1485, 1461, 1423, 1376, 1280, 1240, 1159, 1033, 989, 930, 869, 800, 675, 491 cm^{-1}.

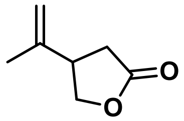

(2R*,4R*)-2-Cyclohexyl-2-methoxy-4-(prop-1-en-2-yl)tetrahydrofuran (34). Prepared analogously from enyne **S42** (30.7 mg, 0.14 mmol); colorless and very acid-sensitive oil (21.2mg, 68 %).^{1}H NMR (400 MHz, C_6D_6) δ 4.8 (dt, J = 2.0, 1.0 Hz, 1H), 4.7 (qd, J = 1.9, 1.2 Hz, 1H), 3.9 (t, J = 7.8 Hz, 1H), 3.7 (dd, J = 10.6, 8.0 Hz, 1H), 3.2 (s, 3H), 2.7 – 2.6 (m, 1H), 2.0 (dd, J = 13.3, 9.8 Hz, 1H), 2.0 – 1.8 (m, 3H), 1.8 (dd, J = 13.3, 9.3 Hz, 1H), 1.8 – 1.7 (m, 2H), 1.7 – 1.6 (m, 4H), 1.2 – 0.8 (m, 5H). ^{13}C NMR (101 MHz, C_6D_6) δ 144.4, 112.2, 111.1, 70.6, 47.5, 47.2, 41.4, 37.7, 28.1, 28.0, 26.9, 26.7, 26.7, 20.6. IR (film) ṽ 2923, 2853, 1647, 1450, 1302, 1094, 1046 cm^{-1}. HRMS (ESI$^+$) for $C_{14}H_{24}O_2Na$ [M+Na]$^+$: calcd 247.1668, found 247.1668.

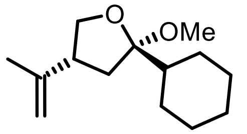

(1R*,4R*,5R*)-1-Cyclohexyl-5-(prop-1-en-2-yl)-2,7-dioxabicyclo[2.2.1]heptane (35). Prepared analogously from enyne **S43** (22.0 mg, 0.10 mmol); colorless, acid sensitive oil (11.0 mg, 49 %). ^{1}H NMR (400 MHz, C_6D_6) δ 4.8 (ddt, J = 6.2, 2.3, 1.4 Hz, 2H), 4.3 (dd, J = 4.6, 3.6 Hz, 1H), 3.7 (d, J = 6.5 Hz, 1H), 3.4 (ddd, J = 6.5, 3.6, 1.9 Hz, 1H), 2.6 (ddddd, J = 10.7, 5.6, 3.7, 1.9, 0.9 Hz, 1H), 2.2 (dtq, J = 10.3, 3.3, 1.6 Hz, 1H), 2.1 – 1.9 (m, 2H), 1.8 (dd, J = 12.1, 6.0 Hz, 1H), 1.8 – 1.7 (m, 3H), 1.6 – 1.5 (m, 1H), 1.4 – 1.3 (m, 5H), 1.3 – 1.1 (m, 3H). ^{13}C NMR (101 MHz, C_6D_6) δ 142.9, 113.4, 111.6, 78.3, 65.6, 47.9, 41.6, 36.0, 28.3, 27.8, 26.8, 26.6, 26.5, 23.4. IR (film) ṽ 3085, 2928, 1649, 1450, 1327, 1020 cm^{-1}. HRMS (ESI$^+$) for $C_{14}H_{23}O_2$ [M+H]$^+$: calcd 223.1693, found 223.1695.

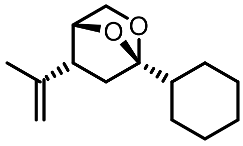

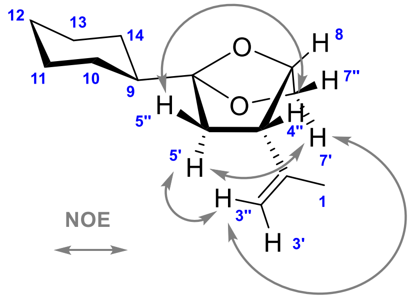

Table S9. Detailed NMR analysis of bridged bicyclic compound **35** ($CDCl_3$, 25 °C, 600 MHz)

Atom[#]		δ [ppm]	J [Hz]	COSY	HSQC	HMBC	NOESY
C	H						
**1**		**23.38**			1	3', 3''	
	**1**	**1.40**	1.60, 0.90	3', 3'', 4	1	2, 3, 4	3', 4, 7', 8
**2**		**142.85**				1, 3'', 4, 5', 5''	
**3**		**111.57**			3', 3''	1, 4	
	**3'**	**4.84**	1.50	1, 4	3	1, 4	1, 3''
	**3''**	**4.83**		1, 4	3	1, 2, 4, 8	3', 5', 7', 8
**4**		**47.85**			4	1, 3', 3'', 5', 5'', 7', 7'', 8	
	**4**	**2.62**	10.70, 6.80, 4.70, 1.90, 0.90	1, 3', 3'', 5', 5'', 7'', 8	4	2, 3, 5, 7, 8	1, 5'', 8
**5**		**35.91**			5', 5''	4, 8, 9	
	**5'**	**1.84**	12.00, 6.00	4, 8	5	2, 4, 6, 8	3'', 7', 9, 10eq, 14ax
	**5''**	**1.70**	11.90, 10.90, 0.70	4, 8	5	2, 4, 6, 8	4, 9, 10ax, 10eq, 14ax, 14eq
**6**		**113.33**				5', 5'', 7', 8, 9, 10ax, 10eq, 14ax	
**7**		**65.58**			7', 7''	4, 8	
	**7'**	**3.73**	6.50	7''	7	4, 6, 8	1, 3'', 5', 7'', 8
	**7''**	**3.40**	6.50, 3.60, 2.00	4, 7', 8	7	4, 8	7', 8
**8**		**78.23**			8	3'', 4, 5', 5'', 7', 7''	
	**8**	**4.35**		4, 5', 5'', 7''	8	4, 5, 6, 7	1, 3'', 4, 7', 7''
**9**		**41.50**			9	10ax, 11ax, 11eq, 13ax, 13eq, 14ax	
	**9**	**1.99**	12.00, 3.20	10ax, 10eq, 14ax, 14eq	9	5, 6, 10, 11, 13, 14	5', 5'', 10eq, 11ax, 13ax, 14eq
**10**		**28.23**			10ax, 10eq	9, 11ax, 11eq, 12ax, 12eq, 14ax, 14eq	
	**10ax**	**1.37**		9, 10eq, 11ax, 11eq	10	6, 9, 11, 12, 14	5'', 10eq, 11eq, 12ax, 14ax
	**10eq**	**2.03**		9, 10ax, 11ax, 11eq	10	6, 11, 12, 14	5', 5'', 9, 10ax, 11ax, 11eq
**11**		**26.54**			11eq	9, 10ax, 10eq, 12ax, 12eq, 13ax, 13eq	
	**11ax**	**1.20**		10ax, 10eq, 11eq, 12ax, 12eq		9, 10, 12, 13	9, 10eq, 12eq, 13ax

**11eq**	**1.73**		10ax, 10eq, 11ax, 12ax, 12eq	11	9, 10, 12, 13	10ax, 10eq, 12eq
**12**	**26.73**				10ax, 10eq, 11ax, 11eq, 13ax, 13eq, 14ax, 14eq	
**12ax**	**1.14**		11ax, 11eq, 12eq, 13ax, 13eq		10, 11, 13, 14	10ax, 12eq, 13eq, 14ax
**12eq**	**1.60**		11ax, 11eq, 12ax, 13ax, 13eq		10, 11, 13, 14	11ax, 11eq, 12ax, 13ax, 13eq
**13**	**26.40**			13ax, 13eq	9, 11ax, 11eq, 12ax, 12eq, 14ax, 14eq	
**13ax**	**1.22**		12ax, 12eq, 13eq, 14ax, 14eq	13	9, 11, 12, 14	9, 11ax, 12eq, 13eq, 14eq
**13eq**	**1.74**		12ax, 12eq, 13ax, 14ax, 14eq	13	9, 11, 12, 14	12ax, 12eq, 13ax, 14ax, 14eq
**14**	**27.77**			14ax, 14eq	9, 10ax, 10eq, 12ax, 12eq, 13ax, 13eq	
**14ax**	**1.39**	12.50, 3.40	9, 13ax, 13eq, 14eq	14	6, 9, 10, 12, 13	5', 5'', 10ax, 12ax, 13eq, 14eq
**14eq**	**2.19**		9, 13ax, 13eq, 14ax	14	10, 12, 13	5'', 9, 13ax, 13eq, 14ax

(4*S,5*S**)-4-(Cyclohex-1-en-1-yl)-2,2-dimethyl-1,6-dioxaspiro[4.5]decane (36a) and (4*S**,5*R**)-4-(cyclohex-1-en-1-yl)-2,2-dimethyl-1,6-dioxaspiro[4.5]decane (36b)** Prepared analogously from enyne **S44** (30.7 mg, 0.090 mmol); flash chromatography (silica, hexane:*tert*-butyl methyl ether, 98:2) allowed the diastereoisomers to be isolated in analytically pure form.

36a: Colorless oil (8.7 mg, 31 %). ^{1}H NMR (400 MHz, C_6D_6) δ 5.6 (tt, *J* = 3.7, 1.4 Hz, 1H), 4.0 (ddd, *J* = 12.6, 10.9, 2.5 Hz, 1H), 3.5 (ddt, *J* = 11.1, 5.0, 1.8 Hz, 1H), 2.5 – 2.4 (m, 2H), 2.3 (dd, *J* = 13.0, 11.4 Hz, 1H), 2.1 – 1.9 (m, 4H), 1.8 (td, *J* = 12.9, 4.5 Hz, 1H), 1.7 – 1.4 (m, 8H), 1.4 – 1.4 (m, 4H), 1.2 (s, 3H). ^{13}C NMR (101 MHz, C_6D_6) δ 135.5, 125.1, 106.4, 79.4, 60.7, 57.2, 40.8, 33.3, 30.9, 29.7, 27.6, 26.1, 26.0, 23.7, 23.1, 20.7. IR (film) ṽ 2927, 2873, 1449, 1364, 1155, 1072 cm^{-1}. HRMS (CI^{+}) for $C_{16}H_{26}O_2$ $[M]^{+}$: calcd 250.1927, found 250.1927.

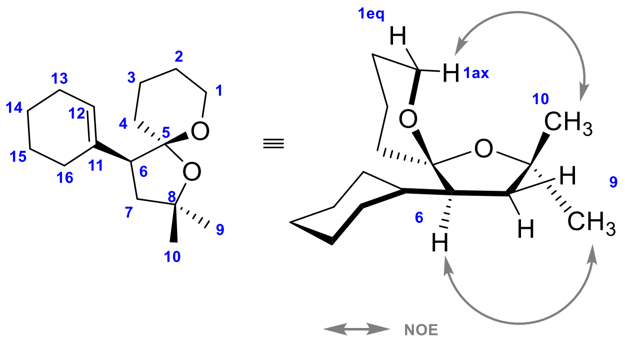

Table S10. Detailed NMR analysis of compound **36a** (C_6D_6, 25 °C, 500 MHz).

Atom C H	δ [ppm]	J [Hz]	COSY	HSQC	HMBC	NOESY
**1**	**60.74**			1ax, 1eq	2ax, 3ax	
**1ax**	**3.99**	12.70, 10.90, 2.50	1eq, 2ax, 2eq	1		1eq, 2eq, 3ax, 10
**1eq**	**3.55**	11.10, 5.10, 1.70	1ax, 2ax, 2eq	1	2, 3, 5	1ax, 2ax, 2eq
**2**	**25.99**			2ax, 2eq	1eq, 3ax, 3eq, 4ax, 4eq	
**2ax**	**1.42**		1ax, 1eq, 2eq, 3ax, 3eq	2	1, 3	1eq, 4ax
**2eq**	**1.23**		1ax, 1eq, 2ax, 3ax, 3eq	2		1ax, 1eq
**3**	**20.70**			3ax, 3eq	1eq, 2ax, 4ax	
**3ax**	**1.99**		2ax, 2eq, 3eq, 4ax, 4eq	3	1, 2, 4	1ax
**3eq**	**1.51**		2ax, 2eq, 3ax, 4ax, 4eq	3	2	
**4**	**33.27**			4ax, 4eq	3ax, 6	
**4ax**	**1.85**		3ax, 3eq, 4eq	4	2, 3, 5	2ax, 12
**4eq**	**1.48**		3ax, 3eq, 4ax	4	2	6
**5**	**106.38**				1eq, 4ax, 6, 7''	
**6**	**57.21**			6	7', 7'', 12, 16'	
**6**	**2.50**	7.40, 12.90	7', 7''	6	4, 5, 7, 11, 12, 16	4eq, 7'', 9, 12
**7**	**40.76**			7', 7''	6, 9, 10	
**7'**	**2.31**	13.00, 11.50, 0.50	6, 7'', 9	7	6, 8, 9, 10, 11	7'', 10, 12, 16''
**7''**	**1.61**	11.40, 7.40	6, 7', 9	7	5, 6, 9	6, 7', 9, 10
**8**	**79.35**				7', 9, 10	
**9**	**29.72**			9	7', 7'', 10	
**9**	**1.21**		7', 7''	9	7, 8, 10	6, 7''
**10**	**30.93**			10	7', 9	
**10**	**1.41**			10	7, 8, 9	1ax, 7', 7''
**11**	**135.52**				6, 7', 15, 16', 16''	
**12**	**125.14**			12	6, 14', 14'', 16', 16''	
**12**	**5.60**		13	12	6, 13, 14, 16	4ax, 6, 7', 13
**13**	**26.07**			13	12, 14', 14'', 15	

Atom C	Atom H	δ [ppm]	J [Hz]	COSY	HSQC	HMBC	NOESY
	13	2.05		12, 14', 14''	13		12
14		23.10			14', 14''	12, 15	
	14'	1.60		13, 14'', 15	14	12, 13, 15, 16	
	14''	1.53		13, 14', 15	14	12, 13, 15, 16	
15		23.70			15	14', 14''	
	15	1.66		14', 14'', 16', 16''	15	11, 13, 14, 16	16', 16''
16		27.59			16', 16''	6, 12, 14', 14'', 15	
	16'	2.47		15, 16''	16	6, 11, 12	15
	16''	2.04		15, 16'	16	11, 12	7', 15

36b: Colorless oil (7.2 mg, 27 %). ^{1}H NMR (400 MHz, C_6D_6) δ 5.5 (dq, *J* = 3.8, 1.8 Hz, 1H), 4.1 (ddd, *J* = 12.5, 10.9, 2.5 Hz, 1H), 3.7 – 3.6 (m, 1H), 3.1 (t, *J* = 8.6 Hz, -OH), 2.3 – 2.2 (m, 1H), 2.0 – 1.9 (m, 4H), 1.8 (dd, *J* = 9.1, 2.4 Hz, 2H), 1.6 – 1.4 (m, 8H), 1.4 (s, 3H), 1.3 (s, 3H), 1.3 (dtt, *J* = 13.1, 2.9, 1.7 Hz, 1H). ^{13}C NMR (101 MHz, C_6D_6) δ 136.1, 123.1, 107.7, 79.8, 62.6, 57.0, 41.5, 32.9, 30.2, 29.3, 29.1, 26.1, 25.8, 23.5, 22.9, 20.1. IR (film) ṽ 2923, 2874, 1448, 1364, 11395, 1078 cm^{-1}. HRMS (CI$^+$) for $C_{16}H_{26}O_2$ [M]$^+$: calcd 250.1927, found 250.1926.

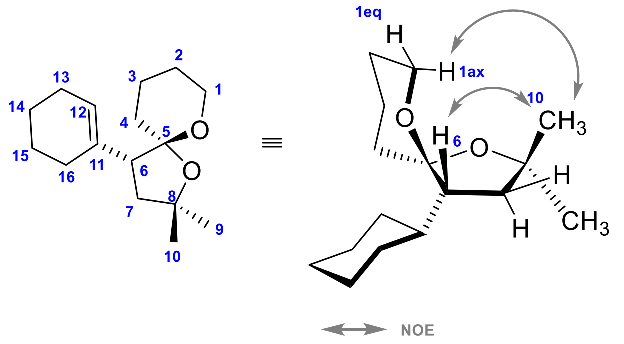

Table S11. Detailed NMR analysis of compound **36b** (C_6D_6, 25 °C, 500 MHz).

Atom C	Atom H	δ [ppm]	J [Hz]	COSY	HSQC	HMBC	NOESY
1		62.57			1ax, 1eq	2ax	
	1ax	4.10	12.60 11.00, 2.50	1eq, 2ax, 2eq	1		1eq, 2eq, 3ax, 10
	1eq	3.65	11.00, 4.80	1ax, 2ax	1	2, 3, 5	1ax, 2ax, 2eq
2		26.11			2ax, 2eq	1eq, 3ax, 3eq, 4	
	2ax	1.49		1ax, 1eq, 2eq, 3ax, 3eq	2	1, 3, 4	1eq, 2eq

**2eq**	**1.26**	13.00	1ax, 2ax, 3ax, 3eq	2		1ax, 1eq, 2ax, 3ax, 3eq
**3**	**20.08**			3ax, 3eq	1eq, 2ax, 4	
**3ax**	**1.98**		2ax, 2eq, 3eq	3	2, 4	1ax, 2eq
**3eq**	**1.51**		2ax, 2eq, 3ax	3	2, 5	2eq
**4**	**32.92**			4	2ax, 3ax	
**4**	**1.49**			4	2, 3	
**5**	**107.73**				1eq, 3eq, 6, 7	
**6**	**57.01**			6	7, 12	
**6**	**3.07**	9.50, 8.50, 1.10	7	6	5, 7, 11, 12, 16	7, 10, 12, 16', 16''
**7**	**41.52**			7	6, 9, 10	
**7**	**1.80**		6	7	5, 6, 8, 9, 10, 11	6, 9, 10, 12
**8**	**79.82**				7, 9, 10	
**9**	**30.17**			9	7, 10	
**9**	**1.32**			9	7, 8, 10	7, 10, 16', 16''
**10**	**29.29**			10	7, 9	
**13**	**1.41**			10	7, 8, 9	1ax, 6, 7, 9
**11**	**136.14**				6, 7, 15	
**12**	**123.08**			12	6, 14	
**12**	**5.50**		13	12	6, 13, 14, 16	6, 7, 13
**13**	**25.79**			13	12, 14, 15	
**13**	**1.98**		12, 14	13		12
**14**	**22.93**			14	12, 15	
**14**	**1.50**		13, 15	14	12, 13, 15, 16	
**15**	**23.46**			15	14	
**15**	**1.57**		14, 16', 16''	15	11, 13, 14, 16	
**16**	**29.12**			16', 16''	6, 12, 14, 15	
**16'**	**2.24**		15, 16''	16		6, 9
**16''**	**1.96**		15, 16'	16		6, 9

(1S*,5S*,7S*)-7-(Prop-1-en-2-yl)-5-((trimethylsil)oxy)-2-oxabicyclo[3.2.1]octane (40). Prepared analogously from enyne **39** (23.8 mg, 0.1 mmol); colorless oil (16.9 mg, 70 %). ^{1}H NMR (400 MHz, $CDCl_3$) δ 4.9 (ddd, J = 2.4, 1.7, 0.9 Hz, 1H), 4.9 (dp, J = 3.0, 1.5 Hz, 1H), 4.4 (ddd, J = 4.4, 3.1, 1.3 Hz, 1H), 3.9 – 3.6 (m, 2H), 2.8 – 2.7 (m, 1H), 2.1 – 2.0 (m, 2H), 2.0 – 1.8 (m, 5H), 1.8 – 1.7 (m, 1H), 1.6 – 1.5 (m, 1H), 0.1 (s, 9H). ^{13}C NMR (101 MHz, $CDCl_3$) δ 144.3, 110.0, 79.5, 77.3, 61.9, 48.3, 47.4, 41.8, 38.9, 22.8, 2.5. IR (film) ṽ 2956, 2888, 1644, 1447, 1333, 1249, 1161 cm^{-1}. HRMS (CI$^+$) for $C_{13}H_{24}O_2Si$ [M+H]$^+$: calcd 240.1540, found 240.1539.

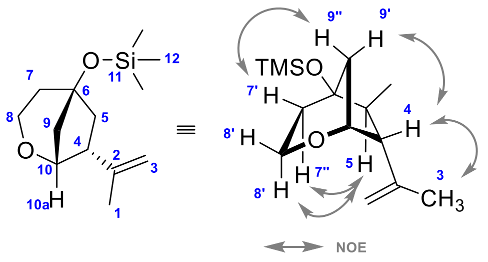

Table S13. Detailed NMR analysis of the oxabicyclo[3.2.1]octane **40** ($CDCl_3$, 25 °C, 500 MHz)

Atom C	Atom H	δ (ppm)	J	COSY	HSQC	HMBC	NOESY
1		22.61			1	3', 3''	
	1	1.84		3', 3''	1	2, 3, 4	3'', 4, 10a
2		144.12				1, 5	
3		109.89			3', 3''	1	
	3'	4.94		1, 4	3	1, 4	5
	3''	4.91	2.70, 1.50	1, 4	3	1, 4	1
4		48.14			4	1, 3', 3'', 5, 9''	
	4	2.73		3', 3'', 5, 10a	4		1, 5, 9', 10a
5		38.75			5	7', 9'', 10a	
	5	2.02		4	5	2, 4, 6, 7, 9, 10	3', 4, 7'', 8', 12
6		79.32				5, 7', 7'', 8'', 9', 9'', 10a	
7		41.65			7', 7''	5, 8', 8'', 9', 9''	
	7'	1.95	12.00, 7.00, 1.20	7'', 8', 8''	7	5, 6, 8, 9	8'', 9''
	7''	1.60	11.70, 3.30	7', 8', 8''	7	6, 9	5, 8', 8''
8		61.74			8', 8''	7', 10a	
	8'	3.75	12.00, 4.10	7', 7'', 8''	8	7	5, 7''
	8''	3.7	11.90, 7.00, 1.00	7', 7'', 8'	8	6, 7, 10	7', 7''
9		47.28			9', 9''	5, 7', 7''	
	9'	1.87	11.00, 3.20	9'', 10a	9	6, 7	4, 10a
	9''	1.75	11.00, 1.30	9'	9	4, 5, 6, 7, 10	7', 10a
10		77.11			10a	5, 8'', 9''	
	10a	4.35		4, 9'	10	5, 6, 8	1, 4, 9', 9''
12		2.35			12		
	12	0.15			12		5
**Atom Si**		**δ (ppm)**	**J**	**COSY**	**HSQC**	**HMBC**	**NOESY**
11		11.09					

3-(Prop-1-en-2-yl)-1,7-dioxaspiro[4.4]nonane (41). Prepared analogously from enyne **S33** (29.2, 0.18 mmol); colorless oil (19.9 mg, 68 %, mixture of diastereoisomers ~ 1.2:1). ^{1}H NMR (400 MHz, $CDCl_3$) δ 4.8 – 4.7 (m, 2H), 4.1 – 3.9 (m, 3H), 3.9 – 3.7 (m, 1H), 3.7 – 3.5 (m, 2H), 3.0 – 2.8 (m, 1H), 2.2 – 2.0 (m, 2H), 2.0 – 1.8 (m, 2H), 1.8 – 1.7 (m, 3H). ^{13}C NMR (101 MHz, $CDCl_3$) major δ 144.3, 110.7, 89.7, 77.8, 71.2, 67.9, 46.6, 38.9, 21.1; minor ^{13}C NMR (101 MHz, $CDCl_3$) δ 144.2, 110.7, 89.7, 77.0, 71.2, 68.2, 46.4, 39.4, 38.2, 21.1. IR (film) ṽ 2969, 2938, 2862, 1647 1438, 1055, 889 cm^{-1}. HRMS (CI$^+$) for $C_{10}H_{16}O_2$ [M]$^+$: calcd 168.1145, found 168.1145.

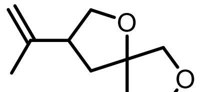

***tert*-Butyl 3-(prop-1-en-2-yl)-1-oxa-7-azaspiro[4.4]nonane-7-carboxylate (42).** Prepared analogously from enyne **S32** (26:0 mg, 0.098 mmol); colorless oil (20.0 mg, 77% (contains 5% of an unknown impurity), mixture of diastereoisomers 1:1). ^{1}H NMR (400 MHz, $CDCl_3$) δ 4.9 – 4.7 (m, 2H), 4.0 – 3.9 (m, 1H), 3.6 (dt, *J* = 11.4, 8.7 Hz, 1H), 3.5 – 3.3 (m, 3H), 3.3 – 3.1 (m, 1H), 2.9 (p, *J* = 8.2 Hz, 1H), 2.1 – 1.9 (m, 2H), 1.9 – 1.7 (m, 2H), 1.7 (s, 3H), 1.4 (s, 9H). Analysis is further complicated by the fact that the signals in the ^{13}C NMR spectra of each diastereoisomer are split (ca. 1:1) due to the presence of rotamers of the Boc group.

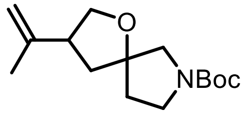

Repeated flash chromatography furnished a pure sample of one diastereoisomers, whereas the second isomer is contaiminated by the mentioned impurity of unknown composition, see copies of Spectra provided below

Isomer 42a: ^{1}H NMR (400 MHz, $CDCl_3$) δ 4.8 – 4.7 (m, 2H), 4.0 (t, *J* = 8.1 Hz, 1H), 3.7 – 3.6 (m, 1H), 3.5 – 3.3 (m, 2H), 3.3 – 3.2 (m, 1H), 2.9 (p, *J* = 8.4 Hz, 1H), 2.2 – 2.0 (m, 2H), 2.0 – 1.8 (m, 2H), 1.7 (s, 3H), 1.5 (s, 9H). ^{13}C NMR (101 MHz, $CDCl_3$) rotamer mixture δ 154.7, 154.7, 110.9, 110.8, 88.5, 87.7, 79.3, 71.1, 56.9, 56.2, 46.5, 46.4, 45.2, 44.8, 39.1, 38.9, 37.2, 36.6, 28.7, 28.5, 21.1. IR (film) ṽ 2973, 2931, 1964, 1402, 1153, 1096 cm^{-1}.

Isomer 42b: ^{1}H NMR (400 MHz, $CDCl_3$) δ 4.7 – 4.7 (m, 2H), 3.9 (t, *J* = 8.0 Hz, 1H), 3.6 – 3.5 (m, 1H), 3.5 – 3.3 (m, 3H), 3.2 (d, *J* = 11.5 Hz, 1H), 2.9 (p, *J* = 8.6 Hz, 1H), 2.1 – 1.9 (m, 2H), 1.9 – 1.7 (m, 2H), 1.7 – 1.6 (m, 3H), 1.4 (s, 9H). ^{13}C NMR (101 MHz, $CDCl_3$) δ 154.6, 144.8, 110.9, 110.8, 88.1, 79.3, 71.2, 56.5, 56.1, 46.3, 45.3, 44.9, 38.6, 38.3, 37.6, 36.8, 29.9, 28.7, 28.5, 21.1.

(1*S,5*S**,7*S**)-7-(Prop-1-en-2-yl)-2-tosyl-5-((trimethylsilyl)oxy)-2-azabicyclo[3.2.1]octane (43)**. Prepared analogously from enyne **S45** (38.8 mg, 0.099 mmol) but using 4 mol% of $[Cp^*RuCl]_4$ (30.0 mg, 77 %).^{1}H NMR (400 MHz, $CDCl_3$) δ 7.7 – 7.7 (m, 2H), 7.3 – 7.3 (m, 2H), 4.9 (dt, *J* = 2.5, 1.3 Hz, 1H), 4.9 (p, *J* = 1.1 Hz, 1H), 4.5 (dt, *J* = 5.1, 2.7 Hz, 1H), 3.8 (ddt, *J* = 15.0, 5.6, 1.9 Hz, 1H), 3.2 (ddd, *J* = 15.0, 11.7, 5.8 Hz, 1H), 2.9 – 2.8 (m, 1H), 2.4 (s, 3H), 2.0 (ddd, *J* = 13.3, 6.9, 1.8 Hz, 1H), 1.9 – 1.8 (m, 4H), 1.7 (ddd, *J* = 11.1, 3.8, 2.4 Hz, 1H), 1.5 – 1.3 (m, 2H), 1.2 – 1.1 (m, 1H), 0.0 (s, 9H). ^{13}C NMR (101 MHz, $CDCl_3$) δ 143.3, 143.1, 138.5, 129.9, 127.1, 110.7, 79.5, 58.0, 48.2, 44.5, 41.5, 38.6, 37.4, 22.7, 21.6, 2.3. IR (film) ṽ 2958, 2867, 1697, 1333, 1250, 11154, 1092 cm^{-1}. HRMS (ESI$^+$) for $C_{20}H_{31}NO_3SiNa$ [M+Na]$^+$: calcd 416.1686, found 416.1855.

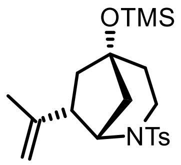

6-(Prop-1-en-2-yl)-4-((trimethylsilyl)oxy)-2-oxabicyclo[2.2.1]heptane (44). Prepared analogously from enyne **S46** (27.7 mg, 0.12 mmol); colorless oil (14.0 mg, 51 %). ^{1}H NMR (400 MHz, $CDCl_3$) δ 4.9 (p, *J* = 1.5 Hz, 1H), 4.9 (dq, *J* = 1.8, 0.9 Hz, 1H), 4.2 – 4.2 (m, 1H), 3.6 (dd, *J* = 6.1, 3.6 Hz, 1H), 3.5 (dd, *J* = 6.6, 1.3 Hz, 1H), 2.6 – 2.6 (m, 1H), 2.0 – 1.9 (m, 2H), 1.9 – 1.7 (m, 4H), 1.3 – 1.2 (m, 1H), 0.2 (d, *J* = 0.7 Hz, 9H). ^{13}C NMR (101 MHz, $CDCl_3$) δ 144.9, 110.6, 82.0, 77.6, 75.5, 49.4, 44.1, 37.3, 22.3, 1.9. IR (film) ṽ 2957, 2872, 1648, 1442, 1308, 1251, 1172, 1021 cm^{-1}. HRMS (ESI$^+$) for $C_{12}H_{22}O_2SiNa$ [M+Na]$^+$: calcd 249.1281, found 249.1281.

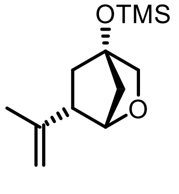

***tert*-Butyl 4-hydroxy-6-(prop-1-en-2-yl)-2-azabicyclo[2.2.1]heptane-2-carboxylate (45).** [Cp*RuCl]$_4$ (5.4 mg, 4 mol%) was added to a stirred solution of enyne **S47** (40.8 mg, 0.13 mmol) in 1,2-dichloroethane (1.6 mL, 0.1 M) in a flame-dried Schlenk tube under argon. H_2 was bubbled through the mixture for 2 min before the flask was immersed into a pre-heated oil bath at 70 °C keeping a static H_2 atmosphere (ambient pressure, balloon filled with H_2). After stirring for 3 h at 70 °C, the mixture was cooled to room temperature and then filtered hrough a plug of Florisil, eluting with Et_2O (10 mL). The filtrate was evaporated and the residue re-dissolved in THF (2.0 mL),

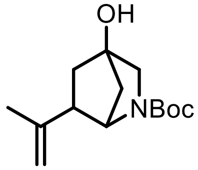

TBAF·3H_2O (79.5 mg, 0.25 mmol) was added to this solution of the crude material and the mixture was stirred at or 2h before it was poured in water. The aqueous phase was extracted with Et_2O (3 x 5 mL), the combined organic layers were dried over $MgSO_4$ and evaporated, and the residue was purified by flash chromatography (silica, Hex/EtOAc 6:4) to yield the title product as a colorless oil (25.9 mg, 81%). ^{1}H NMR (400 MHz, $CDCl_3$, rotamers) δ 4.9 (q, *J* = 1.2 Hz, 1H), 4.8 – 4.8 (m, 1H), 4.1 (dt, *J* = 2.8, 1.3 Hz, 1H), 3.3 (dd, *J* = 8.8, 3.4 Hz, 1H), 3.1 (dd, *J* = 8.8, 1.4 Hz, 1H), 2.7 (ddd, *J* = 11.4, 5.2, 2.5 Hz, 2H), 1.9 – 1.8 (m, 2H), 1.8 (t, *J* = 0.9 Hz, 3H), 1.8 – 1.7 (m, 2H), 1.4 (s, 9H); minor δ 4.8 (p, *J* = 1.4 Hz, 1H), 4.8 – 4.7 (m, 1H), 4.3 (q, *J* = 2.1 Hz, 1H), 3.2 (dd, *J* = 8.9, 3.4 Hz, 1H), 3.1 (dd, *J* = 8.9, 1.3 Hz, 1H), 2.8 – 2.7 (m, 1H), 1.9 – 1.8 (m, 2H), 1.8 – 1.8 (m, 3H), 1.8 – 1.7 (m, 3H), 1.4 (s, 9H). ^{13}C NMR (101 MHz, $CDCl_3$, major rotamer): δ 154.3, 143.6, 110.7, 80.0, 79.5, 58.2, 55.9, 49.9, 45.1, 37.1, 28.4, 23.8; minor: δ 154.1, 144.7, 109.9, 80.4, 79.1, 57.6, 56.5, 49.7, 44.7, 37.1, 28.5, 23.3. IR (film) ṽ 3392 (br), 2975, 2880, 1666, 1411, 1239, 1117, 1085 cm^{-1}. HRMS (ESI$^+$) for $C_{14}H_{23}NO_3Na$ [M+Na]$^+$: calcd 276.1570, found 276.1570.

2,2-Dimethyl-4-(prop-1-en-2-yl)-1-tosylpyrrolidine (47). Prepared analogously from enyne **46** (56.9 mg, 0.195 mmol); white solid (52.1 mg, 90 %). ^{1}H NMR (400 MHz, $CDCl_3$) δ 7.7 – 7.6 (m, 2H), 7.2 (dt, *J* = 6.7, 0.8 Hz, 2H), 4.7 (h, *J* = 1.5 Hz, 1H), 4.6 (q, *J* = 1.3 Hz, 1H), 3.6 (ddd, *J* = 8.9, 7.2, 1.4 Hz, 1H), 3.0 – 2.9 (m, 1H), 2.7 (tt, *J* = 12.0, 6.6 Hz, 1H), 2.3 (s, 3H), 1.8 (ddd, *J* = 12.1, 6.2, 1.3 Hz, 1H), 1.7 – 1.6 (m, 4H), 1.4 (d, *J* = 5.8 Hz, 6H). ^{13}C NMR (101 MHz, $CDCl_3$) δ 143.8, 142.7, 138.6, 129.5, 127.2, 110.8, 65.6, 53.0, 47.5, 42.0, 29.1, 28.6, 21.6, 21.4. IR (film) ṽ 2968, 2871, 1648, 1599, 1334, 1150, 1098 cm^{-1}. HRMS (CI$^+$) for $C_{16}H_{23}NO_2S$ [M+H]$^+$: calcd 293.1444, found 293.1444.

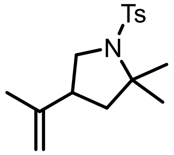

***tert*-Butyl 2,2-dimethyl-4-(prop-1-en-2-yl)pyrrolidine-1-carboxylate (48).** Prepared according to the Representative Procedure from enyne **S48** (30.8 mg, 0.13 mmol); colorless oil (17.2 mg, 55%). ^{1}H NMR (600 MHz, $CDCl_3$, -40 °C) major rotamer δ 4.76 – 4.74 (m, 1H), 4.68 (s, 1H), 3.72 (ddd, *J* = 10.7, 7.4, 1.6 Hz, 1H), 3.10 (t, *J* = 11.0 Hz, 1H), 2.71 (m, 1H), 1.88 (ddd, *J* = 12.1, 5.9, 1.6 Hz, 1H), 1.75 (t, *J* = 12.5 Hz, 1H), 1.70 (s, 3H), 1.46 (s, 9H), 1.38 (s, 3H), 1.28 (s, 3H); minor rotamer δ 4.77 – 4.75 (m, 1H), 4.71 (s, 1H), 3.60 (ddd, *J* = 10.7, 7.5, 1.4 Hz, 1H), 3.07 (t, *J* = 11.0 Hz, 1H),

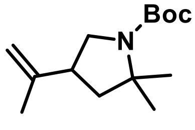

2.72 (m, 1H), 1.83 (ddd, *J* = 12.1, 5.9, 1.2 Hz, 1H), 1.72 (s, 3H), 1.72 – 1.68 (m, 1H), 1.46 (s, 3H), 1.42 (s, 9H), 1.32 (s, 3H). ^{13}C NMR (151 MHz, $CDCl_3$, -40 °C) major rotamer δ 154.4, 144.5, 110.0, 79.2, 59.8, 51.5, 46.5, 40.5, 28.4, 28.3, 26.2, 21.5; minor rotamer δ 153.2, 144.6, 110.2, 78.6, 60.2, 51.5, 45.7, 41.1, 28.5, 27.4, 25.4, 21.3. IR (film) ṽ 2967, 2931, 1686, 1363, 1178, 1142, 1061 cm^{-1}. HRMS (EI$^+$) for $C_{14}H_{25}NO_2$ [M]$^+$: calcd 239.1880, found 239.1880.

***tert*-Butyl (2*R**,3*R**)5,5-dimethyl-3-(prop-1-en-2-yl)-1-tosylpyrrolidine-2-carboxylate (49).** Prepared analogously from enyne **S49** (38.5 mg, 0.10 mmol); colorless oil (26.0 mg, 67 %).^{1}H NMR (400 MHz, $CDCl_3$) δ 7.8 – 7.7 (m, 2H), 7.3 – 7.2 (m, 2H), 4.8 (h, *J* = 1.4 Hz, 1H), 4.7 (q, *J* = 1.2 Hz, 1H), 4.4 (d, *J* = 8.0 Hz, 1H), 2.9 (ddd, *J* = 13.5, 8.2, 5.7 Hz, 1H), 2.4 – 2.3 (m, 4H), 1.8 (p, *J* = 0.7 Hz, 3H), 1.7 – 1.6 (m, 4H), 1.5 (s, 3H), 1.2 (s, 9H). ^{13}C NMR (101 MHz, $CDCl_3$) δ 169.1, 143.1, 140.8, 138.5, 129.4, 127.9, 112.6, 81.7, 66.6, 64.8, 46.0, 43.6, 29.2, 28.7, 28.0, 23.5, 21.6. IR (film) ṽ 2974, 2931, 1737, 14453, 1337, 1151, 1091 cm^{-1}. HRMS (ESI$^+$) for $C_{21}H_{31}NO_4SNa$ [M+Na]$^+$: calcd 416.1866, found 416.1866.

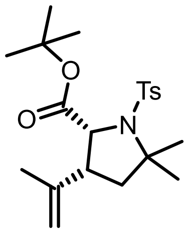

(4*R,5*S**)5-Methoxy-2,2-dimethyl-4-(prop-1-en-2-yl)-1-tosylpyrrolidine (50)** Prepared analogously from enyne **S50** (33.5 mg, 0.10 mmol); colorless oil (20.1 mg, 60 %).^{1}H NMR (400 MHz, C_6D_6) δ 7.9 – 7.8 (m, 2H), 6.8 – 6.7 (m, 2H), 5.3 (d, *J* = 4.3 Hz, 1H), 4.9 (h, *J* = 1.5 Hz, 1H), 4.7 – 4.7 (m, 1H), 3.4 (s, 3H), 2.4 – 2.3 (m, 1H), 2.2 (dd, *J* = 13.4, 11.7 Hz, 1H), 1.9 (s, 3H), 1.7 (dt, *J* = 1.5, 0.7 Hz, 3H), 1.5 (s, 3H), 1.4 (ddd, *J* = 11.7, 6.2, 0.8 Hz, 1H), 1.2 (s, 3H). ^{13}C NMR (101 MHz, C_6D_6) δ 142.3, 142.2, 141.9, 129.4, 127.5, 112.8, 94.2, 64.1, 56.7, 48.3, 43.3, 30.9, 27.4, 22.5, 21.1. IR (film) ṽ 2969, 2941, 1620, 1599, 1448, 1333, 1150, 1058 cm^{-1}. HRMS (ESI$^+$) for $C_{17}H_{25}NO_3SNa$ [M+Na]$^+$: calcd 346.1447, found 346.1447.

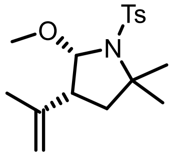

Table S14. Detailed NMR analysis of the pyrrolidine **50** ($CDCl_3$, 25 °C, 600 MHz)

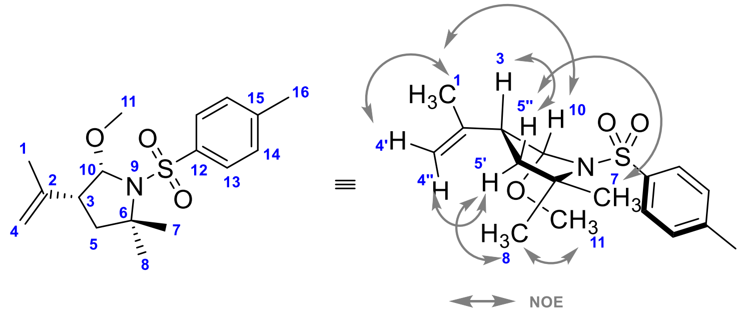

Atom C	Atom H	δ [ppm]	J	COSY	HSQC	HMBC	NOESY
1		22.41			1	3, 4', 4''	
	1	1.65	1.50, 0.80	4', 4''	1	2, 3, 4	3, 4', 5', 10, 11
2		141.84				1, 3, 5'	
3		48.20			3	1, 4', 4'', 5', 5''	
	3	2.37	13.40, 5.30	5', 5'', 10	3	1, 2, 4, 5, 10	1, 5'', 7, 10
4		112.70			4', 4''	1, 3	
	4'	4.86	1.50	1, 4''	4	1, 3	1, 4''
	4''	4.70	1.70, 0.80	1, 4'	4	1, 3	4', 5'
5		43.29			5', 5''	3, 10	
	5'	2.16	13.40, 11.80, 0.60	3, 5''	5	2, 3, 6, 7, 8	1, 4'', 5'', 8
	5''	1.36	11.70, 6.20, 0.80	3, 5'	5	3, 7, 10	3, 5', 7
6		64.00				5', 10	
7		27.38			7	5', 5'', 8	
	7	1.23			7	8	3, 5'', 8, 10, 13
8		30.82			8	5', 7	
	8	1.47			8	7	5', 7, 11, 13
10		94.19			10	3, 5'', 11	
	10	5.33	4.40	3	10	5, 6, 11	1, 3, 7, 11, 13
11		56.67			11	10	
	11	3.40			11	10	1, 8, 10, 13
12		142.25				14	
13		127.43			13	13, 14	
	13	7.86		14	13	13, 15	7, 8, 10, 11, 14
14		129.33			14	16	
	14	6.79		13, 16	14	12, 13, 16	13, 16
15		142.13				13, 16	
16		21.04			16	14	
	16	1.91		14	16	14, 15	14

Atom N	δ [ppm]	J	COSY	HSQC	HMBC	NOESY
9	-247.15					

Exemplary Downstream Functionalization

***tert*-Butyl 7-acetyl-5-oxa-2-azaspiro[3.4]octane-2-carboxylate (52).** Ozone (O_3/O_2 mixture generated

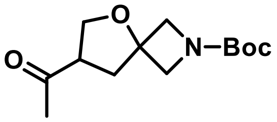

from ozonizer) was bubbled through a solution of alkene **20** (57 mg, 0.23 mmol) in CH_2Cl_2 (6 mL) at –78 °C until a faint blue color persisted (ca. 2 min). The solution was purged with argon before dimethylsulfide (0.82 mL, 11.2 mmol) was added and the mixture was allowed to reach room temperature. After stirring for 7 h, the solvent was removed under reduced pressure and the crude product was purified by flash chromatography (silica, hexanes/EtOAc 2:1 – 1:1) to yield the title compound as a colorless oil (43.6 mg, 76%). ^{1}H NMR (400 MHz, $CDCl_3$) δ 4.05 – 3.92 (m, 5H), 3.87 (dd, *J* = 9.1, 1.1 Hz, 1H), 3.26 (tt, *J* = 8.3, 6.3 Hz, 1H), 2.37 (dd, *J* = 13.0, 6.4 Hz, 1H), 2.25 (dd, *J* = 13.0, 8.7 Hz, 1H), 2.20 (s, 3H), 1.43 (s, 9H). ^{13}C NMR (101 MHz, $CDCl_3$) δ 206.7, 156.4, 79.8, 78.9, 68.9, 61.8 (br, 2C), 51.5, 38.0, 29.3, 28.5. IR (film) ṽ 2975, 2877, 1694, 1392, 1159, 1089 cm^{-1}. HRMS (ESI$^+$) for $C_{14}H_{21}NO_4$ [M+Na]$^+$: calcd 278.1363, found 278.1361.

***tert*-Butyl 7-(2-hydroxypropan-2-yl)-5-oxa-2-azaspiro[3.4]octane-2-carboxylate (53).** $PhSiH_3$ (58 mg,

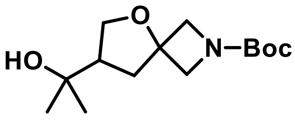

0.54 mmol) and Co(acac)$_2$ (12.7 mg, 0.05 mmol) were added to a solution of alkene **20** (50.3 mg, 0.20 mmol) in THF (3.9 mL) before O_2 was bubbled through the mixture for 2 min. The solution was stirred for 2 h under an atmosphere of oxygen at room temperature. The solvent was removed under reduced pressure and the crude product was purified by flash chromatography (silica, CH_2Cl_2/MeOH 100:1 – 50:1 – 20:1) to yield the title compound as a colorless oil (37.2 mg, 69%). ^{1}H NMR (400 MHz, $CDCl_3$) δ 4.02 (dd, *J* = 9.4, 1.1 Hz, 1H), 3.96 – 3.87 (m, 3H), 3.80 (dd, *J* = 8.9, 1.0 Hz, 1H), 3.75 (t, *J* = 8.5 Hz, 1H), 2.34 (dq, *J* = 9.3, 8.2 Hz, 1H), 2.14 (dd, *J* = 12.6, 8.2 Hz, 1H), 1.99 (dd, *J* = 12.7, 9.4 Hz, 1H), 1.42 (s, 9H), 1.21 (s, 3H), 1.19 (s, 3H). ^{13}C NMR (101 MHz, $CDCl_3$) δ 156.5, 79.6, 78.8, 70.5, 68.8, 62.8, 61.3, 49.6, 37.3, 28.7 (2C), 28.5. IR (film) ṽ 3451 (br), 2973, 2876, 1680, 1393, 1366, 1160, 1098 cm^{-1}. HRMS (ESI$^+$) for $C_{14}H_{25}NO_4$ [M+Na]$^+$: calcd 294.1676, found 294.1674.

***tert*-Butyl 7-(5-methoxy-2-methyl-5-oxopentan-2-yl)-5-oxa-2-azaspiro[3.4]octane-2-carboxylate (54).**

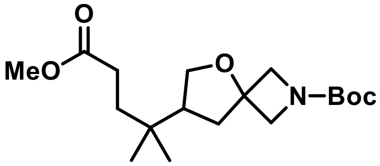

Prepared according to a previously described method.[21] Fe(acac)$_3$ (70.2 mg, 0.2 mmol) was added to a solution of alkene **20** (50.4 mg, 0.20 mmol) in 1,2-dichloroethane (1.6 mL) and ethylene glycol (0.4 mL). Methyl acrylate (51 mg, 0.60 mmol) and phenylsilane (54 mg, 0.50 mmol) were added and the mixture was stirred at 60 °C for 1 h before brine (1 mL) and water (1 mL) were introduced. The mixture was extracted with EtOAc (3 x 25 mL) and the combined organic layers were washed with brine and dried over $MgSO_4$. The solvent was removed under reduced pressure and the crude product was purified by flash chromatography (silica, hexanes/EtOAc 10:1 – 4:1) to yield the title compound as a colorless oil (50.5 mg, 74%). ^{1}H NMR (400 MHz, $CDCl_3$) δ 4.02 (dd, *J* = 9.4, 1.0 Hz, 1H), 3.89 (m, 2H), 3.83 (t, *J* = 8.3 Hz, 1H), 3.76 (dd, *J* = 8.9, 1.1 Hz, 1H), 3.65 (s, 3H), 3.59 (t, *J* = 8.8 Hz, 1H), 2.30 – 2.24 (m, 2H), 2.16 (m, 1H), 2.07 (dd, *J* = 12.3, 7.6 Hz, 1H), 1.78 (dd, *J* = 12.3, 10.5 Hz, 1H), 1.56 – 1.49 (m, 2H), 1.41 (s, 9H), 0.84 (s, 3H), 0.82 (s, 3H). ^{13}C NMR (101 MHz, $CDCl_3$) δ 174.4, 156.5, 79.6, 78.6, 68.7, 63.1, 61.0, 51.8, 48.2, 37.1, 36.2, 33.4, 29.2, 28.5, 23.9, 23.8. IR

(film) ṽ 2962, 2876, 2250, 1737, 1698, 1392, 1161, 1098 cm^{-1}. HRMS (ESI$^+$) for $C_{18}H_{31}NO_5$ [M+Na]$^+$: calcd 364.2094, found 364.2096.

***tert*-Butyl 7-((2,2,6,6-tetramethylpiperidin-1-yl)oxy)-5-oxa-2-azaspiro[3.4]octane-2-carboxylate (55).**

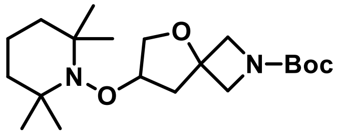

Prepared according to a previously described method.[22] Ozone was bubbled through a solution of alkene **20** (51.0 mg, 0.20 mmol) in MeOH (8 mL) at −78 °C until a faint blue color persisted. The solution was purged with argon before TEMPO (63 mg, 0.40 mmol) in MeOH (0.5 mL) was added. A solution of $FeSO_4{\cdot}7H_2O$ (5 wt% in H_2O, 1.46 mL, 0.26 mmol) was introduced and the mixture was allowed to warm to room temperature with stirring for 10 min. $Na_2S_2O_3$ solution (10% sat., 2 mL) and sat. $NaHCO_3$ solution (1 ml) were added. The mixture was extracted with EtOAc (3 x 20 mL) and the combined organic layers were washed with brine and dried over $MgSO_4$. The crude product was purified by flash chromatography (silica, hexanes/EtOAc 10:1) to yield the title compound as a colorless oil (46.9 mg, 63%). ^{1}H NMR (400 MHz, $CDCl_3$) δ 4.53 (pent, *J* = 5.0 Hz, 1H), 4.04 – 3.91 (m, 5H), 3.85 (d, *J* = 8.7 Hz, 1H), 2.29 (d, *J* = 5.7 Hz, 2H), 1.69 – 1.27 (m, 6H), 1.43 (s, 9H), 1.17 (s (br), 3H), 1.08 (s (br), 6H), 1.04 (s (br), 3H). ^{13}C NMR (101 MHz, $CDCl_3$) δ 156.5, 85.6, 79.6, 77.8, 72.8, 62.5 (br), 61.6 (br), 60.0, 59.6, 42.5, 40.2, 34.3 (br), 33.6 (br), 28.5, 20.3 (br), 17.2. IR (film) ṽ 2973, 2931, 2874, 1703, 1391, 1090 cm^{-1}. HRMS (ESI$^+$) for $C_{20}H_{36}N_2O_4$ [M+H]$^+$: calcd 369.2748, found 369.2742.

***tert*-Butyl 7-oxo-5-oxa-2-azaspiro[3.4]octane-2-carboxylate (56).** *Prepared in analogy to a previously described method*.[23]

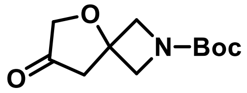

Ozone was bubbled through a solution of alkene **20** (50.5 mg, 0.20 mmol) in MeOH (8 mL) at −78 °C until a faint blue color persisted. The solution was purged with argon before a solution of TEMPO (47 mg, 0.20 mmol) in MeOH (0.5 mL) was added. Next, a solution of $FeSO_4{\cdot}7H_2O$ (5 wt% in H_2O, 1.44 mL, 0.26 mmol) was introduced and the mixture was warmed to room temperature and stirred for 10 min. The mixture was cooled to 0 °C and magnesium bis(monoperoxyphthalate) hexahydrate (MMPP, 185 mg, ~80%, 0.30 mmol) was added. After stirring for 2 h at 0 °C, $Na_2S_2O_3$ solution (10% *w/w*, 2 mL) and sat. $NaHCO_3$ solution (1 ml) were added. The mixture was extracted with EtOAc (3 x 20 mL) and the combined organic layers were washed with brine, dried over $MgSO_4$, and evaporated. The crude product was purified by flash chromatography (silica, hexanes/EtOAc 10:1 – 4:1 – 3:1) to yield the title compound as a colorless oil (28.2 mg, 62%). ^{1}H NMR (400 MHz, $CDCl_3$) δ 4.11 (dd, *J* = 9.3, 1.1 Hz, 2H), 4.00 (s, 2H), 3.99 (dd, *J* = 9.4, 1.1 Hz, 2H), 2.70 (s, 2H), 1.43 (s, 9H). ^{13}C NMR (101 MHz, $CDCl_3$) δ 212.3, 156.3, 80.2, 77.5, 71.0, 61.2, 45.7, 28.4. The spectral data is consistent with literature.[10]

***tert*-Butyl 7-(phenylsulfonyl)-5-oxa-2-azaspiro[3.4]octane-2-carboxylate (57).** *Prepared in analogy to a previously described procedure*.[24]

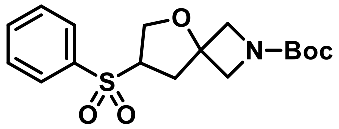

Ozone was bubbled through a solution of alkene **20** (50.8 mg, 0.20 mmol) in MeOH (8 mL) at −78 °C until a faint blue color persisted. The solution was purged with argon before diphenyl disulfide (129 mg, 0.59 mmol) was added. The mixture was warmed to 0 °C and stirred for 10 min at that temperature before a $FeSO_4{\cdot}7H_2O$ solution (5 wt% in H_2O, 1.34 mL, 0.24 mmol) was introduced over 1 min. After stirring for another 5 min, the mixture was diluted with water (5 mL) and CH_2Cl_2 (10 mL) and the layers were separated. The aqueous phase was extracted with CH_2Cl_2 (2 x 10 mL) and the combined organic layers were washed with brine and dried over $MgSO_4$. The solvent

was removed under reduced pressure and the crude product was purified by flash chromatography (silica, hexanes/EtOAc 10:1 – 4:1) to yield the corresponding thioether product (43.3 mg) contaminated with hydrodealkenylated side product.

m-CPBA (123 mg, ~70%, 0.50 mmol) was added to a solution of this crude material in CH_2Cl_2 (2 mL) and the resulting mixture was stirred for 2 h at room temperature before sat. $Na_2S_2O_3$ solution (1 mL) and sat. $NaHCO_3$ solution (1 mL) were added. The biphasic mixture was extracted with CH_2Cl_2 (2 x 15 mL) and the combined organic layers were washed with brine and dried over $MgSO_4$. The solvent was removed under reduced pressure and the crude product was purified by flash chromatography (silica, hexanes/EtOAc 2:1 – 1:1) to yield the title compound as a colorless oil (31.8 mg, 45%). ^{1}H NMR (400 MHz, $CDCl_3$) δ 7.92 – 7.87 (m, 2H), 7.73 – 7.67 (m, 1H), 7.63 – 7.57 (m, 2H), 4.22 (dd, *J* = 10.2, 5.9 Hz, 1H), 4.04 – 3.93 (m, 4H), 3.87 – 3.78 (m, 2H), 2.62 (dd, *J* = 14.0, 5.6 Hz, 1H), 2.34 (dd, *J* = 14.0, 9.4 Hz, 1H), 1.42 (s, 9H). ^{13}C NMR (101 MHz, $CDCl_3$) δ 156.3, 138.1, 134.4, 129.7, 128.6, 80.0, 79.2, 66.8, 63.7, 61.7 (br), 60.9 (br), 36.6, 28.5. IR (film) ṽ 2977, 2878, 1692, 1394, 1147, 1086 cm^{-1}. HRMS (ESI$^+$) for $C_{17}H_{23}NO_5S$ $[M+Na]^+$: calcd 376.1189, found 376.1186.

Copies of NMR Spectra
Building Blocks

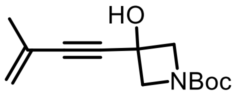

S12

^{1}H NMR (400 MHz, $CDCl_3$)

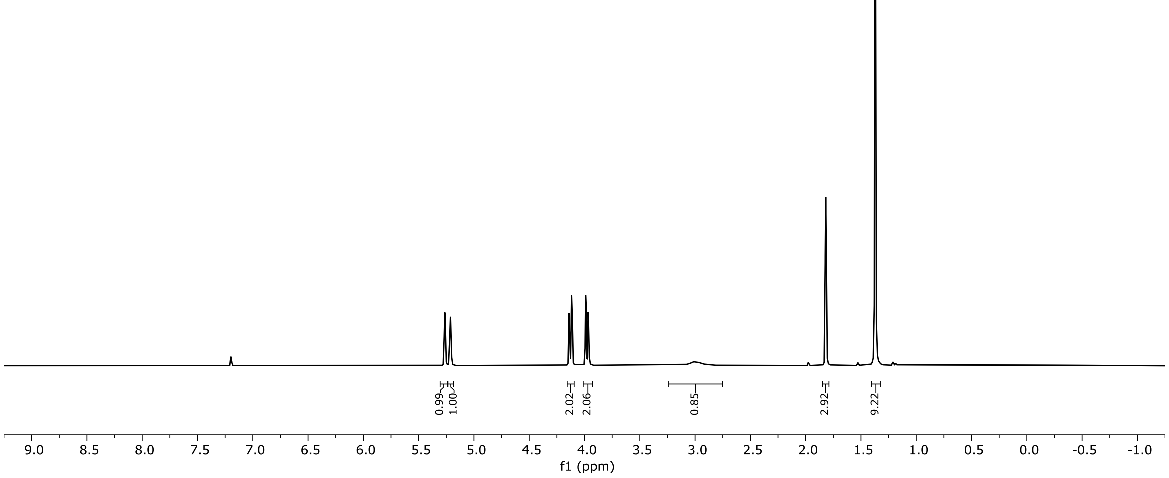

^{13}C NMR (101 MHz, $CDCl_3$)

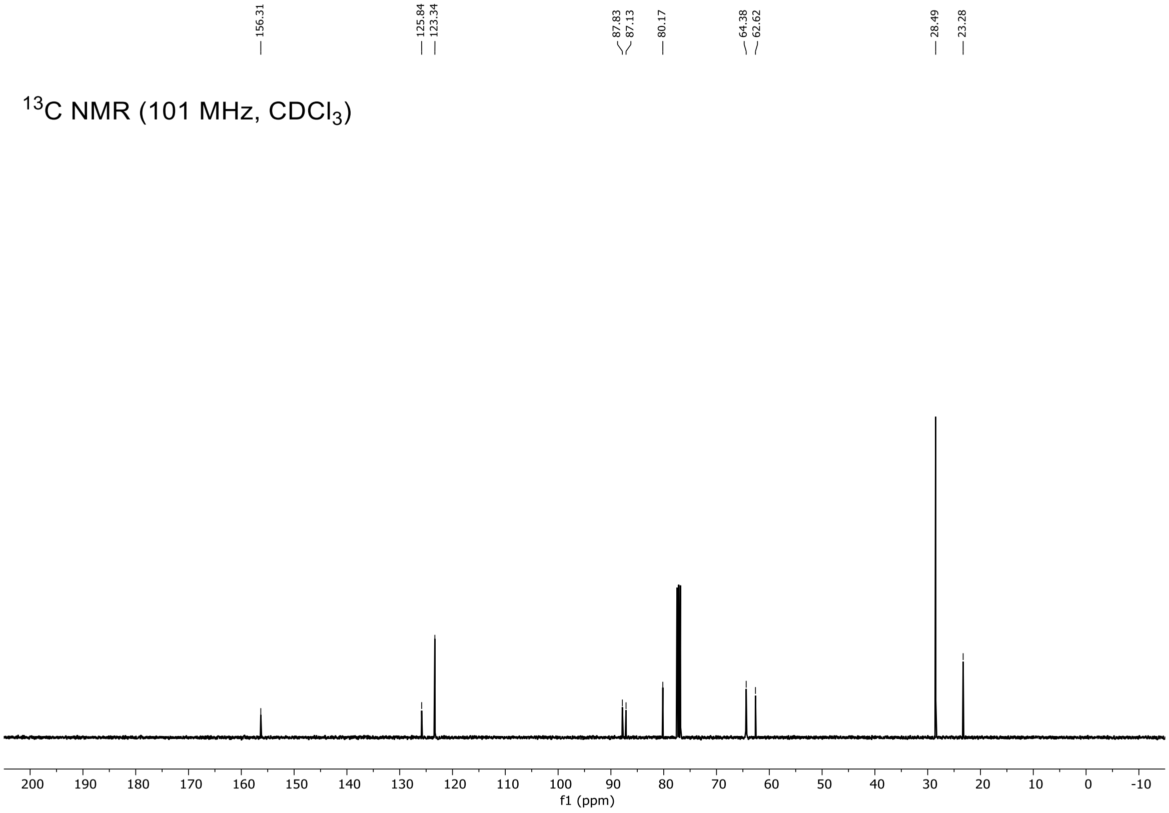

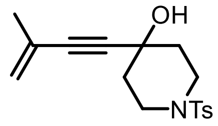

S13

^{1}H NMR (400 MHz, $CDCl_3$)

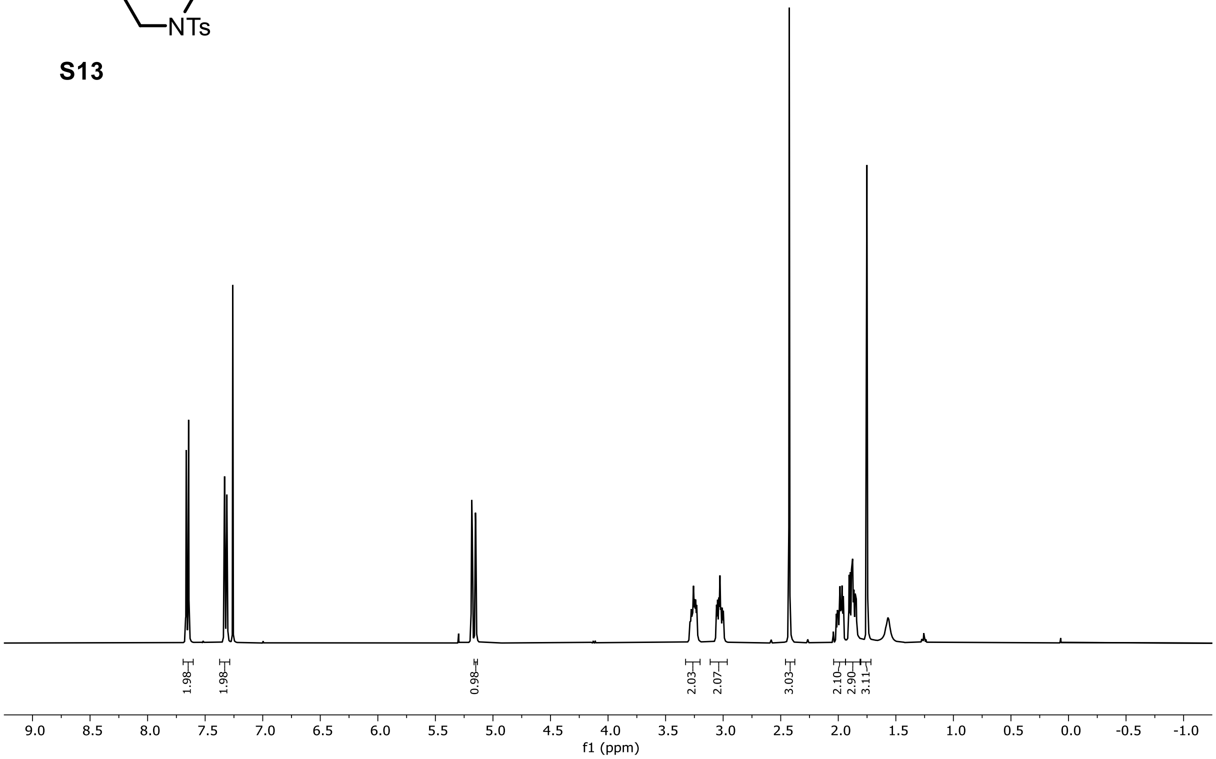

^{13}C NMR (101 MHz, $CDCl_3$)

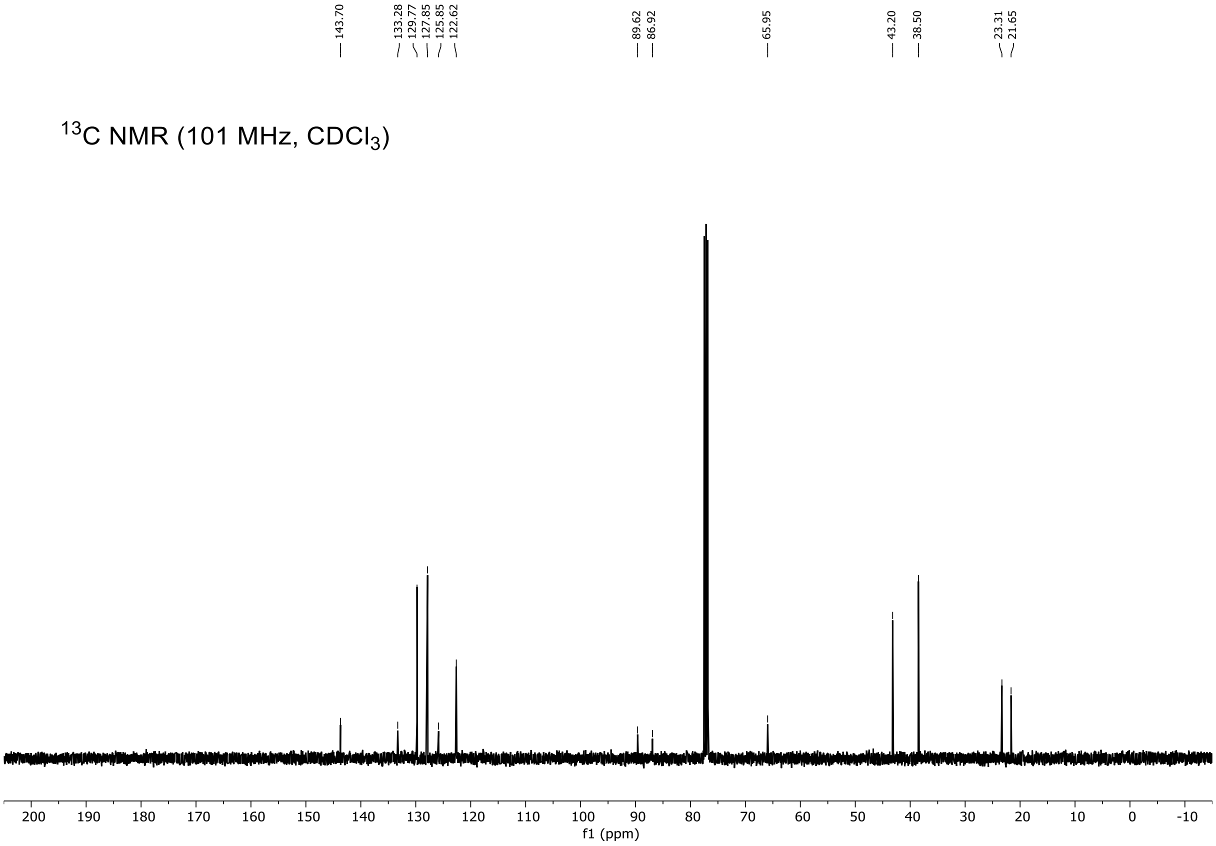

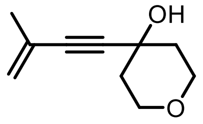

S14

^{1}H NMR (400 MHz, $CDCl_3$)

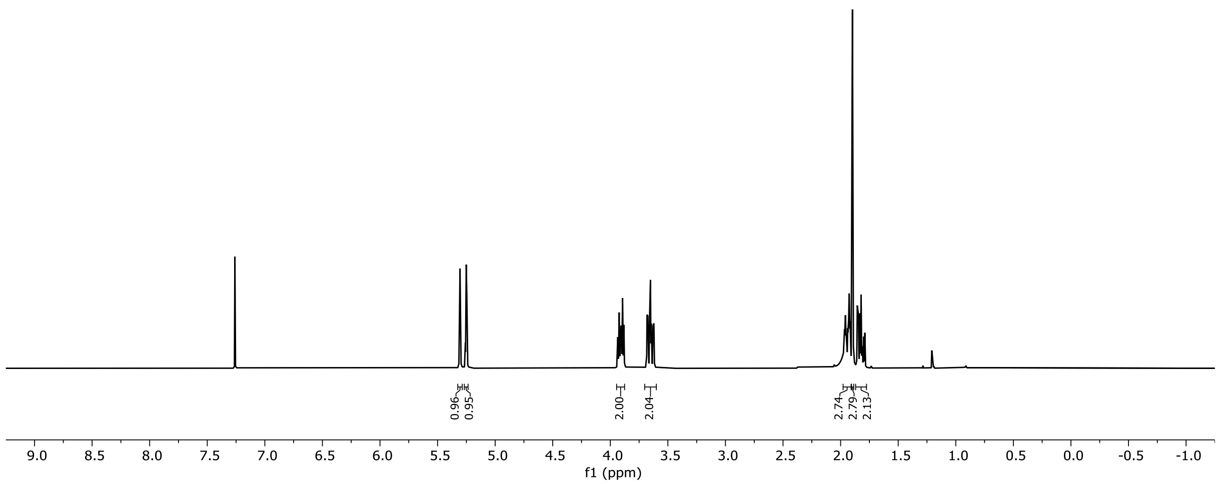

^{13}C NMR (101 MHz, $CDCl_3$)

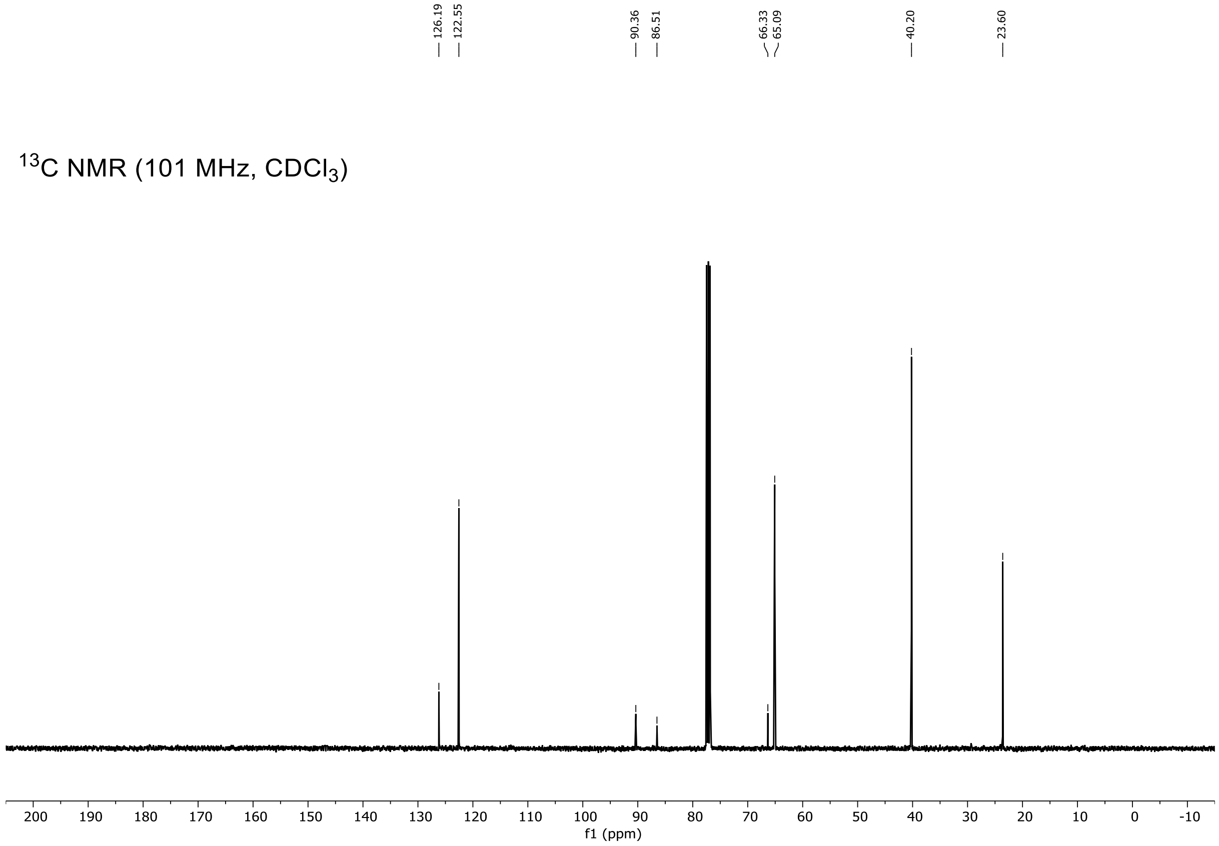

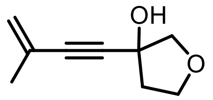

S15

^{1}H NMR (400 MHz, $CDCl_3$)

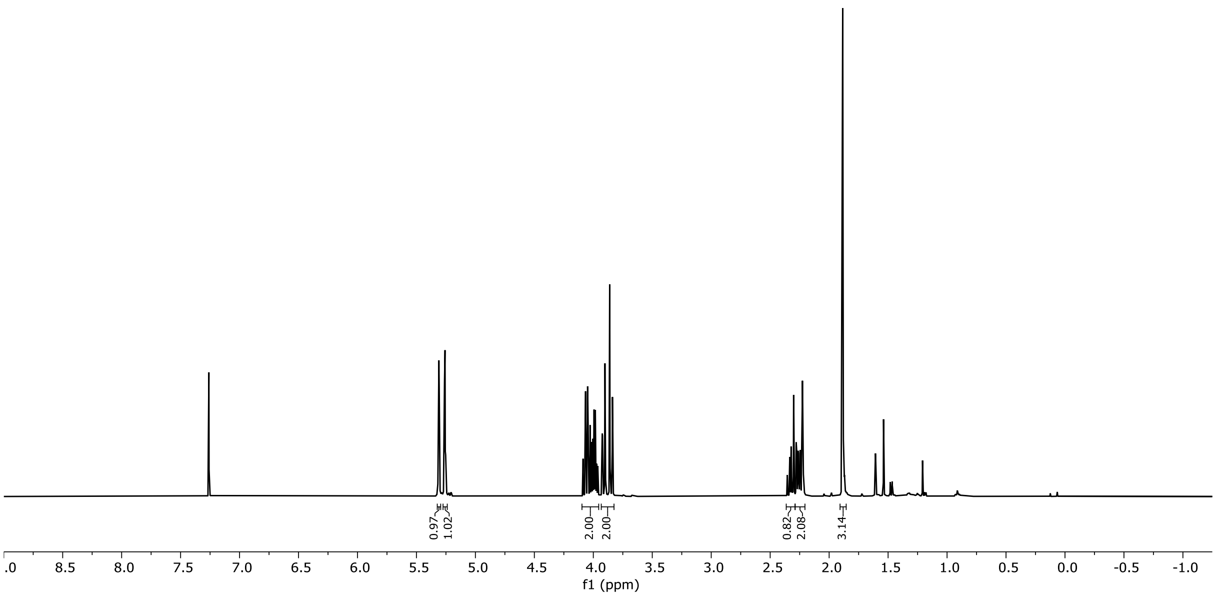

^{13}C NMR (101 MHz, $CDCl_3$)

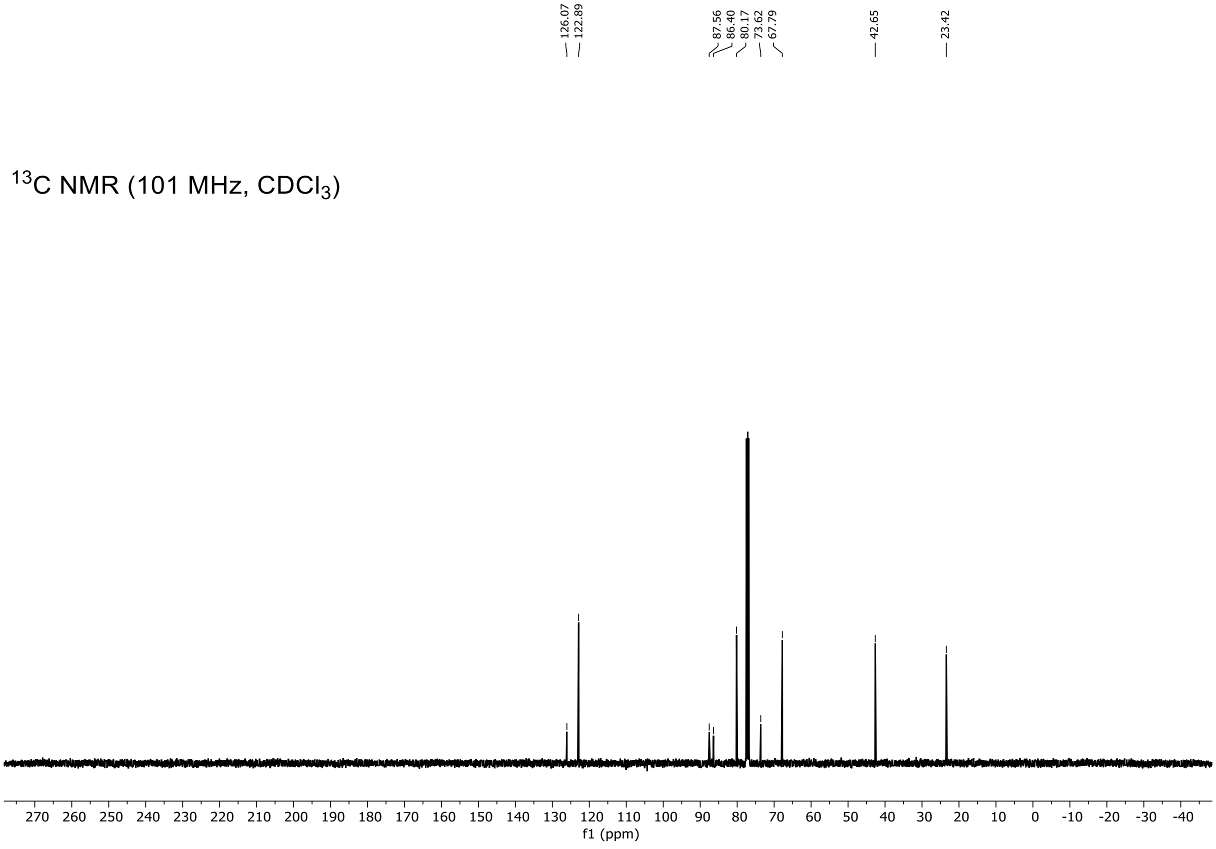

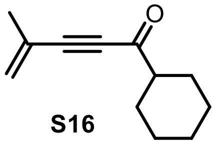

S16

^{1}H NMR (400 MHz, $CDCl_3$)

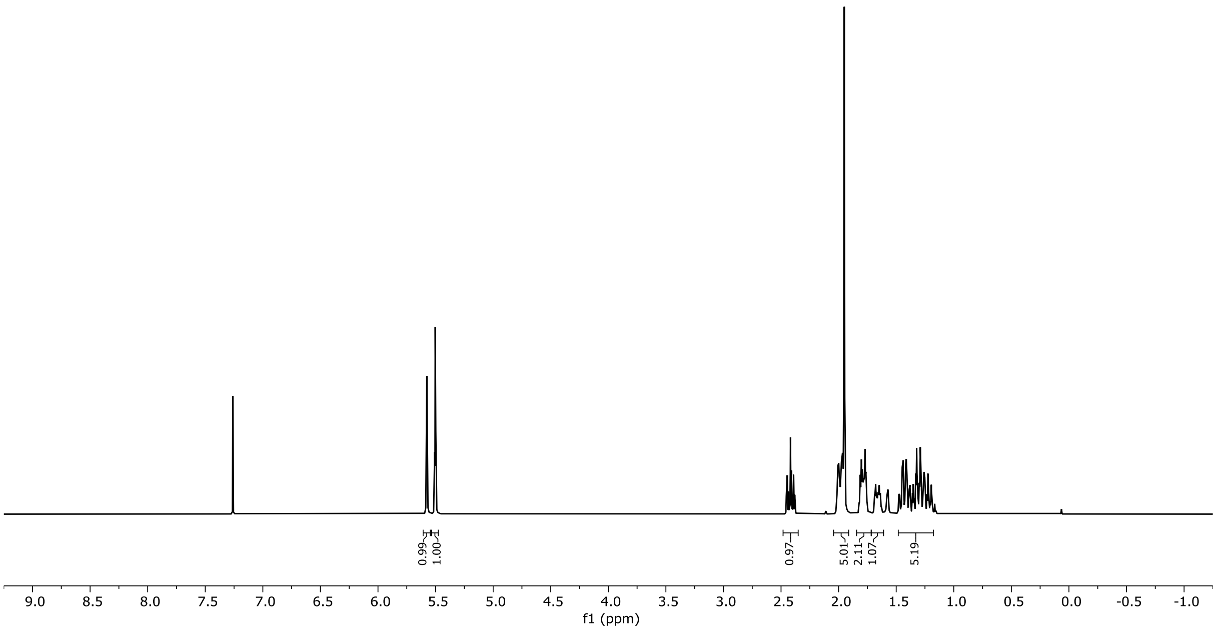

^{13}C NMR (101 MHz, $CDCl_3$)

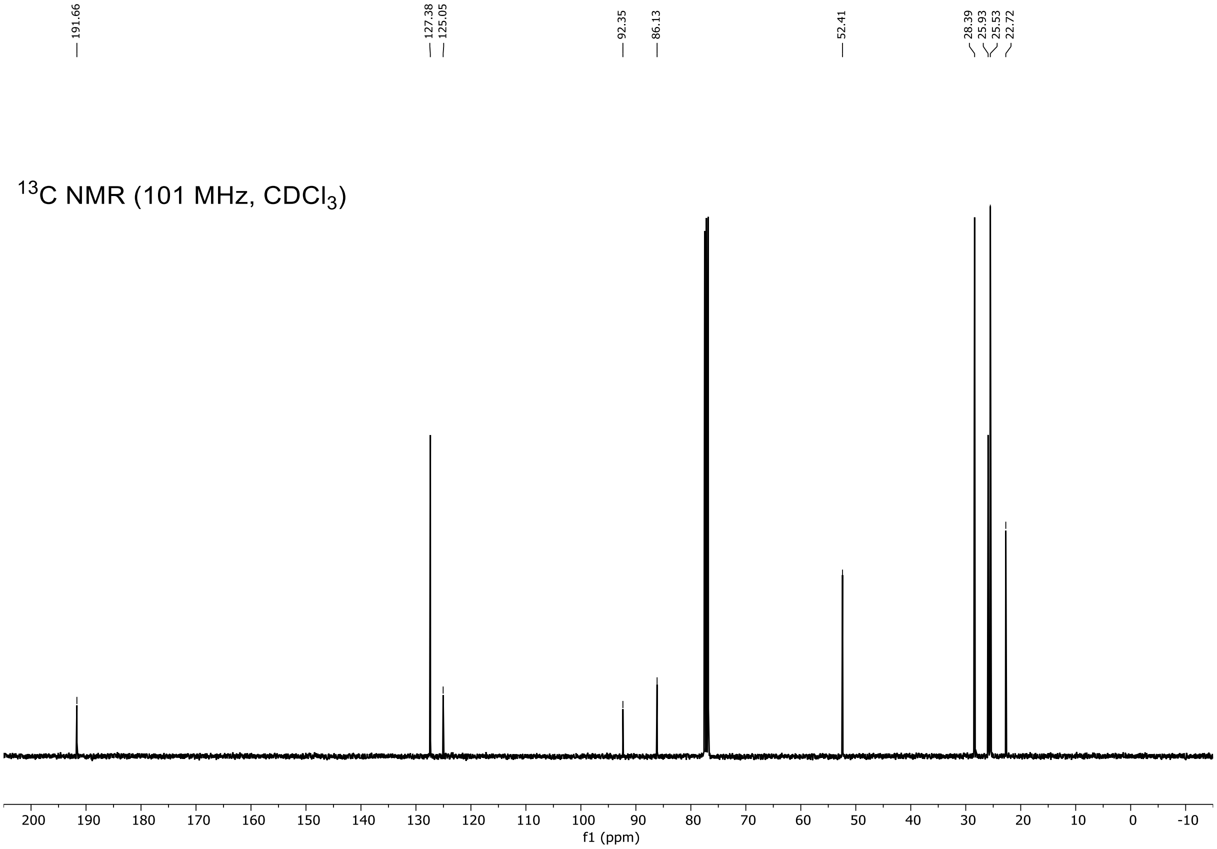

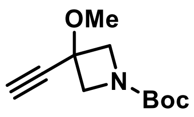

S17

^1H NMR (400 MHz, $CDCl_3$)

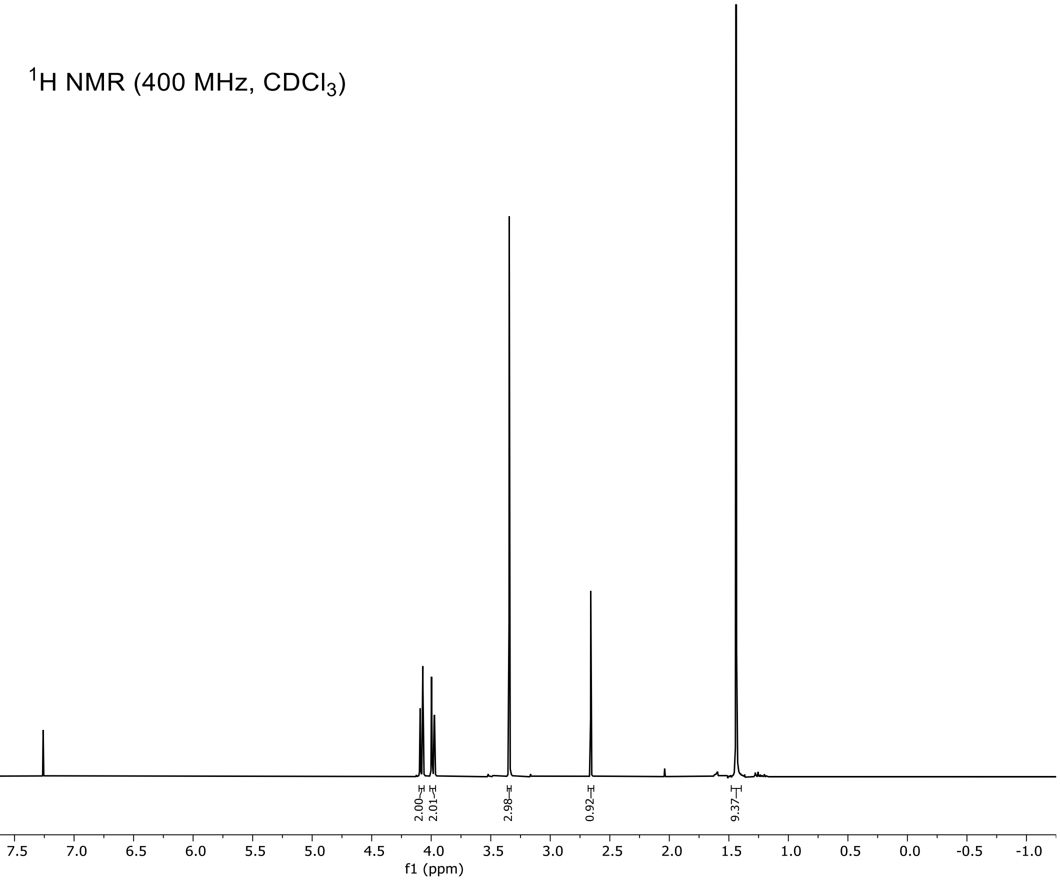

^{13}C NMR (101 MHz, $CDCl_3$)

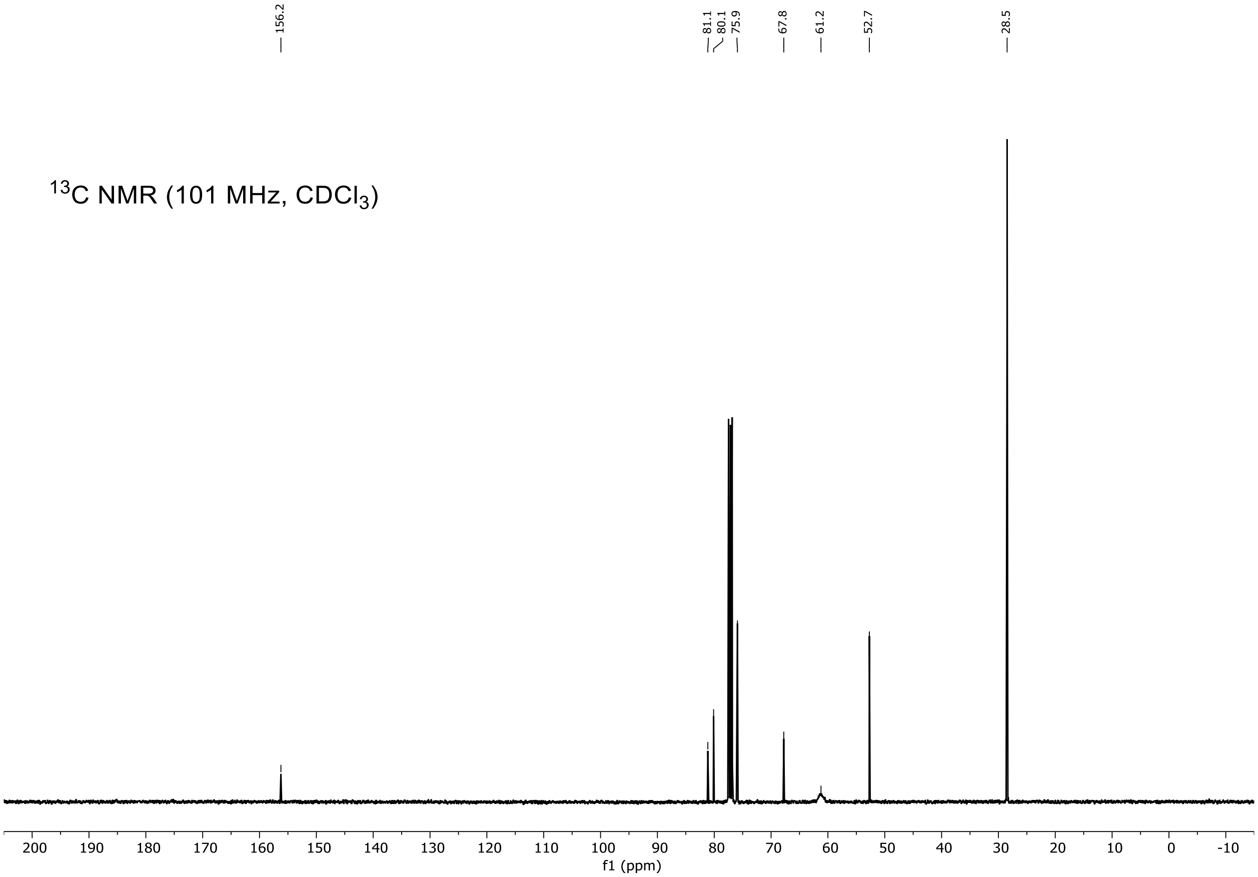

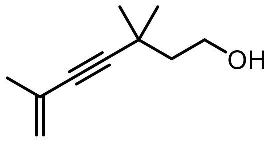

S18 ^{1}H NMR (400 MHz, $CDCl_3$)

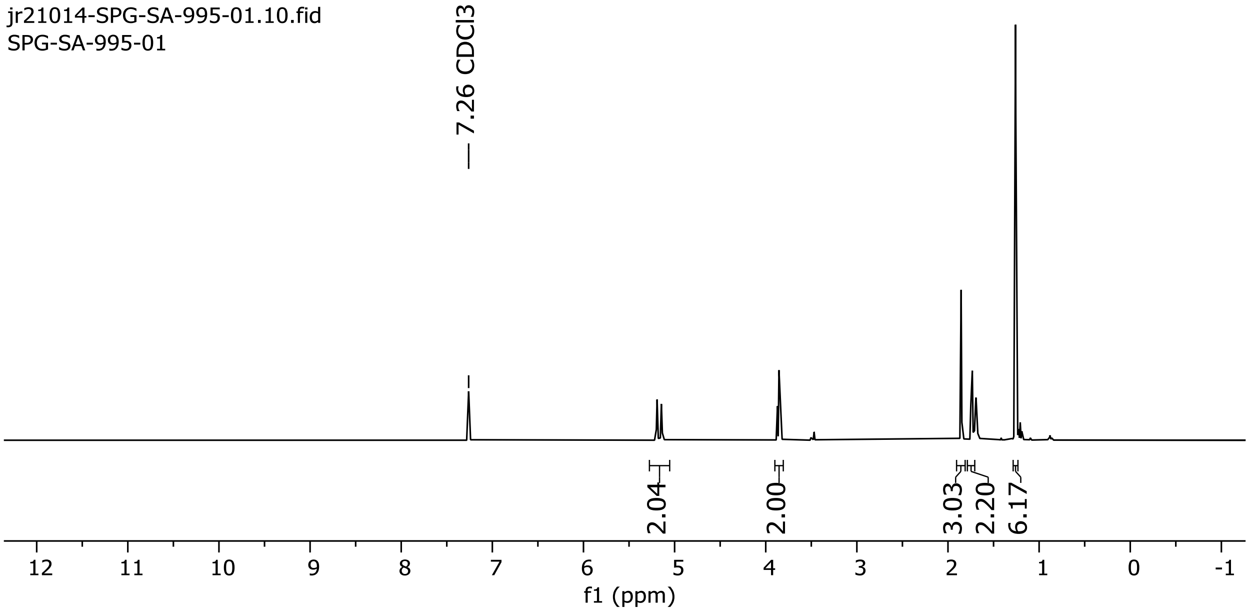

^{13}C NMR (101 MHz, $CDCl_3$)

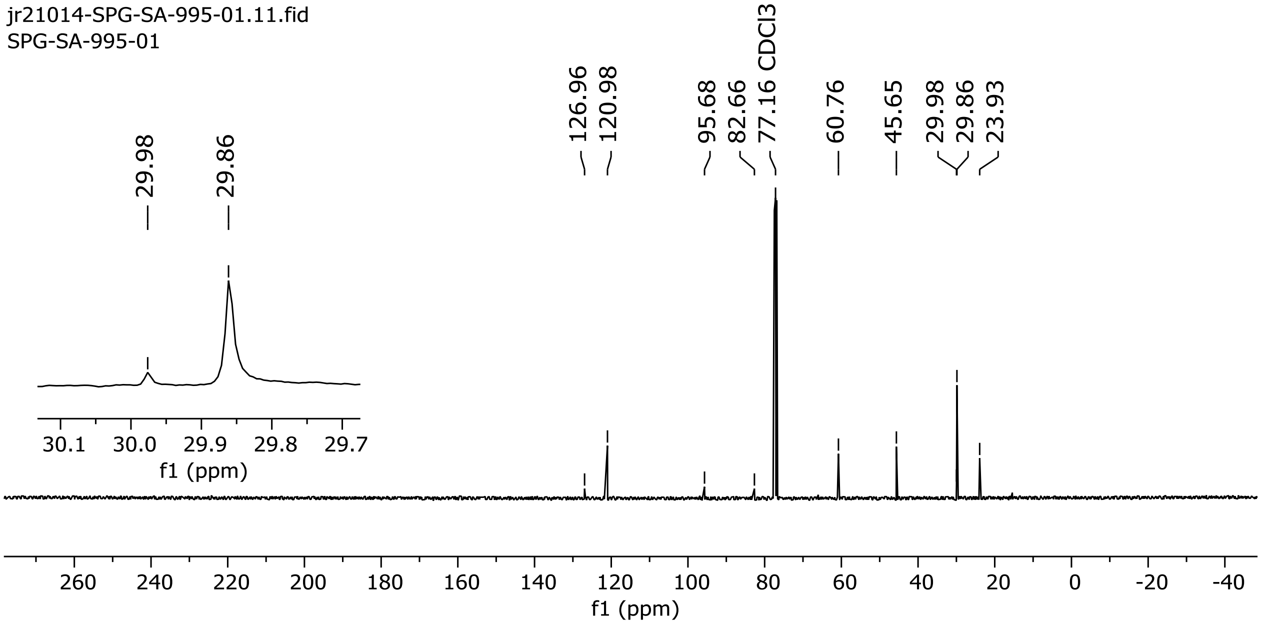

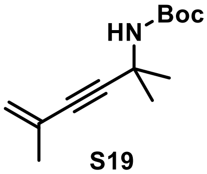

S19

^{1}H NMR (400 MHz, $CDCl_3$)

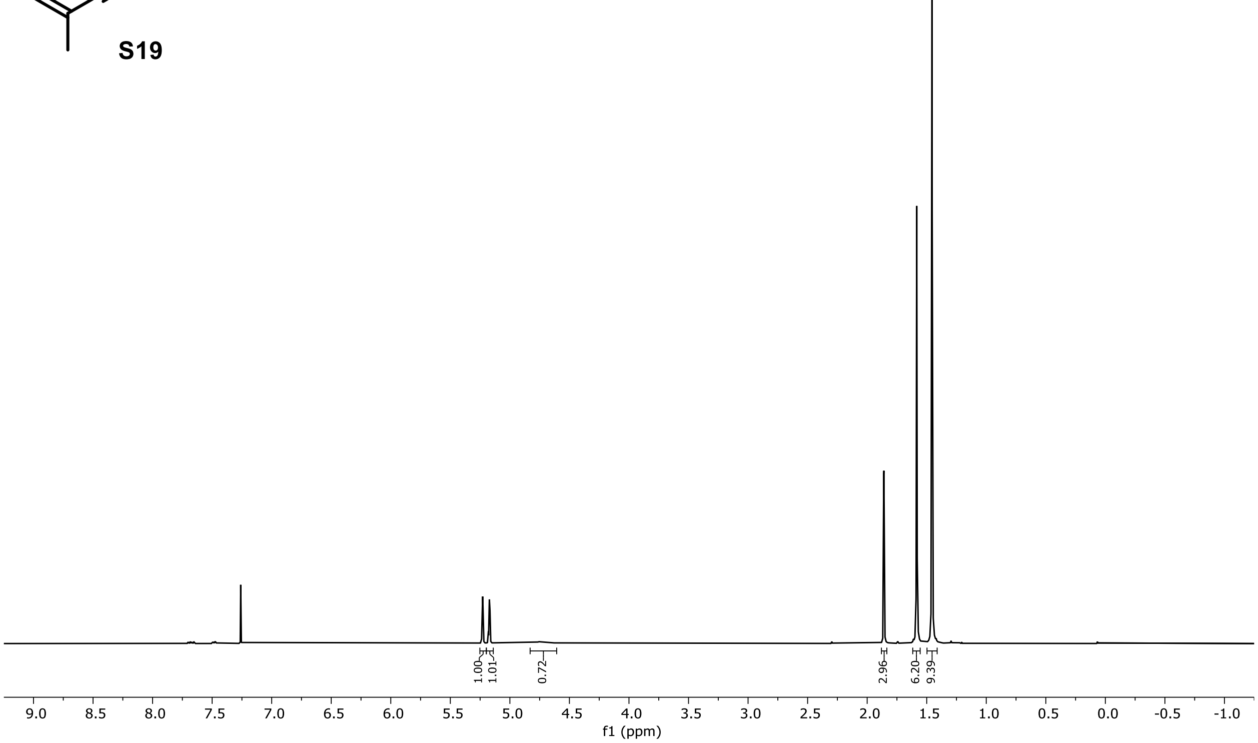

^{13}C NMR (101 MHz, $CDCl_3$)

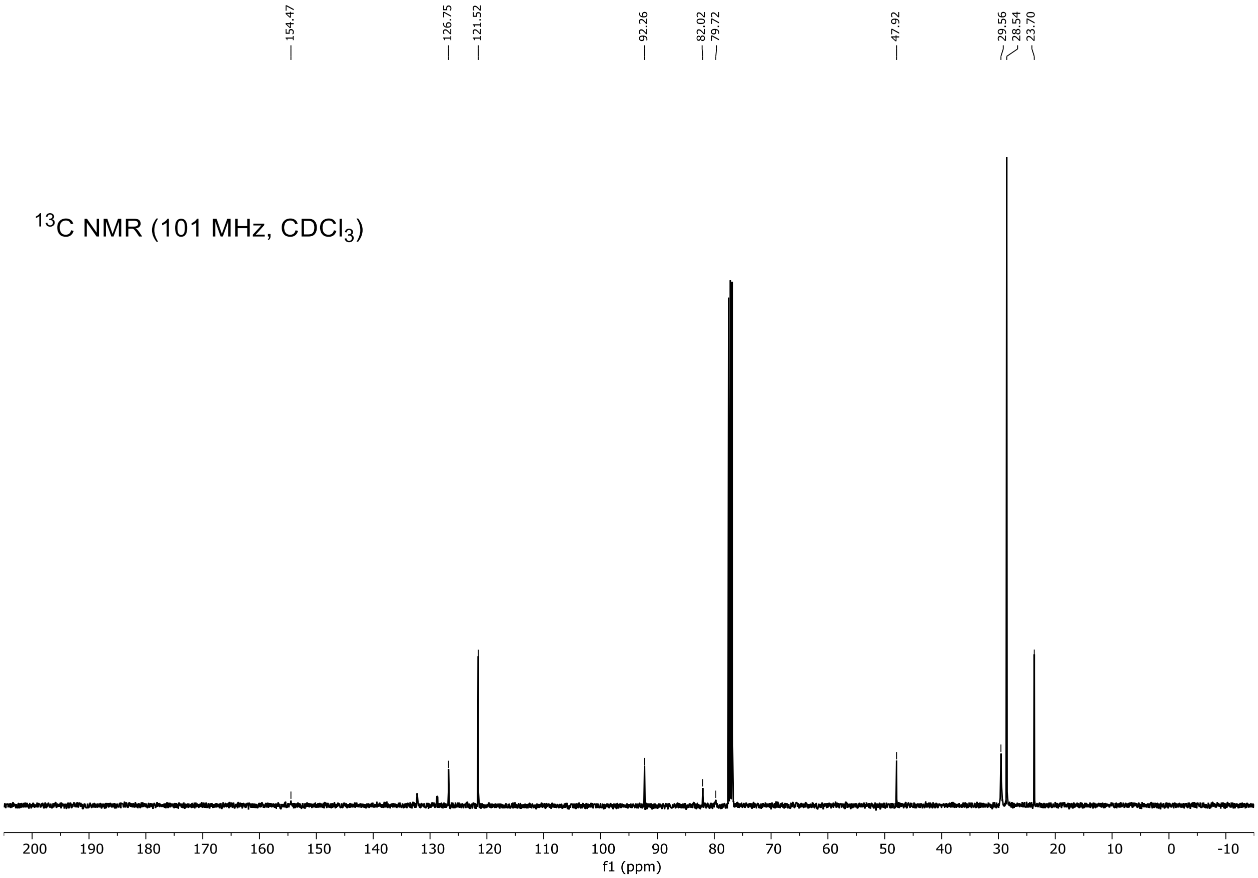

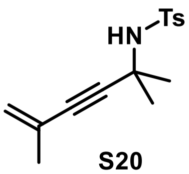

S20

^{1}H NMR (400 MHz, $CDCl_3$)

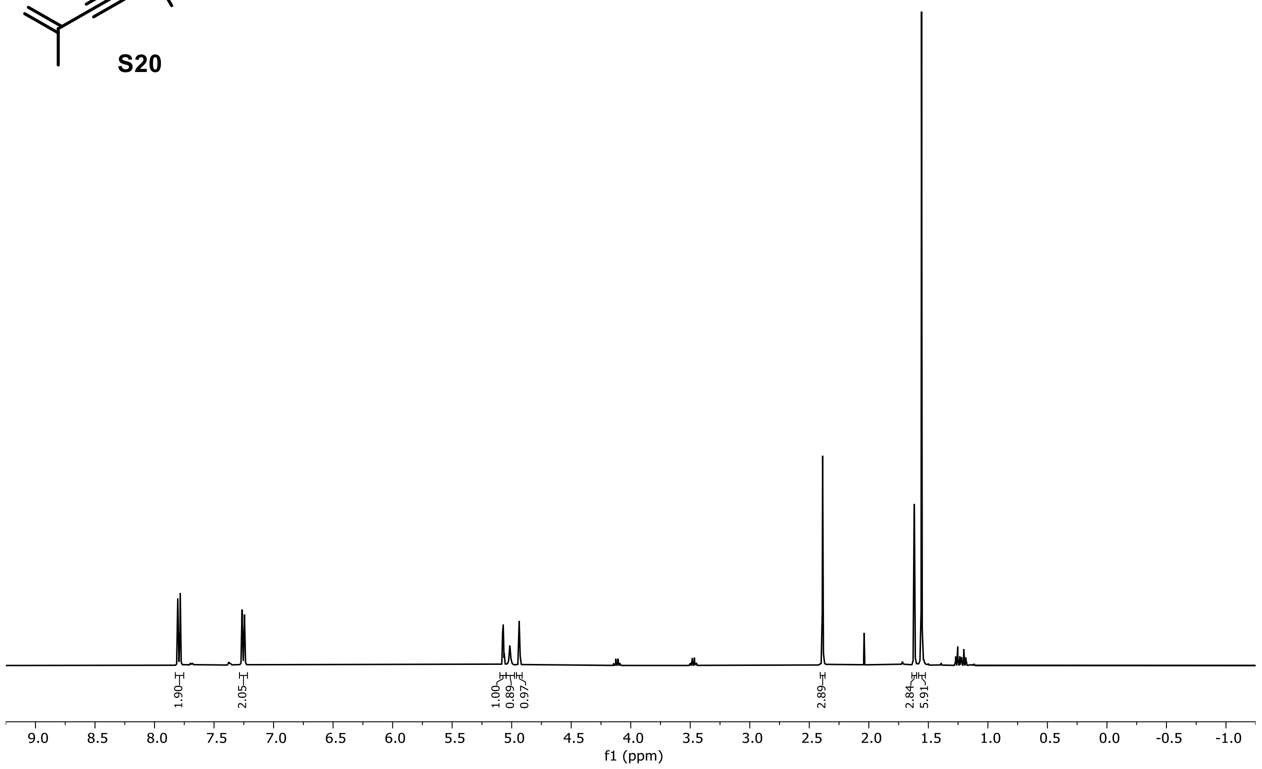

^{13}C NMR (101 MHz, $CDCl_3$)

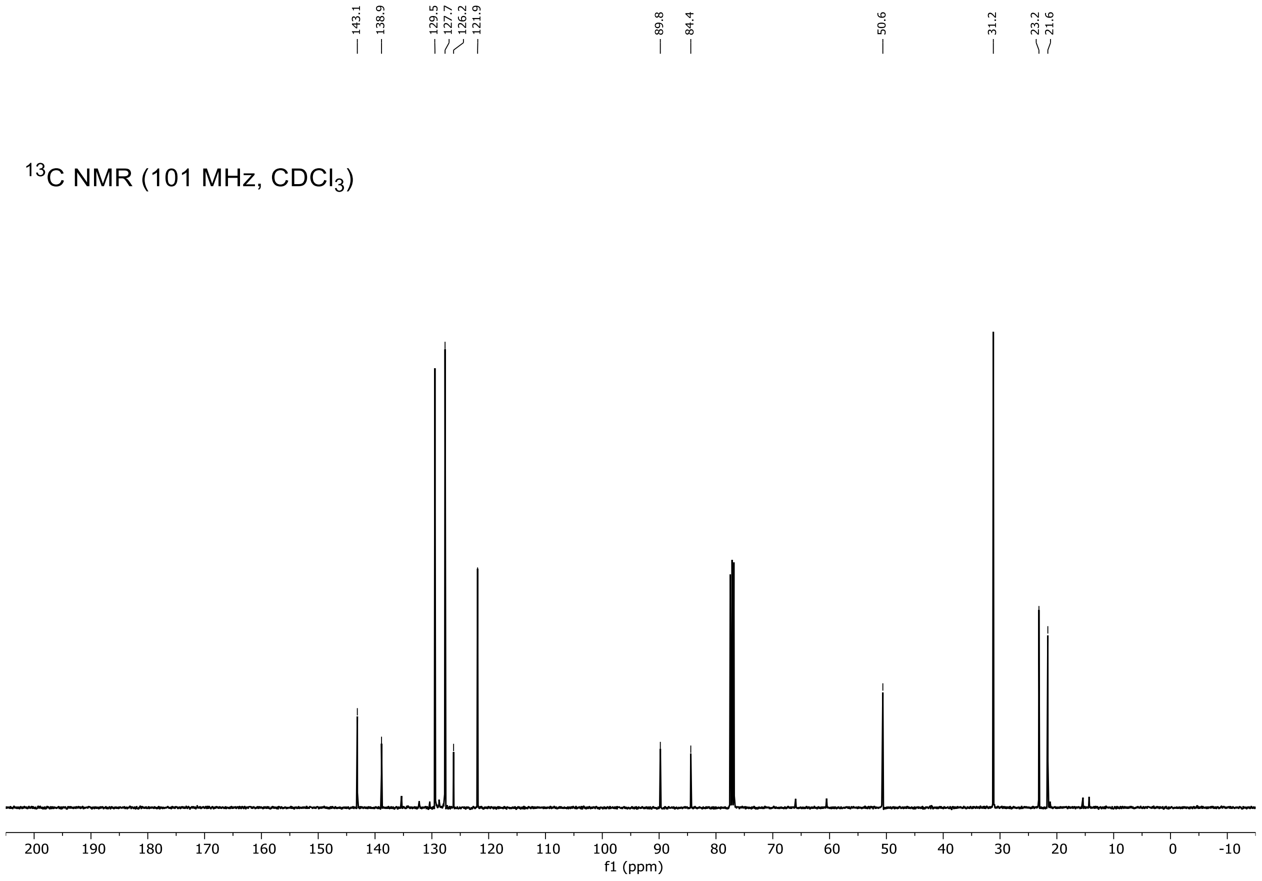

Substrates for *gem*-Hydrogenation/C–H Insertion

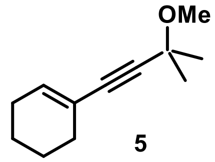

5

^{1}H NMR (400 MHz, $CDCl_3$)

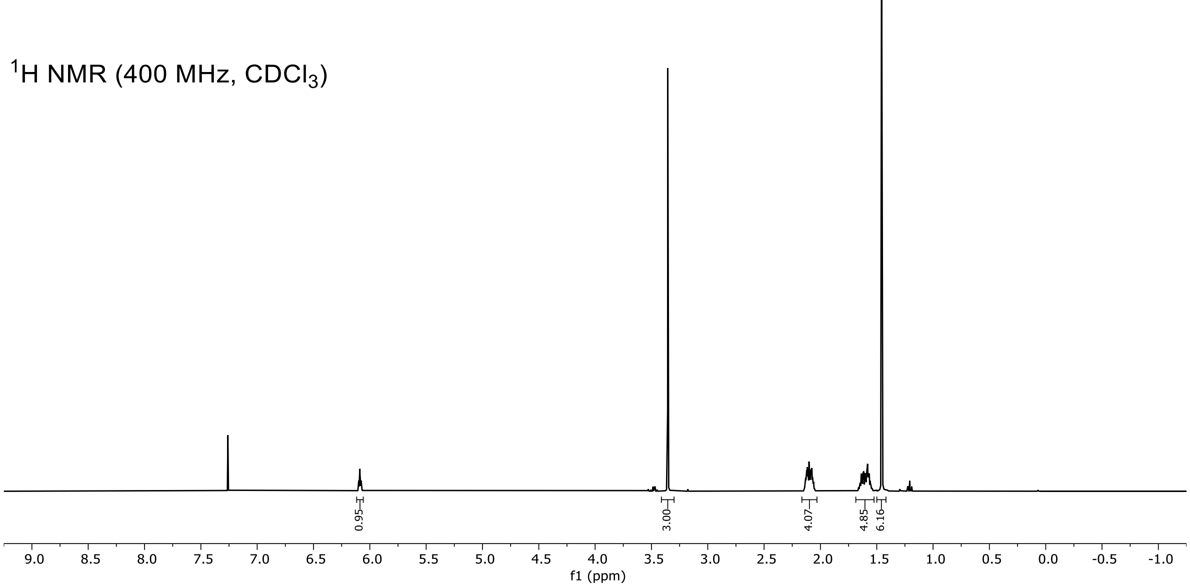

^{13}C NMR (101 MHz, $CDCl_3$)

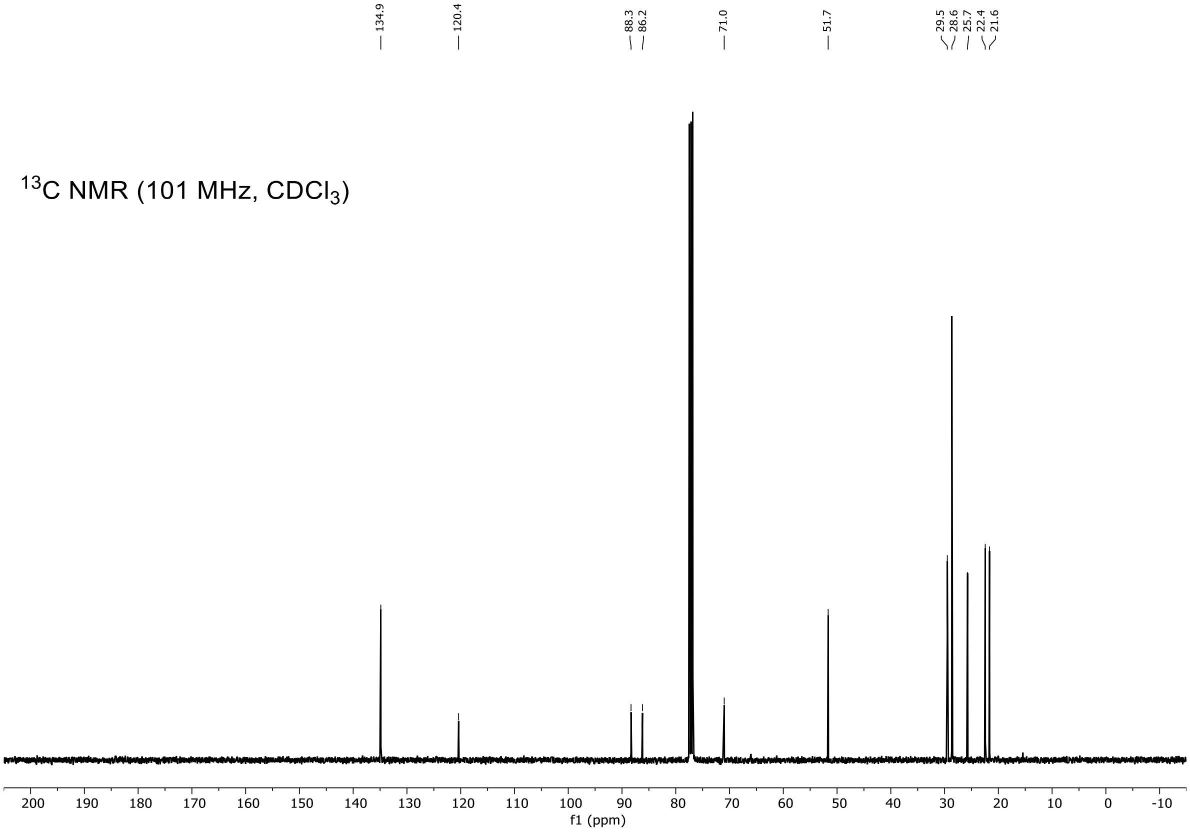

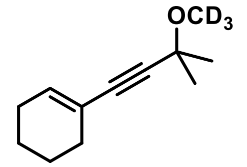
OCD3

[D$_3$]-5

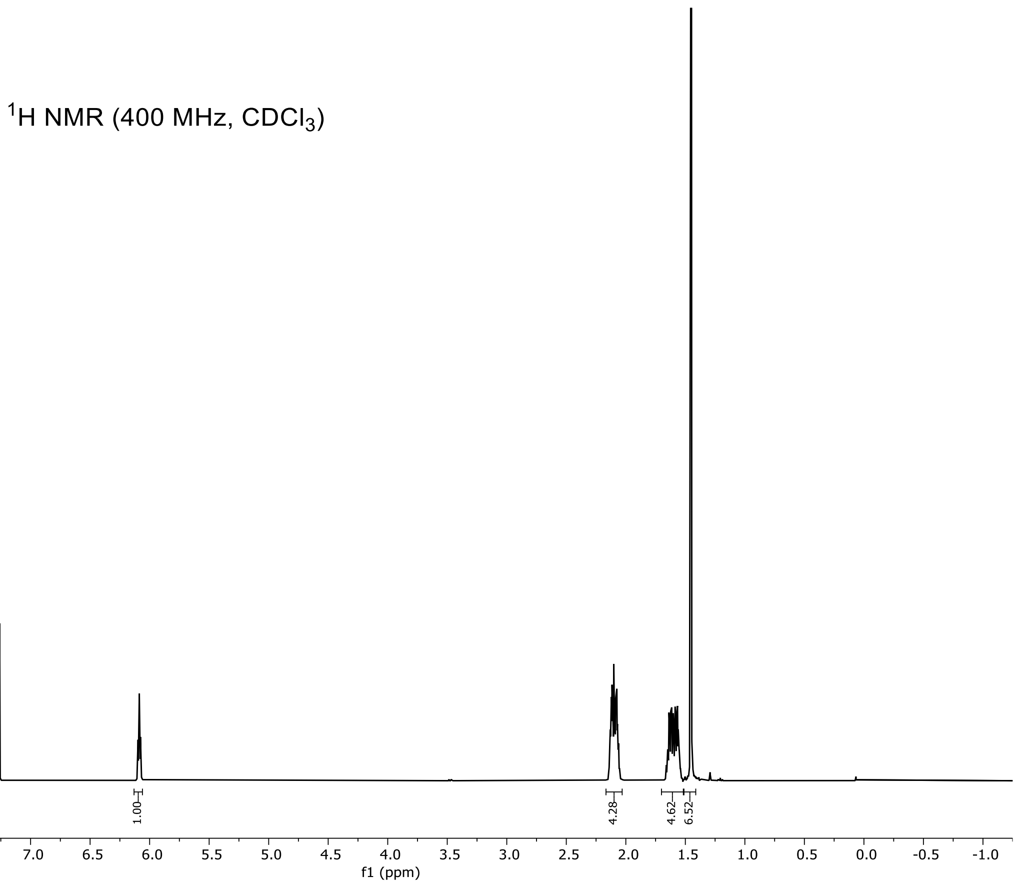
1H NMR (400 MHz, CDCl3)
1.00
4.28
4.62
6.52
9.0 8.5 8.0 7.5 7.0 6.5 6.0 5.5 5.0 4.5 4.0 3.5 3.0 2.5 2.0 1.5 1.0 0.5 0.0 -0.5 -1.0
f1 (ppm)

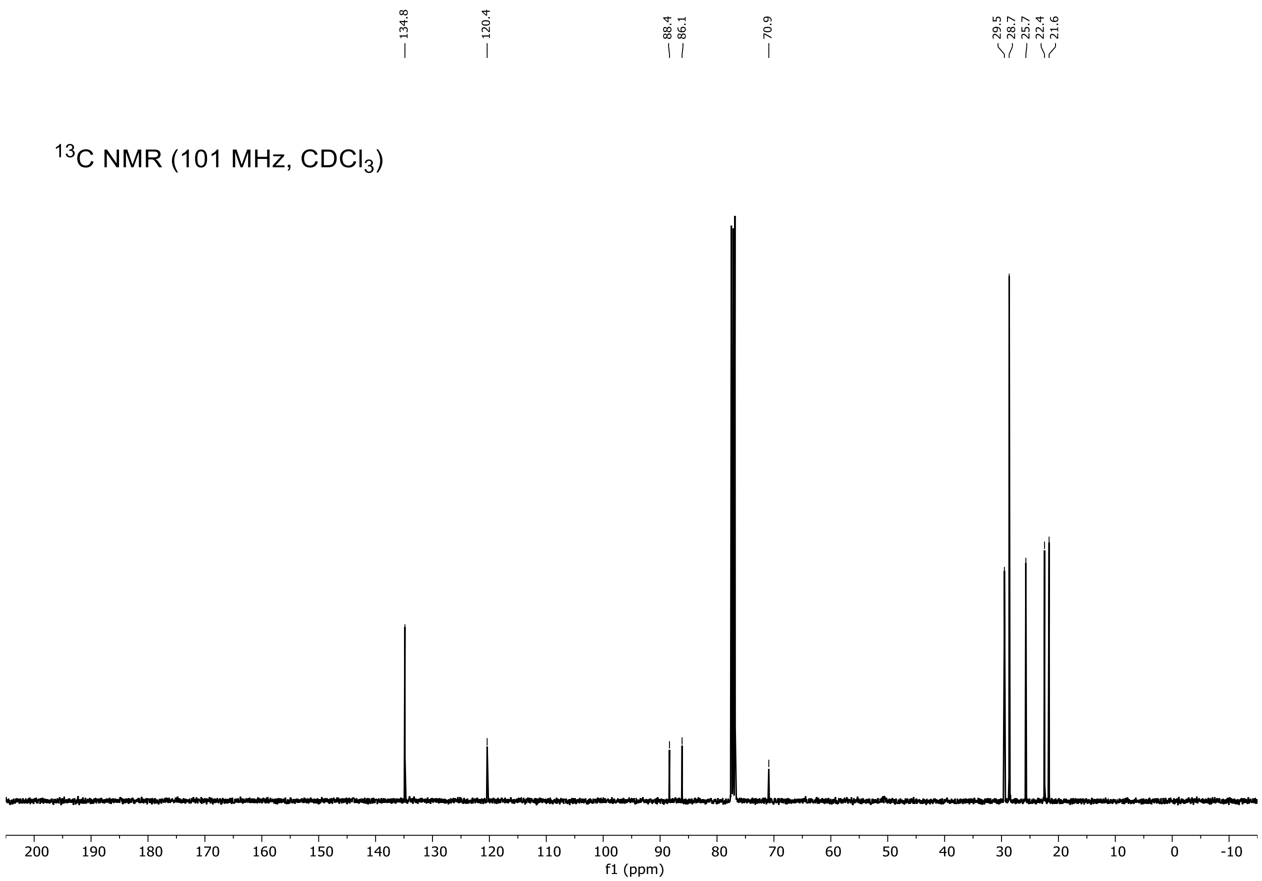
134.8
120.4
88.4
86.1
70.9
29.5
28.7
25.7
22.4
21.6
13C NMR (101 MHz, CDCl3)
200 190 180 170 160 150 140 130 120 110 100 90 80 70 60 50 40 30 20 10 0 -10
f1 (ppm)

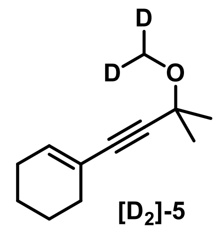
D
D
O

[D$_2$]-5

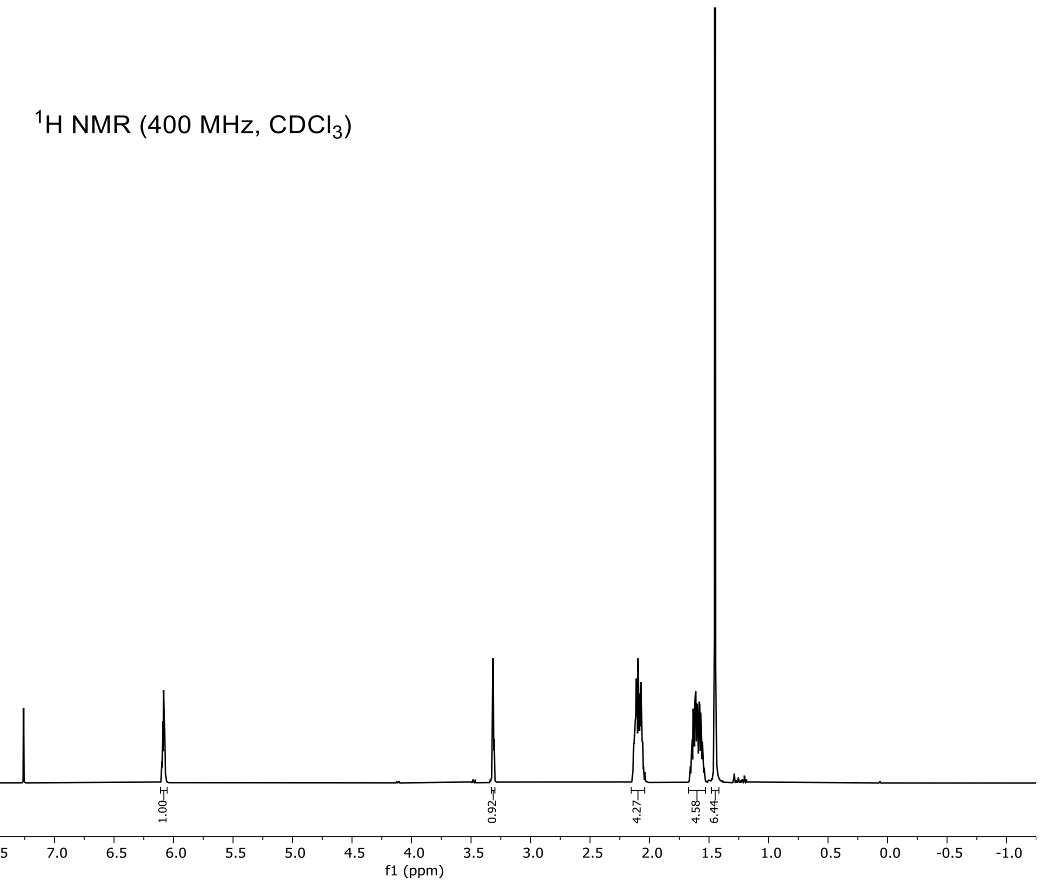
^{1}H NMR (400 MHz, CDCl$_3$)
1.00
0.92
4.27
4.58
6.44
9.0 8.5 8.0 7.5 7.0 6.5 6.0 5.5 5.0 4.5 4.0 3.5 3.0 2.5 2.0 1.5 1.0 0.5 0.0 -0.5 -1.0
f1 (ppm)

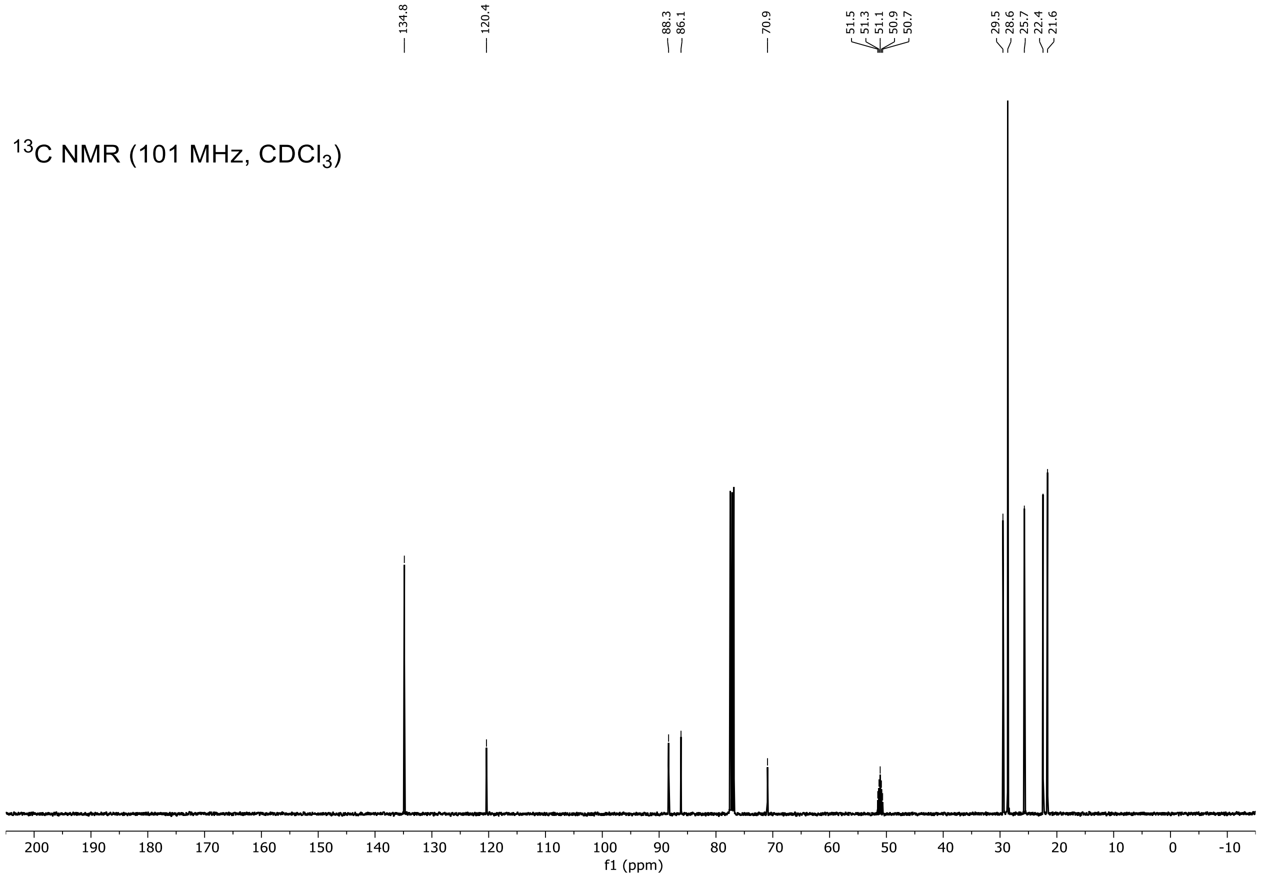
134.8
120.4
88.3
86.1
70.9
51.5
51.3
51.1
50.9
50.7
29.5
28.6
25.7
22.4
21.6
^{13}C NMR (101 MHz, CDCl$_3$)
200 190 180 170 160 150 140 130 120 110 100 90 80 70 60 50 40 30 20 10 0 -10
f1 (ppm)

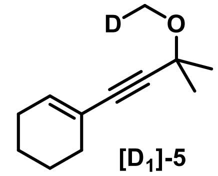

[D$_1$]-5

^{1}H NMR (400 MHz, $CDCl_3$)

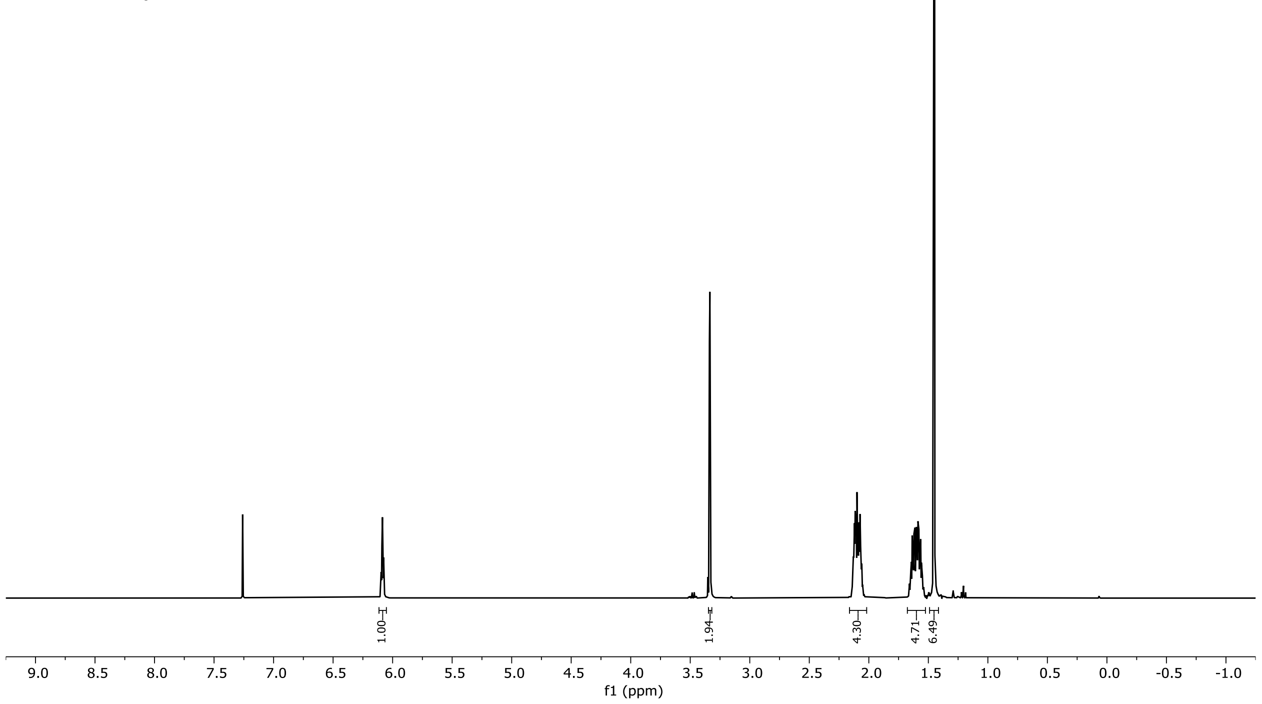

^{13}C NMR (101 MHz, $CDCl_3$)

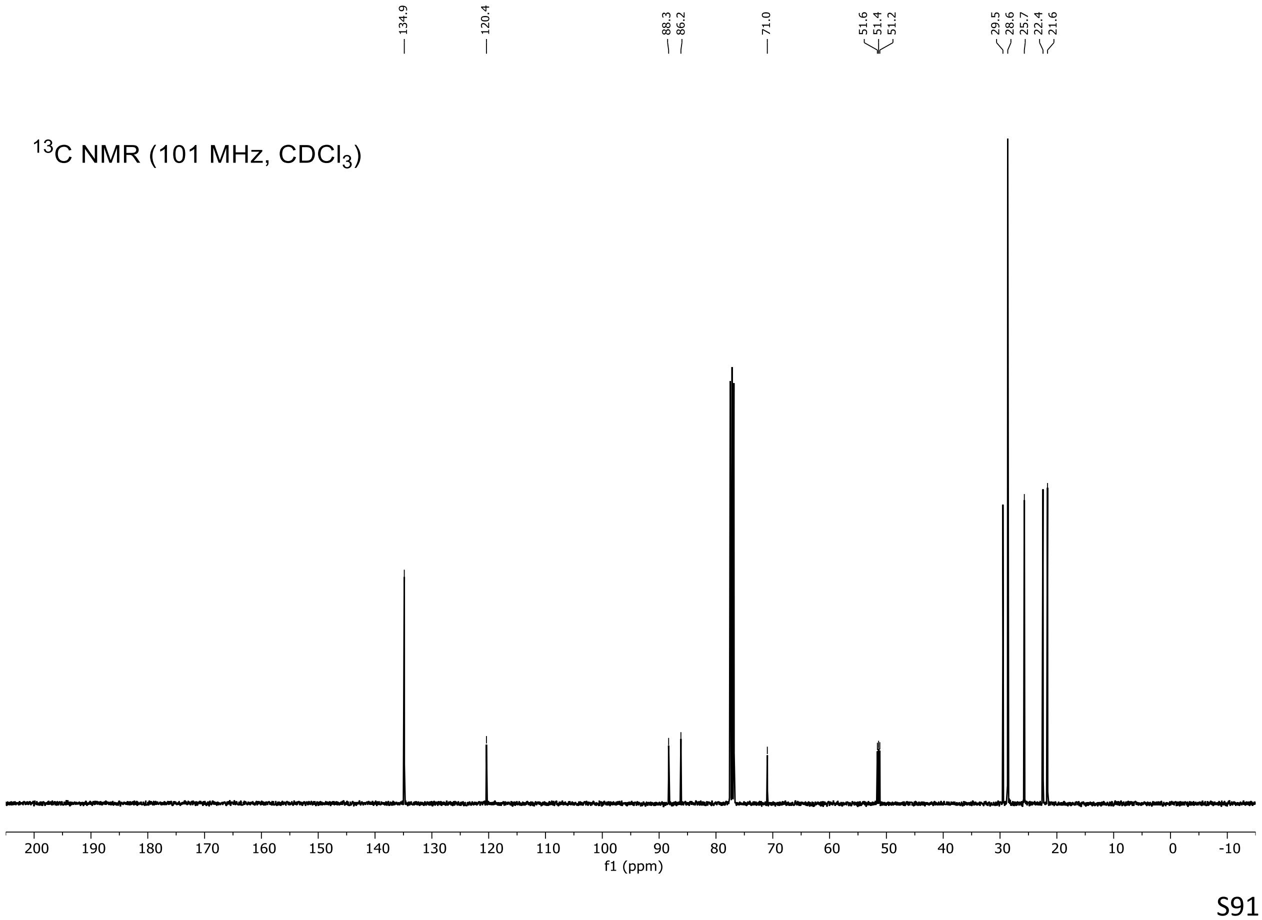

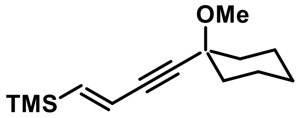

8

^{1}H NMR (400 MHz, $CDCl_3$)

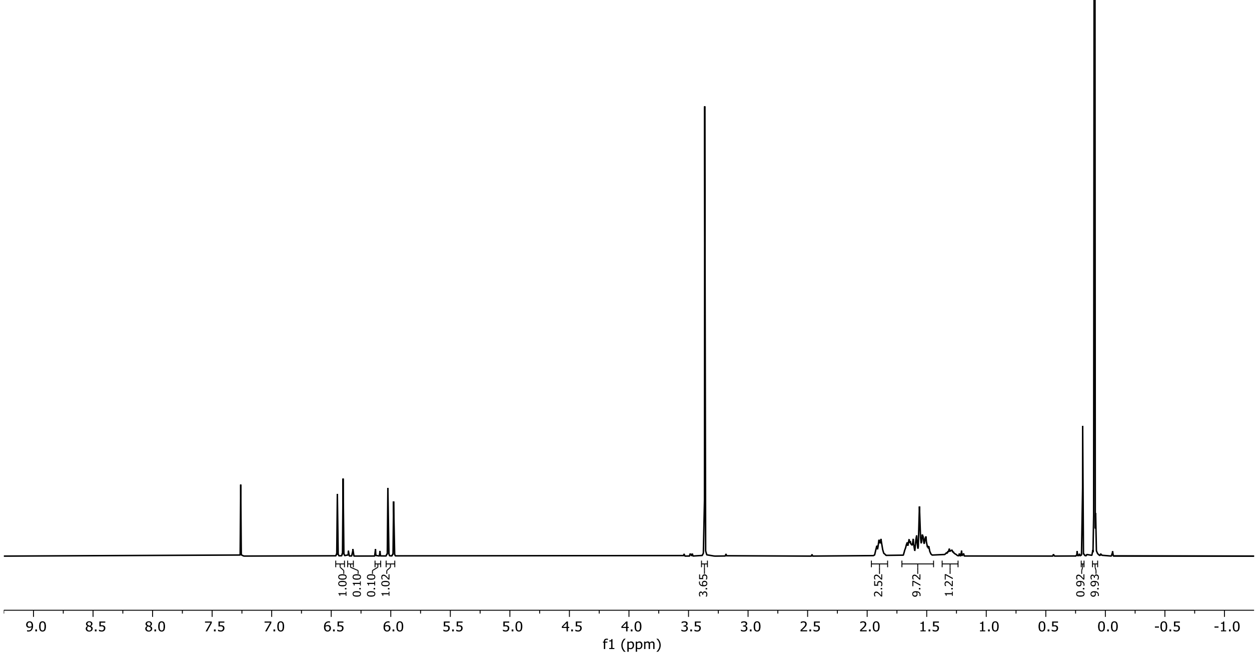

^{13}C NMR (101 MHz, $CDCl_3$)

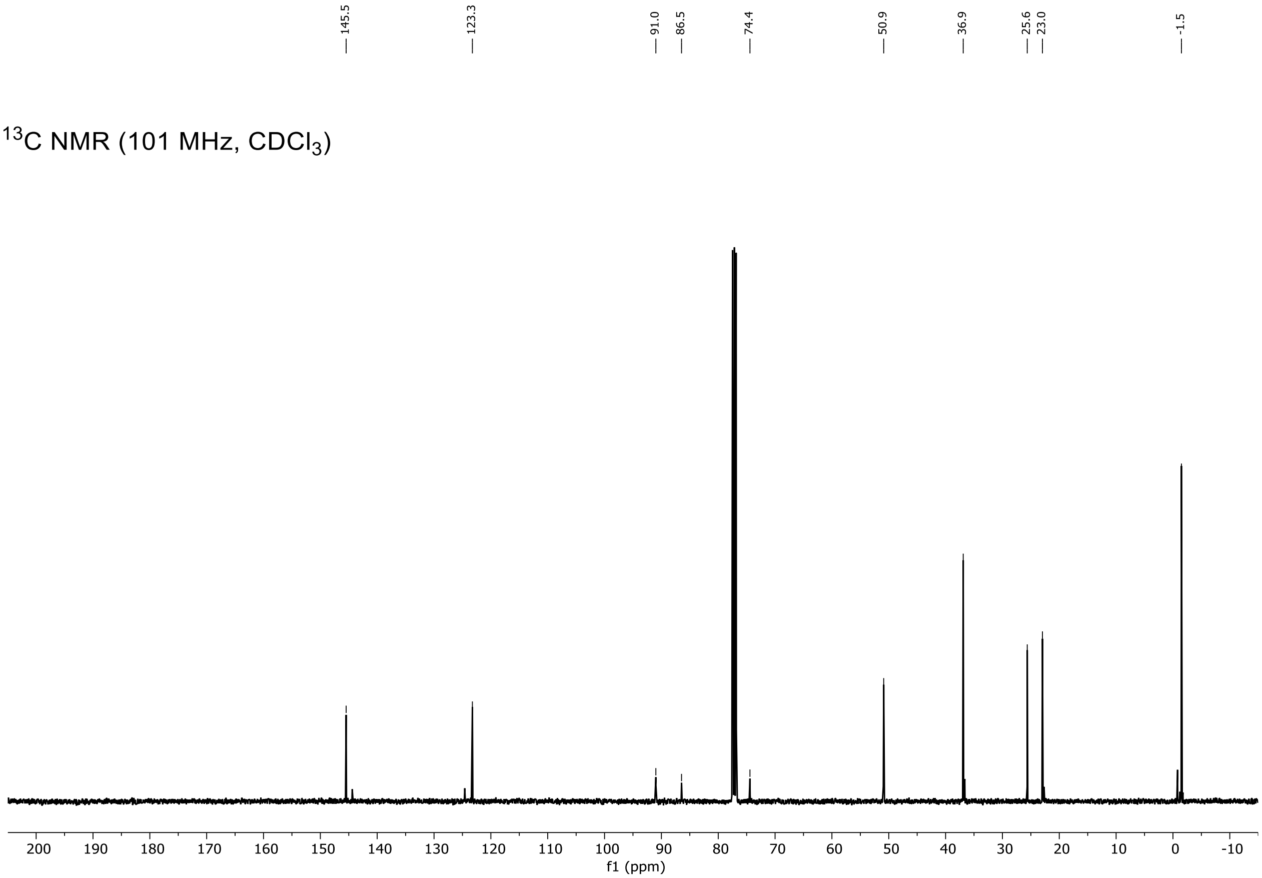

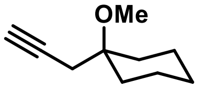

9

^{1}H NMR (400 MHz, $CDCl_3$)

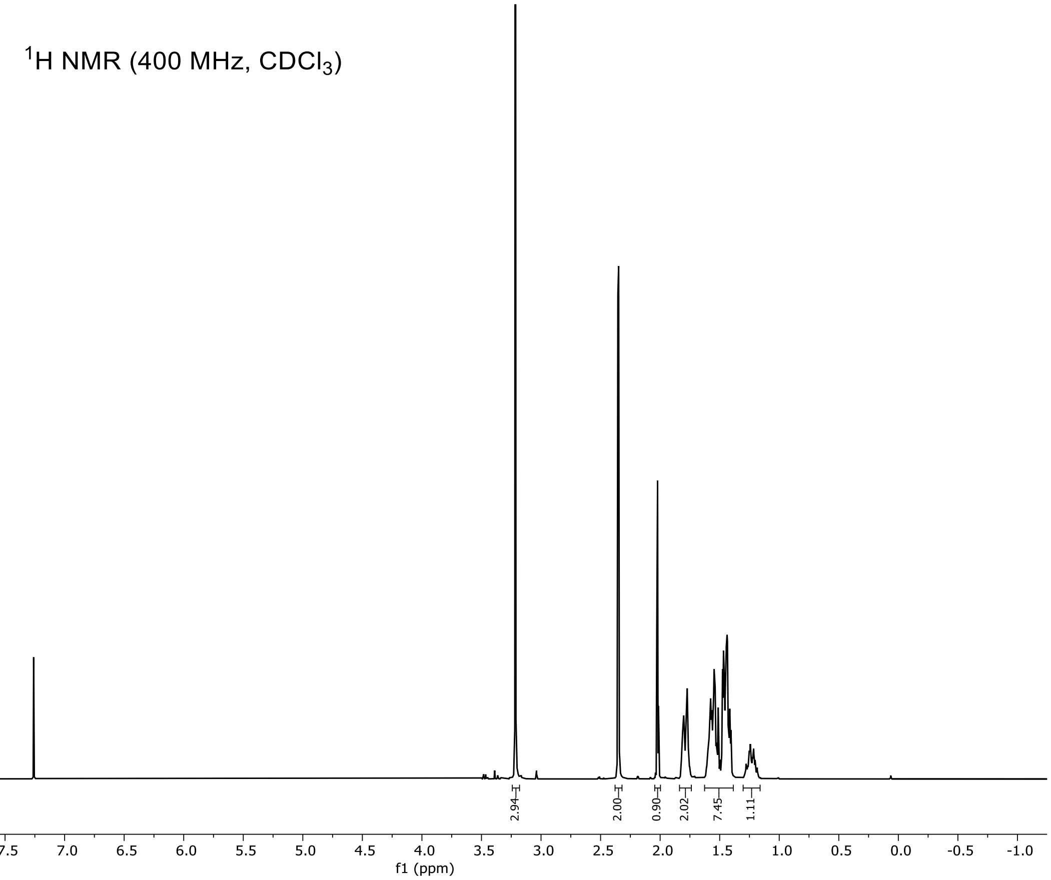

^{13}C NMR (101 MHz, $CDCl_3$)

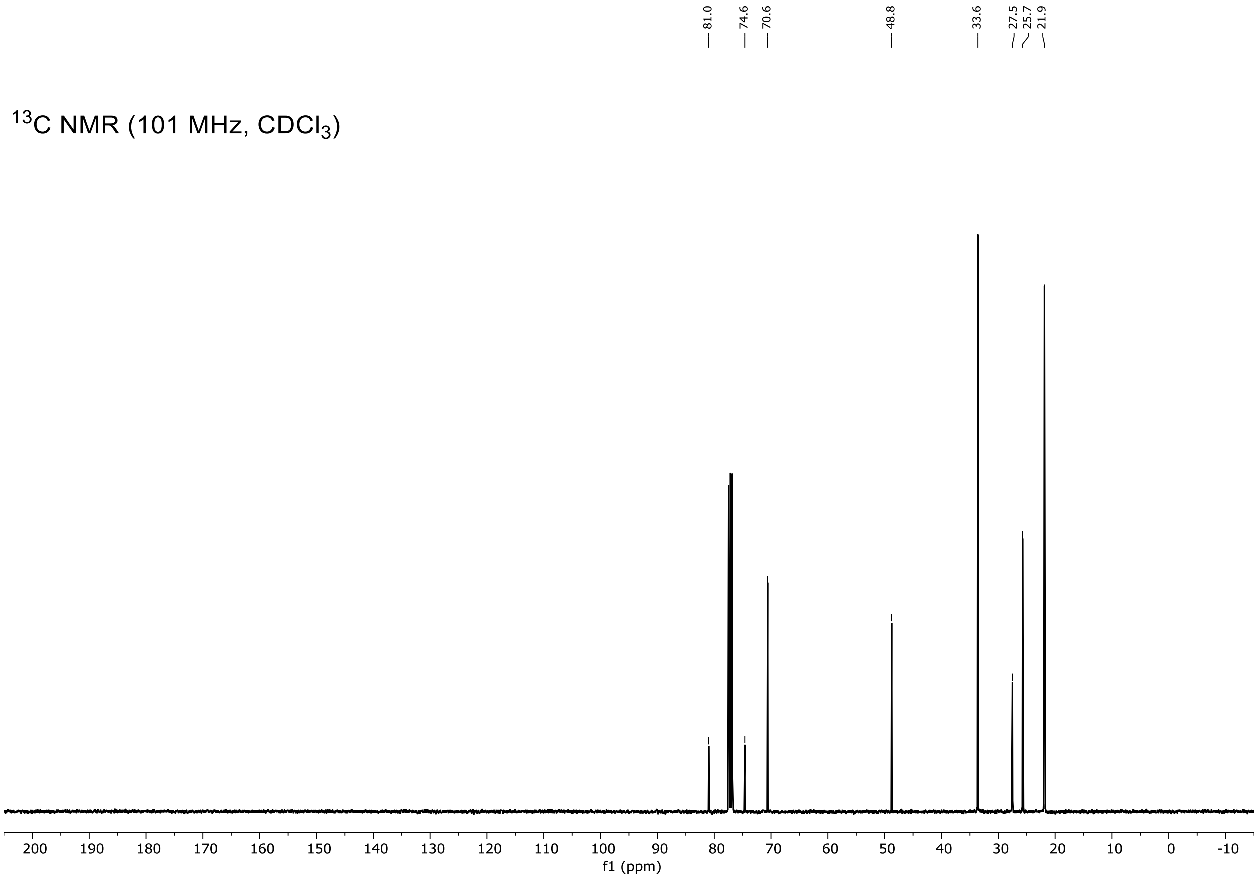

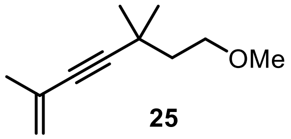

25

^{1}H NMR (400 MHz, $CDCl_3$)

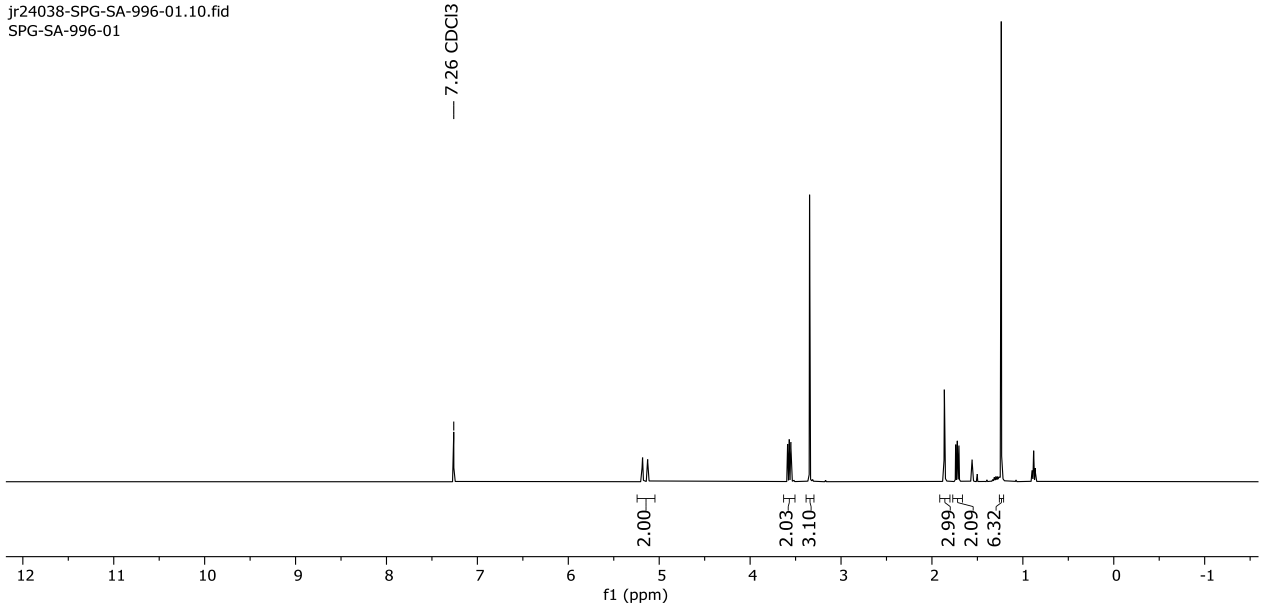

^{13}C NMR (101 MHz, $CDCl_3$)

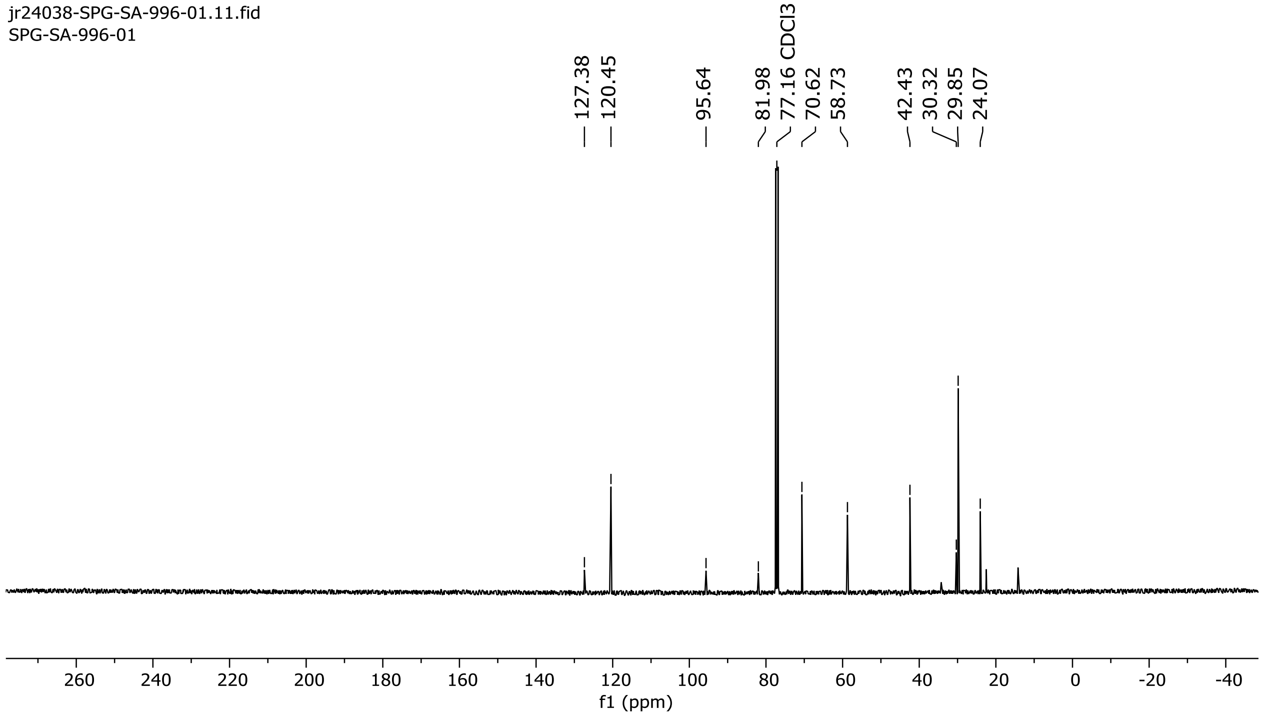

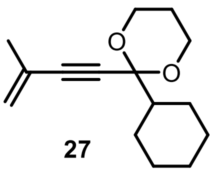

27

^{1}H NMR (400 MHz, C_6D_6)

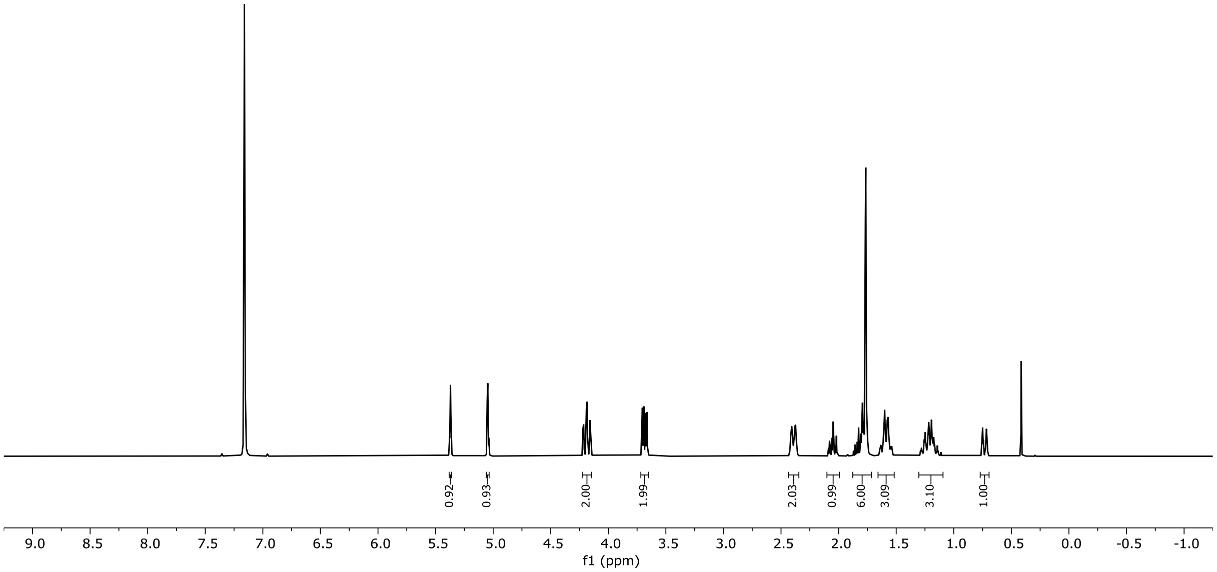

^{13}C NMR (101 MHz, C_6D_6)

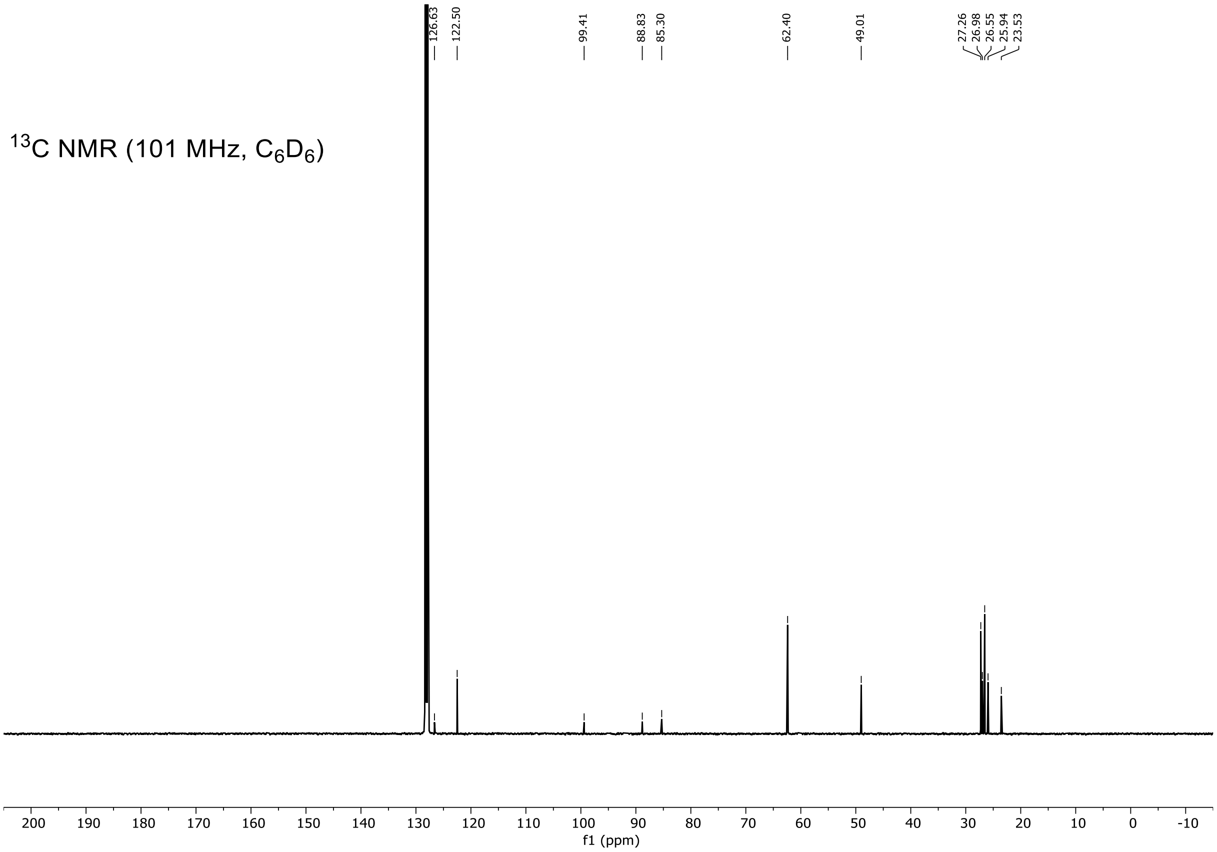

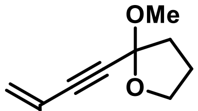

28a

^{1}H NMR (400 MHz, C_6D_6)

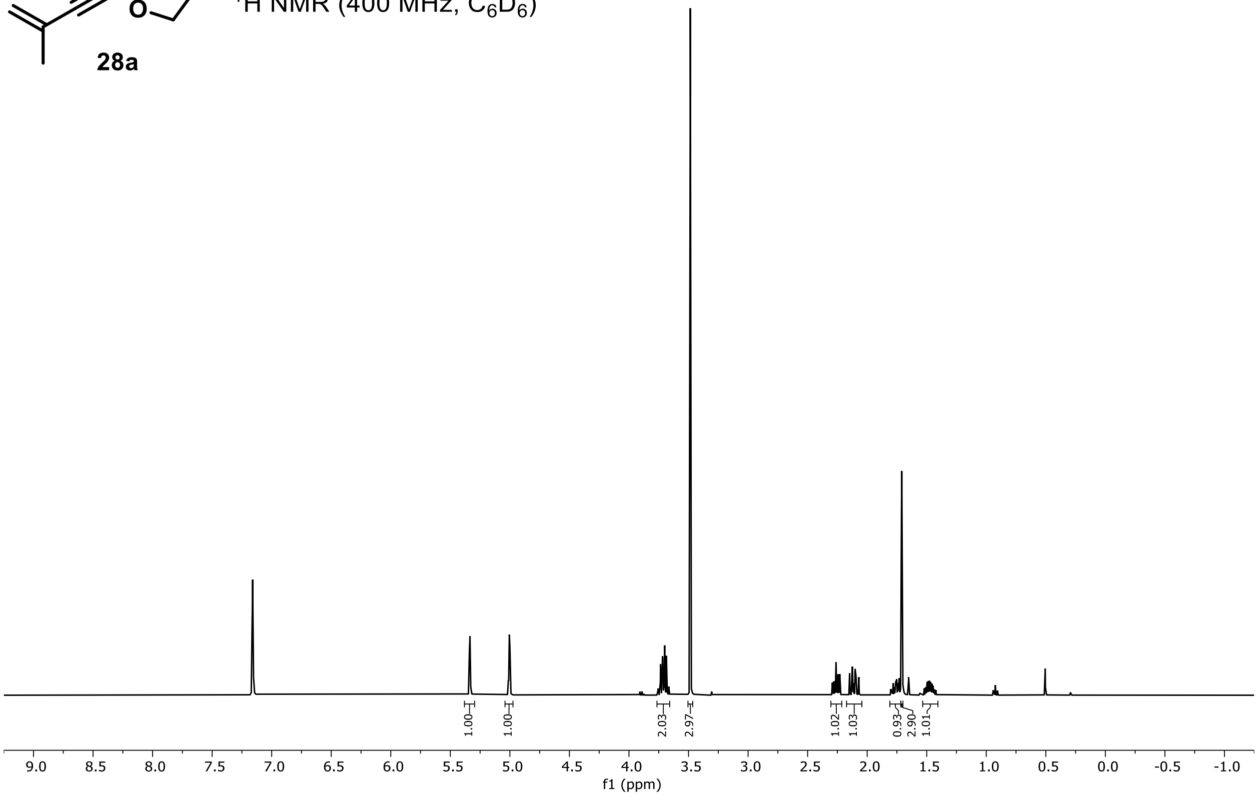

^{13}C NMR (101 MHz, C_6D_6)

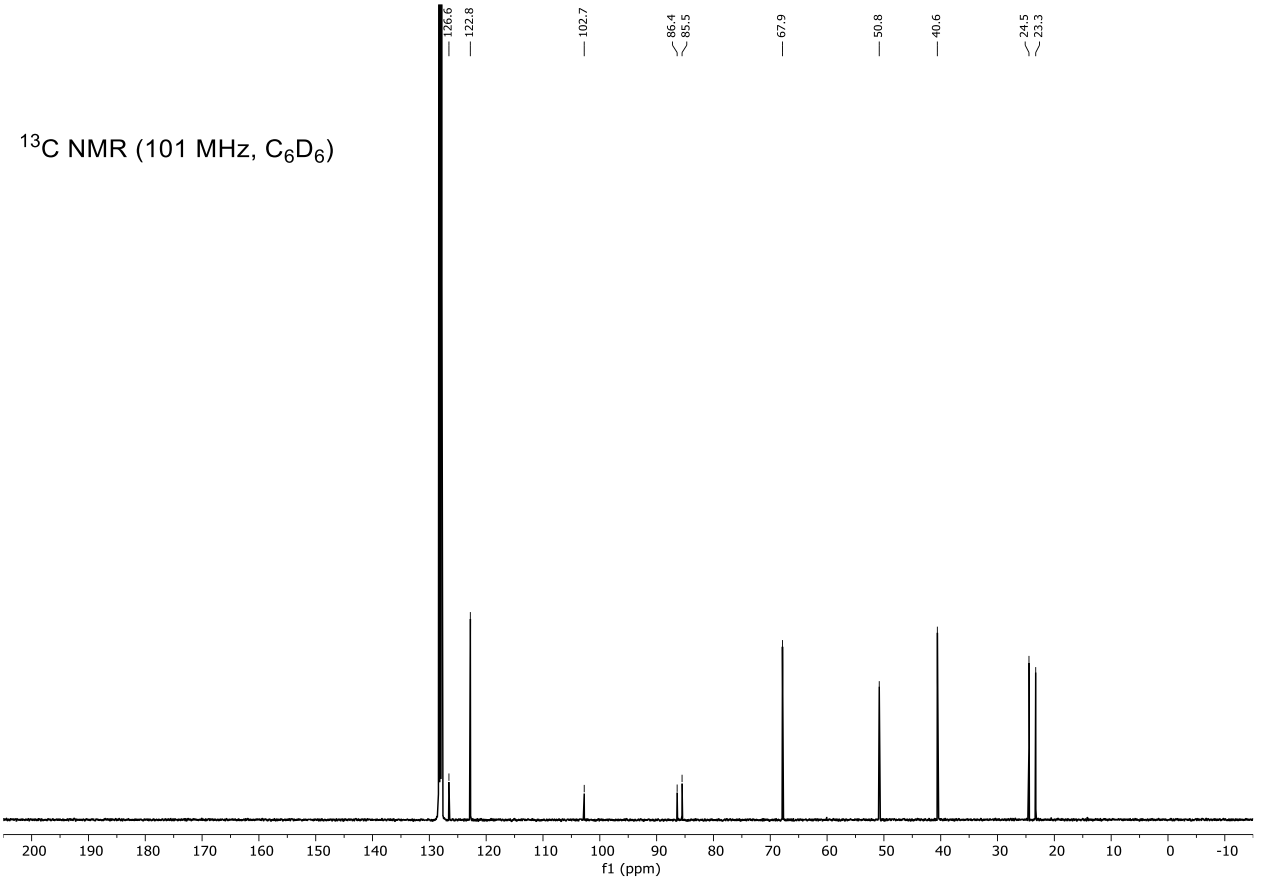

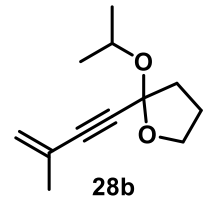

28b

^{1}H NMR (400 MHz, C_6D_6)

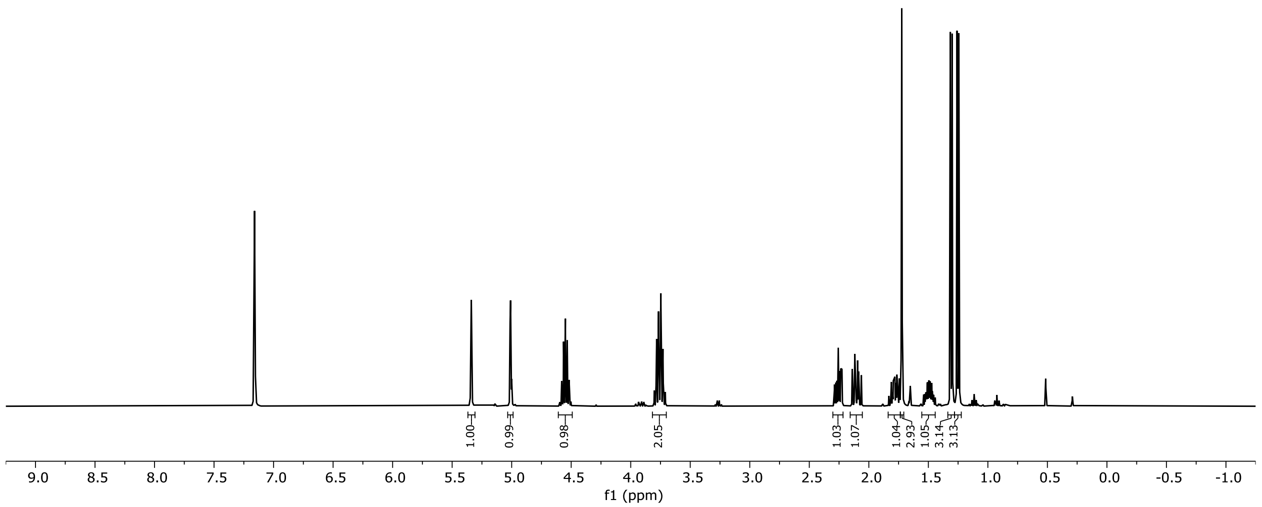

^{13}C NMR (101 MHz, C_6D_6)

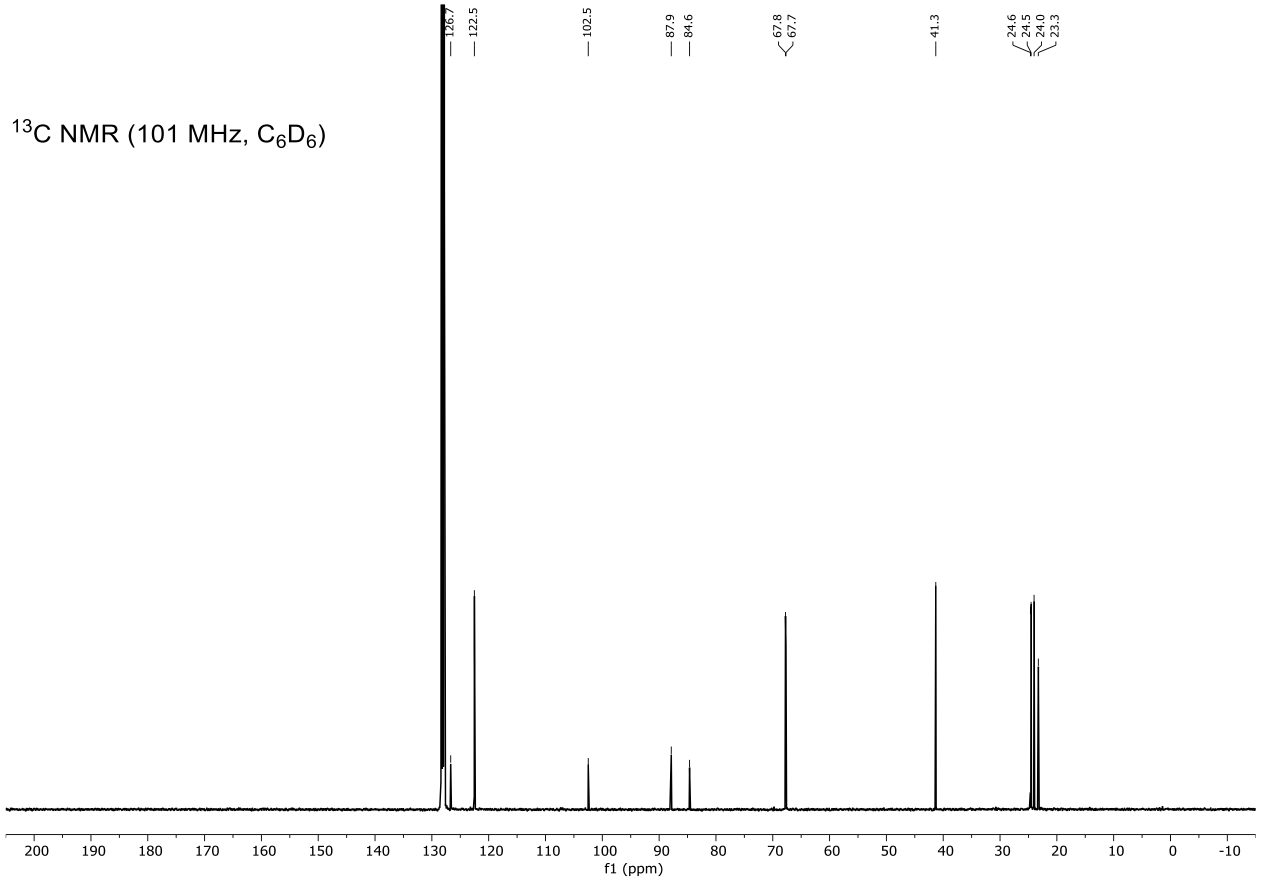

sr27025-WEU-WA-180-01.10.fid
sr27025-WEU-WA-180-01 CRUDE

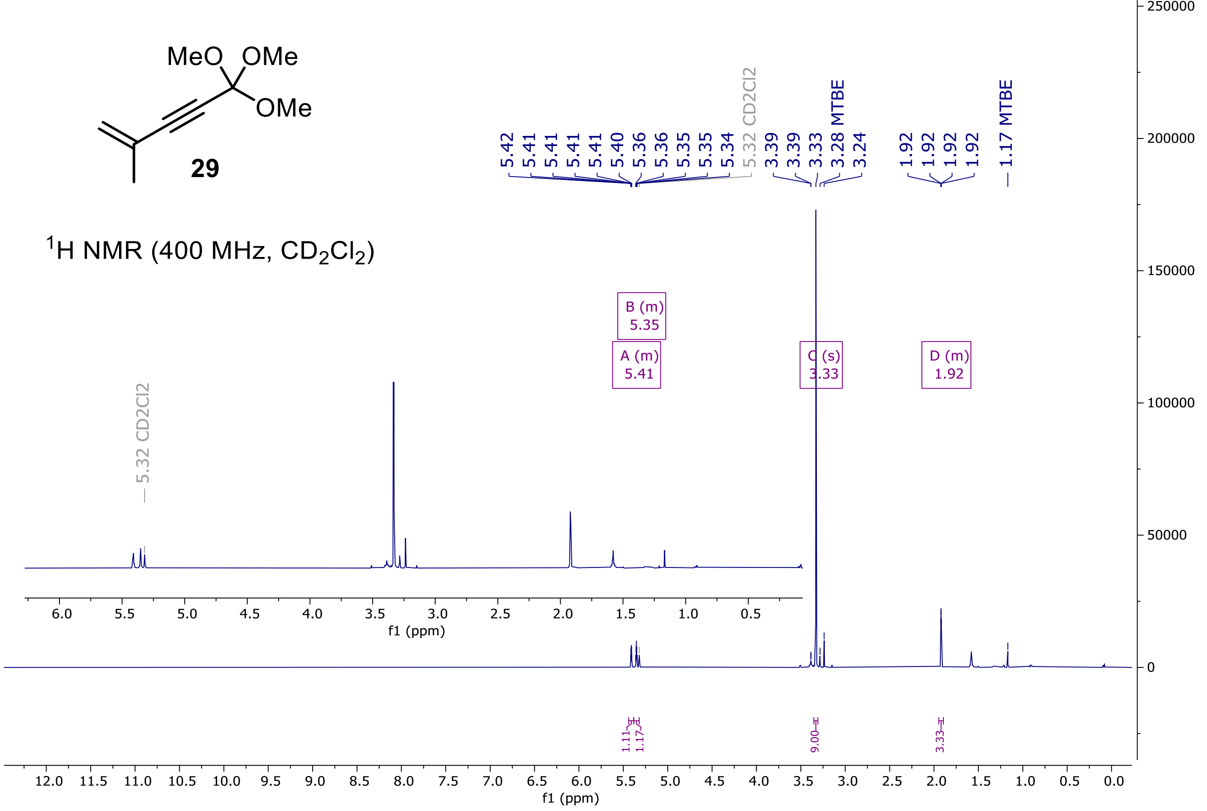

sr27025-WEU-WA-180-01.11.fid
sr27025-WEU-WA-180-01 CRUDE

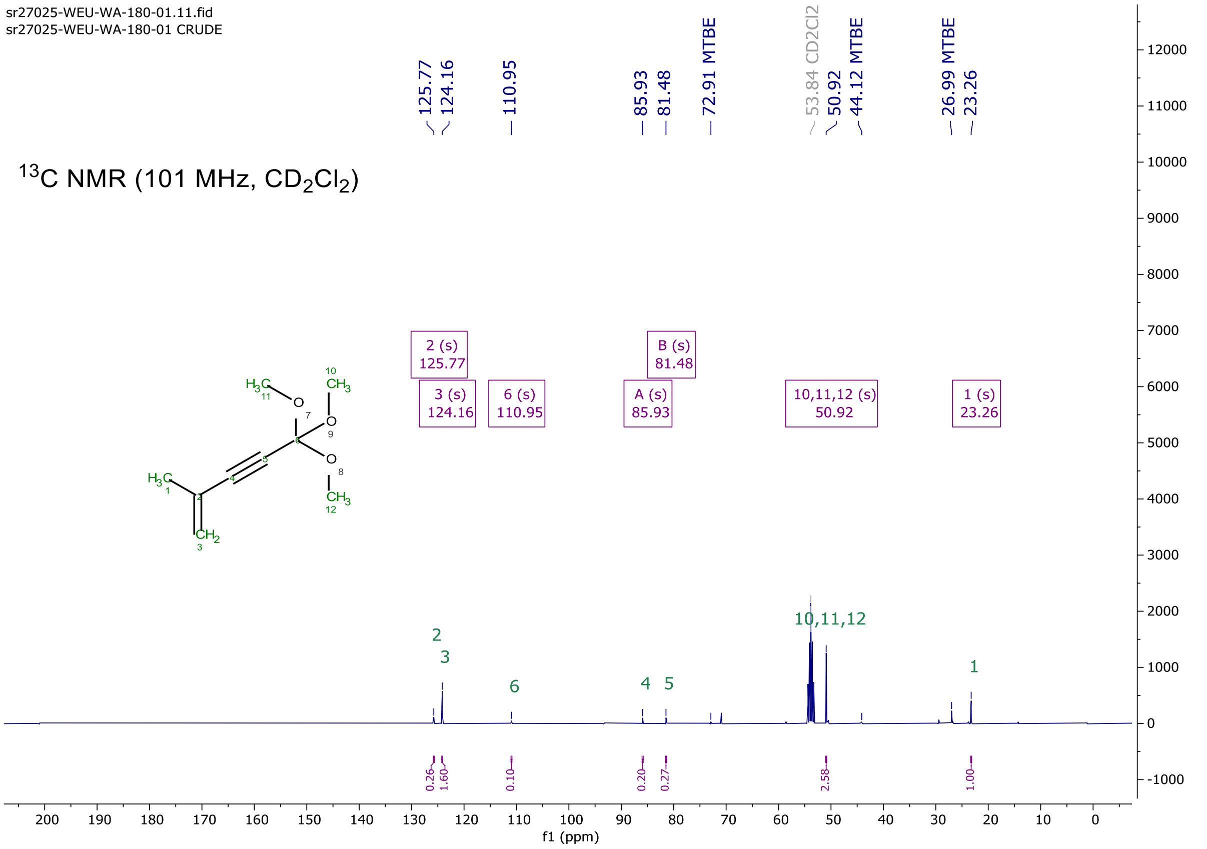

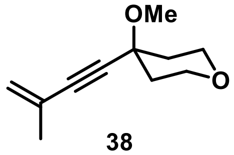

38

^{1}H NMR (400 MHz, $CDCl_3$)

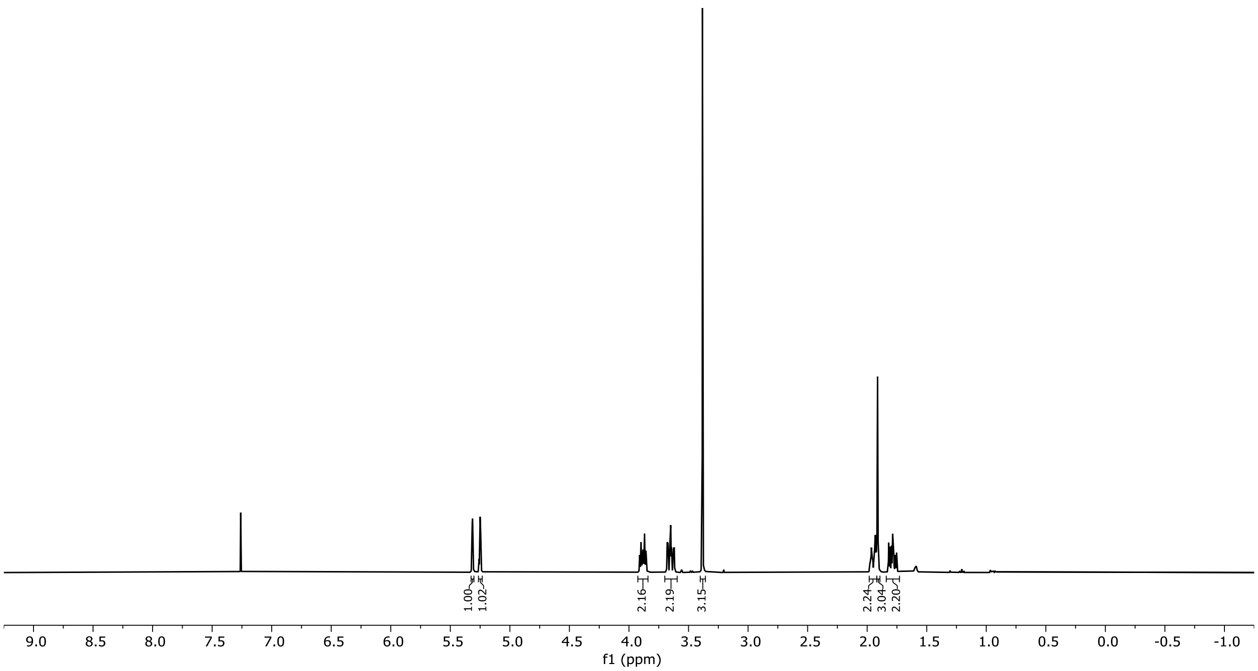

^{13}C NMR (101 MHz, $CDCl_3$)

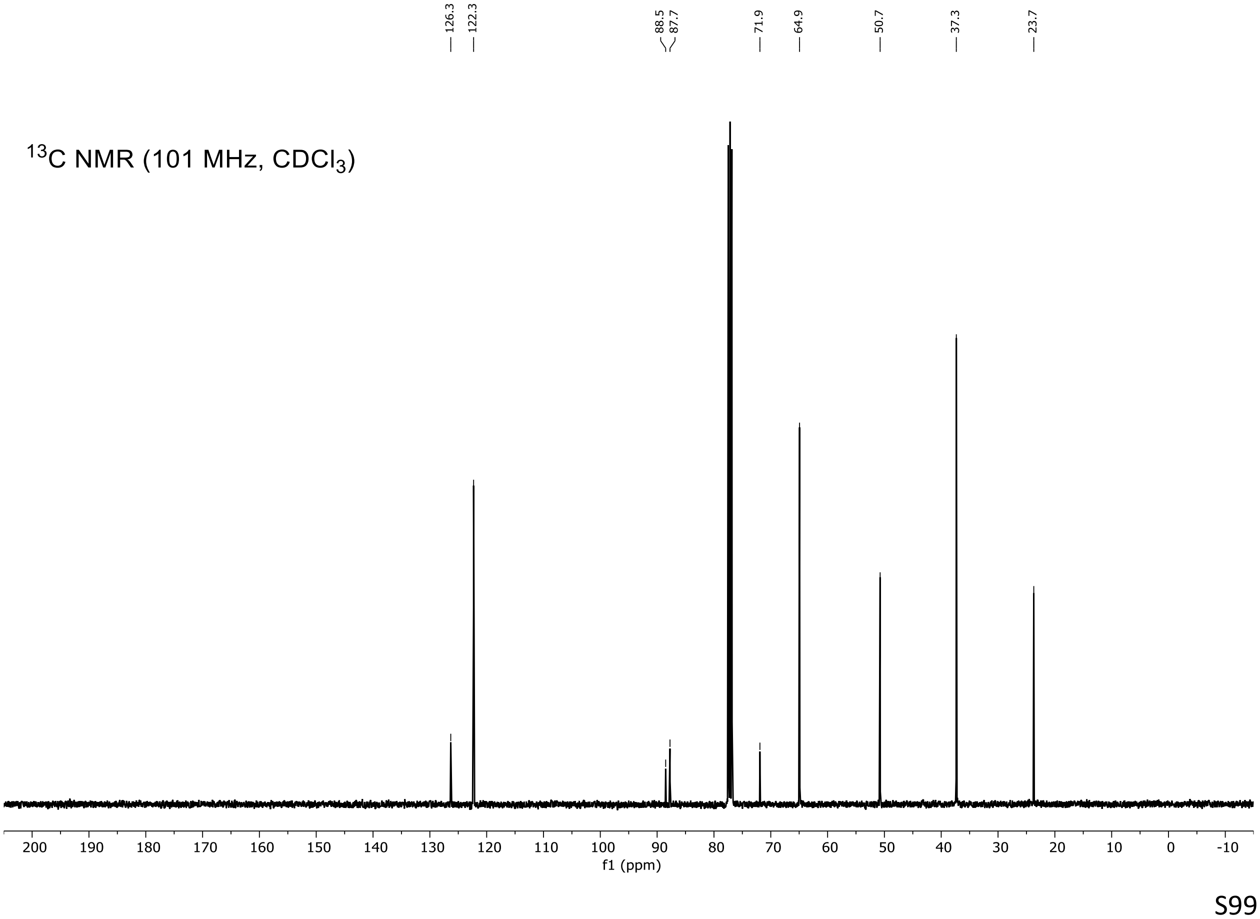

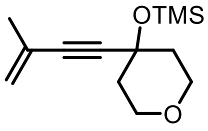

39

^{1}H NMR (400 MHz, $CDCl_3$)

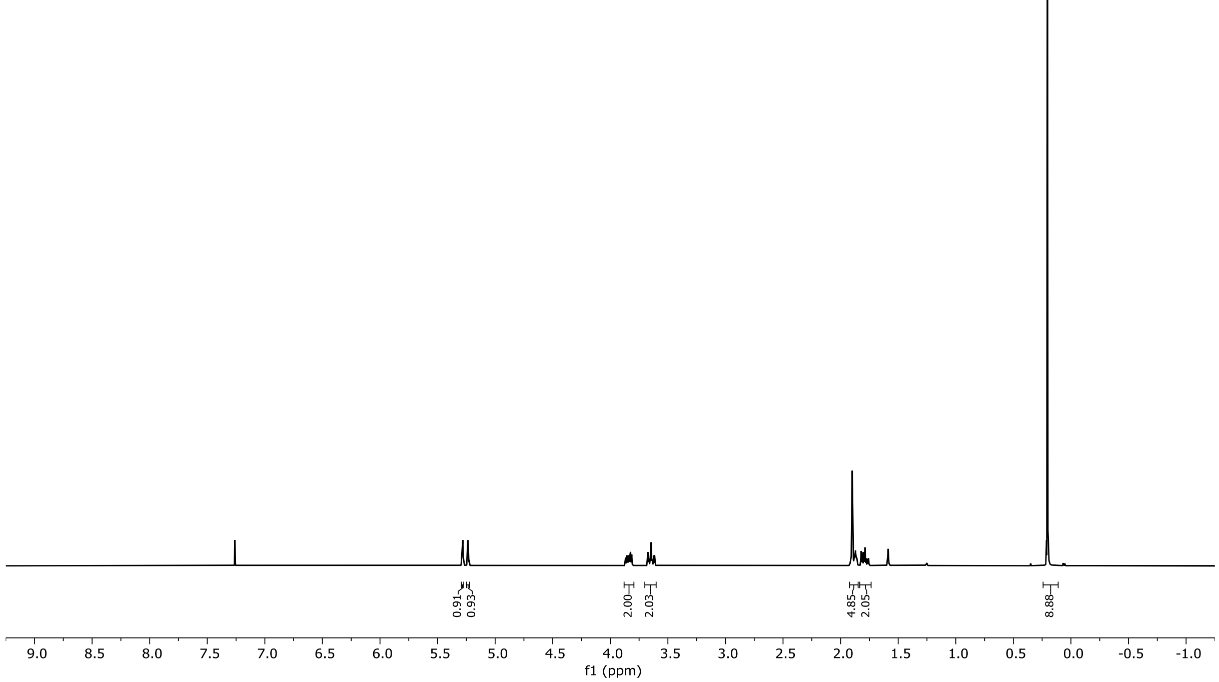

^{13}C NMR (101 MHz, $CDCl_3$)

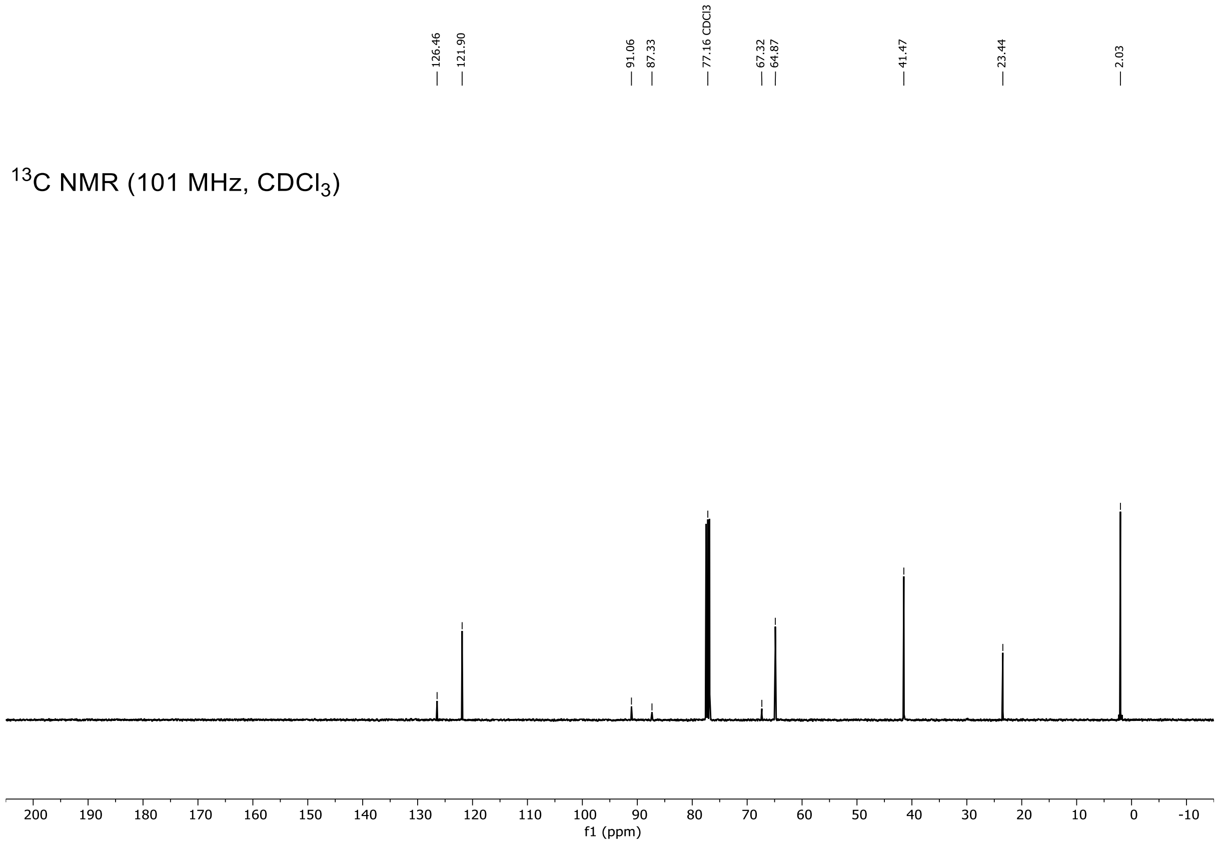

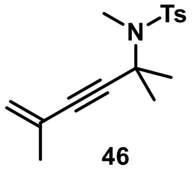

46

^{1}H NMR (400 MHz, $CDCl_3$)

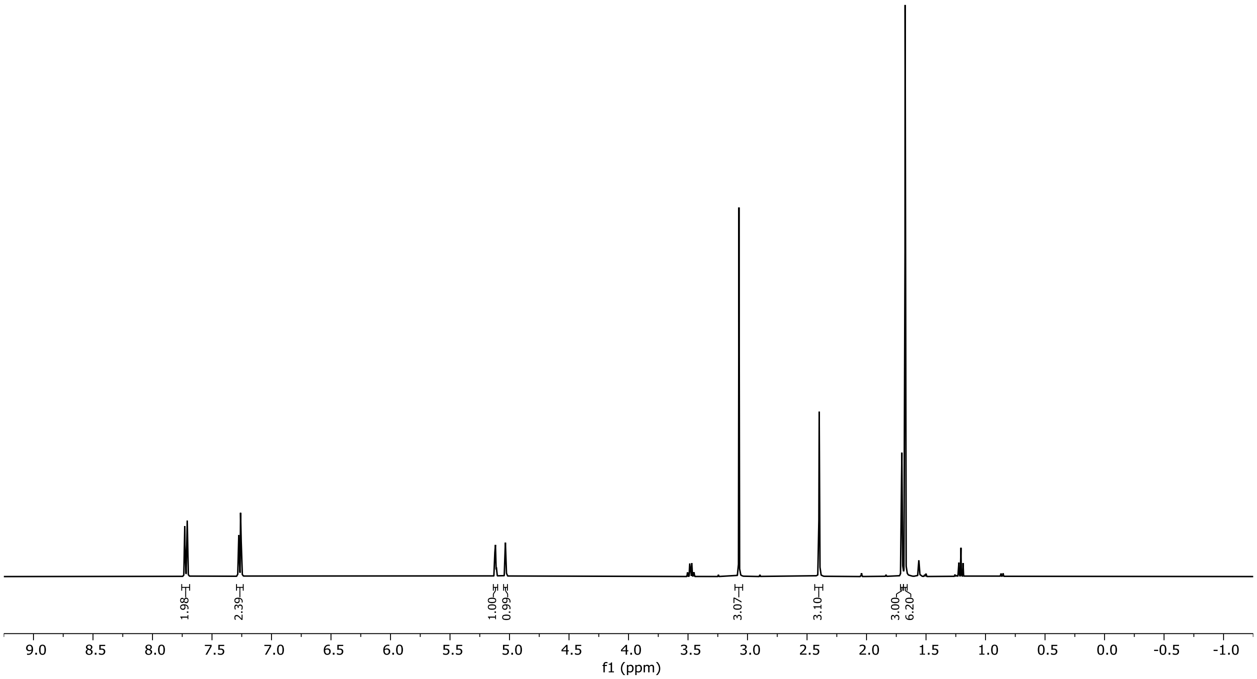

^{13}C NMR (101 MHz, $CDCl_3$)

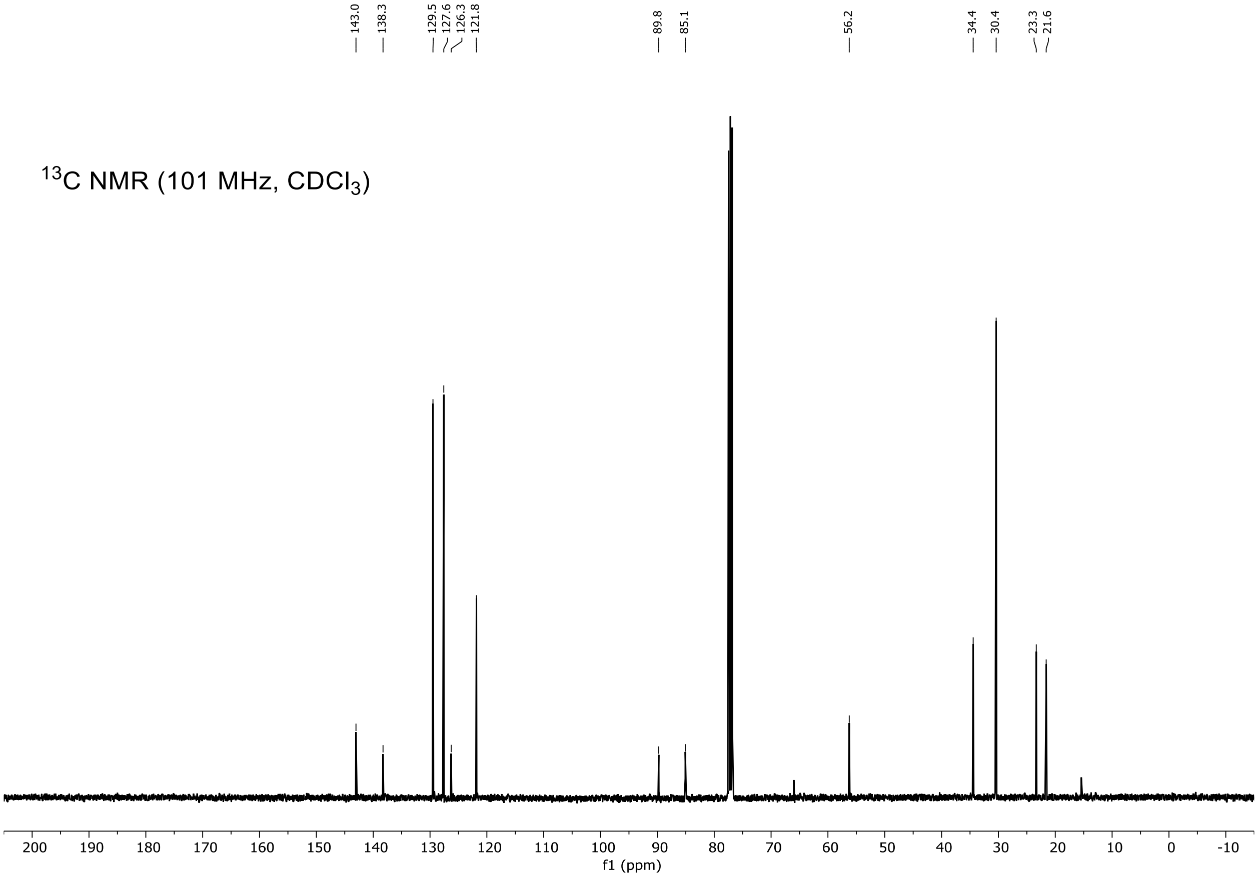

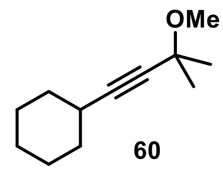

60

^{1}H NMR (400 MHz, $CDCl_3$)

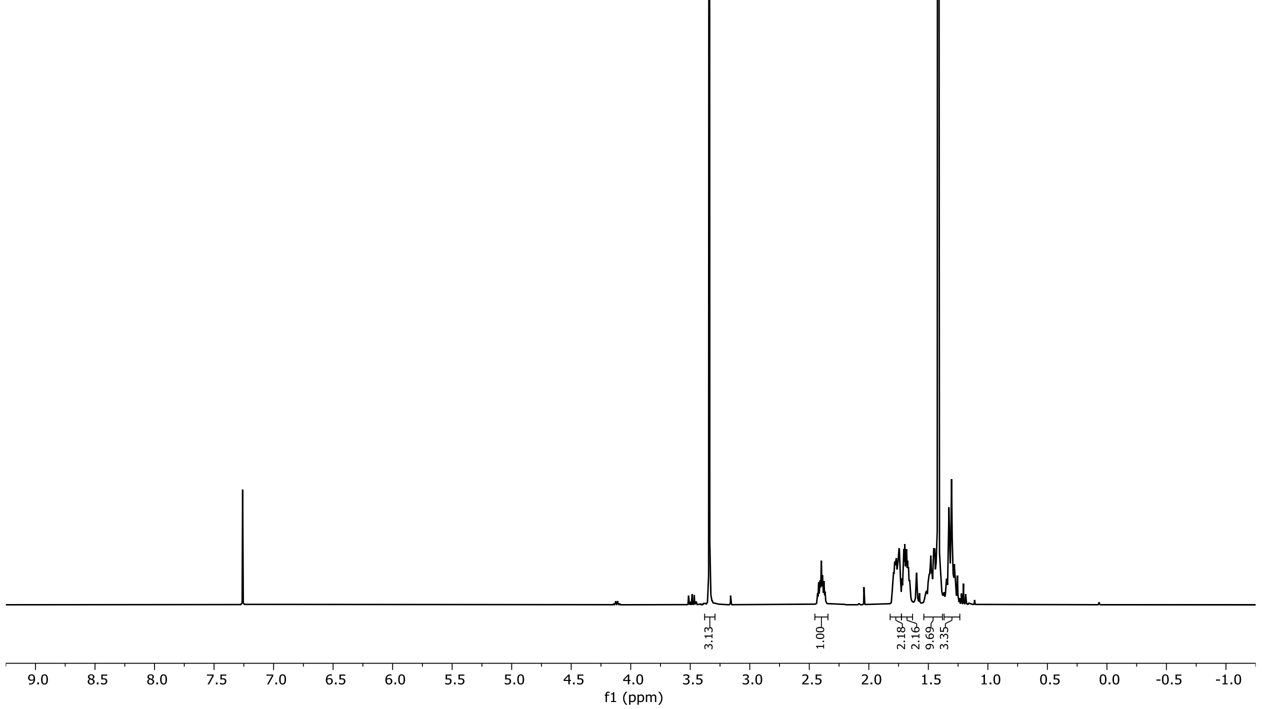

^{13}C NMR (101 MHz, $CDCl_3$)

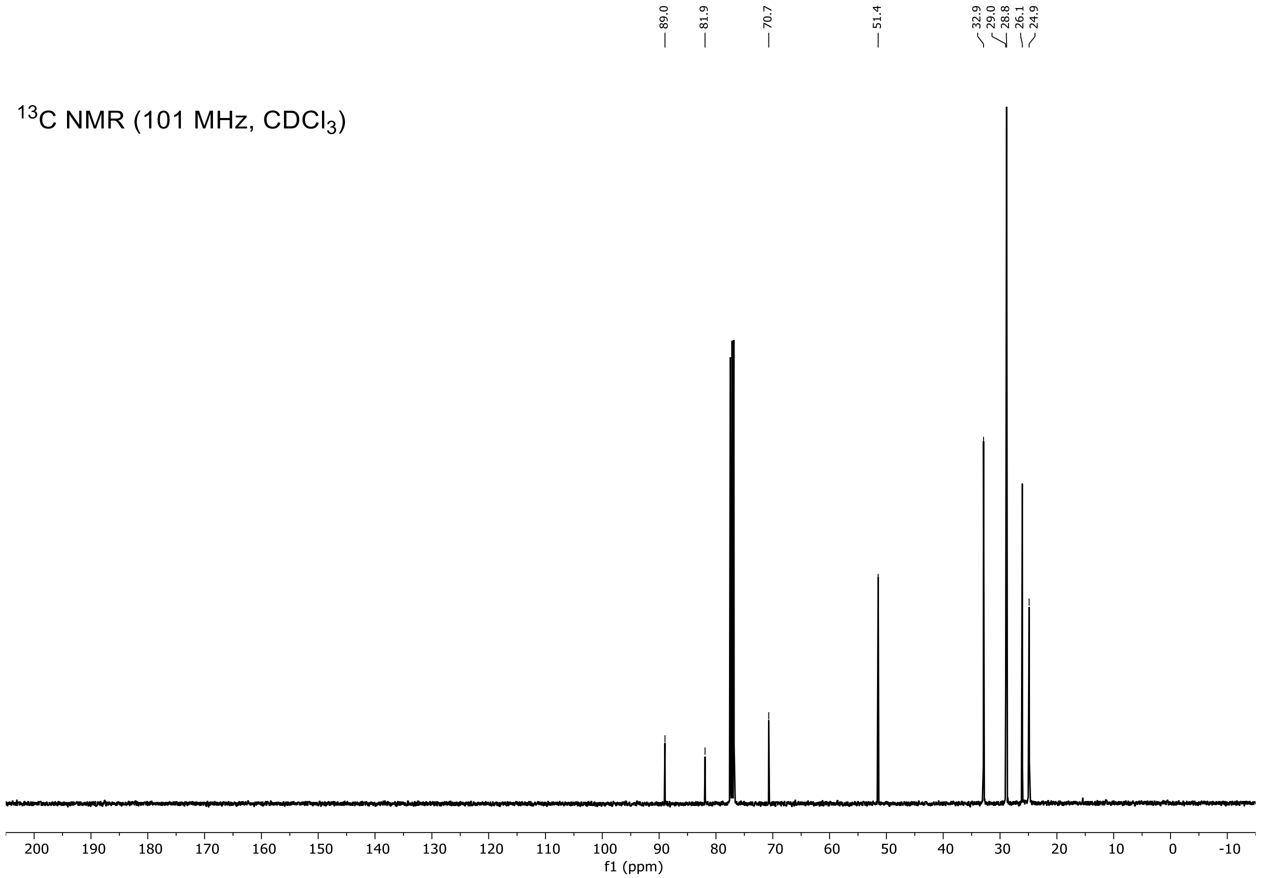

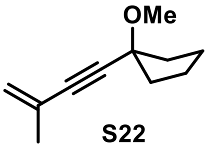

S22

^{1}H NMR (400 MHz, $CDCl_3$)

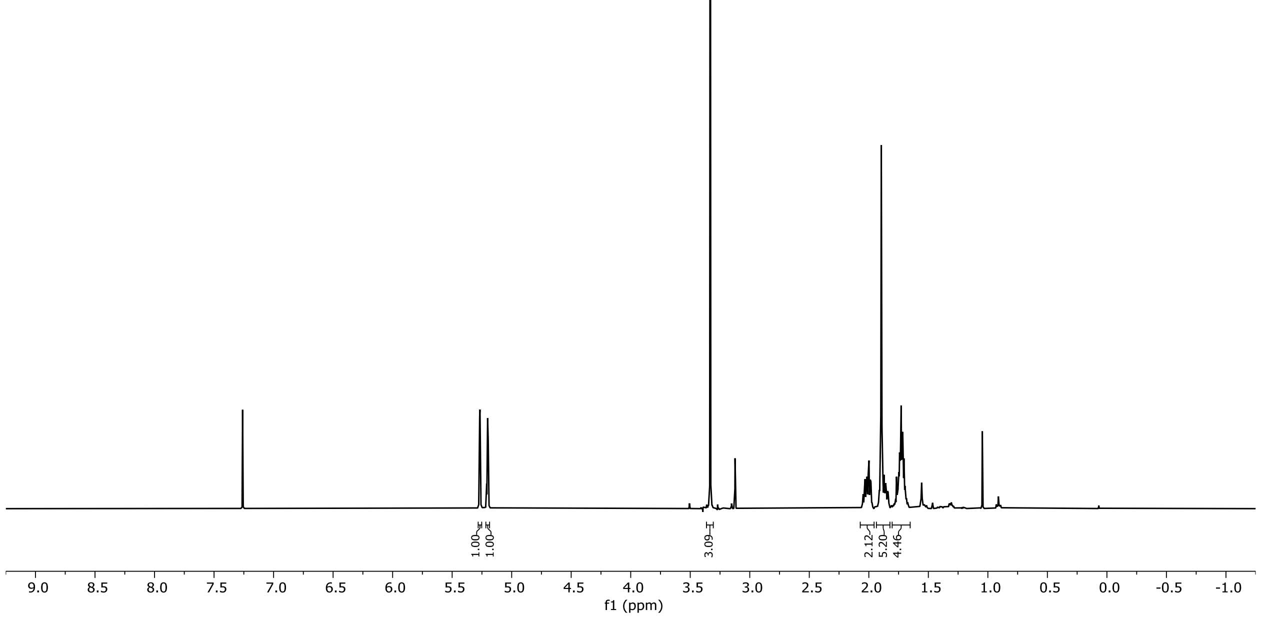

^{13}C NMR (101 MHz, $CDCl_3$)

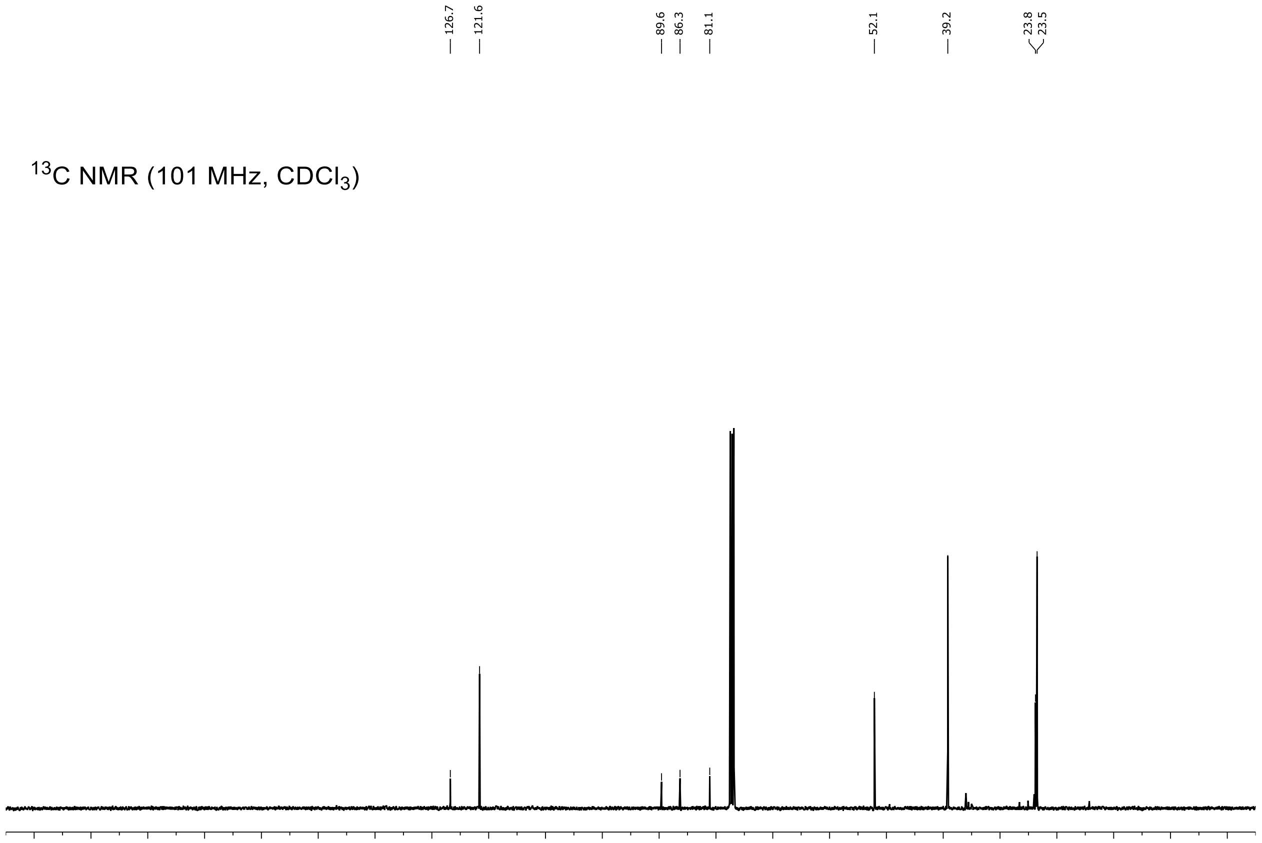

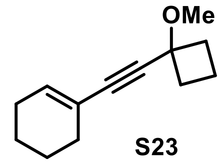

S23

^{1}H NMR (400 MHz, $CDCl_3$)

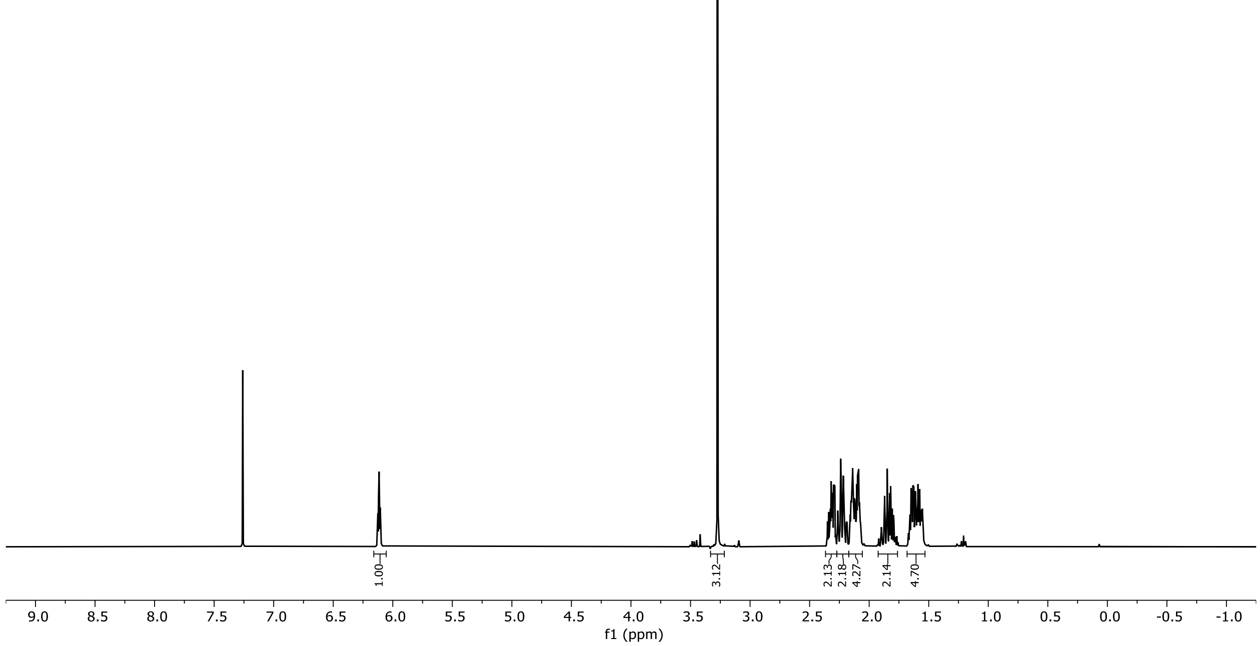

^{13}C NMR (101 MHz, $CDCl_3$)

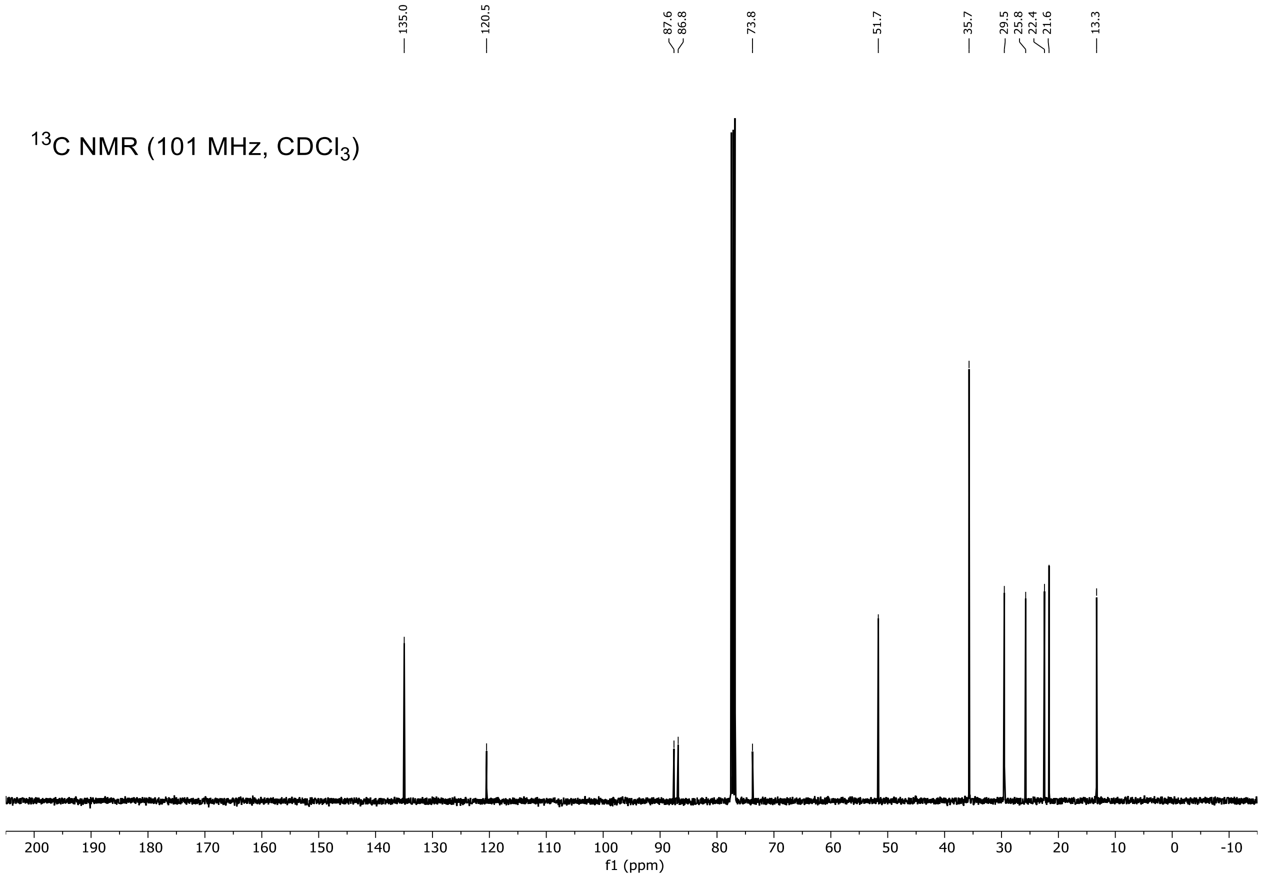

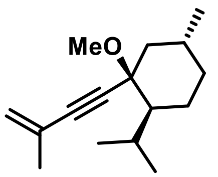

S24

^{1}H NMR (400 MHz, $CDCl_3$)

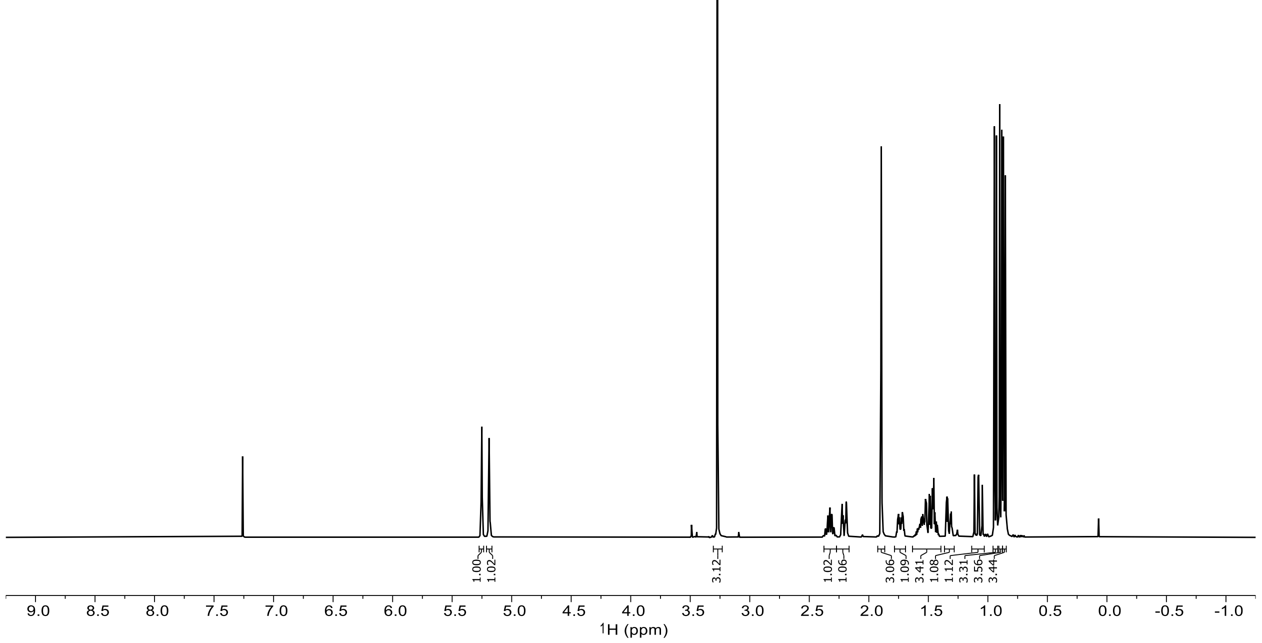

^{13}C NMR (101 MHz, $CDCl_3$)

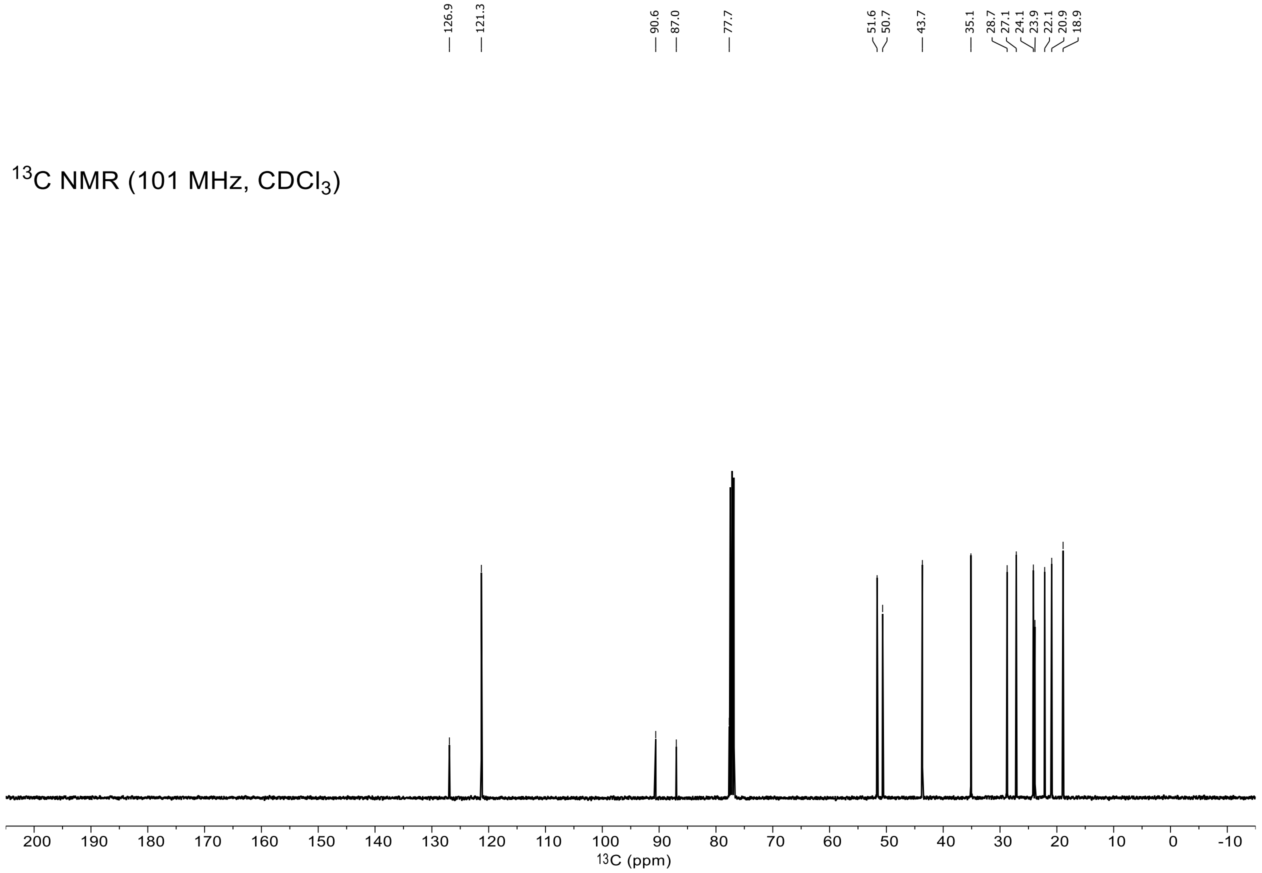

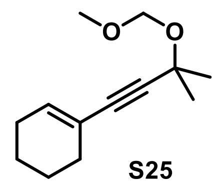

S25

^1H NMR (400 MHz, $CDCl_3$)

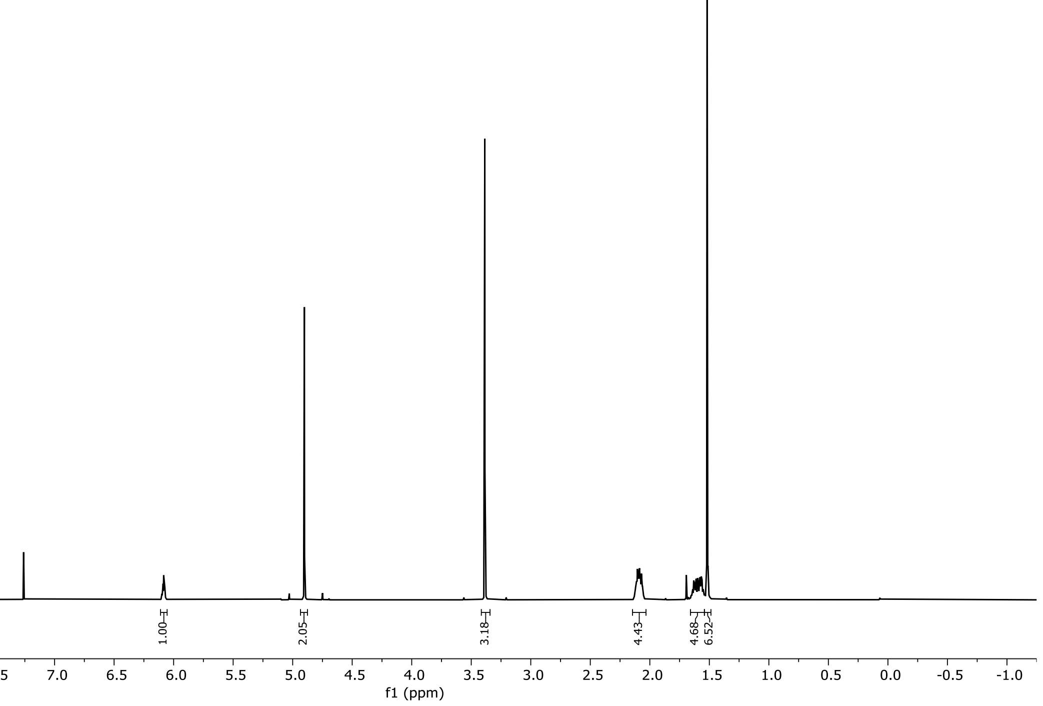

^{13}C NMR (101 MHz, $CDCl_3$)

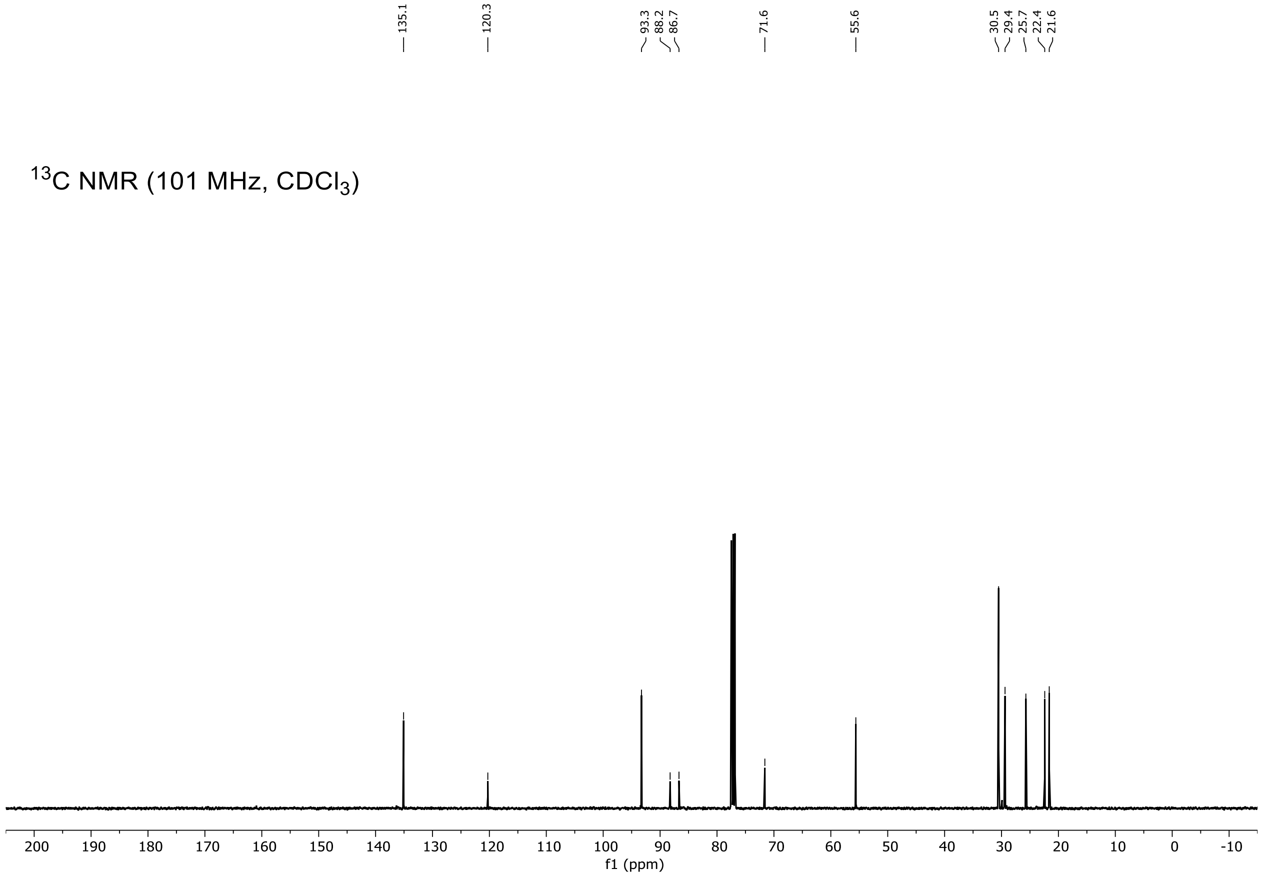

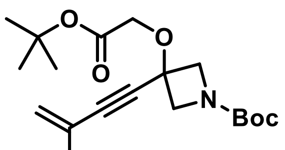

S26

^{1}H NMR (400 MHz, $CDCl_3$)

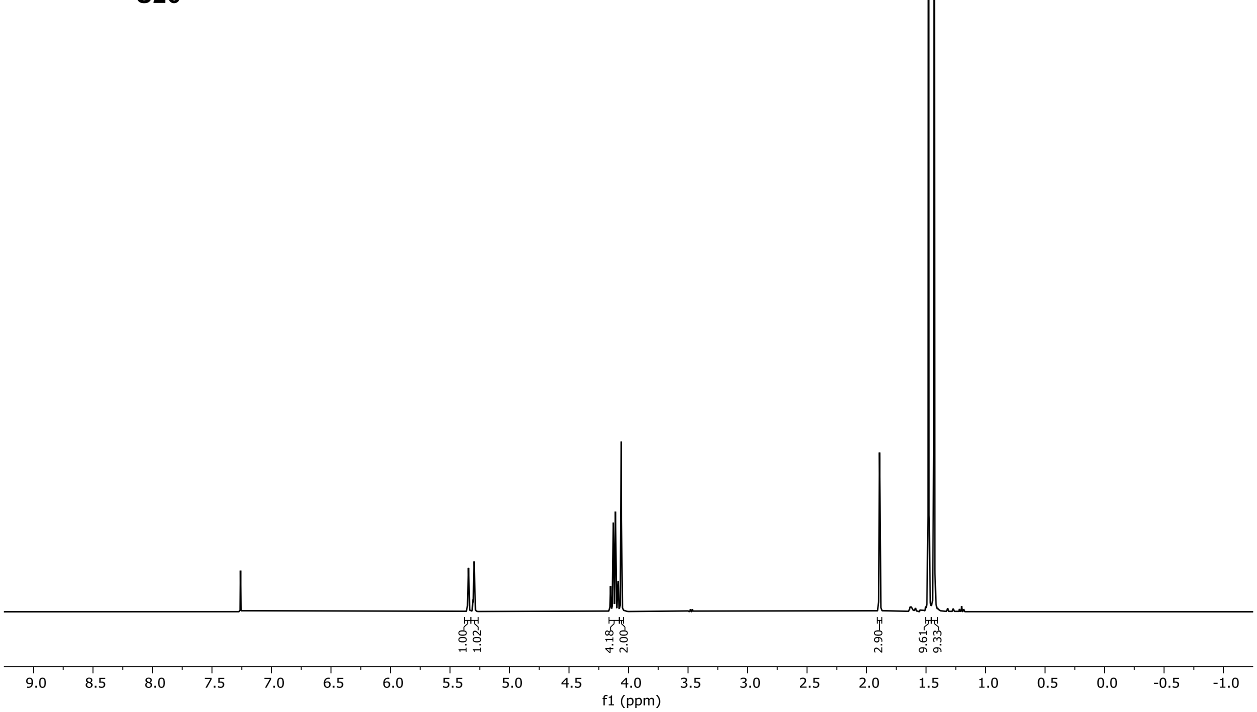

^{13}C NMR (101 MHz, $CDCl_3$)

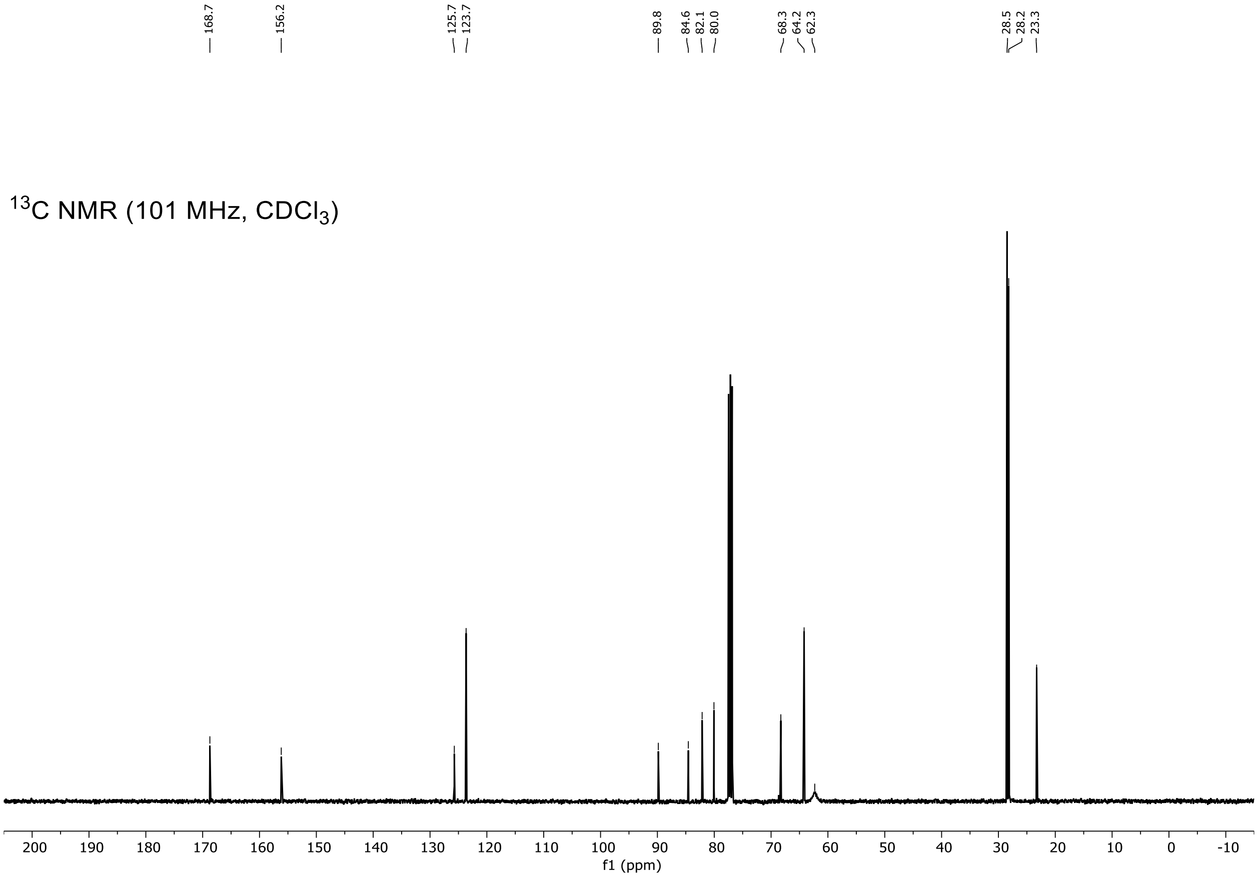

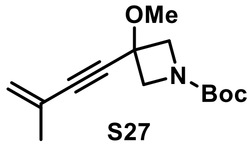

S27

^1H NMR (400 MHz, $CDCl_3$)

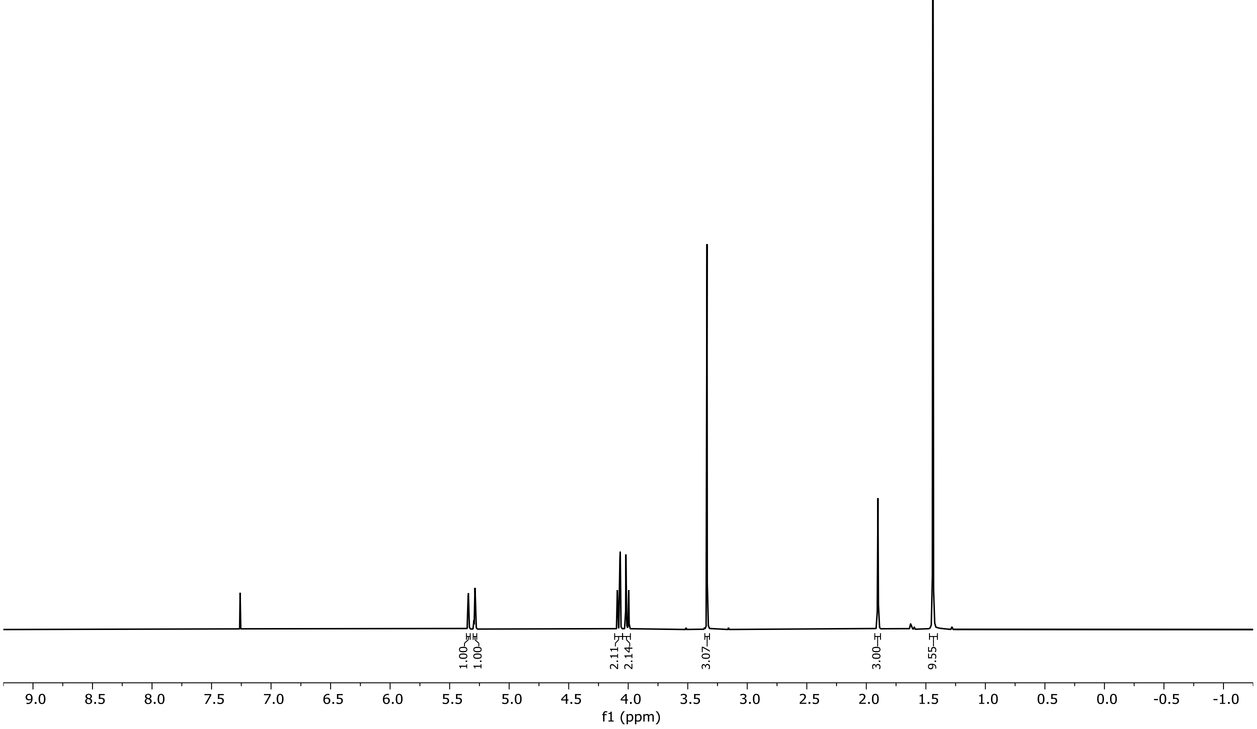

^{13}C NMR (101 MHz, $CDCl_3$)

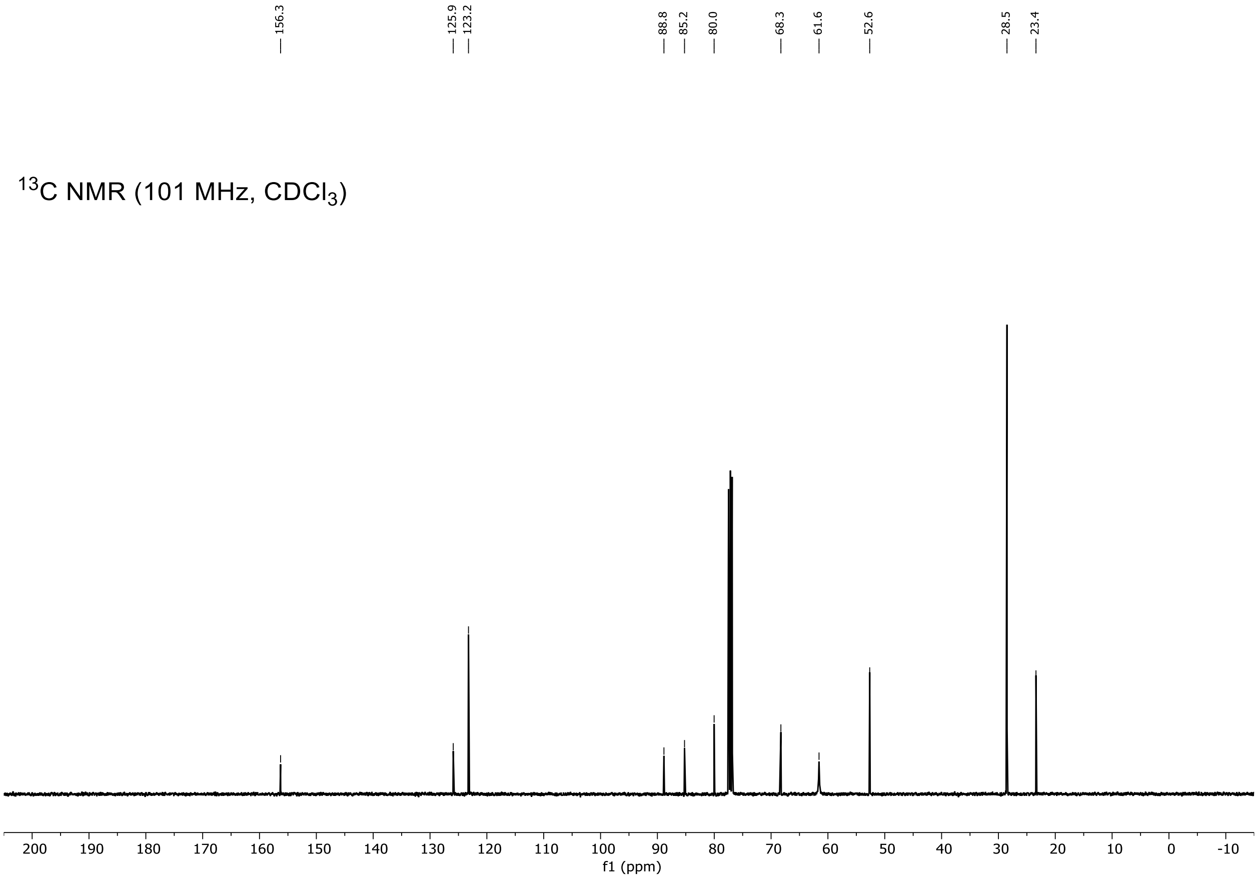

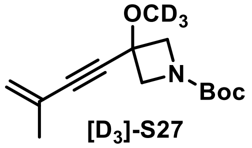

[D$_3$]-S27

^{1}H NMR (400 MHz, $CDCl_3$)

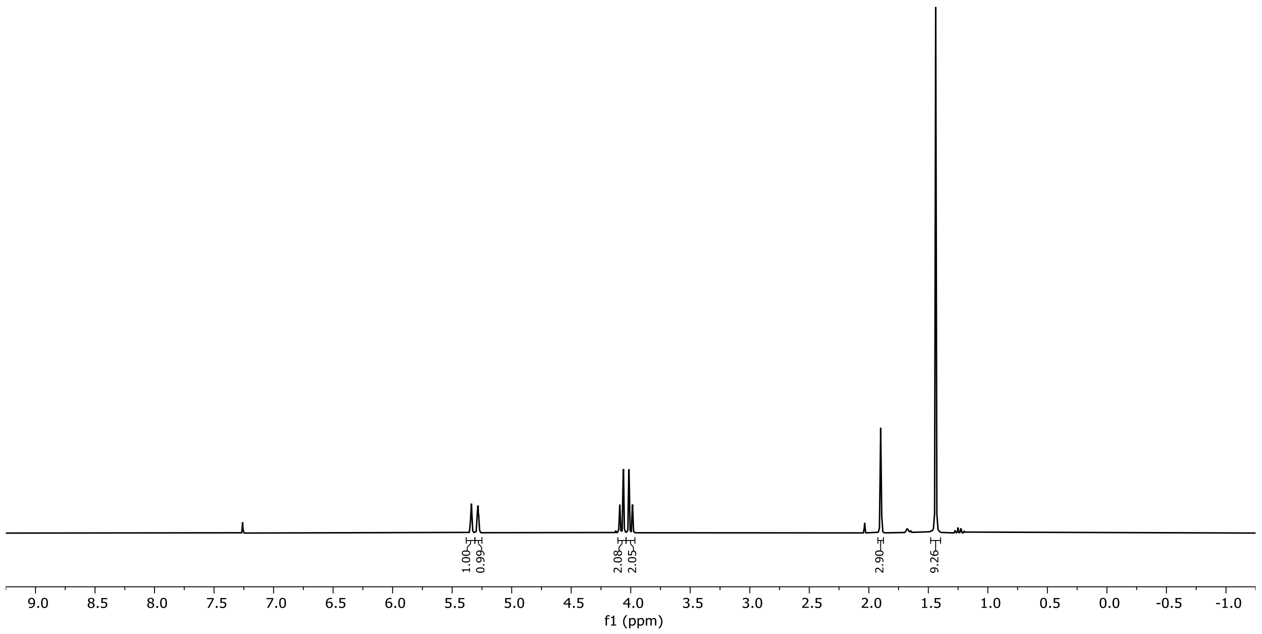

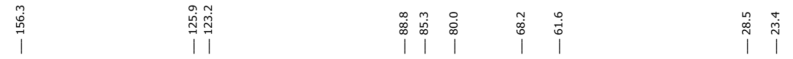

^{13}C NMR (101 MHz, $CDCl_3$)

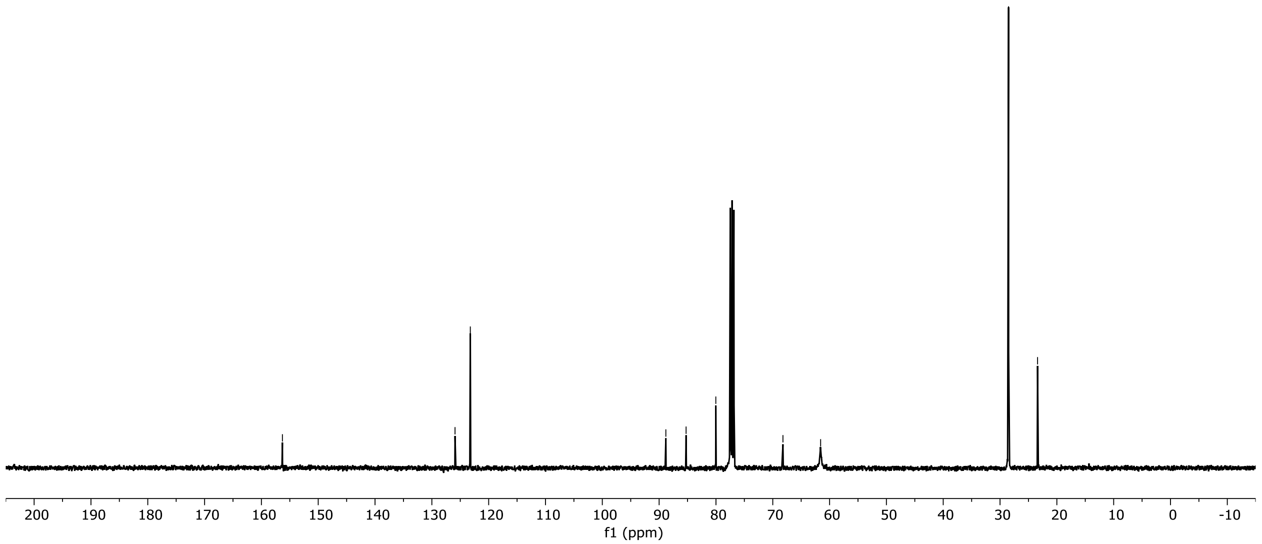

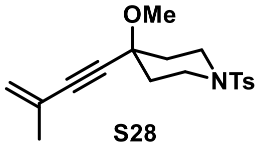

S28

^{1}H NMR (400 MHz, $CDCl_3$)

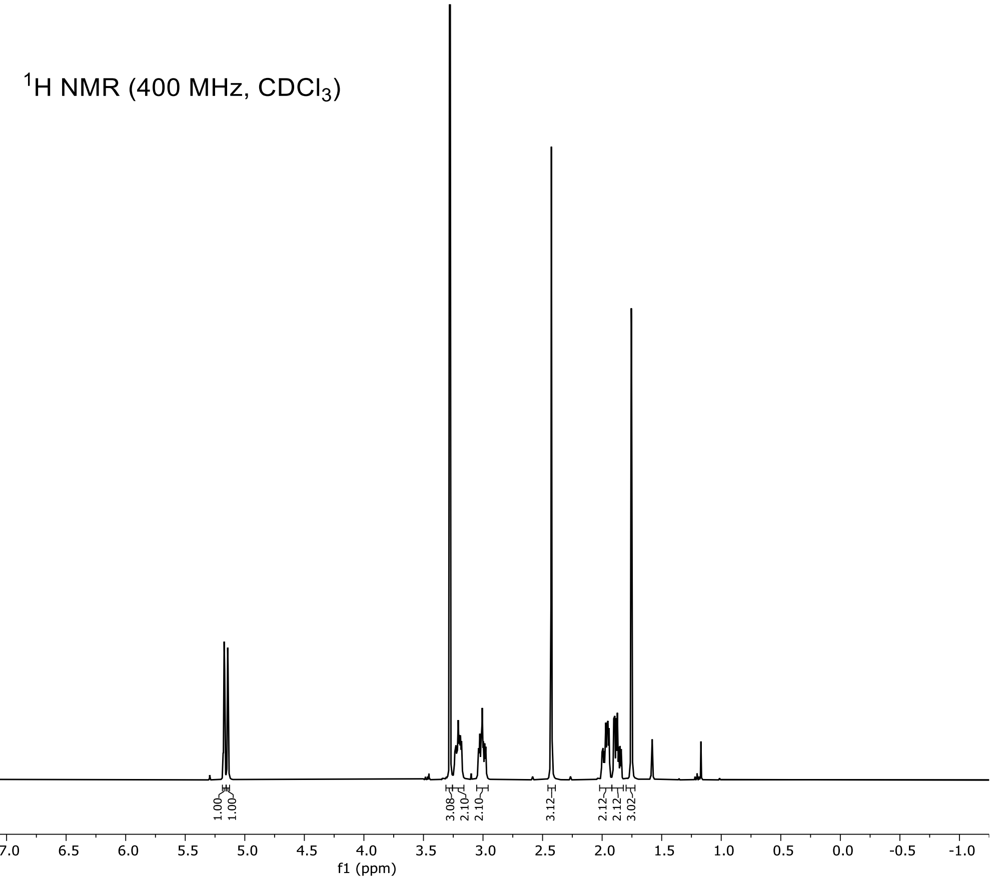

^{13}C NMR (101 MHz, $CDCl_3$)

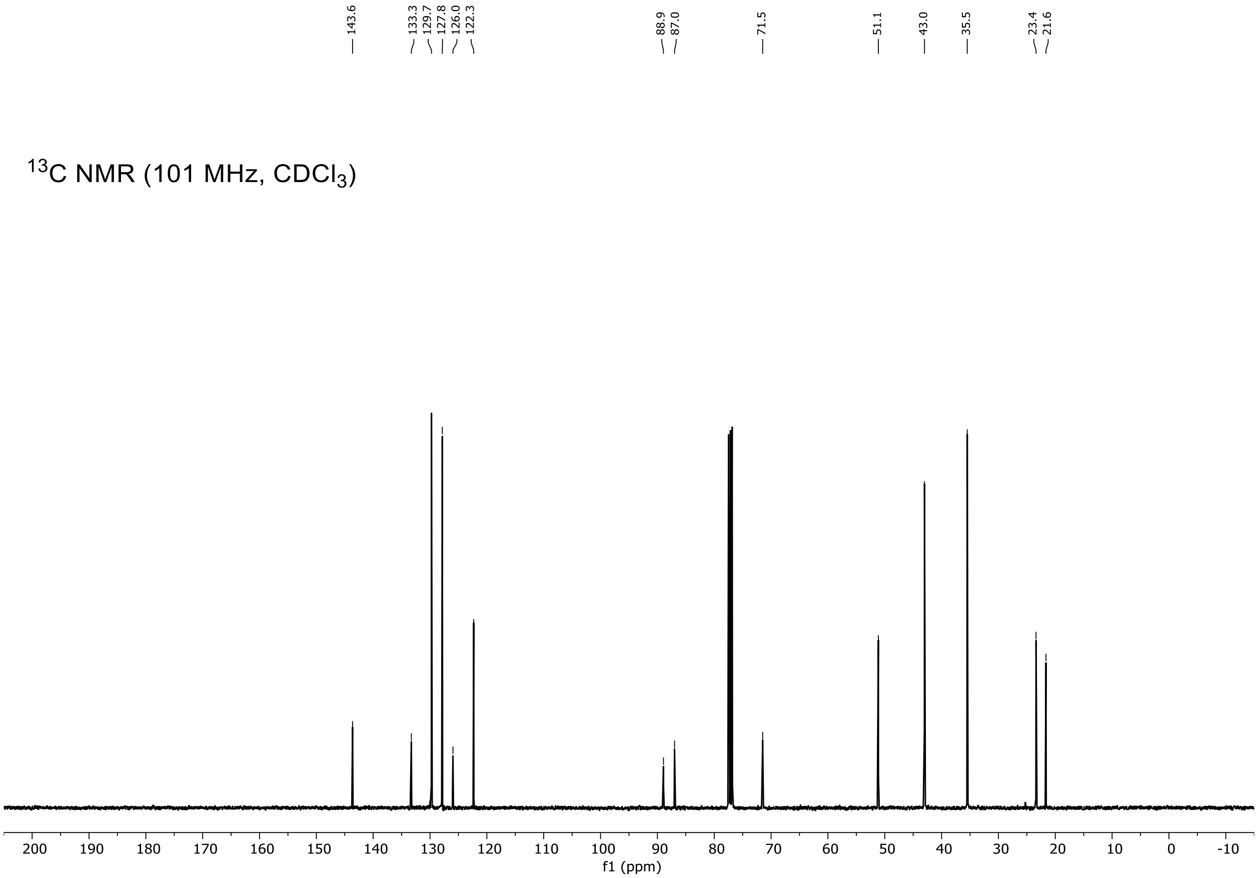

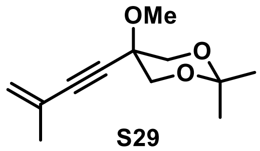

S29

^{1}H NMR (400 MHz, $CDCl_3$)

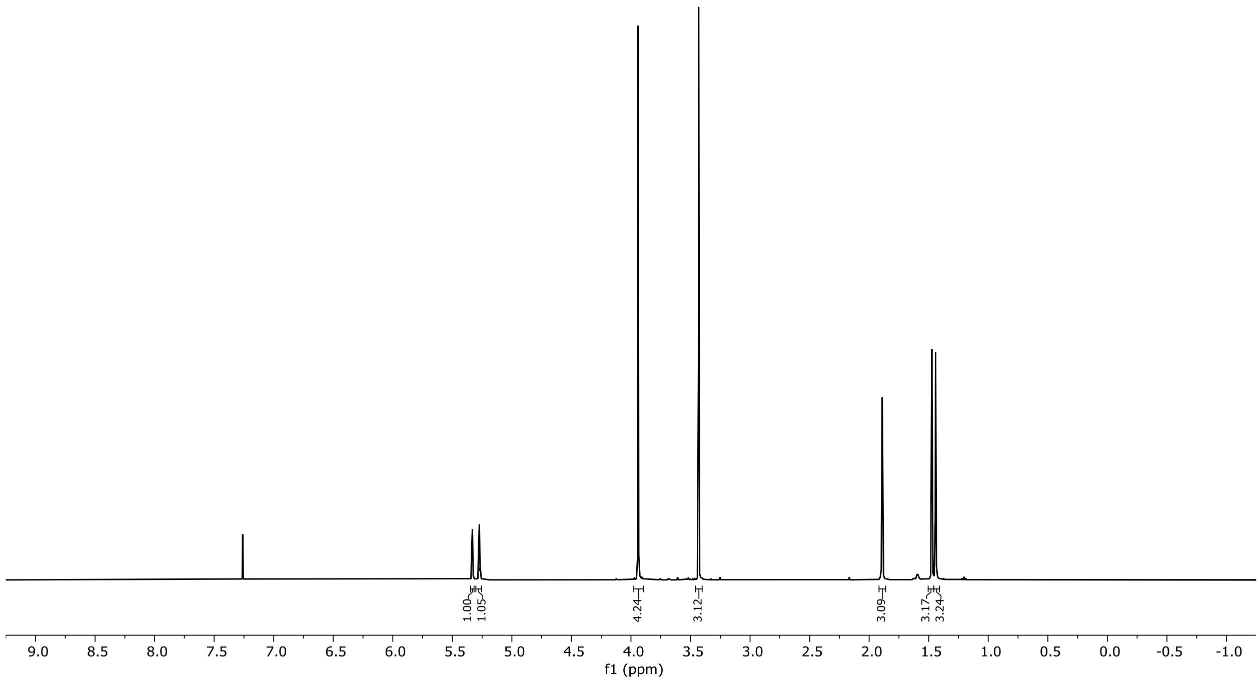

^{13}C NMR (101 MHz, $CDCl_3$)

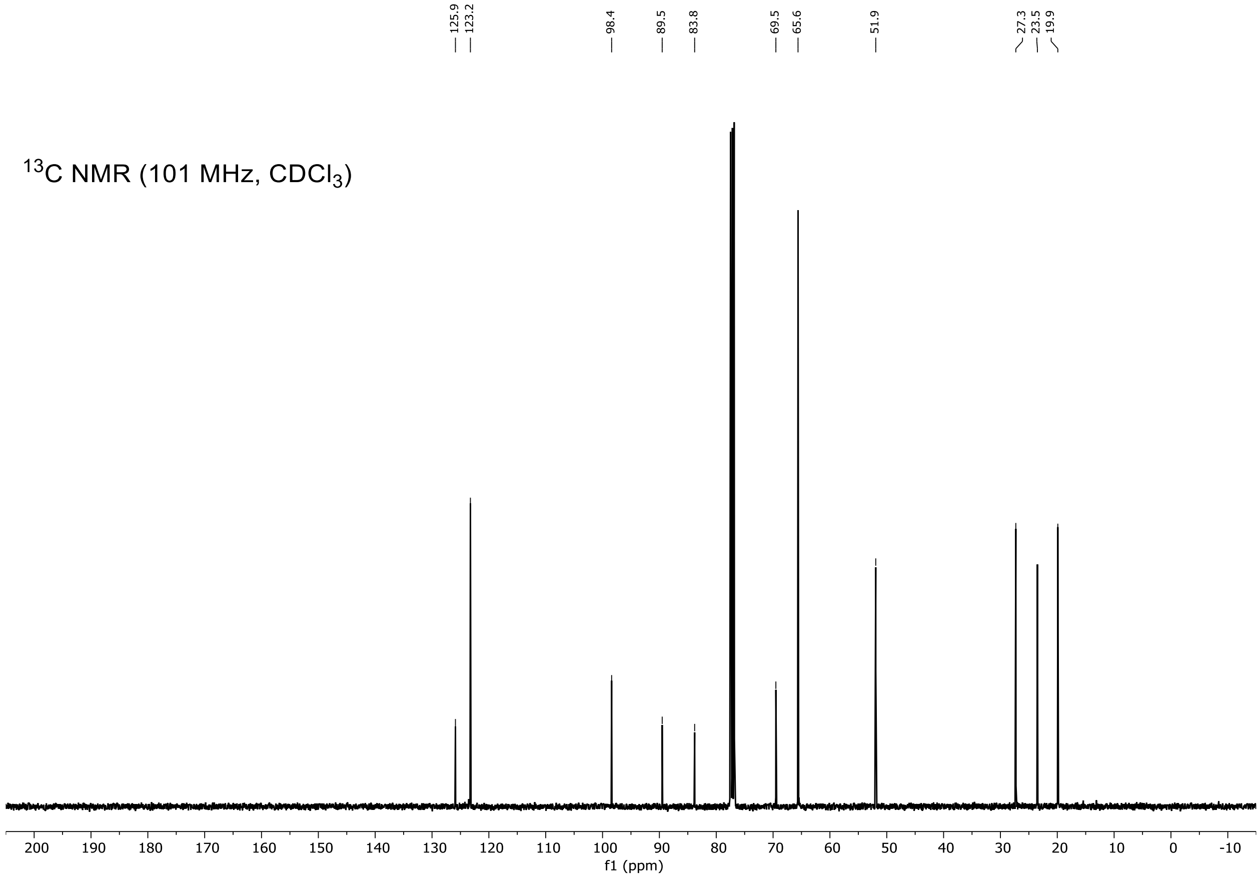

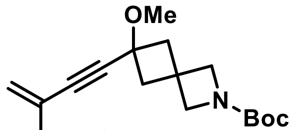

S30

^{1}H NMR (400 MHz, $CDCl_3$)

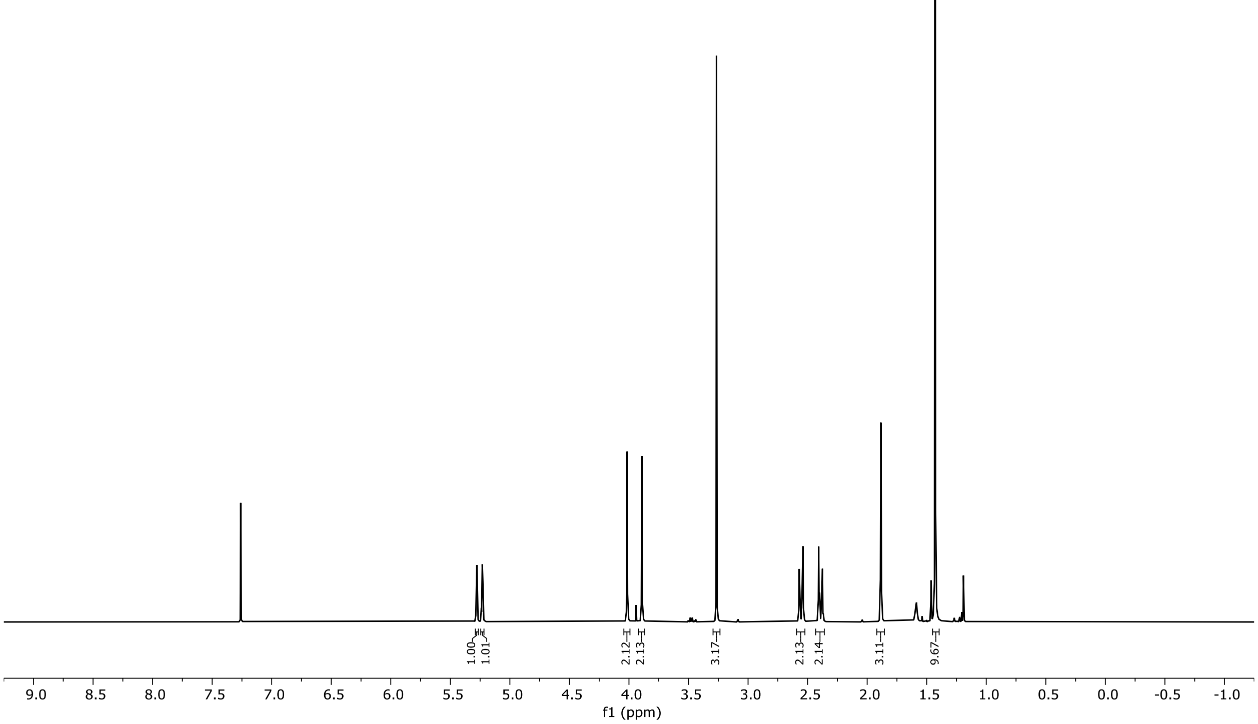

^{13}C NMR (101 MHz, $CDCl_3$)

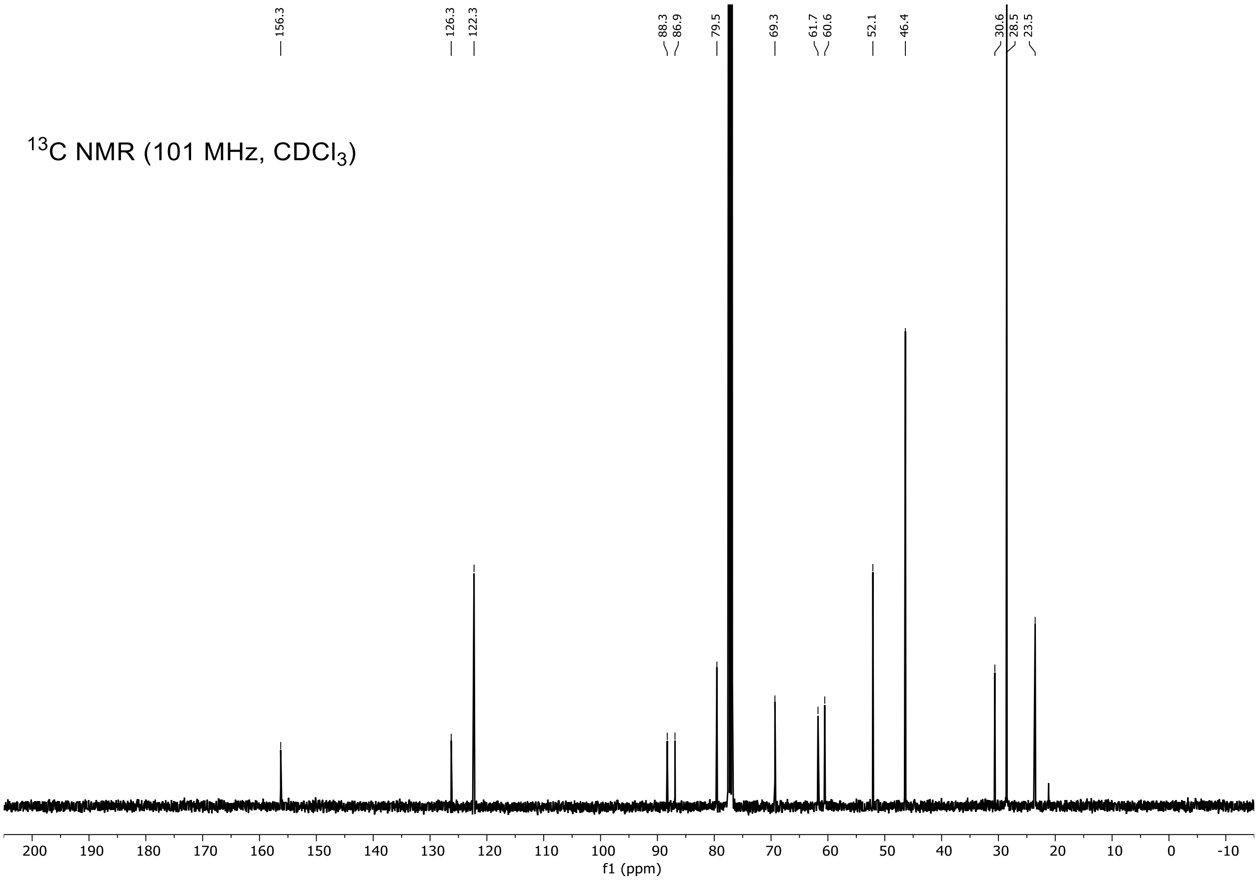

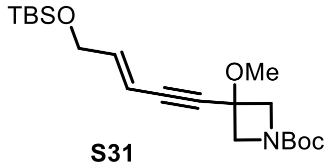

S31

^{1}H NMR (400 MHz, $CDCl_3$)

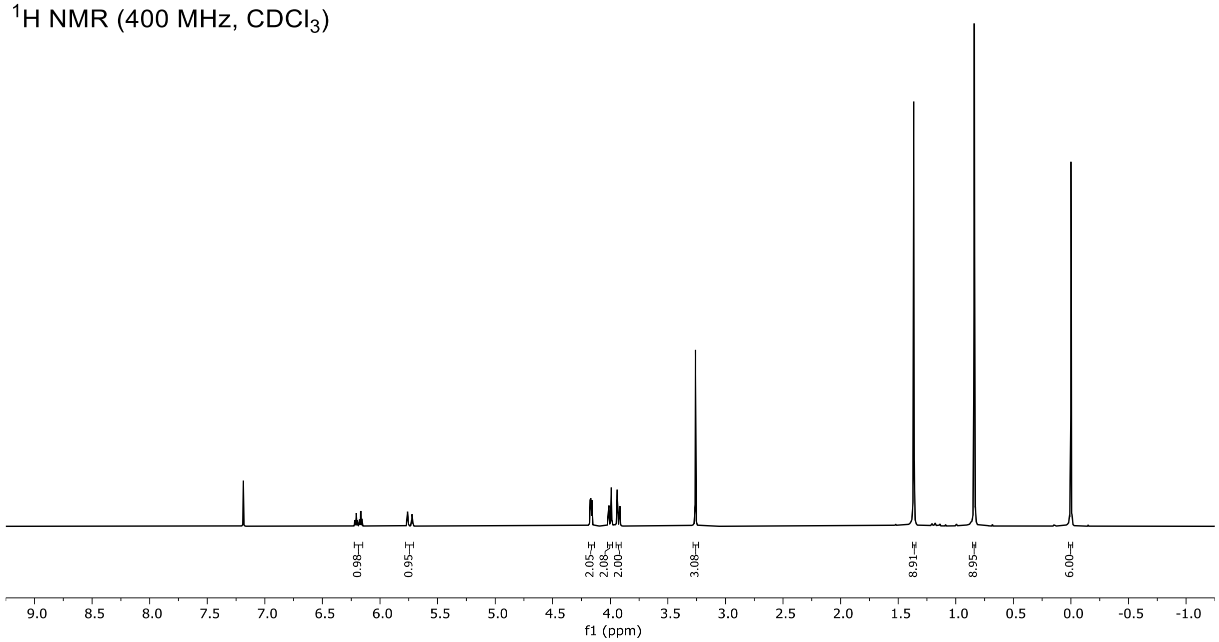

^{13}C NMR (101 MHz, $CDCl_3$)

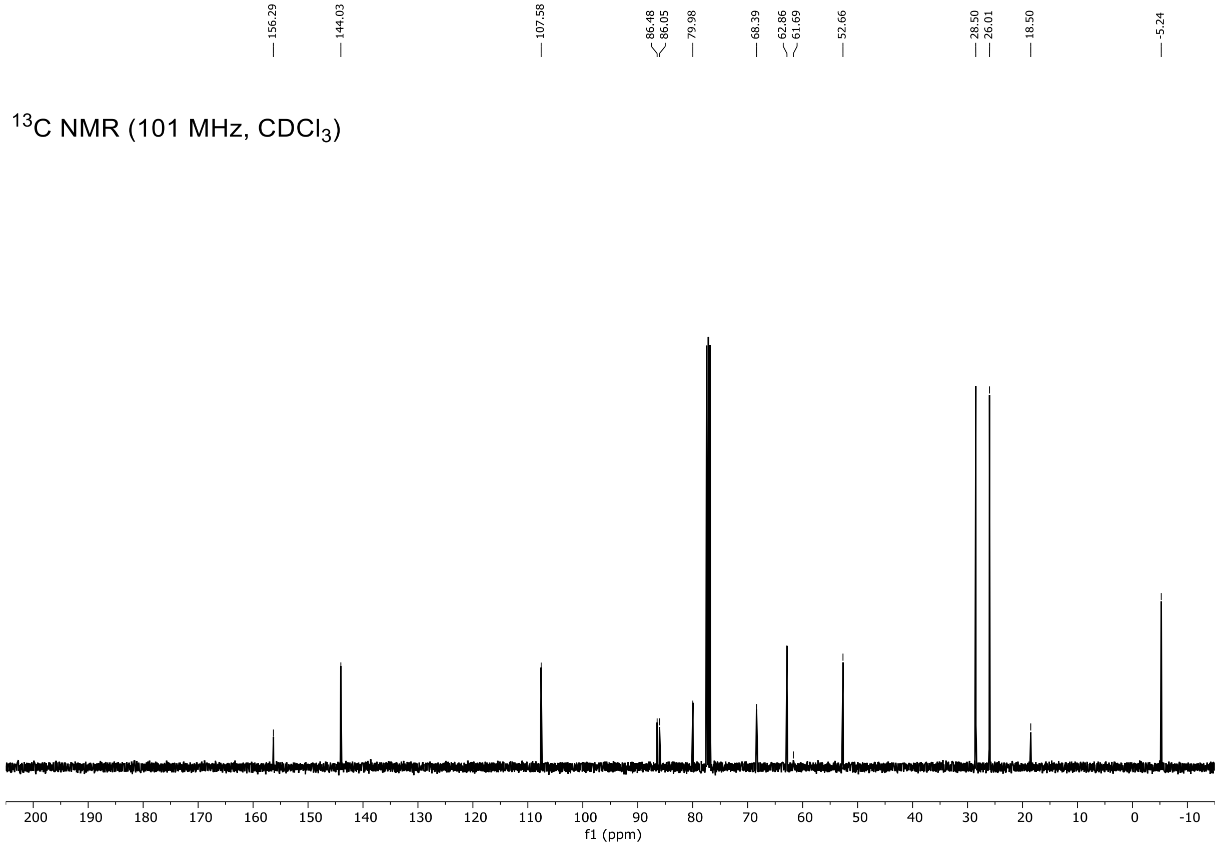

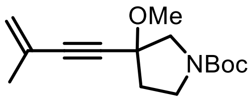

S32

^{1}H NMR (400 MHz, $CDCl_3$)

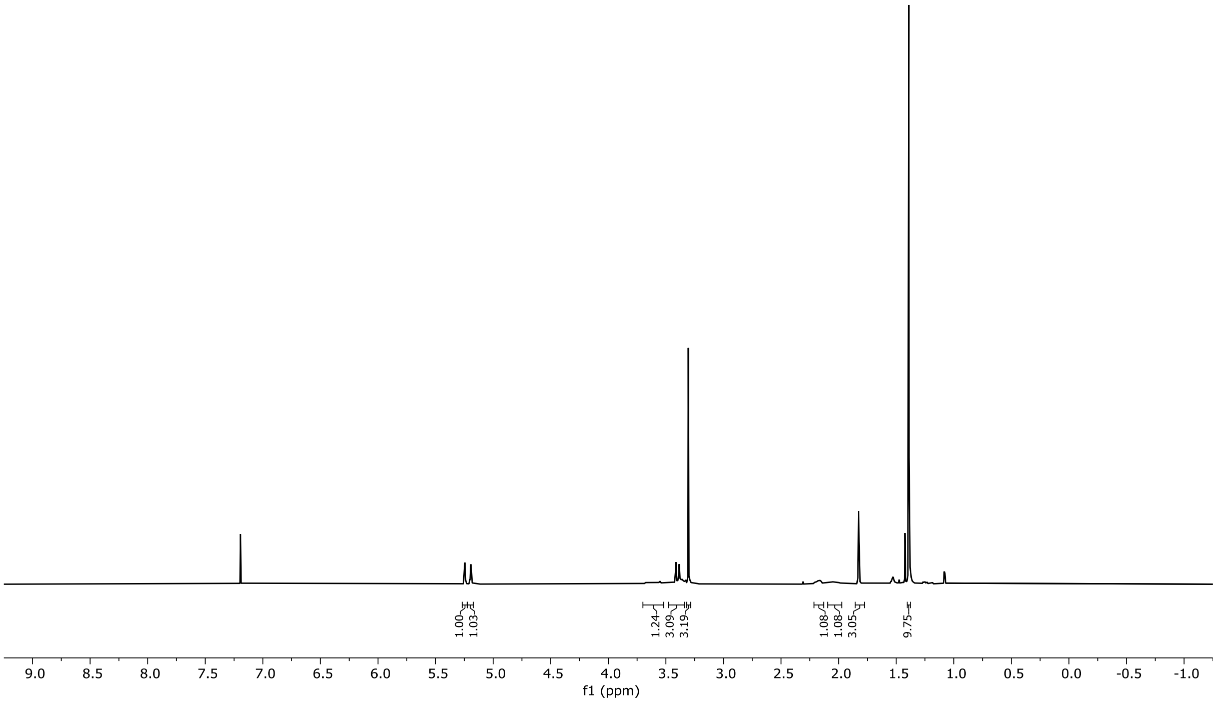

^{13}C NMR (101 MHz, $CDCl_3$)

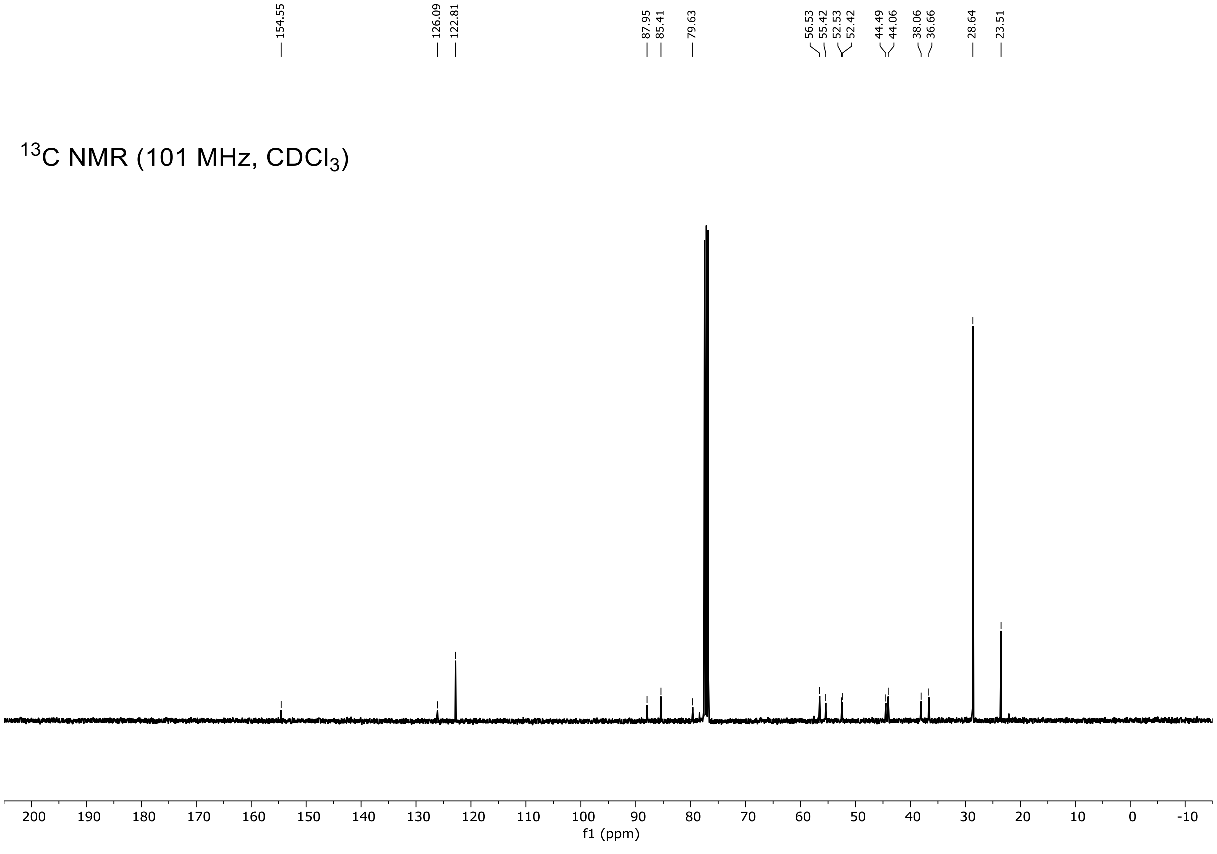

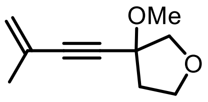

S33

^{1}H NMR (400 MHz, $CDCl_3$)

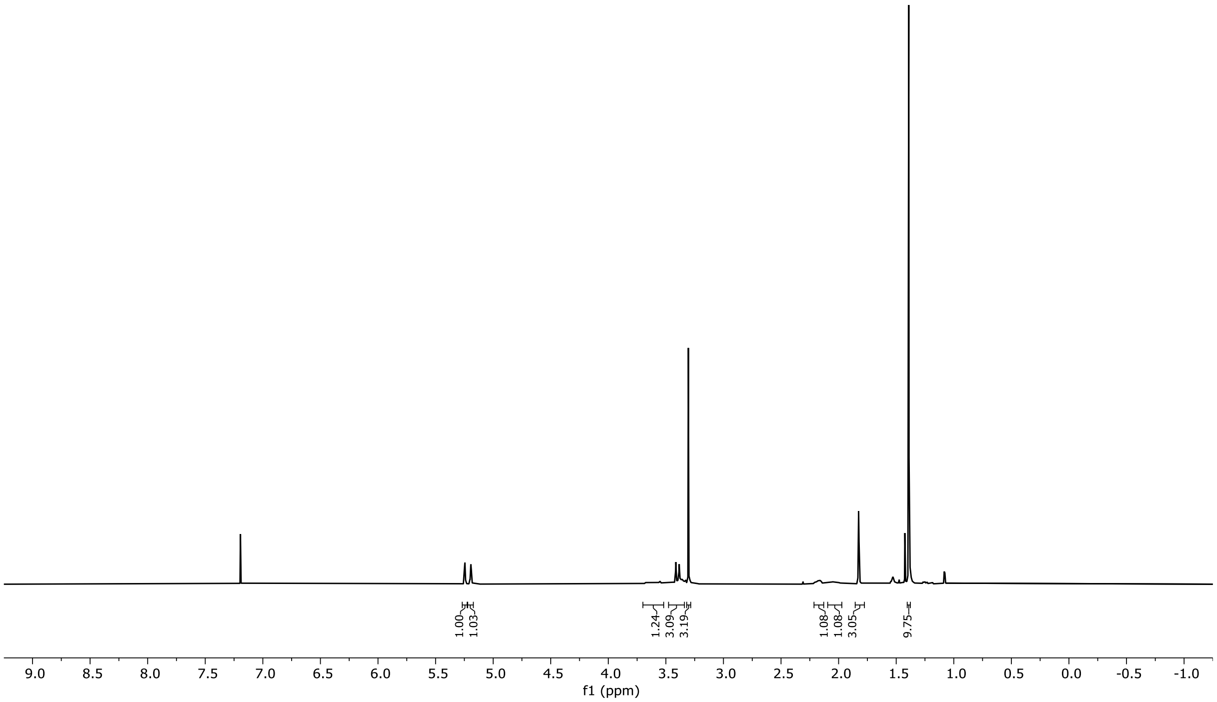

^{13}C NMR (101 MHz, $CDCl_3$)

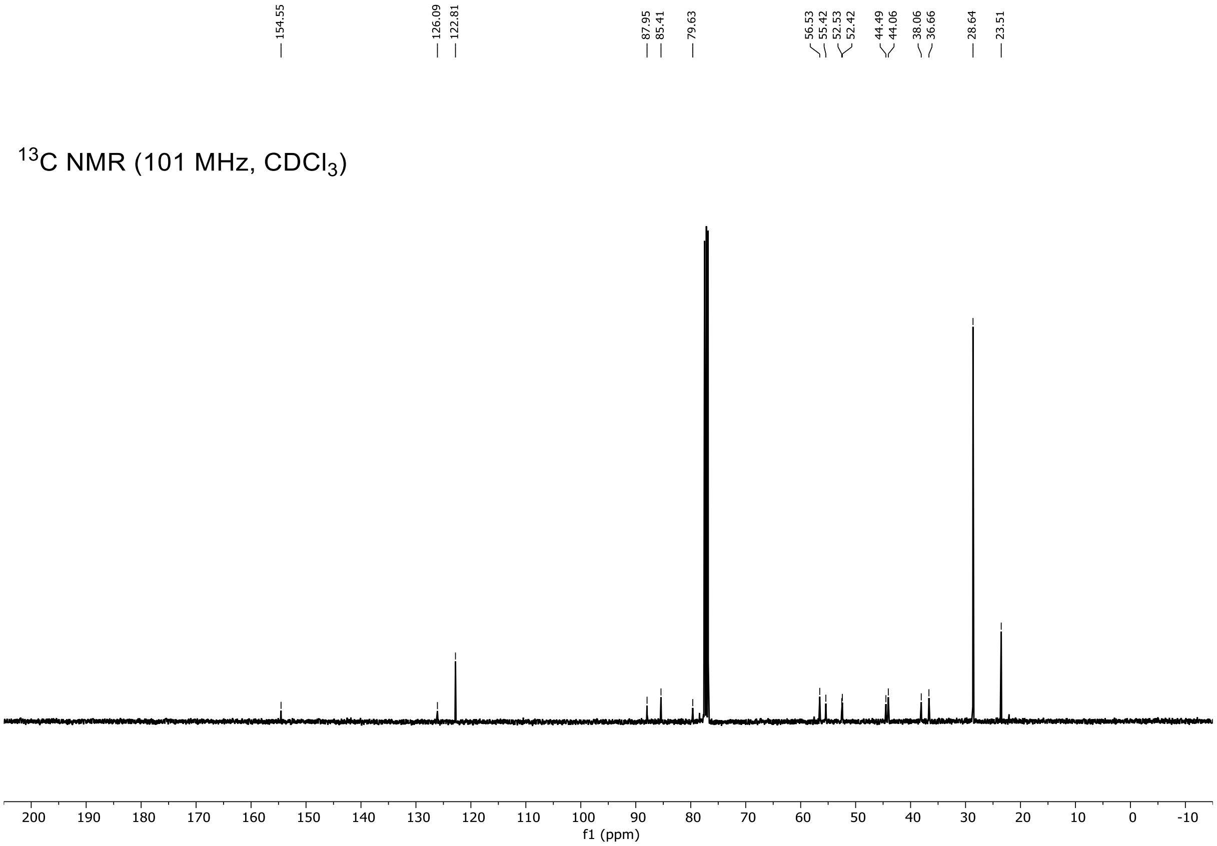

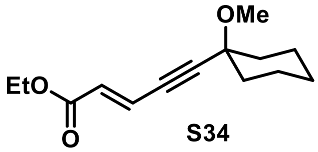

S34

^{1}H NMR (400 MHz, $CDCl_3$)

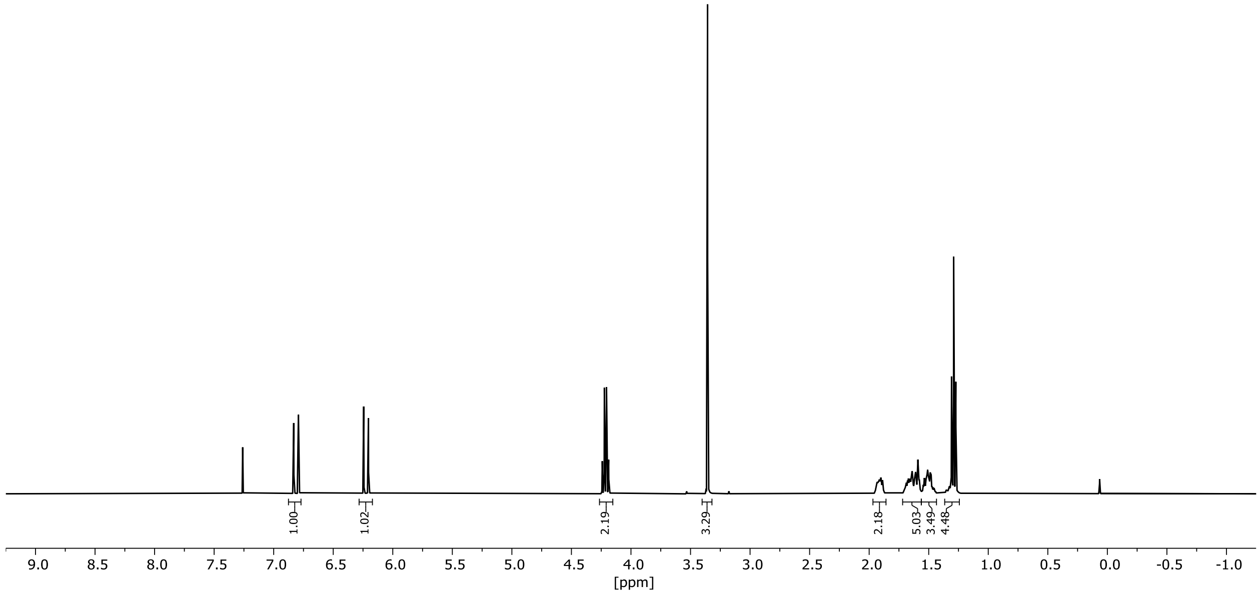

^{13}C NMR (101 MHz, $CDCl_3$)

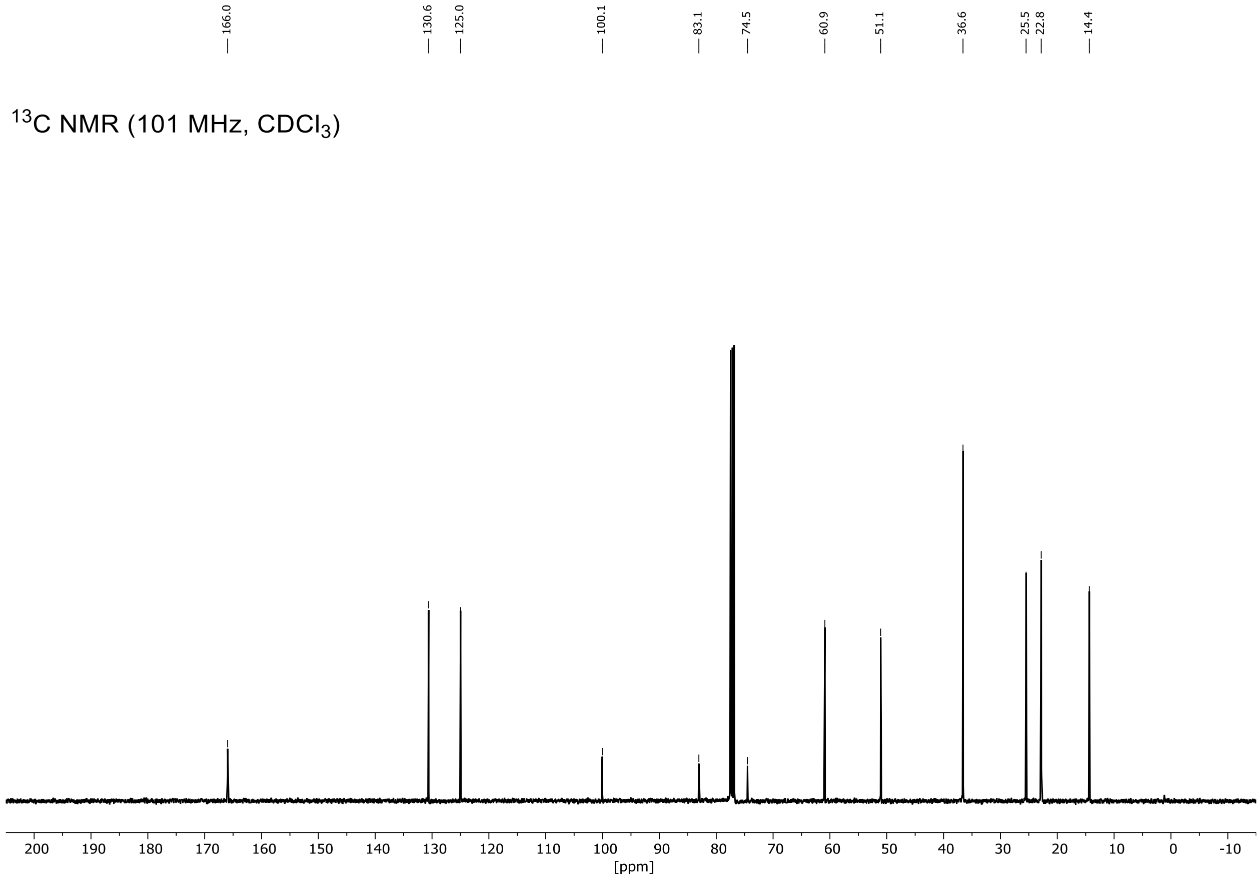

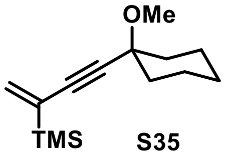

S35

^{1}H NMR (400 MHz, $CDCl_3$)

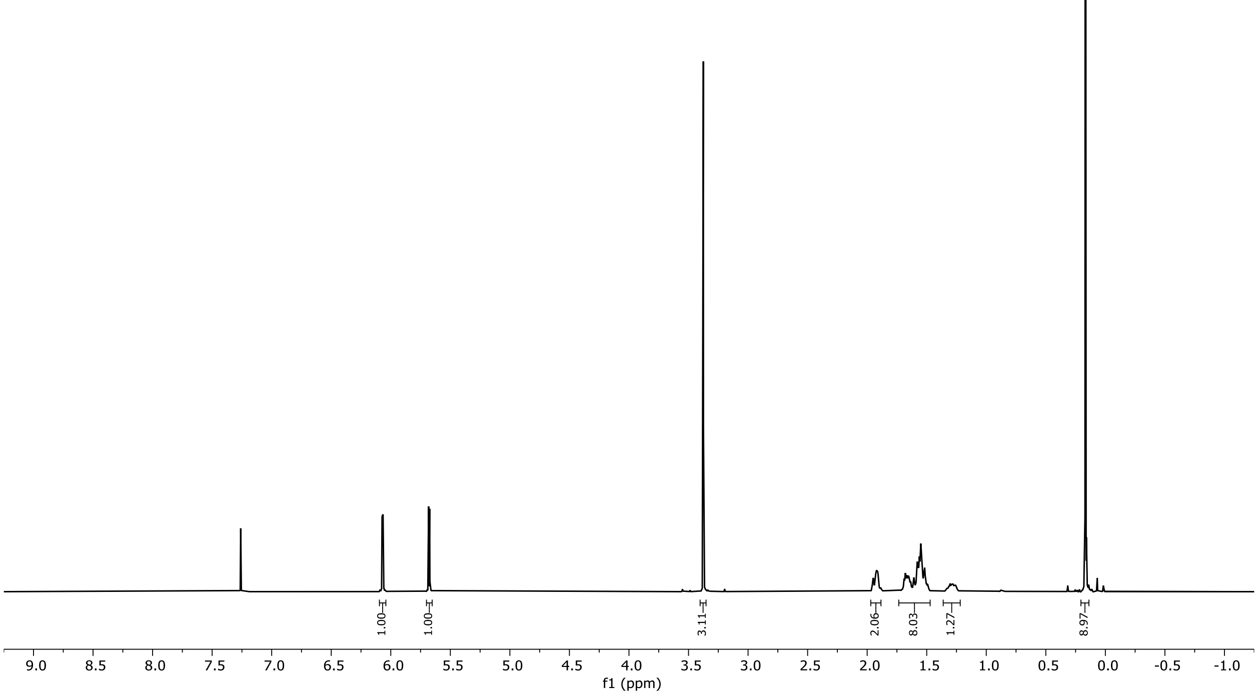

^{13}C NMR (101 MHz, $CDCl_3$)

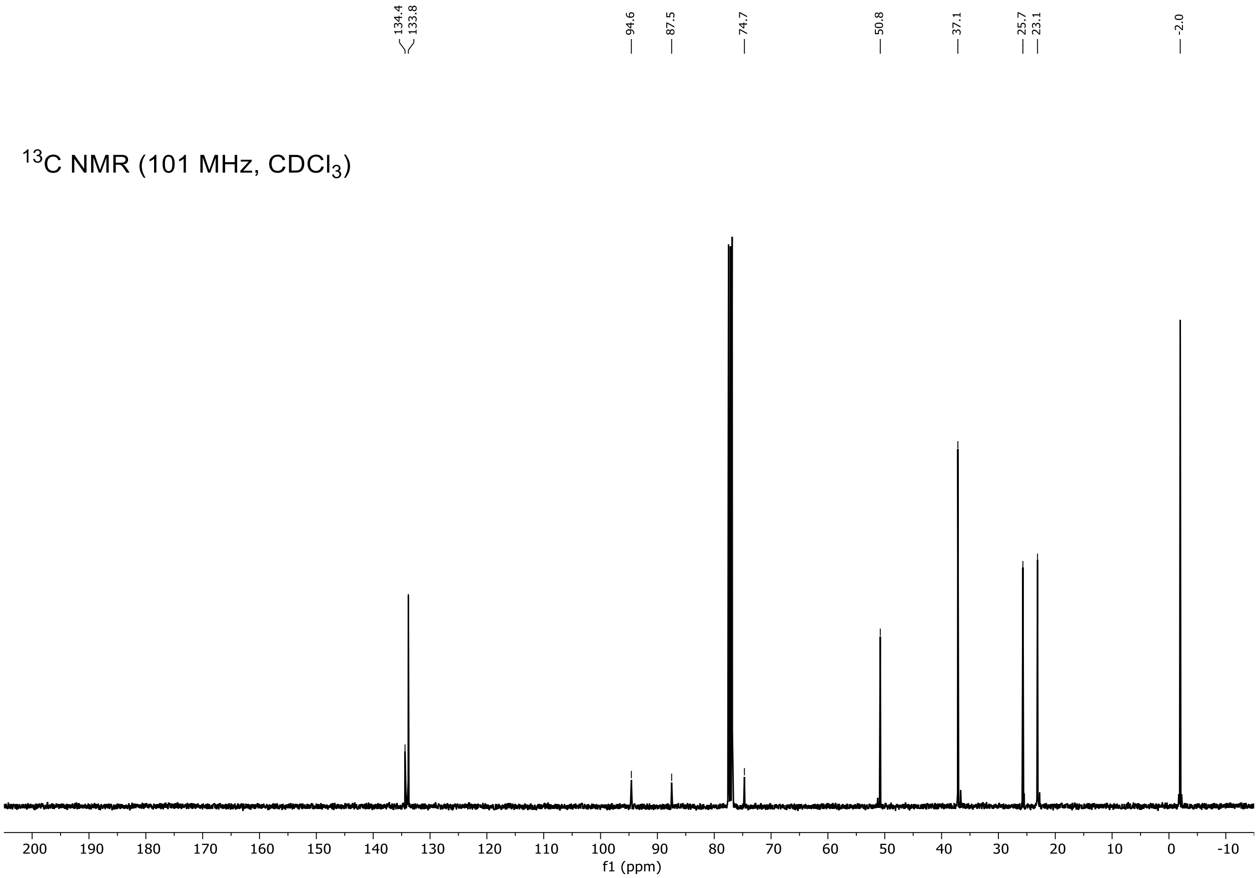

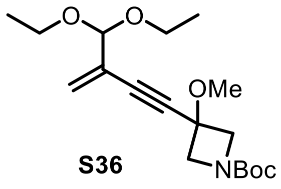

S36

^{1}H NMR (400 MHz, C_6D_6)

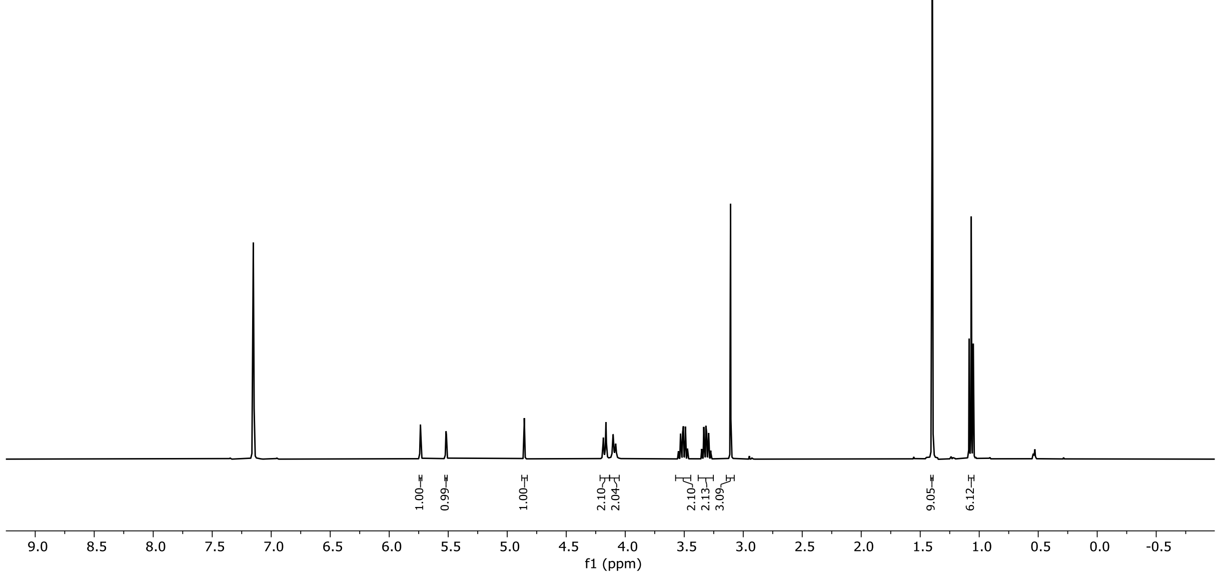

^{13}C NMR (101 MHz, C_6D_6)

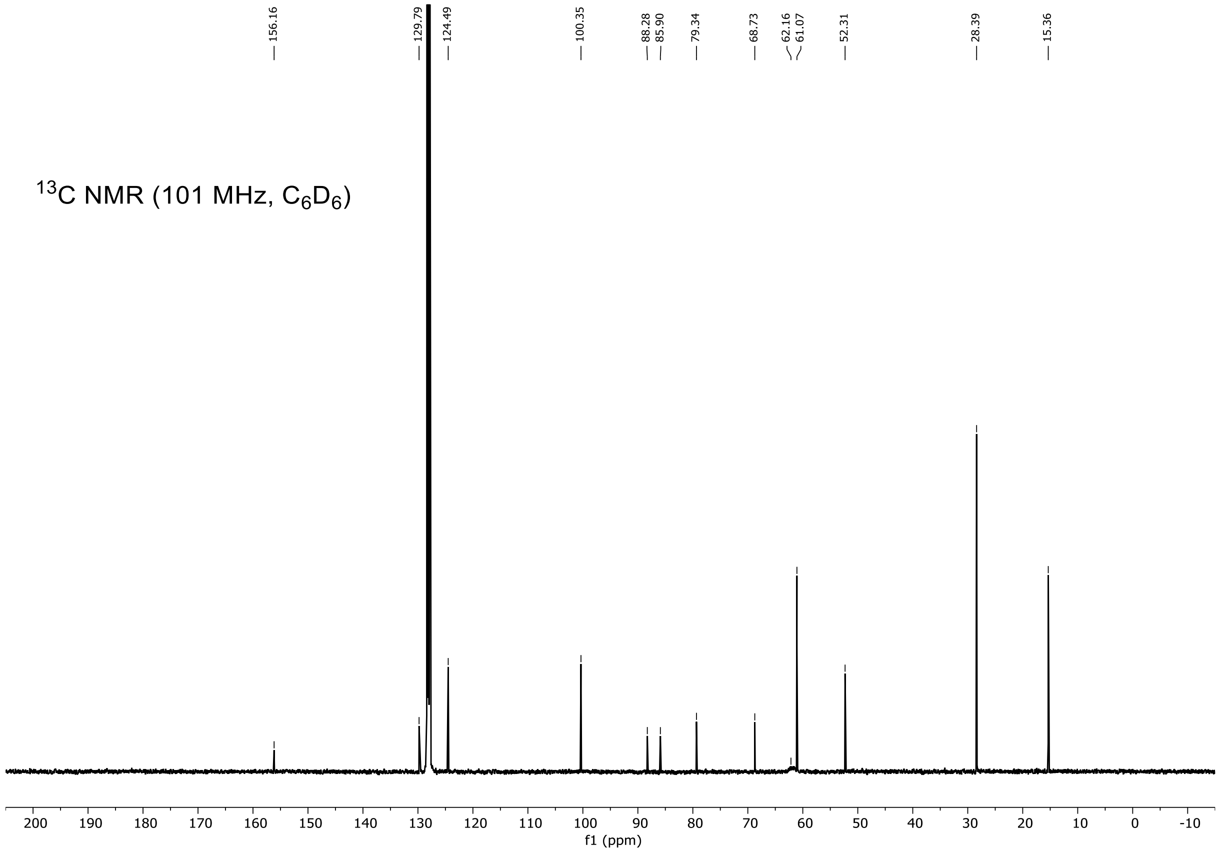

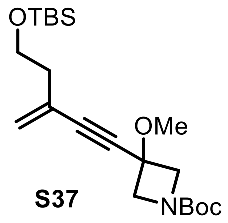

S37

^1H NMR (400 MHz, $CDCl_3$)

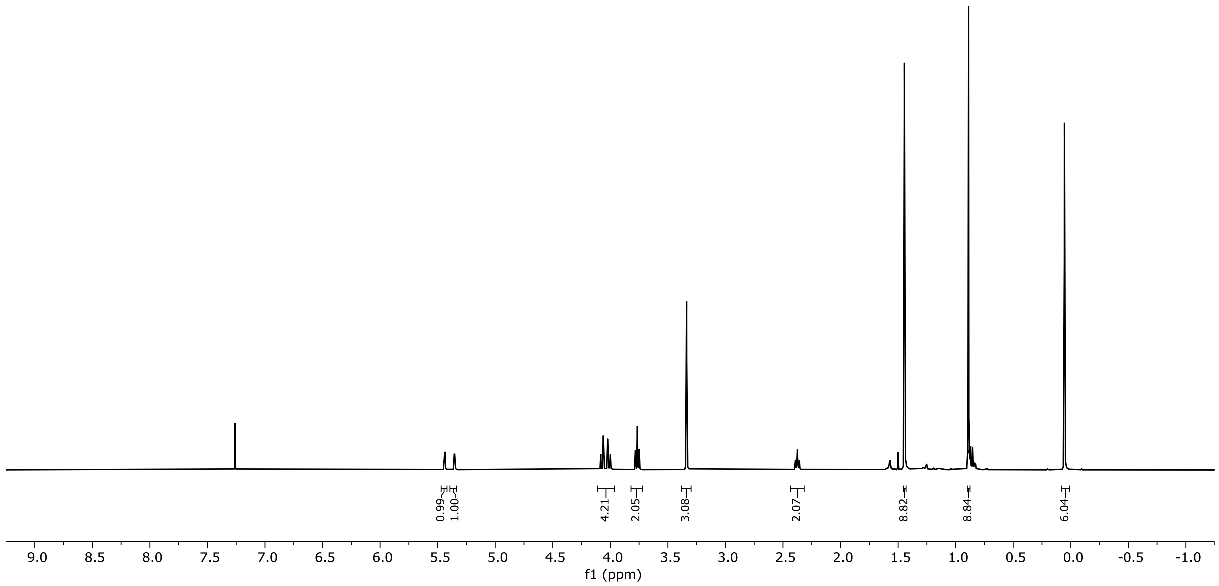

^{13}C NMR (101 MHz, $CDCl_3$)

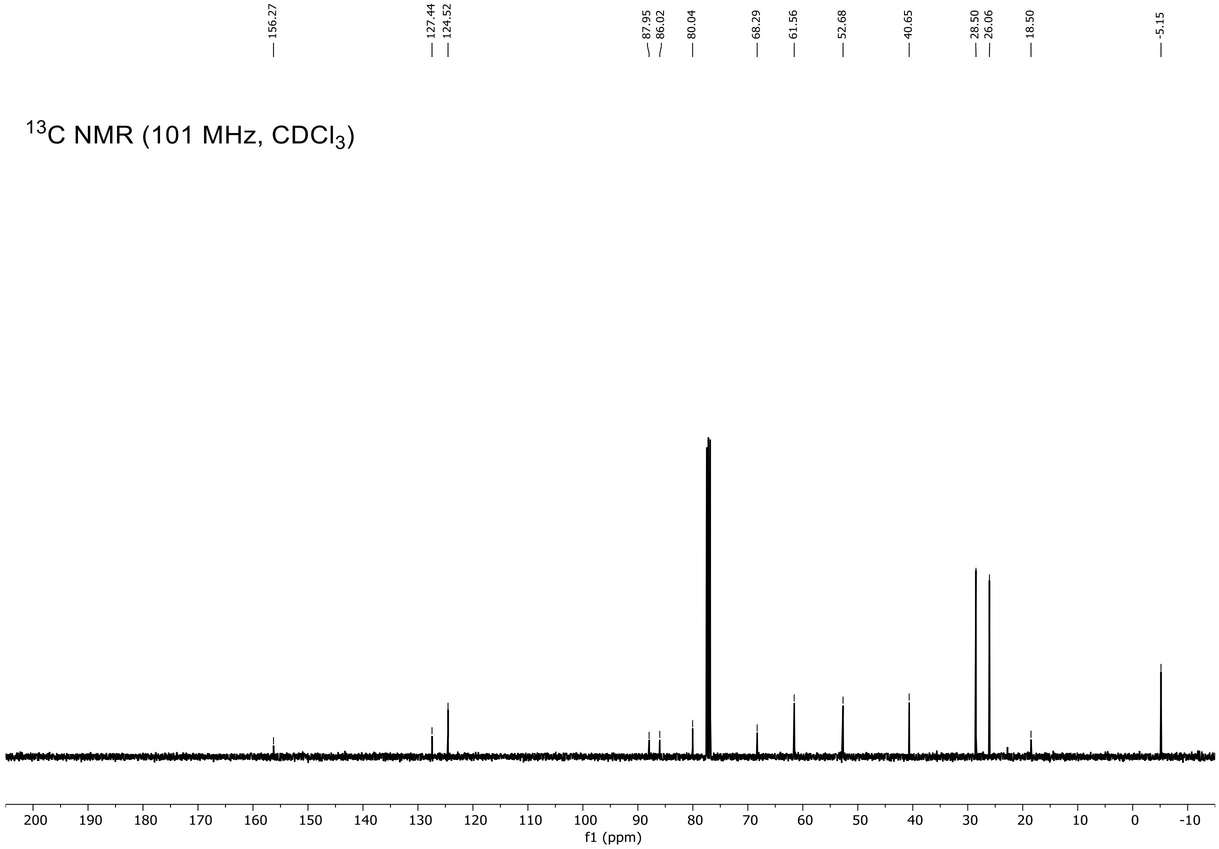

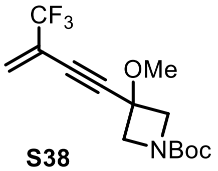

S38

^{1}H NMR (400 MHz, $CDCl_3$)

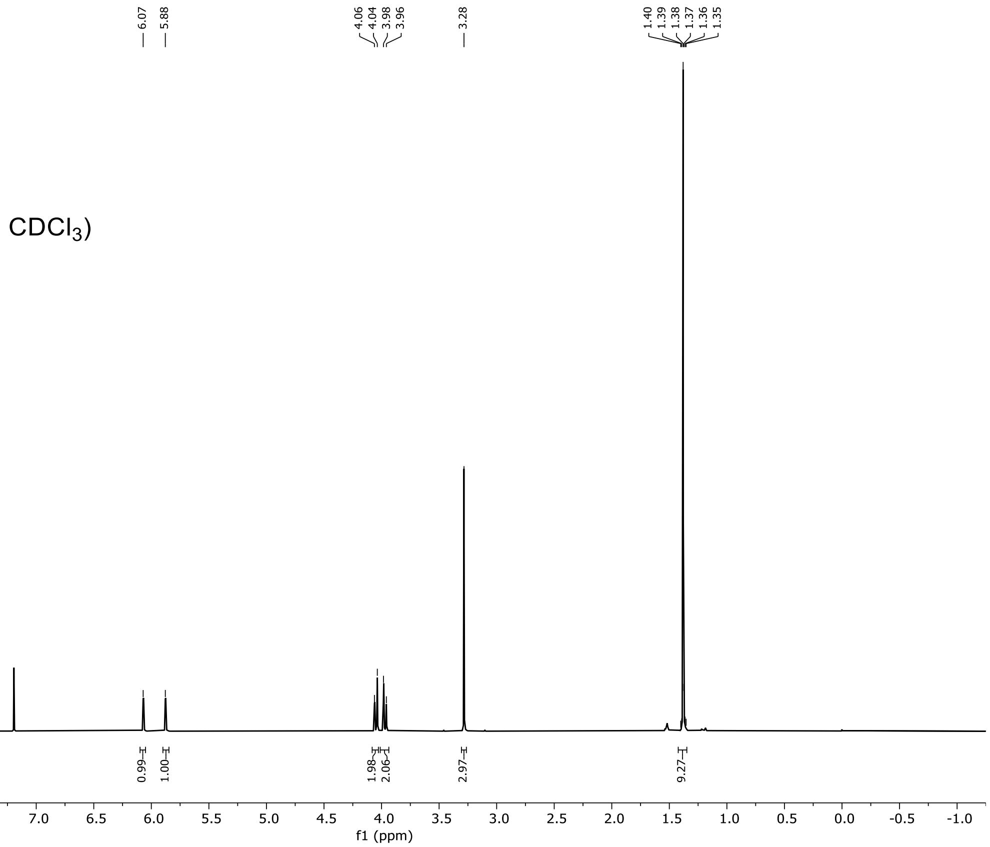

^{13}C NMR (101 MHz, $CDCl_3$)

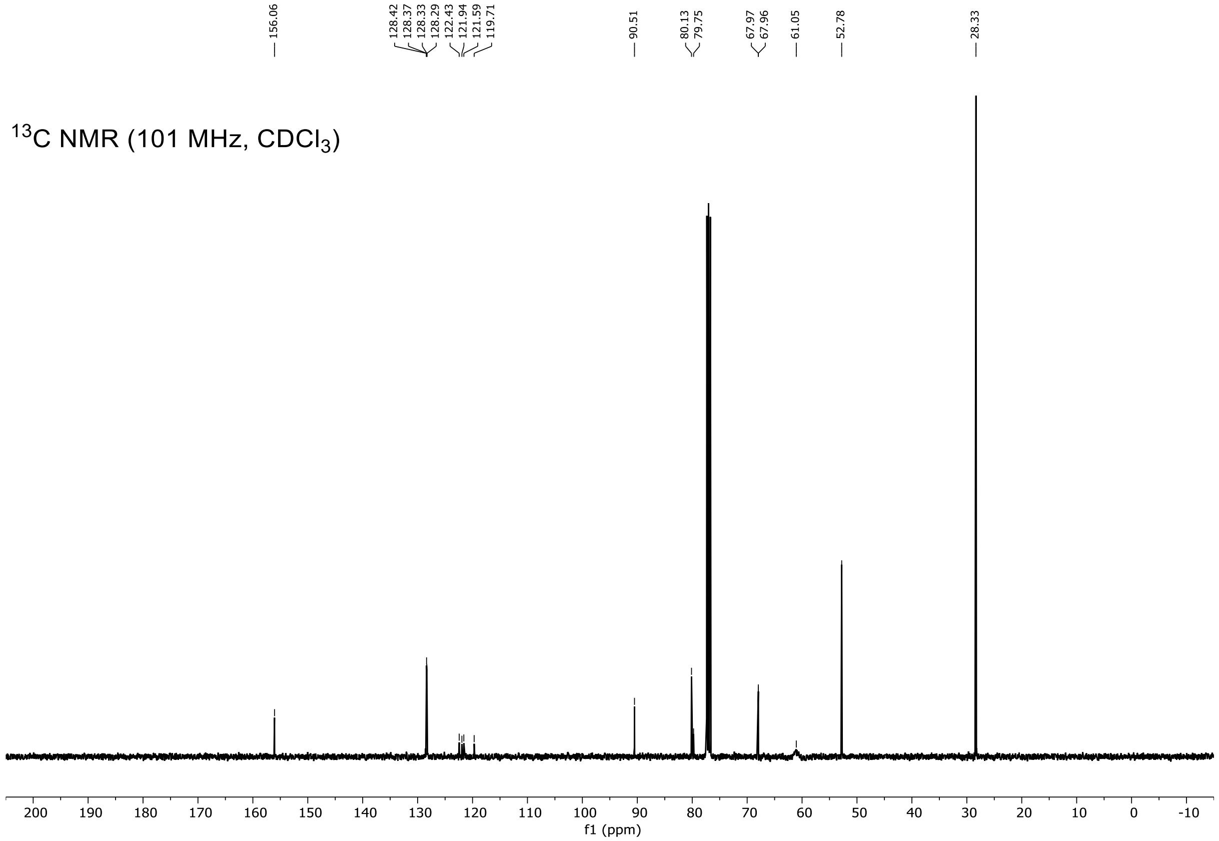

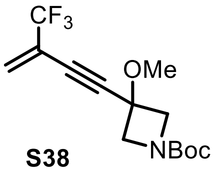

S38

^{19}F NMR (282 MHz, $CDCl_3$)

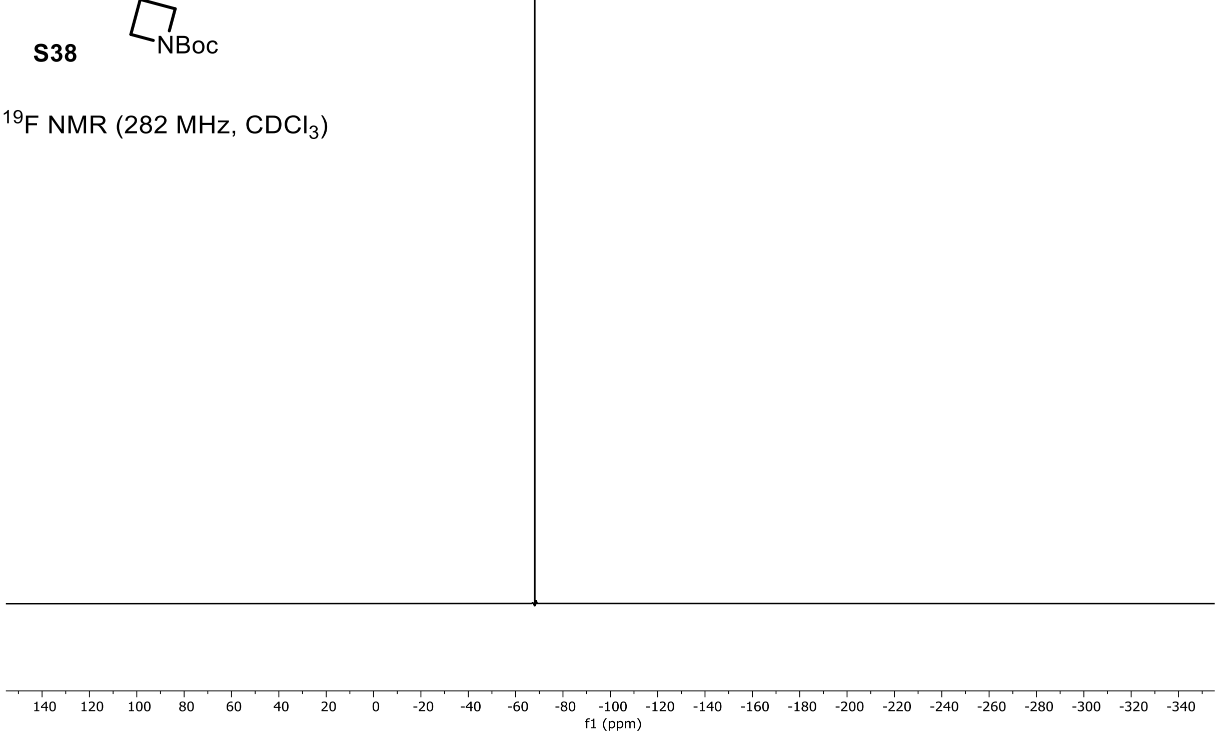

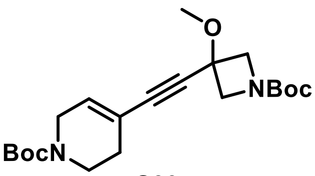
NBoc
BocN

S39

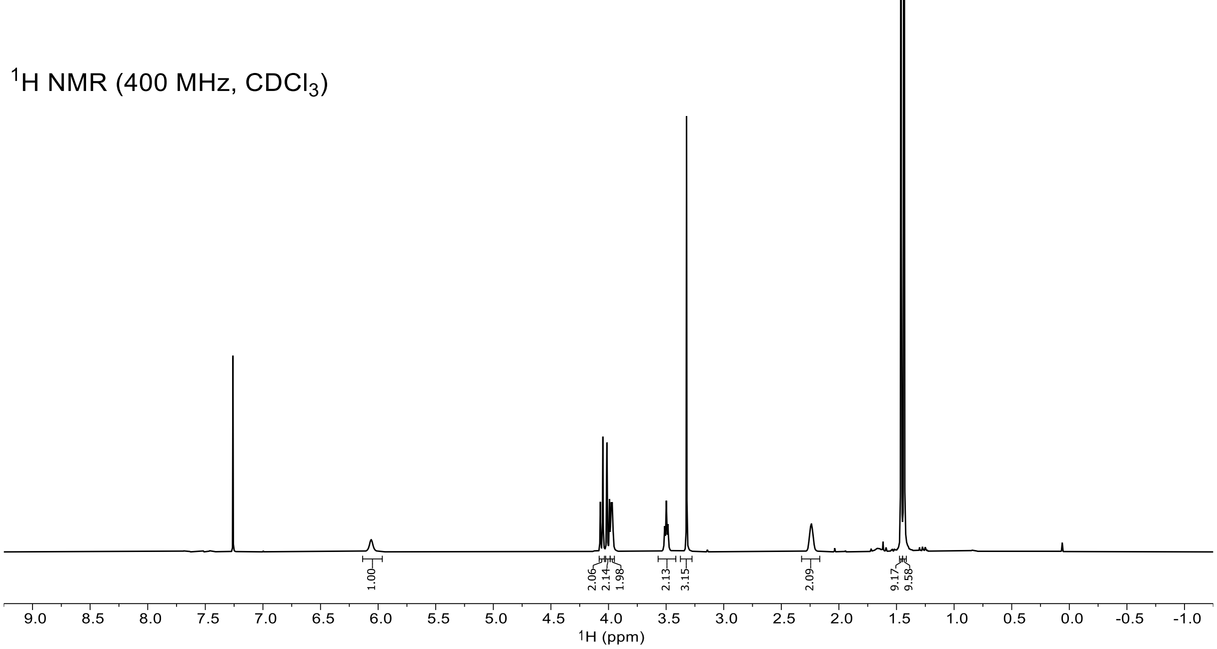
^{1}H NMR (400 MHz, CDCl$_3$)
1.00
2.06
2.14
1.98
2.13
3.15
2.09
9.17
9.58
9.0 8.5 8.0 7.5 7.0 6.5 6.0 5.5 5.0 4.5 4.0 3.5 3.0 2.5 2.0 1.5 1.0 0.5 0.0 -0.5 -1.0
^{1}H (ppm)

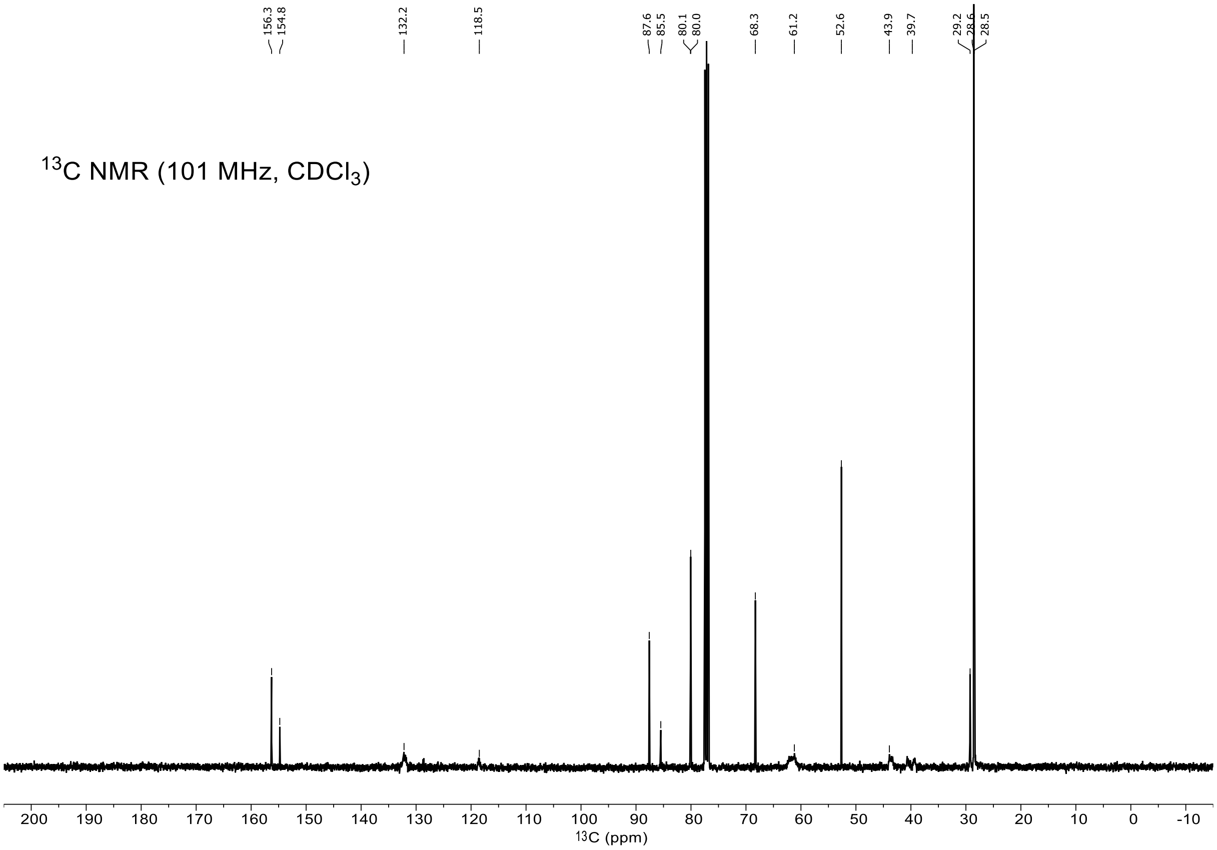
156.3
154.8
132.2
118.5
87.6
85.5
80.1
80.0
68.3
61.2
52.6
43.9
39.7
29.2
28.6
28.5
^{13}C NMR (101 MHz, CDCl$_3$)
200 190 180 170 160 150 140 130 120 110 100 90 80 70 60 50 40 30 20 10 0 -10
^{13}C (ppm)

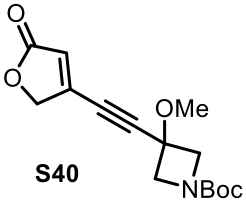

S40

^{1}H NMR (400 MHz, $CDCl_3$)

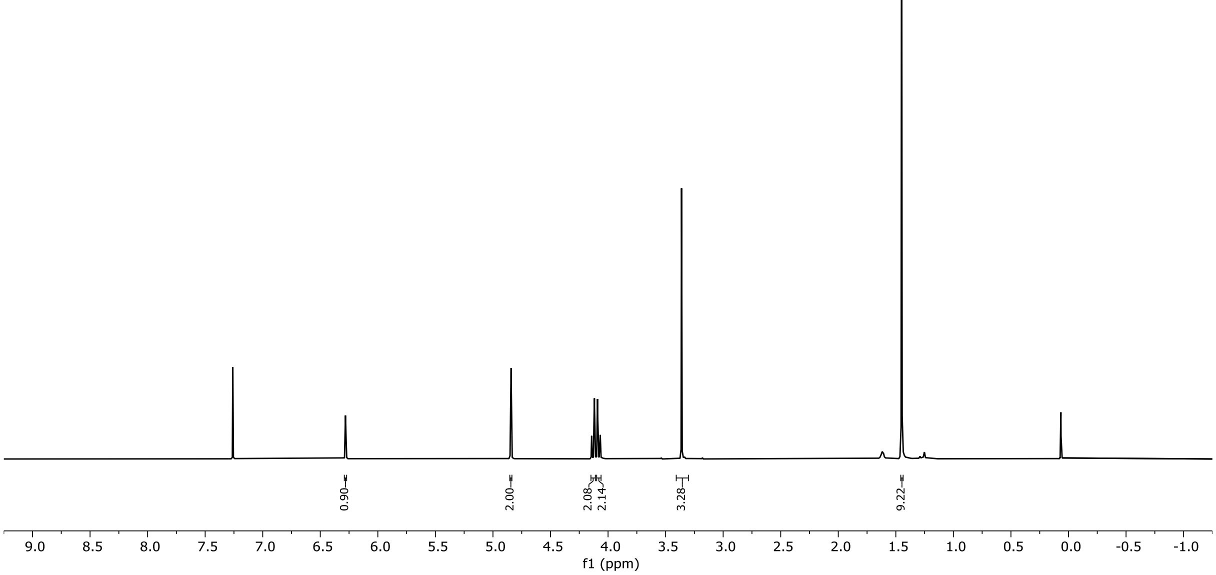

^{13}C NMR (101 MHz, $CDCl_3$)

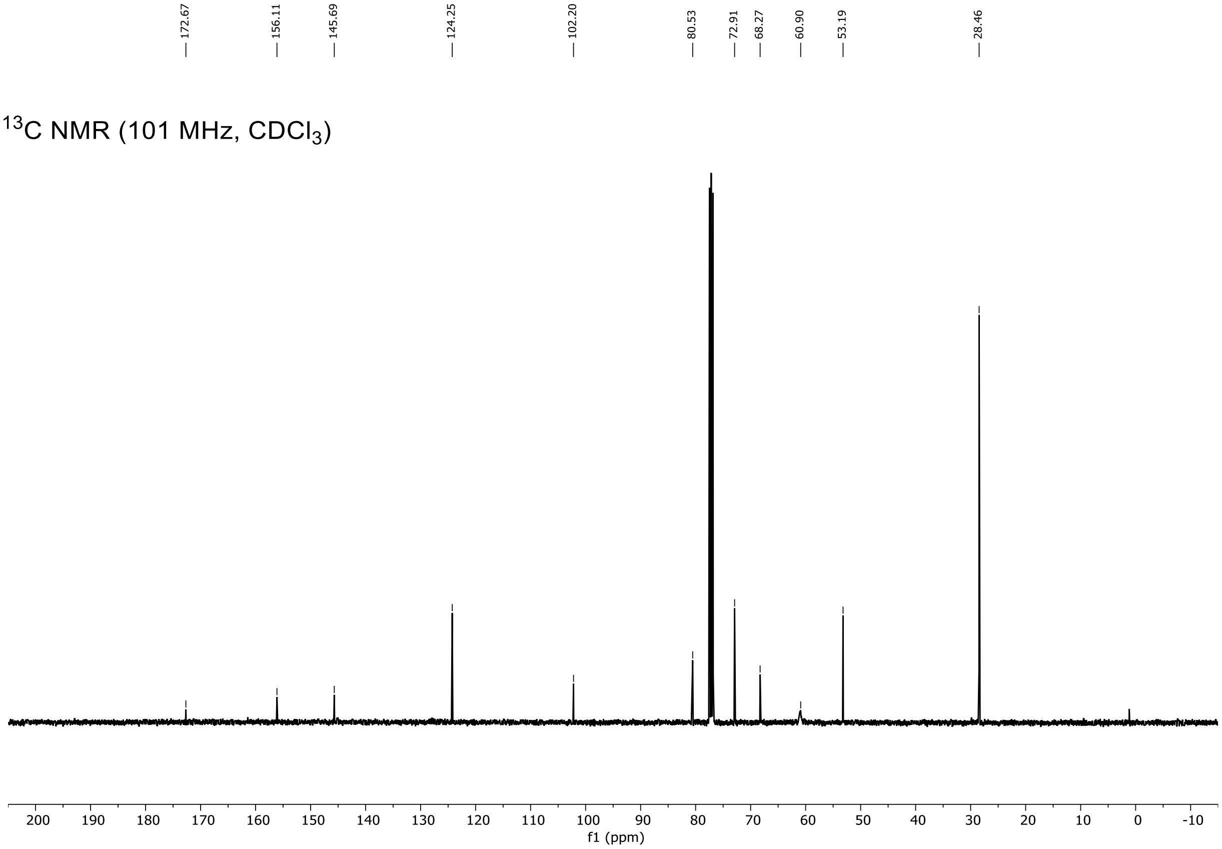

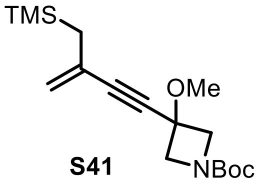

S41

^{1}H NMR (400 MHz, $CDCl_3$)

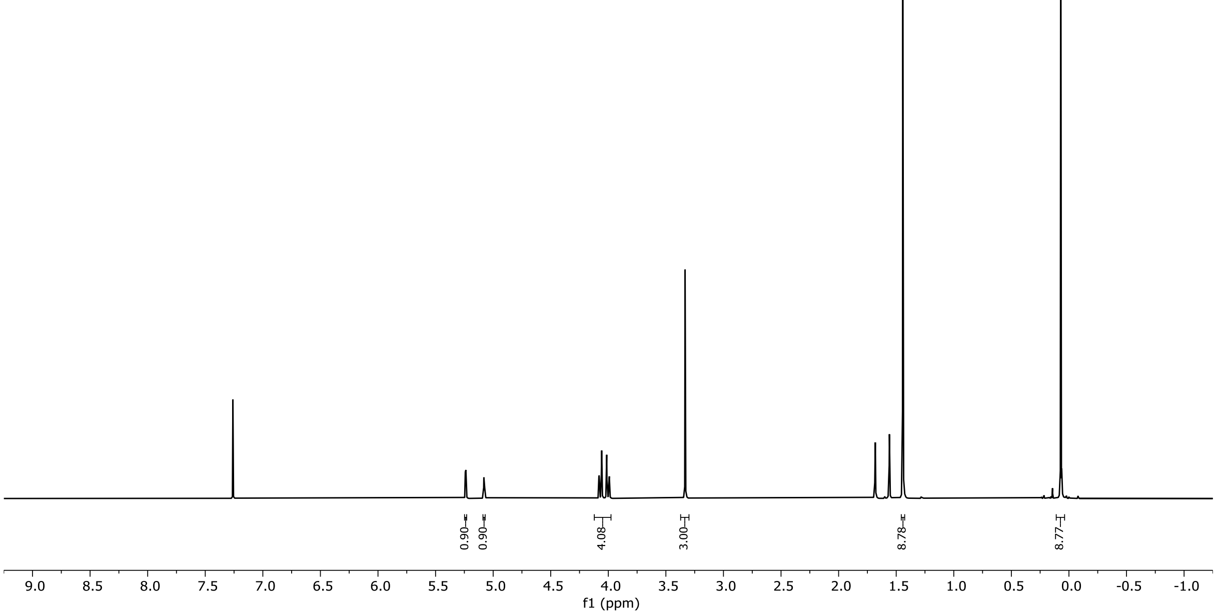

^{13}C NMR (101 MHz, $CDCl_3$)

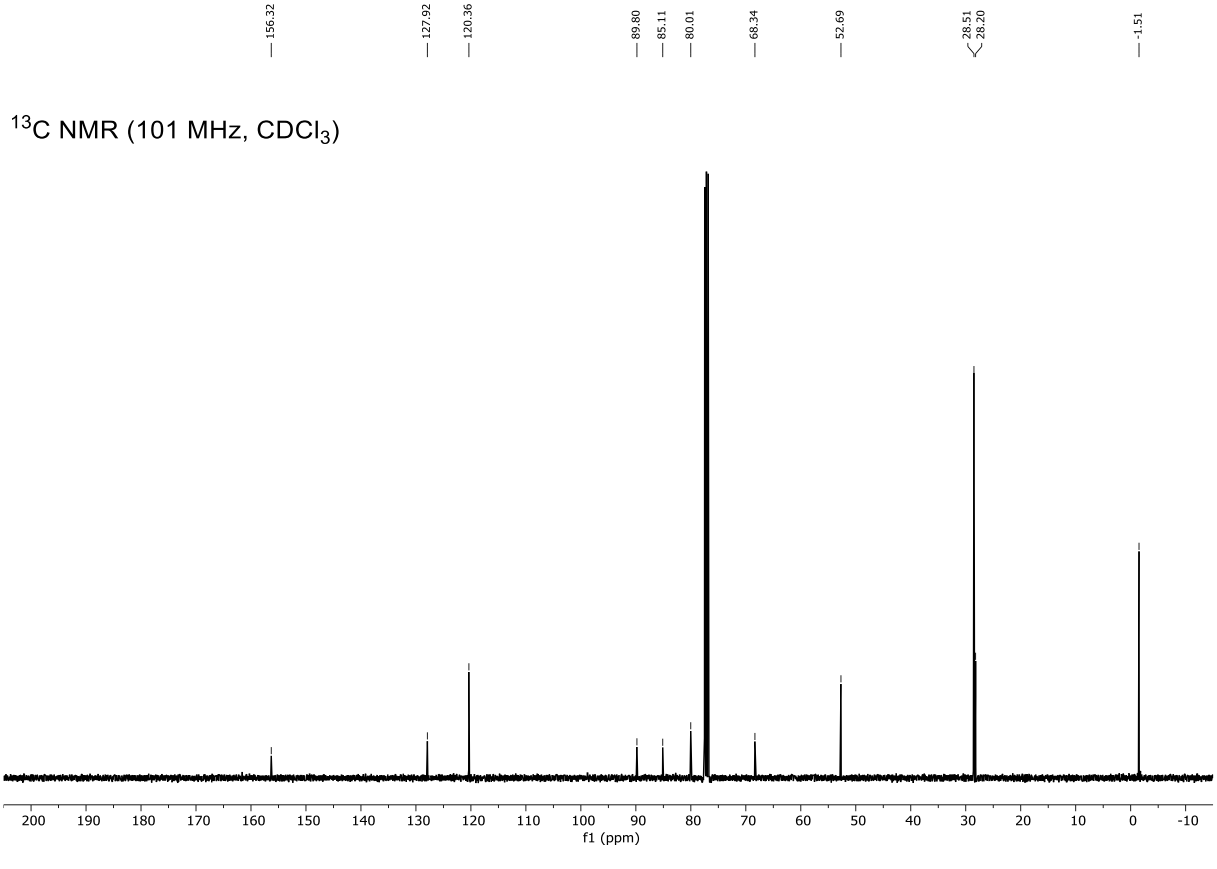

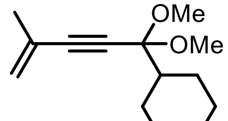

S42

^{1}H NMR (400 MHz, C_6D_6)

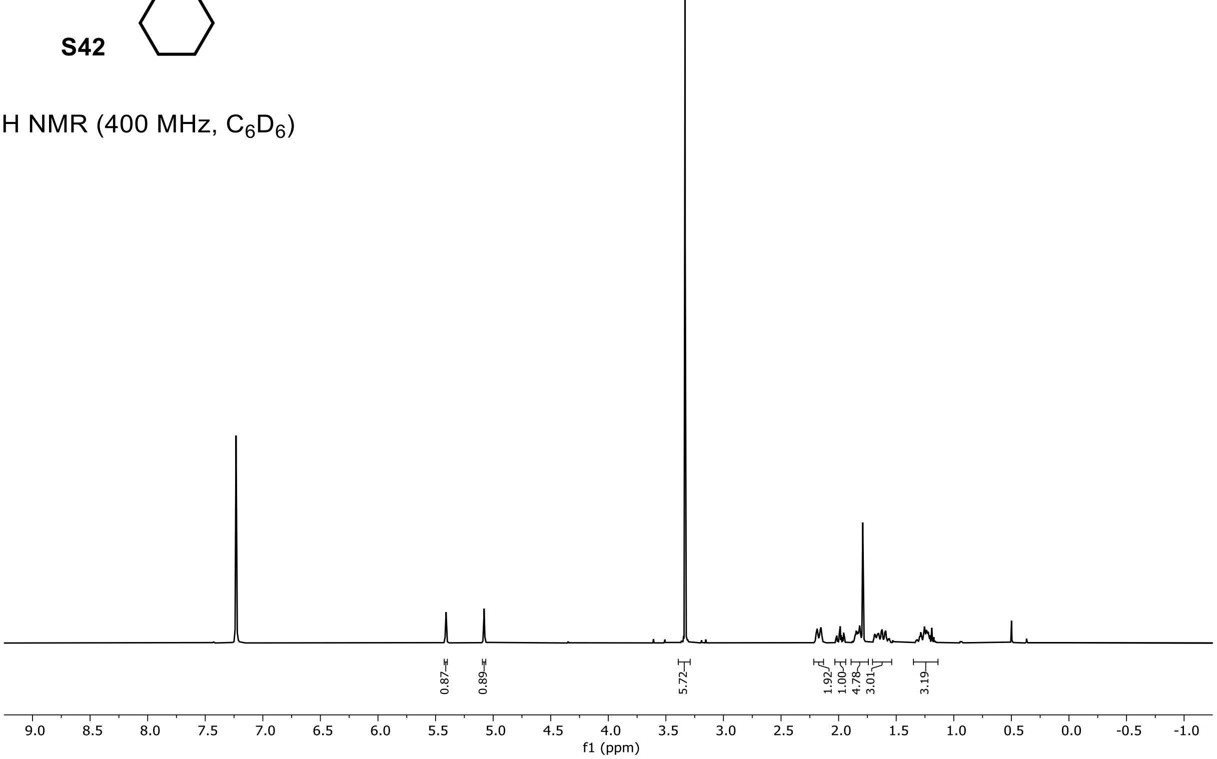

^{13}C NMR (101 MHz, C_6D_6)

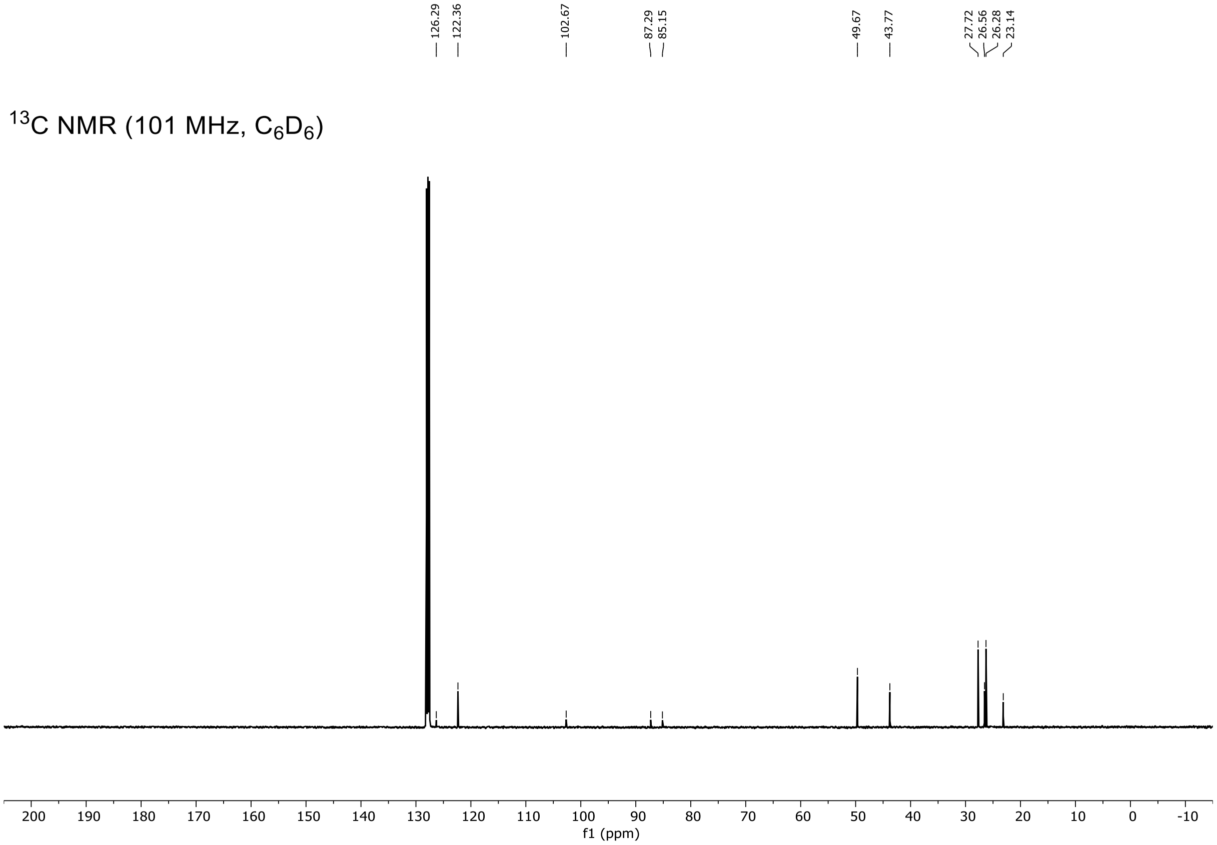

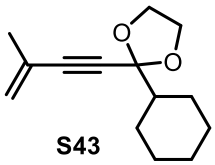

S43

^{1}H NMR (400 MHz, C_6D_6)

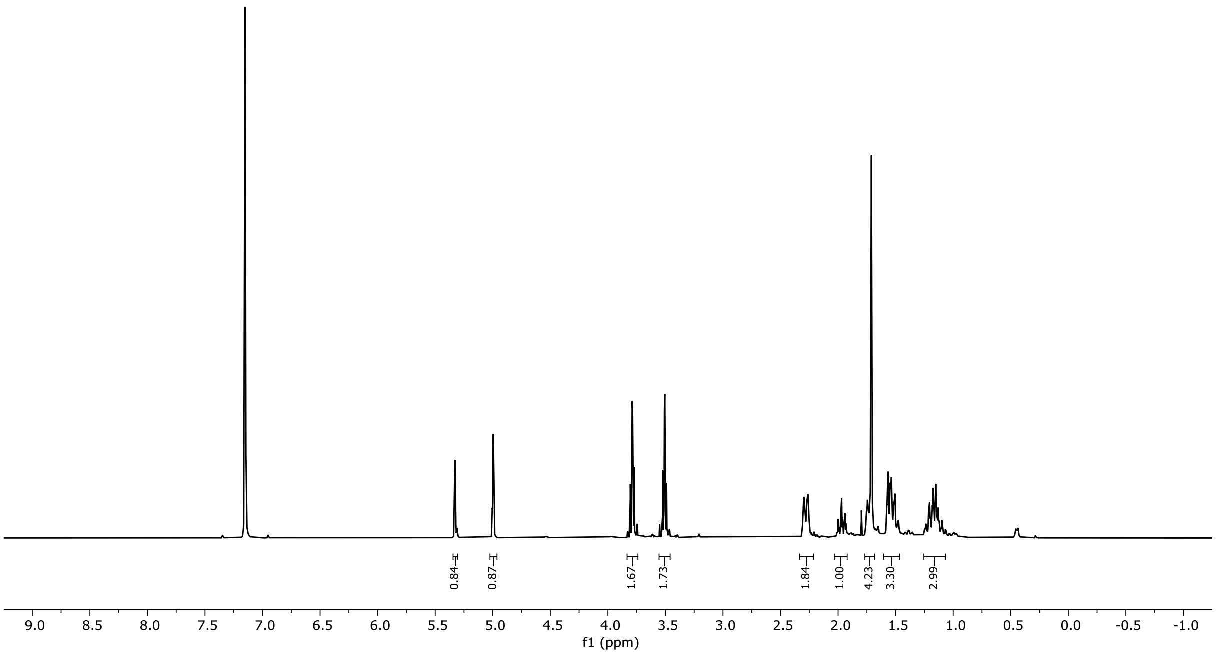

^{13}C NMR (101 MHz, C_6D_6)

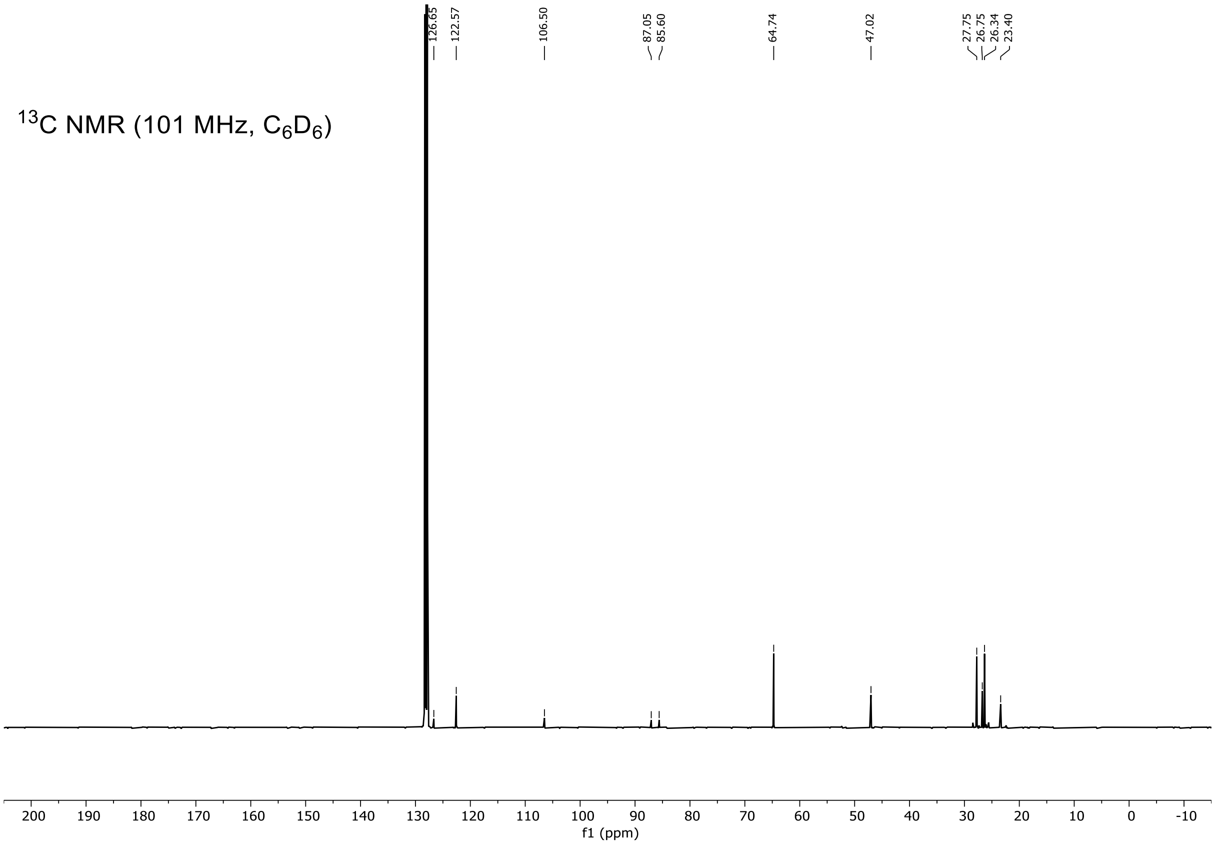

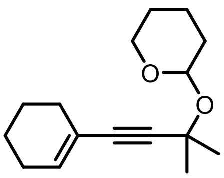

S44

^{1}H NMR (400 MHz, C_6D_6)

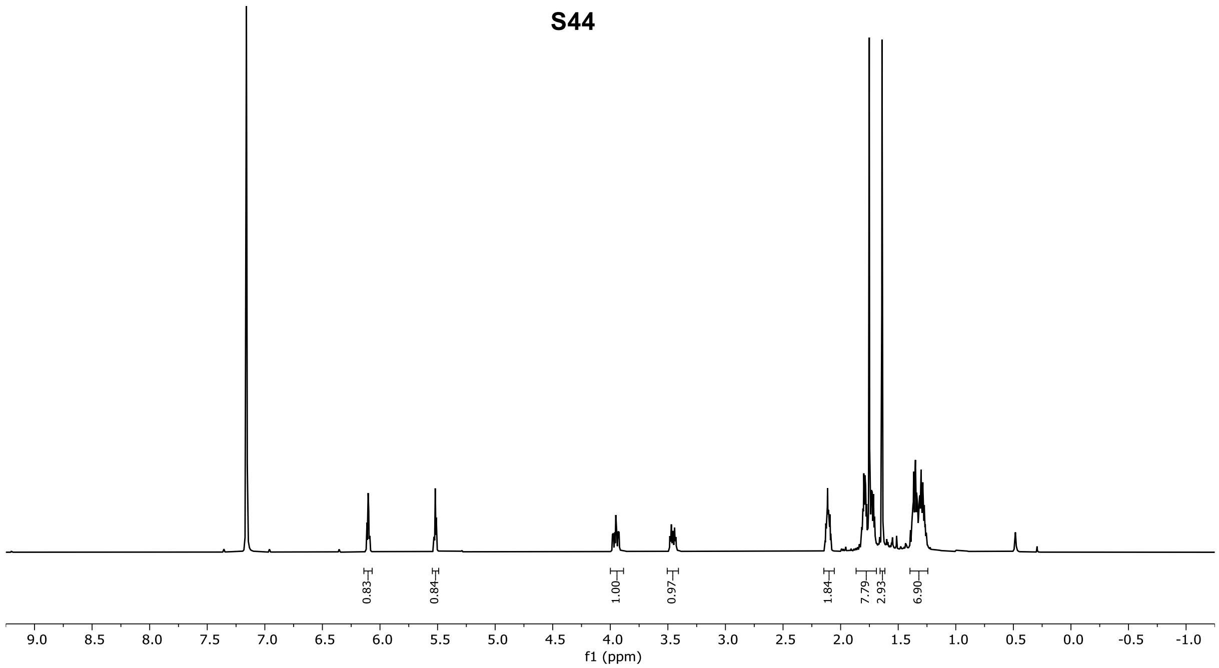

^{13}C NMR (101 MHz, C_6D_6)

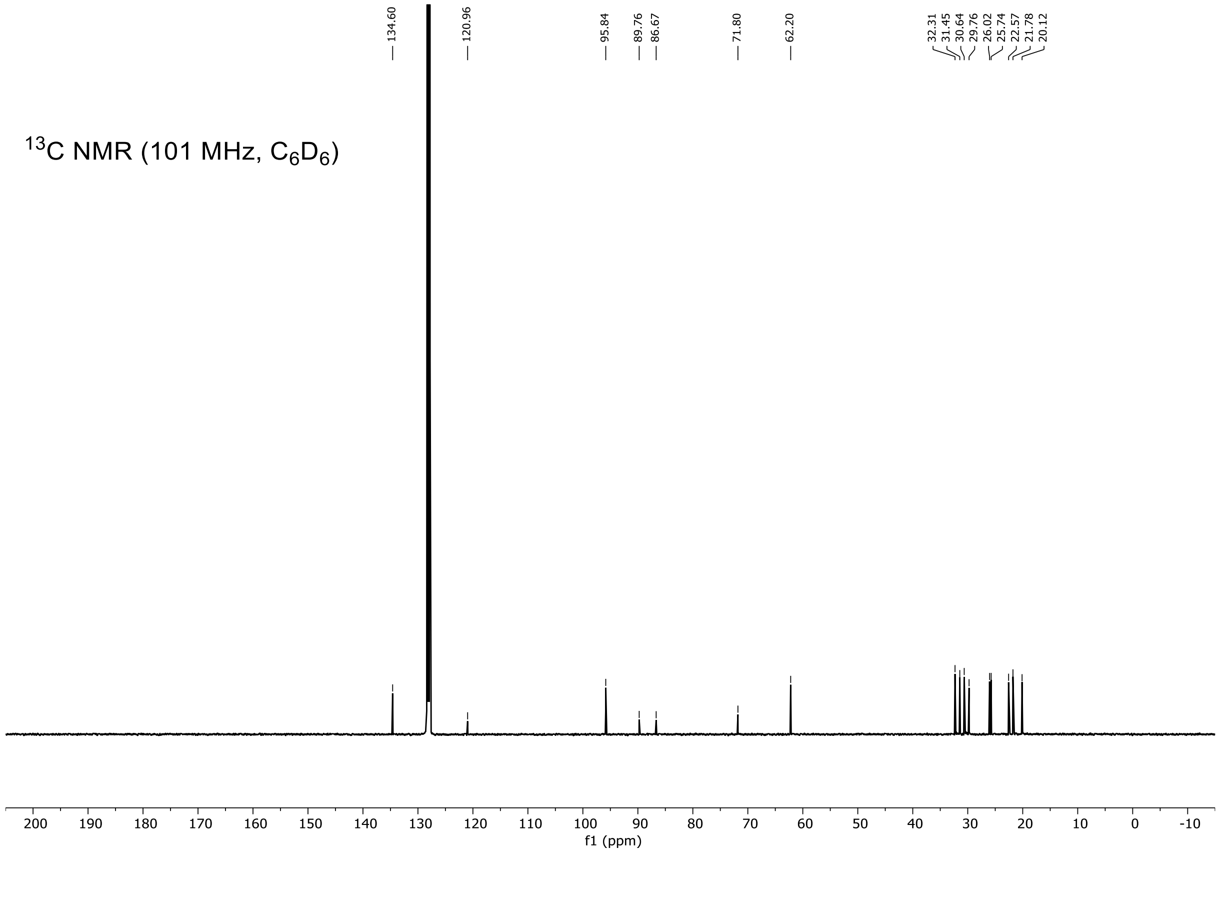

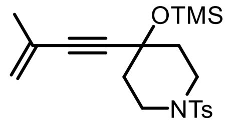

S45

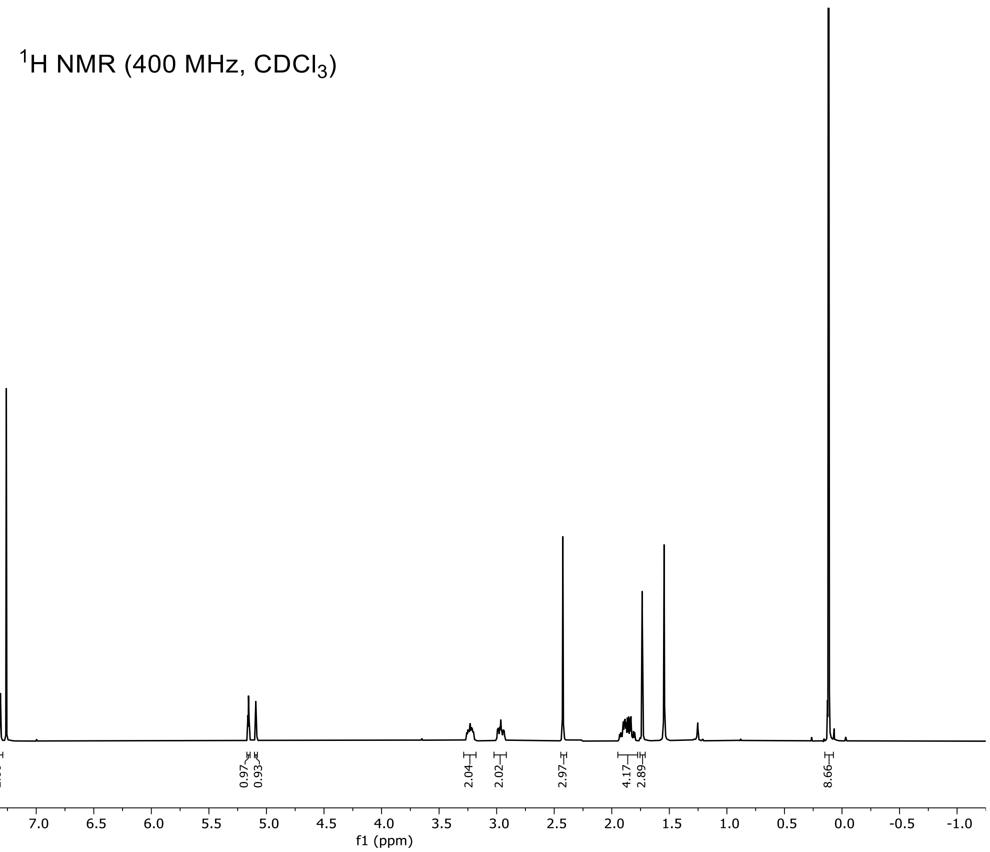

^{1}H NMR (400 MHz, $CDCl_3$)

^{13}C NMR (101 MHz, $CDCl_3$)

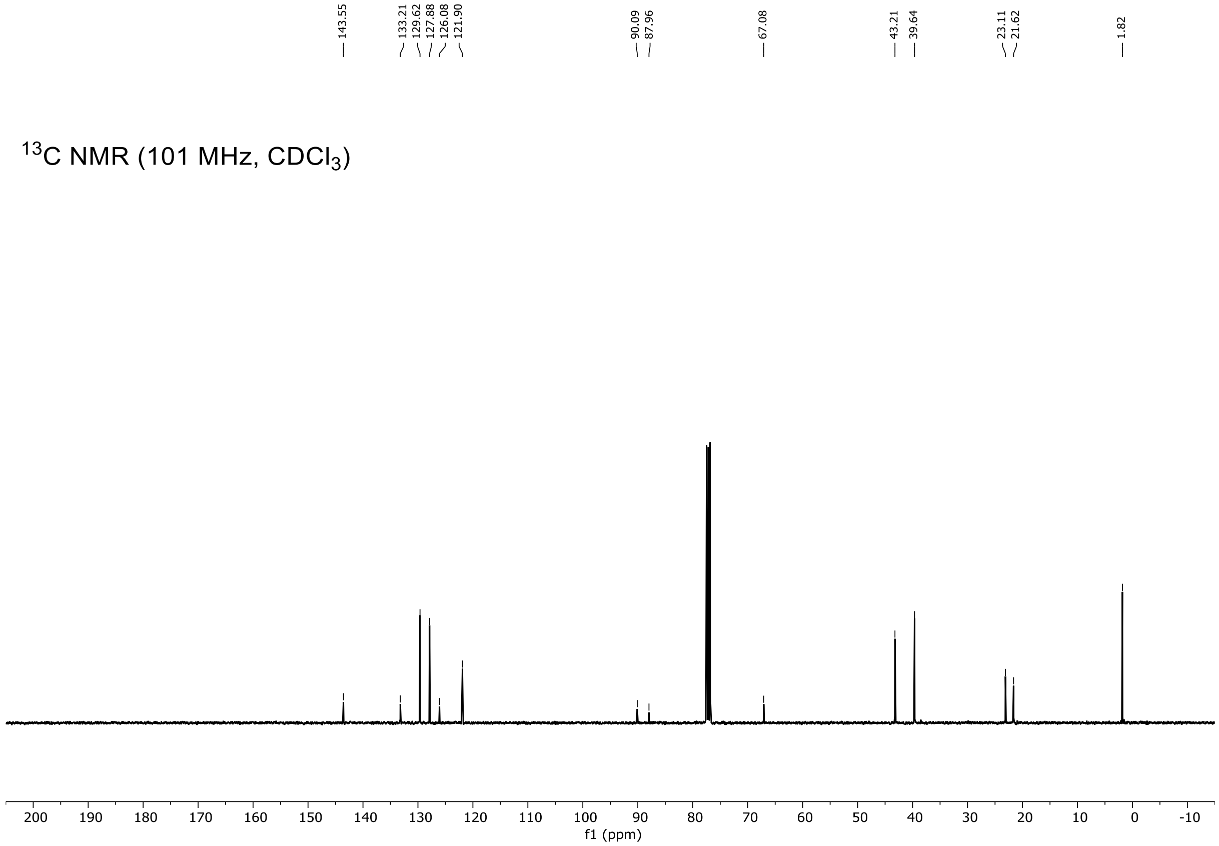

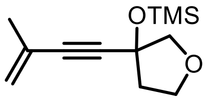

S46

^{1}H NMR (400 MHz, $CDCl_3$)

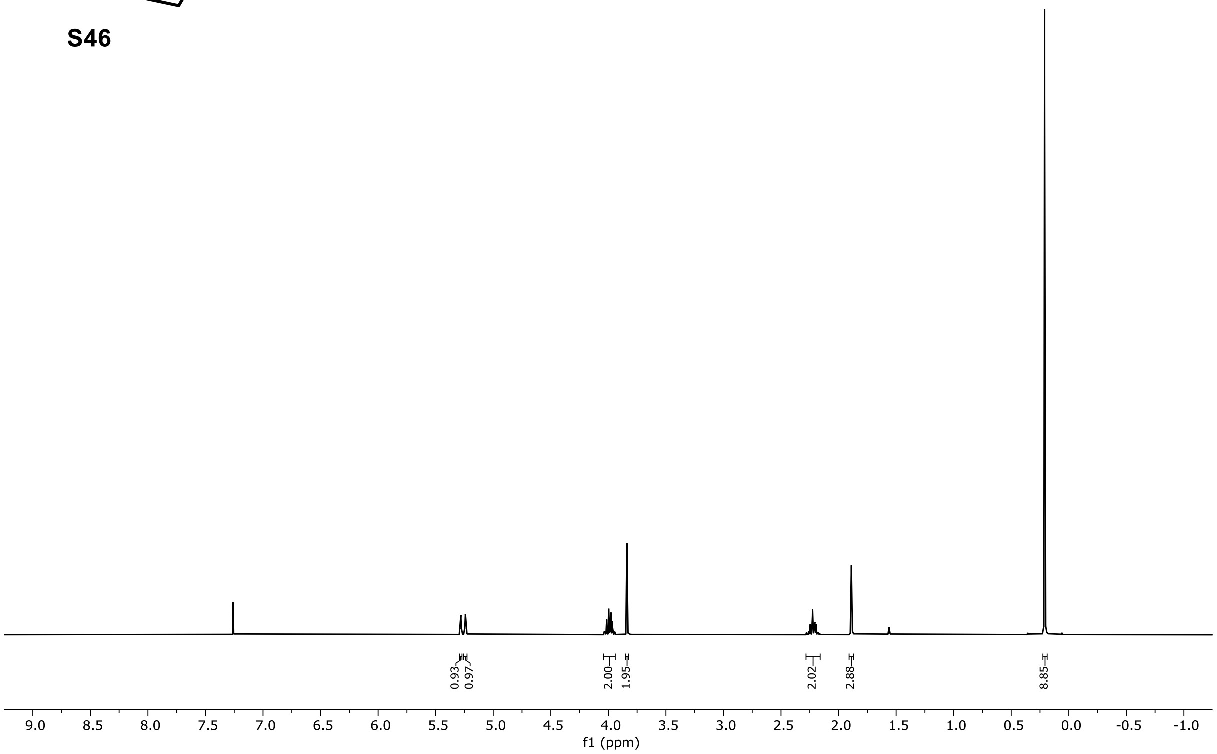

^{13}C NMR (101 MHz, $CDCl_3$)

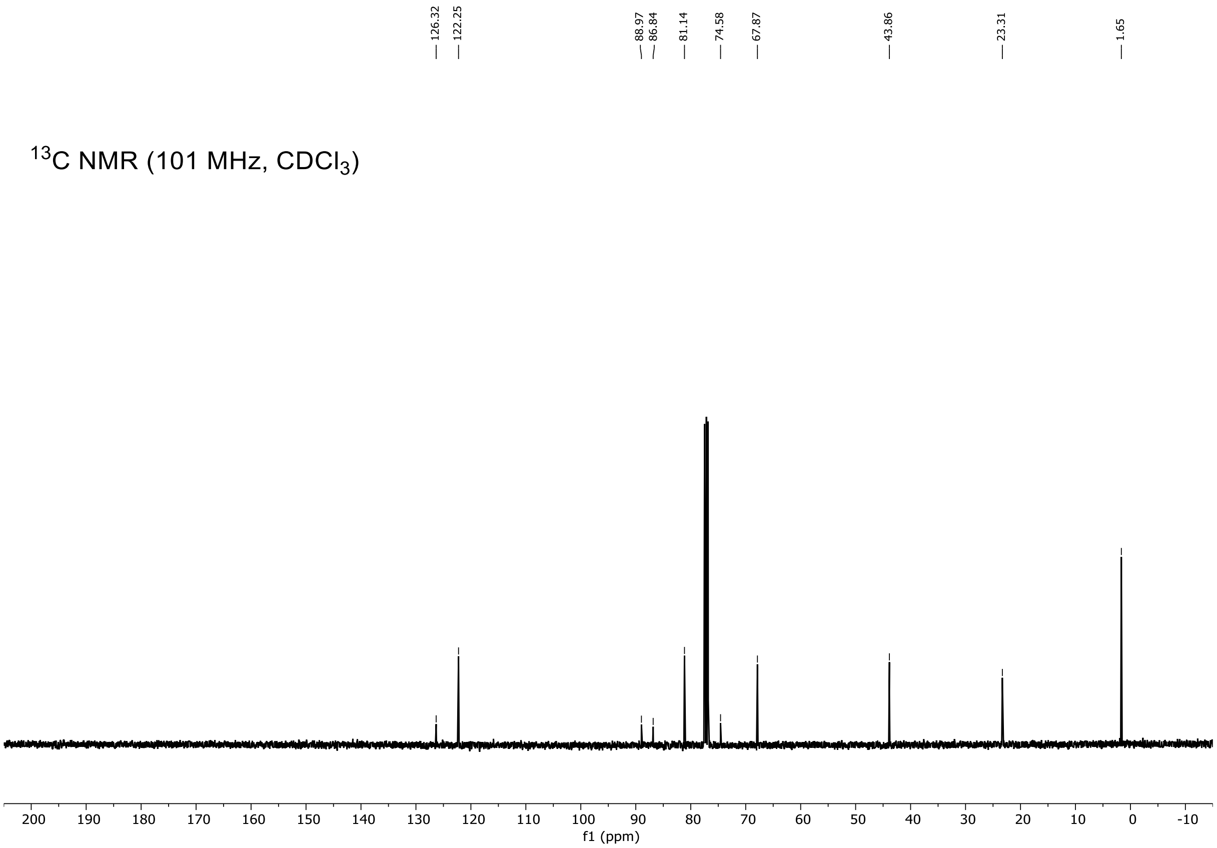

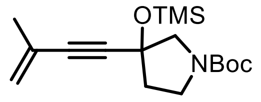

S47

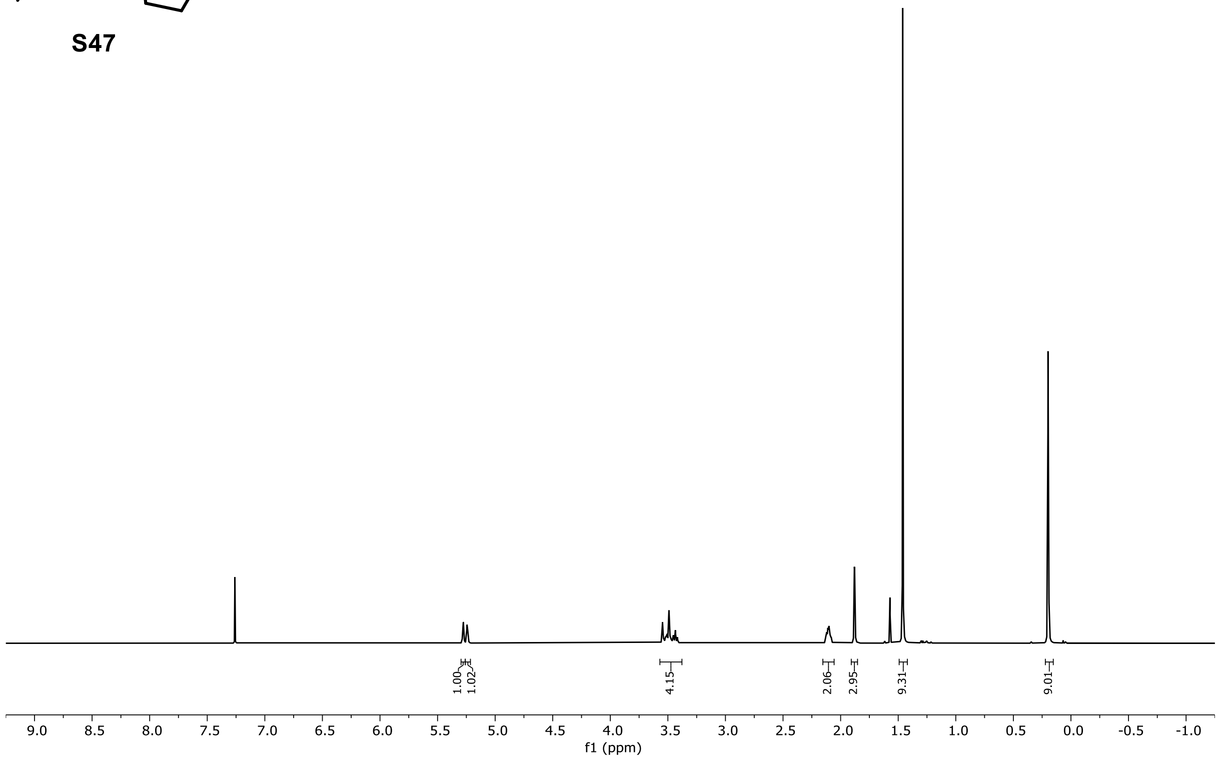

1H NMR (400 MHz, CDCl3)
1.00
1.02
4.15
2.06
2.95
9.31
9.01
9.0
8.5
8.0
7.5
7.0
6.5
6.0
5.5
5.0
4.5
4.0
3.5
3.0
2.5
2.0
1.5
1.0
0.5
0.0
-0.5
-1.0
f1 (ppm)

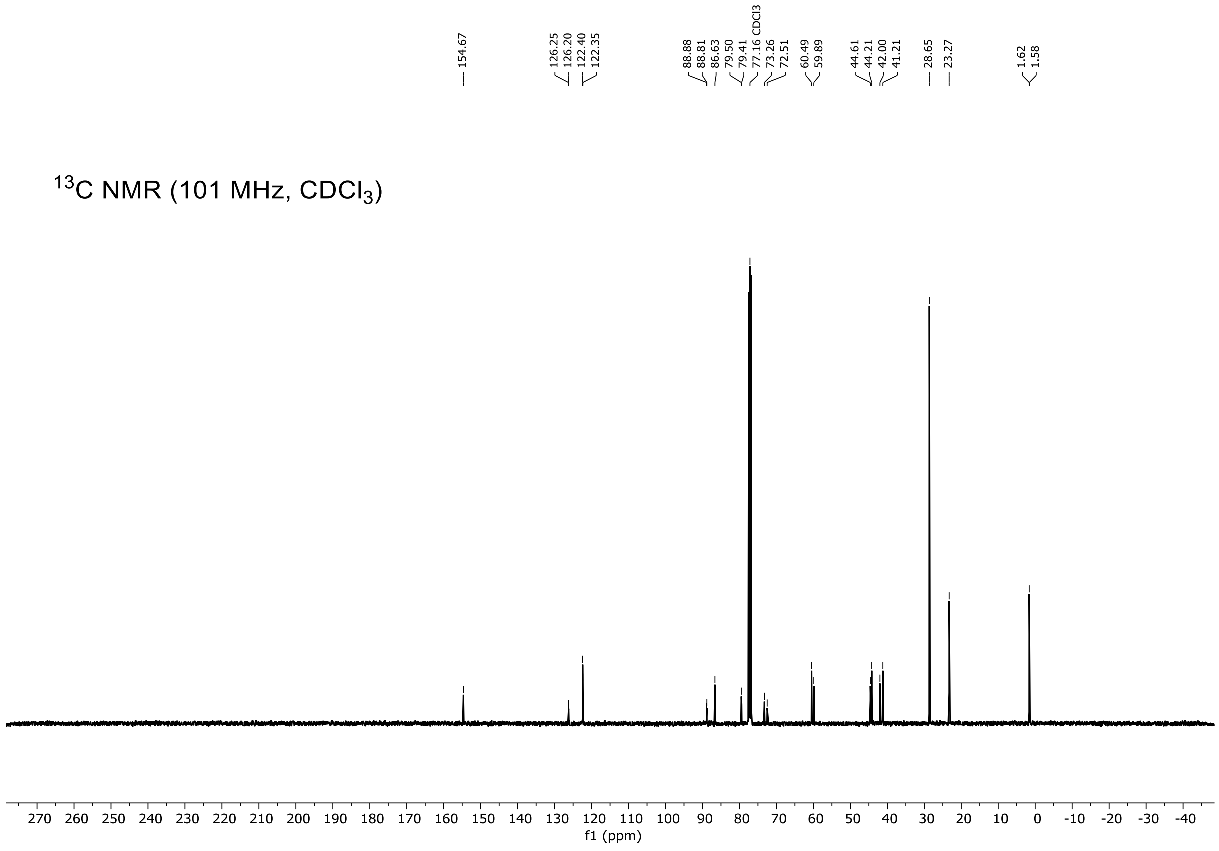

154.67
126.25
126.20
122.40
122.35
88.88
88.81
86.63
79.50
79.41
77.16 CDCl3
73.26
72.51
60.49
59.89
44.61
44.21
42.00
41.21
28.65
23.27
1.62
1.58
13C NMR (101 MHz, CDCl3)
270
260
250
240
230
220
210
200
190
180
170
160
150
140
130
120
110
100
90
80
70
60
50
40
30
20
10
0
-10
-20
-30
-40
f1 (ppm)

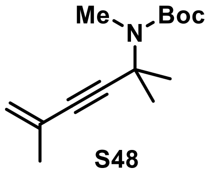

S48

^{1}H NMR (400 MHz, $CDCl_3$)

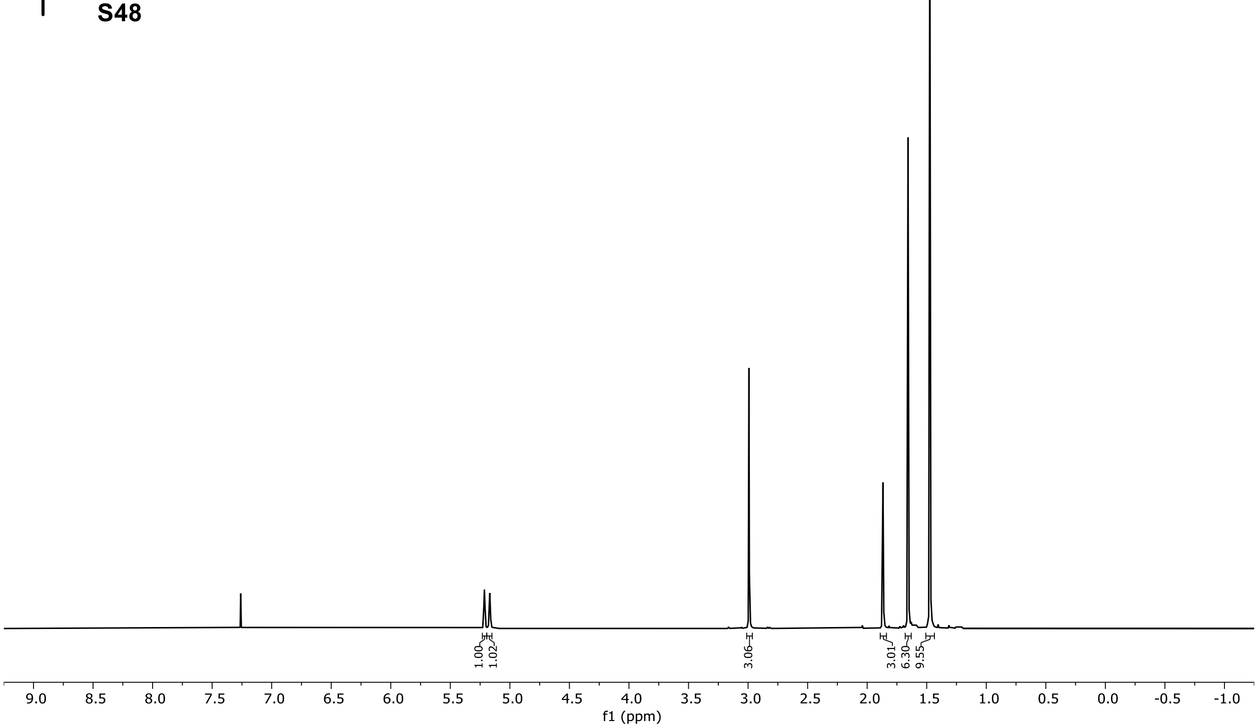

^{13}C NMR (101 MHz, $CDCl_3$)

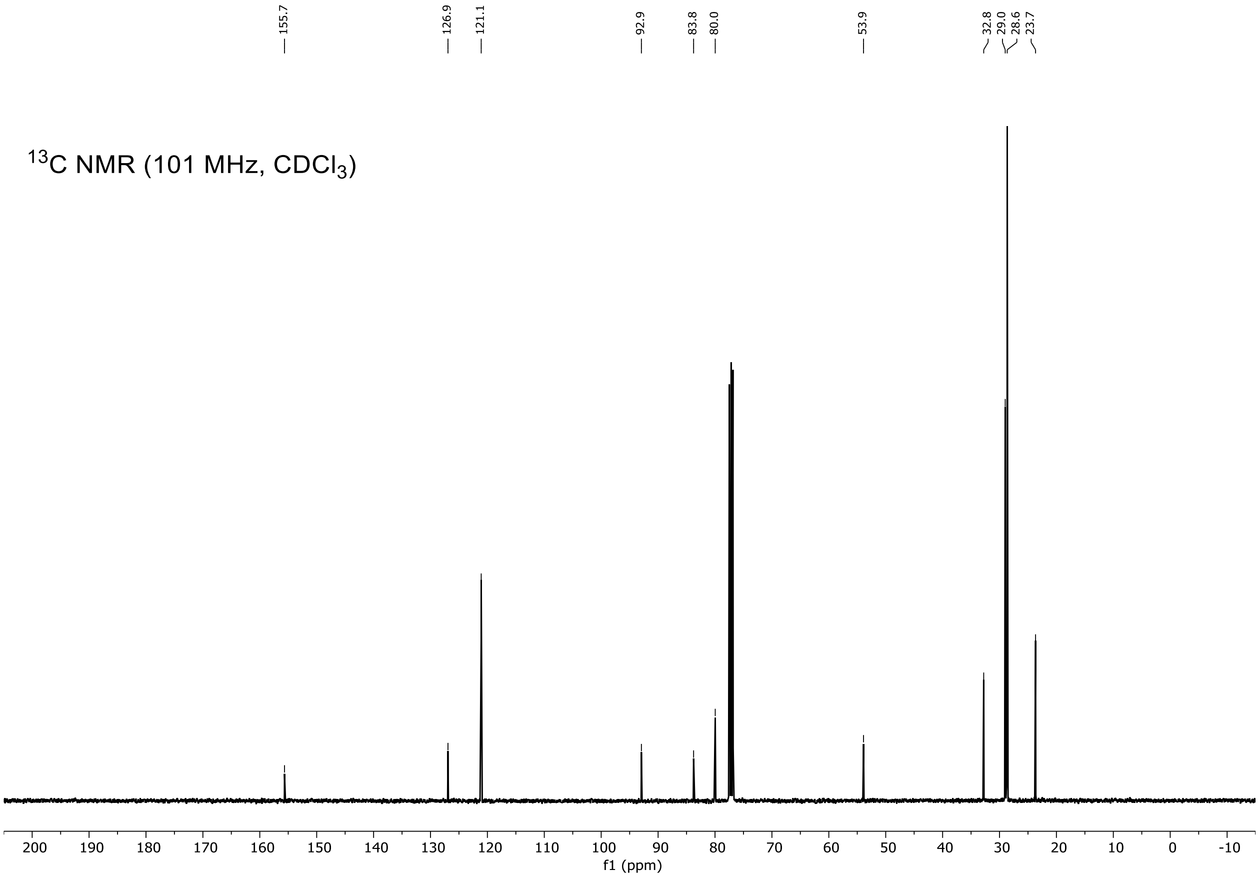

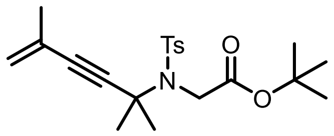

S49

^1H NMR (400 MHz, $CDCl_3$)

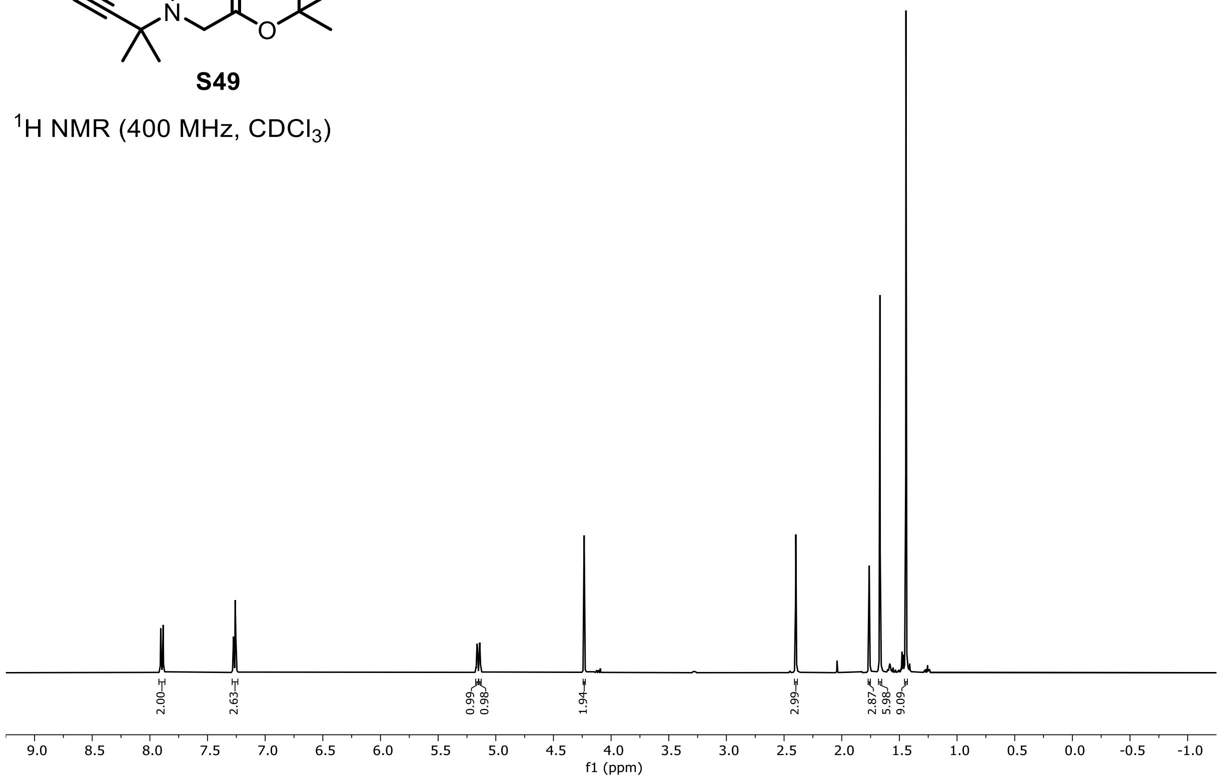

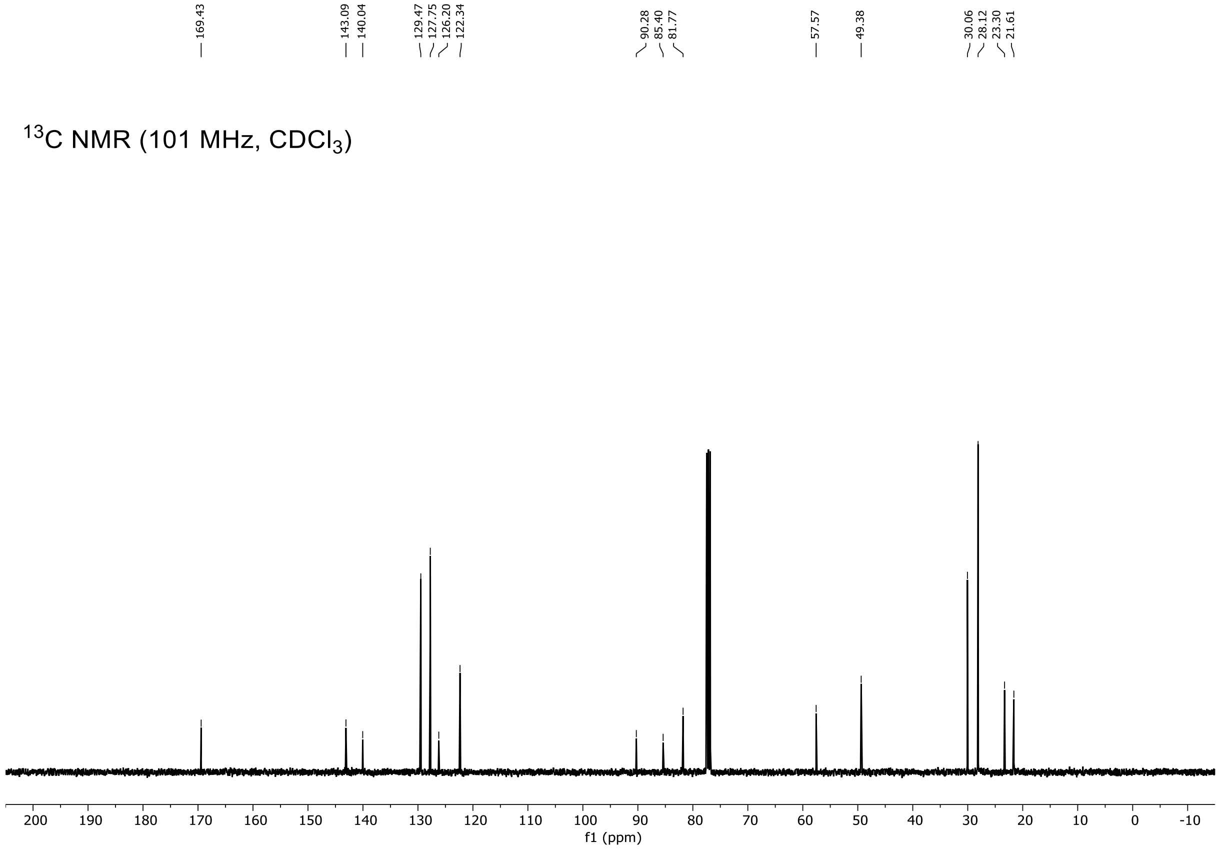

^{13}C NMR (101 MHz, $CDCl_3$)

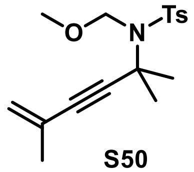

S50

^1H NMR (400 MHz, $CDCl_3$)

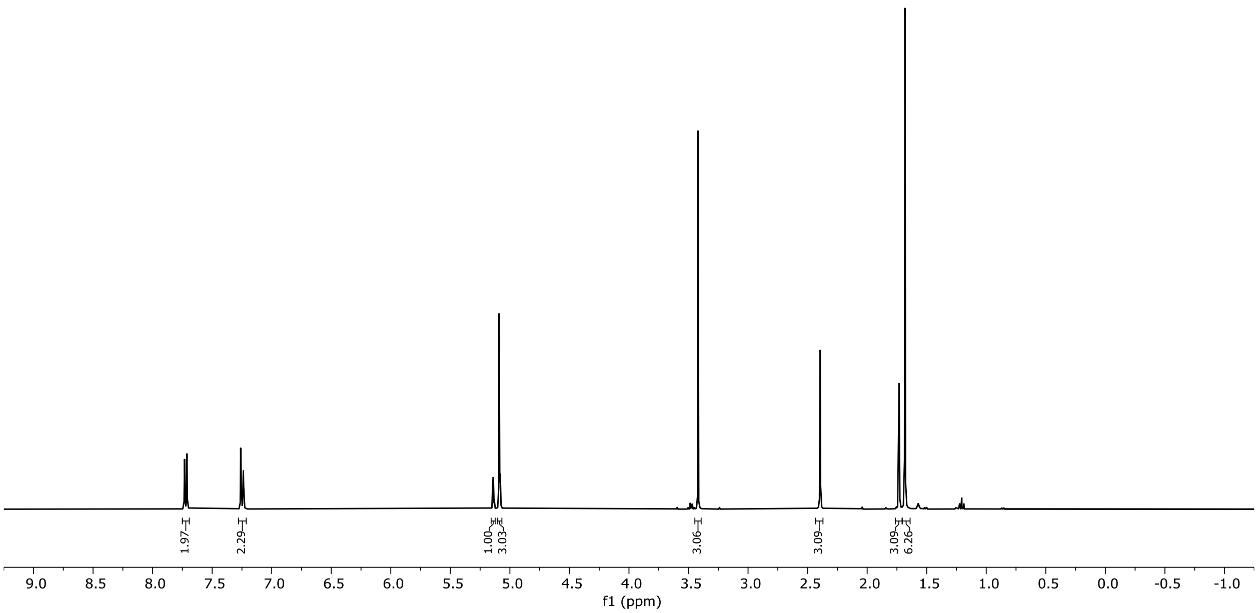

^{13}C NMR (101 MHz, $CDCl_3$)

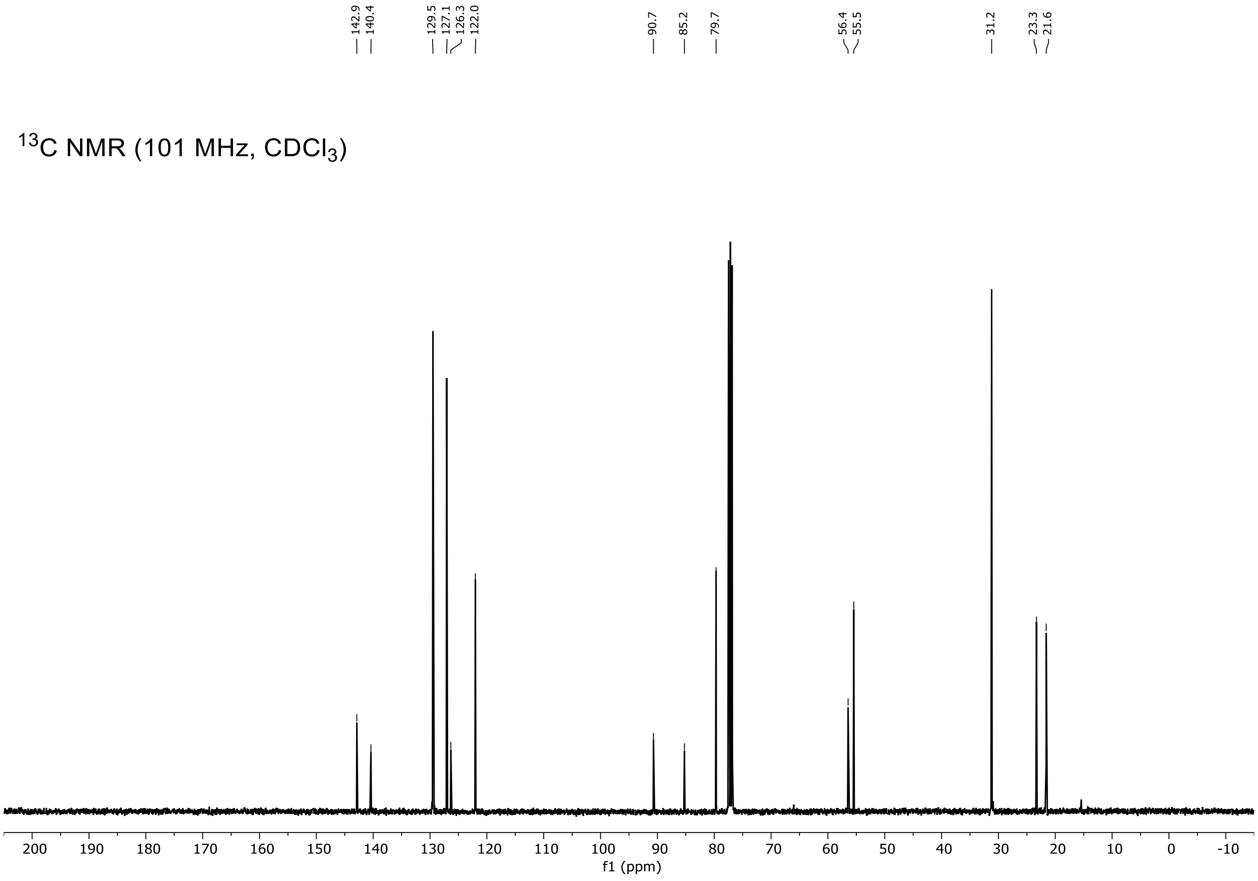

Products of *gem*-Hydrogenation / C–H Insertion

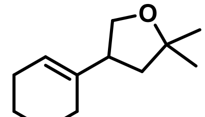

7

^{1}H NMR (400 MHz, $CDCl_3$)

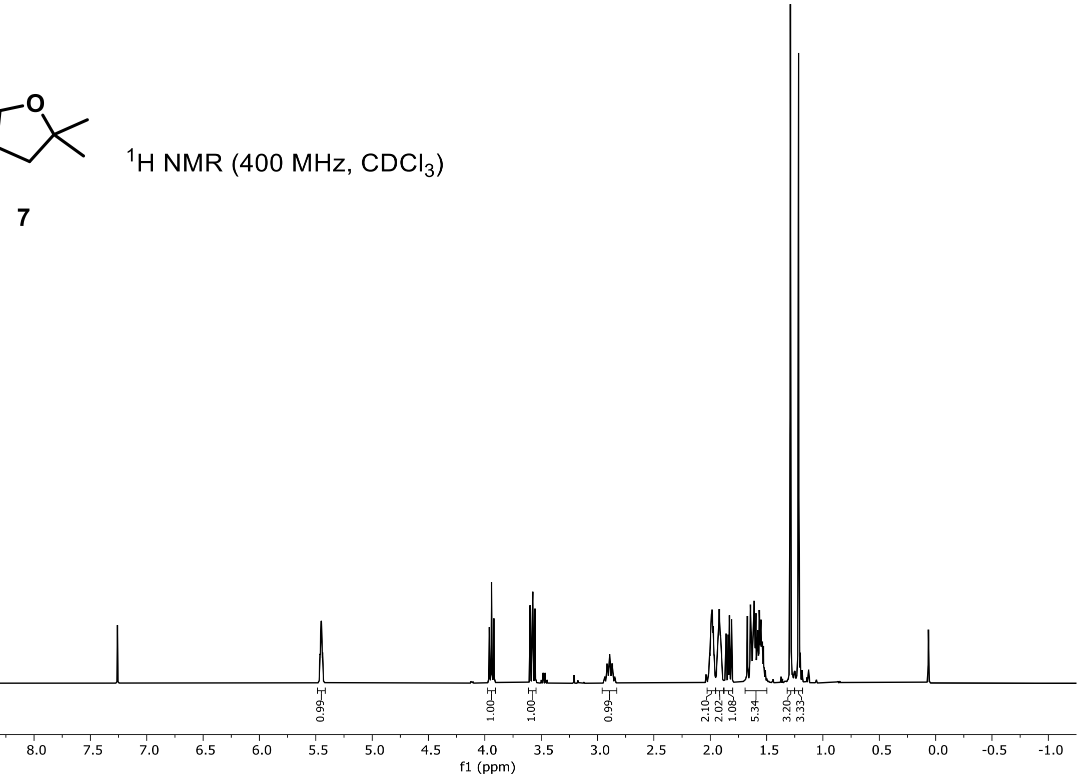

^{13}C NMR (101 MHz, $CDCl_3$)

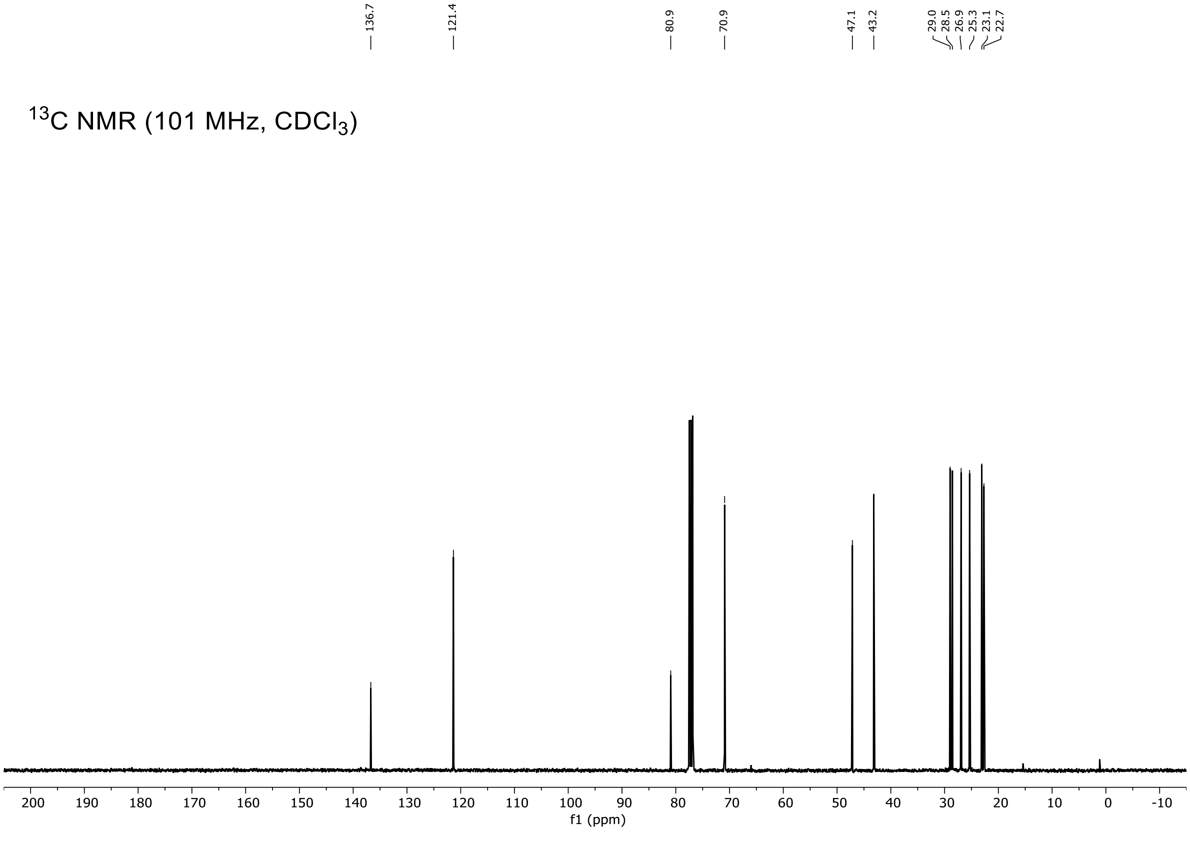

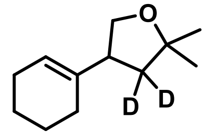

[D$_2$]-7

^{1}H NMR (600 MHz, $CDCl_3$)

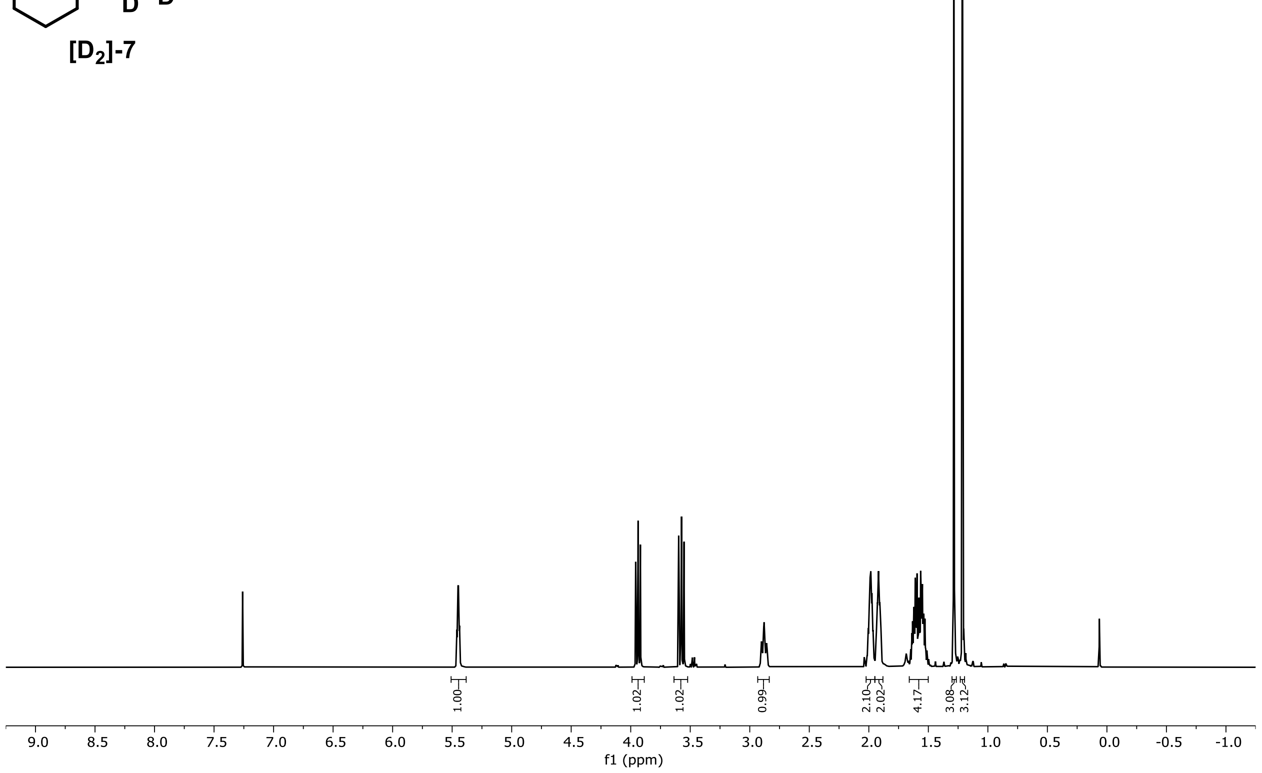

^{13}C NMR (151 MHz, $CDCl_3$)

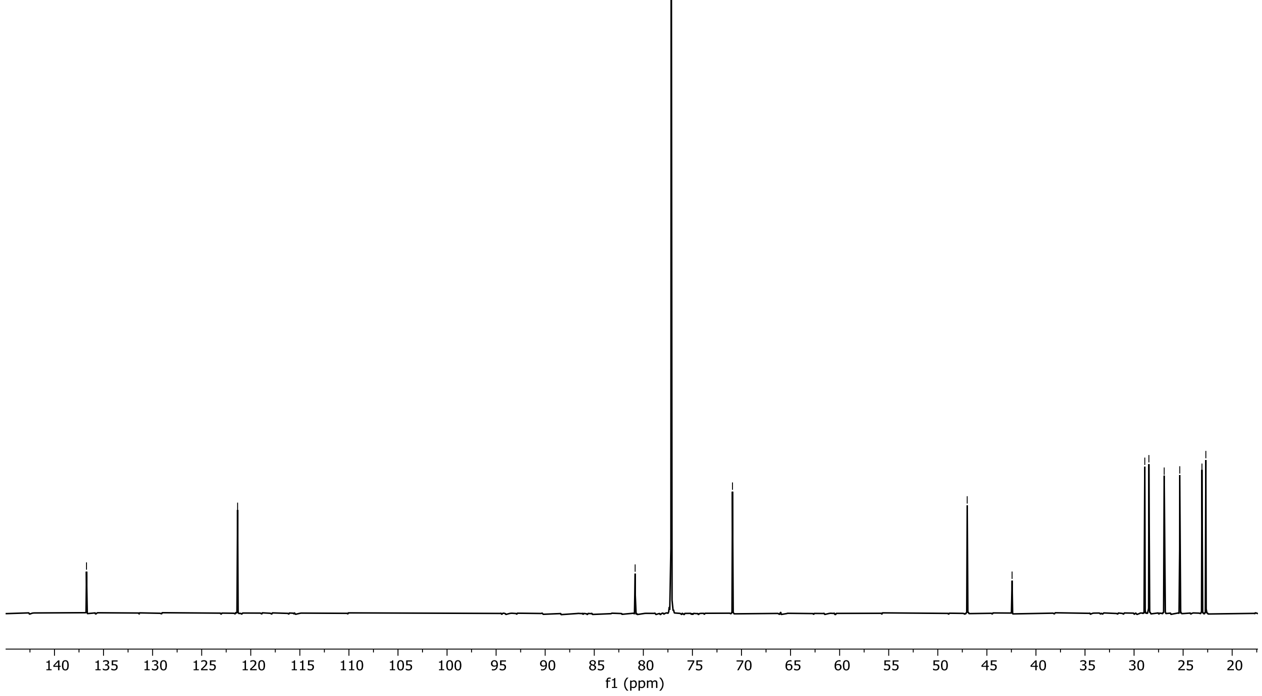

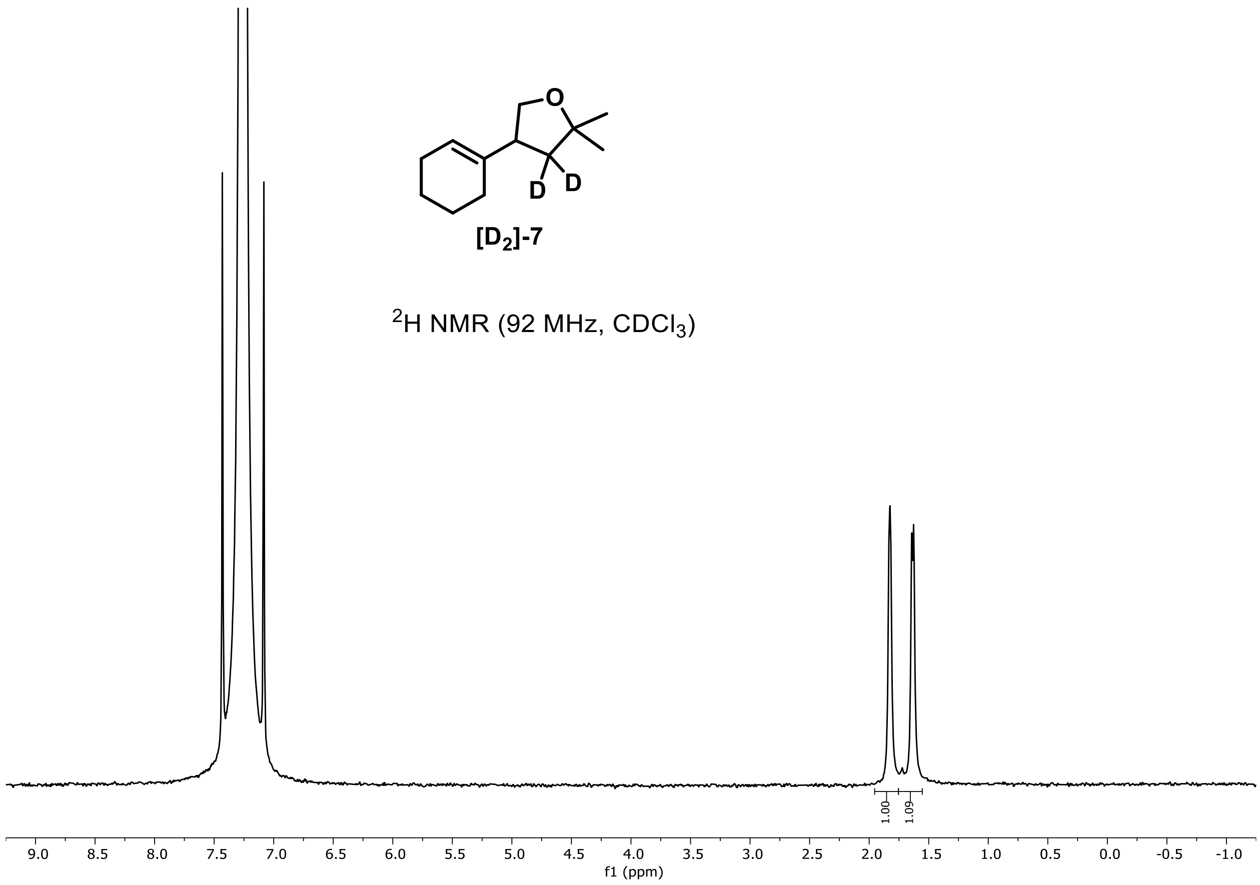

O
D
D
[D2]-7
2H NMR (92 MHz, CDCl3)
1.00
1.09
9.0 8.5 8.0 7.5 7.0 6.5 6.0 5.5 5.0 4.5 4.0 3.5 3.0 2.5 2.0 1.5 1.0 0.5 0.0 -0.5 -1.0
f1 (ppm)

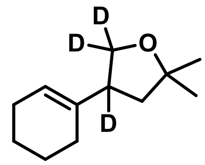

[D$_3$]-7

^{1}H NMR (600 MHz, $CDCl_3$)

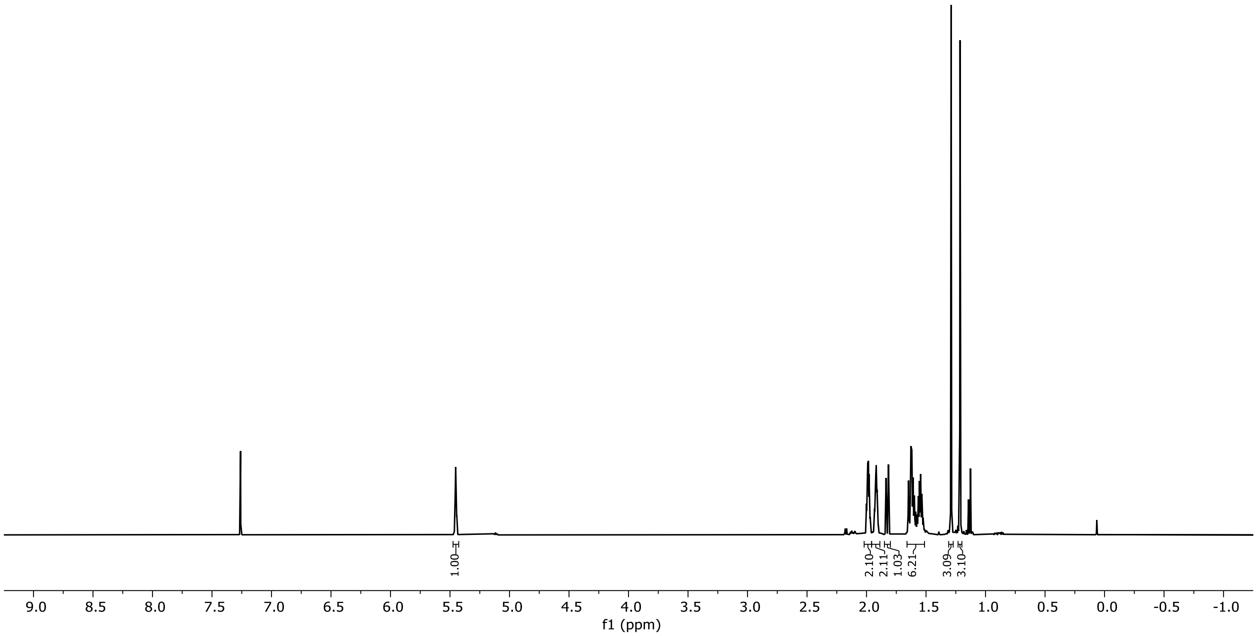

^{13}C NMR (151 MHz, $CDCl_3$)

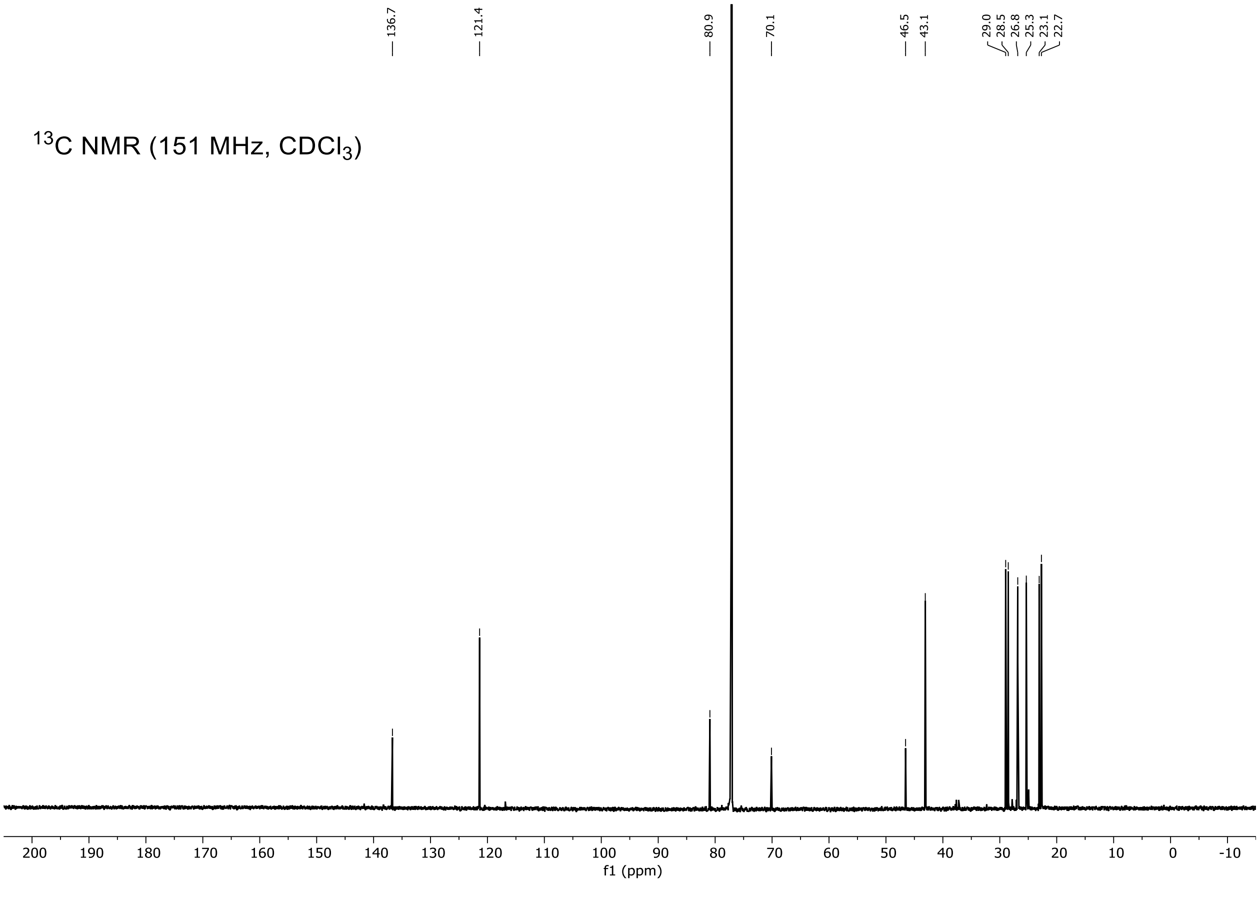

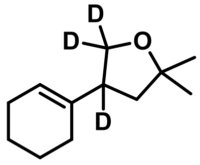

[D$_3$]-7

^{2}H NMR (92 MHz, $CDCl_3$)

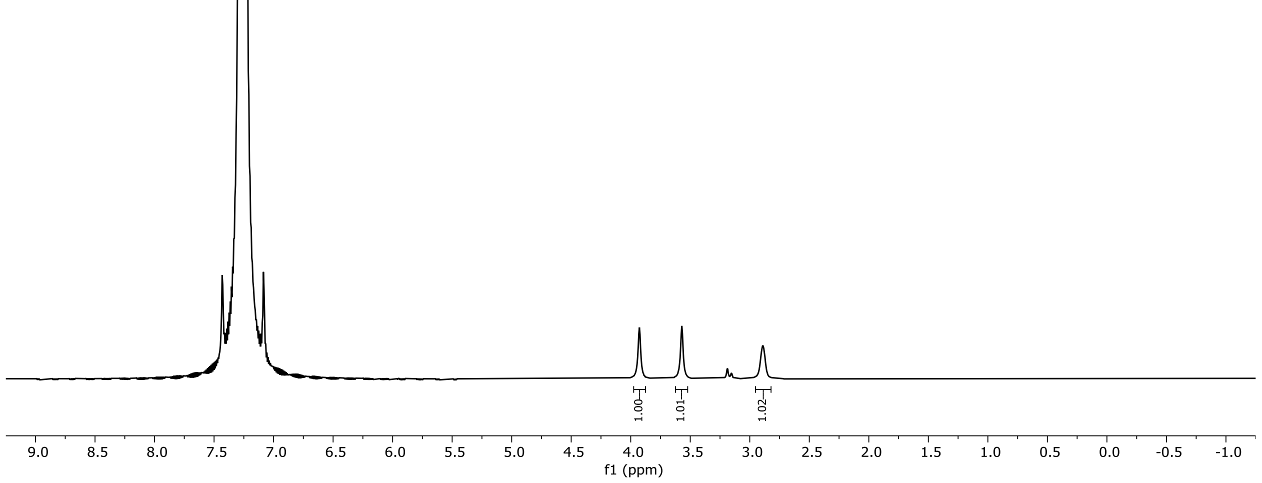

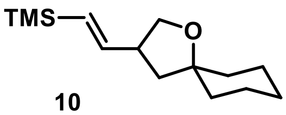

10

^{1}H NMR (400 MHz, $CDCl_3$)

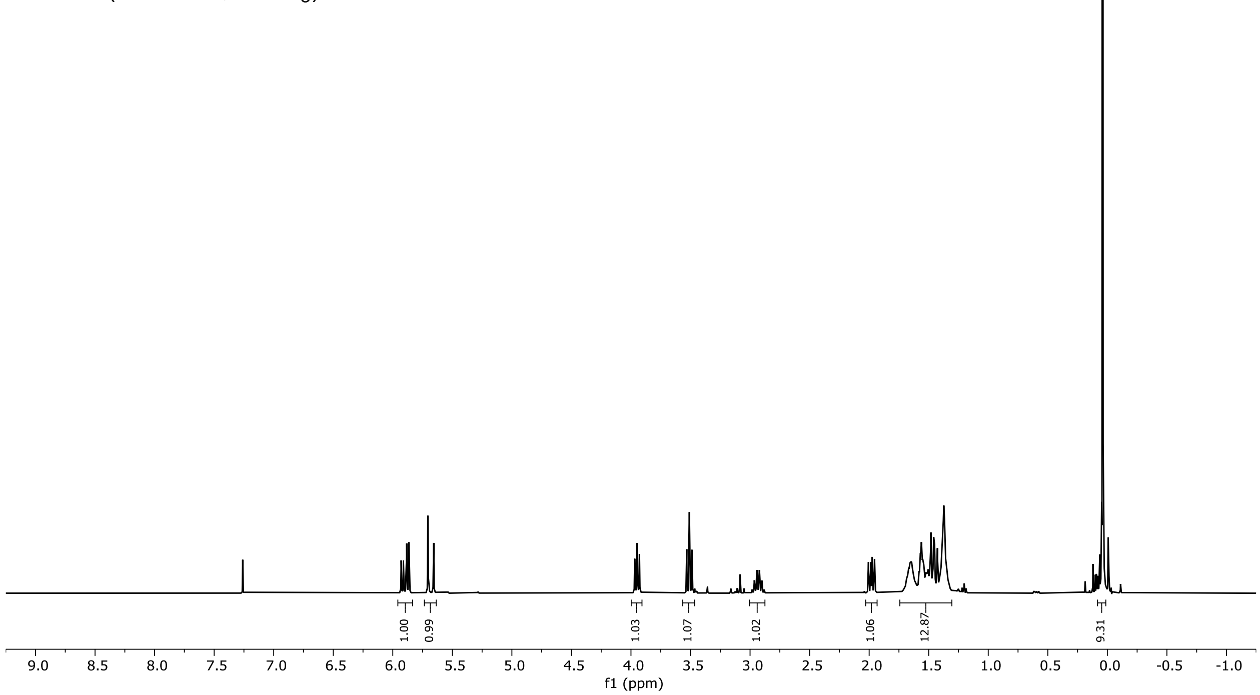

^{13}C NMR (101 MHz, $CDCl_3$)

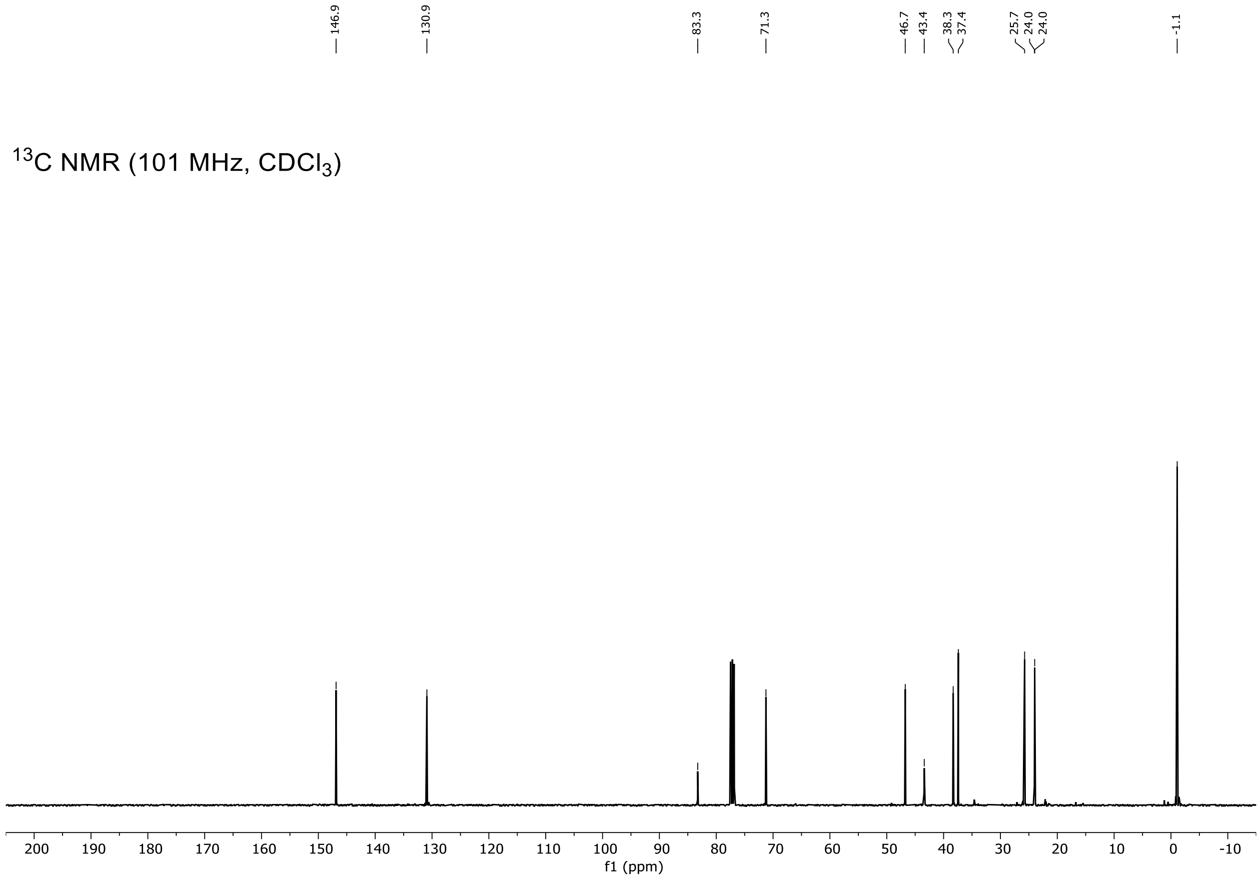

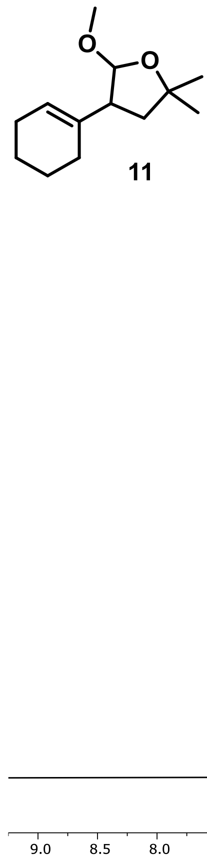

11

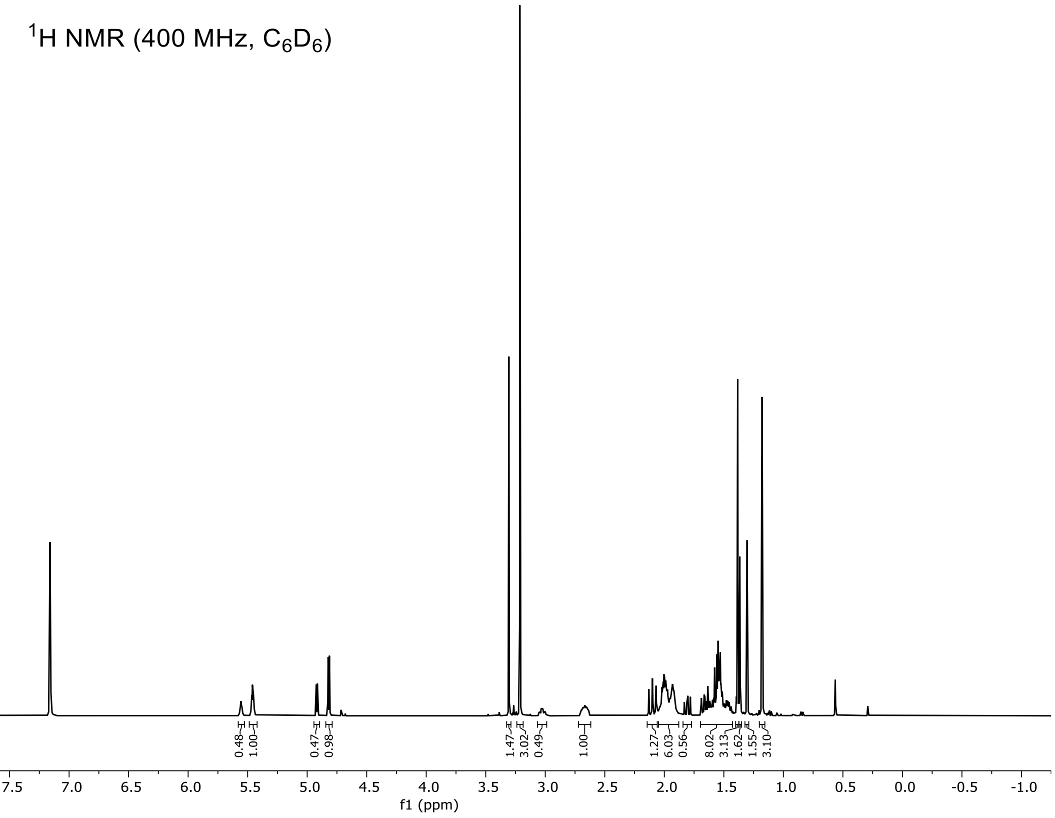

^{1}H NMR (400 MHz, C_6D_6)
0.48 1.00 0.47 0.98 1.47 3.02 0.49 1.00 1.27 6.03 0.56 8.02 3.13 1.62 1.55 3.10
9.0 8.5 8.0 7.5 7.0 6.5 6.0 5.5 5.0 4.5 4.0 3.5 3.0 2.5 2.0 1.5 1.0 0.5 0.0 -0.5 -1.0
f1 (ppm)

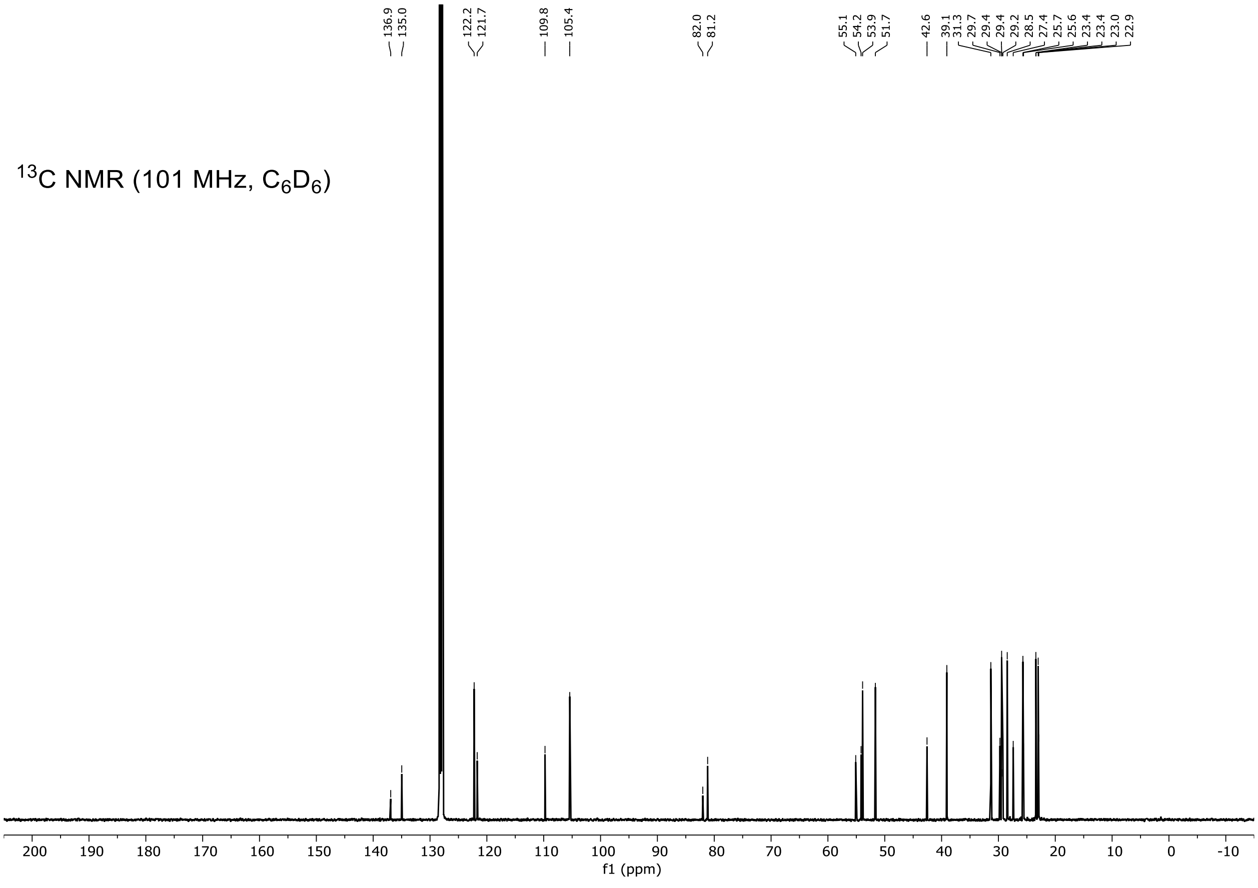

136.9 135.0 122.2 121.7 109.8 105.4 82.0 81.2 55.1 54.2 53.9 51.7 42.6 39.1 31.3 29.7 29.4 29.4 29.2 28.5 27.4 25.7 25.6 23.4 23.4 23.0 22.9
^{13}C NMR (101 MHz, C_6D_6)
200 190 180 170 160 150 140 130 120 110 100 90 80 70 60 50 40 30 20 10 0 -10
f1 (ppm)

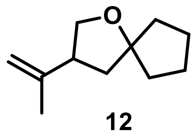

12

^{1}H NMR (400 MHz, $CDCl_3$)

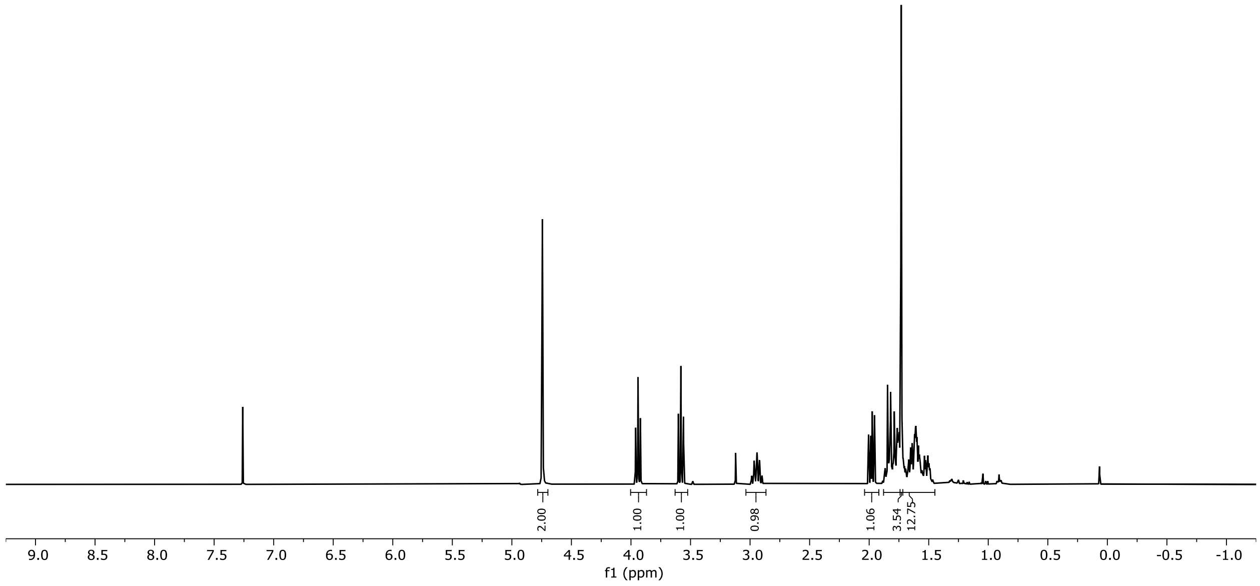

^{13}C NMR (101 MHz, $CDCl_3$)

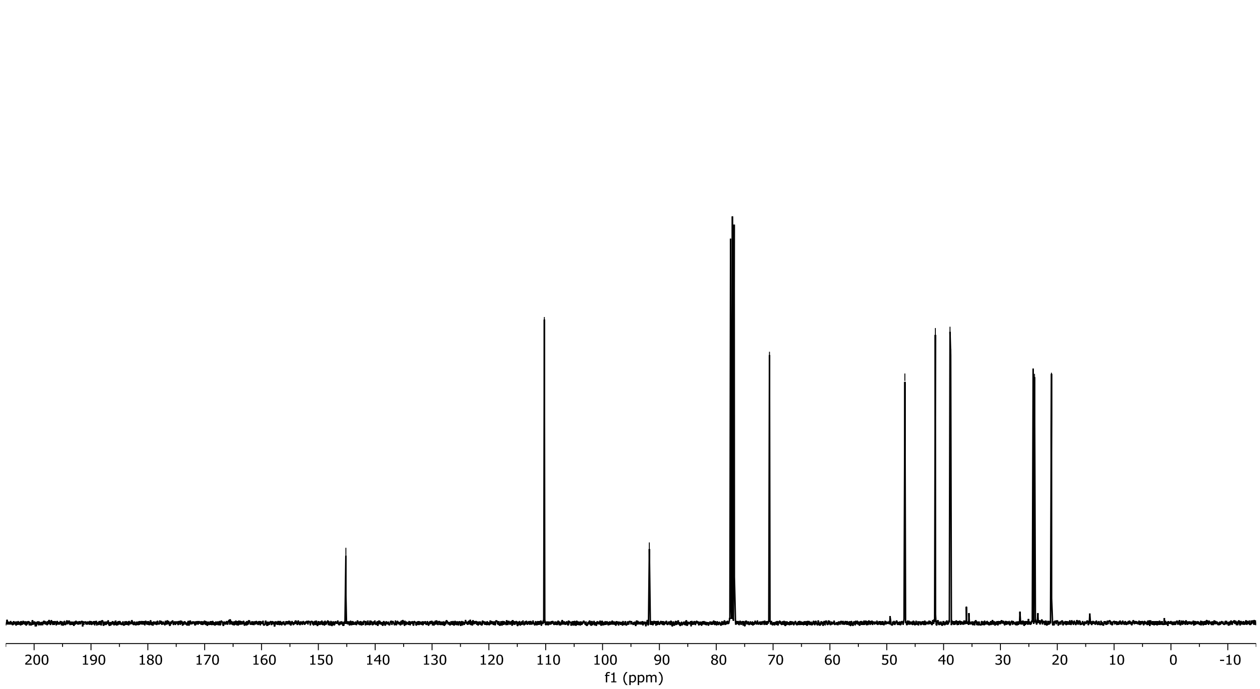

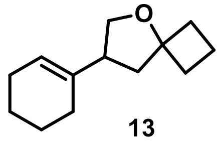

13

^{1}H NMR (400 MHz, $CDCl_3$)

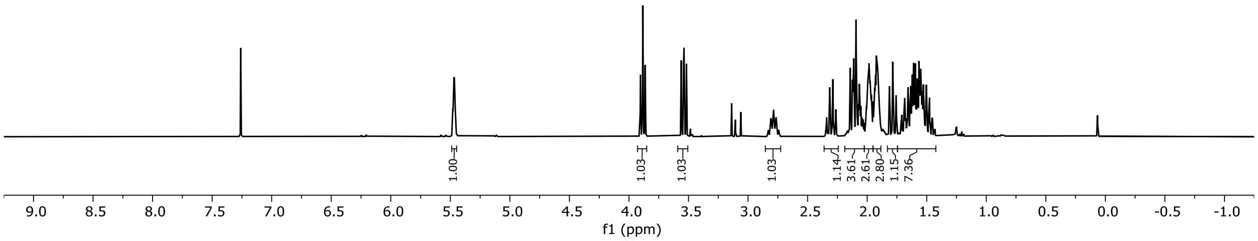

^{13}C NMR (101 MHz, $CDCl_3$)

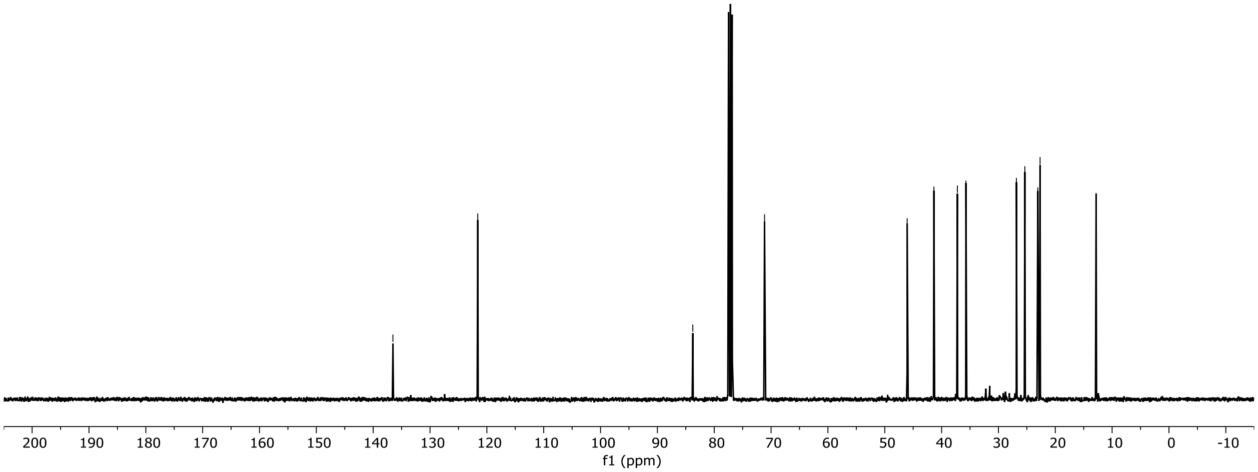

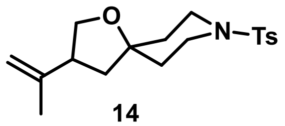

14

^{1}H NMR (400 MHz, $CDCl_3$)

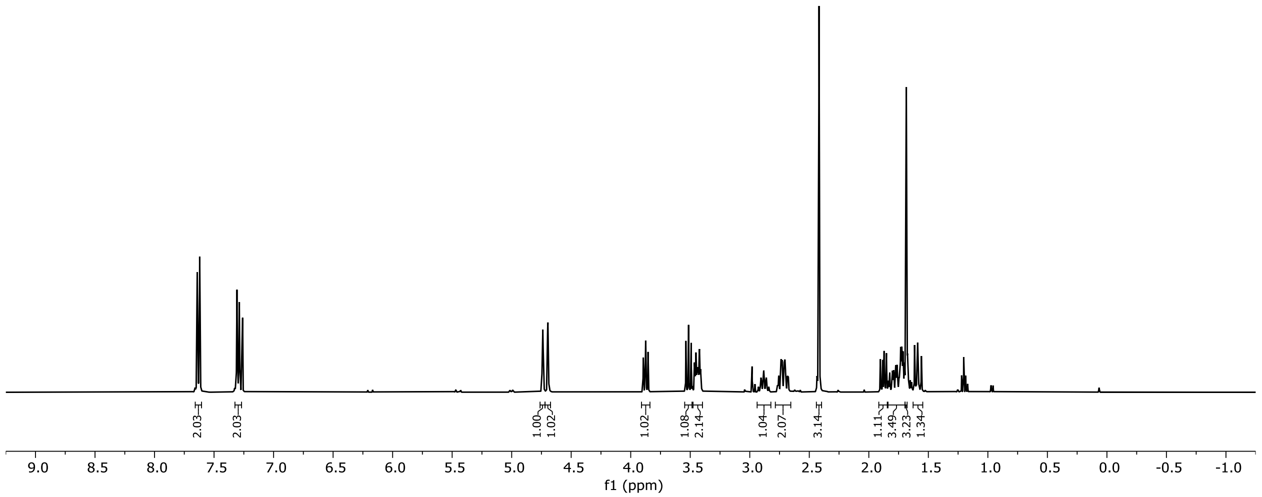

^{13}C NMR (101 MHz, $CDCl_3$)

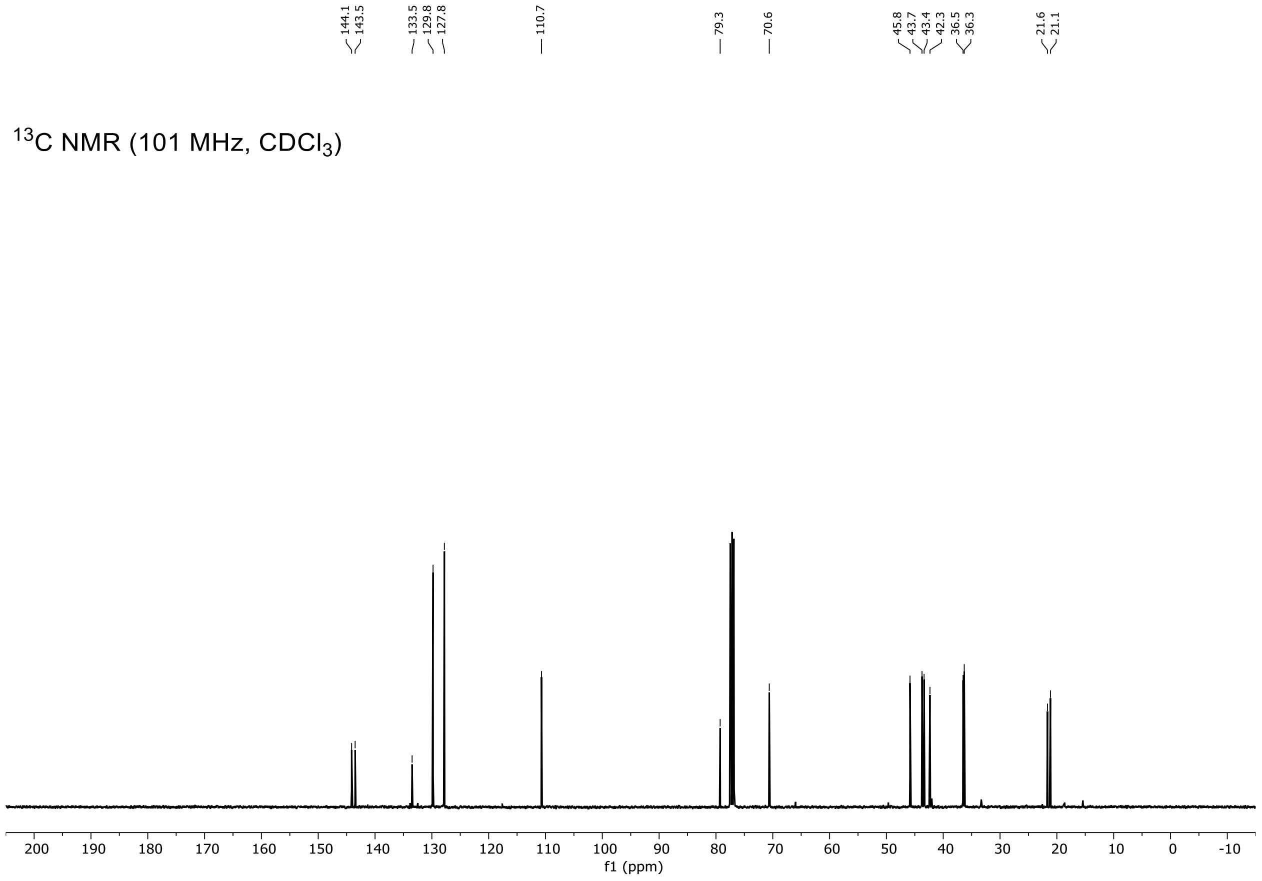

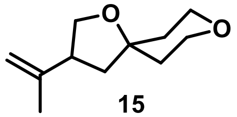

15

^1H NMR (400 MHz, $CDCl_3$)

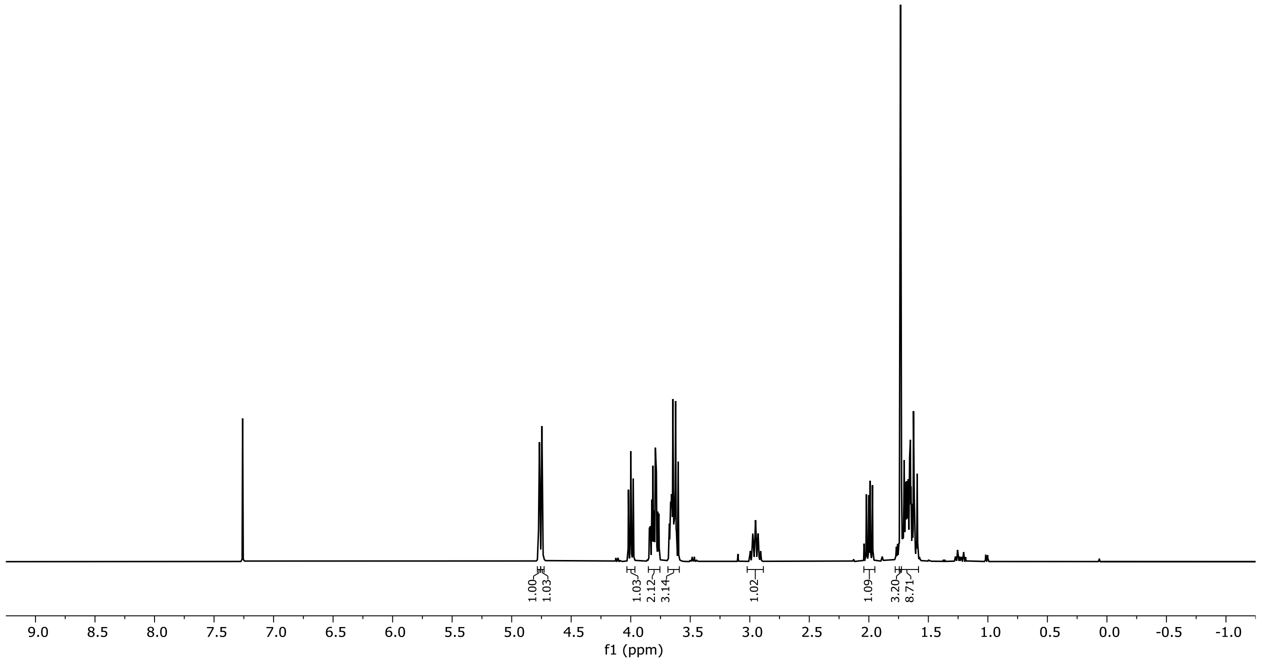

^{13}C NMR (101 MHz, $CDCl_3$)

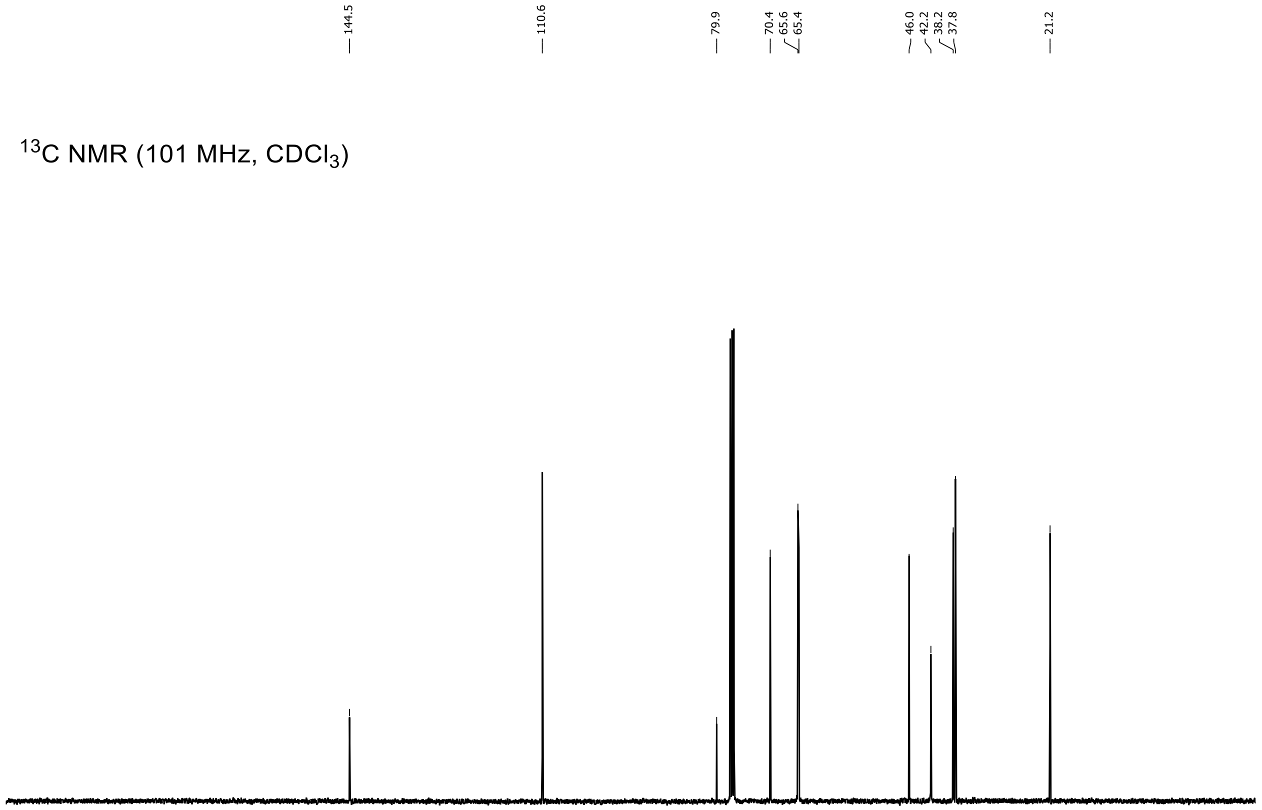

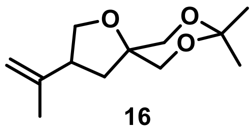

16

^{1}H NMR (400 MHz, $CDCl_3$)

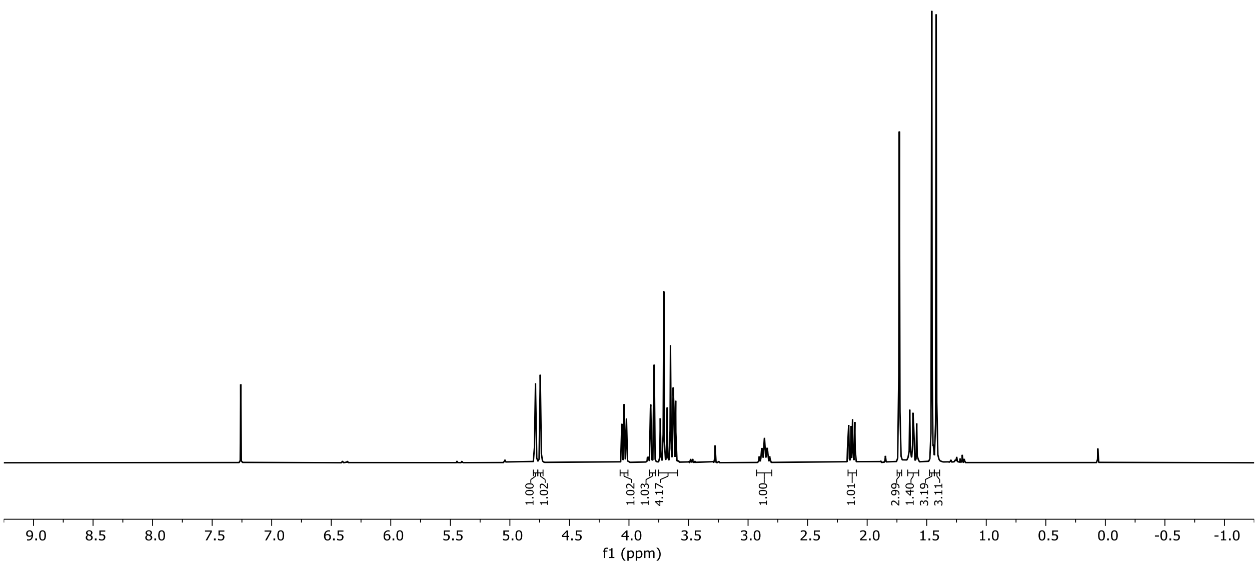

^{13}C NMR (101 MHz, $CDCl_3$)

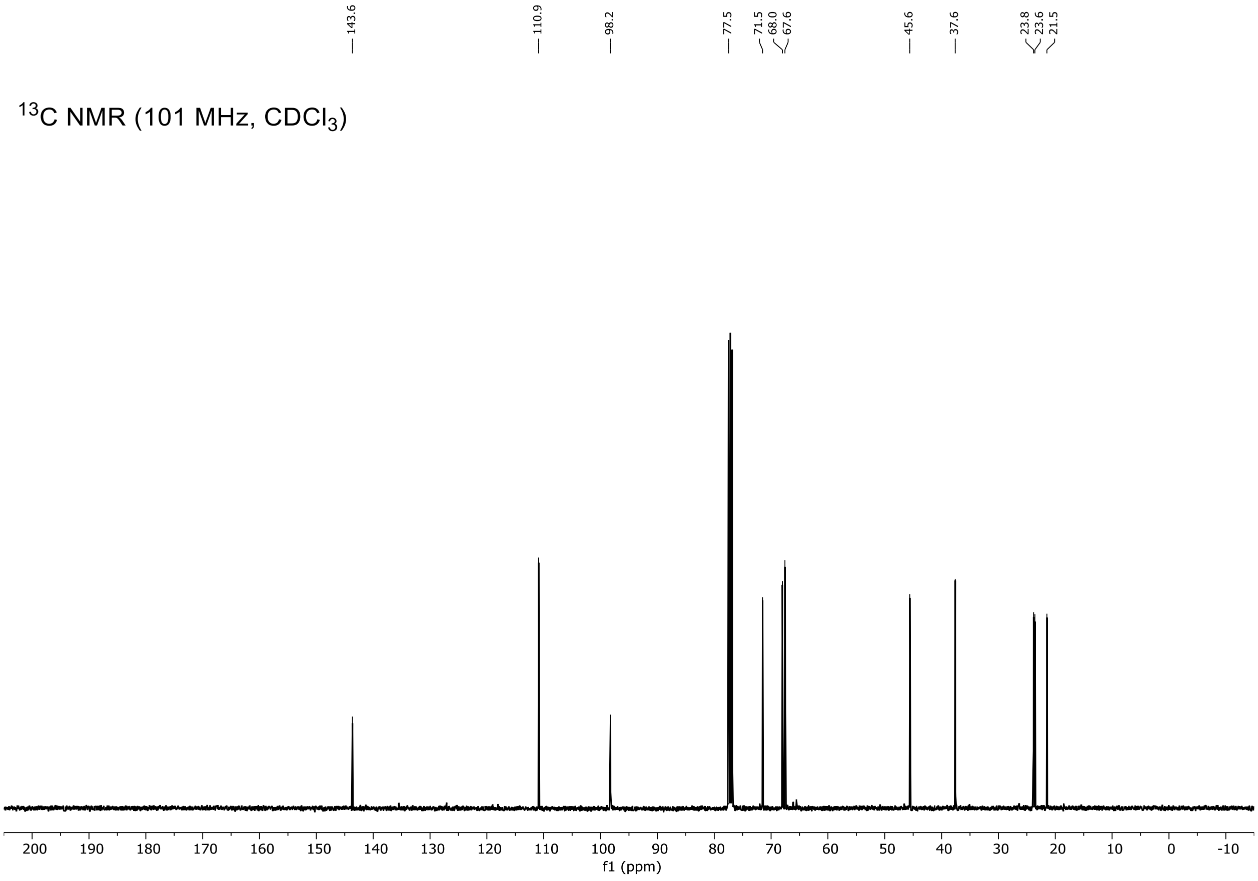

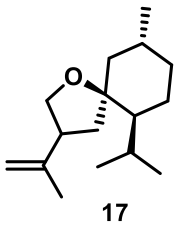

17

^{1}H NMR (400 MHz, $CDCl_3$)

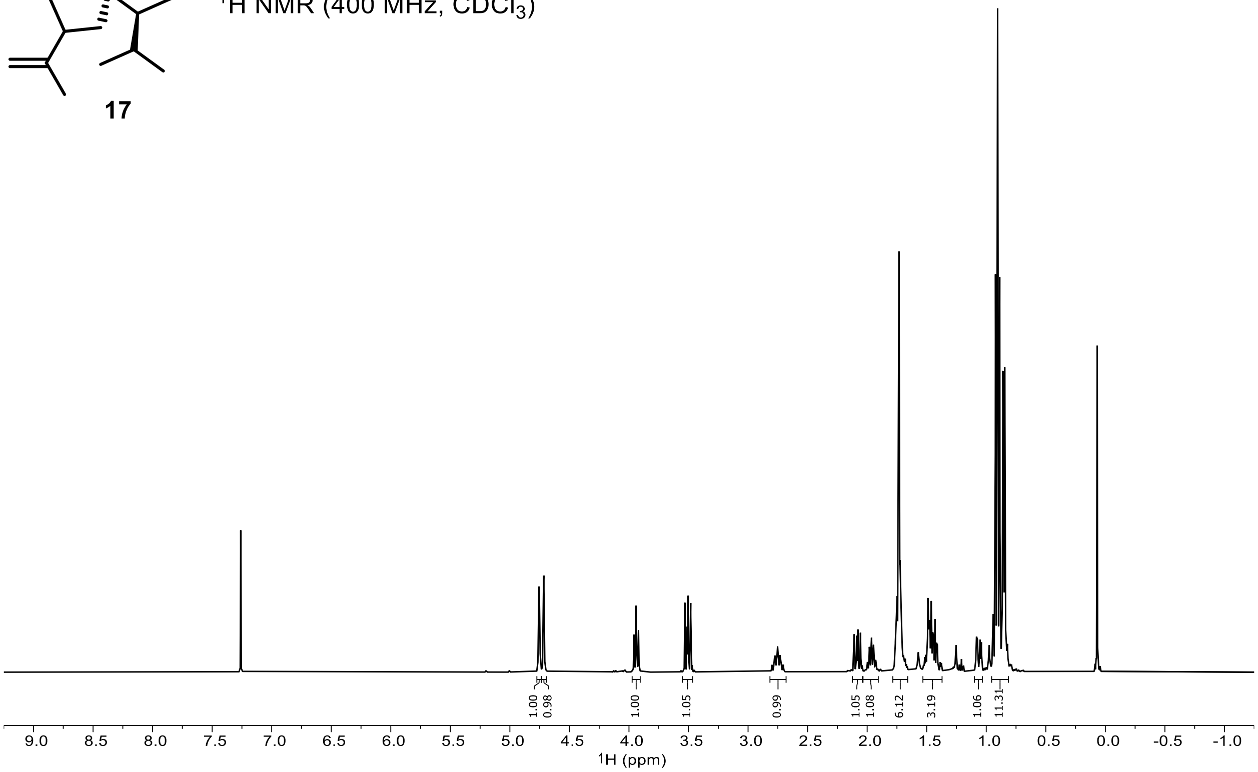

^{13}C NMR (101 MHz, $CDCl_3$)

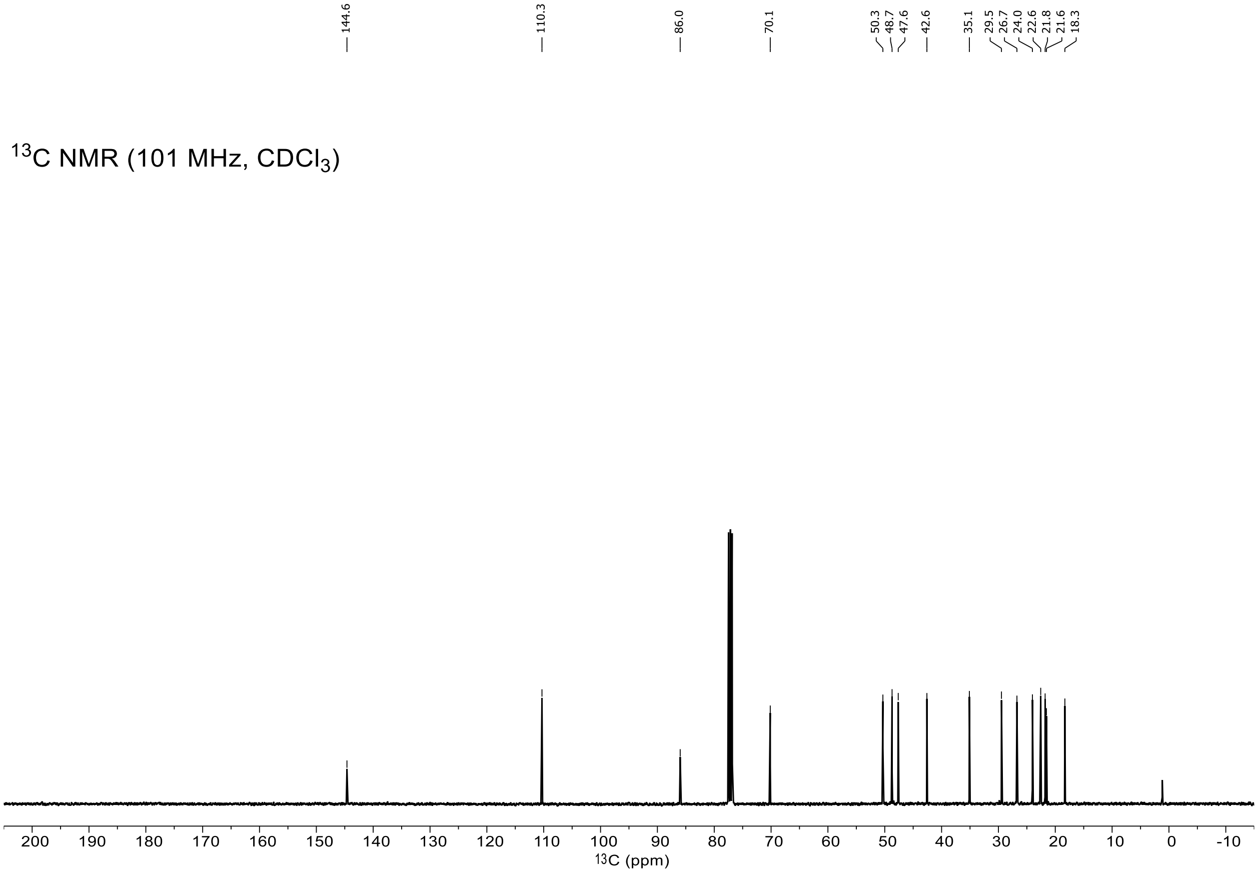

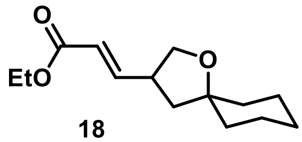

18

^{1}H NMR (400 MHz, $CDCl_3$)

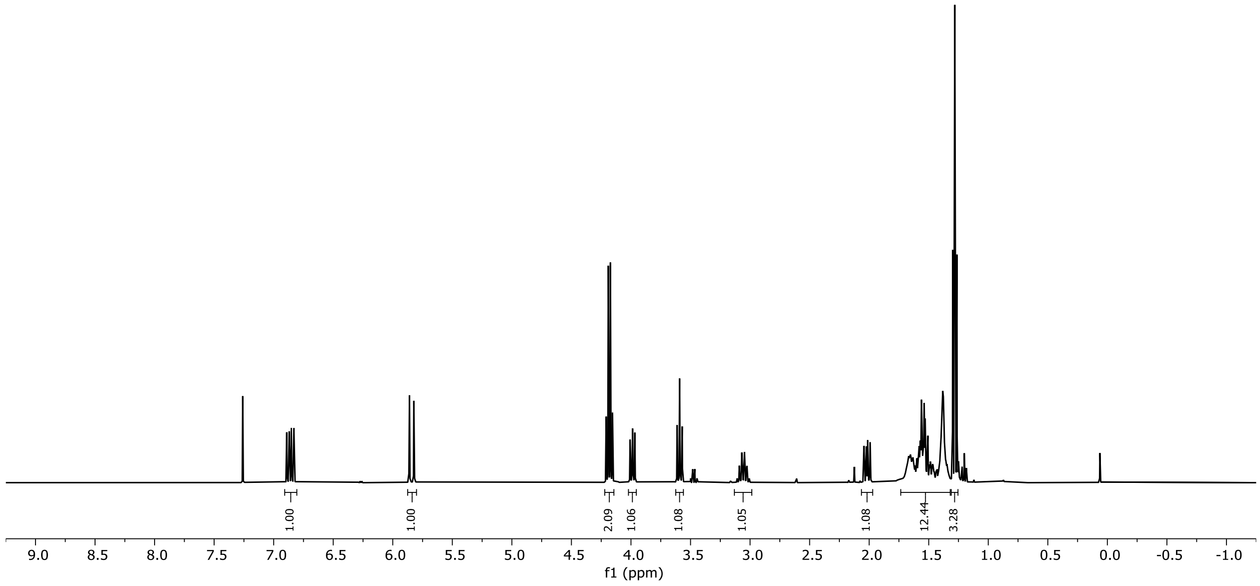

^{13}C NMR (101 MHz, $CDCl_3$)

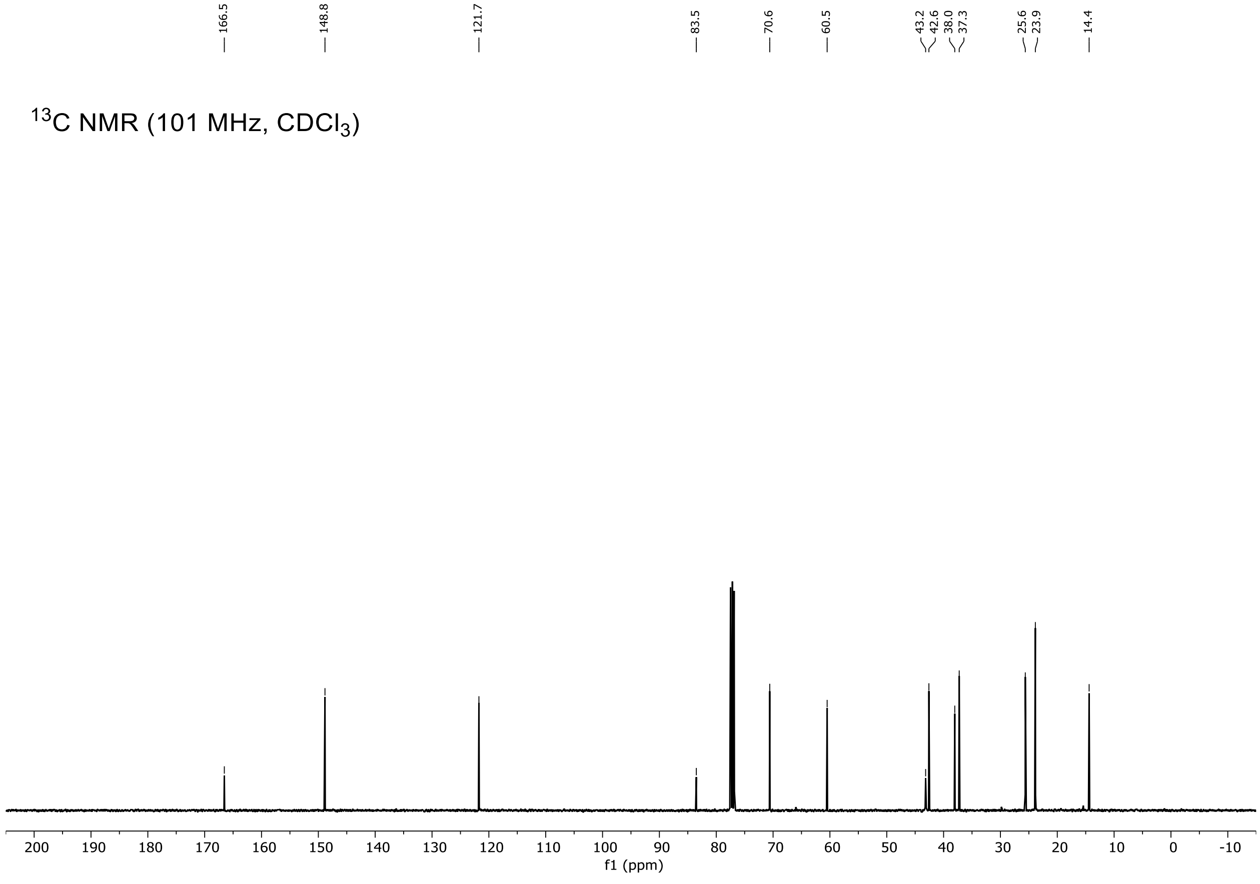

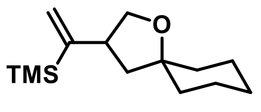

19

^{1}H NMR (400 MHz, $CDCl_3$)

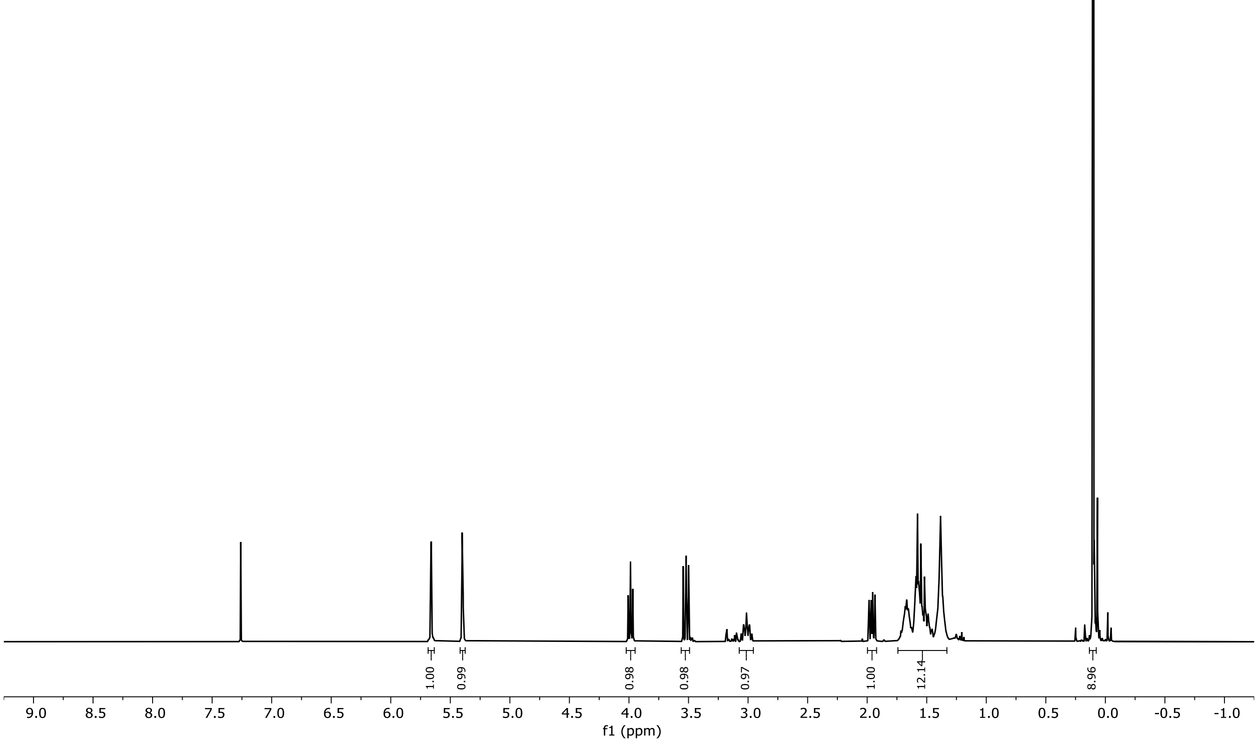

^{13}C NMR (101 MHz, $CDCl_3$)

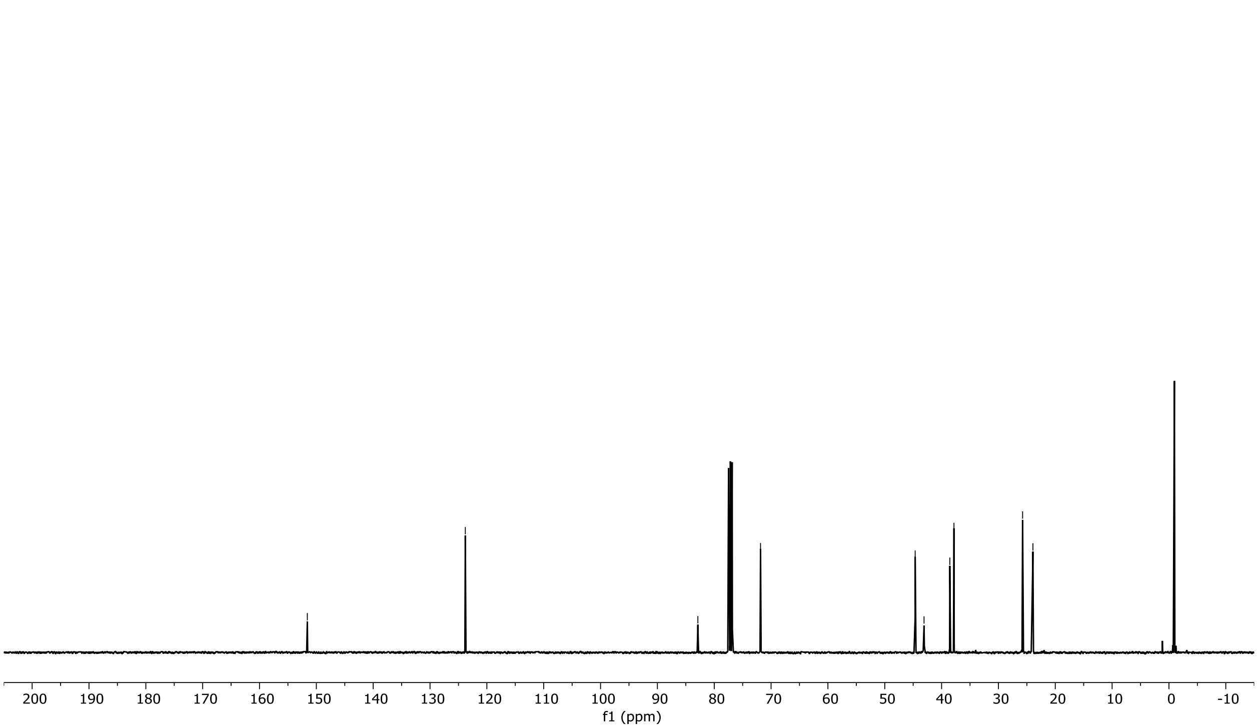

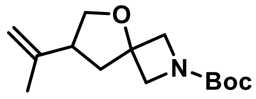

20

^{1}H NMR (400 MHz, $CDCl_3$)

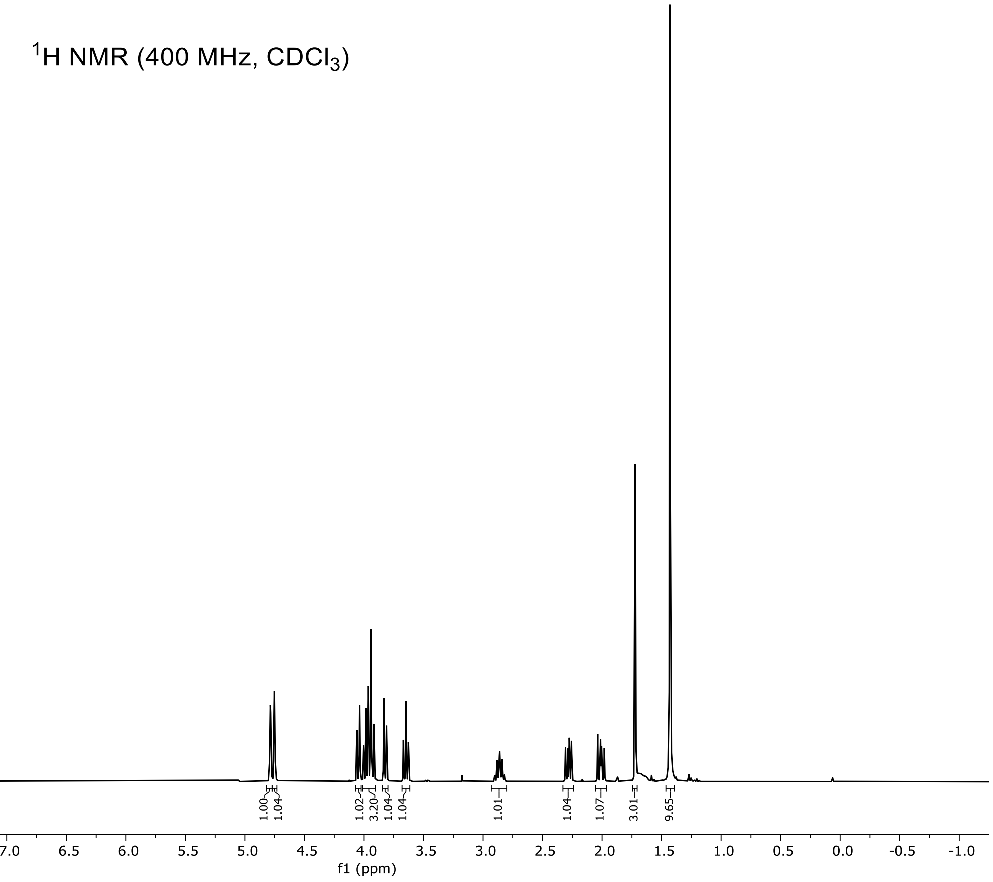

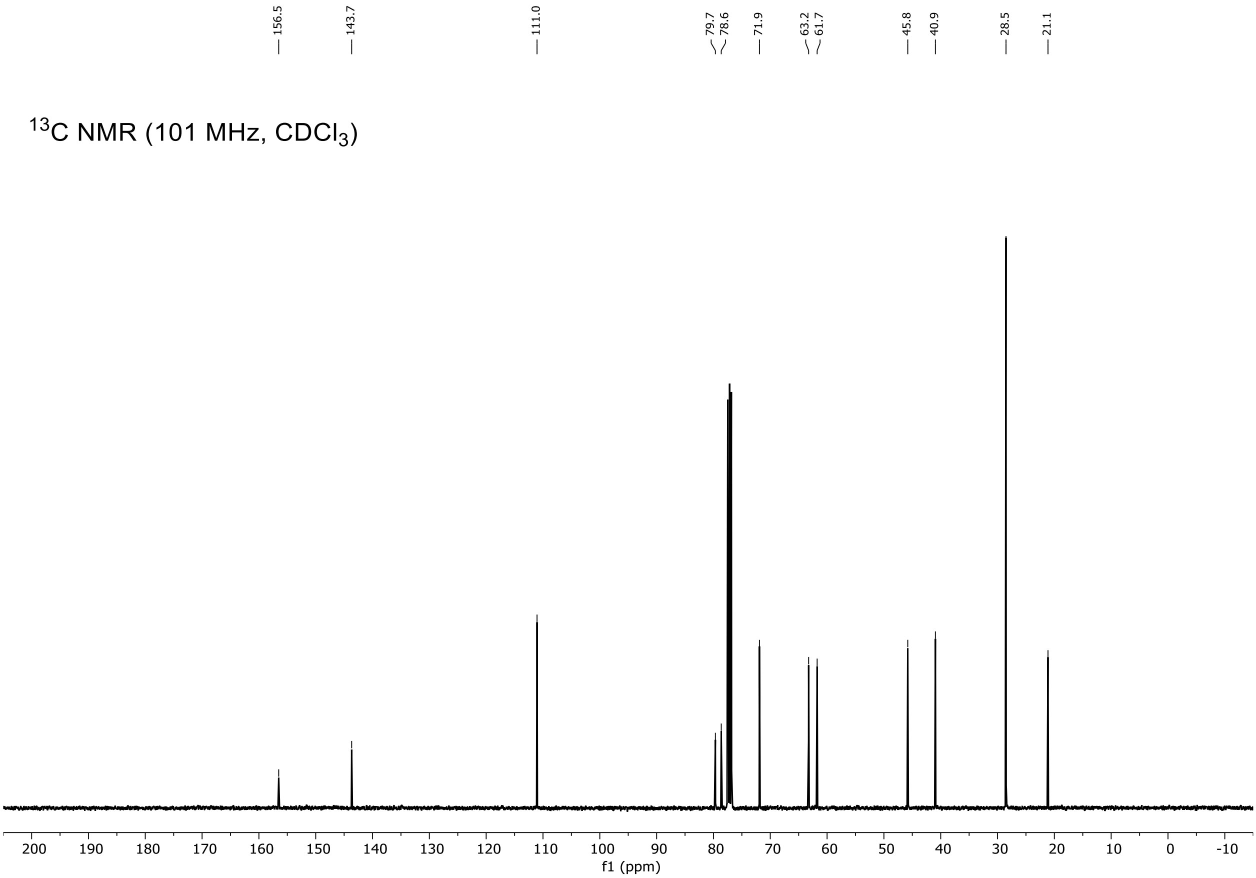

^{13}C NMR (101 MHz, $CDCl_3$)

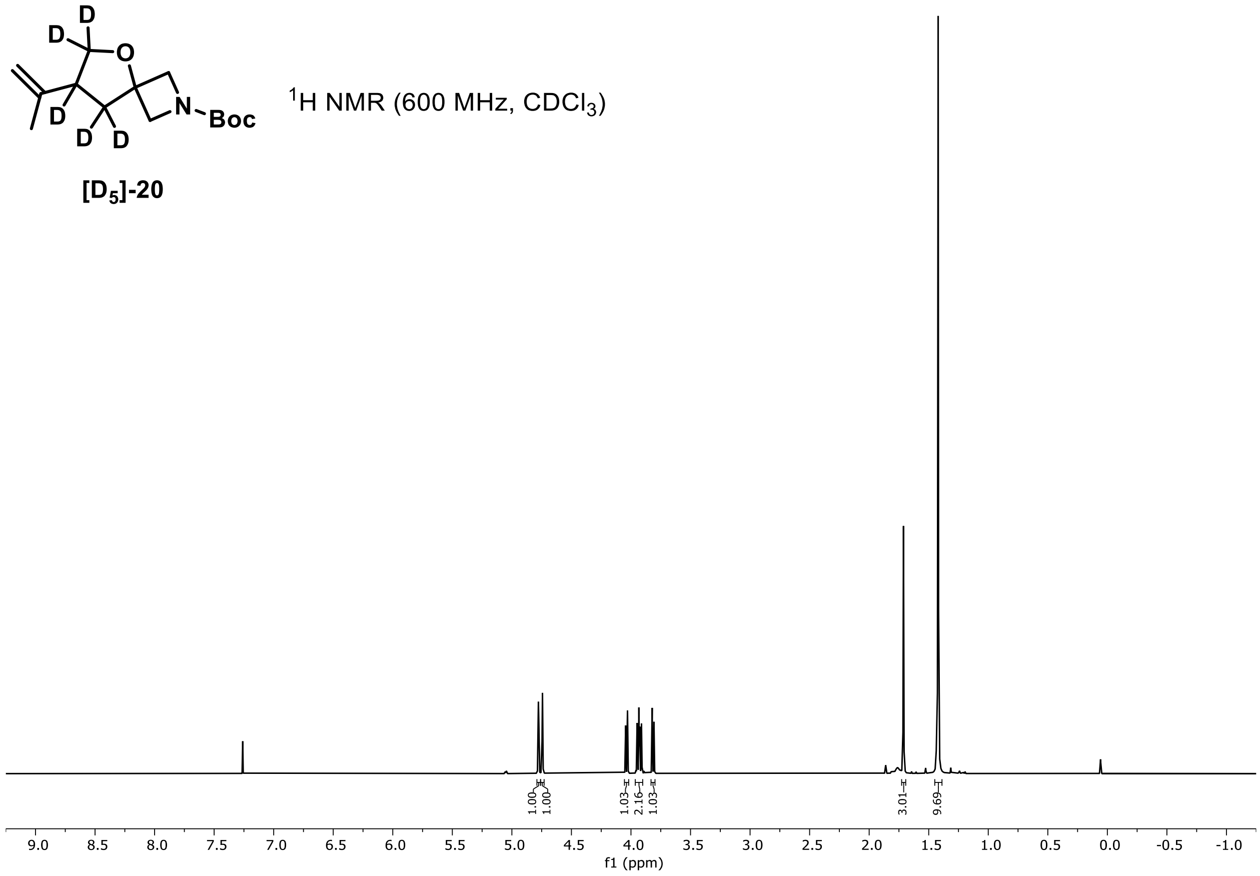
D
D
O
N
Boc
D
D
D
[D$_5$]-20
^{1}H NMR (600 MHz, $CDCl_3$)
1.00
1.00
1.03
2.16
1.03
3.01
9.69
9.0 8.5 8.0 7.5 7.0 6.5 6.0 5.5 5.0 4.5 4.0 3.5 3.0 2.5 2.0 1.5 1.0 0.5 0.0 -0.5 -1.0
f1 (ppm)

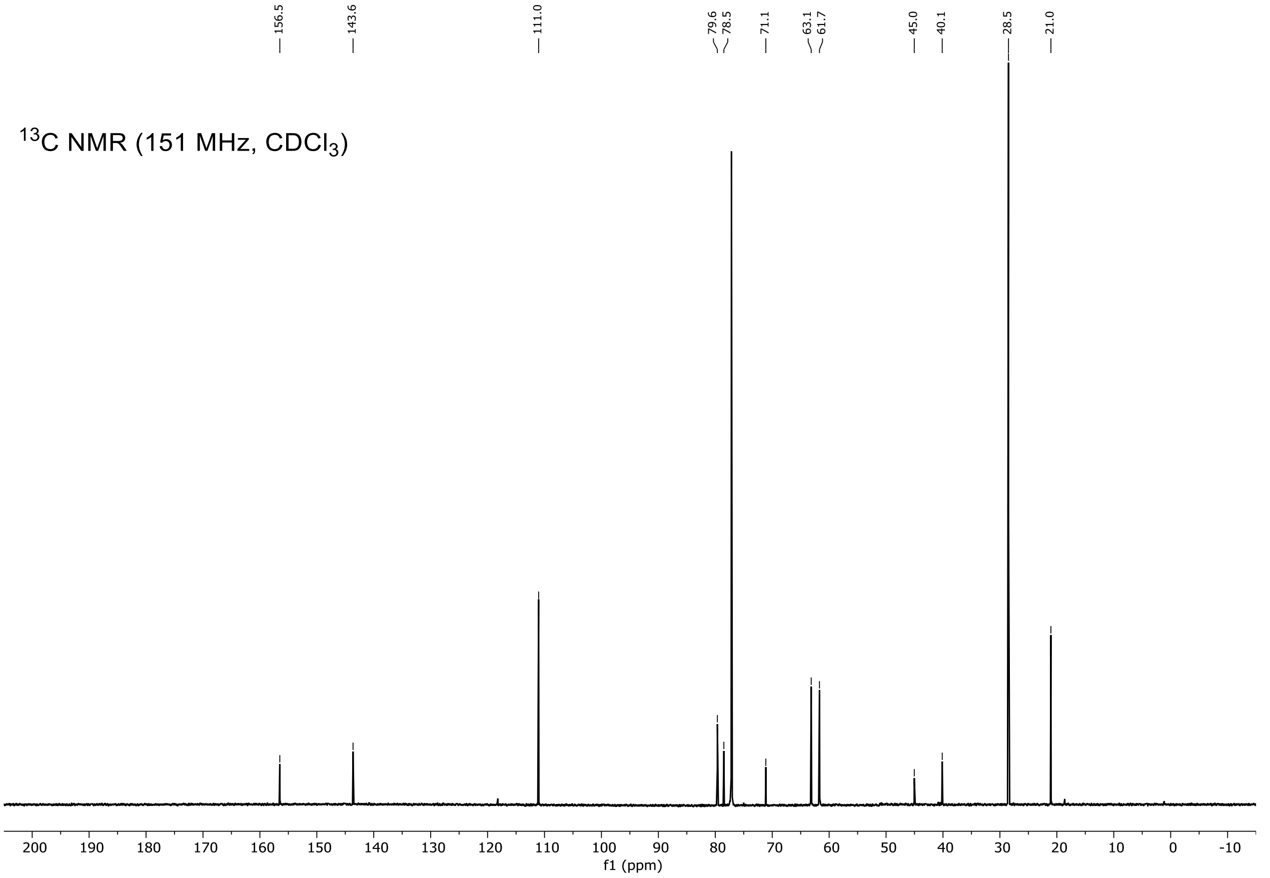
156.5
143.6
111.0
79.6
78.5
71.1
63.1
61.7
45.0
40.1
28.5
21.0
^{13}C NMR (151 MHz, $CDCl_3$)
200 190 180 170 160 150 140 130 120 110 100 90 80 70 60 50 40 30 20 10 0 -10
f1 (ppm)

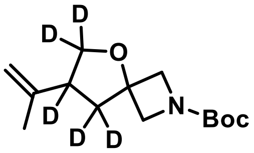

[D$_5$]-20

^{2}H NMR (92 MHz, $CDCl_3$)

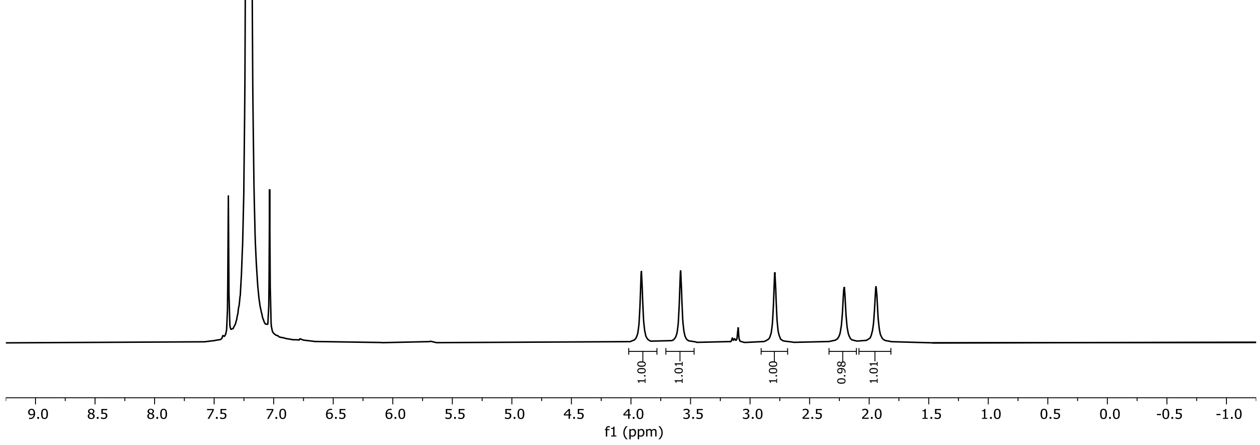

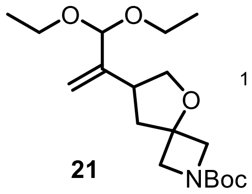

21

^1H NMR (400 MHz, C_6D_6)

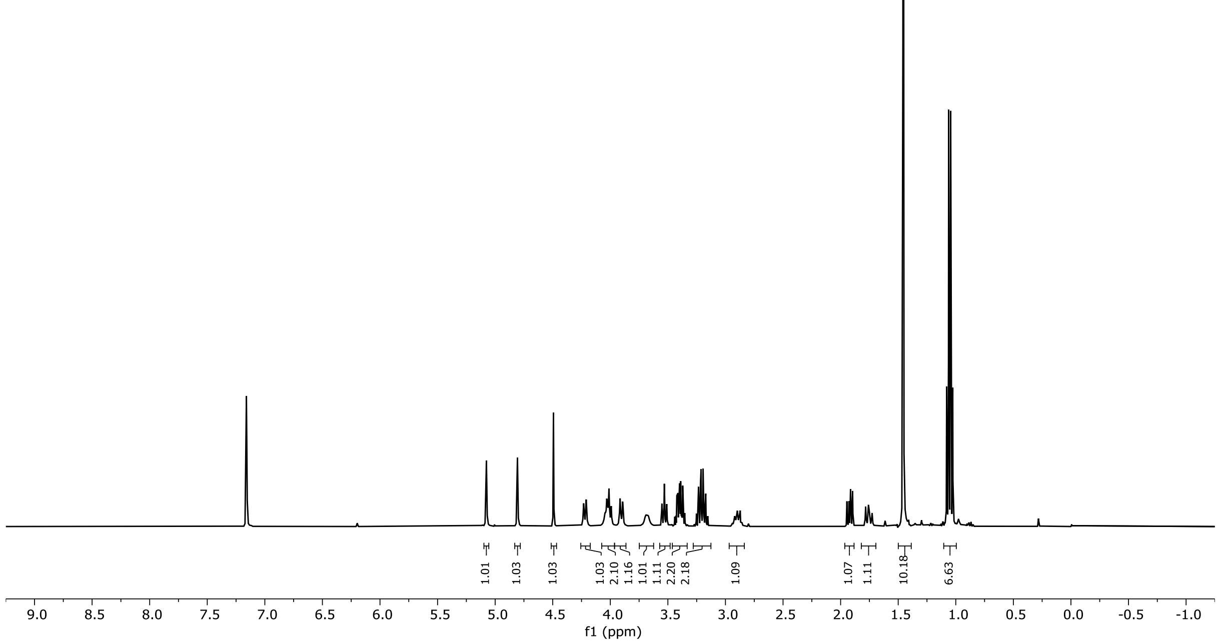

^{13}C NMR (101 MHz, C_6D_6)

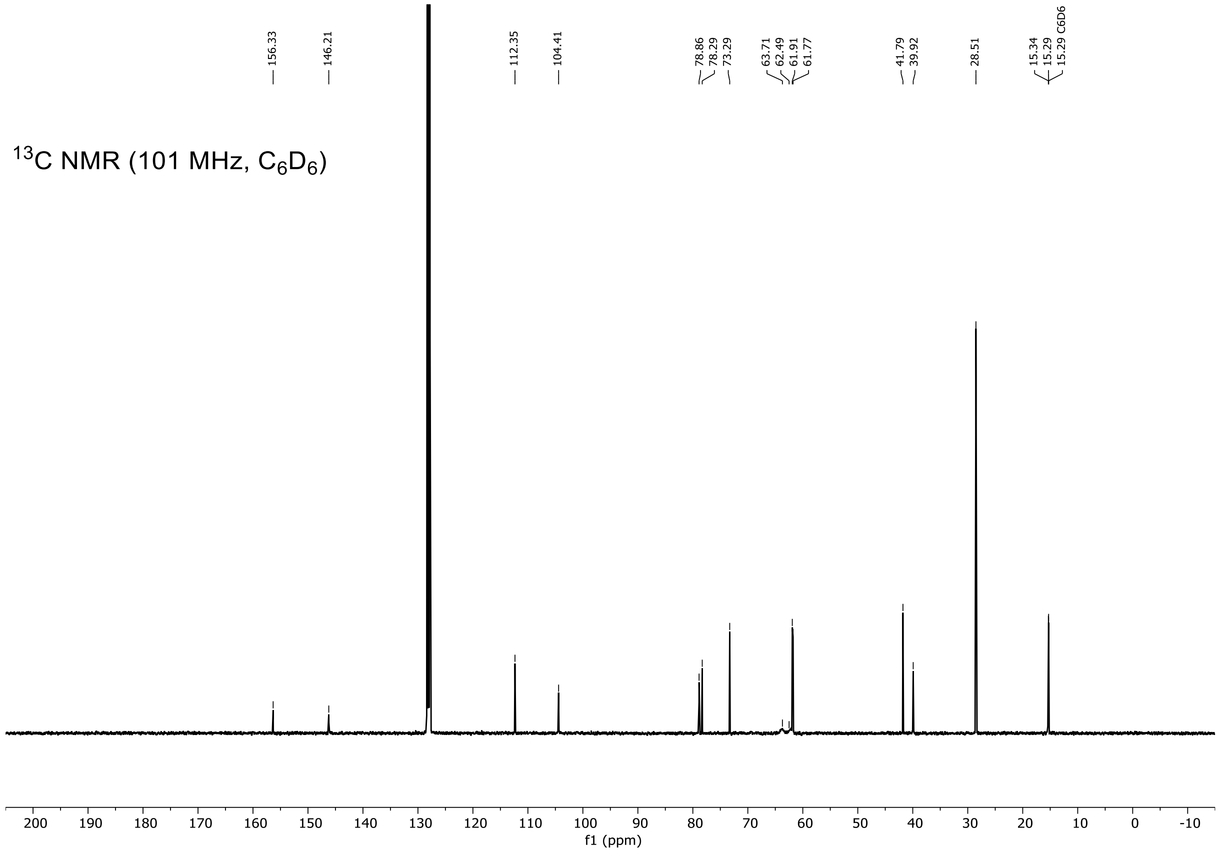

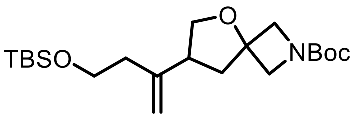

22

^{1}H NMR (400 MHz, $CDCl_3$)

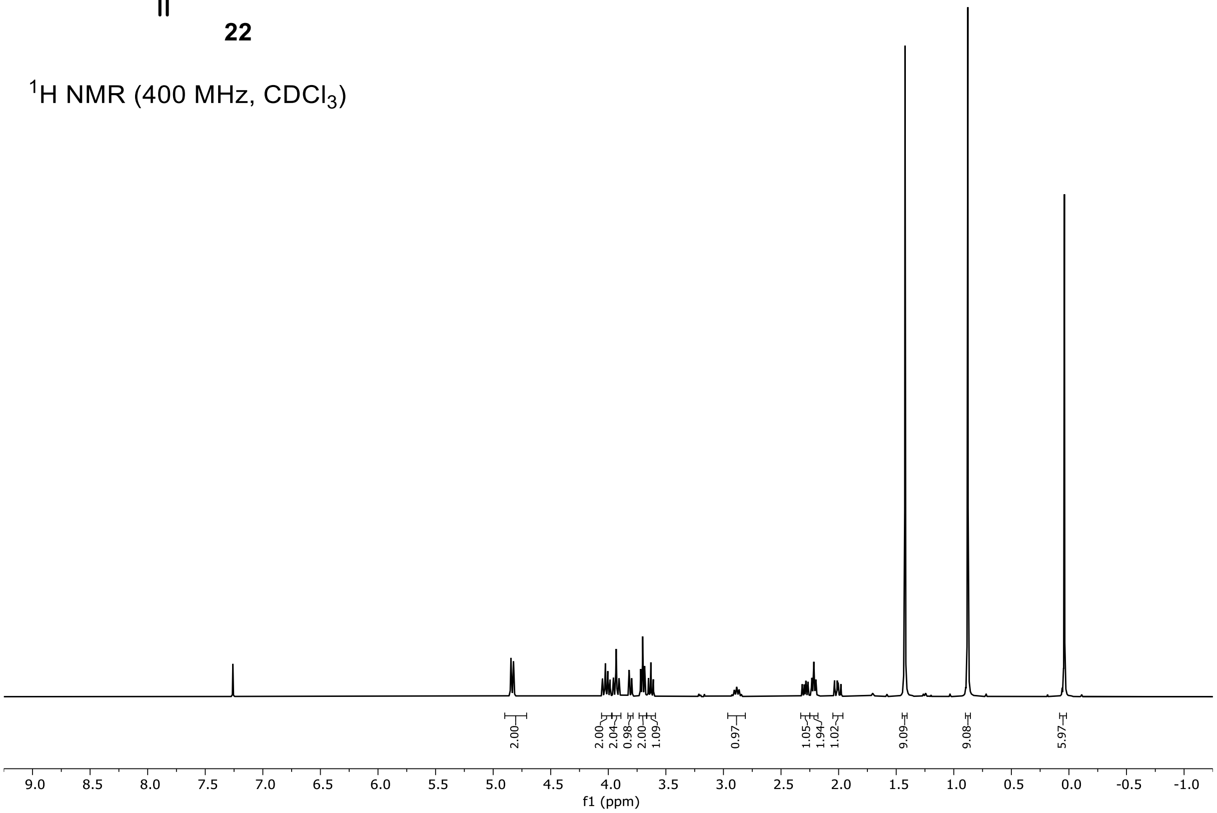

^{13}C NMR (101 MHz, $CDCl_3$)

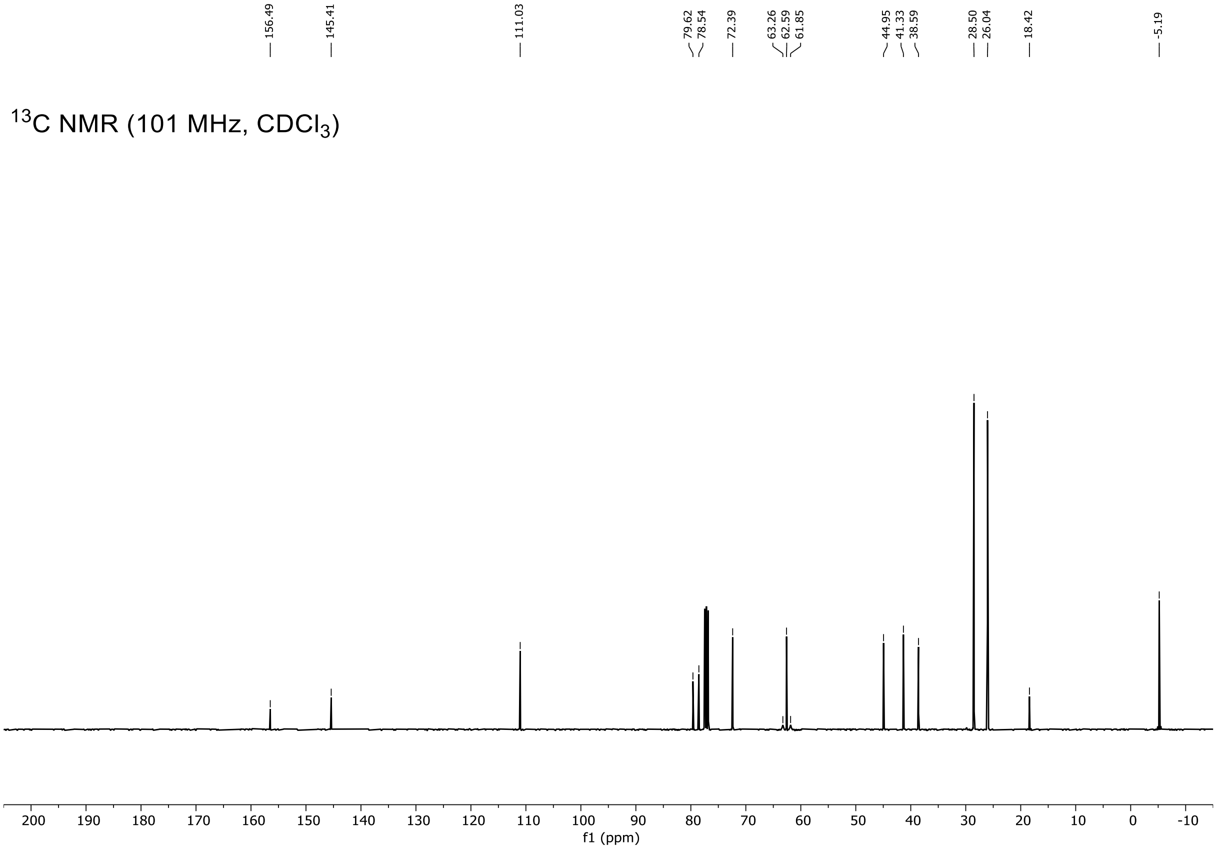

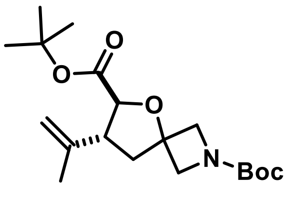

23 (dr.: 3:1)

^{1}H NMR (400 MHz, $CDCl_3$)

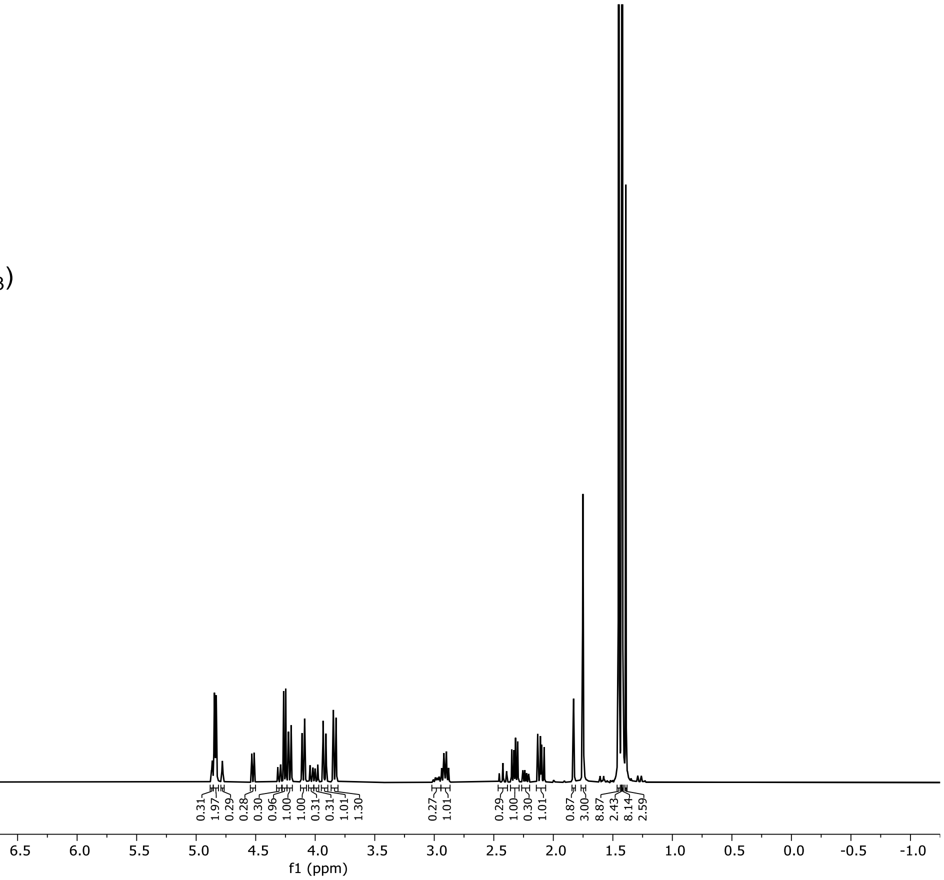

^{13}C NMR (101 MHz, $CDCl_3$)

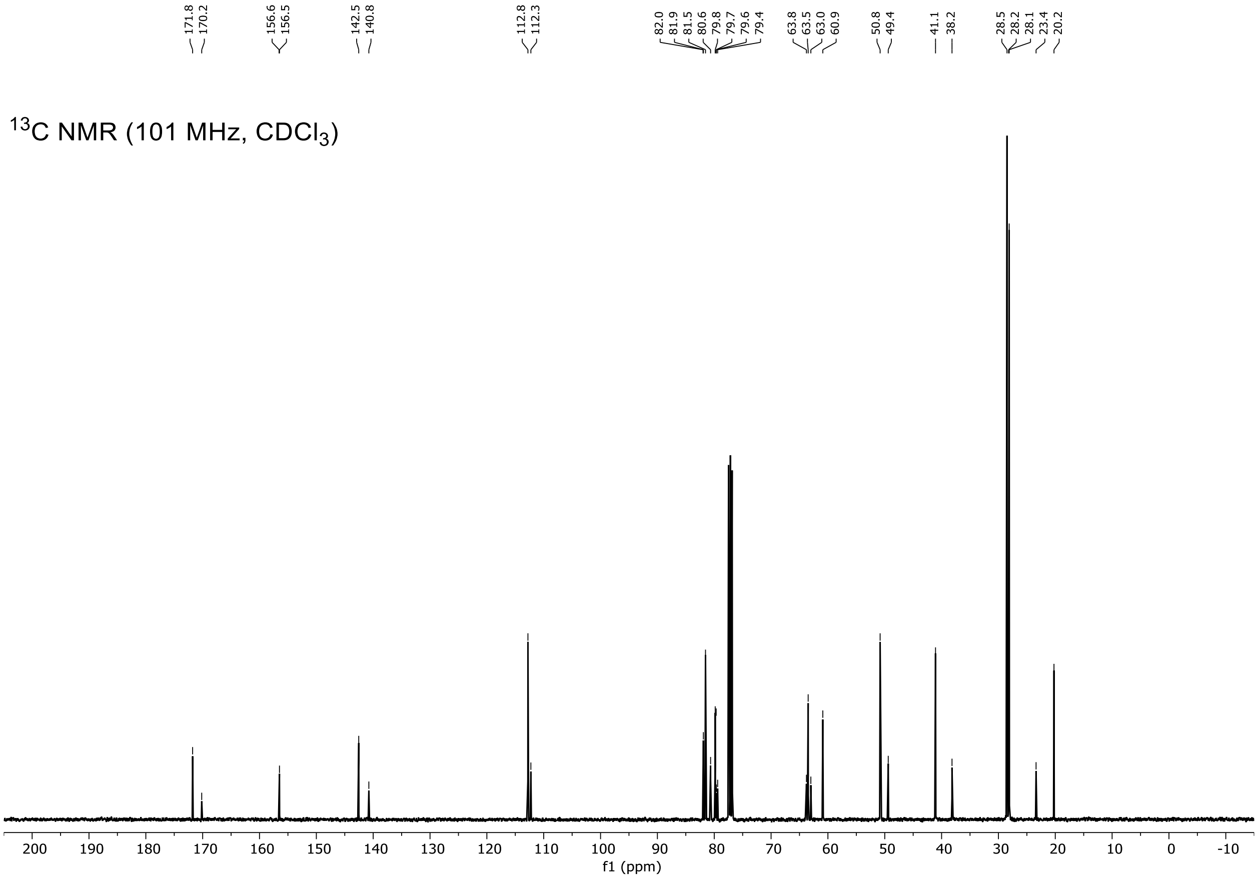

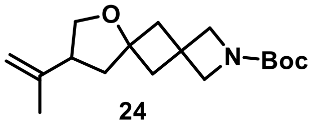

24

^{1}H NMR (400 MHz, $CDCl_3$)

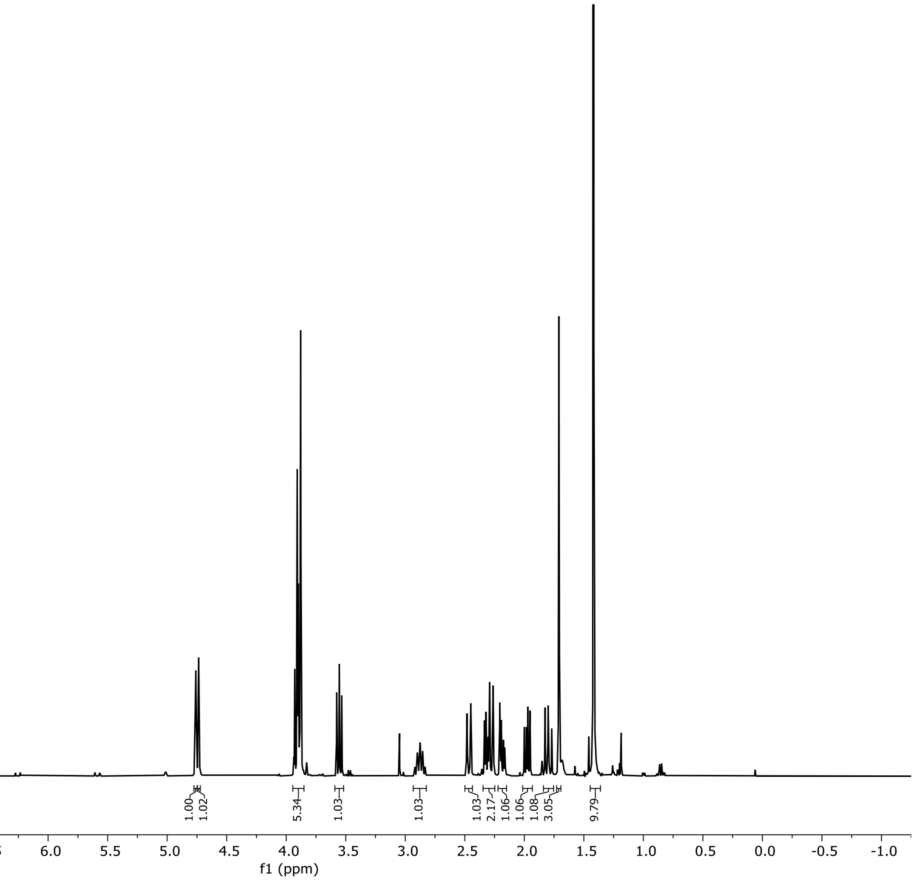

^{13}C NMR (101 MHz, $CDCl_3$)

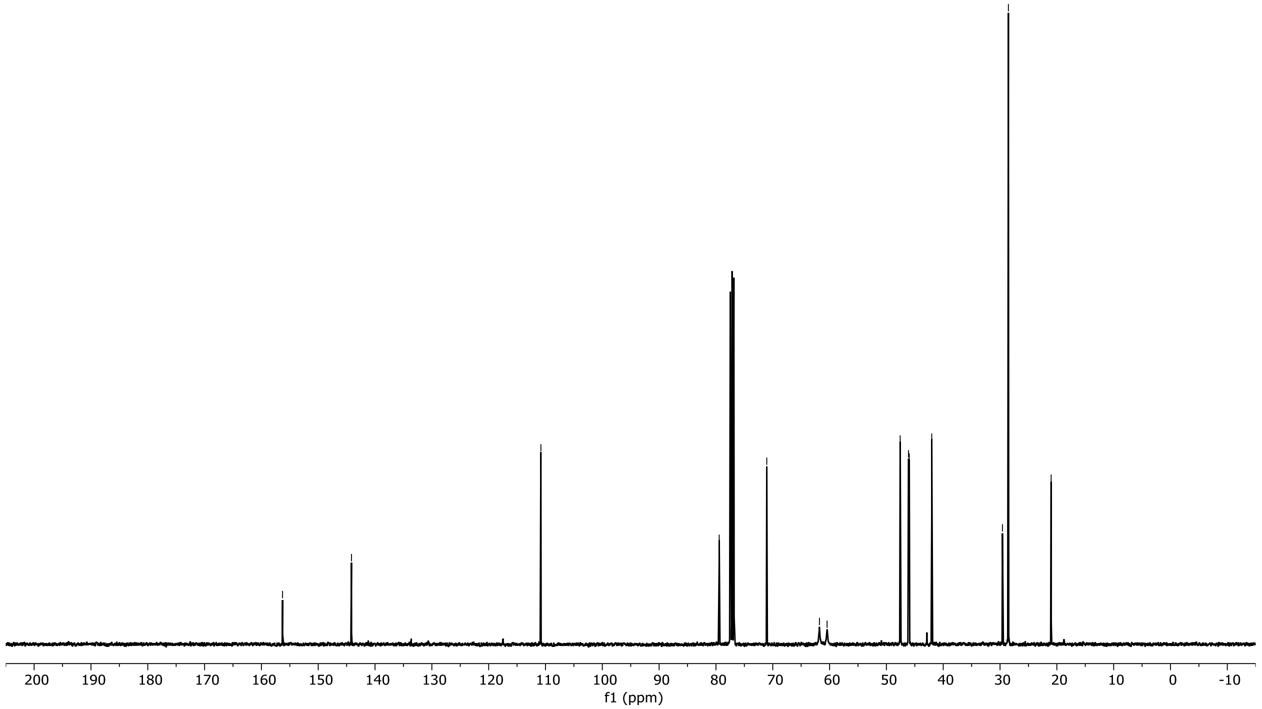

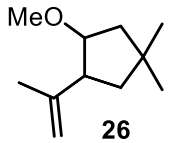

26

^{1}H NMR (400 MHz, $CDCl_3$)

jr27016-SPG-SB-003-01.10.fid
SPG-SB-003-01
12 mg

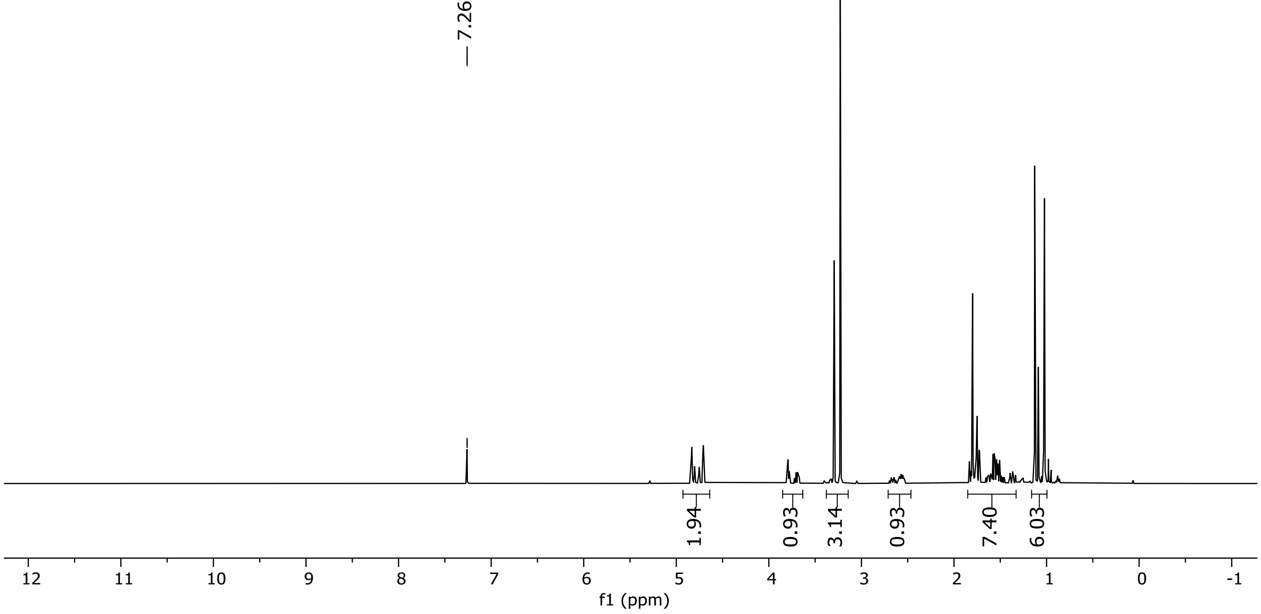

^{13}C NMR (101 MHz, $CDCl_3$)

jr27011-SPG-SB-003-01.13.fid
SPG-SB-003-01

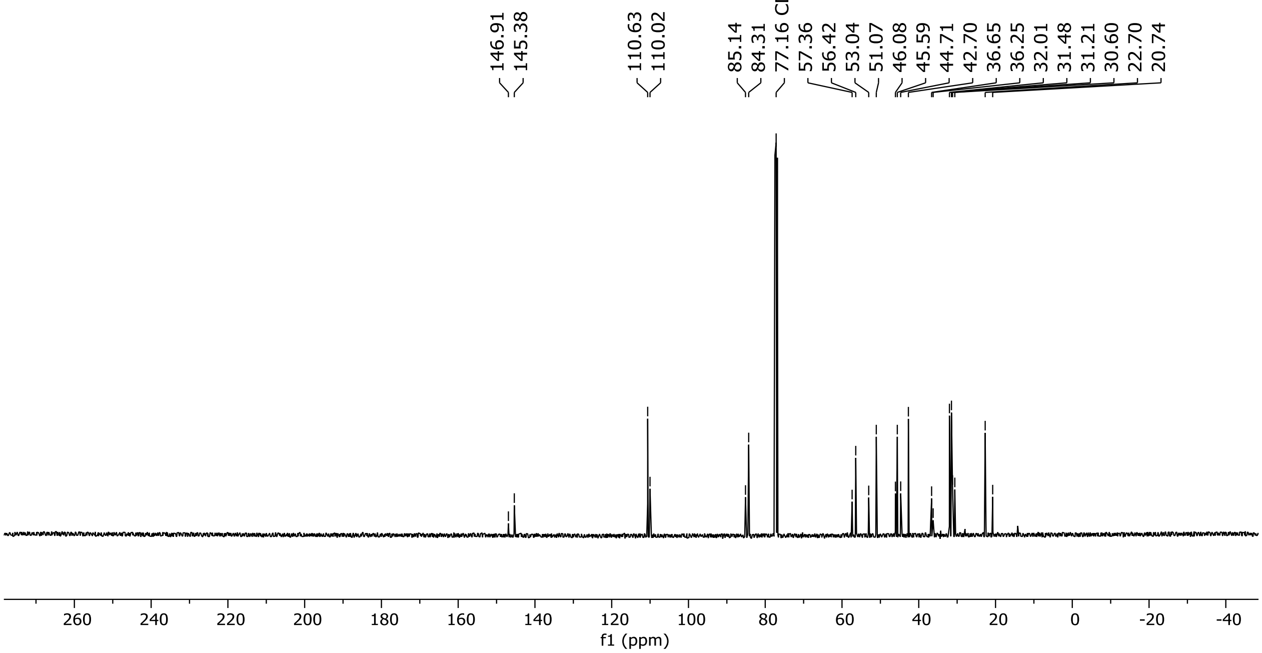

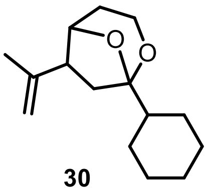

30

^{1}H NMR (400 MHz, C_6D_6)

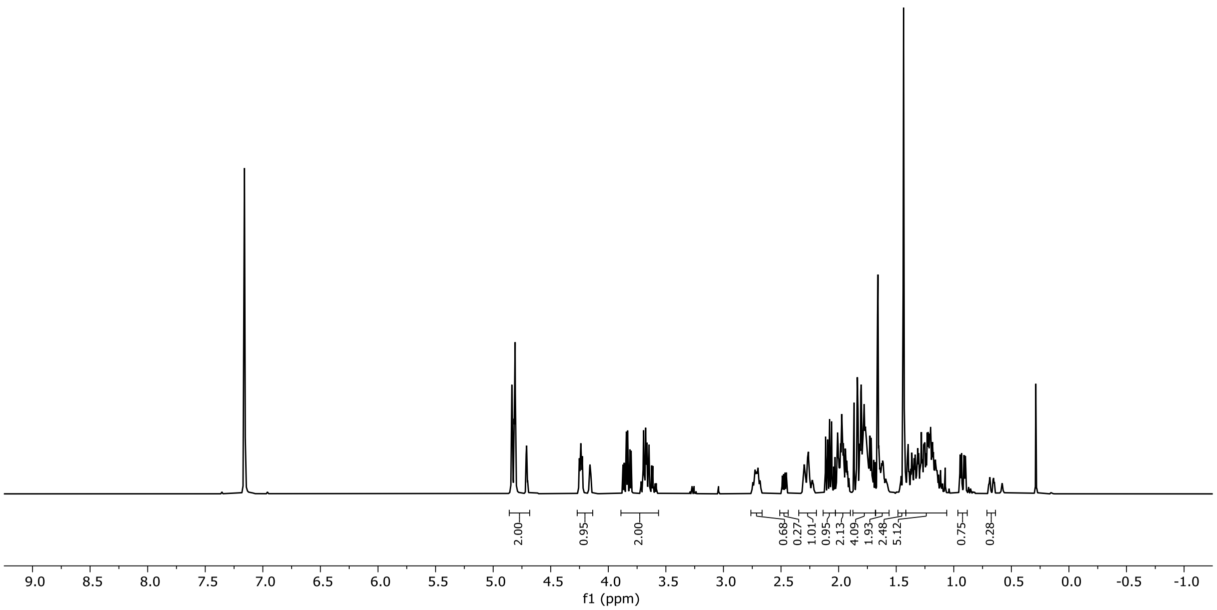

^{13}C NMR (101 MHz, C_6D_6)

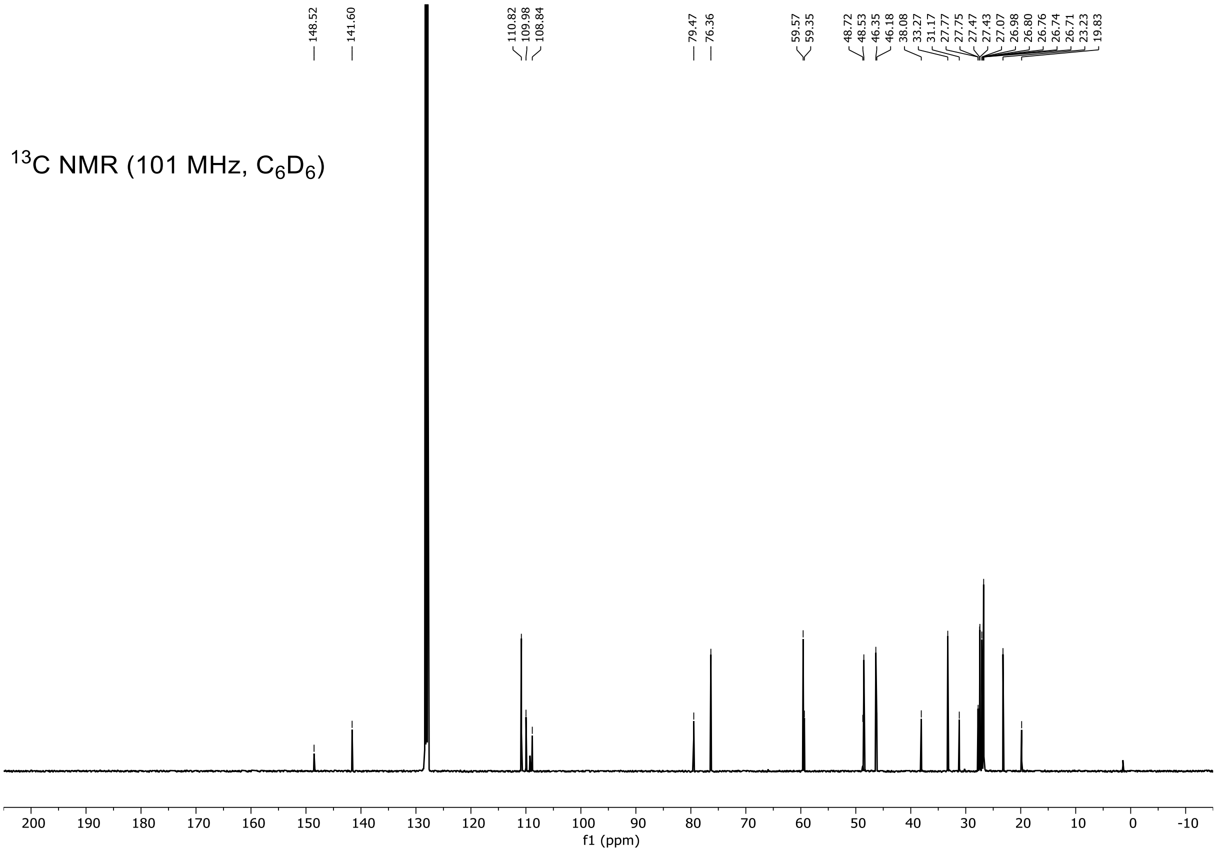

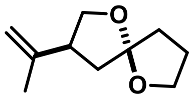

31a

^{1}H NMR (400 MHz, C_6D_6)

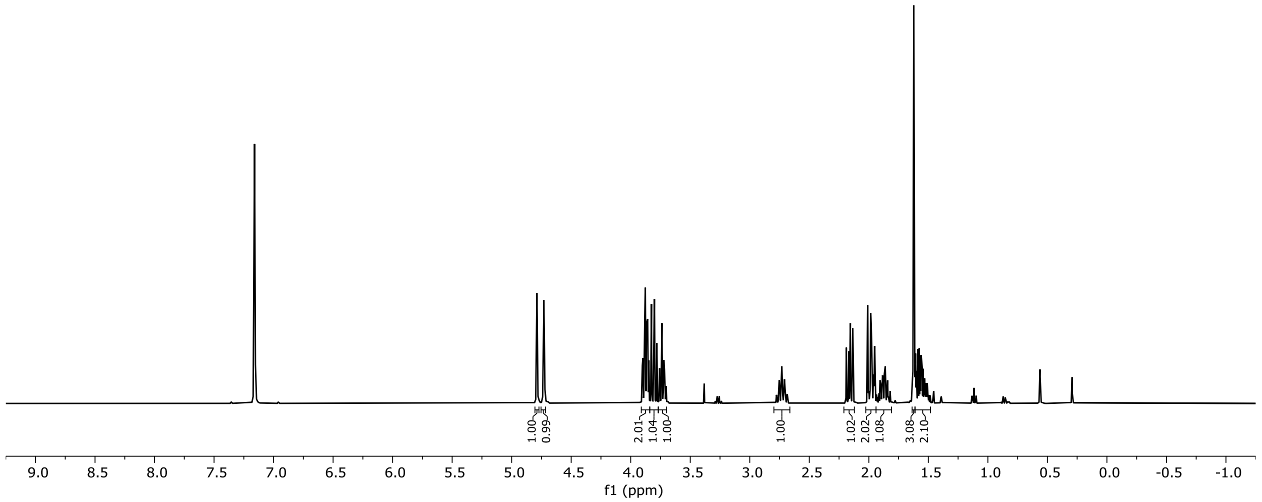

^{13}C NMR (101 MHz, C_6D_6)

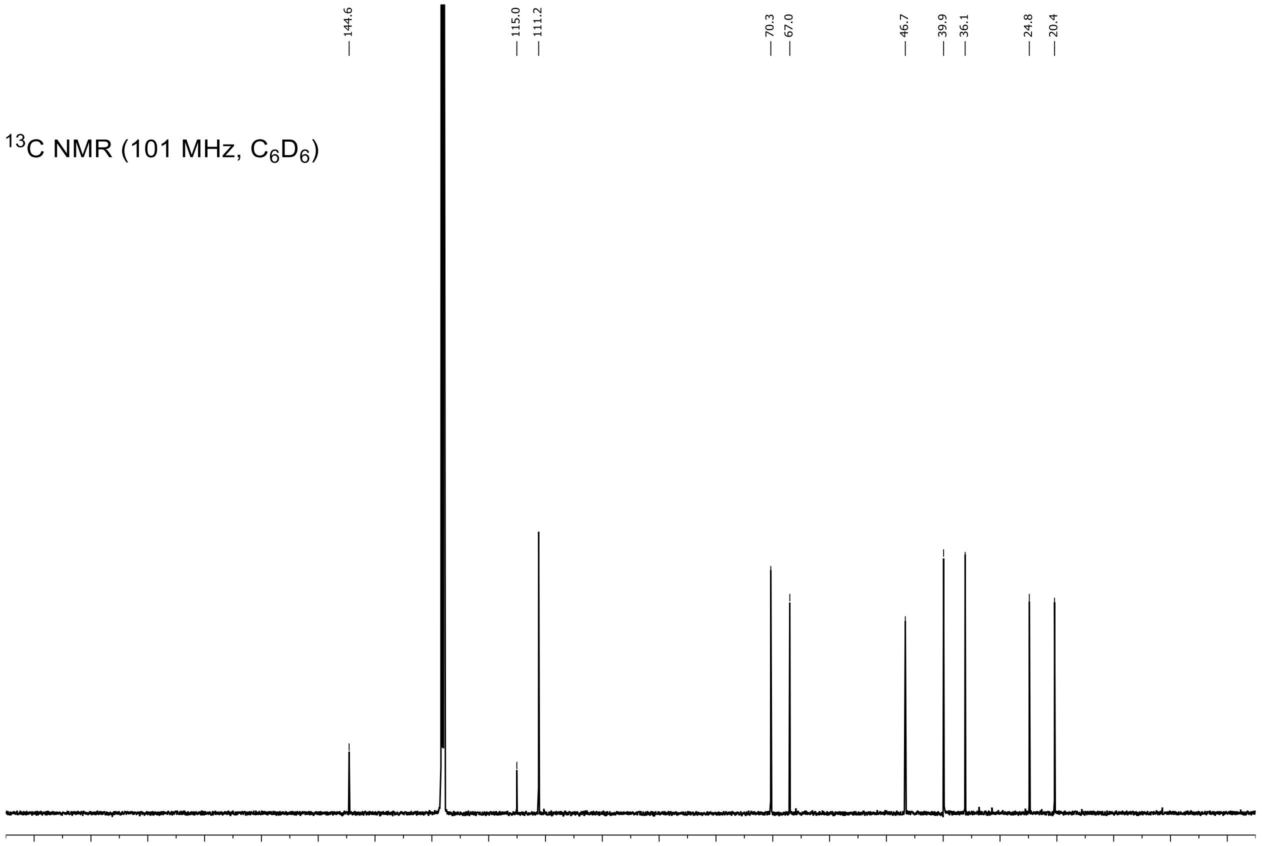

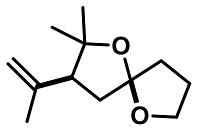

31b (dr.: 3:1)

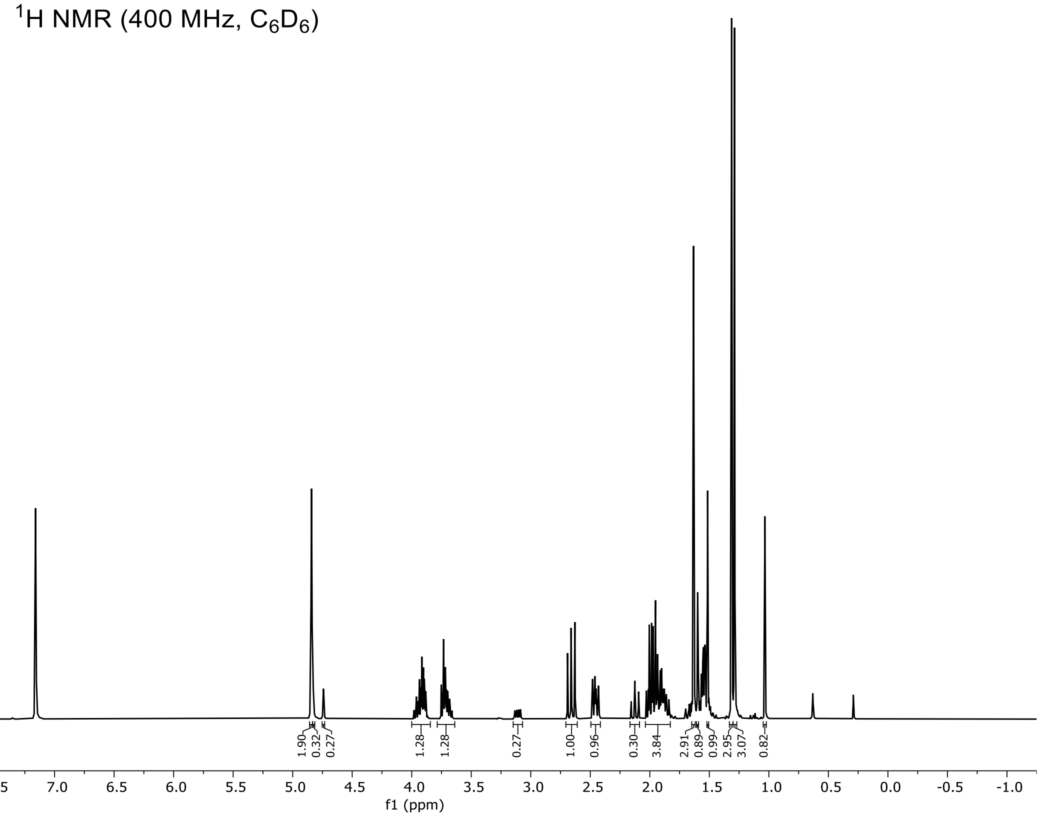

^{1}H NMR (400 MHz, C_6D_6)
1.90 0.32 0.27 1.28 1.28 0.27 1.00 0.96 0.30 3.84 2.91 0.89 0.99 2.95 3.07 0.82
9.0 8.5 8.0 7.5 7.0 6.5 6.0 5.5 5.0 4.5 4.0 3.5 3.0 2.5 2.0 1.5 1.0 0.5 0.0 -0.5 -1.0
f1 (ppm)

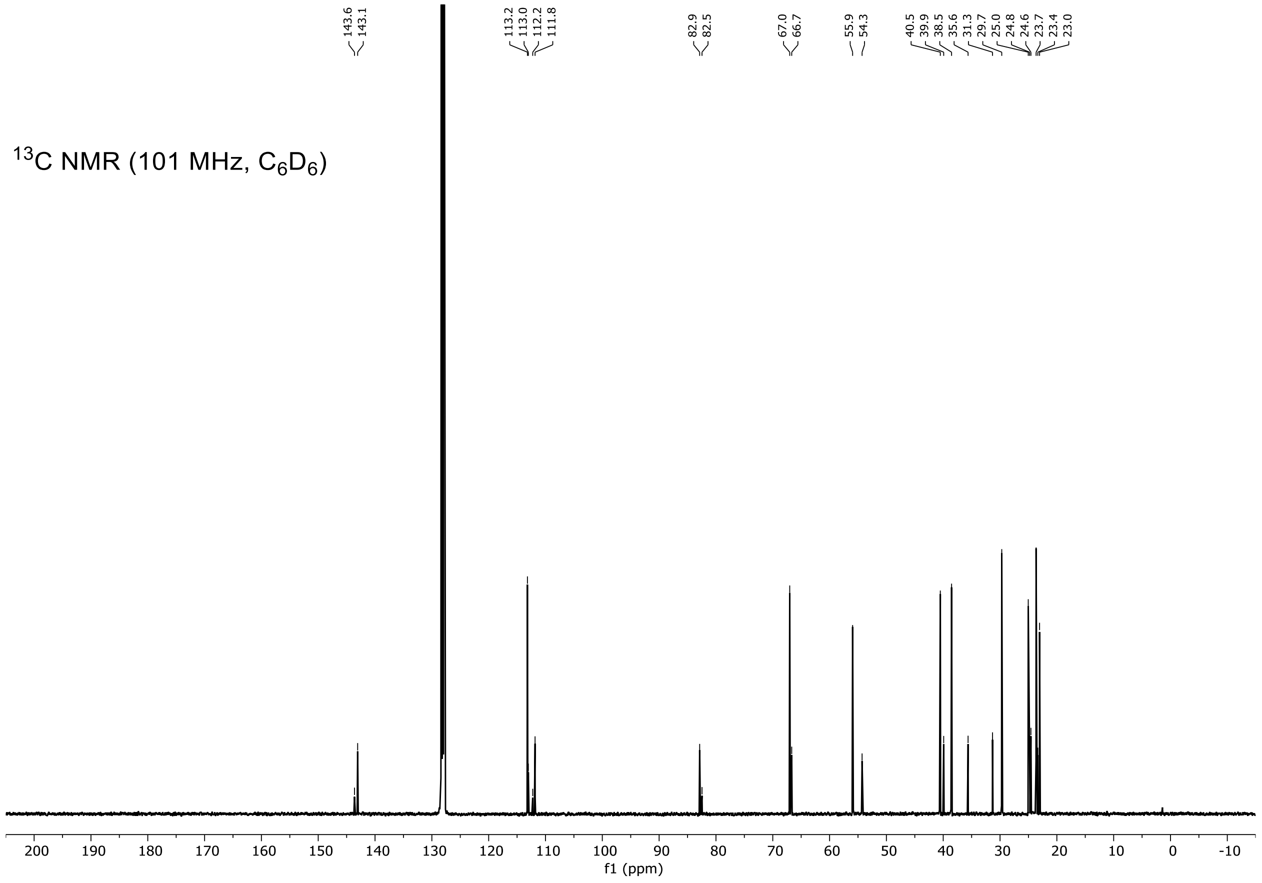

143.6 143.1 113.2 113.0 112.2 111.8 82.9 82.5 67.0 66.7 55.9 54.3 40.5 39.9 38.5 35.6 31.3 29.7 25.0 24.8 24.6 23.7 23.4 23.0
^{13}C NMR (101 MHz, C_6D_6)
200 190 180 170 160 150 140 130 120 110 100 90 80 70 60 50 40 30 20 10 0 -10
f1 (ppm)

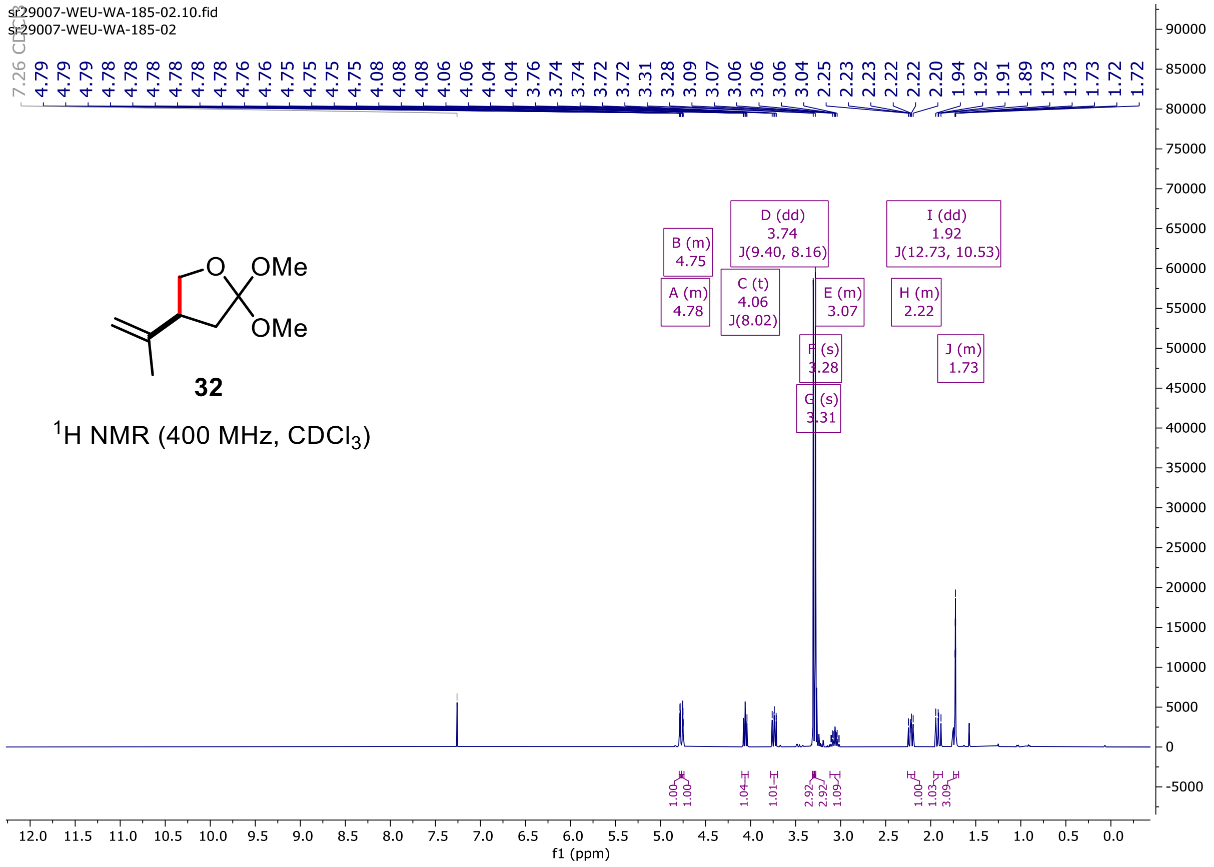
sr29007-WEU-WA-185-02.10.fid
sr29007-WEU-WA-185-02
7.26 CDCl3
4.79 4.79 4.79 4.78 4.78 4.78 4.78 4.78 4.76 4.76 4.75 4.75 4.75 4.75 4.08 4.08 4.08 4.06 4.06 4.04 4.04 3.76 3.74 3.74 3.72 3.72 3.31 3.28 3.09 3.07 3.06 3.06 3.06 3.04 2.25 2.23 2.22 2.22 2.22 2.20 1.94 1.92 1.91 1.89 1.73 1.73 1.73 1.72 1.72
OMe
OMe
O
32
1H NMR (400 MHz, CDCl3)
A (m) 4.78
B (m) 4.75
C (t) 4.06 J(8.02)
D (dd) 3.74 J(9.40, 8.16)
E (m) 3.07
F (s) 3.28
G (s) 3.31
H (m) 2.22
I (dd) 1.92 J(12.73, 10.53)
J (m) 1.73
1.00 1.00 1.04 1.01 2.92 2.92 1.09 1.00 1.03 3.09
f1 (ppm)

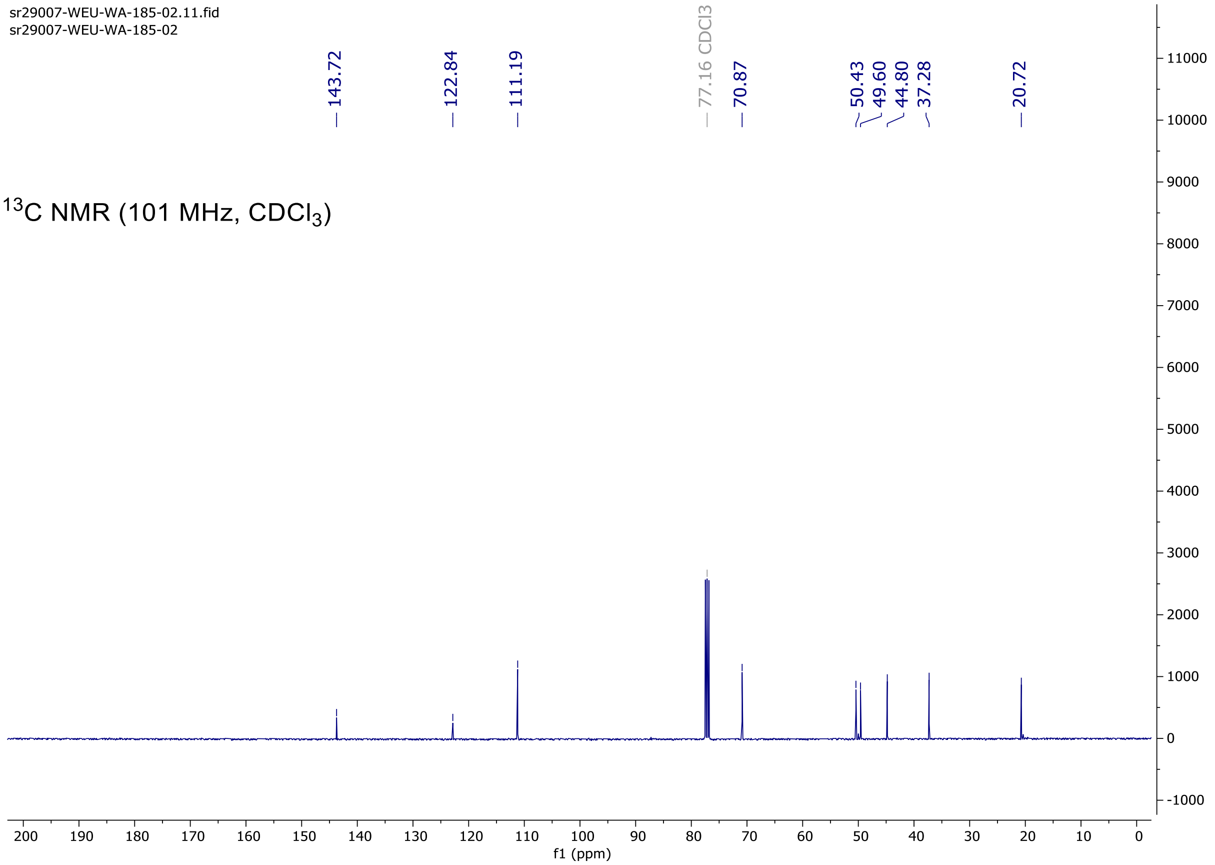
sr29007-WEU-WA-185-02.11.fid
sr29007-WEU-WA-185-02
143.72
122.84
111.19
77.16 CDCl3
70.87
50.43
49.60
44.80
37.28
20.72
13C NMR (101 MHz, CDCl3)
f1 (ppm)

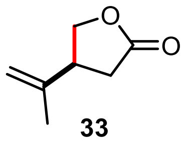

33

^{1}H NMR (400 MHz, $CDCl_3$)

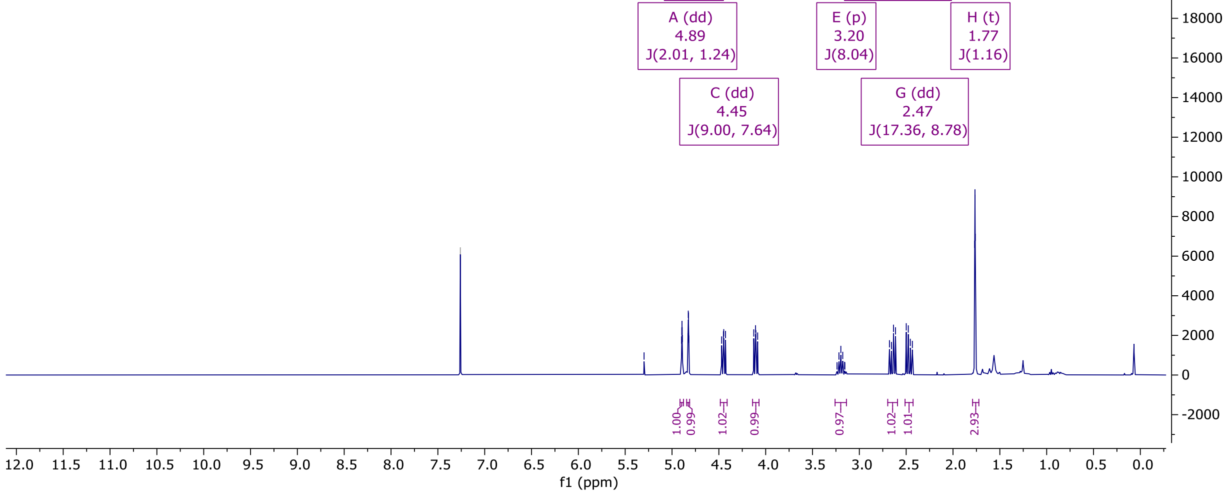

^{13}C NMR (101 MHz, $CDCl_3$)

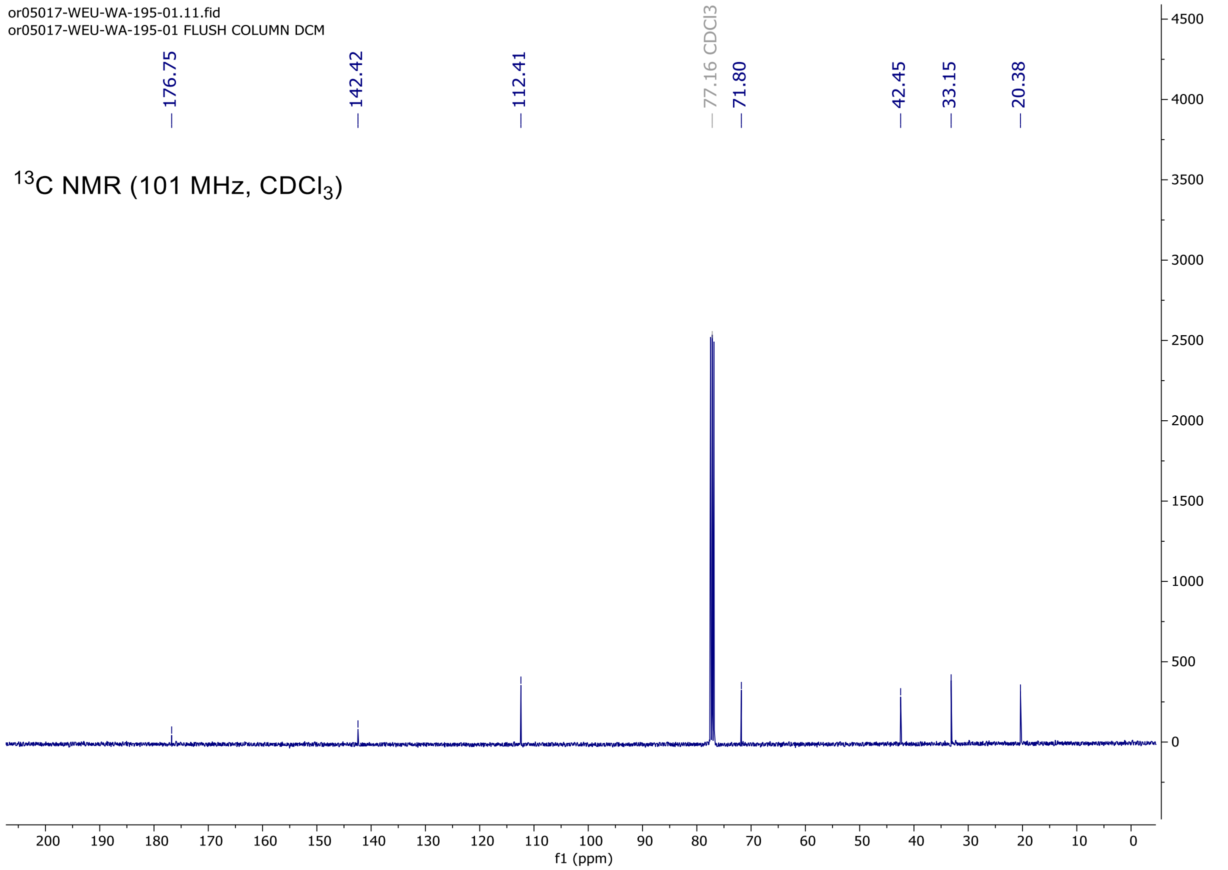

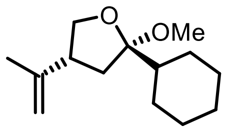

34

^{1}H NMR (400 MHz, C_6D_6)

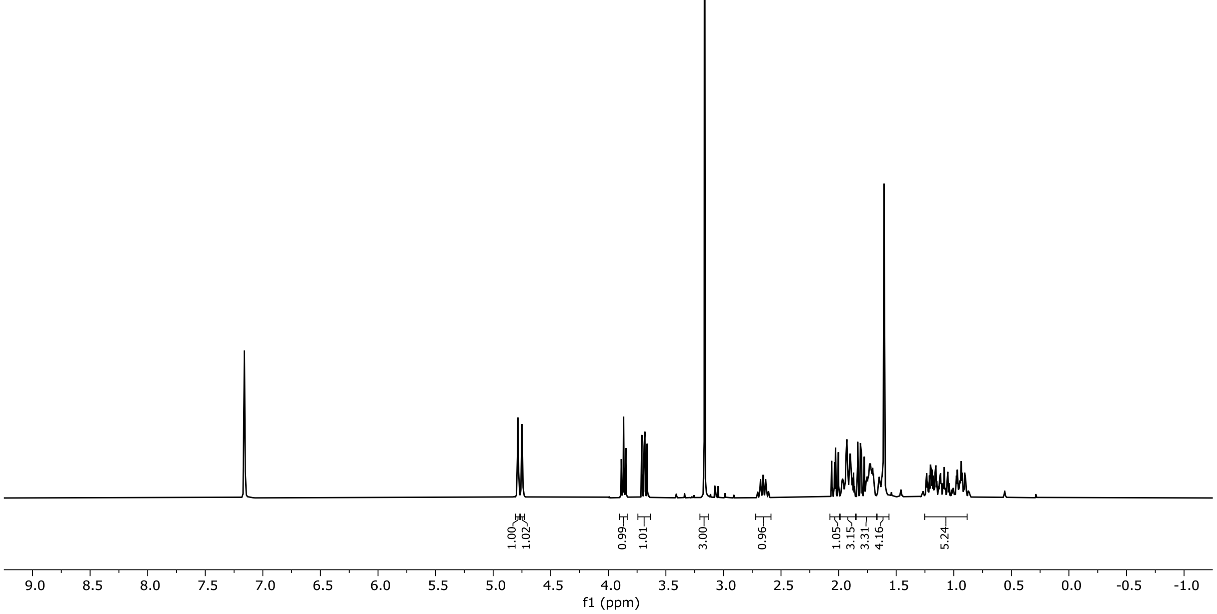

^{13}C NMR (101 MHz, C_6D_6)

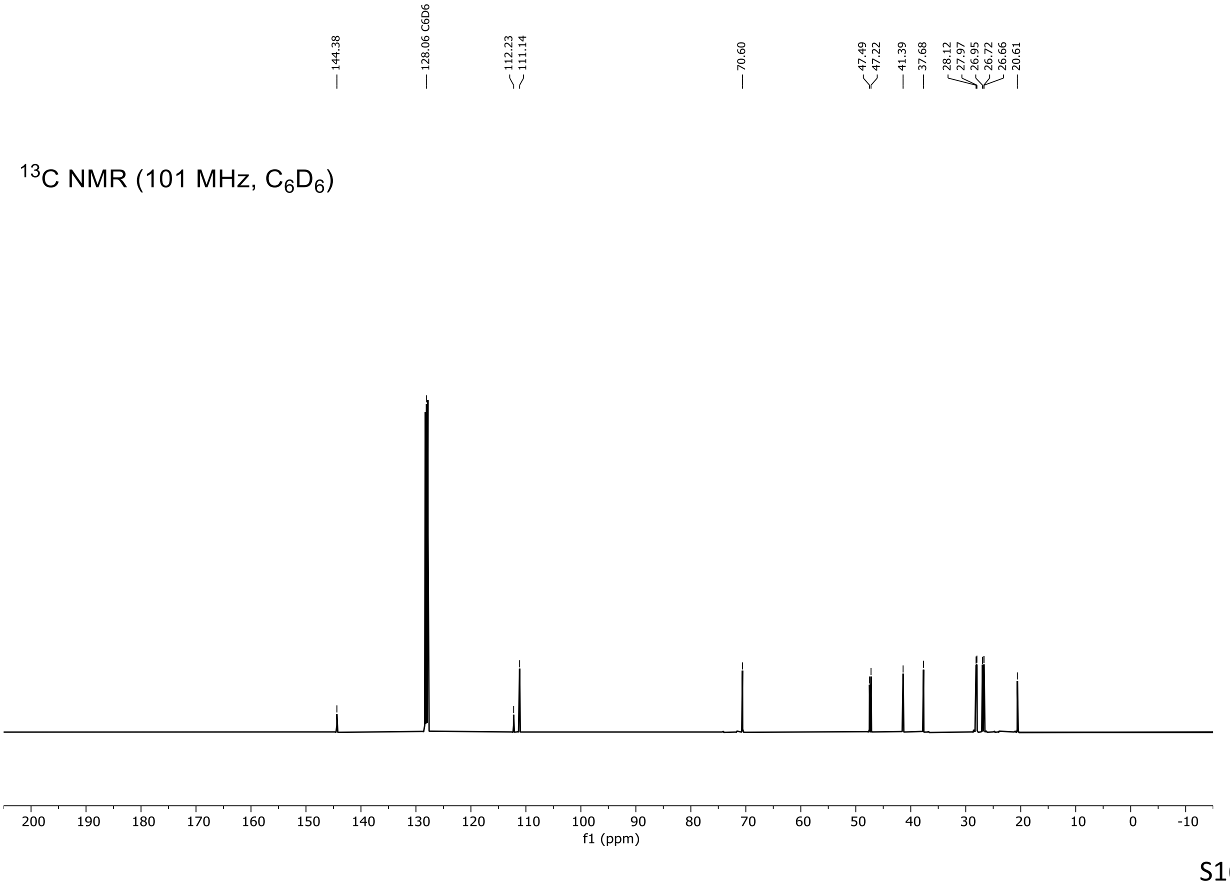

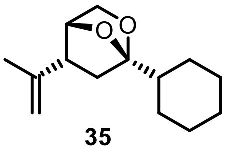

35

^{1}H NMR (400 MHz, C_6D_6)

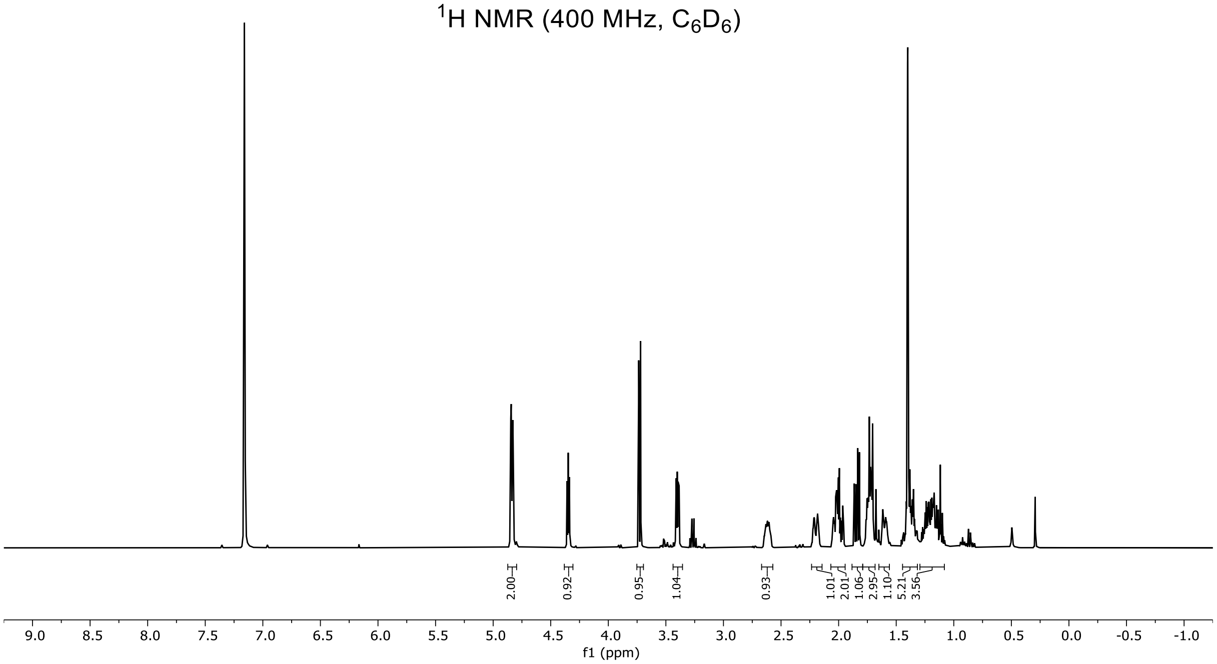

^{13}C NMR (101 MHz, C_6D_6)

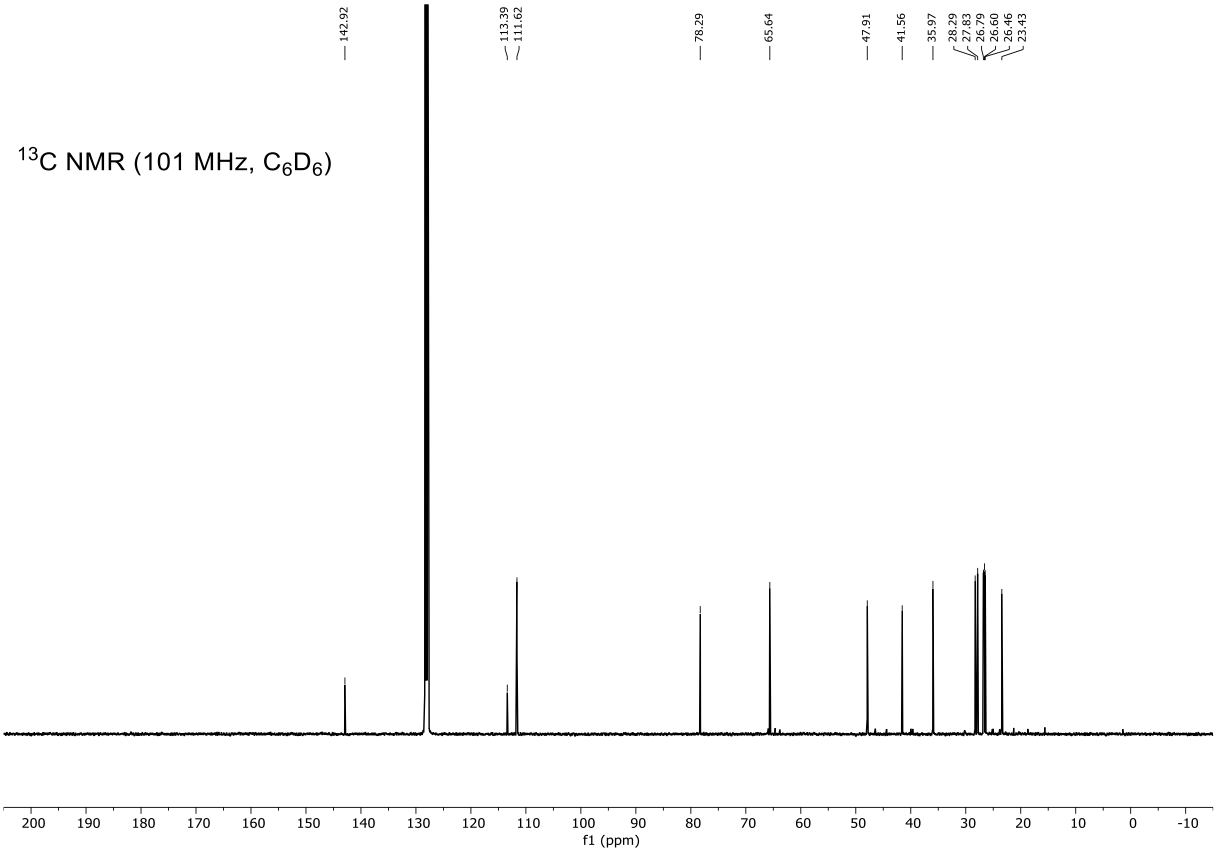

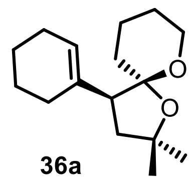

36a

^{1}H NMR (400 MHz, C_6D_6)

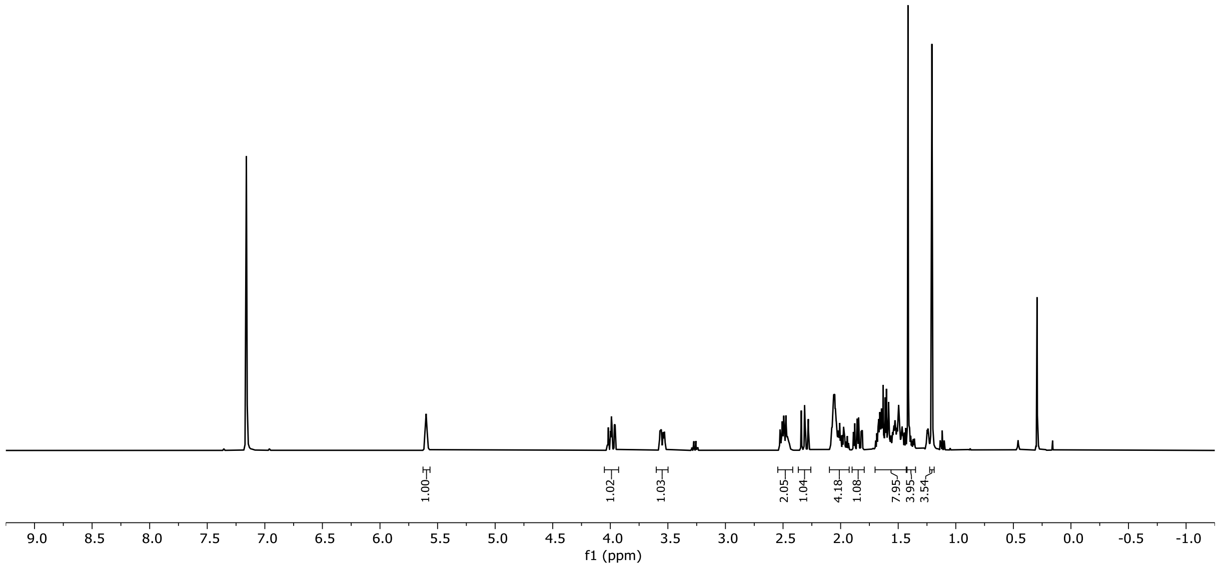

^{13}C NMR (101 MHz, C_6D_6)

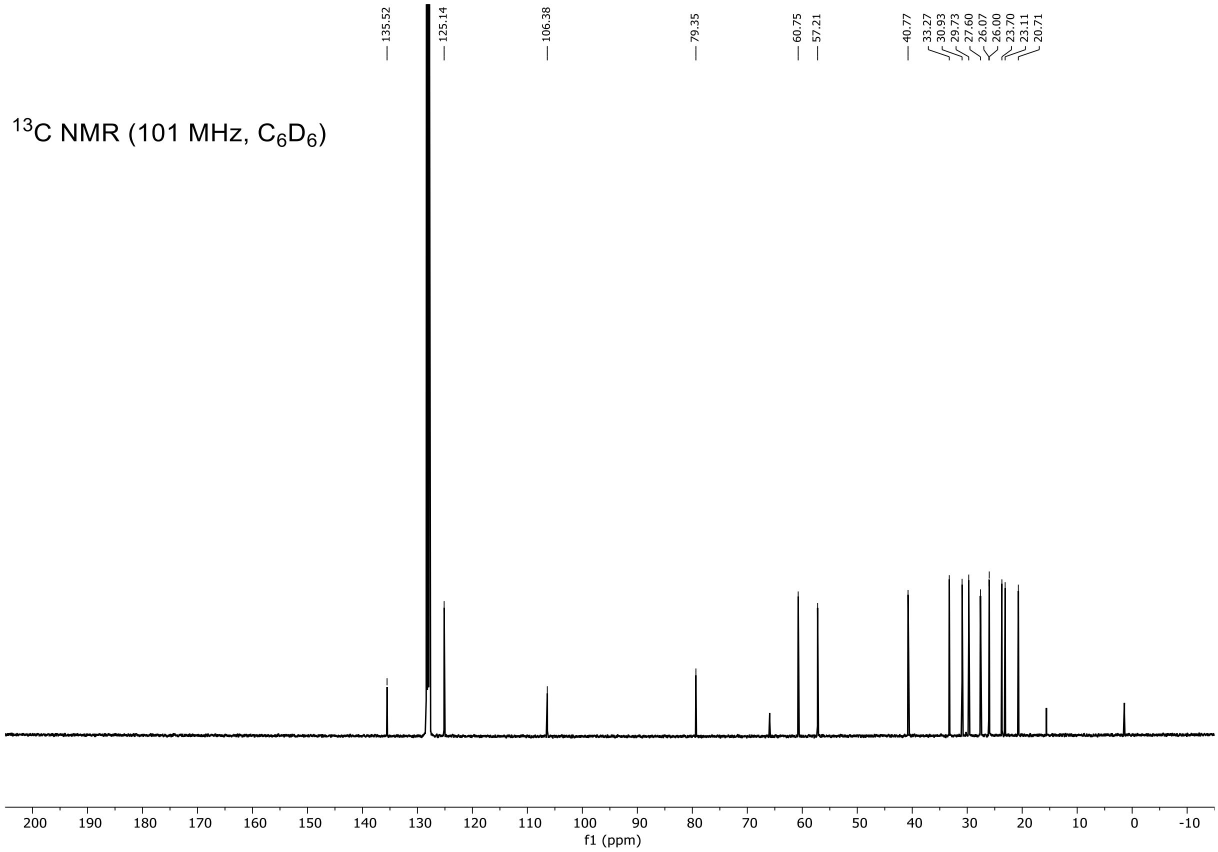

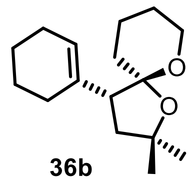

36b

^1H NMR (400 MHz, C_6D_6)

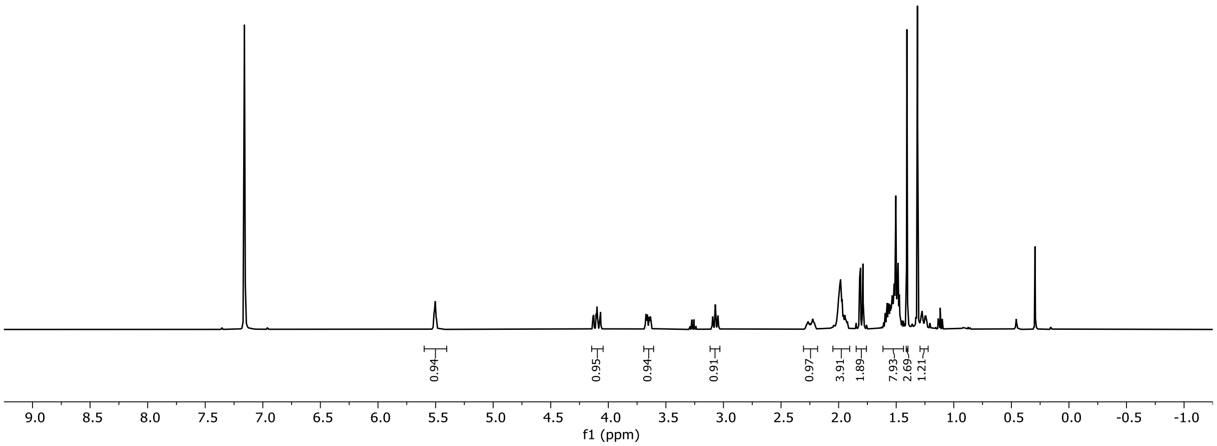

^{13}C NMR (101 MHz, C_6D_6)

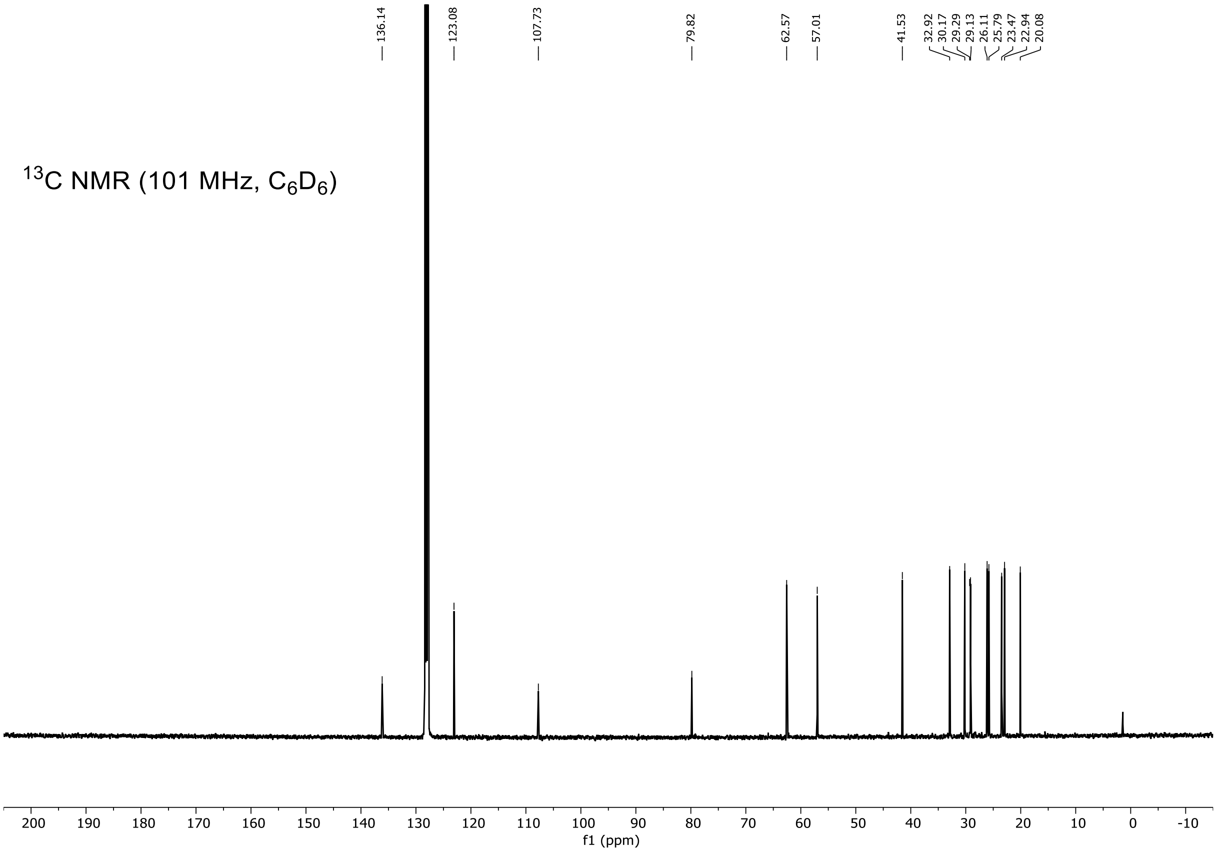

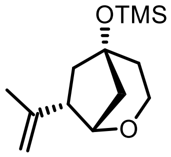

40

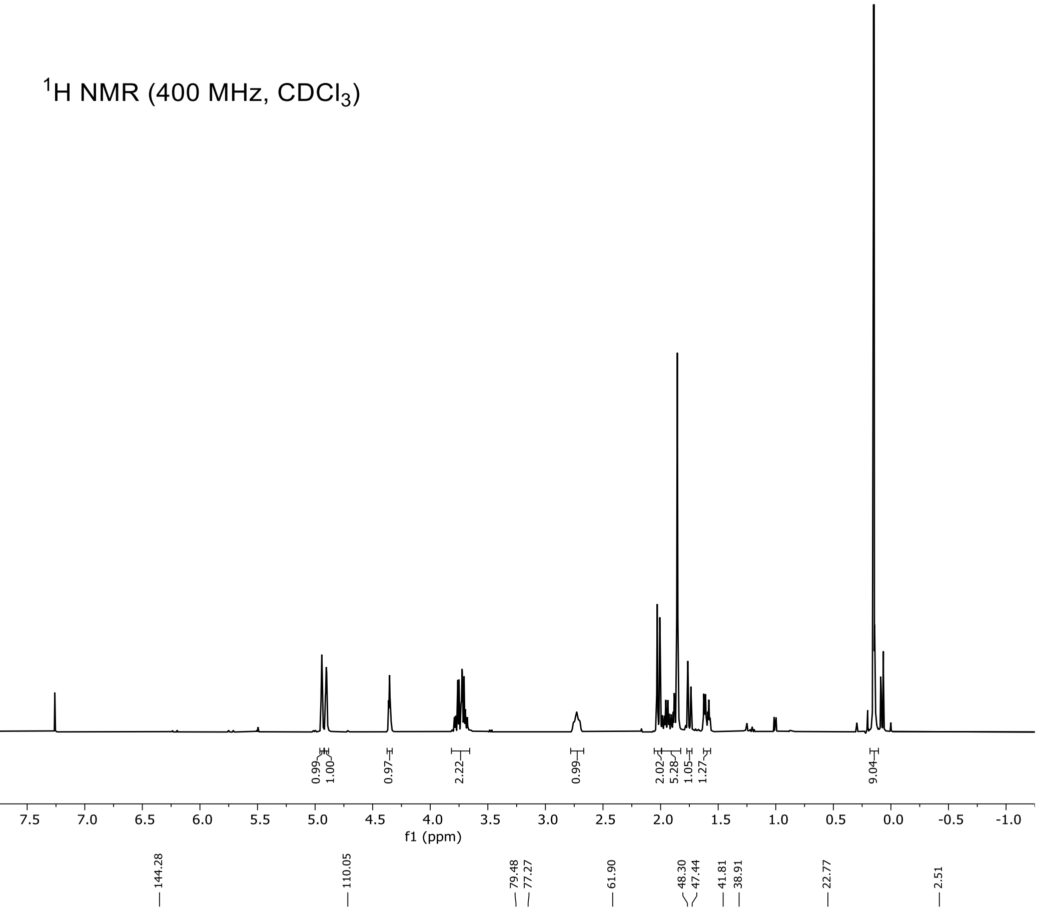

^{1}H NMR (400 MHz, $CDCl_3$)

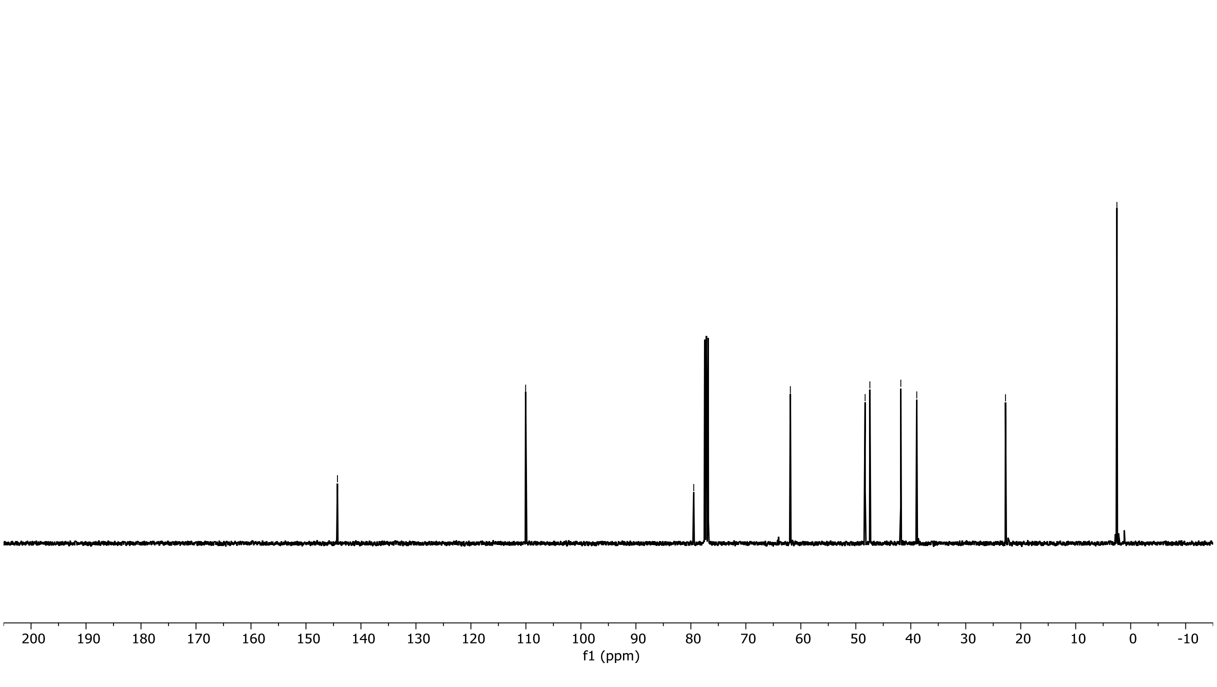

^{13}C NMR (101 MHz, $CDCl_3$)

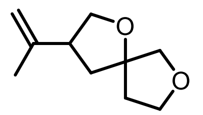

^{1}H NMR (400 MHz, $CDCl_3$)

41 (dr = 1.2:1)

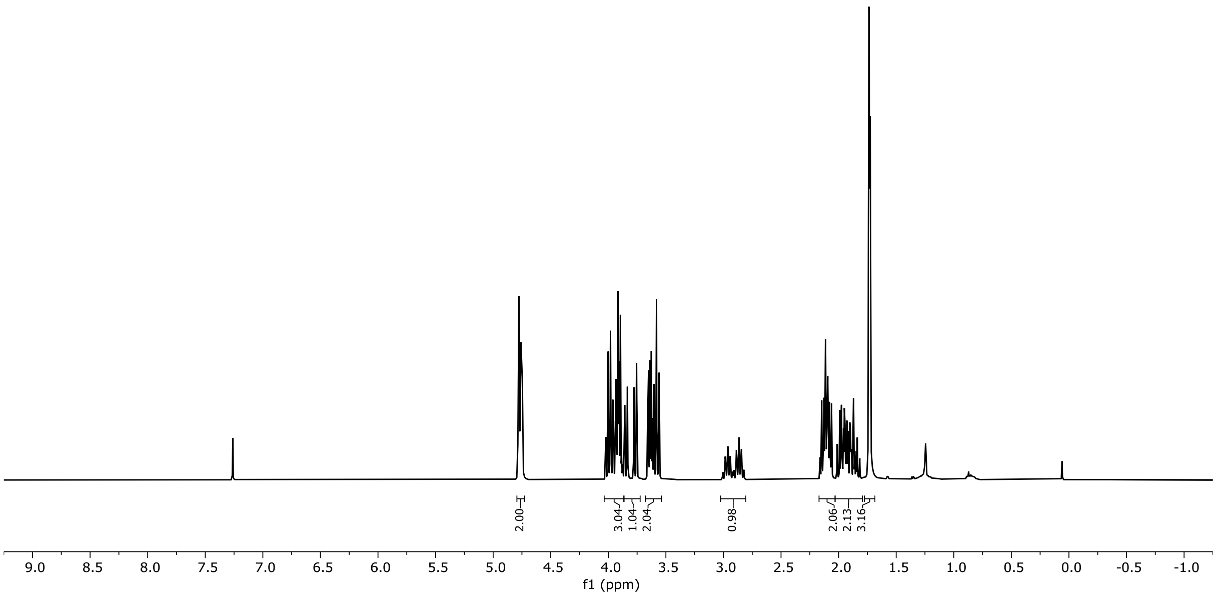

^{13}C NMR (101 MHz, $CDCl_3$)

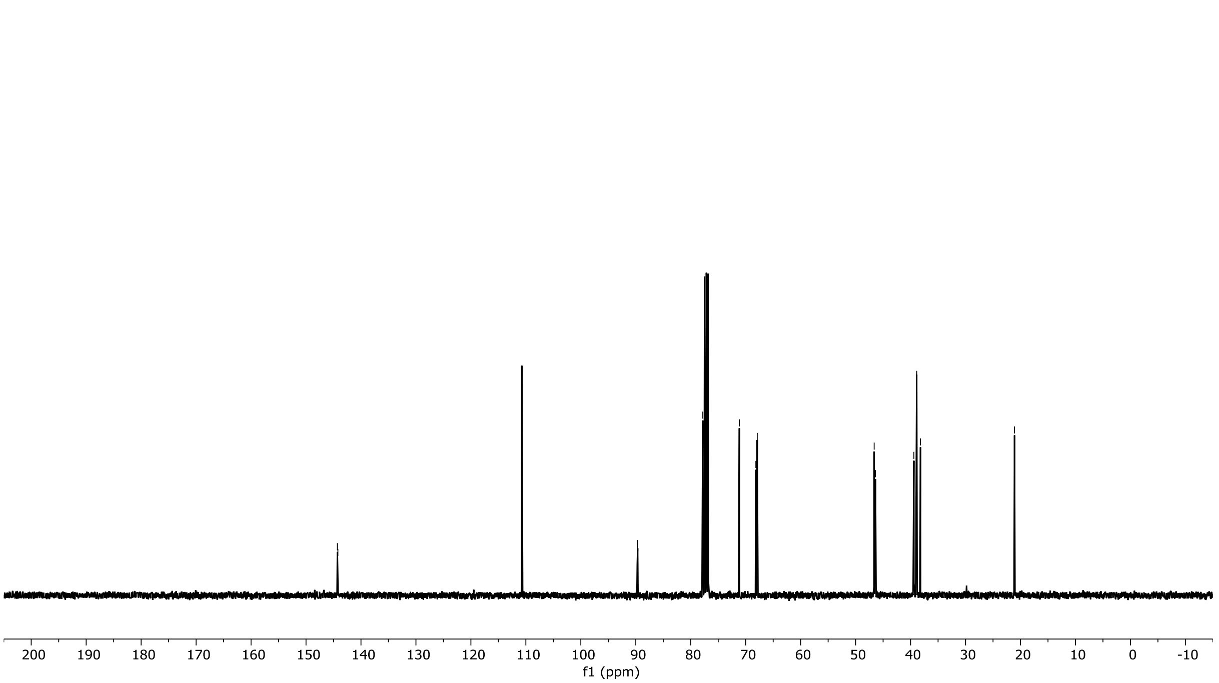

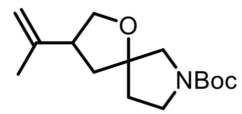

42 (dr = 1:1 & rotamers)

^{1}H NMR (400 MHz, $CDCl_3$)

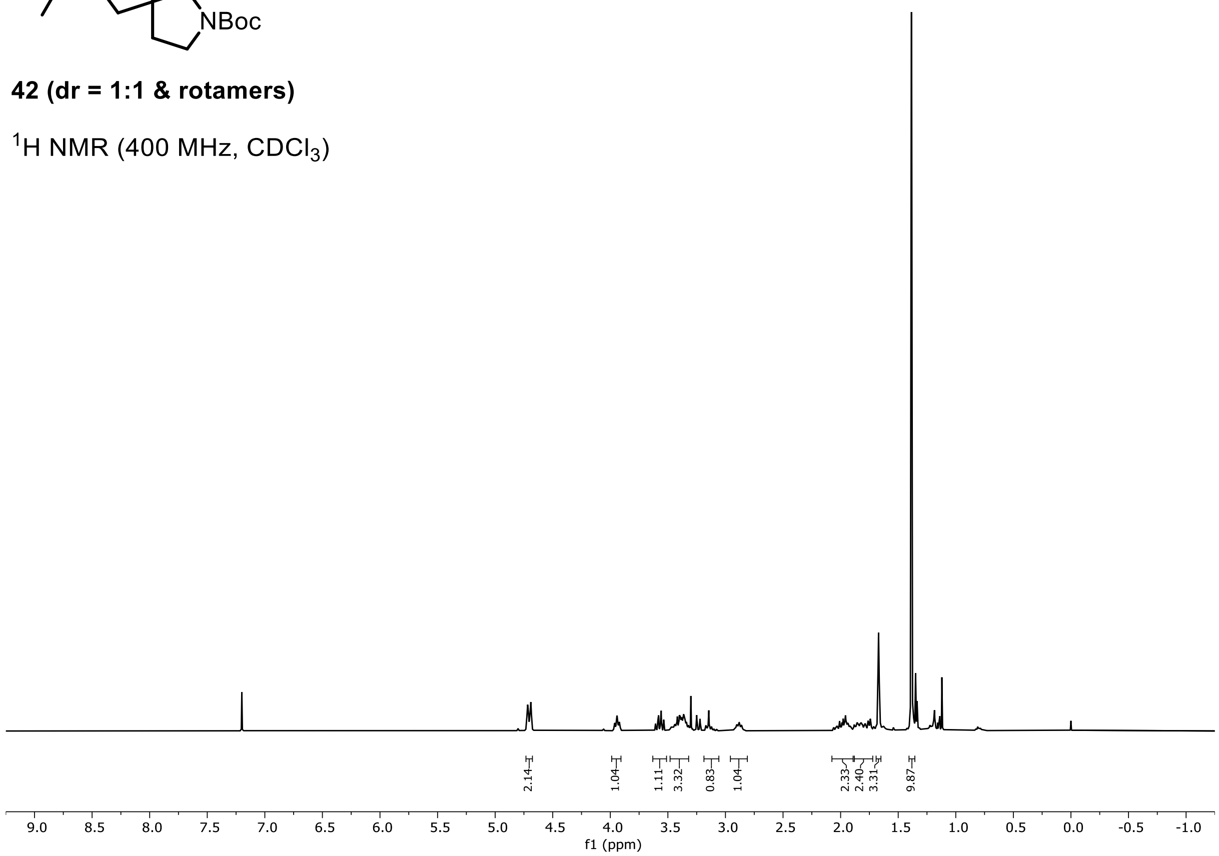

Mixture + rotamers

^{13}C NMR (101 MHz, $CDCl_3$)

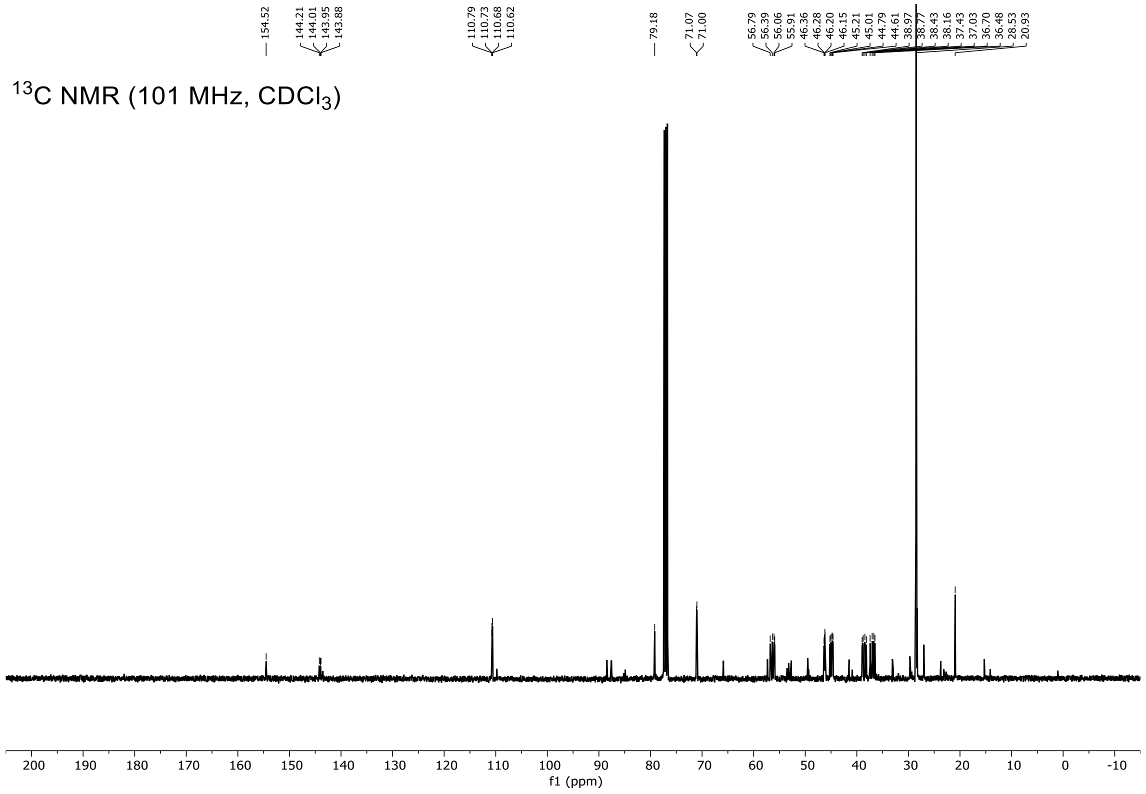

Diastereoisomer **42a** (rotamers)

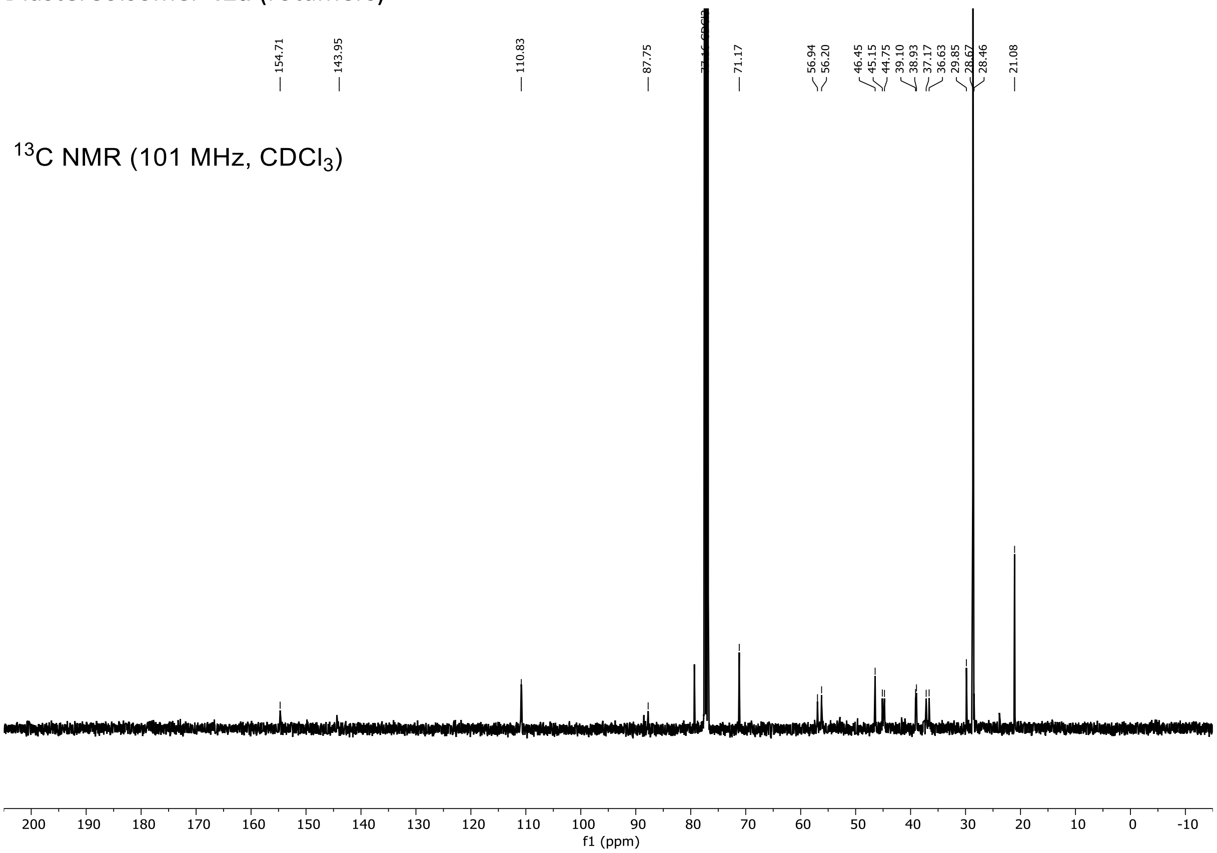

43

^{1}H NMR (400 MHz, $CDCl_3$)

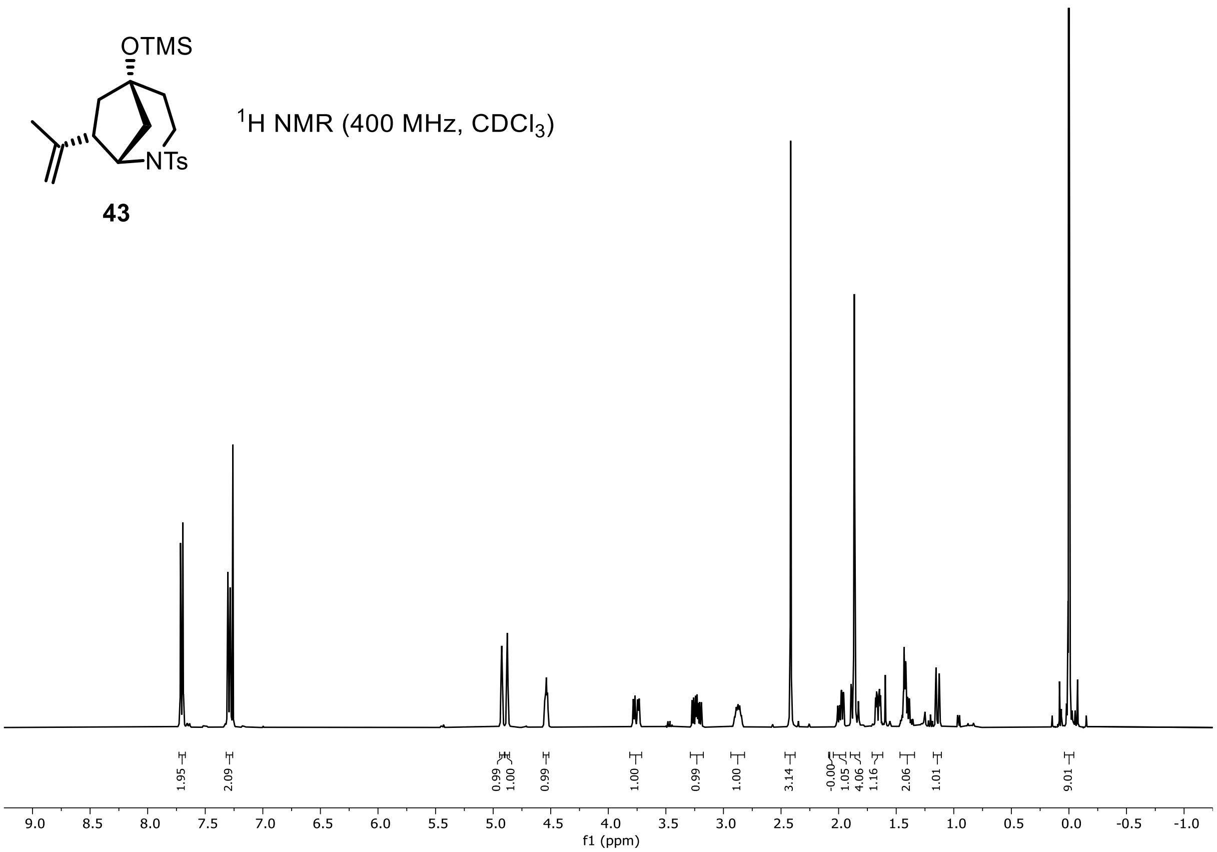

^{13}C NMR (101 MHz, $CDCl_3$)

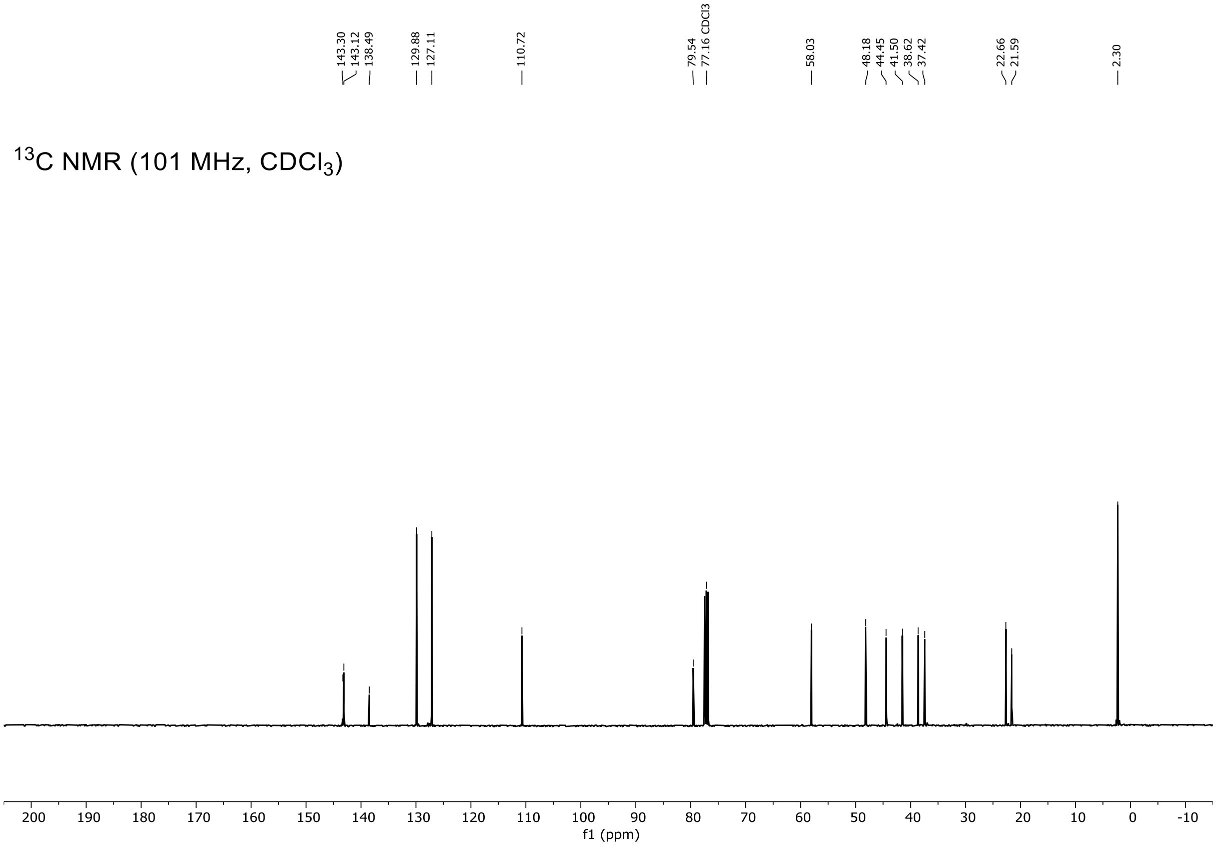

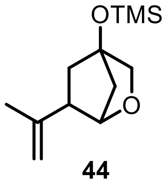

44

^{1}H NMR (400 MHz, $CDCl_3$)

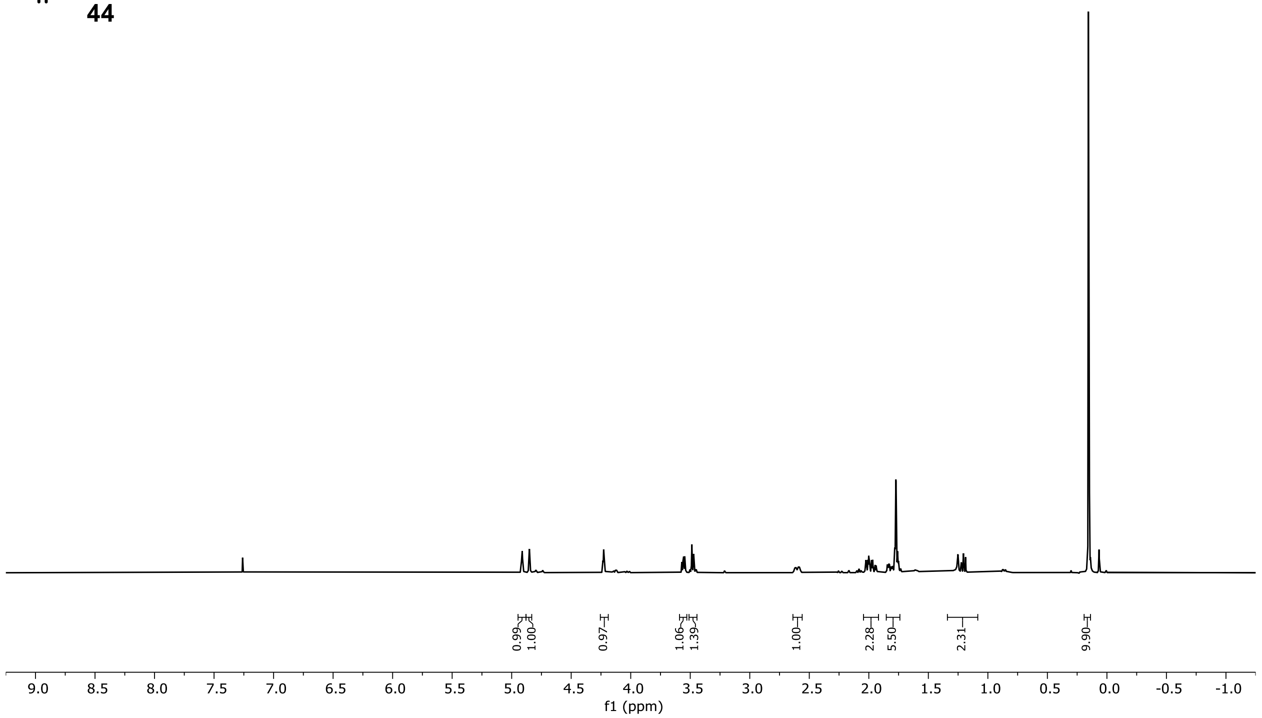

^{13}C NMR (101 MHz, $CDCl_3$)

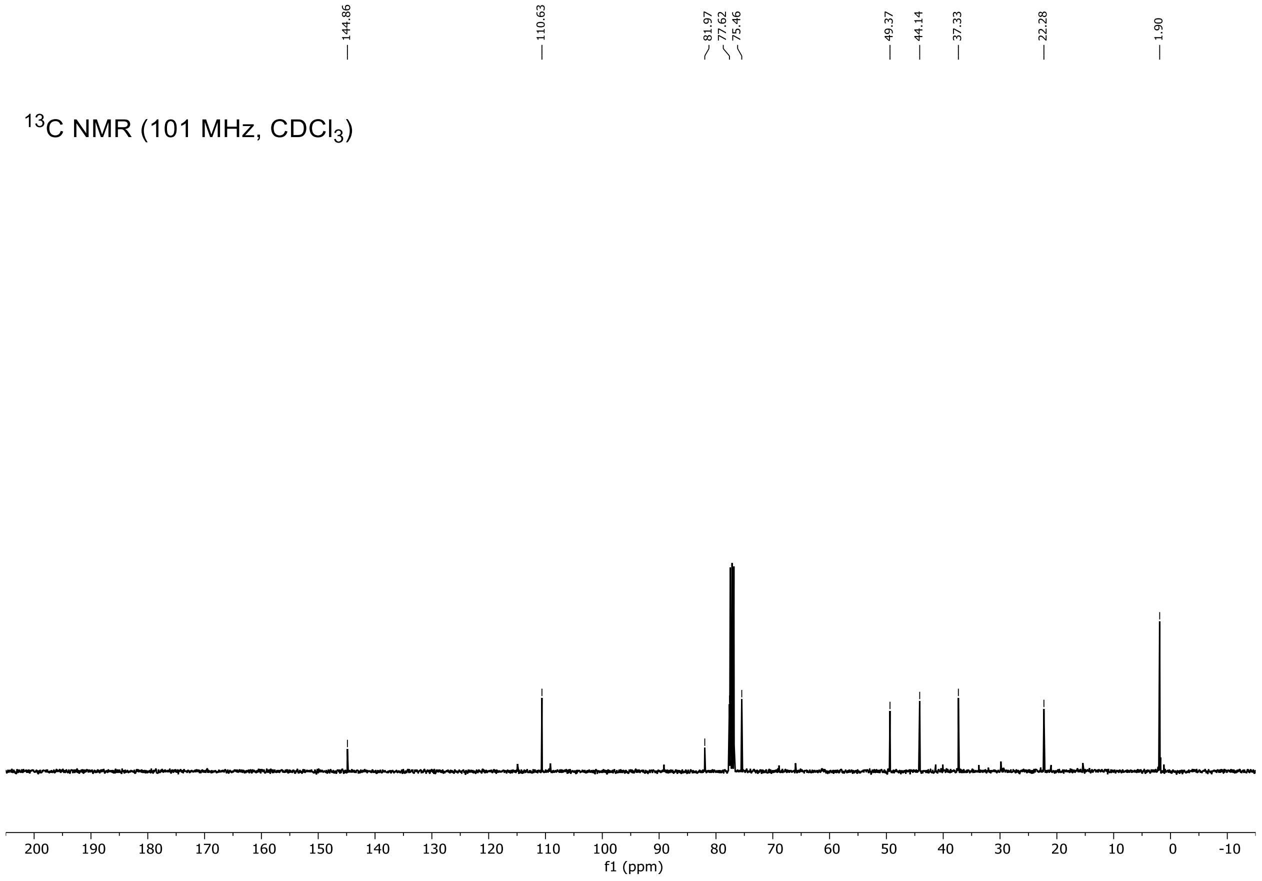

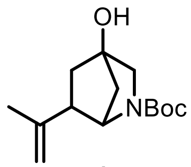

45

^{1}H NMR (400 MHz, $CDCl_3$, rotamers)

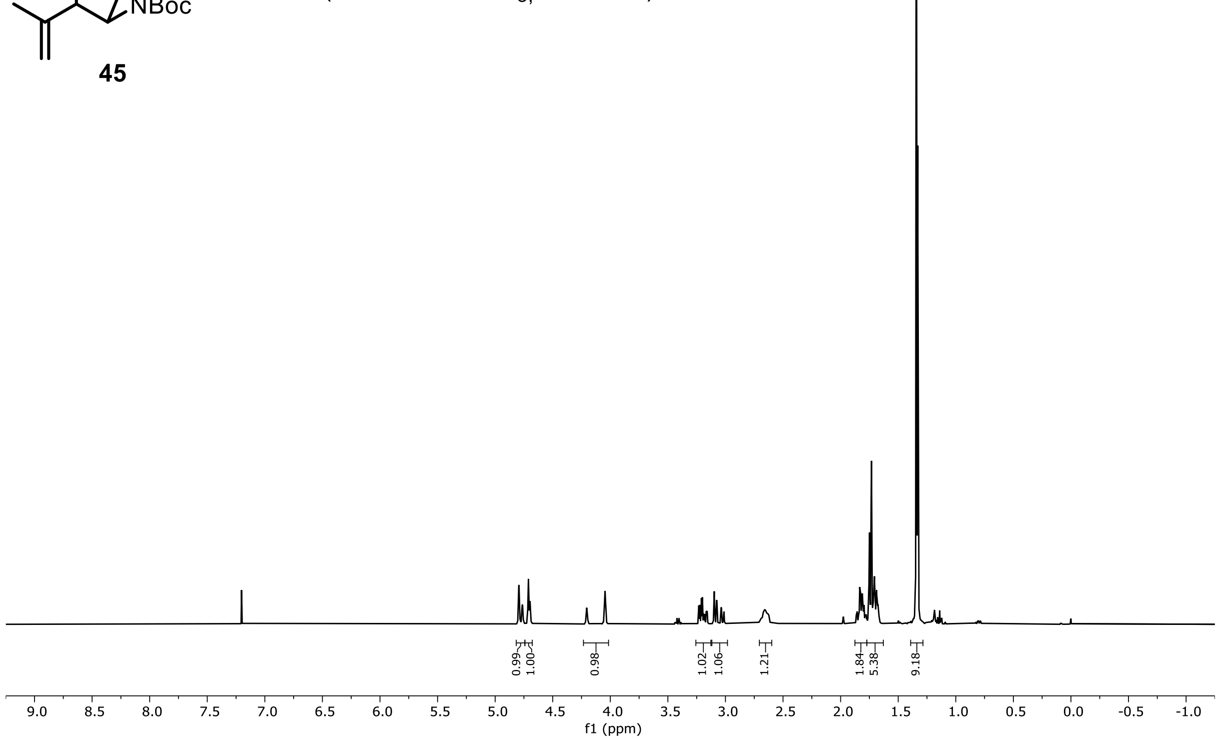

^{13}C NMR (101 MHz, $CDCl_3$, rotamers)

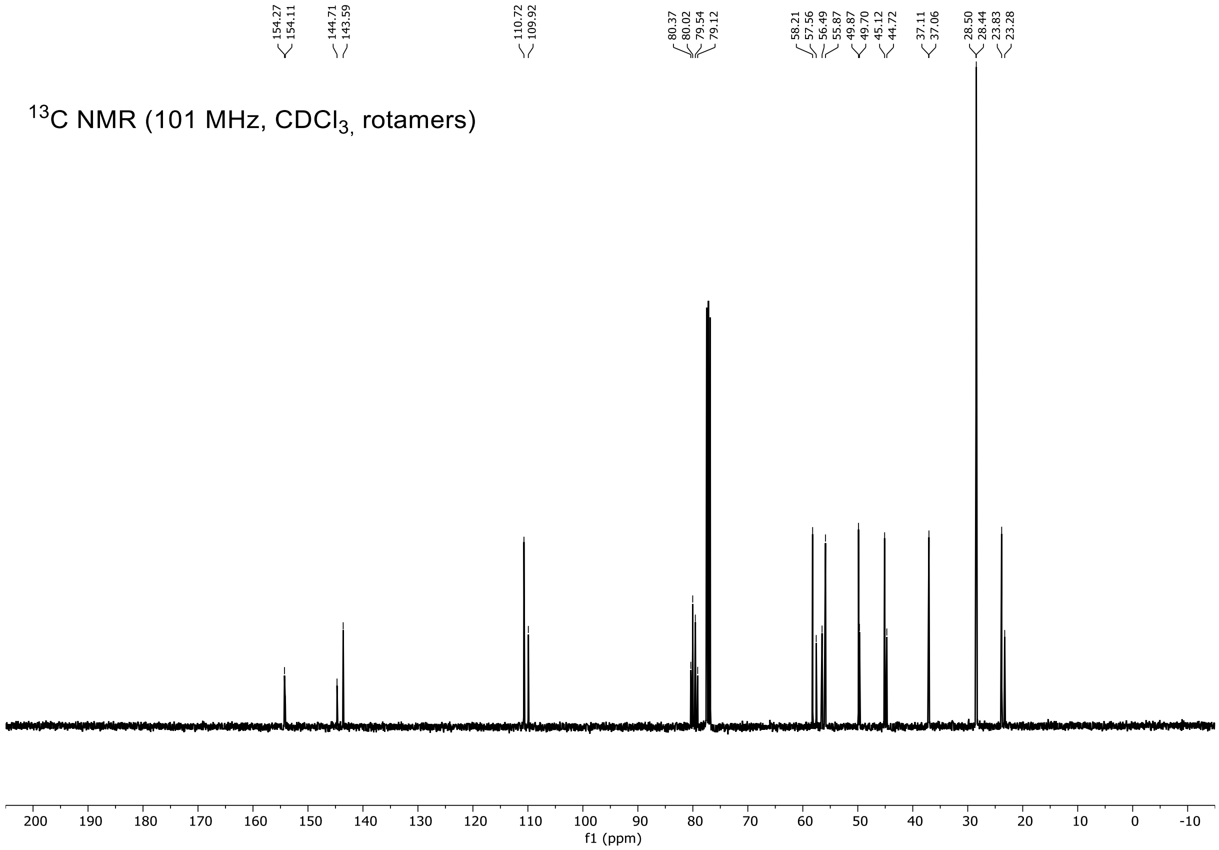

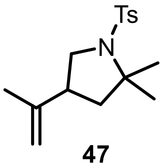

47

^{1}H NMR (400 MHz, $CDCl_3$)

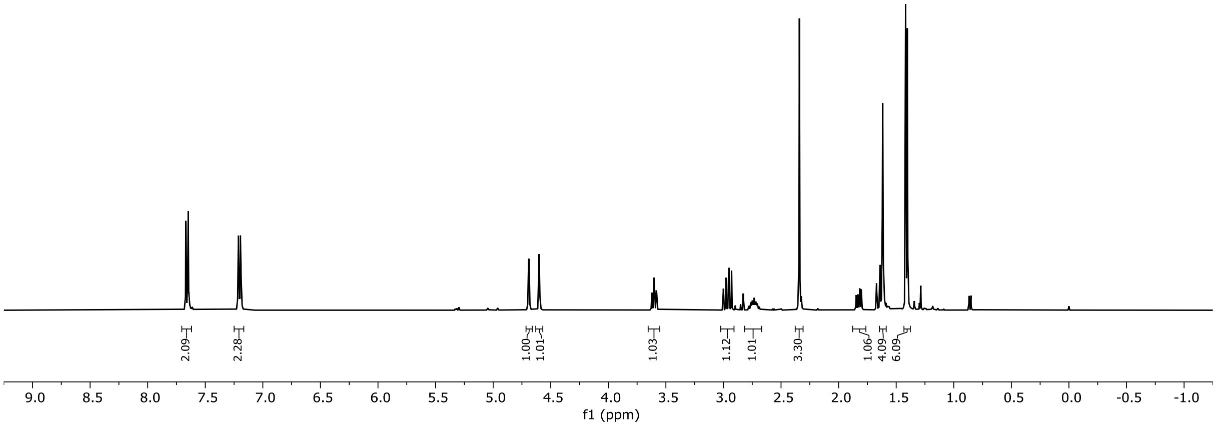

^{13}C NMR (101 MHz, $CDCl_3$)

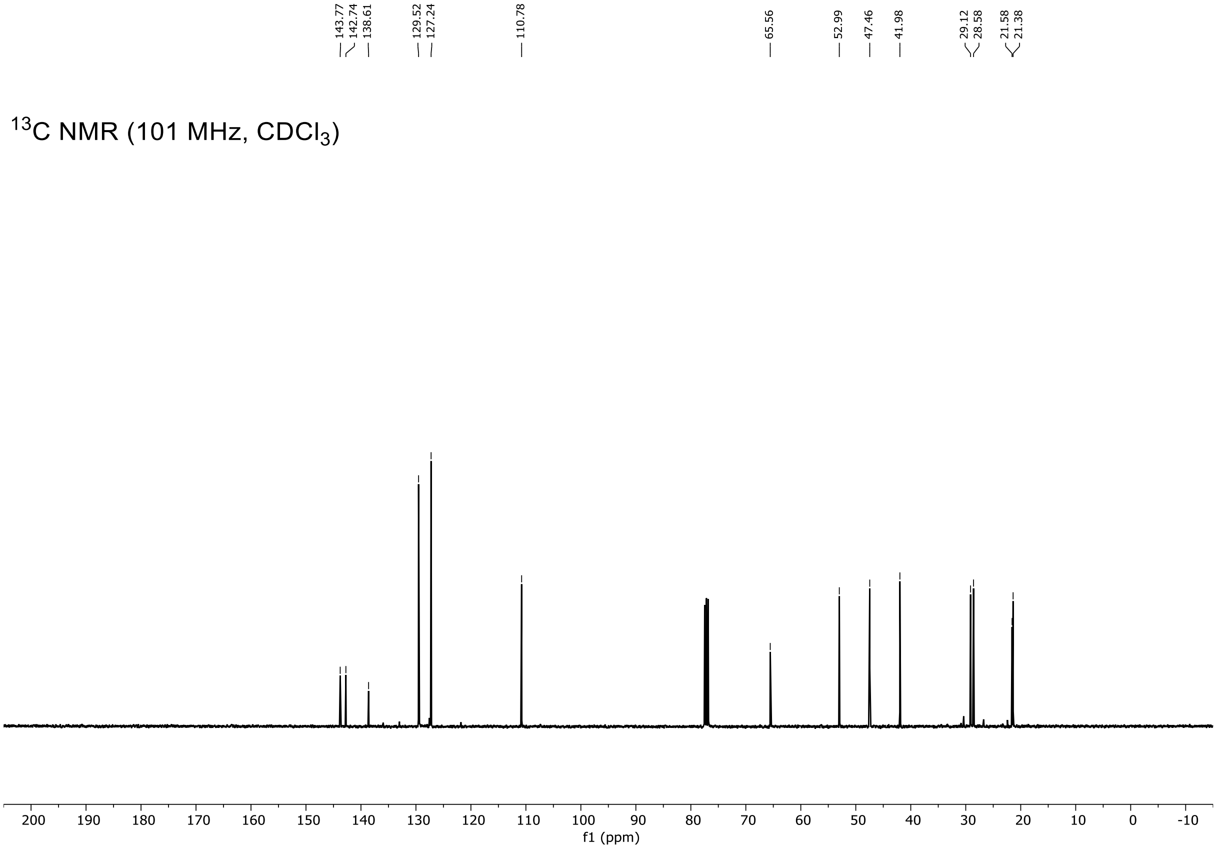

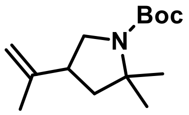

48

^{1}H NMR (600 MHz, $CDCl_{3,}$ -40°C, rotamers)

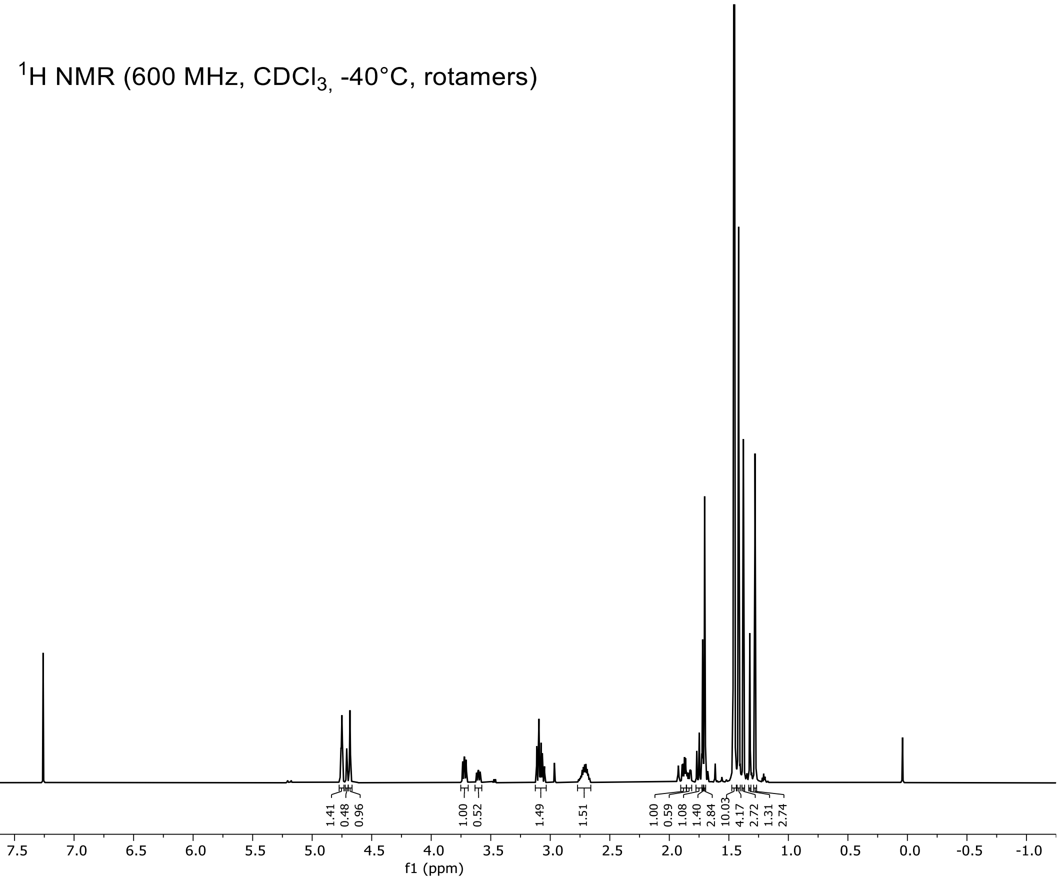

^{13}C NMR (151 MHz, $CDCl_{3,}$ -40°C, rotamers)

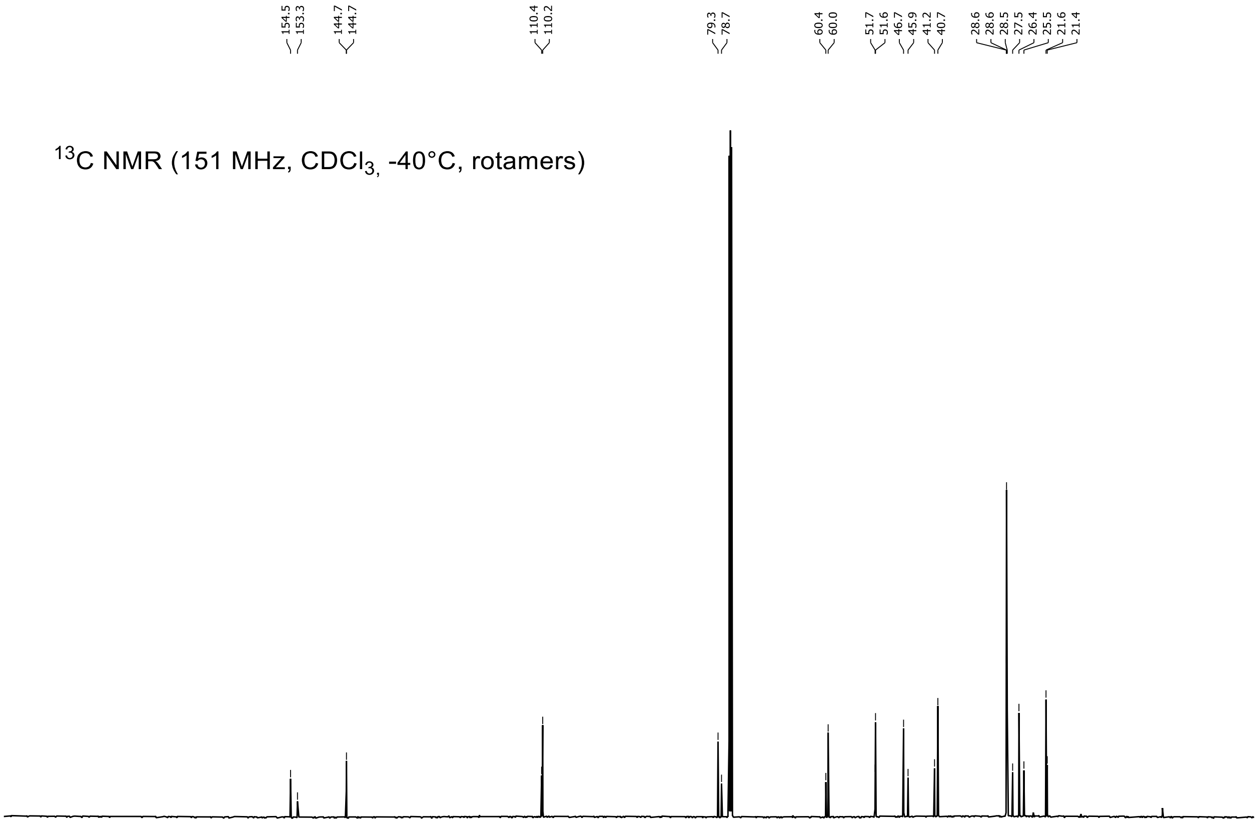

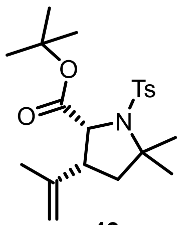

49

^{1}H NMR (400 MHz, $CDCl_3$)

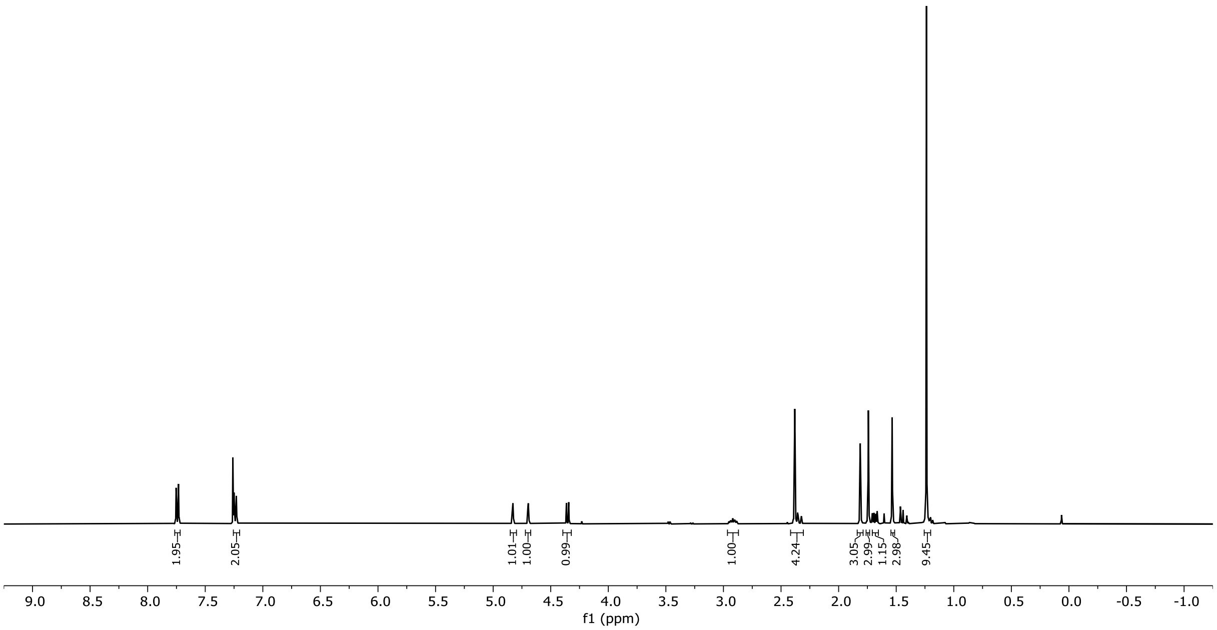

^{13}C NMR (101 MHz, $CDCl_3$)

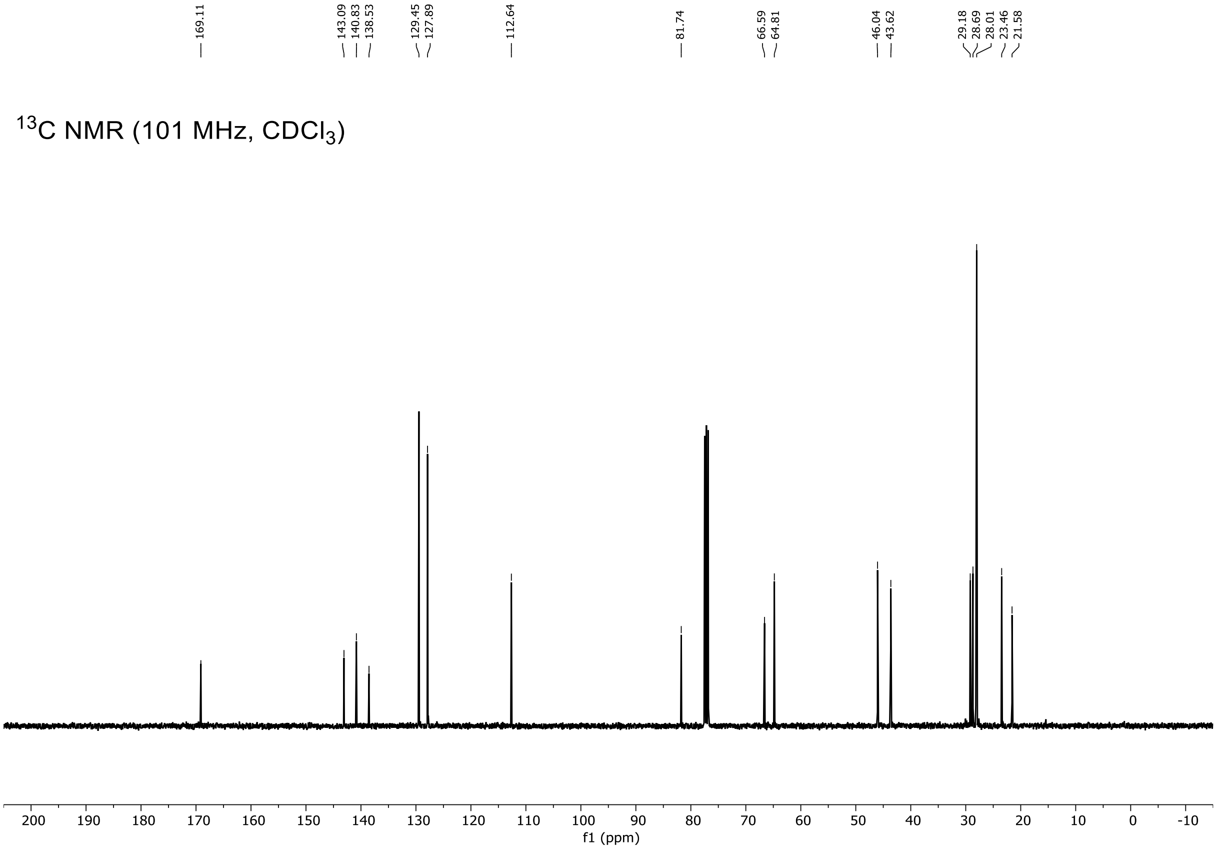

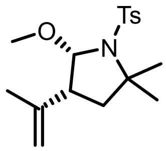

50

^{1}H NMR (400 MHz, C_6D_6)

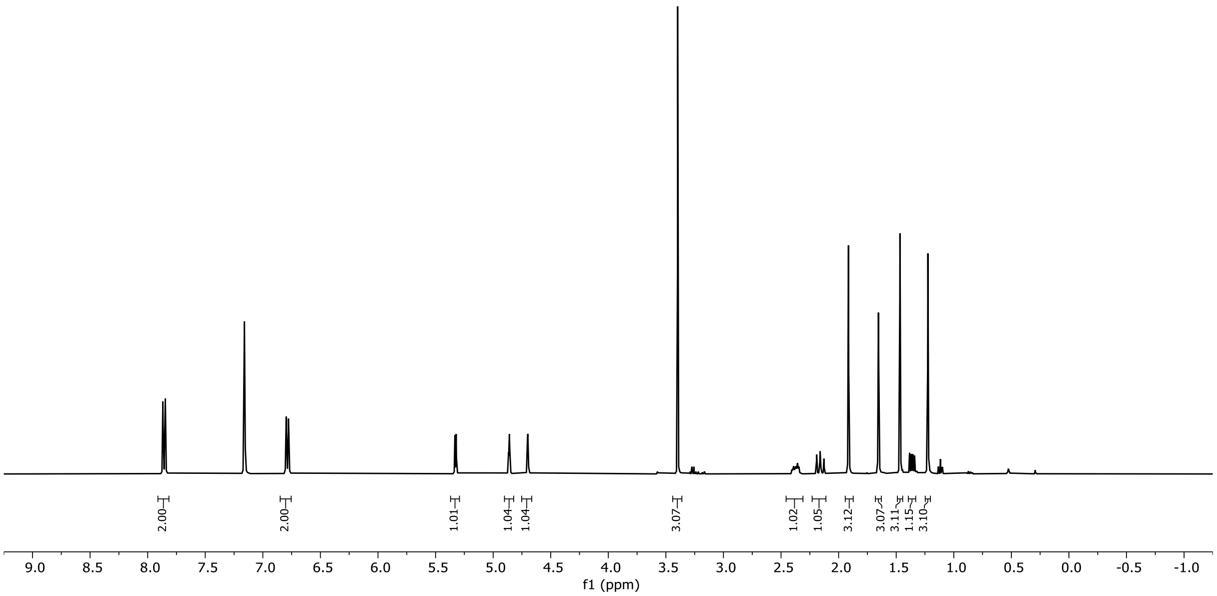

^{13}C NMR (101 MHz, C_6D_6)

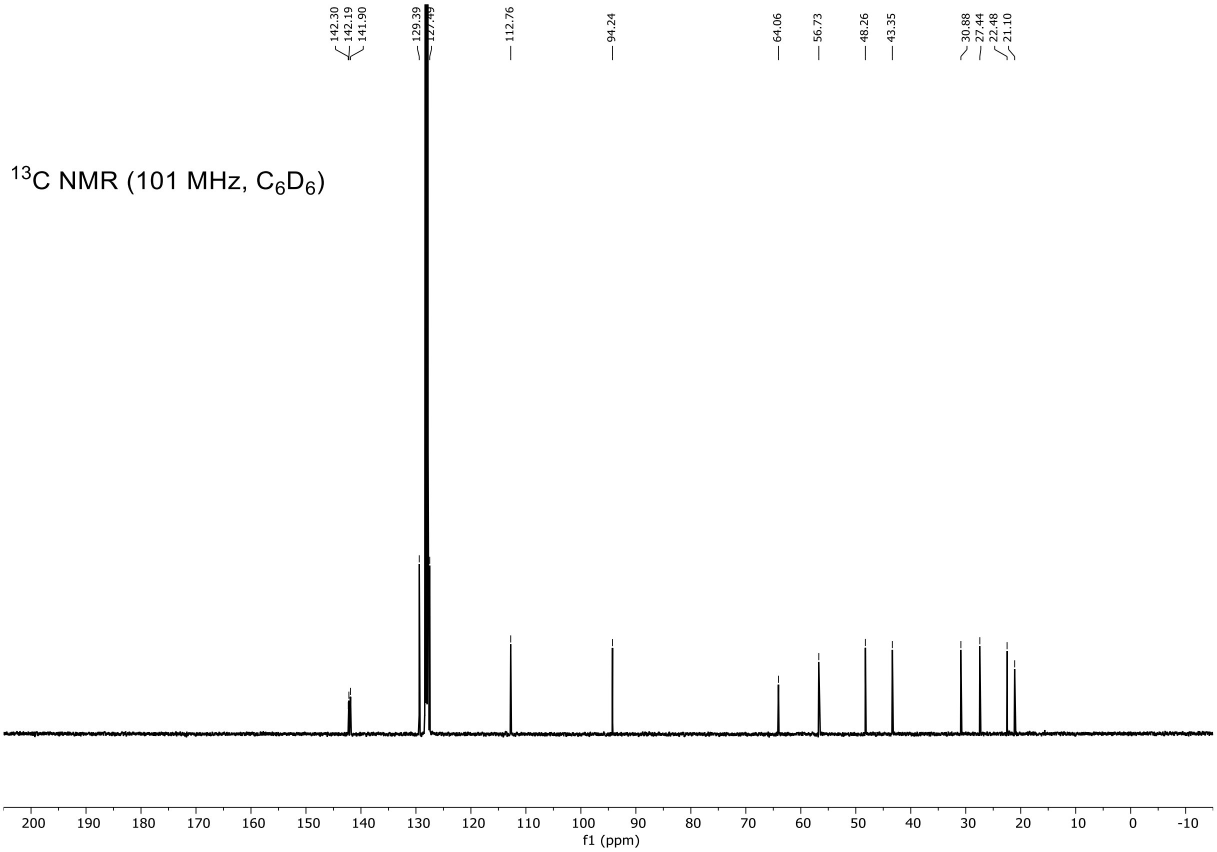

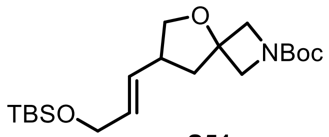

S51

^{1}H NMR (400 MHz, $CDCl_3$)

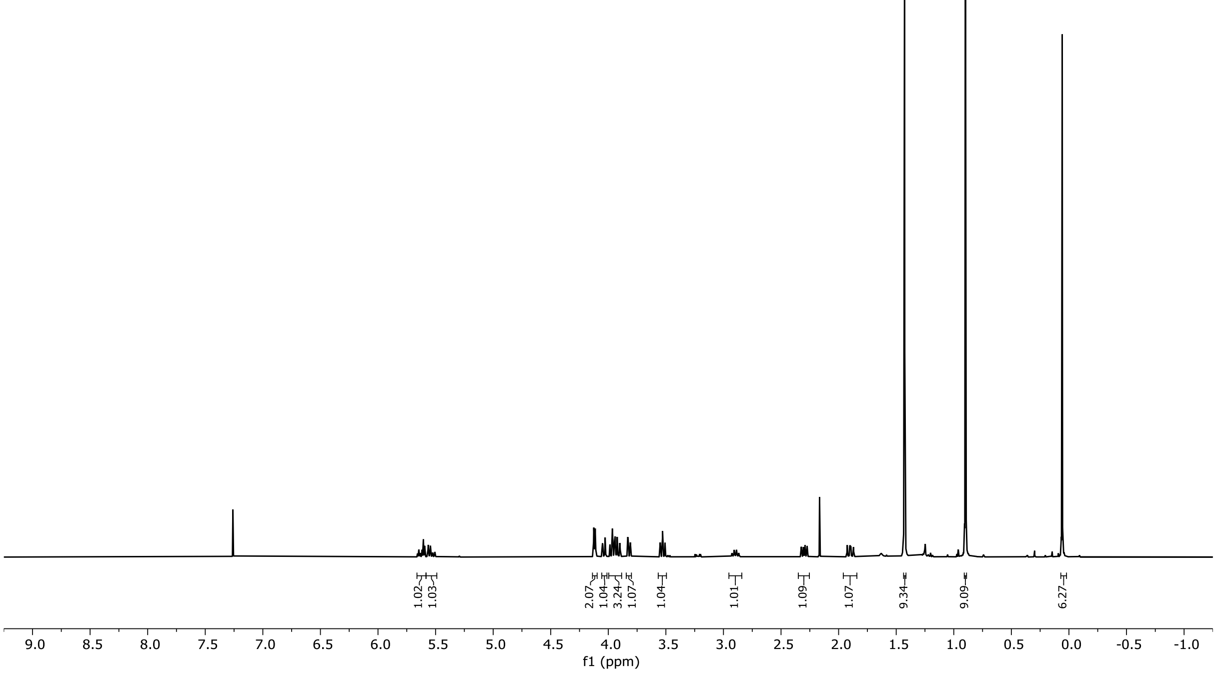

^{13}C NMR (101 MHz, $CDCl_3$)

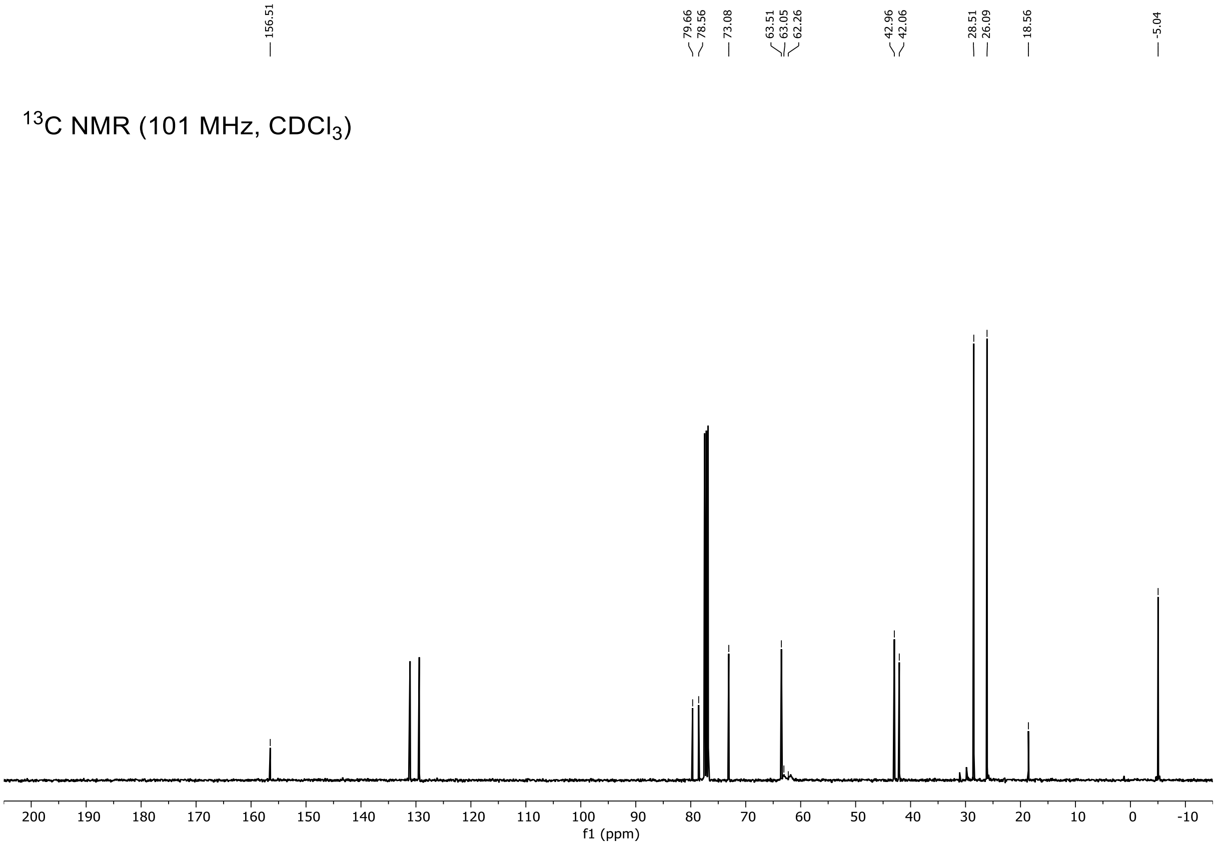

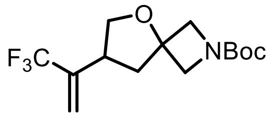

S52

^{1}H NMR (400 MHz, $CDCl_3$)

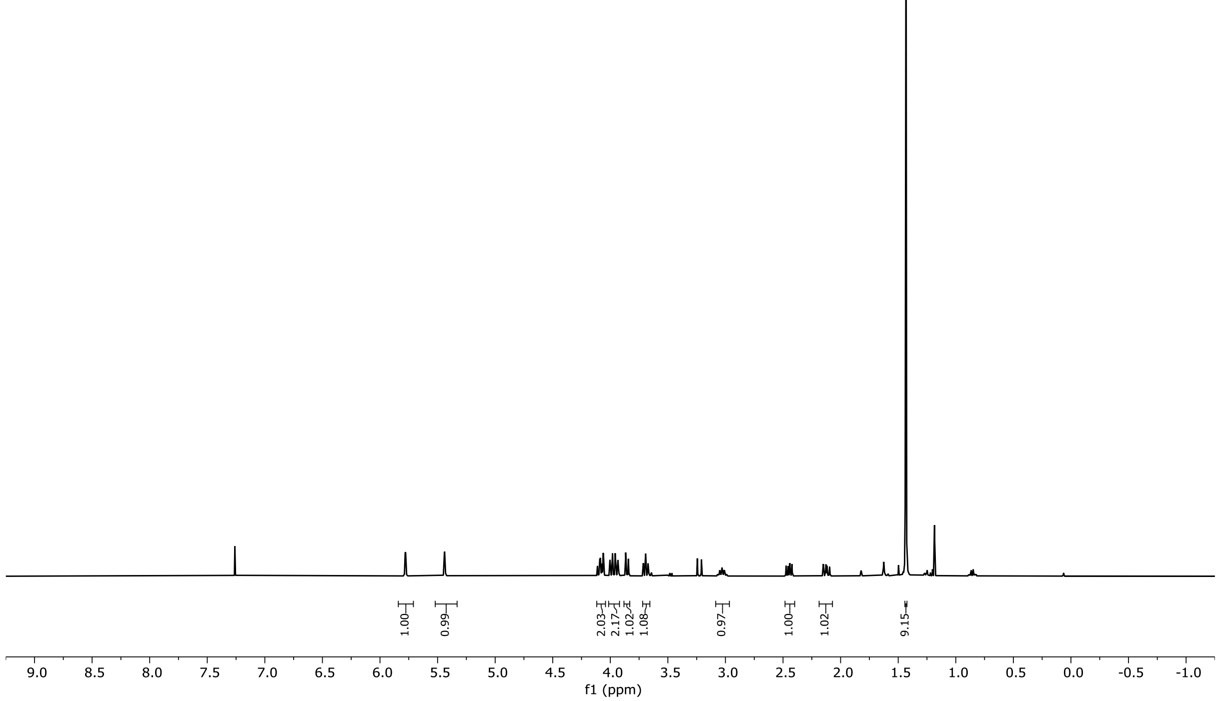

^{13}C NMR (101 MHz, $CDCl_3$)

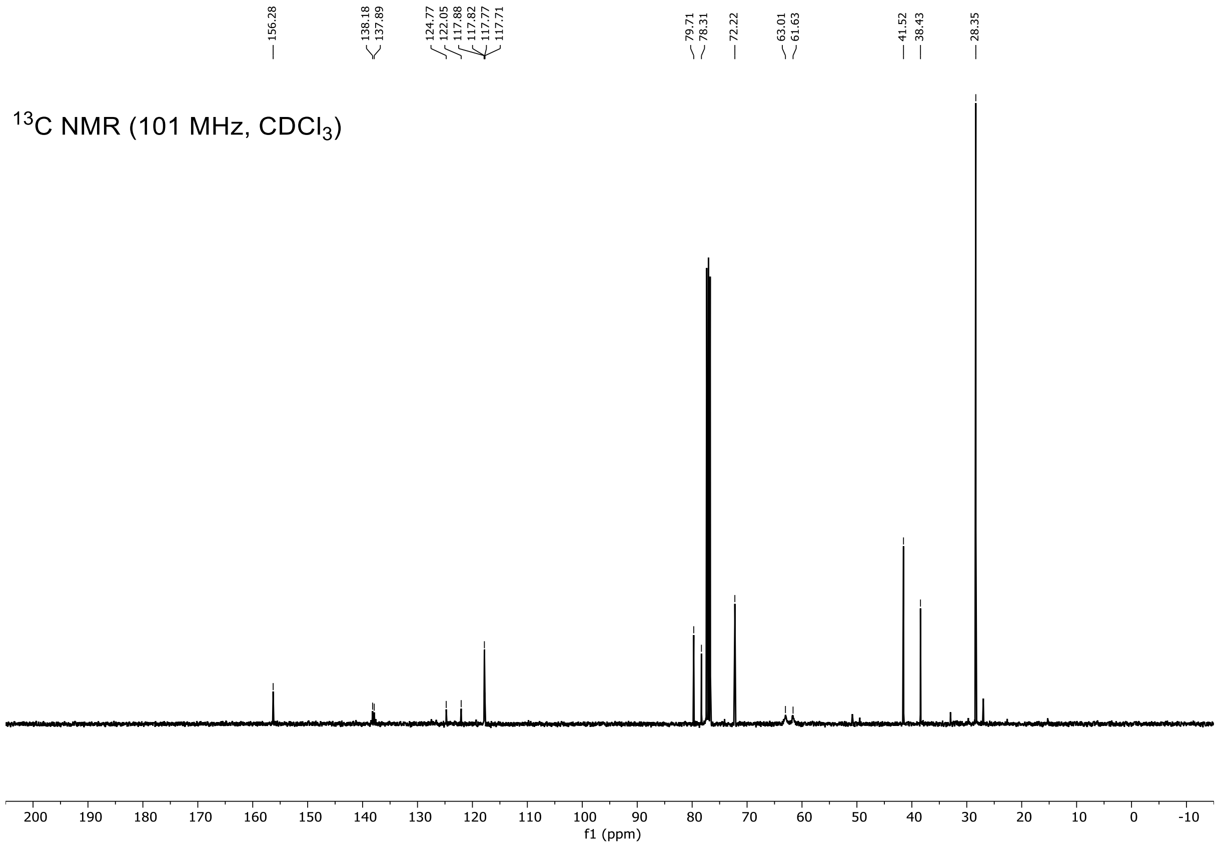

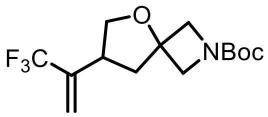

S52

^{19}F NMR (282 MHz, $CDCl_3$)

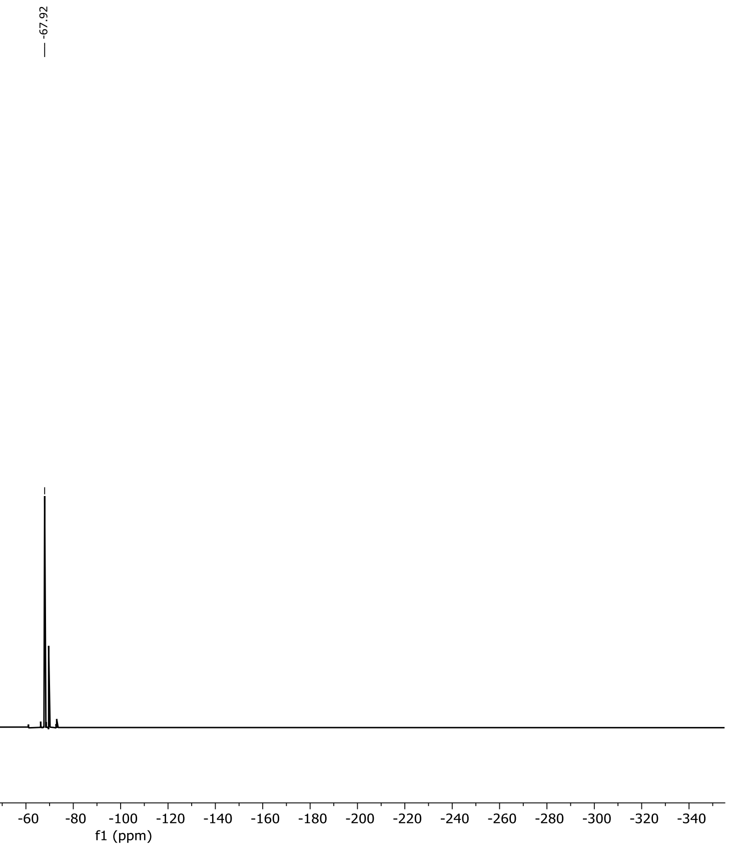

Exemplary Downstream Functionalization

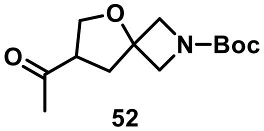

52

^{1}H NMR (400 MHz, $CDCl_3$)

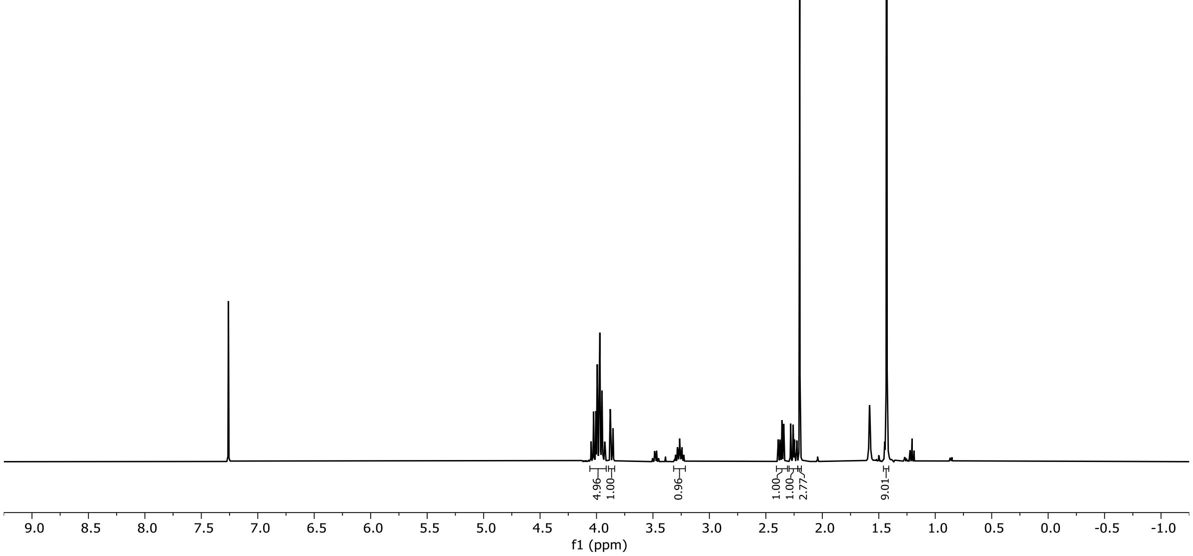

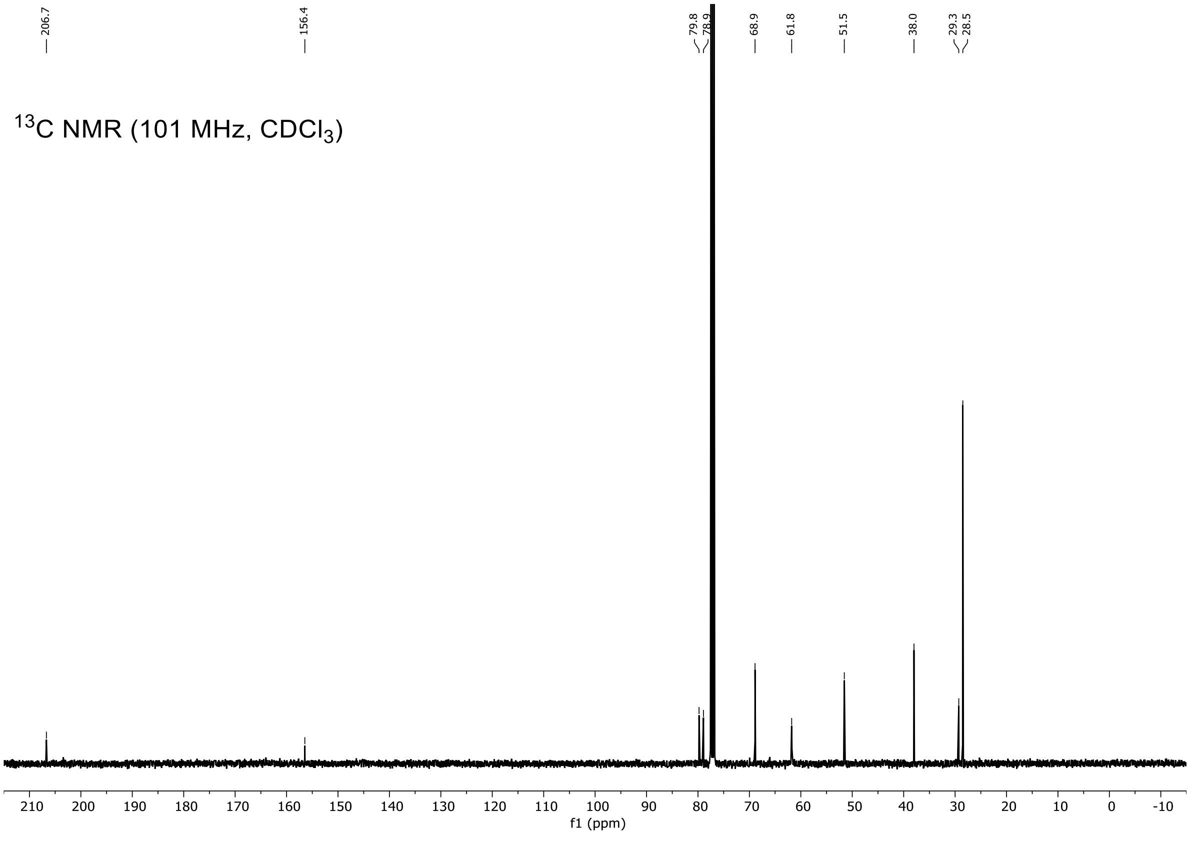

^{13}C NMR (101 MHz, $CDCl_3$)

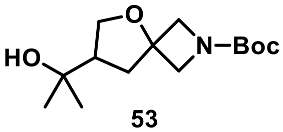

53

^{1}H NMR (400 MHz, $CDCl_3$)

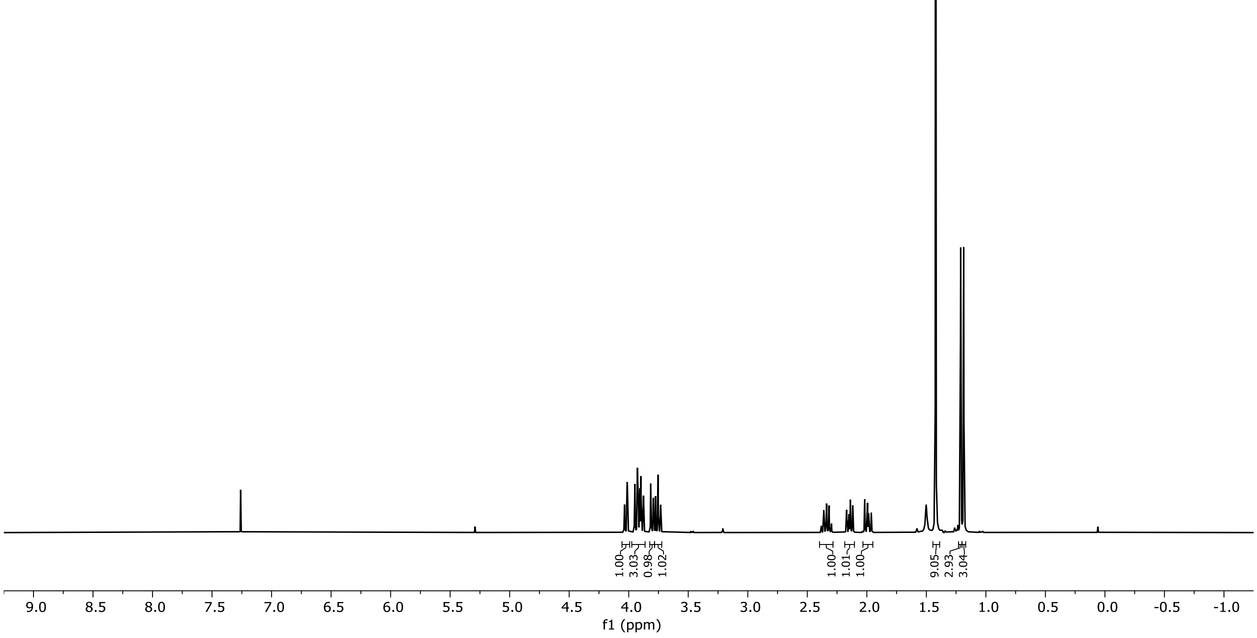

^{13}C NMR (101 MHz, $CDCl_3$)

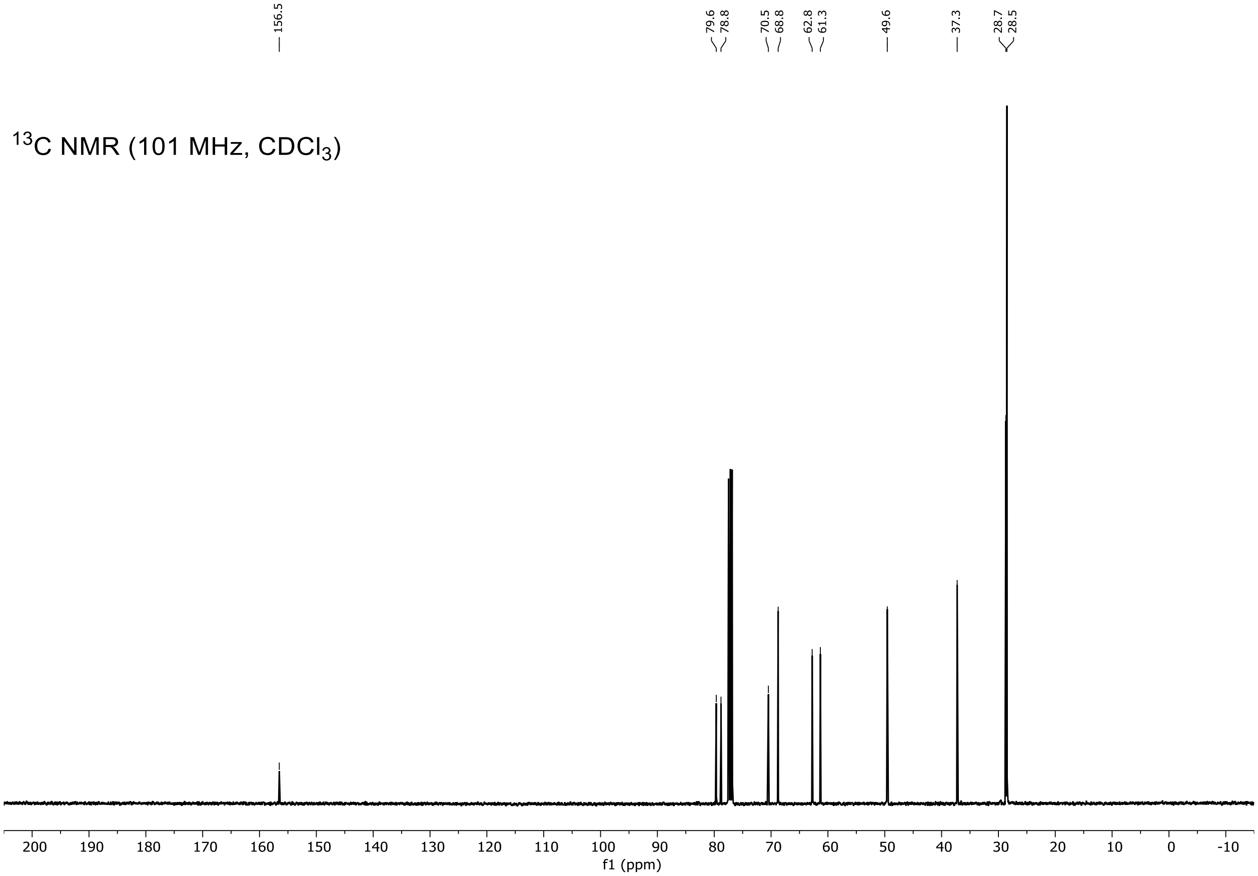

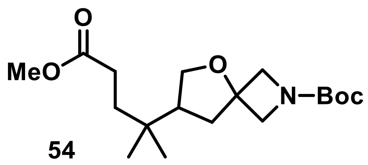

54

^{1}H NMR (400 MHz, $CDCl_3$)

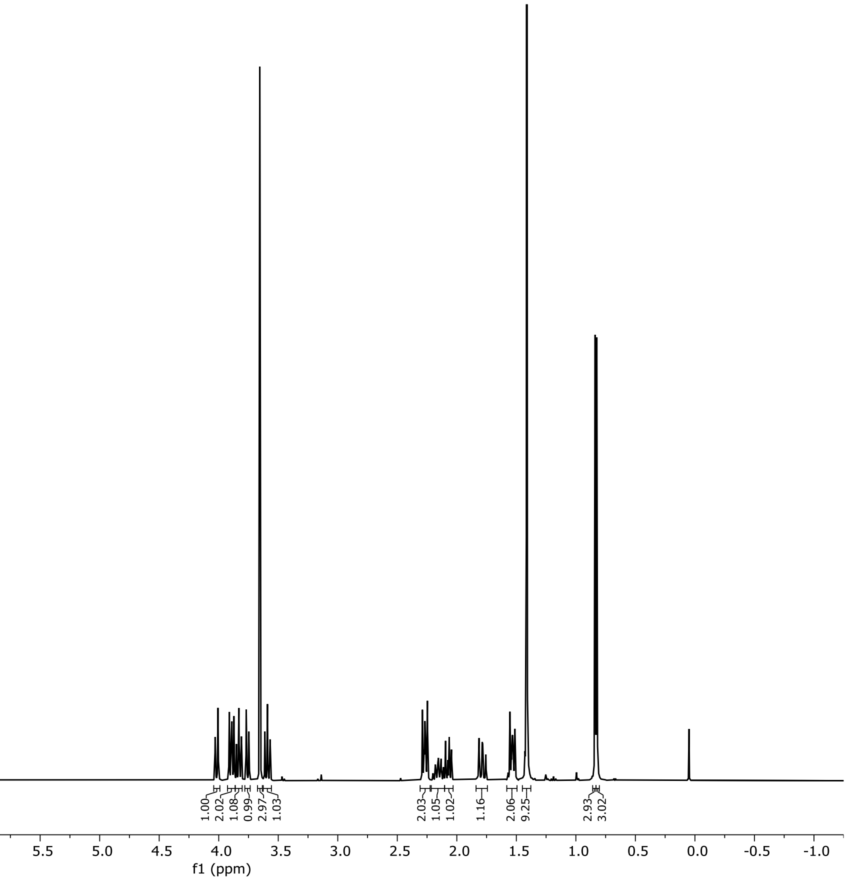

^{13}C NMR (101 MHz, $CDCl_3$)

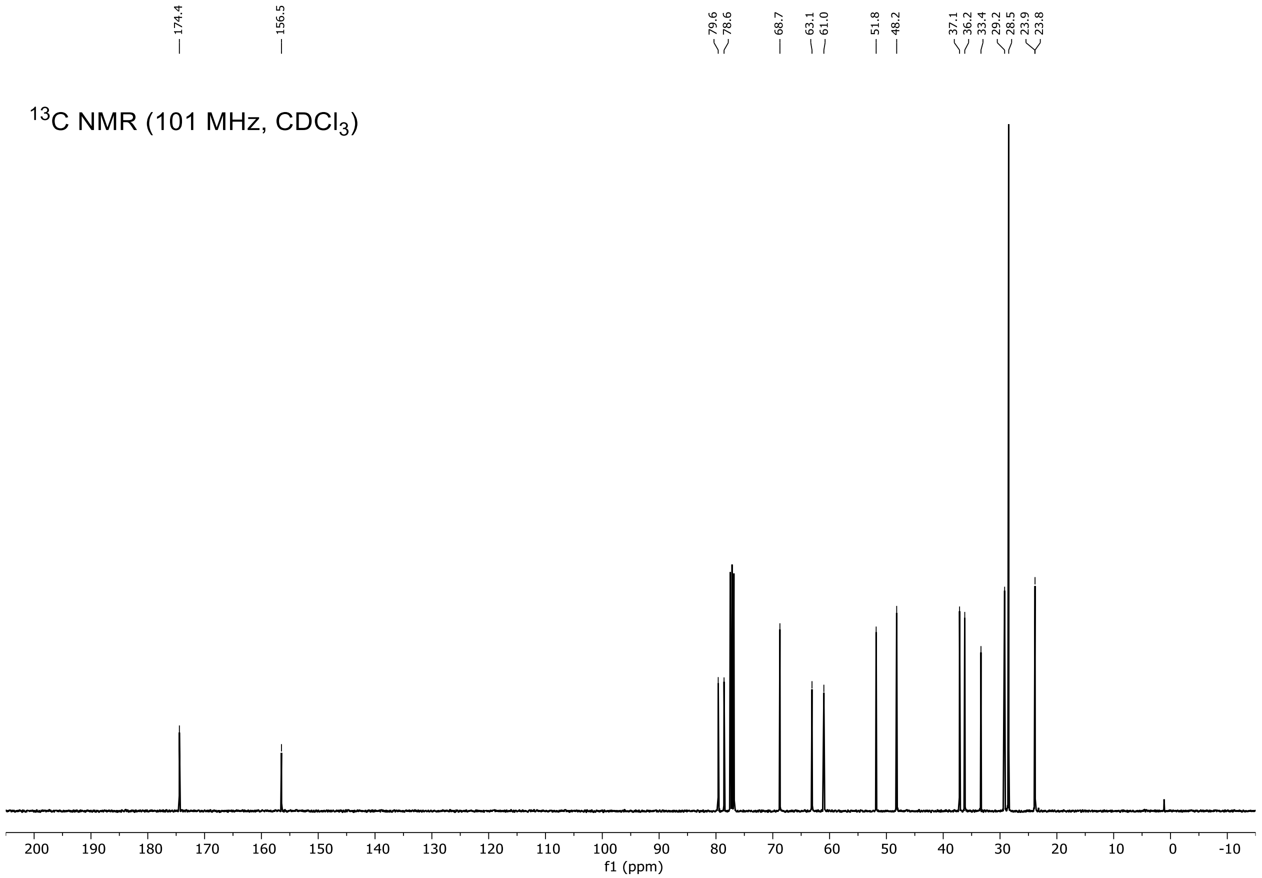

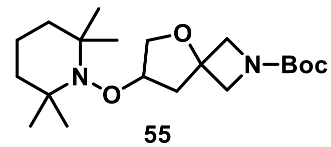

55

^{1}H NMR (400 MHz, $CDCl_3$)

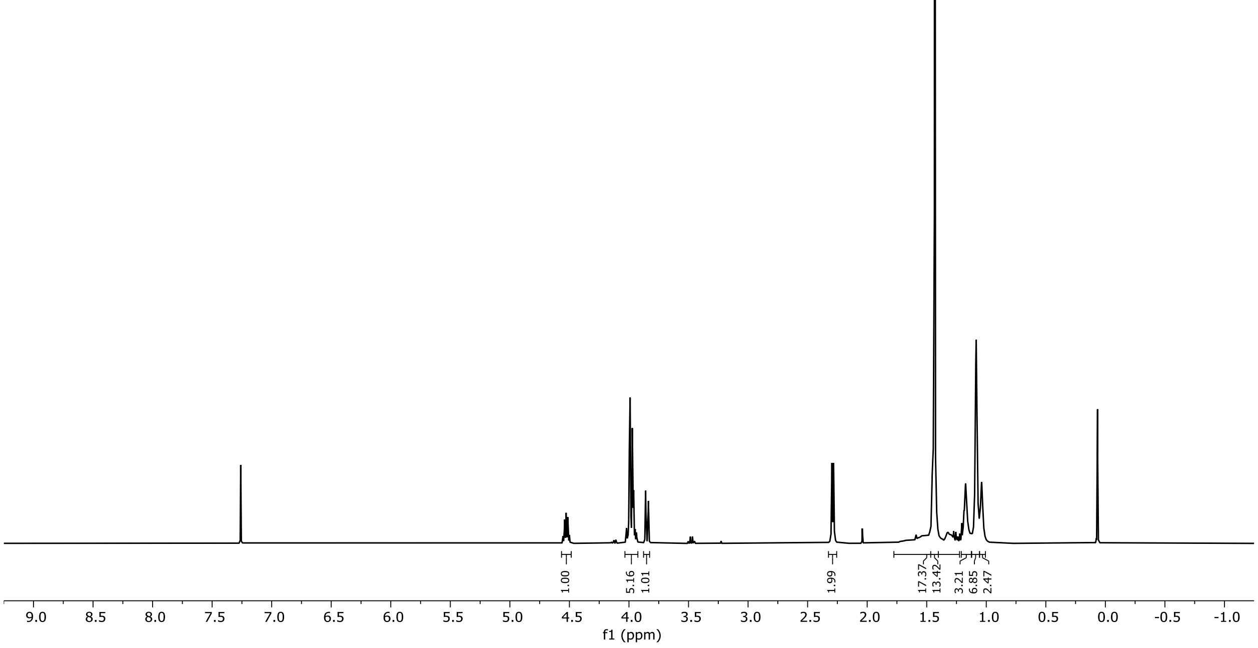

^{13}C NMR (101 MHz, $CDCl_3$)

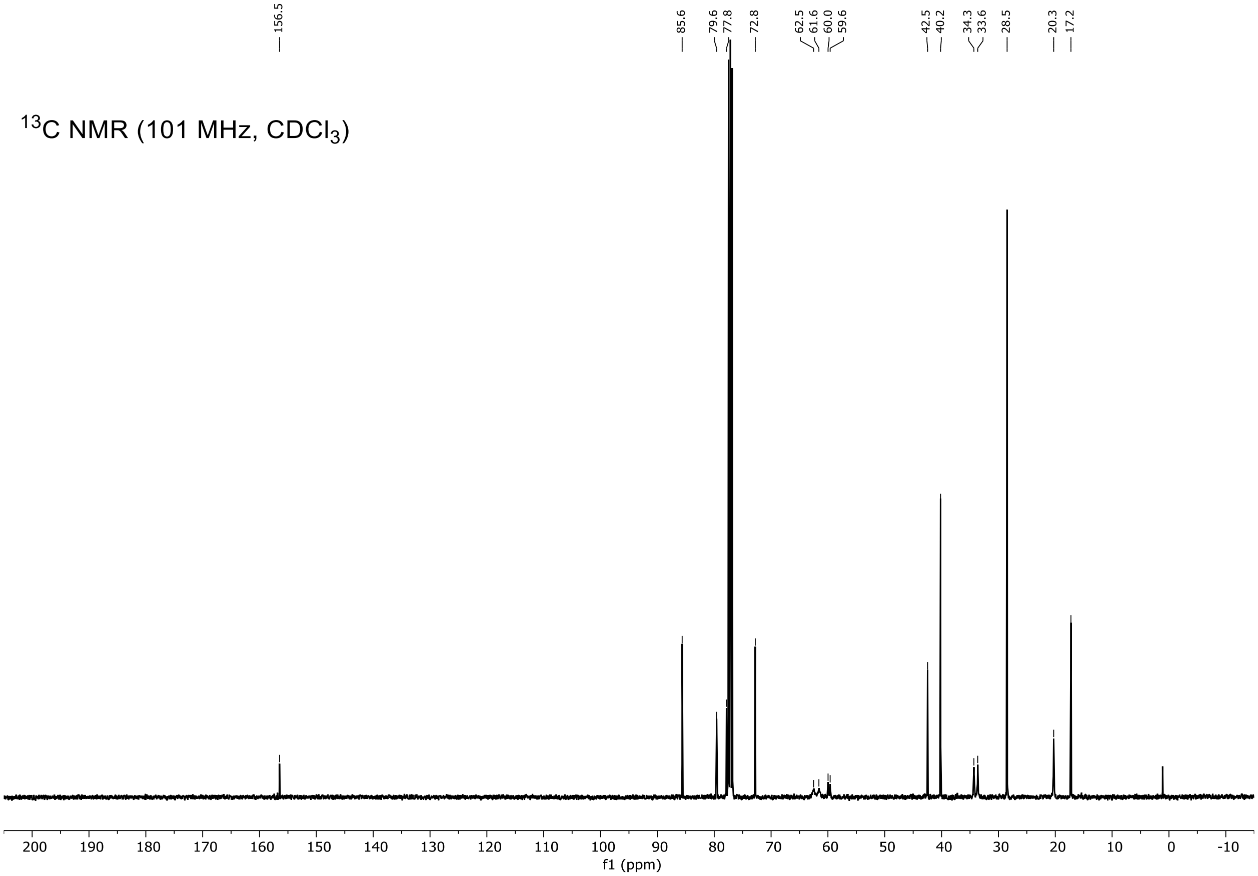

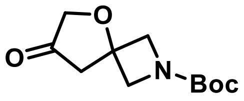

56

^{1}H NMR (400 MHz, $CDCl_3$)

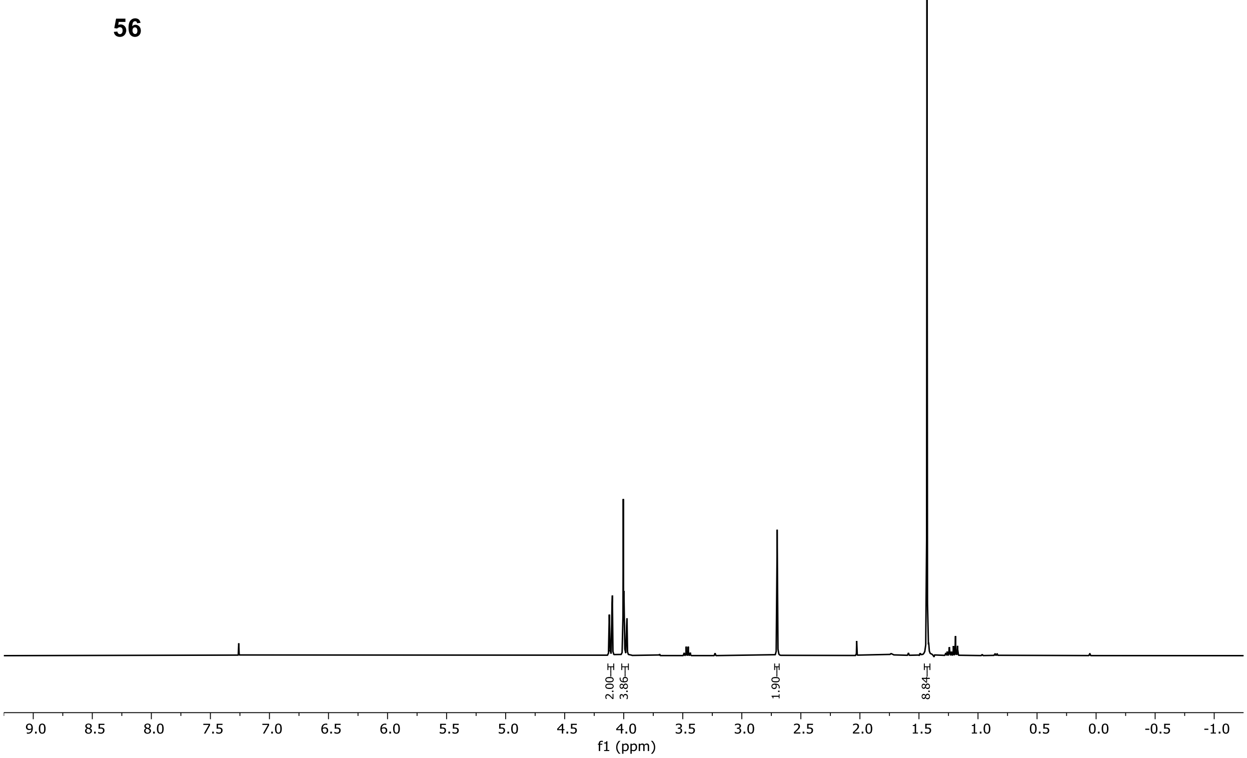

^{13}C NMR (101 MHz, $CDCl_3$)

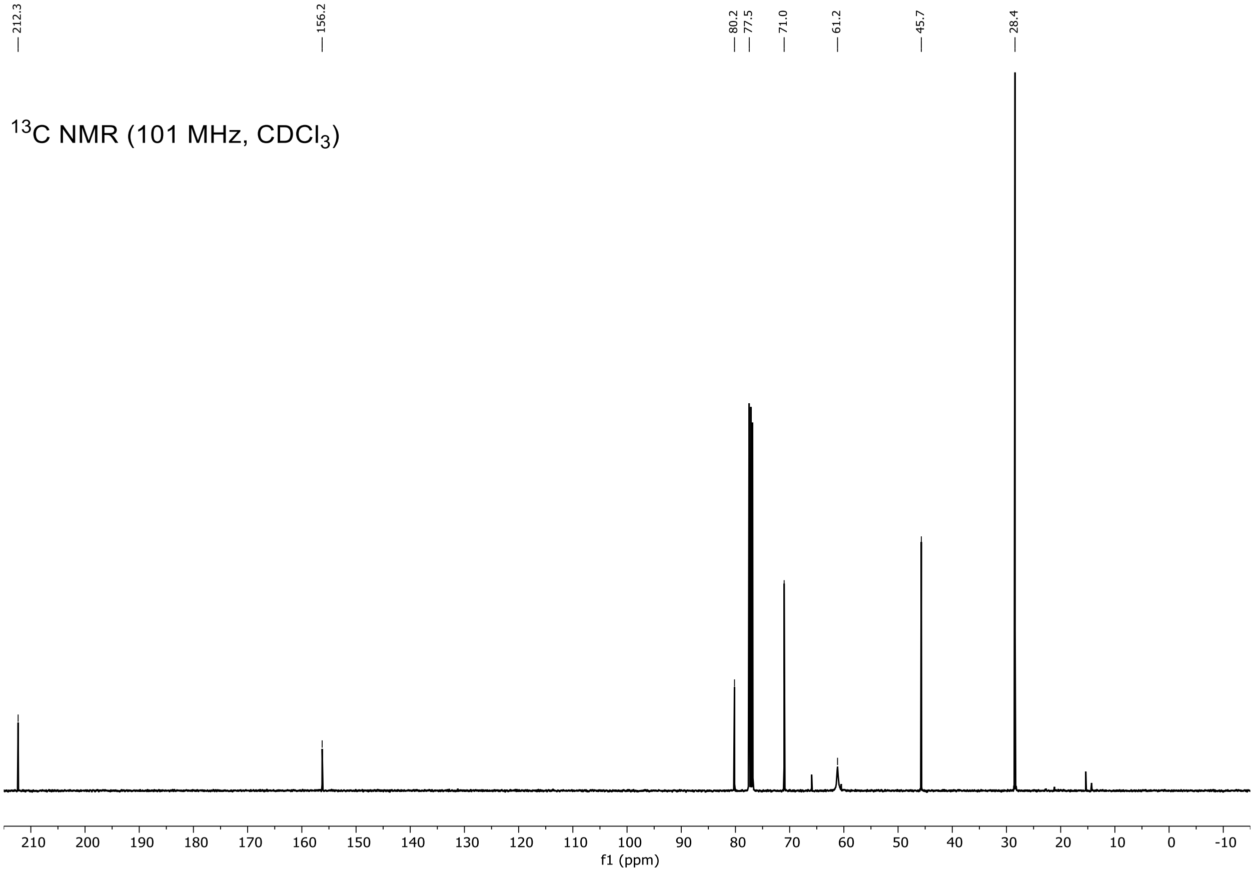

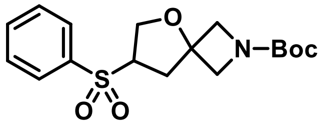

57

^{1}H NMR (400 MHz, $CDCl_3$)

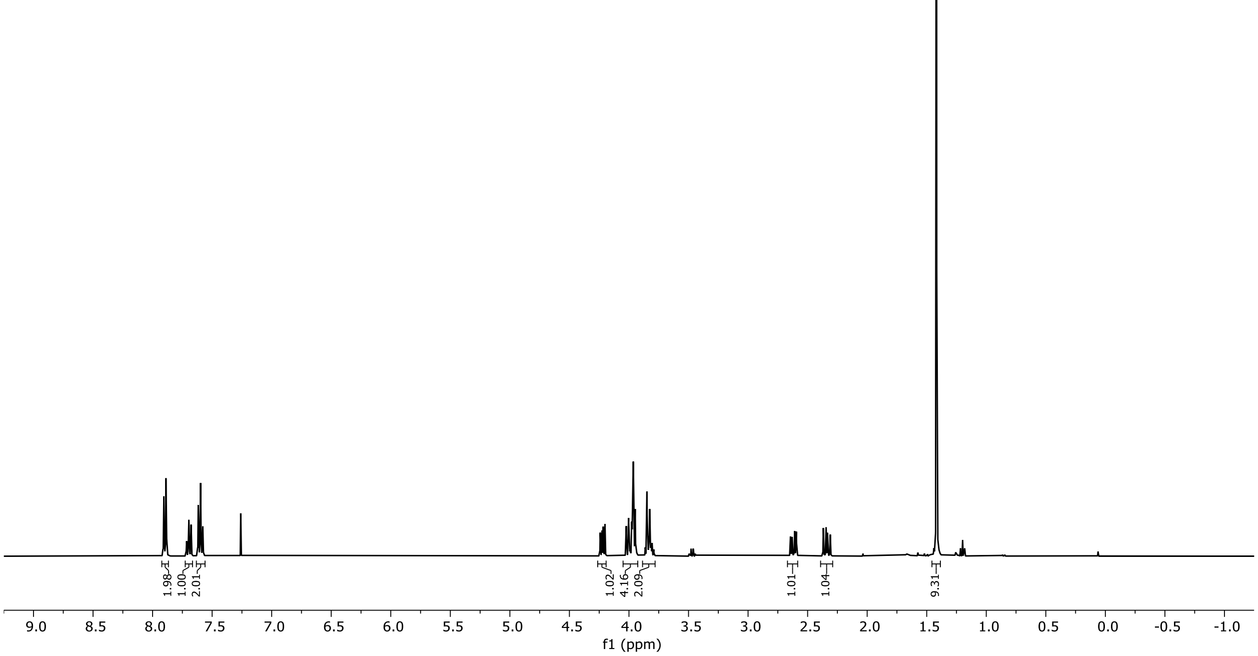

^{13}C NMR (101 MHz, $CDCl_3$)

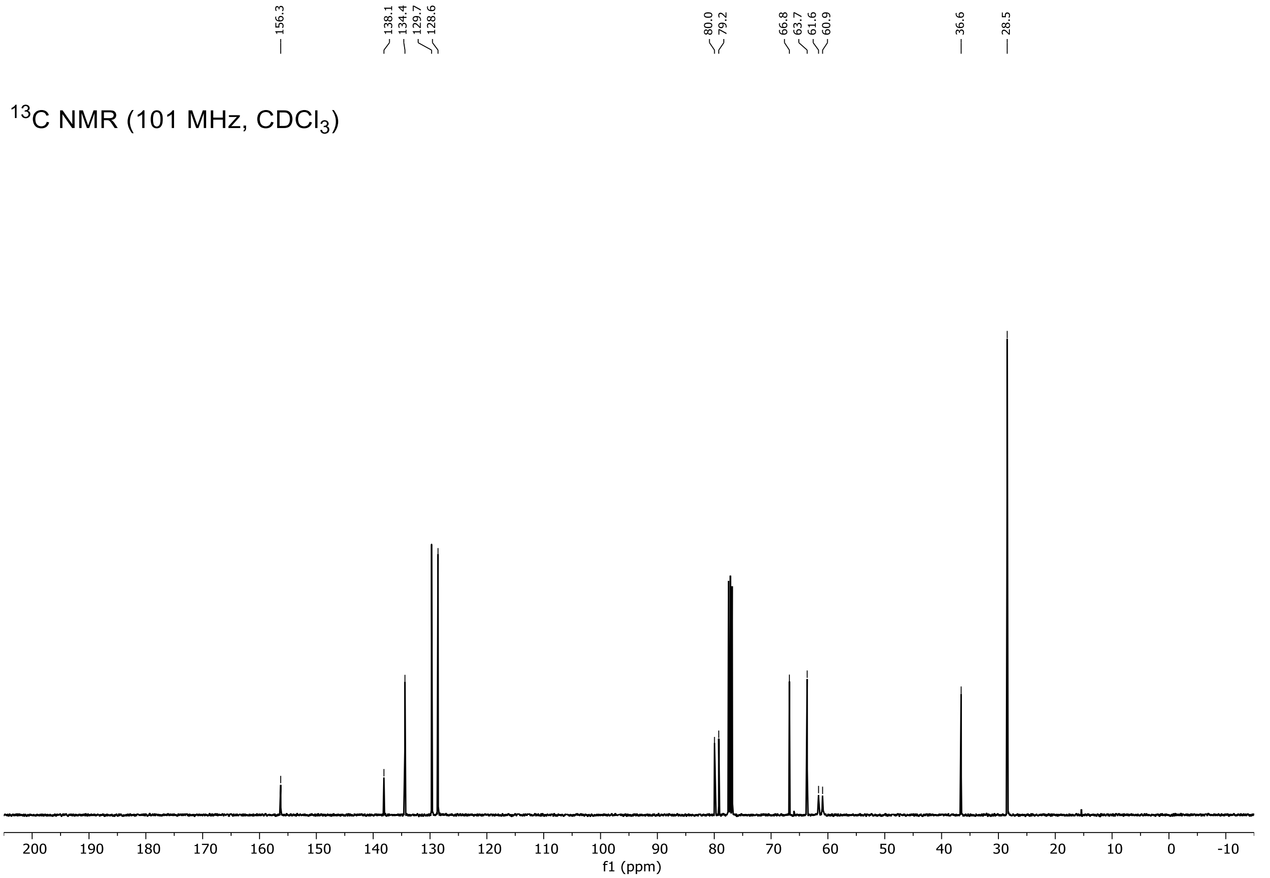

References

1. Aguilar, J. A.; Elliott, P. I. P.; López-Serrano, J.; Adams, R. W.; Duckett, S. B., Only *para*-Hydrogen Spectroscopy (OPSY), a Technique for the Selective Observation of *para*-Hydrogen Enhanced NMR Signals. *Chem. Commun.* **2007,** (11), 1183-1185.

2. Aguilar, J. A.; Adams, R. W.; Duckett, S. B.; Green, G. G. R.; Kandiah, R., Selective Detection of Hyperpolarized NMR Signals Derived from *para*-Hydrogen using the Only *Para*-Hydrogen SpectroscopY (OPSY) Approach. *J. Magn. Res.* **2011,** *208* (1), 49-57.

3. Fagan, P. J.; Mahoney, W. S.; Calabrese, J. C.; Williams, I. D., Structure and Chemistry of the Complex Tetrakis(η^5-pentamethylcyclopentadienyl)tetrakis(μ^3-chloro)tetraruthenium(II): A Useful Precursor to (Pentamethylcyclopentadienyl)ruthenium(0), -(II), and -(IV) Complexes. *Organometallics* **1990,** *9* (6), 1843-1852.

4. Biberger, T.; Gordon, C. P.; Leutzsch, M.; Peil, S.; Guthertz, A.; Copéret, C.; Fürstner, A., Alkyne *gem*-Hydrogenation: Formation of Pianostool Ruthenium Carbene Complexes and Analysis of Their Chemical Character. *Angew. Chem. Int. Ed.* **2019,** *58*, 8845-8850.

5. Kwart, H., Temperature dependence of the primary kinetic hydrogen isotope effect as a mechanistic criterion. *Acc. Chem. Res.* **1982,** *15* (12), 401-408.

6. Cambeiro, F.; López, S.; Varela, J. A.; Saá, C., Cyclization by Cationic Ruthenium Carbene Insertion into Csp^3–H Bonds. *Angew. Chem. Int. Ed.* **2012,** *51*, 723-727.

7. Cambeiro, F.; Martínez-Núñez, E.; Varela, J. A.; Saá, C., DFT and Kinetic Monte Carlo Study of TMS-Substituted Ruthenium Vinyl Carbenes: Key Intermediates for Stereoselective Cyclizations. *ACS Catal.* **2015,** *5*, 6255-6262.

8. Padin, D.; Varela, J. A.; Saá, C., Cp*RuCl-Vinyl Carbenes: Two Faces and the Bifunctional Role in Catalytic Processes. *Chem. - Eur. J.* **2020,** *26*, 74707478.

9. Guthertz, A.; Leutzsch, M.; Wolf, L. M.; Gupta, P.; Rummelt, S. M.; Goddard, R.; Farès, C.; Thiel, W.; Fürstner, A., Half-Sandwich Ruthenium Carbene Complexes Link *trans*-Hydrogenation and *gem*-Hydrogenation of Internal Alkynes. *J. Am. Chem. Soc.* **2018,** *140* (8), 3156-3169.

10. Li, D. B.; Rogers-Evans, M.; Carreira, E. M., Construction of Multifunctional Modules for Drug Discovery: Synthesis of Novel Thia/Oxa-Azaspiro[3.4]octanes. *Org. Lett.* **2013,** *15* (18), 4766-4769.

11. Liu, W.; Li, W.; Spannenberg, A.; Junge, K.; Beller, M., Iron-catalysed regioselective hydrogenation of terminal epoxides to alcohols under mild conditions. *Nat. Catal.* **2019,** *2* (6), 523-528.

12. Grandclaudon, C.; Michelet, V.; Toullec, P. Y., Synthesis of Polysubstituted 2-Iodoindenes via Iodonium-Induced Cyclization of Arylallenes. *Org. Lett.* **2016,** *18* (4), 676-679.

13. Wagner, C. E.; Shea, K. J., Synthesis and First Molecular Structure of a Bis-2-spiro-1-boraadamantane Derivative. *Org. Lett.* **2004,** *6* (3), 313-316.

14. Cordonnier, M.-C.; Blanc, A.; Pale, P., Gold(I)-Catalyzed Rearrangement of Alkynyloxiranes: A Mild Access to Divinyl Ketones. *Org. Lett.* **2008,** *10* (8), 1569-1572.

15. Kim, S.; Chung, Y. K., Rhodium-Catalyzed Carbonylative [3 + 2 + 1] Cycloaddition of Alkyne-Tethered Alkylidenecyclopropanes to Phenols in the Presence of Carbon Monoxide. *Org. Lett.* **2014,** *16* (17), 4352-4355.

16. Gao, D.; O'Doherty, G. A., Total Synthesis of Fostriecin: Via a Regio- and Stereoselective Polyene Hydration, Oxidation, and Hydroboration Sequence. *Org. Lett.* **2010,** *12* (17), 3752-3755.

17. Basil, L. F.; Nakano, H.; Frutos, R.; Kopach, M.; Meyers, A. I., Asymmetric Tandem Additions to Chiral 2,3-Dihydronaphthyloxazolines: Synthesis of the Triptoquinone/Triptinin A Ring System. *Synthesis* **2002,** 2064-2074.

18. Tanaka, Y.; Kanai, M.; Shibasaki, M., Catalytic Enantioselective Construction of β-Quaternary Carbons via a Conjugate Addition of Cyanide to β,β-Disubstituted α,β-Unsaturated Carbonyl Compounds. *J. Am. Chem. Soc.* **2010,** *132* (26), 8862-8863.

19. Kern, N.; Blanc, A.; Miaskiewicz, S.; Robinette, M.; Weibel, J.-M.; Pale, P., Coinage Metals-Catalyzed Cascade Reactions of Aryl Alkynylaziridines: Silver(I)-Single vs Gold(I)-Double Cyclizations. *J. Org. Chem.* **2012,** *77* (9), 4323-4341.

20. McMurry, J. E.; Matz, J. R.; Kees, K. L., Synthesis of Macrocyclic Terpenoids by Intramolecular Carbonyl Coupling: Flexibilene and Humulene. *Tetrahedron* **1987,** *43* (23), 5489-5498.

21. Lo, J. C.; Kim, D.; Pan, C.-M.; Edwards, J. T.; Yabe, Y.; Gui, J.; Qin, T.; Gutiérrez, S.; Giacoboni, J.; Smith, M. W.; Holland, P. L.; Baran, P. S., Fe-Catalyzed C–C Bond Construction from Olefins via Radicals. *J. Am. Chem. Soc.* **2017,** *139* (6), 2484-2503.

22. Smaligo, A. J.; Swain, M.; Quintana, J. C.; Tan, M. F.; Kim, D. A.; Kwon, O., Hydrodealkenylative C(sp^3)–C(sp^2) Bond Fragmentation. *Science* **2019,** *364* (6441), 681.

23. Smaligo, A. J.; Wu, J.; Burton, N. R.; Hacker, A. S.; Shaikh, A. C.; Quintana, J. C.; Wang, R.; Xie, C.; Kwon, O., Oxodealkenylative Cleavage of Alkene C(sp^3)−C(sp^2) Bonds: A Practical Method for Introducing Carbonyls into Chiral Pool Materials. *Angew. Chem. Int. Ed.* **2020,** *59* (3), 1211-1215.

24. Smaligo, A. J.; Kwon, O., Dealkenylative Thiylation of C(sp^3)–C(sp^2) Bonds. *Org. Lett.* **2019,** *21* (21), 8592-8597.
